# Synthesis of Functionalized 3*H*-pyrrolo-[1,2,3-*de*] Quinoxalines via Gold-Catalyzed Intramolecular Hydroamination of Alkynes

**DOI:** 10.3390/molecules28155831

**Published:** 2023-08-02

**Authors:** Antonia Iazzetti, Giancarlo Fabrizi, Antonella Goggiamani, Federico Marrone, Alessio Sferrazza, Karim Ullah

**Affiliations:** 1 Dipartimento di Scienze Biotecnologiche di Base, Cliniche Intensivologiche e Perioperatorie, Università Cattolica del Sacro Cuore, L.go Francesco Vito 1, 00168 Rome, Italy; antonia.iazzetti@unicatt.it; 2 Policlinico Universitario ‘A. Gemelli’ Foundation-IRCCS, 00168 Rome, Italy; 3 Dipartimento di Chimica e Tecnologie del Farmaco, Sapienza, Università di Roma, P. le A. Moro 5, 00185 Rome, Italy; antonella.goggiamani@uniroma1.it (A.G.); federico.marrone@uniroma1.it (F.M.); karim.ullah@uniroma1.it (K.U.); 4 Medicinal Chemistry Department, IRBM S.p.A., 00071 Pomezia, Italy

**Keywords:** 3*H*-pyrrolo-[1,2,3-*de*] quinoxalines, gold catalysis, quinoxalines, hydroamination

## Abstract

A gold-catalyzed protocol to obtain functionalized 3*H*-pyrrolo [1,2,3-*de*] quinoxalines from suitable substituted *N-alkynyl* indoles has been proposed. The mild reaction conditions were revealed to be compatible with different functional groups, including halogen, alkoxyl, cyano, ketone, and ester, allowing the isolation of title compounds with yields from good to high. A reaction mechanism has been proposed, and theoretical calculations have been provided to rationalize the final step of the hypothesized reaction mechanism. As quinoxaline-containing polycyclic compounds, this class of molecules may represent a valuable template in medicinal chemistry and material science.

## 1. Introduction

Nitrogen-containing heterocycles are a class of compounds of great importance to life science since they are present as scaffolds in several biologically active natural products and synthetic drugs [1]. For this reason, a great deal of attention has been devoted to the development of methods for their preparation and, in particular, to those catalytic protocols that overcome the limitations of traditional C-N bond-forming reactions. In this regard, the transition metal-catalyzed hydroamination assumed great significance [2], with a lot of expedient routes based on gold catalysis [2,3,4,5].

**Scheme 1:**
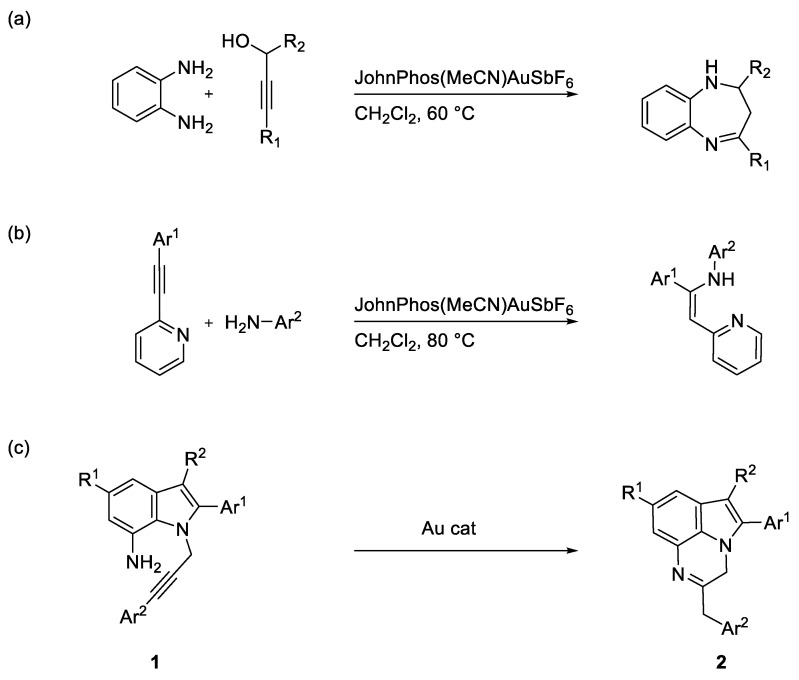
(**a**) Our previous work reporting on the Au-catalyzed synthesis of 1,5-benzodiazepines from *o*-phenylenediamines and propargylic alcohols. (**b**) Our previous work reporting on the stereo- and regioselective synthesis of enamines via the Au(I)-catalyzed hydroamination of 2-(Arylethynyl)pyridines with anilines. (**c**) Our work hypothesis for the gold-catalyzed synthesis of functionalized 3*H*-pyrrolo-[1,2,3-*de*] quinoxalines from substituted *N-alkynyl* indoles.

We previously described a domino approach to 4-substituted 1,5-benzodiazepines based on the reactive sequence gold-catalyzed hydroamination/cyclization [6] (Scheme 1a), as well as a stereo and regioselective approach to *Z*-enamine products via an intermolecular gold-catalyzed reaction of the 2-(arylethynyl)pyridines with anilines [7] (Scheme 1b). Continuing our investigations in this research field, we focused on the intra-molecular gold-catalyzed hydroamination as a tool for the construction of condensed polycyclic structures, envisaging the possibility of obtaining the 3*H*-pyrrolo-[1,2,3-*de*] quinoxalines **2** starting from suitable substituted *N*-alkynyl indoles **1** (Scheme 1c).

**Figure 1:**
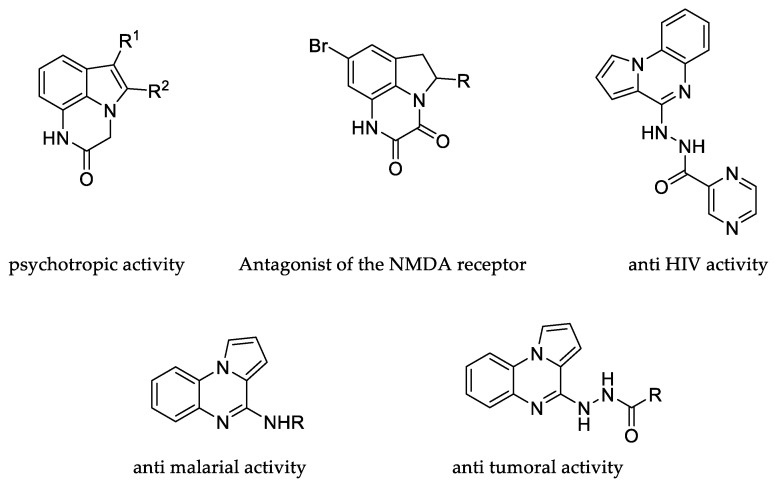
Bioactive synthetic products containing a tricyclic quinoxaline-containing scaffold.

To the best of our knowledge, the derivatives of which the synthesis was pursued are unknown compounds with a rather infrequently reported heterocyclic core [8,9]. Extensive state-of-the-art studies revealed a lack of methods to achieve their construction, even though their synthesis might be of interest in medicinal chemistry. Indeed, the tricyclic quinoxaline-containing compounds are widespread in a variety of therapeutic agents such as anti-HIV [10], antiparasitic [11,12,13,14], and antitumoral [15] (Figure 1), and make our new condensed cyclic systems promising candidates for diverse uses.

**Figure 2:**
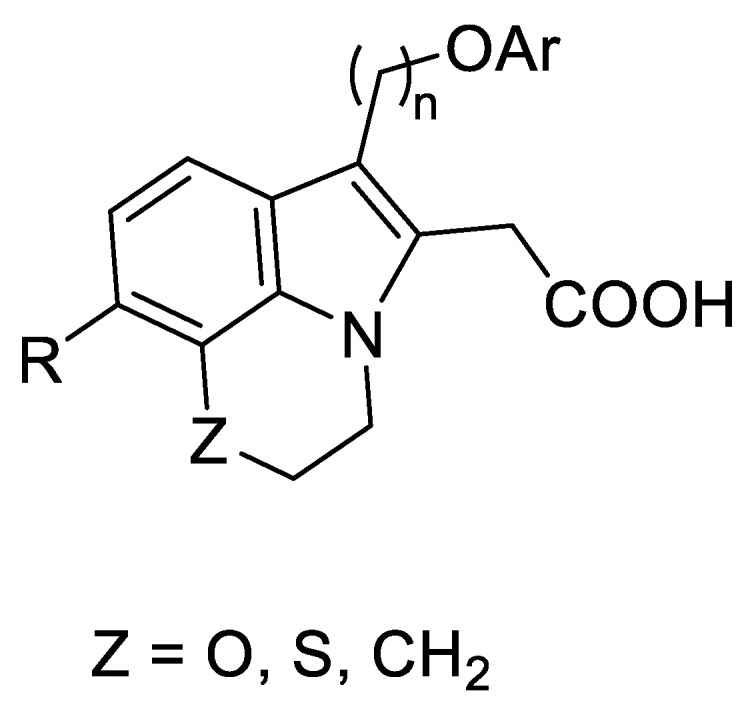
Structure of known Mcl-1 inhibitors.

In addition, the 3*H*-pyrrolo-[1,2,3-*de*] quinoxalines **2** may be structurally related to a class of compounds with antiapoptotic activity acting as potent inhibitors of the Mcl-1 protein (Figure 2) [16].

As to industrial applications, substituted quinoxalines and their derivatives are known as metal corrosion inhibitors and are often found as constituents of electroluminescent materials [17].

Given this broad range of applications of quinoxaline derivatives and the gap of synthetic methodology for the 3*H*-pyrrolo-[1,2,3-*de*] quinoxalines, we decided to study the cyclization of the indole derivatives **1,** which were suitably designed to provide an intramolecular gold-catalyzed hydroamination reaction. The proposed methodology is strongly based on the background of the research group, which has been continuously devoted to the construction and functionalization of indoles.

## 2. Results and Discussion

**Scheme 2:**
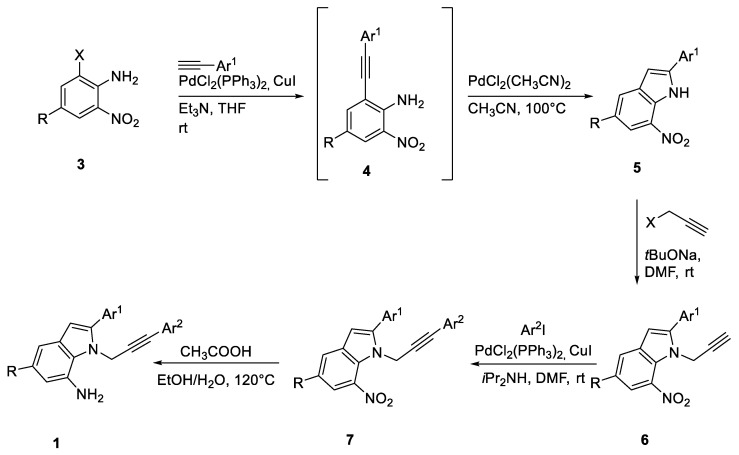
Synthesis of substrates **1**.

Substrates for our studies have been synthesized according to slightly modified known procedures depicted in the following Scheme 2 (for a detailed description of the procedures, see Materials and Methods (Section 3)).

Based on the working hypothesis (Scheme 1c), the reaction of substrate **1a** was selected as the model system for a series of preliminary experiments aimed at identifying the best reaction conditions. For our first attempt, we decided to perform the reaction under the same condition previously used for the synthesis of 1,5-benzodiazepines [6]. Pleasingly, a smooth gold-catalyzed intramolecular hydroamination of **1a** took place, and the 6-*exo-dig* cyclization product **2a** was isolated in almost quantitative yield after 1 h (Table 1, entry 1).

Given the high reaction rate, we decided to carry out the reaction at room temperature, obtaining similar results in terms of yield even though with a longer reaction time (Table 1, entry 2). A lower yield (42%) was obtained by switching to the PPh_3_AuCl/AgSbF_6_ combination (Table 1, entry 3), with a significant amount of starting material (55%) recovered after 24 h. The reaction was also performed in the presence of other transition metal catalysts leading to worse results in terms of efficiency, time, and harsher reaction conditions. Indeed, the use of PtCl_2_ resulted in a slower and less efficient cyclization at 80 °C (Table 1, entry 4), while, at the same temperature, using PdCl_2_(CH_3_CN)_2,_ the final compound **2a** was isolated in 36% yield, along with degradation compounds (Table 1, entry 5). Switching to the Brønsted acid catalyst TsOH, poor results were observed in obtaining a 2 h mixture of degradation compounds (Table 1, entry 6). In this case, very likely, the formation of the imine derivative **2a** also occurred, but in the acidic reaction conditions, it was rapidly degraded.

Once we established the best reaction conditions, we investigated this method’s scope. Variously substituted quinoxaline derivatives were obtained in good to excellent yield both in the presence of electron-donating groups and electron-withdrawing groups (Table 2).

**Figure 3:**
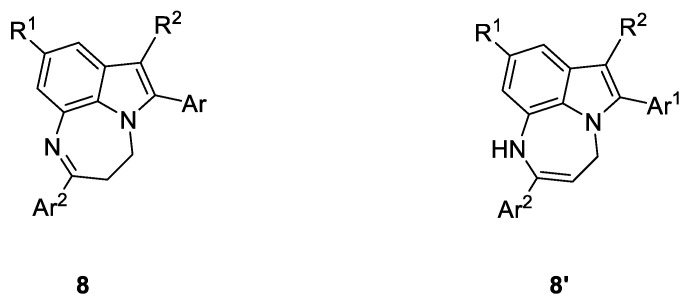
Structures of two possible isomeric 7-*endo-dig* cyclization products. The formation of these compounds was never observed.

Notably, in all experiments, compound **2** was the only observed product, with no traces of any 7-*endo-dig* cyclization product (compounds **8** and **8′**, Figure 3).

**Scheme 3:**
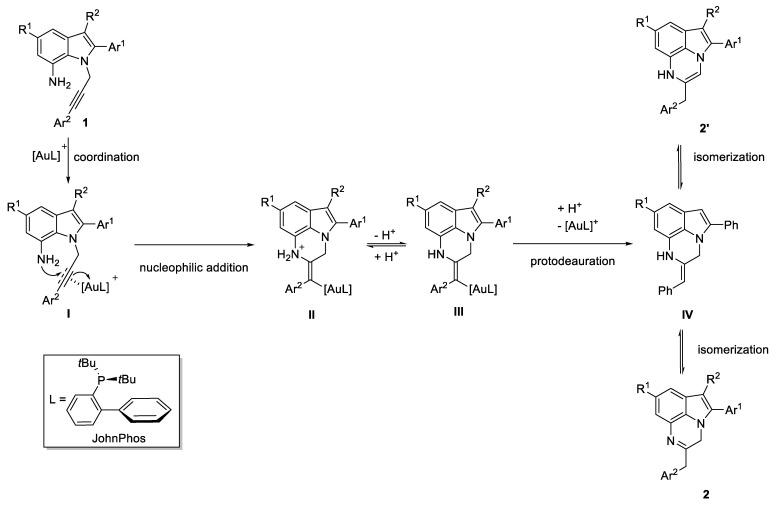
Hypothesized mechanism for the formation of **2**.

The formation of **2** may be rationalized based on the general mechanism of the gold-catalyzed hydroamination [18] through the basic steps shown in Scheme 3. Particularly, the hydroamination intermediate IV is formed by admitting the nucleophilic addition of the amine group towards the triple bond activated by the Au(I) coordination (I) followed by a protodeauration step (Scheme 3).

Then, the isomerization of IV can take place in two different modalities providing, alternatively, the imine-derivative **2** or the enamine-derivative **2′**. In the reaction conditions, this step proceeds, providing only compound **2,** which is very likely the most stable.

**Figure 4:**
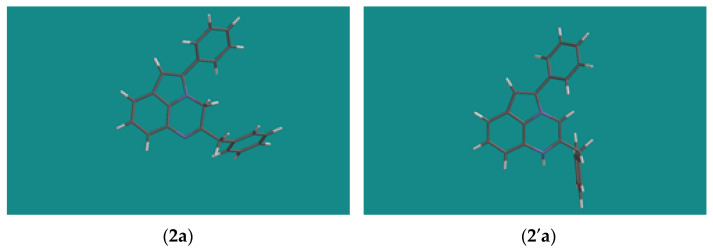
HF (6-31G**) calculations on (**2a**) and (**2′a**).

To this regard, HF (6-31G**) calculations performed on the two isomeric compounds **2a** and **2′a** revealed a higher stability of **2a** than **2′a** by 5.2 kcal/mol (Figure 4) [19] and explained the exclusive formation of imine-derivative **2** in the reaction conditions. In addition, similar isomerization modes are described in the literature [20].

**Scheme 4:**
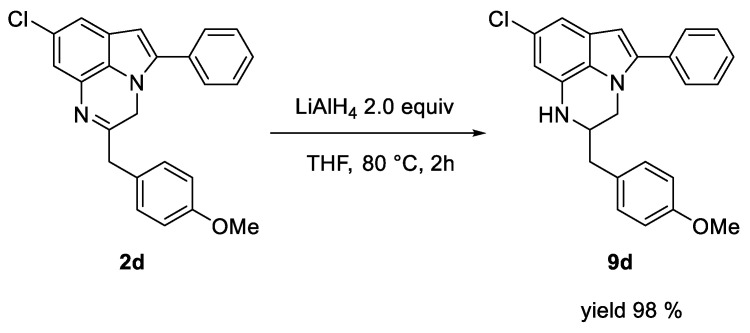
Reduction of compound **2d** to obtain **9d**.

The 5-aryl-3*H*-pyrrole [1,2,3-*de*]quinoxaline derivatives synthesized according to the proposed method are poised for further manipulations in different positions, providing access to compounds with an increased molecular complexity through simple organic reactions. For instance, as reported in Scheme 4, compound **9d** can be easily obtained in almost quantitative yield by treating **2d** with LiAlH_4_.

## 3. Materials and Methods

### 3.1. General Information

All of the commercially available reagents, catalysts, bases, and solvents were used as purchased without further purification. Starting materials and reaction products were purified by flash chromatography using SiO_2_ as a stationary phase, eluting with *n*-hexane/ethyl acetate mixtures. ^1^H NMR (400.13 MHz), ^13^C NMR (100.6 MHz), and ^19^F spectra (376.5 MHz) were recorded with a Bruker Avance 400 spectrometer. Splitting patterns are designed as s (singlet), d (doublet), t (triplet), q (quartet), m (multiplet), or bs (broad singlet). HRMS of samples were recorded on an Orbitrap Exactive (Thermo Fisher, Waltham, MA, USA). Melting points were determined with a Büchi B-545 apparatus and are uncorrected.

### 3.2. General Experimental Procedures

#### 3.2.1. Synthetic Procedures for Starting Materials

##### General Procedure for the Preparation of Substituted 1-(3-Arylprop-2-yn-1-yl)-2-aryl-1*H*-indol-7-amine 1

Starting materials **1** were prepared according to literature procedures through the four-step sequence of reactions depicted in Scheme 2.

- Typical Procedure for the Preparation of 5-substituted-7-nitro-2-phenyl-1*H*-indole **5**

STEP 1: Synthesis of 5-chloro-7-nitro-2-phenyl-1H-indole **5a**.

In a 100 mL two-necked round bottom flask equipped with a magnetic stirring bar, PdCl_2_(PPh_3_)_2_ (0.329 g, 0.469 mmol, 0.04 equiv.) and CuI (0.045 g, 0.234 mmol, 0.02 equiv.) were dissolved in 36.0 mL of THF and 1.56 mL of Et_3_N at room temperature under a nitrogen atmosphere. Then, 2-iodo-4-chloro-6-nitroaniline (3.5 g, 11.74 mmol, 1.0 equiv.) was added, and, dropwise, phenylacetylene (1.93 mL, 17.61 mmol, 1.5 equiv.). The solution was stirred for 2 h. After this time, the reaction mixture was diluted with Et_2_O and washed with a saturated solution of NH_4_Cl, NaHCO_3,_ and brine. The organic layer was separated, dried over Na_2_SO_4_, filtered, and concentrated under reduced pressure. The residue, containing 4-chloro-2-nitro-6-(phenylethynyl)aniline **4a**, was transferred with 60 mL of MeCN in a two-necked 100 mL round bottom flask equipped with a condenser, and a magnetic stirring bar, then, PdCl_2_(CH_3_CN)_2_ was added. The solution was stirred for 2.5 h at 100 °C. After this time, the mixture was cooled to room temperature, concentrated under reduced pressure, purified by chromatography on SiO_2_ (25–40 μm), eluting with a 92/8 (*v*/*v*) *n*-hexane/ethyl acetate mixture (R*_f_* = 0.26) to obtain 5-chloro-7-nitro-2-phenyl-1*H*-indole **5a** (2,57 g, 80% yield).

The 5-chloro-7-nitro-2-phenyl-1*H*-indole **5a**: yield: 80%; orange solid; mp: 164–166 °C; ^1^H NMR (400.13 MHz) (CDCl_3_): δ 10.07 (bs, 1 H), 8.11 (d, *J* = 1.5 Hz, 1 H), 7.93–7.88 (m, 1 H), 7.74 (d, *J* = 7.8 Hz, 1 H), 7.53 (t, *J* = 7.3 Hz, 2 H), 7.45 (t, *J* = 7.3 Hz, 2 H), 6.90 (d, *J* = 2.4 Hz, 1 H), 5.05 (d, *J* = 2.4 Hz, 2 H), and 2.20 (t, *J* = 2.4 Hz, 1 H); ^13^C NMR (100.6 MHz) (CDCl_3_): δ 142.0 (C), 133.7 (C), 133.5 (C), 130.4 (C), 129.34 (CH), 129.27 (CH), 128.8 (C), 127.6 (CH), 125.7 (CH), 125.1 (C), 118.7 (CH), and 100.1 (CH).

b. Typical Procedure for the Preparation of Substituted 7-nitro-2-phenyl-1-(prop-2-yn-1-yl)-1*H*-indoles **6**.

STEP 2: Synthesis of 5-chloro-7-nitro-2-phenyl-1-(prop-2-yn-1-yl)-1*H*-indole **6a**.

A 250 mL round bottom flask, equipped with a magnetic stirring bar, was charged with *t*-BuONa (1.35 g, 14.02 mmol, 1.5 equiv) and 90 mL of anhydrous DMF. The reaction mixture was cooled at 0 °C, and 5-chloro-7-nitro-2-phenyl-1*H*-indole (2.4 g, 9.35 mmol, 1.0 equiv) was added dropwise. Then, propargyl bromide (1.21 mL, 14.02 mmol, 1.5 equiv) was added, and the solution was warmed to room temperature and stirred for 6 h. After this time, the reaction mixture was diluted with Et_2_O and washed with a saturated solution of NaHCO_3_ and brine. The organic layer was dried over Na_2_SO_4_, filtered, and concentrated under reduced pressure. The residue was purified by chromatography on SiO_2_ (25–40 μm), eluting with a 96/4 (*v*/*v*) *n*-hexane/ethyl acetate mixture (R*_f_* = 0.25) to obtain 5-chloro-7-nitro-2-phenyl-1-(prop-2-yn-1-yl)-1*H*-indole **6a** (2.324 g, 80% yield).

The 5-chloro-7-nitro-2-phenyl-1-(prop-2-yn-1-yl)-1*H*-indole **6a**: 80% yield; brown solid; mp 103–104 °C; ^1^H NMR (400.13 MHz) (CDCl_3_): δ 7.92 (d, *J* = 1.9 Hz, 1 H), 7.87 (d, *J* = 1.9 Hz, 1 H), 7.58–7.50 (m, 5 H), 6.70 (s, 1 H), 5.05 (d, *J* = 2.4 Hz, 2 H), and 2.20 (t, *J* = 2.4 Hz, 1 H); ^13^C NMR (100.6 MHz) (CDCl_3_): δ 146.8 (C), 137.3 (C), 134.3 (C), 130.6 (C), 129.6 (CH), 129.4 (CH), 129.1 (CH), 127.1 (C), 126.0 (CH), 125.2 (C), 119.8 (CH), 104.1 (CH), 77.1 (C), 74.4 (CH), and 37.1 (CH_2_).

c. Typical Procedure for the Preparation of Substituted 1-(3-arylprop-2-yn-1-yl)-7-nitro-2-phenyl-1*H*-indoles **7**.

STEP 3: Synthesis of 5-chloro-1-(3-(4-methoxyphenyl)prop-2-yn-1-yl)-7-nitro-2-phenyl-1*H*-indole **7c**.

In a two-necked 50 mL round bottom flask, equipped with a magnetic stirring bar, PdCl_2_(PPh_3_)_2_ (0.084 g, 0.119 mmol, 0.04 equiv.) and CuI (0.011 g, 0.0597 mmol, 0.02 equiv.) were dissolved in 12.3 mL of *i*Pr_2_NH and 6.1 mL of DMF at room temperature and under nitrogen; then, 4-iodoanisole (0.839 g, 3.584 mmol, 1.2 equiv.) and 5-chloro-7-nitro-2-phenyl-1-(prop-2-yn-1-yl)-1*H*-indole (0.928 g, 2.98 mmol, 1.0 equiv.) were added, and the resulting mixture was stirred for 24 h. After this time, the mixture was diluted with Et_2_O and washed with a saturated solution of NH_4_Cl, a saturated solution of NaHCO_3,_ and with brine. The organic layer was dried over Na_2_SO_4_, filtered, and concentrated under reduced pressure. The residue was purified by chromatography on SiO_2_ (25–40 μm), eluting with a 93/7 (*v*/*v*) *n*-hexane/ethyl acetate mixture (R*_f_* = 0.27) to obtain 5-chloro-1-(3-(4-methoxyphenyl)prop-2-yn-1-yl)-7-nitro-2-phenyl-1*H*-indole **7c** (0.860 g, 70% yield).

The 5-chloro-1-(3-(4-methoxyphenyl)prop-2-yn-1-yl)-7-nitro-2-phenyl-1*H*-indole 7c: 70% yield; yellow solid; mp 133–134 °C; ^1^H NMR (400.13 MHz) (CDCl_3_): δ 7.91 (d, *J* = 1.9 Hz, 1 H), 7.87 (d, *J* = 1.9 Hz, 1 H), 7.60–7.50 (m, 5 H), 7.21 (d, *J* = 8.8 Hz, 2 H), 6.78 (d, *J* = 8.8 Hz, 2 H), 6.70 (s, 1 H), 5.23 (s, 2 H), and 3.78 (s, 3 H); ^13^C NMR (100.6 MHz) (CDCl_3_): δ 159.9 (C), 146.7 (C), 138.2 (C), 137.5 (C), 134.2 (C), 133.2 (CH), 130.8 (C), 129.6 (CH), 129.3 (CH), 129.0 (CH), 127.1 (C), 125.8 (CH), 124.9 (C), 119.6 (CH), 113.8 (CH), 103.8 (CH), 86.1 (C), 80.9 (C), 55.2 (CH_3_), and 38.2 (CH_2_).

d. Typical Procedure for the Synthesis of Substutited 1-(3-arylprop-2-yn-1-yl)-2-aryl-1*H*-indol-7-amine **1**.

STEP 4: Synthesis of 5-chloro-1-(3-(4-methoxyphenyl)prop-2-yn-1-yl)-2-phenyl-1*H*-indol-7-amine **1c**.

In a 50 mL Carousel Tube Reactor (Radely Discovery Technology), equipped with a magnetic stirring bar, 5-chloro-1-(3-(4-methoxyphenyl)prop-2-yn-1-yl)-7-nitro-2-phenyl-1*H*-indole (0.180 g, 0.431 mmol, 1.0 equiv.) was added to a solution of EtOH/H_2_O (3:1) and stirred at 120 °C for 10 min. Then, 51 μL of acetic acid and 72 mg of Fe (0) (0.431 mmol, 1.0 equiv.) were added in three portions every 15 min. The reaction mixture was then stirred for 2 h before being cooled at room temperature and concentrated under reduced pressure. Subsequently, the mixture was diluted with Et_2_O and washed with a saturated solution of NaHCO_3_ and with brine. The organic layer was dried over Na_2_SO_4_, filtered, and concentrated under reduced pressure. The residue was purified by filtration on a pad of Celite eluting with DCM to obtain 5-chloro-1-(3-(4-methoxyphenyl)prop-2-yn-1-yl)-2-phenyl-1*H*-indol-7-amine **1a** (0.140 g, 85% yield).

The 5-chloro-1-(3-(4-methoxyphenyl)prop-2-yn-1-yl)-2-phenyl-1*H*-indol-7-amine **1c**: 85% yield; orange solid; mp 91–92 °C; ^1^H NMR (400.13 MHz) (CDCl_3_): δ 7.71–7.68 (m, 2 H), 7.55–7.49 (m, 2 H), 7.48–7.44 (m, 1 H), 7.42 (d, *J* = 8.8 Hz, 2 H), 7.09 (d, *J* = 1.8 Hz, 1 H), 6.89 (d, *J* = 8.8 Hz, 2H), 6.54 (d, *J* = 1.8 Hz, 1 H), 6.47 (s, 1 H), 5.17 (s, 2 H), 4.35 (bs, 2 H), and 3.84 (s, 3 H); ^13^C NMR (100.6 MHz) (CDCl_3_): δ 160.1 (C), 143.2 (C), 134.0 (C), 133.2 (CH), 131.9 (C), 130.8 (C), 129.3 (CH), 128.7 (CH), 128.3 (CH), 127.1 (C), 126.3 (C), 114.1 (CH), 113.8 (C), 111.3 (CH), 110.1 (CH), 102.4 (CH), 86.6 (C), 84.9 (C), 55.3 (CH_3_), and 36.5 (CH_2_).

#### 3.2.2. Synthetic Procedures for Final Products

##### Typical Procedure for the Preparation of Substituted 5-Aryl-3*H*-pyrrolo [1,2,3-*de*] Quinoxalines 2: Synthesis of 8-Chloro-2-(4-methoxybenzyl)-5-phenyl-3*H*-pyrrolo [1,2,3-*de*]quinoxaline 2c

A 50 mL Carousel Tube Reactor (Radely Discovery Technology), equipped with a magnetic stirring bar, was charged with 5-chloro-1-(3-(4-methoxyphenyl)prop-2-yn-1-yl)-2-phenyl-1*H*-indol-7-amine **1c** (0.140 g, 0.361, 1.0 equiv.) and 2 mL of CH_2_Cl_2_ before adding 5.2 mg of (acetonitrile)-[(2-diphenyl)-di-*tert*-butylphosphine]Au(I) hexafluoroantimonate (0.0072 mmol, 0.02 equiv.). The solution was stirred for 1.5 h at room temperature, monitoring the disappearance of the starting material by TLC. Then, the mixture was concentered under reduced pressure and filtered on a pad of Celite to obtain 8-chloro-2-(4-methoxybenzyl)-5-phenyl-3*H*-pyrrolo [1,2,3-*de*]quinoxaline 2c (0.112 g, 80% yield).

The 8-chloro-2-(4-methoxybenzyl)-5-phenyl-3*H*-pyrrolo [1,2,3-*de*]quinoxaline **2c**: 80% yield; brown oil; ^1^H NMR (400.13 MHz) (CDCl_3_): δ 7.87 (d, *J* = 8.9 Hz, 2 H), 7.41–7.30 (m, 6 H), 7.29 (d, *J* = 1.9 Hz, 1 H), 6.87 (d, *J* = 8.8 Hz, 2 H), 6.46 (s, 1 H), 4.32–4.27 (m, 2 H), 3.76 (s, 3 H), and 3.13–3.08 (m, 2 H); ^13^C NMR (100.6 MHz) (CDCl_3_): δ 167.1 (C), 161.7 (C), 161.4 (C), 142.8 (C), 134.3 (C), 132.1 (CH), 131.9 (C), 130.8 (C), 129.3 (CH), 129.0 (CH), 128.6 (CH), 128.4 (C), 128.3 (CH), 125.7 (C), 123.7 (CH), 117.9 (CH), 113.9 (CH), 101.9 (CH), 55.4 (CH_3_), 48.5 (CH_2_), and 32.6 (CH_2_). HRMS *m*/*z* [M + H]^+^ calcd for C_24_H_20_ClN_2_O: 387.1259; found: 387.1274.

### 3.3. Characterization Data of Synthesized Compounds

Characterization data of starting materials and of **9d** are reported in Supplementary Materials.

#### Characterization Data of Final Compounds **2a**–**i** and **2k**

The 2-benzyl-8-chloro-5-phenyl-3*H*-pyrrolo [1,2,3-*de*]quinoxaline **2a**: 98% yield; brown oil; ^1^H NMR (400.13 MHz) (CDCl_3_): δ 8.06–7.98 (m, 2 H), 7.58–7.41 (m, 10 H), 6.60 (s, 1 H), 4.50–4.43 (m, 2 H), and 3.34–3.25 (m, 2 H); ^13^C NMR (100.6 MHz) (CDCl_3_): δ 167.7 (C), 142.8 (C), 139.7 (C), 134.0 (C), 131.8 (C), 131.1(C), 130.8 (C), 130.4 (CH), 129.3 (CH), 128.67 (CH), 128.64 (CH), 128.3 (CH), 127.2 (CH), 125.7 (C), 124.1 (CH), 118.4 (CH), 102.0 (CH), 48.4 (CH_2_), and 33.08 (CH_2_). HRMS *m*/*z* [M + H]^+^ calcd for C_23_H_18_ClN_2_: 357.1153; found: 357.1133.

The 8-chloro-2-(4-chlorobenzyl)-5-phenyl-3*H*-pyrrolo [1,2,3-*de*]quinoxaline **2b**: 86% yield; brown oil; ^1^H NMR (400.13 MHz) (CDCl_3_): δ 7.96 (d, *J* = 8.7 Hz, 2 H), 7.56–7.42 (m, 10 H), 6.59 (s, 1 H), 4.46–4.48 (m, 2 H), and 3.26–3.20 (m, 2 H); ^13^C NMR (100.6 MHz) (CDCl_3_): δ 166.2 (C), 142.8 (C), 138.0 (C), 136.7 (C), 133.7 (C), 131.7 (C), 130.9 (C), 129.3 (CH), 128.8 (CH), 128.7 (CH), 128.6 (CH), 128.4 (CH), 128.3 (CH), 125.7 (C), 124.2 (CH), 118.6 (CH), 102.1 (CH), 48.3 (CH_2_), and 32.8 (CH_2_). HRMS *m*/*z* [M + H]^+^ calcd for C_15_H_22_N_3_: 391.0763; found: 391.0754.

The 8-chloro-2-(4-methoxybenzyl)-5-phenyl-3*H*-pyrrolo [1,2,3-*de*]quinoxaline **2c**: 80% yield; brown oil; ^1^H NMR (400.13 MHz) (CDCl_3_): δ 8.00 (d, *J* = 8.9 Hz, 2 H), 7.59–7.39 (m, 7 H), 7.00 (d, *J* = 8.9 Hz, 2 H), 7.59 (s, 1 H), 4.46–4.39 (m, 2 H), 3.89 (s, 3 H), and 3.26–3.19 (m, 2 H); ^13^C NMR (100.6 MHz) (CDCl_3_): δ 167.1 (C), 161.7 (C), 142.8 (C), 134.3 (C), 132.1 (C), 131.9 (C), 130.8 (C), 129.3 (CH), 129.0 (CH), 128.6 (CH), 128.4 (C), 128.3 (CH), 125.7 (C), 123.6 (CH), 117.9 (CH), 113.9 (CH), 101.9 (CH), 55.4 (CH_3_), 48.5 (CH_2_), and 32.6 (CH_2_). HRMS *m*/*z* [M + H]^+^ calcd for C_24_H_20_ClN_2_O: 387.1259; found: 387.1274.

The 1-(4-((8-chloro-5-phenyl-3*H*-pyrrolo [1,2,3-*de*]quinoxalin-2-yl)methyl)phenyl)ethan-1-one **2d**: 85% yield; brown solid; mp 77–78 °C; ^1^H NMR (400.13 MHz) (CDCl_3_): δ 8.12–8.0 (m, 4 H), 7.57–7.41 (m, 7 H), 6.60 (s, 1 H), 4.50–4.42 (m, 2 H), 3.33–3.25 (m, 2 H), and 2.67 (s, 3 H); ^13^C NMR (100.6 MHz) (CDCl_3_): δ 197.6 (C), 166.3 (C), 143.6 (C), 142.9 (C), 138.1 (C), 133.6 (C), 131.7 (C), 130.9 (C), 129.3 (CH), 128.7 (CH), 128.6 (CH), 128.5 (CH), 128.4 (C), 128.3 (C), 127.4 (CH), 125.8 (C), 124.5 (CH), 119.0 (CH), 102.1 (CH), 48.2 (CH_2_), and 33.0 (CH_2_). HRMS *m*/*z* [M + H]^+^ calcd for C_25_H_20_ClN_2_O: 399.1259; found: 399.1263.

The 8-chloro-5-(4-methoxyphenyl)-2-(3-(trifluoromethyl)benzyl)-3*H*-pyrrolo [1,2,3-*de*]quinoxaline **2e**: 80% yield; brown solid; mp 170–171 °C; ^1^H NMR (400.13 MHz) (CDCl_3_): δ 8.27 (s, 1 H), 8.21 (d, *J* = 7.9 Hz, 1 H), 7.76 (d, *J* = 7.9 Hz, 1 H), 7.62 (t, *J* = 7.7 Hz, 1 H), 7.53–7.49 (m, 1 H), 7.48–7.40 (m, 3 H), 7.00 (d, *J* = 8.6 Hz, 2 H), 6.54 (s, 1 H), 4.48–4.40 (m, 2 H), 3.90 (s, 3 H), and 3.34–3.27 (m, 2 H); ^13^C NMR (100.6 MHz)(CDCl_3_): δ 165.6 (C), 159.9 (C), 142.8 (C), 140.4 (C), 133.4 (C), 131.1 (q, *J_CF_* = 32.0 Hz, C), 131.0 (C), 130.6 (CH), 130.3 (CH), 129.2 (CH), 128.1 (C), 126.8 (q, *J_CF_* = 3.6 Hz, CH), 125.7 (C), 124.0 (q, *J_CF_* = 273.0 Hz, C), 124.1 (CH), 124.05 (C), 124.00 (q, *J_CF_* = 3.6 Hz, CH), 118.8 (CH), 114.2 (CH), 101.4 (CH), 55.4 (CH_3_), 48.1 (CH_2_), and 32.9 (CH_2_); ^19^ F NMR (376.5 MHz)(CDCl_3_): δ −63.0. HRMS *m*/*z* [M + H]^+^ calcd for C_25_H_19_ClF_3_N_2_O: 455.1133; found: 455.1147.

The 8-chloro-2-(4-methoxybenzyl)-5-(4-methoxyphenyl)-3*H*-pyrrolo [1,2,3-*de*]quinoxaline **2f**: 80% yield; brown solid; mp 150–152 °C; ^1^H NMR (400.13 MHz) (CDCl_3_): δ 7.99 (d, *J* = 8.8 Hz, 2 H), 7.46–7.37 (m, 4 H), 7.05–7.35 (m, 4 H), 6.51 (s, 1 H), 4.44–4.35 (m, 2 H), 3.89 (s, 6 H), and 3.28–3.16 (m, 2 H); ^13^C NMR (100.6 MHz) (CDCl_3_): δ 167.0 (C), 161.7 (C), 159.7 (C), 142.7 (C), 134.2 (C), 132.1 (C), 130.8 (C), 130.6 (CH), 129.0 (CH), 128.2.0 (C), 125.6 (C), 124.0 (C), 123.3 (CH), 117.6 (CH), 114.1 (CH), 113.9 (CH), 101.2 (CH), 55.45 (CH_3_), 55.41 (CH_3_), 48.4 (CH_2_), and 32.6 (CH_2_). HRMS *m*/*z* [M + H]^+^ calcd for C_25_H_22_ClN_2_O_2_: 417.1364; found: 417.1351.

The 1-(4-((8-methyl-5-phenyl-3*H*-pyrrolo [1,2,3-*de*]quinoxalin-2-yl)methyl)phenyl)ethan-1-one **2g**: 90% yield; brown solid; mp 141–142 °C; ^1^H NMR (400.13 MHz) (CDCl_3_): δ 8.10 (d, *J* = 8.51 Hz, 2 H), 8.06 (d, *J* = 8.51 Hz, 2 H), 7.55–7.46 (m, 4 H), 7.45–7.40 (m, 1 H), 7.39 (s, 1 H), 7.35 (s, 1 H), 6.60 (s, 1 H), 4.51–4.46 (m, 2 H), 3.32–3.27 (m, 2 H), 2.67 (s, 3 H), and 2.54 (s, 3 H); ^13^C NMR (100.6 MHz) (CDCl_3_): δ 197.7 (C), 164.9 (C), 144.3 (C), 141.7 (C), 137.7 (C), 132.7 (C), 132.3 (C), 130.4 (C), 130.0 (C), 129.3 (CH), 128.6 (CH), 128.5 (CH), 128.1 (C), 128.0 (CH), 127.3 (CH), 126.4 (CH), 120.0 (CH), 102.0 (CH), 48.4 (CH_2_), 33.1 (CH_2_), 26.8, (CH_3_), and 21.1 (CH_3_); HRMS *m*/*z* [M + H]^+^ calcd for C_26_H_23_N_2_O: 379.1805; found: 379.1796.

The methyl 4-(2-(4-chlorobenzyl)-8-methyl-3*H*-pyrrolo [1,2,3-*de*]quinoxalin-5-yl)benzoate **2h**: 80% yield; yellow solid; mp 215–216 °C; ^1^H NMR (400.13 MHz) (CDCl_3_): δ 8.14 (d, *J* = 8.0 Hz, 2 H), 7.97 (d, *J_1_* = 8.3 Hz, 2 H), 7.60 (d, *J* = 8.0 Hz, 2 H), 7.46 (d, *J* = 8.3 Hz, 2 H), 7.37 (s, 1 H), 7.33 (s, 1 H), 6.68 (s, 1 H), 4.54–4.44 (m, 2 H), 3.98 (s, 3 H), 3.30–3.21 (m, 2 H), and 2.42 (s, 3 H); ^13^C NMR (100.6 MHz) (CDCl_3_): δ 166.7 (C), 165.0 (C), 140.6 (C), 138.3 (C), 136.7 (C), 136.4 (C), 133.0 (C), 132.9 (C), 133.2 (C), 129.8 (CH), 129.3 (C), 128.9 (CH), 128.8 (CH), 128.5 (CH), 126.6 (CH), 119.8 (CH), 103.4 (CH), 52.3 (CH_3_), 48.9 (CH_2_), 32.7 (CH_2_), and 21.1 (CH_3_). HRMS *m*/*z* [M + H]^+^ calcd for C_26_H_22_ClN_2_O_2_: 429.1364; found: 429.1375.

The 2-(4-chlorobenzyl)-8-methyl-5-phenyl-3*H*-pyrrolo [1,2,3-*de*]quinoxaline **2i**: 79% yield; brown solid; mp 128–129 °C; ^1^H NMR (400.13 MHz) (CDCl_3_): δ 7.96 (d, *J* = 8.6 Hz, 2 H), 7.59–7.39 (m, 7 H), 7.37 (s, 1 H), 7.32 (s, 1 H), 6.60 (s, 1 H), 4.48–4.42 (m, 2 H), 3.27–3.20 (m, 2 H), and 2.53 (s, 3 H); ^13^C NMR (100.6 MHz) (CDCl_3_): δ 164.9 (C), 141.7 (C), 138.6 (C), 136.2 (C), 132.8 (C), 132.4 (C), 130.3 (C), 129.9 (C), 129.3 (CH), 128.9 (C), 128.7 (CH), 128.6 (CH), 128.5 (CH), 128.2 (C), 128.0 (CH), 126.1 (CH), 119.6 (CH), 102.0 (CH), 48.5 (CH_2_), 32.8 (CH_2_), and 21.1 (CH_3_). HRMS *m*/*z* [M + H]^+^ calcd for C_24_H_20_ClN_2_: 371.1310; found: 371.1321.

The 1-(4-((6-(4-methoxyphenyl)-8-methyl-5-phenyl-3*H*-pyrrolo [1,2,3-*de*]quinoxalin-2-yl)methyl)phenyl)ethan-1-one **2k**: 84% yield; orange solid; mp 133–134 °C; ^1^H NMR (400.13 MHz) (CDCl_3_): δ 8.10 (d, *J* = 8.4 Hz, 2 H), 8.06 (d, *J* = 8.4 Hz, 2 H), 7.46 (s, 1 H), 7.42–7.31 (m, 6 H), 7.24 (d, *J* = 8.4 Hz, 2 H), 6.88 (d, *J* = 8.4 Hz, 2 H), 7.37 (s, 1 H), 7.32 (s, 1 H), 6.60 (s, 1 H), 4.41–4.34 (m, 2 H), 3.83 (m, 3 H), 3.38–3.31 (m, 2 H), and 2.68 (s, 3 H); ^13^C NMR (100.6 MHz) (CDCl_3_): δ 197.7 (C), 164.8 (C), 157.8 (C), 144.4 (C), 137.7 (C), 137.3 (C), 132.6 (C), 131.5 (C), 131.1 (CH), 130.2 (C), 129.8 (C), 128.6 (CH), 128.4 (CH), 128.0 (CH), 127.29 (C), 127.27 (CH), 127.1 (C), 127.0 (CH), 119.2 (CH), 115.2 (C), 113.8 (CH) (overlapping), 55.2 (CH_3_), 47.9 (CH_2_), 32.2 (CH_2_), 26.8 (CH_3_), and 21.1 (CH_3_). HRMS *m*/*z* [M + H]^+^ calcd for C_33_H_29_N_2_O_2_: 485.2224; found: 485.2239.

## 4. Conclusions

A protocol for the synthesis of functionalized 3H-pyrrolo-[1,2,3-*de*] quinoxalines from substituted N-alkynyl indoles has been developed. The reaction proved to be highly selective in promoting the exclusive formation of the 6-exo-dig cyclization product, which, after isomerization, affords the final compound. As to the isomerization mode, theoretical calculations were provided to support the experimental data indicating that differences in terms of stability between the two possible isomers determine the formation of the imine-type product. The mild reaction conditions in which the reaction takes place led to the synthesis of derivatives with useful functional groups, including halogen, alkoxyl, cyano, ketone, and ester, with yields from good to high in all the cases reported.

## Figures and Tables

**Table 1:**
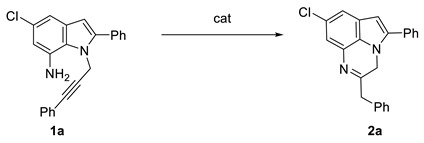
Synthesis of 2-benzyl-7-chloro-5-phenyl-3*H*-pyrrolo [1,2,3-*de*]quinoxaline **2a** from 4-chloro-2-phenyl-1-(3-phenylprop-2-yn-1-yl)-1*H*-indol-7-amine **1a**.

Entry ^1^	Catalysts (mmol%)	Solvent (mL)	Temperature (°C)	Time (h)	Yield 2a ^2^ (%)
1	JP(MeCN)AuSb_6_^3^ (2)	CH_2_Cl_2_	60	1	98
2	JP(MeCN)AuSb_6_^3^ (2)	CH_2_Cl_2_	rt	7	98
3	PPh_3_AuCl/AgSbF_6_ (2/2)	CH_2_Cl_2_	rt	24	42 ^4^
4	PtCl_2_ (4)	EtOH	80	24	83 ^5^
5	PdCl_2_(CH_3_CN)_2_ (5)	CH_3_CN	80	2	36
6	TsOH	EtOH	80	2	- ^6^

^1^ Reactions were carried out with 0.25 mmol of **1** in 1.0 mL of solvent and in the presence of a catalyst in the reported mmol percentage. ^2^ Yields are given for isolated products. ^3^ JP = JohnPhos. ^4^ A total of 55% of starting material **1** recovered. ^5^ A total of 14% of starting material **1** recovered. ^6^ In this condition, a mixture of degradation compounds was obtained.

**Table 2:**
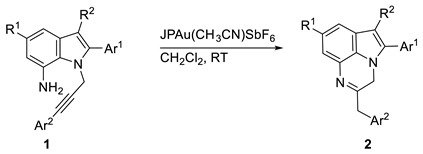
Synthesis of functionalized 3*H*-pyrrolo-[1,2,3-*de*] quinoxalines **2** from substituted *N*-propargyl indoles **1** through a gold-catalyzed intramolecular hydroamination.

Entry ^1^	1	R ^1^	R ^2^	Ar ^1^	Ar ^2^	2	Time (h)	Yield 2 (%) ^2^
1	**1a**	Cl	H	Ph	Ph	**2a**	7	98
2	**1b**	Cl	H	Ph	4-Cl-C_6_H_4_	**2b**	1	86
3	**1c**	Cl	H	Ph	4-OMe-C_6_H_4_	**2c**	1.5	80
4	**1d**	Cl	H	Ph	4-COMe-C_6_H_4_	**2d**	1.5	85
5	**1e**	Cl	H	4-OMe	3-CF_3_-C_6_H_4_	**2e**	4	80
6	**1f**	Cl	H	4-OMe	4-OMe-C_6_H_4_	**2f**	18	80
7	**1g**	Me	H	Ph	4-COMe-C_6_H_4_	**2g**	24	90
8	**1h**	Me	H	4-CO_2_Me	4-Cl-C_6_H_4_	**2h**	3	80
9	**1i**	Me	H	Ph	4-Cl-C_6_H_4_	**2i**	5	79
10	**1j**	Me	H	Ph	-	**2j**	1	- ^3^
11	**1k** ^4^	Me	4-OMe-C_6_H_4_	4-COMe-C_6_H_4_	Ph	**2k**		84

^1^ Reactions were carried out on 0.25 mmol of **1** in 1.0 mL of CH_2_Cl_2_, in the presence of 0.02 equiv of JPAu(CH_3_CN)SbF_6_. ^2^ Yields are given for isolated products. ^3^ Degradation compounds. ^4^ Starting material **1k** has been prepared according to the procedure detailed in Supplementary Materials.

## Data Availability

Data contained within the article or Supplementary Materials.

## Supplementary Materials

The following supporting information can be downloaded at: https://www.mdpi.com/article/10.3390/molecules28155831/s1.
