# Supplementary material for: Synthesis of Functionalized 3H-pyrrolo-[1,2,3-de] Quinoxalines via Gold-Catalyzed Intramolecular Hydroamination of Alkynes
Source: Molecules. 2023 Aug 2;28(15):5831. doi: 10.3390/molecules28155831 (PMC10421283; doi:10.3390/molecules28155831)

Supplementary Materials

Synthesis of Functionalized 3*H*-pyrrolo-[1,2,3-*de*] Quinoxalines via Gold-Catalyzed Intramolecular Hydroamination of Alkynes

Antonia Iazzetti [1,2,†], Giancarlo Fabrizi [3,*], Antonella Goggiamani [3], Federico Marrone [3], Alessio Sferrazza [3,4,*] and Karim Ullah [3]

[1] Dipartimento di Scienze Biotecnologiche di base, Cliniche Intensivologiche e Perioperatorie, Università Cattolica del Sacro Cuore, L.go Francesco Vito 1, 00168 Rome, Italy; antonia.iazzetti@unicatt.it
[2] Policlinico Universitario 'A. Gemelli' Foundation-IRCCS, 00168 Rome, Italy
[3] Dipartimento di Chimica e Tecnologie del Farmaco, Sapienza, Università di Roma, P. le A. Moro 5, 00185 Rome, Italy; antonella.goggiamani@uniroma1.it (A.G.); federico.marrone@uniroma1.it (F.M.); karim.ullah@uniroma1.it (K.U.)
[4] Medicinal Chemistry Department, IRBM S.p.A., 00071 Pomezia, Italy
* Correspondence: giancarlo.fabrizi@uniroma1.it (G.F.); a.sferrazza@irbm.com (A.S.)
† Alessio Sferrazza is currently a research scientist in Medicinal Chemistry Department, IRBM S.p.A., 00071 Pomezia, Italy

Contents

1. SYNTHETIC PROCEDURES FOR STARTING MATERIALS 3

1.1 General procedure for the preparation of substituted 1-(3-arylprop-2-yn-1-yl)-2-aryl-1*H*-indol-7-amine **1** 3

1.1.a. Typical procedure for the preparation of 5- substituted-7-nitro-2-phenyl-1H-indole **5** 3

1.1.b. Typical procedure for the preparation of substituted 7-nitro-2-phenyl-1-(prop-2-yn-1-yl)-1H-indoles **6** 4

1.1.c. Typical procedure for the preparation of substituted 1-(3-arylprop-2-yn-1-yl)-7-nitro-2-phenyl-1H-indoles **7** 4

1.1.d. Typical procedure for the synthesis of substutited 1-(3-arylprop-2-yn-1-yl)-2-aryl-1H-indol-7-amine **1** 5

1.2. General procedure for the preparation of 1-(4-(3-(7-amino-5-methyl-2,3-diphenyl-1*H*-indol-1-yl)prop-1-yn-1-yl)phenyl)ethan-1-one **1l** 5

2. PROCEDURE FOR THE SYNTHESIS OF COMPOUND 9d 7

3. CHARACTERIZATION DATA OF STARTING MATERIALS 9

3.1 Characterization data of 5-substituted 7-nitro-2-aryl-1*H*-indole **5** 9

3.2 Characterization data of 7-nitro-2-aryl-1-(prop-2-yn-1-yl)-1*H*-indole **6** 9

3.3 Characterization data of 7-nitro-2-aryl-1-(3-phenylprop-2-yn-1-yl)-1*H*-indole **7** 11

3.4 Characterization data of 2-aryl-1-(3-phenylprop-2-yn-1-yl)-1*H*-indol-7-amines **1** 15

4. REFERENCES **18**

5. HF CALCULATION ON ISOMERS 2a and 2'a **19**

6. COPIES OF NMR SPECTRA **198**

1. SYNTHETIC PROCEDURES FOR STARTING MATERIALS

1.1 General procedure for the preparation of substituted 1-(3-arylprop-2-yn-1-yl)-2-aryl-1*H*-indol-7-amine 1

Starting materials **1** were prepared according to literature procedures through the four-step sequence of reactions depicted in scheme S1.

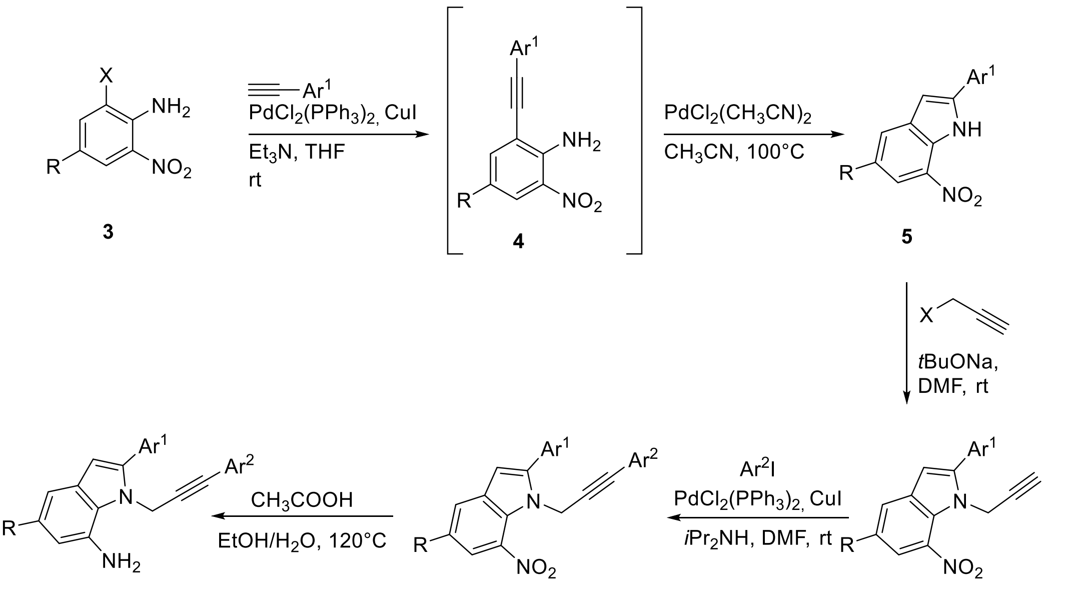

Scheme S1. Preparation of starting materials **1**

1.1.a. Typical procedure for the preparation of 5- substituted-7-nitro-2-phenyl-1H-indole ***5***

STEP 1: synthesis of 5-chloro-7-nitro-2-phenyl-1H-indole ***5a***

In a 100 ml two-necked round bottom flask, equipped with a magnetic stirring bar, $PdCl_2(PPh_3)_2$ (0.329 g, 0.469 mmol, 0.04 equiv.) and CuI (0.045 g, 0.234 mmol, 0.02 equiv.) were dissolved in 36.0 mL of THF and 1.56 mL of Et_3N at room temperature and under a nitrogen atmosphere. Then, 2-iodo-4-chloro-6-nitroaniline (3.5 g, 11.74 mmol, 1.0 equiv.) was added and, dropwise, phenylacetylene (1.93 mL, 17.61 mmol, 1.5 equiv.). The solution was stirred for 2h. After this time, the reaction mixture was diluted with Et_2O, and washed with a saturated solution of NH_4Cl, $NaHCO_3$, and brine. The organic layer was separated, dried over Na_2SO_4, filtered, and concentrated under reduced pressure. The residue, containing 4-chloro-2-nitro-6-(phenylethynyl)aniline **4a**, was transferred with 60 mL of MeCN in a two-necked 100-mL round bottom flask equipped with a condenser, and a magnetic stirring bar, then $PdCl_2(CH_3CN)_2$ was added. The solution was stirred for 2.5 h at 100 °C. After this time, the mixture was cooled to room temperature, concentrated under reduced pressure, purified by chromatography on SiO_2 (25-40 µm), eluting with an 92/8 (v/v) *n*-hexane-AcOEt mixture (R_f = 0.26) to obtain 5-chloro-7-nitro-2-phenyl-1*H*-indole **5a** (2,57 g, 80 % yield).

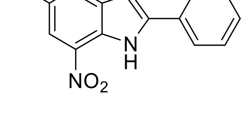

5-chloro-7-nitro-2-phenyl-1*H*-indole 5a: yield: 80%; orange solid; mp: 164 - 166°C; ^{1}H NMR (400.13 MHz) ($CDCl_3$): δ 10.07 (bs, 1 H), 8.11 (d, *J* = 1.5 Hz, 1 H), 7.93 - 7.88 (m, 1 H), 7.74 (d, *J* = 7.8 Hz, 1 H), 7.53 (t, *J*

= 7.3 Hz, 2 H), 7.45 (t, *J* = 7.3 Hz, 2 H), 6.90 (d, *J* = 2.4 Hz, 1 H), 5.05 (d, *J* = 2.4 Hz, 2 H), 2.20 (t, *J* = 2.4 Hz, 1 H); ^{13}C NMR (100.6 MHz) ($CDCl_3$): δ 142.0 (C), 133.7 (C), 133.5 (C), 130.4 (C), 129.34 (CH), 129.27 (CH), 128.8 (C), 127.6 (CH), 125.7 (CH), 125.1 (C), 118.7 (CH), 100.1 (CH).

1.1.b. Typical procedure for the preparation of substituted 7-nitro-2-phenyl-1-(prop-2-yn-1-yl)-1H-indoles ***6***
STEP 2: synthesis of 5-chloro-7-nitro-2-phenyl-1-(prop-2-yn-1-yl)-1H-indole 6a

A 250 mL round bottom flask, equipped with a magnetic stirring bar, was charged with ^{t}BuONa (1.35 g, 14.02 mmol, 1.5 equiv) and 90 mL of anhydrous DMF. The reaction mixture was cooled at 0°C and 5-chloro-7-nitro-2-phenyl-1*H*-indole (2.4 g, 9.35 mmol, 1.0 equiv) was added dropwise. Then, propargyl bromide (1.21 mL, 14.02 mmol, 1.5 equiv) was added and the solution was warmed to room temperature and stirred for 6 h. After this time, the reaction mixture was diluted with Et_2O and washed with saturated solution of $NaHCO_3$ and brine. The organic layer was dried over Na_2SO_4, filtered, and concentrated under reduced pressure. The residue was purified by chromatography on SiO_2 (25-40 μm), eluting with an 96/4 (v/v) *n*-hexane-AcOEt mixture (R_f = 0.25) to obtain 5-chloro-7-nitro-2-phenyl-1-(prop-2-yn-1-yl)-1*H*-indole **6a** (2.324 g, 80 % yield).

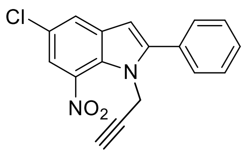

5-chloro-7-nitro-2-phenyl-1-(prop-2-yn-1-yl)-1*H*-indole 6a: 80 % yield; brown solid; mp 103 - 104 °C; ^{1}H NMR (400.13 MHz) ($CDCl_3$): δ 7.92 (d, *J* = 1.9 Hz, 1 H), 7.87 (d, *J* = 1.9 Hz, 1 H), 7.58-7.50 (m, 5 H), 6.70 (s, 1 H), 5.05 (d, *J* = 2.4 Hz, 2 H), 2.20 (t, *J* = 2.4 Hz, 1 H); ^{13}C NMR (100.6 MHz) ($CDCl_3$): δ 146.8 (C), 137.3 (C), 134.3 (C), 130.6 (C), 129.6 (CH), 129.4 (CH), 129.1 (CH), 127.1 (C), 126.0 (CH), 125.2 (C), 119.8 (CH), 104.1 (CH), 77.1 (C), 74.4 (CH), 37.1 (CH_2).

1.1.c. Typical procedure for the preparation of substituted 1-(3-arylprop-2-yn-1-yl)-7-nitro-2-phenyl-1H-indoles ***7***
STEP 3: synthesis of 5-chloro-1-(3-(4-methoxyphenyl)prop-2-yn-1-yl)-7-nitro-2-phenyl-1H-indole ***7c***

In a two-necked 50-mL round bottom flask, equipped with a magnetic stirring bar, $PdCl_2(PPh_3)_2$ (0.084 g, 0.119 mmol, 0.04 equiv.) and CuI (0.011 g, 0.0597 mmol, 0.02 equiv.) were dissolved in 12.3 mL of *i*Pr_2NH and 6.1 mL of DMF at room temperature and under nitrogen; then, 4-iodoanisole (0.839 g, 3.584 mmol, 1.2 equiv.) and 5-chloro-7-nitro-2-phenyl-1-(prop-2-yn-1-yl)-1*H*-indole (0.928 g, 2.98 mmol, 1.0 equiv.) were added and the resulting mixture was stirred for 24 h. After this time, the mixture was diluted with Et_2O and washed with a saturated solution of NH_4Cl, a saturated solution of $NaHCO_3$, and with brine. The organic layer was dried over Na_2SO_4, filtered, and concentrated under reduced pressure. The residue was purified by chromatography on SiO_2 (25-40 μm), eluting with a 93/7 (v/v) *n*-hexane/AcOEt mixture (R_f = 0.27) to obtain 5-chloro-1-(3-(4-methoxyphenyl)prop-2-yn-1-yl)-7-nitro-2-phenyl-1*H*-indole **7c** (0.860 g, 70% yield).

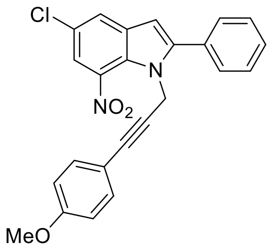

5-chloro-1-(3-(4-methoxyphenyl)prop-2-yn-1-yl)-7-nitro-2-phenyl-1*H*-indole 7c: 70 % yield; yellow solid; mp 133 - 134 °C; ^{1}H NMR (400.13 MHz) ($CDCl_3$): δ 7.91 (d, *J* = 1.9 Hz, 1 H), 7.87 (d, *J* = 1.9 Hz, 1 H), 7.60 - 7.50 (m, 5 H), 7.21 (d, *J* = 8.8 Hz, 2 H), 6.78 (d, *J* = 8.8 Hz, 2 H), 6.70 (s, 1 H), 5.23 (s, 2 H), 3.78 (s, 3 H); ^{13}C NMR (100.6 MHz) ($CDCl_3$): δ 159.9 (C), 146.7 (C), 138.2 (C), 137.5 (C), 134.2 (C), 133.2 (CH), 130.8 (C), 129.6 (CH), 129.3 (CH), 129.0 (CH), 127.1 (C), 125.8 (CH), 124.9 (C), 119.6 (CH), 113.8 (CH), 103.8 (CH), 86.1 (C), 80.9 (C), 55.2 (CH_3), 38.2 (CH_2).

*1.1.d. Typical procedure for the synthesis of substutited 1-(3-arylprop-2-yn-1-yl)-2-aryl-1H-indol-7-amine **1***
*STEP 4: synthesis of 5-chloro-1-(3-(4-methoxyphenyl)prop-2-yn-1-yl)-2-phenyl-1H-indol-7-amine **1c***

In a 50 mL Carousel Tube Reactor (Radely Discovery Technology), equipped with a magnetic stirring bar, 5-chloro-1-(3-(4-methoxyphenyl)prop-2-yn-1-yl)-7-nitro-2-phenyl-1*H*-indole (0.180 g, 0.431 mmol, 1.0 equiv.) was added to a solution of EtOH/H_2O (3:1) and stirred at 120°C for 10 minutes. Then, 51 μl of acetic acid and 72 mg of Fe (0) (0.431 mmol, 1.0 equiv.) were added in three portions every 15 minutes. The reaction mixture was then stirred for 2 hours before being cooled at room temperature, and concentrated under reduced pressure. Subsequently, the mixture was diluted with Et_2O and washed with a saturated solution of $NaHCO_3$, and with brine. The organic layer was dried over Na_2SO_4, filtered, and concentrated under reduced pressure. The residue was purified by filtration on a pad of celite eluting with DCM to obtain 5-chloro-1-(3-(4-methoxyphenyl)prop-2-yn-1-yl)-2-phenyl-1*H*-indol-7-amine **1a** (0.140 g, 85% yield)

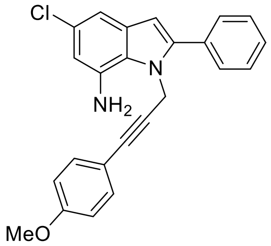

5-chloro-1-(3-(4-methoxyphenyl)prop-2-yn-1-yl)-2-phenyl-1*H*-indol-7-amine 1c: 85% yield; orange solid; mp 91 - 92 °C; ^{1}H NMR (400.13 MHz) ($CDCl_3$): δ 7.71 - 7.68 (m, 2 H), 7.55 - 7.49 (m, 2 H), 7.48 - 7.44 (m, 1 H), 7.42 (d, , *J* = 8.8 Hz, 2 H), 7.09 (d, *J* = 1.8 Hz, 1 H), 6.89 (d, *J* = 8.8 Hz, 2H), 6.54 (d, *J* = 1.8 Hz, 1 H), 6.47 (s, 1 H), 5.17 (s, 2 H), 4.35 (bs, 2 H), 3.84 (s, 3 H); ^{13}C NMR (100.6 MHz) ($CDCl_3$): δ 160.1 (C), 143.2 (C), 134.0 (C), 133.2 (CH), 131.9 (C), 130.8 (C), 129.3 (CH), 128.7 (CH), 128.3 (CH), 127.1 (C), 126.3 (C), 114.1 (CH), 113.8 (C), 111.3 (CH), 110.1 (CH), 102.4 (CH), 86.6 (C), 84.9 (C), 55.3 (CH_3), 36.5 (CH_2).

1.2. General procedure for the preparation of 1-(4-(3-(7-amino-5-methyl-2,3-diphenyl-1*H*-indol-1-yl)prop-1-yn-1-yl)phenyl)ethan-1-one 1l

Starting material 1l was prepared according to literature procedures from 5-methyl-7-nitro-2-phenyl-1*H*-indole through the sequence of reactions depicted in scheme S2.

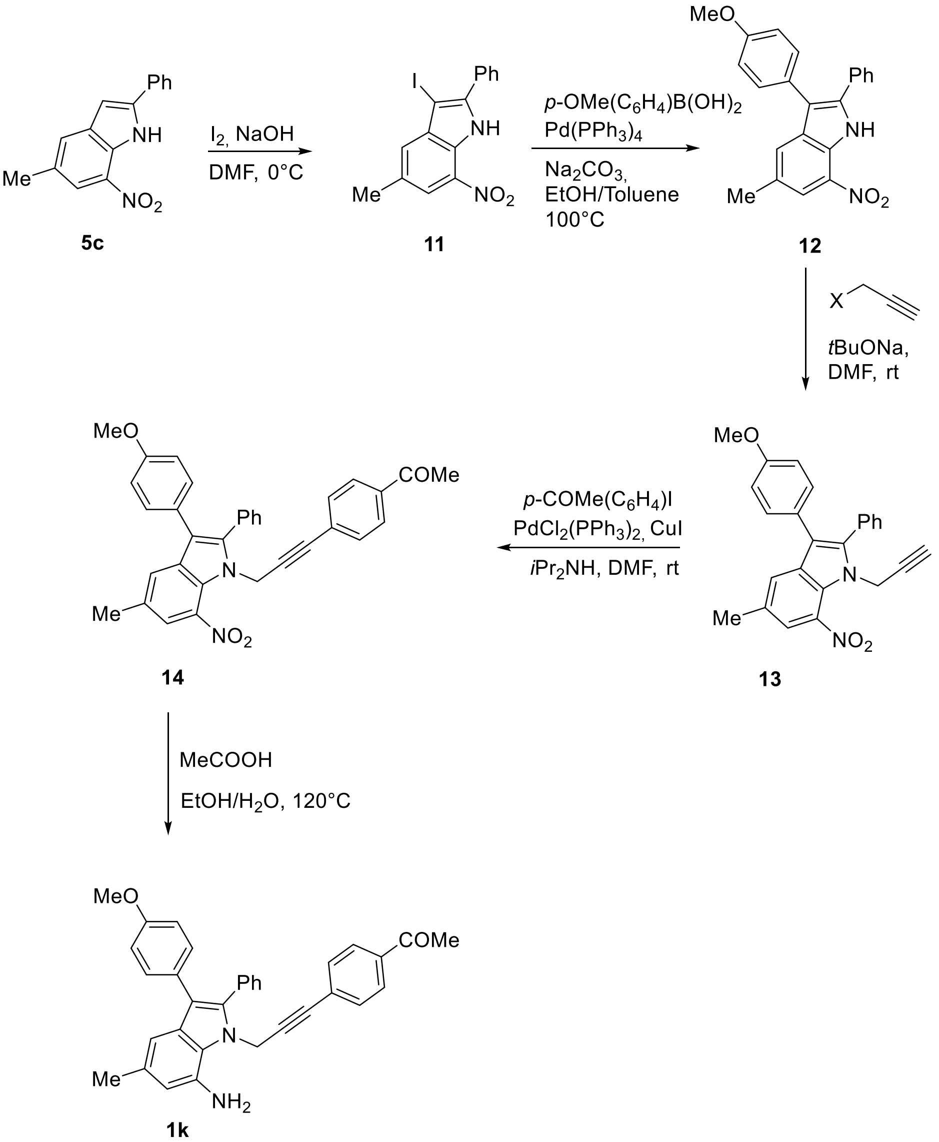

Scheme S2. Preparation of starting materials **1k**

<u>*STEP 1*: synthesis of 3-iodo-5-methyl-7-nitro-2-phenyl-1*H*-indole</u>

To a solution of 5-methyl-7-nitro-2-phenyl-1*H*-indole **5c** (1.0 g, 3.982 mmol, 1.0 equiv.) in DMF (8.0 mL) KOH (0.671 g, 11.94 mmol, 3.0 equiv.) was added at 0°C and the resulting mixture was stirred for 10 minutes before a solution of iodine (1.061 g, 4.181 mmol, 1.05 equiv.) in DMF (10.0 mL) was added dropwise over 5 minutes. After 1 h, the mixture was poured into a saturated solution of NH_4Cl and $Na_2S_2O_3$ to precipitate the product. The solid material was filtered off, solubilized in Et_2O, washed with water and dried over Na_2SO_4. After filtration, the mixture was concentrated under reduced pressure to give of 3-iodo-5-methyl-7-nitro-2-phenyl-1*H*-indole as an orange powder (0.97 g, 65% yield).

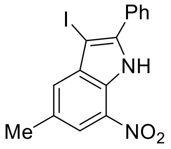

11

3-iodo-5-methyl-7-nitro-2-phenyl-1*H*-indole 11: 65% yield; orange solid; mp: 143-145 °C; ^{1}H NMR (400.13 MHz) ($CDCl_3$): δ 10.06 (bs, 1 H), 8.07 (s, 1 H), 7.88-7.82 (m, 2 H), 7.67 (s, 1 H), 7.61-7.47 (m, 3 H), 2.59 (s, 3 H); ^{13}C NMR (100.6 MHz) ($CDCl_3$): δ 140.6 (C), 135.3 (C), 132.4 (C), 131.0 (C), 130.3 (C), 129.9 (CH), 129.4 (CH), 128.9 (CH), 128.6 (C), 128.5 (CH), 121.0 (CH), 58.4 (C), 21.2 (CH_3).

STEP 2: synthesis of 5-methyl-7-nitro-2,3-diphenyl-1H-indole

In a three-necked round bottom flask, equipped with a condenser and magnetic stirring bar, [Pd(PPh_3)$_4$] (115.4 mg, 0.10 mmol, 0.05 equiv.) was dissolved at room temperature in 25 mL of EtOH/Toluene (2:1) under argon; then, 3-iodo-5-methyl-7-nitro-2-phenyl-1*H*-indole (0.756 g, 2.0 mmol, 1.0 equiv.), 4-methoxyphenylboronic acid (0.912 g, 6.0 mmol, 3.0 equiv.), and Na_2CO_3 (2.39 g, 22.6 mmol,3.0 equiv.) were added and the mixture was refluxed for 16 hours. After this time, the mixture was cooled to room temperature, diluted with CH_2Cl_2 and washed with brine. The organic layer was dried over Na_2SO_4, filtered, and concentrated under reduced pressure. The residue was purified by chromatography on SiO_2 (25-40 μm), eluting with an 80/20 (v/v) *n*-hexane-AcOEt mixture (R_f = 0.22) to obtain the desired product (0.609 g, 85% yield)

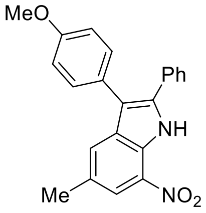

12

3-(4-methoxyphenyl)-5-methyl-7-nitro-2-phenyl-1*H*-indole 12: 85% yield; yellow solid; mp: 133-135 °C; ^{1}H NMR (400.13 MHz) ($CDCl_3$): δ 9.89 (bs, 1 H), 8.03 (s, 1 H), 7.75 (s, 1 H), 7.53-7.46 (m, 2 H), 7.43-7.31 (m, 5 H), 6.99 (d, J = 8.7 Hz, 1 H), 3.89 (s, 3 H), 2.52 (s, 3 H); ^{13}C NMR (100.6 MHz) ($CDCl_3$): δ 158.7 (C), 136.1 (C), 133.1 (C), 132.3 (C), 131.5 (C), 131.3 (CH), 129.6 (C), 128.9 (CH), 128.7 (C), 128.4 (CH), 128.1 (CH), 127.9 (CH), 125.9 (C), 120.3 (CH), 115.0 (C), 114.3 (CH), 55.3 (CH_3), 21.1 (CH_3).

STEPS 3 – 5 were carried out with procedures described in paragraphs *2.1b -d*

2. PROCEDURE FOR THE SYNTHESIS OF COMPOUND 9d

A flame dried 50 mL Carousel Tube Reactor (Radely Discovery Technology), equipped with a magnetic stirring bar, was charged with 8-chloro-2-(4-methoxybenzyl)-5-phenyl-3*H*-pyrrolo[1,2,3-*de*]quinoxaline **2c** (50.0 mg, 0.13 mmol, 1.0 equiv) dissolved in anhydrous 2 mL of THF under argon. Then, a solution of $LiAlH_4$ 2 M in THF (108 μl, 0.26 mmol, 2.0 equiv.) was added at 0°C and the mixture was stirred for 15 minutes at 80°C. After this time, the mixture was diluted with Et_2O and washed with a saturated $NaHCO_3$ solution and brine. The organic layer was dried over Na_2SO_4, filtered, concentrated under reduced pressure. The resulting residue was purified by chromatography on SiO_2 (25-40 μm), eluting with a 93/7 (v/v) *n*-hexane/AcOEt mixture (R_f = 0.27) to obtain 8-chloro-2-(4-methoxybenzyl)-5-phenyl-2,3-dihydro-1*H*-pyrrolo[1,2,3-*de*]quinoxaline (49.5 mg, 98 % yield).

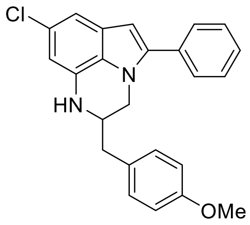

8-chloro-2-(4-methoxybenzyl)-5-phenyl-2,3-dihydro-1*H*-pyrrolo[1,2,3-*de*]quinoxaline 9d: 98 % yield; brown oil; ^{1}H NMR (400.13 MHz) ($CDCl_3$): 7.53-7.42 (m, 4 H), 7.44-7.35 (m, 3 H), 7.08 (d, J = 1.8 Hz, 1 H), 6.93 (d, J = 8.8 Hz, 2 H), 6.48 (s, 1 H), 6.47 (d, J = 1.8 Hz, 1 H), 4.76 (bs, 1 H), 4.45 - 4.33 (m, 2 H), 3.84 (s, 3 H), 3.74-3.64 (m, 1 H), 2.45-2.28 (m, 2 H); ^{13}C NMR (100.6 MHz) ($CDCl_3$): δ 159.2 (C), 143.7 (C), 136.6 (C), 135.9 (C), 132.7 (C), 131.7 (C), 129.3 (CH), 128.6 (CH), 128.1 (CH), 127.7 (CH), 127.4 (C), 126.2 (C), 114.2 (CH), 110.4 (CH), 108.5 (CH), 103.4 (CH), 62.4 (CH), 55.4 (CH_3), 46.6 (CH_2), 38.6 (CH_2).

3. CHARACTERIZATION DATA OF STARTING MATERIALS

3.1 Characterization data of 5-substituted 7-nitro-2-aryl-1*H*-indole 5

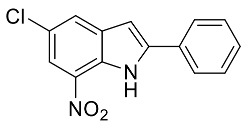

5-chloro-7-nitro-2-phenyl-1*H*-indole 5a 80 % yield; orange solid; mp: 164 - 166 °C; ^{1}H NMR (400.13 MHz) ($CDCl_3$): δ 10.07 (bs, 1 H), 8.11 (d, *J* = 1.5 Hz, 1 H), 7.93 - 7.88 (m, 1 H), 7.74 (d, *J* = 7.8 Hz, 1 H), 7.53 (t, *J* = 7.3 Hz, 2 H), 7.45 (t, *J* = 7.3 Hz, 2 H), 6.90 (d, *J* = 2.4 Hz, 1 H), 5.05 (d, *J* = 2.4 Hz, 2 H), 2.20 (t, *J* = 2.4 Hz, 1 H); ^{13}C NMR (100.6 MHz) ($CDCl_3$): δ 142.0 (C), 133.7 (C), 133.5 (C), 130.4 (C), 129.34 (CH), 129.27 (CH), 128.8 (C), 127.6 (CH), 125.7 (CH), 125.1 (C), 118.7 (CH), 100.1 (CH).

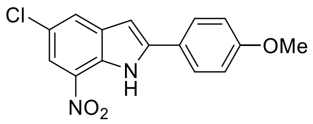

5-chloro-2-(4-methoxyphenyl)-7-nitro-1*H*-indole 5b: 72 % yield; yellow - orange solid; mp: 196 - 198 °C; ^{1}H NMR (400.13 MHz) (DMSO- d_6): δ 11.78 (bs, 1 H), 8.13 (d, *J* = 1.3 Hz, 1 H), 8.05 (d, *J* = 1.3 Hz, 1 H), 8.01 (d, *J* = 8.7 Hz, 2 H), 7.12 (d, *J* = 8.7 Hz, 2 H), 7.1 (s, 1 H), 3.89 (s, 3 H); ^{13}C NMR (100.6 MHz) (DMSO-d_6): δ 160.3 (C), 143.8 (C), 134.6 (C), 133.0 (C), 128.8 (CH), 128.7 (C), 127.0 (CH), 123.6 (C), 123.3 (C), 117.5 (CH), 114.7 (CH), 99.9 (CH), 55.8 (CH_3).

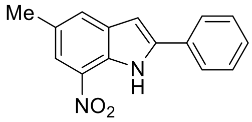

5-methyl-7-nitro-2-phenyl-1*H*-indole 5c: 97 % yield; yellow solid; mp 172 - 174 °C [1]; ^{1}H NMR (400.13 MHz) ($CDCl_3$): δ 9.87 (s, 1H), 7.88 (s, 1H), 7.73–7.57 (m, 3H), 7.56–7.24 (m, 3H), 6.78 (s, 1H), 2.47 (s, 3H); ^{13}C NMR (100.6 MHz, $CDCl_3$) δ 140.5 (C), 133.04 (C), 132.3 (C), 131.0 (C), 129.5 (C), 129.2 (CH), 128.8 (C), 128.8 (CH), 128.7(CH), 125.5 (CH), 119.9 (CH), 100.0 (CH), 21.1 (CH_3).

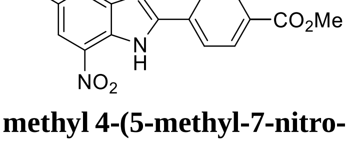

methyl 4-(5-methyl-7-nitro-1*H*-indol-2-yl)benzoate 5d 98 % yield; yellow solid; mp: 244 - 246 °C; ^{1}H NMR (400.13 MHz) ($CDCl_3$): δ 9.96 (s, 1H), 8.08 (d, *J* = 8.2 Hz, 2H), 7.93 (s, 1H), 7.72 (d, *J* = 8.7 Hz, 3H), 6.90 (d, *J* = 1.7 Hz, 1H), 3.89 (s, 3H), 2.46 (s, 3H).; ^{13}C NMR (100.6 MHz, $CDCl_3$) δ 166.5 (C), 139.1 (C), 135.1 (C), 132.8 (C), 132.5 (C), 130.5 (CH), 129.9 (C), 129.9 (C), 129.1 (CH), 125.2 (CH), 120.7 (CH), 101.7 (CH), 52.3 (CH_3), 21.1 (CH_3).

3.2 Characterization data of 7-nitro-2-aryl-1-(prop-2-yn-1-yl)-1*H*-indole 6

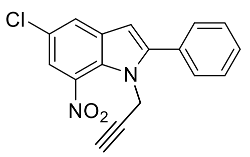

5-chloro-7-nitro-2-phenyl-1-(prop-2-yn-1-yl)-1*H*-indole 6a: 80 % yield; brown solid; mp: 114 - 116 °C; ^{1}H NMR (400.13 MHz) ($CDCl_3$): δ 7.92 (d, *J* = 1.9 Hz, 1 H), 7.87 (d, *J* = 1.9 Hz, 1 H), 7.58-7.50 (m, 5 H), 6.70 (s, 1 H), 5.05 (d, *J* = 2.4 Hz, 2 H), 2.20 (t, *J* = 2.4 Hz, 1 H); ^{13}C NMR (100.6 MHz) ($CDCl_3$): δ 160.6 (C), 147.1 (C), 137.3 (C), 134.5 (C), 131.4 (CH), 126.7 (C), 126.3 (CH), 124.4 (C), 122.6 (C), 119.3 (CH), 115.1 (CH), 104.0 (CH), 78.0 (C), 77.5 (CH), 55.8 (CH_3), 37.2 (CH_2).

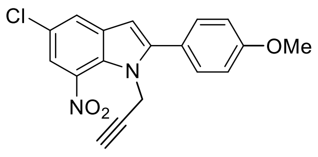

5-chloro-2-(4-methoxyphenyl)-7-nitro-1-(prop-2-yn-1-yl)-1*H*-indole 6b: 50 % yield; yellow solid; mp: 146 - 148 °C; ^{1}H NMR (400.13 MHz) (DMSO d_6): δ 8.12 (d, *J* = 1.7 Hz, 1 H), 7.96 (d, *J* = 1.7 Hz, 1 H), 7.56 (d, *J* = 8.6 Hz, 2 H), 7.14 (d, *J* = 8.6 Hz, 2 H), 6.80 (s, 1 H), 4.97 (d, *J* = 2.4 Hz, 2 H), 3.85 (s, 1 H), 3.33 - 3.30 (m, 1 H); ^{13}C NMR (100.6 MHz) (DMSO d_6): δ 160.5 (C), 146.8 (C), 137.2 (C), 134.4 (C), 130.9 (CH), 125.7 (CH), 125.1 (C), 122.8 (C), 119.5 (CH), 114.7 (C), 114.5 (CH), 103.5 (CH), 77.2 (C), 74.4 (CH), 55.45 (CH_3), 37.1 (CH_2).

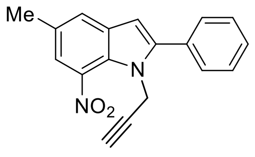

5-methyl-7-nitro-2-phenyl-1-(prop-2-yn-1-yl)-1*H*-indole 6c: 88 % yield; brown solid; mp: 94 - 96 °C; ^{1}H NMR (400.13 MHz, $CDCl_3$) δ 7.63 (s, 1H), 7.53 (s, 1H), 7.49–7.10 (m, 5H), 6.50 (s, 1H), 4.90 (d, *J* = 2.3 Hz, 2H), 2.35 (s, 3H), 1.98 (t, *J* = 2.3 Hz, 1H).^{13}C NMR (100.6 MHz) ($CDCl_3$): δ 145.6 (C), 137.1 (C), 133.7 (C), 131.3 (C), 129.9 (C), 129.6 (CH), 129.0 (CH), 129.0 (CH), 127.4 (C), 127.1 (CH), 121.2 (CH), 104.3 (CH), 77.68 (C), 73.93 (C), 36.94 (CH_2), 20.83 (CH_3).

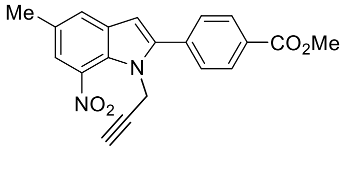

methyl 4-(5-methyl-7-nitro-1-(prop-2-yn-1-yl)-1H-indol-2-yl)benzoate 6d: 63 % yield; orange solid; mp 166 - 164 °C; ^{1}H NMR (400.13 MHz) ($CDCl_3$): δ 8.20 (d, *J* = 8.5 Hz, 2 H), 7.82 (s, 1 H), 7.74 (s, 1 H), 8.20 (d, *J* = 8.5 Hz, 2 H), 7.61 (d, *J* = 8.5 Hz, 2 H), 6.75 (s, 1 H), 5.05 (d, *J* = 2.4 Hz, 2 H), 3.99 (s, 3 H), 2.54 (s, 3 H), 2.17 (t, *J* = 2.4 Hz, 2 H); ^{13}C NMR (100.6 MHz) (400.13 MHz): δ 166.5 (C), 144.5 (C), 137.2 (C), 135.6 (C), 133.5 (C), 130.4 (C), 130.2 (C), 130.1 (CH), 129.3 (CH), 127.8 (C), 127.3 (CH), 121.8 (CH), 105.4 (CH), 77.3 (C), 74.0 (CH), 52.4 (CH_3), 37.1 (CH_2), 20.8 (CH_3).

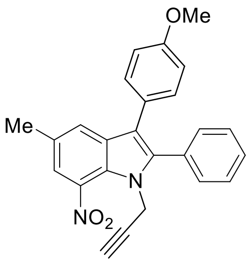

5-methyl-7-nitro-2-phenyl-1-(prop-2-yn-1-yl)-1*H*-indole 13: 70 % yield; orange liquid; ^{1}H NMR (400.13 MHz) ($CDCl_3$): δ 7.72 (d, *J* = 0.9 Hz, 1H), 7.66 (d, *J* = 0.9 Hz, 1H), 7.30 (m, 5H), 7.12–7.02 (m, 2H), 6.85–6.72 (m, 2H), 4.89 (d, *J* = 2.5 Hz, 2H), 3.73 (s, 3H), 2.43 (s, 3H), 2.10 (t, *J* = 2.4 Hz, 1H); ^{13}C NMR (100.6 MHz) ($CDCl_3$): δ 158.3 (C), 141.1 (C), 137.0 (C), 133.4 (C), 131.2 (CH), 131.1 (CH), 130.4 (C), 129.9 (C), 128.8 (CH), 128.7 (CH), 126.2 (C), 126.0 (CH), 125.7 (C), 121.5 (CH), 117.1 (C), 113.9 (CH), 77.8 (C), 73.8 (C), 55.2 (CH_3), 36.6 (CH_2), 20.9 (CH_3).

3.3 Characterization data of 7-nitro-2-aryl-1-(3-phenylprop-2-yn-1-yl)-1*H*-indole 7

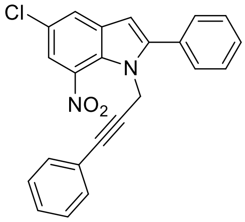

5-chloro-7-nitro-2-phenyl-1-(3-phenylprop-2-yn-1-yl)-1*H*-indole 7a: 73 % yield; yellow - orange solid; mp 152 - 153 °C; ^{1}H NMR (400.13 MHz) ($CDCl_3$): δ 7.92 (d, *J* = 1.9 Hz, 1 H), 7.88 (d, *J* = 1.9 Hz, 1 H), 7.61 - 7.52 (m, 5 H), 7.32 - 7.22 (m, 5 H), 6.72 (s, 1 H), 5.26 (s, 2 H); ^{13}C NMR (100.6 MHz) ($CDCl_3$): δ 146.8 (C), 137.5 (C), 134.2 (C), 131.7 (CH), 130.8 (C), 129.6 (CH), 129.3 (CH), 129.0 (CH), 128.7 (CH), 128.2 (CH), 127.1 (C), 125.9 (CH), 125.0 (C), 121.7 (C), 119.7 (CH), 103.9 (CH), 86.1 (C), 82.2 (C), 38.1 (CH_2).

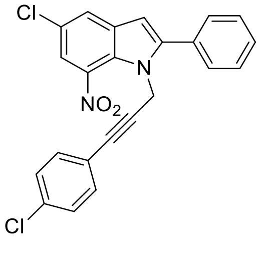

5-chloro-1-(3-(4-chlorophenyl)prop-2-yn-1-yl)-7-nitro-2-phenyl-1*H*-indole 7b: 73 % yield; orange solid; mp 109 - 110 °C; ^{1}H NMR (400.13 MHz) ($CDCl_3$): δ 7.91 (d, *J* = 1.7 Hz, 1 H), 7.88 (d, *J* = 1.7 Hz, 1 H), 7.62 - 7.50 (m, 5 H), 7.26 - 7.17 (m, 4 H), 6.72 (s, 1 H), 5.24 (s, 2 H); ^{13}C NMR (100.6 MHz) ($CDCl_3$): δ 146.8 (C), 137.4 (C), 134.9 (C), 134.2 (C), 133.0 (CH), 130.7 (C), 129.6 (CH), 129.4 (CH), 129.0 (CH), 128.6 (CH), 127.1 (C), 126.0 (CH), 125.1 (C), 120.2 (C), 119.7 (CH), 103.9 (CH), 85.1 (C), 83.3 (C), 38.0 (CH_2).

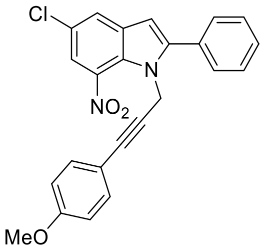

5-chloro-1-(3-(4-methoxyphenyl)prop-2-yn-1-yl)-7-nitro-2-phenyl-1*H*-indole 7c: 70 % yield; yellow solid; mp 133 - 134 °C; ^{1}H NMR (400.13 MHz) ($CDCl_3$): δ 7.91 (d, *J* = 1.9 Hz, 1 H), 7.87 (d, *J* = 1.9 Hz, 1 H), 7.60 - 7.50 (m, 5 H), 7.21 (d, *J* = 8.8 Hz, 2 H), 6.78 (d, *J* = 8.8 Hz, 2 H), 6.70 (s, 1 H), 5.23 (s, 2 H), 3.78 (s, 3 H); ^{13}C NMR (100.6 MHz) ($CDCl_3$): δ 159.9 (C), 146.7 (C), 138.2 (C), 137.5 (C), 134.2 (C), 133.2 (CH), 130.8 (C), 129.6 (CH), 129.3 (CH), 129.0 (CH), 127.1 (C), 125.8 (CH), 124.9 (C), 119.6 (CH), 113.8 (CH), 103.8 (CH), 86.1 (C), 80.9 (C), 55.2 (CH_3), 38.2 (CH_2).

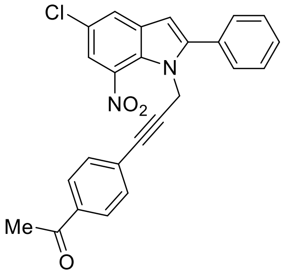

1-(4-(3-(5-chloro-7-nitro-2-phenyl-1*H*-indol-1-yl)prop-1-yn-1-yl)phenyl)ethan-1-one 7d: 52 % yield; brown solid; mp 133 - 134 °C; ^{1}H NMR (400.13 MHz) ($CDCl_3$): δ 7.92 (d, *J* = 1.9 Hz, 1 H), 7.90 (d, *J* = 1.9 Hz, 1 H), 7.84 (d, *J* = 8.5 Hz, 2 H), 7.60 - 7.53 (m, 5 H), 7.36 (d, *J* = 8.5 Hz, 2 H), 6.73 (s, 1 H), 5.27 (s, 2 H), 2.58 (s, 3 H); ^{13}C NMR (100.6 MHz) ($CDCl_3$): δ 197.2 (C), 146.8 (C), 137.4 (C), 136.6 (C), 134.2 (C), 131.9 (CH), 130.6 (C), 129.6 (CH), 129.4 (CH), 129.1 (CH), 128.1 (CH), 127.1 (C), 126.5 (C), 126.0 (CH), 125.2 (C), 119.7 (CH), 104.0 (CH), 85.4 (C), 85.3 (C), 38.0 (CH_2), 26.6 (CH_3).

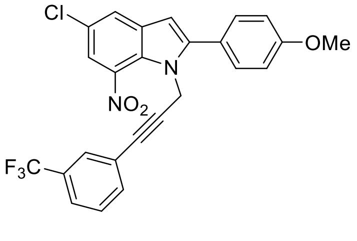

5-chloro-2-(4-methoxyphenyl)-7-nitro-1-(3-(3-(trifluoromethyl)phenyl)prop-2-yn-1-yl)-1*H*-indole 7e: 47 % yield; yellow - orange solid; mp 86 - 87 °C; ^{1}H NMR (400.13 MHz) ($CDCl_3$): δ 7.90 (d, *J* = 1.8 Hz, 1 H), 7.87 (d, *J* = 1.8 Hz, 1 H), 7.57 - 7.44 (m, 5 H), 7.40 (t, *J* = 7.6 Hz,, 1 H), 7.10 (d, *J* = 8.6 Hz, 2 H), 6.67 (s, 1 H), 5.24 (s, 2 H), 3.92 (s, 3 H); ^{13}C NMR (100.6 MHz) ($CDCl_3$): δ 160.5 (C), 146.7 (C), 137.3 (C), 135.0 (CH), 134.3 (C), 131.0 (CH), 130.8 (q, J_{CF} = 33 Hz, C), 128.8 (CH), 128.5 (q, J_{CF} = 4.0 Hz, CH), 126.9 (C), 125.7 (CH), 125.3 (q, J_{CF} = 4.0 Hz, CH), 125.1 (C), 123.5 (q, J_{CF} = 273 Hz, C), 122.9 (C), 122.7 (C), 119.4 (CH), 114.5 (CH), 103.4 (CH), 84.4 (C), 84.0 (C), 55.4 (CH_3), 37.8 (CH_2); ^{19}F NMR (376.5 MHz) ($CDCl_3$): δ -63.0.

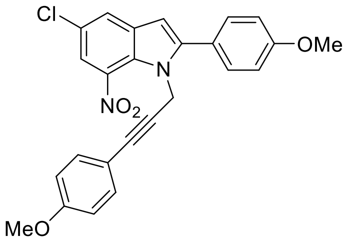

5-chloro-2-(4-methoxyphenyl)-1-(3-(4-methoxyphenyl)prop-2-yn-1-yl)-7-nitro-1*H*-indole 7f: 60 % yield; yellow - orange solid; mp 112 - 113 °C; ^{1}H NMR (400.13 MHz) ($CDCl_3$): δ 7.86 (d, *J* = 1.8 Hz, 1 H), 7.85 (d, *J* = 1.8 Hz, 1 H), 7.50 (d, *J* = 8.6 Hz, 2 H), 7.22 (d, *J* = 8.6 Hz, 2 H), 7.08 (d, *J* = 8.7 Hz, 2 H), 6.78 (d, *J* = 8.7 Hz, 2 H), 6.64 (s, 1 H), 5.20 (s, 2H), 3.92 (s, 3H), 3.78 (s, 3H); ^{13}C NMR (100.6 MHz) ($CDCl_3$): δ 160.4 (C), 159.9 (C), 146.7 (C), 137.4 (C), 134.3 (C), 133.2 (CH), 131.0 (CH), 127.0 (C), 125.6 (CH), 124.9 (C), 123.1 (C), 119.3 (CH), 114.5 (CH), 113.9 (C), 113.8 (CH), 103.2 (CH), 86.0 (C), 81.1 (C), 55.5(CH_3), 55.2 (CH_3), 38.1 (CH_2).

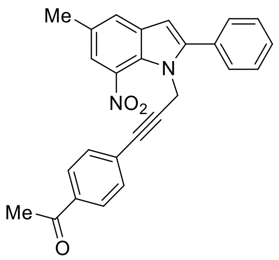

1-(4-(3-(5-methyl-7-nitro-2-phenyl-1*H*-indol-1-yl)prop-1-yn-1-yl)phenyl)ethan-1-one 7g: 73 % yield; yellow solid; mp 109 - 110 °C; ^{1}H NMR (400.13 MHz) ($CDCl_3$): δ 7.72 (d, *J* = 8.5 Hz, 2 H), 7.69 (s, 1 H), 7.63 (s, 1 H), 7.52 - 7.38 (m, 5 H), 7.22 (d, *J* = 8,. Hz, 2 H), 6.60 (s, 1 H), 5.18 (s, 2 H), 2.47 (s, 3 H), 2.44 (s, 3 H); ^{13}C NMR (100.6 MHz) ($CDCl_3$): δ 197.3 (C), 145.5 (C), 137.2 (C), 136.5 (C), 133.6 (C), 131.9 (CH), 131.3 (C), 129.8 (C), 129.6 (CH), 129.0 (CH), 128.9 (CH), 128.0 (CH), 127.3 (C), 127.0 (CH), 126.9 (C), 121.1 (CH), 104.1 (CH), 86.1 (C), 84.8 (C), 38.4 (CH_2), 26.7 (CH_3), 20.9 (CH_3).

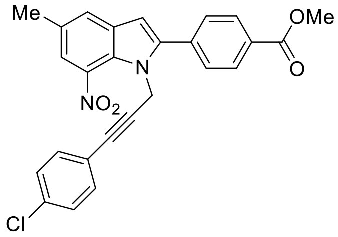

methyl 4-(1-(3-(4-chlorophenyl)prop-2-yn-1-yl)-5-methyl-7-nitro-1*H*-indol-2-yl)benzoate 7h: 58 % yield; yellow solid; mp 150 - 151 °C; ^{1}H NMR (400.13 MHz) ($CDCl_3$): δ 8.21 (d, *J* = 8.5 Hz, 2 H), 7.80 (s, 1 H), 7.73 (s, 1 H), 7.66 (d, *J* = 8.5, 2 H), 7.23 - 7.17 (m, 4 H), 6.76 (s, 1 H), 5.23 (s, 2 H), 4.00 (s, 3 H), 2.53 (s, 3 H); ^{13}C NMR (100.6 MHz) ($CDCl_3$): δ 166.5 (C), 144.3 (C), 137.3 (C), 135.6 (C), 134.7 (C), 133.4 (C), 133.0 (CH), 130.2 (C), 130.16 (CH), 130.13 (C), 129.4 (CH), 128.5 (CH), 127.8 (C), 127.2 (CH), 121.6 (CH), 120.3 (C), 105.1 (CH), 84.8 (C), 83.4 (C), 52.4 (CH), 38.0 (CH_2), 20.8 (CH_3).

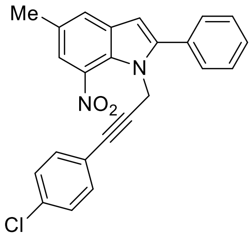

1-(3-(4-chlorophenyl)prop-2-yn-1-yl)-5-methyl-7-nitro-2-phenyl-1*H*-indole 7i: 63 % yield; yellow-orange solid; mp 122 - 123 °C; ^{1}H NMR (400.13 MHz) ($CDCl_3$): δ 7.78 (s, 1 H), 7.72 (s, 1 H), 7.62 - 7.42 (m, 5 H), 7.24 - 7.17 (m, 4 H), 6.69 (s, 1 H), 5.24 (s, 2 H), 2.53 (s, 3 H); ^{13}C NMR (100.6 MHz) ($CDCl_3$): δ 145.5 (C), 137.2 (C), 134.6 (C), 133.5 (C), 133.0 (CH), 131.3 (C), 129.8 (C), 129.6 (CH), 128.96 (CH), 128.94 (CH), 128.5 (CH), 127.2 (C), 127.0 (CH), 121.1 (CH), 120.5 (C), 104.1 (CH), 84.6 (C), 83.8 (C), 37.8 (CH_2), 20.8 (CH_3).

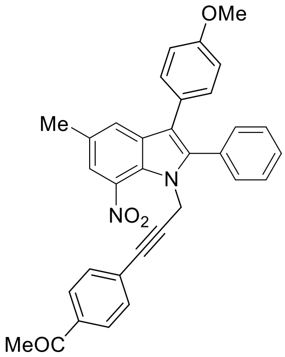

1-(4-(3-(3-(4-methoxyphenyl)-5-methyl-7-nitro-2-phenyl-1*H*-indol-1-yl)prop-1-yn-1-yl)phenyl)ethan-1-one 14: 92 % yield; yellow solid; mp: 200 - 202 °C; ^{1}H NMR (400.13 MHz) ($CDCl_3$): δ 7.85 (d: *J* = 8.3 Hz, 2 H), 7.81 (s, 1 H), 7.76 (s, 1 H), 7.49 - 7.35 (m, 7 H), 7.19 (d, *J* = 8.7 Hz, 2 H), 6.89 (d, *J* = 8.7 Hz, 2 H), 5.20 (s, 2 H), 3.83 (s, 3 H), 2.58 (s, 3 H), 2.52 (s, 3 H); ^{13}C NMR (100.6 MHz) ($CDCl_3$): δ 197.3 (C), 158.3 (C), 140.9 (C), 137.1 (C), 136.5 (C), 133.3 (C), 131.9 (CH), 131.2 (CH), 131.1 (CH), 129.8 (CH), 128.9 (CH), 128.8 (CH), 128.1 (C), 127.0 (C), 126.2 (C), 126.0 (CH), 125.7 (C), 121.4 (CH), 116.9 (C), 114.0 (CH), 104.1 (C), 86.3 (C), 84.8 (CH), 55.2 (CH_3), 37.6 (CH_2), 26.6 (CH_3), 55.2 (CH_3),.20.9 (CH_3).

3.4 Characterization data of 2-aryl-1-(3-phenylprop-2-yn-1-yl)-1*H*-indol-7-amines 1

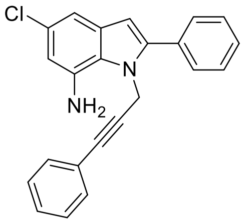

5-chloro-2-phenyl-1-(3-phenylprop-2-yn-1-yl)-1*H*-indol-7-amine 1a: 60% yield; brown oil; ^{1}H NMR (400.13 MHz) ($CDCl_3$): δ 7.68 (d, *J* = 8.8 Hz, 2 H), 7.56 - 7.43 (m, 5 H), 7.41 - 7.34 (m, 3 H), 7.01 (d, *J* = 1.6 Hz, 1 H), 6.47 (s, 1 H), 6.50 (d, *J* = 1.6 Hz, 1 H), 5.19 (s, 2 H), 4.33 (bs, 2 H); ^{13}C NMR (100.6 MHz) ($CDCl_3$): δ 143.2 (C), 134.0 (C), 131.8 (C), 131.7 (CH), 130.9 (C), 129.3 (CH), 129.0 (CH), 128.8 (CH), 128.49 (CH), 128.43 (CH), 127.2 (C), 126.4 (C), 121.8 (C), 111.4 (CH), 110.3 (CH), 102.5 (CH), 86.5 (C), 86.2 (C), 36.5 (CH_2).

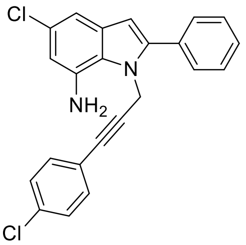

5-chloro-1-(3-(4-chlorophenyl)prop-2-yn-1-yl)-2-phenyl-1*H*-indol-7-amine 1b: 55% yield; yellow - orange solid; mp 109 - 110 °C; ^{1}H NMR (400.13 MHz) ($CDCl_3$): δ 7.60 - 7.64 (m, 2 H), 7.56 - 7.45 (m, 3 H), 7.41 (d, *J* = 8.6 Hz, 2 H), 7.34 (d, *J* = 8.6 Hz, 2 H), 7.11 (d, *J* = 1.9 Hz, 1 H), 6.56 (d, *J* = 1.9 Hz, 1 H), 6.48 (s, 1 H), 5.19 (s, 2 H), 4.27 (bs, 2 H); ^{13}C NMR (100.6 MHz) ($CDCl_3$): δ 143.3 (C), 135.1 (C), 133.9 (C), 132.9 (CH), 131.8 (C), 130.9 (C), 129.3 (CH), 128.87 (CH), 128.83 (CH), 128.5 (CH), 127.2 (C), 126.5 (C), 120.3 (C), 111.6 (CH), 110.5 (CH), 102.7 (CH), 87.2 (C), 85.4 (C), 36.4 (CH_2).

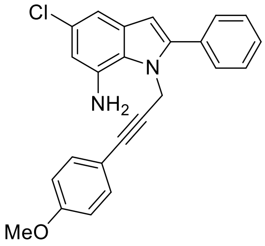

5-chloro-1-(3-(4-methoxyphenyl)prop-2-yn-1-yl)-2-phenyl-1*H*-indol-7-amine 1c: 85 % yield; yellow-orange solid; mp 91 - 92 °C; ^{1}H NMR (400.13 MHz) ($CDCl_3$): δ 7.69 (d, *J* = 7.2 Hz, 2 H), 7.52 (t, *J* = 7.5 Hz, 2 H), 7.48 - 7.40 (m, 3 H), 6.99 (d, *J* = 1.8 Hz, 1 H), 7.00 (d, *J* = 1.9 Hz, 1 H), 6.89 (d, *J* = 8.8 Hz, 2 H), 6.55 (d, *J* = 1.9 Hz, 1 H), 6.46 (s, 1 H), 5.17 (s, 2 H), 4.35 (bs, 2 H), 3.8 (s, 3 H); ^{13}C NMR (100.6 MHz) ($CDCl_3$): δ 160.1 (C), 143.2 (C), 134.0 (C), 133.2 (CH), 131.9 (C), 130.8 (C), 129.3 (CH), 128.7 (CH), 128.3 (CH), 127.1 (C), 126.3 (C), 114.1 (CH), 113.8 (C), 111.3 (CH), 110.1 (CH), 102.4 (CH), 86.6 (C), 84.9 (C), 55.3 (CH_3), 36.6 (CH_2).

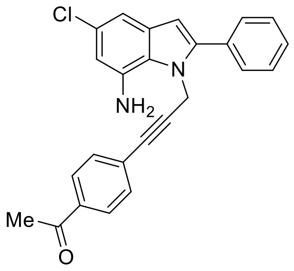

1-(4-(3-(7-amino-5-chloro-2-phenyl-1*H*-indol-1-yl)prop-1-yn-1-yl)phenyl)ethan-1-one 1d: 81 % yield; brown solid; mp 80 - 81 °C; ^{1}H NMR (400.13 MHz) (DMSO d_6): δ 7.90 (d, J_1 = 8.4 Hz, 2 H), 7.60 (d, J = 7.6 Hz, 2 H), 7.55 (t, J = 7.5 Hz, 2 H), 7.48- 7.44 (m, 3 H), 6.71 (s, 1 H), 6.46 (s, 1 H), 6.40 (d, J = 1.1 Hz, 1 H), 5.31 (s, 2 H), 4.89 (bs, 2 H), 2.55 (s, 3 H); ^{13}C NMR (100.6 MHz) (DMSO d_6): δ 197.7 (C), 142.7 (C), 136.8 (C), 134.9 (C), 132.6 (C), 132.1 (CH), 131.0 (C), 130.5 (C), 129.4 (CH), 129.2 (CH), 128.9 (CH), 128.8 (CH), 127.6 (C), 126.7 (C), 112.2 (CH), 110.8 (CH), 104.4 (CH), 90.2 (C), 84.0 (C), 36.3 (CH_2), 27.2 (CH_3).

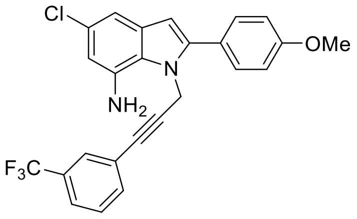

5-chloro-2-(4-methoxyphenyl)-1-(3-(3-(trifluoromethyl)phenyl)prop-2-yn-1-yl)-1*H*-indol-7-amine 1e: 93 % yield; brown solid; mp 101 - 102 °C; ^{1}H NMR (400.13 MHz) ($CDCl_3$): δ 7.73 (s, 1 H), 7.67 - 7.61 (m, 2 H), 7.58 (d, J = 8.6 Hz, 2 H), 7.5 (t, J = 7.8 Hz, 1 H), 7.09 (d, J = 1.5 Hz, 1 H), 7.06 (d, J = 8.6 Hz, 2 H), 6.55 (d, J = 1.5 Hz, 1 H), 6.42 (s, 1 H), 5.20 (s, 2 H), 4.21 (bs, 2 H), 3.90 (s, 3 H); ^{13}C NMR (100.6 MHz) ($CDCl_3$): δ 159.9 (C), 143.2 (C), 134.9 (CH), 133.7 (C), 131.2 (q, J_{CF} = 32 Hz, C), 131.0 (C), 130.7 (CH), 129.1 (CH), 128.5 (q, J_{CF} = 4 Hz, CH), 127.0 (C), 126.4 (C), 125.8 (q, J_{CF} = 4 Hz, CH), 124.1 (C), 123.5 (q, J_{CF} = 273 Hz, C), 122.8 (CH), 114.3 (CH), 111.5 (CH), 110.4 (CH), 102.1 (CH), 87.9 (C), 84.7 (C), 55.4 (CH_3), 36.3 (CH_2); ^{19}F NMR (376.5 MHz) ($CDCl_3$): δ -62.6.

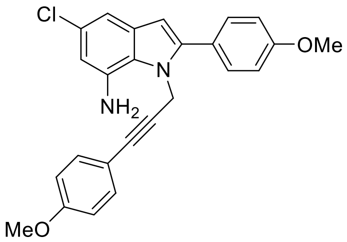

5-chloro-2-(4-methoxyphenyl)-1-(3-(4-methoxyphenyl)prop-2-yn-1-yl)-1*H*-indol-7-amine 1f: 95 % yield; brown solid; mp 105 - 106 °C; ^{1}H NMR (400.13 MHz) ($CDCl_3$): δ 7.61 (d, J = 8.6 Hz, 2 H), 7.42 (d, J =8.4 Hz, 2 H), 7.09 - 7.02 (m, 3 H), 6.88 (d, J =8.4 Hz, 2 H), 6.52 (d, J =1.4 Hz, 2 H), 6.39 (s, 1 H), 5.15 (s, 2 H), 4.33 (bs, 2 H), 3.89 (s, 3 H), 3.85 (s, 3 H); ^{13}C NMR (100.6 MHz) ($CDCl_3$): δ 160.1 (C), 159.8 (C), 143.1 (C), 134.0 (C), 133.2 (CH), 130.9 (C), 130.6 (CH), 126.9 (C), 126.2 (C), 124.3 (C), 120.4 (C), 114.2 (CH), 114.1 (CH), 111.2 (CH), 109.9 (CH), 101.7 (CH), 86.4 (C), 85.0 (C), 55.4 (CH_3), 55.3 (CH_2), 36.5 (CH_2).

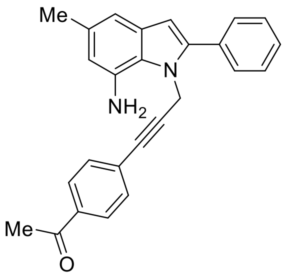

1-(4-(3-(7-amino-5-methyl-2-phenyl-1*H*-indol-1-yl)prop-1-yn-1-yl)phenyl)ethan-1-one 1g: 70 % yield; yellow - orange solid; mp 83 - 84 °C; ^{1}H NMR (400.13 MHz, $CDCl_3$) δ 7.84 (d, *J* = 8.2 Hz, 2H), 7.58 (d, *J* = 7.3 Hz, 2H), 7.50–7.30 (m, 5H), 6.85 (s, 1H), 6.38-6.35 (m, 2H), 5.15 (s, 2H), 4.04 (s, 2H), 2.53 (s, 3H), 2.29 (s, 3H); ^{13}C NMR (100.6 MHz) ($CDCl_3$): δ 197.2 (C), 142.4 (C), 136.7 (C), 132.5 (C), 132.4 (C), 131.9 (CH), 131.0 (C), 130.7 (C), 129.3 (CH), 128.7 (CH), 128.3 (CH), 128.1 (CH), 127.4 (C), 126.9 (C), 112.4 (CH), 112.3 (CH), 102.9 (CH), 90.0 (C), 85.2 (C), 36.5 (CH_2), 26.7 (CH_3), 21.3 (CH_3).

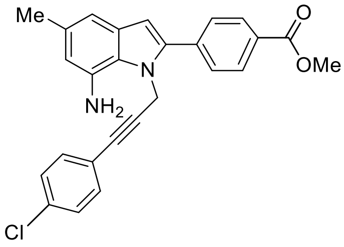

methyl 4-(7-amino-1-(3-(4-chlorophenyl)prop-2-yn-1-yl)-5-methyl-1*H*-indol-2-yl)benzoate 1h: 63 % yield; red wax; ^{1}H NMR (400.13 MHz) ($CDCl_3$): δ 8.08 (d, *J* = 8.5 Hz, 2H), 7.67 (d, *J* = 8.5 Hz, 2H), 7.28 (m, 4H), 6.85 (s, 1H), 6.46 (s, 1H), 6.36 (d, *J* = 1.0 Hz, 1H), 5.10 (s, 2H), 4.06 (s, 2H), 3.88 (s, 3H), 2.29 (s, 3H); ^{13}C NMR (100.6 MHz, $CDCl_3$) δ 166.8 (C), 141.3 (C), 136.8 (C), 135.1 (C), 133.0 (CH), 132.7 (C), 131.2 (C), 130.5 (C), 130.0 (CH), 129.4 (C), 128.9 (CH), 128.9 (CH), 128.1 (C), 120.4 (C), 112.8 (CH), 112.4 (CH), 104.0 (CH), 87.4 (C), 85.3 (C), 52.3 (CH3), 36.6 (CH_2), 21.3 (CH_3).

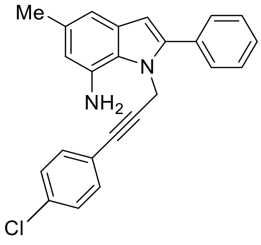

1-(3-(4-chlorophenyl)prop-2-yn-1-yl)-5-methyl-2-phenyl-1H-indol-7-amine 1i: 57 % yield; brown wax; ^{1}H NMR (400.13 MHz) ($CDCl_3$): δ 7.57 (d, *J* = 8.0 Hz, 2H), 7.40 (t, *J* = 8.0, 7.0 Hz, 2H), 7.37–7.27 (m, 3H), 7.23 (d, *J* = 8.5 Hz, 2H), 6.84 (s, 1H), 6.37–6.33 (m, 1H), 5.10 (s, 2H), 4.05 (s, 2H), 2.28 (s, 3H); ^{13}C NMR (100.6 MHz) ($CDCl_3$): δ 142.3 (C), 135.0 (C), 133.0 (CH), 132.6 (C), 132.5 (C), 130.9 (C), 130.6 (C), 129.3 (CH), 128.8 (CH), 128.7 (CH), 128.1 (CH), 127.4 (C), 120.6 (C), 112.3 (CH), 112.2 (CH), 102.7 (CH), 87.8 (C), 85.0 (C), 36.4 (CH_2), 21.3 (CH_3).

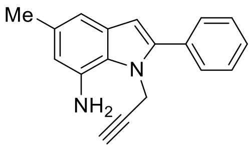

5-methyl-2-phenyl-1-(prop-2-yn-1-yl)-1H-indol-7-amine 1j: 43 % yield; brown wax; ^{1}H NMR (400.13 MHz) ($CDCl_3$): δ 7.55 (d, *J* = 7.1 Hz, 2H), 7.42–7.31 (m, 3H), 6.83 (s, 1H), 6.35 - 6.33 (m, 2H), 4.89 (d, *J* = 2.4 Hz, 2H), 4.00 (s, 2H), 2.52 (t, *J* = 2.4 Hz, 1H), 2.28 (s, 3H).^{13}C NMR (100.6 MHz) ($CDCl_3$): 142.4 (C), 132.5 (C), 132.4 (C), 130.9 (C), 130.6 (C), 129.3 (CH), 128.7 (CH), 128.1 (CH), 112.4 (CH), 112.3 (CH), 102.8 (CH), 81.9 (C), 74.4 (C), 35.7 (CH_2), 21.3 (CH_3).

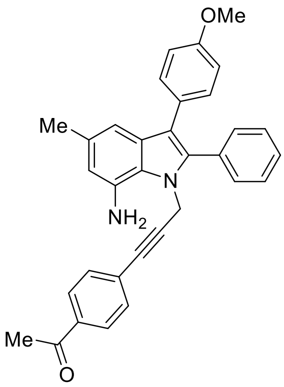

1-(4-(3-(7-amino-3-(4-methoxyphenyl)-5-methyl-2-phenyl-1*H*-indol-1-yl)prop-1-yn-1-yl)phenyl)ethan-1-one 1k: 57 % yield; brown wax; ^{1}H NMR (400.13 MHz) ($CDCl_3$): δ 7.84 (d, *J* = 8.4 Hz, 2H), 7.45 (d, *J* = 8.4 Hz, 2H), 7.38–7.29 (m, 5H), 7.12 (d, *J* = 8.7 Hz, 2H), 6.93 (s, 1H), 6.76 (d, *J* = 8.8 Hz, 2H), 6.40 (s, 1H), 5.10 (s, 2H), 4.05 (m, 2H), 3.72 (s, 3H), 2.53 (s, 3H), 2.28 (s, 3H); ^{13}C NMR (100.6 MHz) ($CDCl_3$): δ 197.2 (C), 157.7 (C), 138.1 (C), 136.7 (C), 132.5 (C), 131.9 (CH), 131.5 (C), 131.3 (CH), 131.1 (CH), 130.1 (C), 128.5 (CH), 128.3 (CH), 128.1 (CH), 127.5 (C), 127.0 (C), 126.3 (C), 115.8 (C), 113.7 (CH), 112.9 (CH), 111.3 (CH), 89.9 (C), 85.0 (C), 55.2 (CH_3), 36.3 (CH_2), 26.7 (CH_3), 21.4 (CH_3).

4. REFERENCES

[1] Sanz R., Guilarte V., Pérez A., Straightforward selective preparation of nitro- or amino-indoles from 2-halonitroanilines and alkynes. First synthesis of 7-amino-5-nitroindoles, *Tetrahedron Lett.*, **2009**, *50,* 4423-4426, doi.org/10.1016/j.tetlet.2009.05.027.

5. HF CALCULATION ON ISOMERS 2a and 2'a

Compound 2a

```
+------------------------------------------------------------------
---+
  |  Jaguar version 3.5, release  42
|
  |
|
  |  Copyright 1991-1998 Schrodinger, Inc.
|
  |  All Rights Reserved.
|
  |
|
  |  Use of this program should be acknowledged in publications
as:    |
  |  Jaguar 3.5, Schrodinger, Inc., Portland, Oregon, 1998.
|
  +------------------------------------------------------------------
-----+

  start of program pre
  Job name: WF3654
  Executables used: D:\TITAN
  Temporary files : C:\Users\gfabr\AppData\Local\Temp\WF3654

  Input file comments:
    Molecule001
    This file created by Spartan

  basis set:             6-31G**
  net molecular charge:    0
  multiplicity:            1

  number of basis functions....          465

 Input geometry:
                               angstroms
  atom           x                 y                 z
  N1       -0.1550260000      0.4374990000      1.1210390000
  N2       -1.0086730000     -2.3256400000      1.1332130000
  C4       -1.1860510000     -0.0350800000      1.9315790000
  C5       -0.0104550000     -1.9237530000      0.4028750000
  C6       -1.6456290000     -1.3760560000      1.9632780000
  C7       -1.7070180000      1.0262900000      2.7388420000
  C13       0.7199200000     -2.8803430000     -0.5063790000
  H4       -3.1185930000     -2.6496450000      2.9223950000
  C8       -2.7656460000      0.7093510000      3.5969080000
  C11      -0.9132890000      2.1852800000      2.3994340000
```

```
 H7       -3.2133600000      1.4833470000      4.2359600000
 C9       -3.2381490000     -0.5972350000      3.6279590000
 H8       -4.0721010000     -0.8455450000      4.3029390000
 C10      -2.7017240000     -1.6349910000      2.8394980000
 C12       0.0268720000      1.8011420000      1.4273890000
 C2        1.0263770000      2.6560040000      0.8075810000
 H13       1.8204060000     -2.6663390000     -0.4286580000
 C14       0.2610050000     -2.7449900000     -1.9177470000
 H15       0.5612370000     -3.9425620000     -0.1705390000
 C15      -0.5895550000     -2.5433530000     -4.5760680000
 C16       1.1246910000     -2.2456410000     -2.8988340000
 C17      -1.0320590000     -3.1444790000     -2.2771670000
 C18      -1.4542200000     -3.0419090000     -3.6012120000
 C19       0.6998040000     -2.1465450000     -4.2231290000
 H16       2.1442750000     -1.9343460000     -2.6271240000
 H17      -1.7134540000     -3.5365780000     -1.5059970000
 H18      -2.4719630000     -3.3556810000     -3.8767030000
 H19       1.3846780000     -1.7555640000     -4.9897570000
 H20      -0.9239070000     -2.4642400000     -5.6207450000
 C1        0.5776390000     -0.5062530000      0.3222060000
 H5        1.6528730000     -0.5444960000      0.6633050000
 H6        0.5619750000     -0.1829560000     -0.7593680000
 C3        2.9246780000      4.3652120000     -0.3378590000
 C26       1.7455110000      3.5549090000      1.6101580000
 C27       1.2625130000      2.6251890000     -0.5732690000
 C28       2.2088580000      3.4770190000     -1.1402160000
 C29       2.6895760000      4.4036320000      1.0366540000
 H1        1.5580180000      3.5843390000      2.6943120000
 H2        0.6891520000      1.9402470000     -1.2156630000
 H3        2.3868000000      3.4502800000     -2.2254160000
 H9        3.2505630000      5.1053430000      1.6714530000
 H10       3.6710440000      5.0358360000     -0.7881560000
 H25      -1.0275920000      3.1833010000      2.8110280000

 Molecular weight:    322.15 amu

 Stoichiometry:        C23N2H18
 Molecular Point Group: C1
 Point Group used: C1
 nuclear repulsion energy.......  1952.777623249 hartrees

Non-default options chosen:
  Geometry will be optimized in redundant internal coordinates
  Initial Hessian: from previous calculation

 end of program pre


 start of program onee
 smallest eigenvalue of S:    3.218E-04
 number of canonical orbitals.....          462
 end of program onee
```

```
  start of program hfig
  initial wavefunction generated automatically from atomic
wavefunctions

  Irreducible     Total no   No of occupied orbitals
  representation  orbitals   Shell_1  Shell_2    ...
  No Symm          462         85
  ------------------------
  Orbital occupation/shell    1.000

  end of program hfig


  start of program probe
  end of program probe


  start of program grid

  number of gridpoints:
     atom         N1     N2      C4      C5      C6      C7     C13
H4
  grid # 1        97    102      88      88      88      90      84
73
  grid # 2       104    112      97      95      98      98      92
118
  grid # 3       214    229     199     184     197     196     170
223
  grid # 4       391    414     331     322     327     347     304
232

  number of gridpoints:
     atom         C8    C11      H7      C9      H8     C10     C12
C2
  grid # 1        89     87      73      87      73      89      86
90
  grid # 2        97     95     118      97     118      97      97
98
  grid # 3       186    182     224     184     222     186     191
195
  grid # 4       332    341     234     329     231     332     331
341

  number of gridpoints:
     atom        H13    C14     H15     C15     C16     C17     C18
C19
  grid # 1        69     93      69      89      88      89      89
89
  grid # 2       110    100     112      97      96      96      97
97
```

```
  grid # 3      210    195    216    187    186    185    186
185
  grid # 4      215    342    223    331    330    331    331
330

  number of gridpoints:
     atom       H16    H17    H18    H19    H20     C1     H5
H6
  grid # 1       72     72     73     73     73     82     70
69
  grid # 2      112    114    118    118    118     90    112
102
  grid # 3      214    217    224    223    224    167    216
205
  grid # 4      221    223    232    231    232    299    217
206

  number of gridpoints:
     atom        C3    C26    C27    C28    C29     H1     H2
H3
  grid # 1       89     89     88     89     89     72     71
73
  grid # 2       97     96     96     97     97    115    111
118
  grid # 3      187    184    186    186    186    218    212
224
  grid # 4      332    330    330    329    330    224    217
232

  number of gridpoints:
     atom        H9    H10    H25  total
  grid # 1       73     73     72   3521
  grid # 2      118    118    115   4498
  grid # 3      224    224    219   8672
  grid # 4      232    232    222  12443

  end of program grid


  start of program rwr
  end of program rwr


  start of program scf
 number of electrons..........        170
 number of alpha electrons....         85
 number of beta electrons.....         85
 number of orbitals, total....        462
 number of core orbitals......         85
 number of open shell orbs....          0
 number of occupied orbitals..         85
 number of virtual orbitals...        377
```

```
 number of hamiltonians.......          1
 number of shells.............          1
 SCF type: HF

       i  u  d  i  g
       t  p  i  c  r                                  RMS    maximum
       e  d  i  u  i                       energy  density    DIIS
       r  t  s  t  d     total energy      change   change     error

etot   1  N  N  5  M  -987.21080013367              3.9E-03  9.8E-02
etot   2  Y  Y  6  M  -989.98476104696  2.8E+00  1.7E-03  5.5E-02
etot   3  Y  Y  6  M  -990.27640419982  2.9E-01  7.8E-04  2.7E-02
etot   4  N  Y  2  U  -990.34536478481  6.9E-02  3.8E-04  1.5E-02
etot   5  Y  Y  6  M  -990.35620931742  1.1E-02  1.0E-03  1.0E-02
etot   6  N  Y  2  U  -990.36578188544  9.6E-03  1.2E-04  2.2E-03
etot   7  Y  Y  6  M  -990.36633784606  5.6E-04  4.0E-05  5.6E-04
etot   8  Y  Y  6  M  -990.36641184175  7.4E-05  1.2E-05  2.3E-04
etot   9  N  Y  2  U  -990.36623705176 -1.7E-04  5.8E-06  1.1E-04
etot  10  Y  Y  6  M  -990.36624305181  6.0E-06  4.9E-06  5.8E-05
etot  11  Y  N  6  M  -990.36624587679  2.8E-06  0.0E+00  0.0E+00


 Energy components, in hartrees:
   (A)  Nuclear repulsion............  1952.77762324898
   (E)  Total one-electron terms..... -5223.87627037117
   (I)  Total two-electron terms.....  2280.73240124540
   (L)  Electronic energy............ -2943.14386912577  (E+I)
   (N)  Total energy.................  -990.36624587679  (A+L)

 SCFE: SCF energy: HF    -990.36624587679 hartrees   iterations:
11


 HOMO energy:    -0.26238
 LUMO energy:     0.08458

  Orbital energies:
  -15.61225  -15.57394  -11.30699  -11.29988  -11.28257  -11.26176
  -11.25976  -11.25489  -11.25065  -11.24883  -11.24706  -11.24570
  -11.24527  -11.24438  -11.24359  -11.24267  -11.24138  -11.24124
  -11.23937  -11.23733  -11.22956  -11.22835  -11.22767  -11.22333
  -11.22233   -1.30891   -1.23521   -1.15989   -1.15811   -1.13132
   -1.07044   -1.06879   -1.03483   -1.01840   -1.01489   -1.01367
   -0.96165   -0.95090   -0.92391   -0.85859   -0.83575   -0.83045
   -0.82627   -0.79990   -0.78600   -0.76574   -0.72486   -0.71505
   -0.69233   -0.69090   -0.66232   -0.65281   -0.64039   -0.63511
   -0.63125   -0.61793   -0.61598   -0.61045   -0.59401   -0.58832
   -0.58652   -0.58354   -0.57683   -0.55347   -0.54722   -0.54082
   -0.53565   -0.51410   -0.50599   -0.49981   -0.49872   -0.49409
   -0.49103   -0.48832   -0.48378   -0.47850   -0.42632   -0.40843
   -0.36790   -0.34360   -0.34155   -0.33783   -0.32751   -0.27573
   -0.26238    0.08458    0.11895    0.13358    0.13414    0.14570
```

```
   0.17403    0.20148    0.21421    0.22686    0.23450

 end of program scf


 start of program der1a
 end of program der1a


 start of program rwr
recomputing RwR matrix   2    grid:  4
 end of program rwr


 start of program der1b

 forces (hartrees/bohr) : total

atom  label          x              y              z
----  -----   -------------  -------------  -------------
  1    N1      -3.240872E-02   9.749688E-03   3.179943E-02
  2    N2       3.994841E-02   1.589784E-02  -2.744144E-02
  3    C4      -4.648588E-03   1.109702E-02   6.253104E-03
  4    C5      -3.592959E-02  -8.241299E-03   3.439027E-02
  5    C6       2.414258E-03   2.916011E-02   4.891493E-03
  6    C7       2.172547E-02  -2.498377E-02  -2.655680E-02
  7    C13      1.776470E-02  -2.489095E-02   2.423915E-02
  8    H4       8.099431E-03   1.571016E-02  -2.748483E-03
  9    C8      -8.989727E-03   2.813047E-03   8.737148E-03
 10    C11      2.073446E-02  -1.403474E-02  -2.135011E-02
 11    H7       5.617432E-03  -1.253712E-02  -8.781297E-03
 12    C9      -1.066947E-02   1.935235E-03   1.014118E-02
 13    H8       1.338669E-02   4.465009E-03  -1.056642E-02
 14    C10      7.534698E-03  -9.961889E-03  -9.389467E-03
 15    C12     -4.116184E-02  -1.291056E-02   3.163420E-02
 16    C2       1.304881E-02   8.436034E-03  -7.725578E-03
 17    H13     -2.288695E-02  -5.875229E-03  -2.856836E-03
 18    C14     -3.001289E-05   9.115626E-04  -2.375293E-02
 19    H15      2.454104E-03   2.667852E-02  -3.093814E-03
 20    C15     -8.755934E-04   1.284549E-03  -8.246274E-03
 21    C16      1.904926E-03   8.743392E-04  -2.548369E-04
 22    C17     -5.241454E-03  -1.814770E-03   2.230640E-03
 23    C18     -7.258278E-03  -2.346623E-03  -2.041341E-04
 24    C19      3.649640E-03   3.130199E-03  -7.320720E-03
 25    H16     -1.529594E-02  -5.126618E-03  -3.849080E-03
 26    H17      1.141142E-02   6.616599E-03  -1.234182E-02
 27    H18      1.586235E-02   4.779575E-03   4.278800E-03
 28    H19     -1.052798E-02  -5.951830E-03   1.156894E-02
 29    H20      5.229882E-03  -1.242672E-03   1.606278E-02
 30    C1       2.113894E-02   9.772674E-03  -1.632786E-02
 31    H5      -2.245682E-02  -6.894493E-04  -1.106135E-02
 32    H6       4.269849E-03  -1.216673E-02   2.603804E-02
```

```
  33    C3      5.645640E-03   4.899727E-03  -3.657363E-03
  34    C26     4.904907E-04   2.576969E-03   4.717966E-03
  35    C27    -4.000485E-04  -2.665549E-03  -5.344084E-03
  36    C28     1.194079E-03  -8.729806E-04  -7.925486E-03
  37    C29     2.773262E-03   5.466727E-03   5.063842E-03
  38    H1      3.964567E-03  -1.650721E-03  -1.828743E-02
  39    H2      8.982905E-03   1.280343E-02   1.060548E-02
  40    H3     -2.910655E-03   4.951888E-04   1.678907E-02
  41    H9     -8.627735E-03  -1.102210E-02  -9.843115E-03
  42    H10    -1.152274E-02  -1.049380E-02   6.921504E-03
  43    H25     2.708121E-03  -9.050340E-03  -6.693826E-03
 -----------   -------------  -------------  -------------
   total        1.123978E-04   1.024489E-03   7.424633E-04

  end of program der1b


  start of program geopt   1

  geometry optimization step  1
  reading input hessian of dimension   129
   in five columns format


  Level shifts adjusted to satisfy step-size constraints
   Step size:    0.3000165
   Cos(theta):   0.8700386

   Final level shift:  -9.0459602E-02

  gradient maximum:        5.6400E-02 .  (  4.5000E-04 )
  gradient rms:            1.1269E-02 .  (  3.0000E-04 )
  step size:   0.30001  trust radius:   0.30000
  displacement maximum:    7.9070E-02 .  (  1.8000E-03 )
  displacement rms:        2.3571E-02 .  (  1.2000E-03 )
  predicted energy change:    -2.2790E-02    geom step:   3.0001E-
01    full step:   3.0001E-01
  molecular structure not yet converged...

  center of mass moved by:
     x:     1.7900E-03     y:     7.9249E-03     z:     5.2280E-03

  new geometry:
                              angstroms
  atom           x                  y                 z
  N1       -0.1851238607      0.4641584805      1.1496398783
  N2       -0.9624306185     -2.2803275995      1.1391335864
  C4       -1.1923369589     -0.0180925749      1.9481977188
  C5        0.0003838455     -1.8866472314      0.4304247849
  C6       -1.6131242204     -1.3444054000      1.9677332987
  C7       -1.7143322129      1.0107834641      2.7508560527
  C13       0.6888484121     -2.8743700008     -0.4705903976
```

```
H4       -3.0326107871     -2.6499595048      2.8909113274
C8       -2.7758076385      0.6768625265      3.6172856202
C11      -0.9256685288      2.1808482739      2.4168546928
H7       -3.2270829119      1.4262828740      4.2504106118
C9       -3.2256779072     -0.6251283660      3.6377251029
H8       -4.0393570567     -0.8900121801      4.2984882981
C10      -2.6561623240     -1.6393208618      2.8310281808
C12      -0.0161416855      1.8156812458      1.4563175807
C2        1.0081011308      2.6662618384      0.8151804166
H13       1.7673251388     -2.7356321765     -0.3960304160
C14       0.2466641767     -2.7462605278     -1.9265734159
H15       0.4459377797     -3.8754027525     -0.1219375680
C15      -0.5453795077     -2.5531842005     -4.6103164906
C16       1.1228101928     -2.2821169158     -2.9004344929
C17      -1.0389296276     -3.1210357258     -2.3122887225
C18      -1.4308521122     -3.0220378034     -3.6483671644
C19       0.7311661555     -2.1855070811     -4.2360240634
H16       2.1179862862     -2.0041675136     -2.6156208078
H17      -1.7271848709     -3.4681454408     -1.5658608400
H18      -2.4200252396     -3.2868875229     -3.9394618092
H19       1.4207969829     -1.8273231853     -4.9765738859
H20      -0.8415814914     -2.4643443665     -5.6434658446
C1        0.5813167973     -0.4636626778      0.3433044617
H5        1.6400268303     -0.4816687726      0.6523424559
H6        0.5606286448     -0.1702832088     -0.7202931642
C3        2.9112386307      4.3243859975     -0.3688081175
C26       1.7641520157      3.5436729388      1.5963361301
C27       1.2174113891      2.6356426600     -0.5675864813
C28       2.1639530001      3.4567854144     -1.1533354185
C29       2.7066639823      4.3656913408      1.0056564678
H1        1.6123726912      3.5626682239      2.6583667114
H2        0.6171678014      1.9866834111     -1.1807727574
H3        2.3092217370      3.4311451005     -2.2201183904
H9        3.2922168597      5.0359612586      1.6129911517
H10       3.6489292197      4.9615184331     -0.8293143894
H25      -1.0462406649      3.1705625677      2.8156287502

nuclear repulsion energy.......  1960.025589350 hartrees
-------------------------------------------------
/  end of geometry optimization iteration   1   /
-------------------------------------------------

end of program geopt


start of program onee
smallest eigenvalue of S:    2.977E-04
number of canonical orbitals.....          461
end of program onee


start of program probe
```

```
  end of program probe


  start of program grid

  number of gridpoints:
     atom        N1     N2     C4     C5     C6     C7    C13
H4
  grid # 1       97    102     87     88     88     92     84
73
  grid # 2      103    112     95     95     96    101     92
118
  grid # 3      214    227    197    186    193    197    164
223
  grid # 4      393    413    326    321    328    351    300
224

  number of gridpoints:
     atom        C8    C11     H7     C9     H8    C10    C12
C2
  grid # 1       89     87     73     87     73     89     86
93
  grid # 2       97     94    118     97    118     97     96
100
  grid # 3      184    184    223    184    222    185    195
197
  grid # 4      328    336    226    331    224    332    319
344

  number of gridpoints:
     atom       H13    C14    H15    C15    C16    C17    C18
C19
  grid # 1       69     93     69     89     88     89     89
89
  grid # 2      109    100    109     97     96     96     97
97
  grid # 3      211    195    214    184    184    184    186
182
  grid # 4      216    340    216    328    328    330    331
328

  number of gridpoints:
     atom       H16    H17    H18    H19    H20     C1     H5
H6
  grid # 1       72     72     73     73     73     82     72
69
  grid # 2      112    115    118    118    118     90    112
102
  grid # 3      213    216    223    222    223    166    217
205
  grid # 4      214    215    224    222    223    294    219
207
```

```
  number of gridpoints:
     atom        C3    C26    C27    C28    C29     H1     H2
H3
  grid # 1       89     87     87     89     89     72     70
73
  grid # 2       97     97     96     97     97    115    111
118
  grid # 3      185    181    183    184    184    218    213
222
  grid # 4      329    328    330    328    328    217    210
224

  number of gridpoints:
     atom        H9    H10    H25  total
  grid # 1       73     73     72   3523
  grid # 2      118    118    115   4494
  grid # 3      223    223    219   8635
  grid # 4      224    224    222  12295

  end of program grid


  start of program rwr
  end of program rwr


  start of program scf

       i  u  d  i  g
       t  p  i  c  r                                 RMS    maximum
       e  d  i  u  i                      energy  density   DIIS
       r  t  s  t  d     total energy     change  change    error

etot   1  N  N  2  U  -990.37561875819            2.2E-04  5.2E-03
etot   2  Y  Y  6  M  -990.38629314976  1.1E-02  8.4E-05  2.1E-03
etot   3  N  Y  2  U  -990.38722199985  9.3E-04  3.1E-05  7.2E-04
etot   4  Y  Y  6  M  -990.38736835388  1.5E-04  1.3E-05  3.4E-04
etot   5  Y  Y  6  M  -990.38740323781  3.5E-05  5.6E-06  1.3E-04
etot   6  Y  Y  6  M  -990.38740916528  5.9E-06  2.4E-06  4.7E-05
etot   7  Y  N  6  M  -990.38741265890  3.5E-06  0.0E+00  0.0E+00


 Energy components, in hartrees:
   (A)  Nuclear repulsion............  1960.02558935013
   (E)  Total one-electron terms..... -5238.29315543660
   (I)  Total two-electron terms.....  2287.88015342757
   (L)  Electronic energy............ -2950.41300200903  (E+I)
   (N)  Total energy.................  -990.38741265890  (A+L)

 SCFE: SCF energy: HF    -990.38741265890 hartrees   iterations:
7
```

```
HOMO energy:    -0.26625
LUMO energy:     0.09232

 Orbital energies:
 -15.60805  -15.56700  -11.29787  -11.29337  -11.27638  -11.25511
 -11.25341  -11.25179  -11.24692  -11.24146  -11.24113  -11.24000
 -11.23941  -11.23867  -11.23797  -11.23592  -11.23504  -11.23469
 -11.23278  -11.23229  -11.22657  -11.22384  -11.22377  -11.21820
 -11.21692   -1.31817   -1.24994   -1.16238   -1.15889   -1.13461
  -1.07262   -1.06754   -1.03658   -1.02296   -1.01802   -1.01474
  -0.96749   -0.95529   -0.93114   -0.85928   -0.83758   -0.83378
  -0.83067   -0.80421   -0.79354   -0.76882   -0.73077   -0.71744
  -0.69958   -0.69803   -0.66500   -0.65657   -0.64316   -0.63792
  -0.63536   -0.62308   -0.61992   -0.61392   -0.59697   -0.59327
  -0.58930   -0.58645   -0.57884   -0.55677   -0.55072   -0.54533
  -0.54289   -0.51775   -0.50819   -0.50301   -0.50024   -0.49594
  -0.49263   -0.49143   -0.48732   -0.48017   -0.42932   -0.40604
  -0.36912   -0.34308   -0.34234   -0.33503   -0.33025   -0.28019
  -0.26625    0.09232    0.12603    0.13625    0.13684    0.14868
   0.17722    0.20851    0.22157    0.23301    0.24218

 end of program scf


 start of program der1a
 end of program der1a


 start of program rwr
 end of program rwr


 start of program der1b

 forces (hartrees/bohr) : total
```

atom	label	x	y	z
1	N1	-9.539155E-03	2.745592E-03	1.044822E-02
2	N2	-2.673465E-03	1.727028E-03	2.906341E-03
3	C4	-1.885556E-04	1.245403E-02	3.193744E-03
4	C5	2.271285E-03	6.211561E-03	1.516907E-03
5	C6	-6.464198E-03	7.479387E-03	7.554108E-03
6	C7	8.793345E-03	-1.029557E-02	-9.843759E-03
7	C13	-5.471678E-04	-7.069051E-03	-5.718790E-03
8	H4	1.852086E-03	3.212933E-03	-7.082379E-04
9	C8	4.338852E-03	4.033060E-03	-3.262937E-03
10	C11	1.606909E-03	-1.006096E-02	-6.249112E-03
11	H7	9.730113E-04	-2.564706E-03	-1.338833E-03
12	C9	-5.458253E-04	-4.327700E-03	-5.240229E-04

```
 13    H8       2.968983E-03   5.464179E-04  -2.555816E-03
 14    C10      4.804096E-03   2.217867E-04  -3.885245E-03
 15    C12     -7.171547E-03  -3.974109E-03   7.707933E-03
 16    C2       9.332330E-04   2.740879E-04  -3.181994E-03
 17    H13     -2.015010E-03  -1.526317E-03  -1.690651E-03
 18    C14      3.524810E-03  -3.916218E-04  -2.663166E-03
 19    H15      1.889224E-03   3.787146E-03   1.319866E-03
 20    C15     -1.027612E-03   6.505881E-05   3.781739E-04
 21    C16     -4.116869E-03  -8.483180E-04  -5.317073E-03
 22    C17     -1.277121E-04   3.725580E-03  -1.022516E-02
 23    C18      8.848389E-03   3.067855E-03   9.831513E-03
 24    C19      3.253650E-03  -3.767333E-04   8.365422E-03
 25    H16      4.090188E-03   1.430527E-03  -2.015014E-04
 26    H17     -2.307131E-03  -1.447538E-03   4.755238E-04
 27    H18     -8.359663E-03  -3.799457E-03  -4.288966E-04
 28    H19      2.299917E-03   9.846778E-04  -2.783170E-04
 29    H20     -1.387235E-03  -1.403497E-03   2.321291E-03
 30    C1       3.683293E-03   3.676776E-03  -8.195002E-03
 31    H5      -9.851720E-03  -8.517076E-04  -2.436073E-03
 32    H6       1.473065E-03  -4.295533E-03   1.252041E-02
 33    C3       3.914041E-04  -3.266543E-04   4.963776E-04
 34    C26     -2.384326E-03  -3.429782E-03  -4.918934E-03
 35    C27     -1.975575E-03  -5.180976E-04   5.429357E-03
 36    C28      1.047866E-03   1.965373E-03   9.943780E-04
 37    C29      2.585439E-03   2.168460E-03  -3.629454E-03
 38    H1       4.052696E-04   7.061260E-05   1.388971E-03
 39    H2       5.731526E-04   1.449645E-03   2.603645E-05
 40    H3       3.735787E-05  -1.572279E-04   8.376755E-04
 41    H9      -9.939832E-04  -7.438282E-04  -2.855378E-04
 42    H10     -1.057111E-03  -6.022944E-04   1.457865E-03
 43    H25      1.116333E-03  -1.534056E-03  -1.866734E-03
-----------   -------------  -------------  -------------
   total        1.027296E-03   7.528330E-04  -2.351347E-04

 end of program der1b


 start of program geopt   2

 geometry optimization step  2
 reading input hessian of dimension   129
  in five columns format


 Level shifts adjusted to satisfy step-size constraints
  Step size:    0.3001262
  Cos(theta):   0.4522028

  Final level shift:  -7.1431608E-03

 energy change:          -2.1167E-02 .  (  5.0000E-05 )
 gradient maximum:        2.0310E-02 .  (  4.5000E-04 )
```

```
  gradient rms:            3.9611E-03 .  (  3.0000E-04 )
  step size:   0.29998  trust radius:   0.30000
  displacement maximum:    1.2227E-01 .  (  1.8000E-03 )
  displacement rms:        2.3569E-02 .  (  1.2000E-03 )
  predicted energy change:    -3.7426E-03    geom step:   2.9998E-
01    full step:   2.9998E-01
  molecular structure not yet converged...

  center of mass moved by:
     x:    -9.2535E-03     y:     3.1123E-03     z:     1.4212E-03

  new geometry:
                                angstroms
  atom            x                y                 z
  N1       -0.2322091003      0.4425447477      1.1722376588
  N2       -1.0170271355     -2.2784648229      1.1730995628
  C4       -1.2206159278     -0.0226657246      1.9837450839
  C5       -0.0448348209     -1.9052512670      0.4751983779
  C6       -1.6507777180     -1.3371163143      2.0094539253
  C7       -1.7024716670      0.9919018866      2.7899381630
  C13       0.6283020713     -2.9170533017     -0.4248611566
  H4       -3.0346295842     -2.6472027368      2.9359624232
  C8       -2.7497248744      0.6679486212      3.6759761067
  C11      -0.9219400908      2.1551243638      2.4291375123
  H7       -3.1822925194      1.4081076944      4.3208527754
  C9       -3.2122659889     -0.6338983252      3.7021230748
  H8       -4.0092368660     -0.8922566392      4.3712351952
  C10      -2.6664125428     -1.6434906978      2.8798451206
  C12      -0.0462186113      1.7852092098      1.4609796706
  C2        0.9639603762      2.6172556086      0.7603660644
  H13       1.7049014864     -2.8504962231     -0.3042157835
  C14       0.2872845063     -2.7186247711     -1.9011082558
  H15       0.3111604572     -3.8957135626     -0.0910075502
  C15      -0.3637160763     -2.3879002288     -4.5957973831
  C16       1.2521389896     -2.2939910017     -2.8167452985
  C17      -1.0099224983     -2.9730715129     -2.3497803317
  C18      -1.3345315137     -2.8115555909     -3.6881685682
  C19       0.9281516443     -2.1274223633     -4.1580106912
  H16       2.2689730521     -2.0919467686     -2.4900654971
  H17      -1.7651731246     -3.3056021719     -1.6519350479
  H18      -2.3449269541     -3.0307603123     -4.0214737620
  H19       1.6928080246     -1.7993395561     -4.8496324883
  H20      -0.6146895035     -2.2677049661     -5.6318950361
  C1        0.5593460744     -0.4968540721      0.4025037161
  H5        1.5835558214     -0.5417582018      0.7564692615
  H6        0.6038790034     -0.2150061772     -0.6400473898
  C3        2.8215489000      4.2490688071     -0.5283409458
  C26       1.8492457635      3.4064361869      1.4997536269
  C27       1.0238745658      2.6583201916     -0.6349113427
  C28       1.9515451393      3.4616543589     -1.2728168602
  C29       2.7672368925      4.2206654051      0.8601900607
  H1        1.8310545892      3.3653506796      2.5769048131
```

```
  H2        0.3330469792      2.0787631427     -1.2253750449
  H3        1.9815361995      3.4802193197     -2.3500591580
  H9        3.4413282681      4.8236548923      1.4492434236
  H10       3.5316247441      4.8745490259     -1.0248311658
  H25      -1.0230567326      3.1472274108      2.8145615026

  nuclear repulsion energy.......  1967.210994655 hartrees
  ------------------------------------------------
 /  end of geometry optimization iteration   2   /
 ------------------------------------------------

  end of program geopt


  start of program onee
  smallest eigenvalue of S:    2.843E-04
  number of canonical orbitals.....          461
  end of program onee


  start of program probe
  end of program probe


  start of program grid

  number of gridpoints:
     atom        N1     N2      C4      C5      C6      C7     C13
H4
  grid # 1       97    102      84      88      87      89      84
73
  grid # 2      104    112      96      95      97      97      92
118
  grid # 3      214    227     196     186     189     198     163
223
  grid # 4      391    413     320     320     324     348     300
224

  number of gridpoints:
     atom        C8    C11      H7      C9      H8     C10     C12
C2
  grid # 1       89     87      73      87      73      89      86
92
  grid # 2       97     94     118      97     118      97      94
100
  grid # 3      184    185     223     186     222     184     194
196
  grid # 4      330    331     226     331     223     327     316
343

  number of gridpoints:
```

```
     atom       H13    C14    H15    C15    C16    C17    C18
C19
  grid # 1       69     93     69     89     88     88     89
89
  grid # 2      109    101    109     97     96     96     97
96
  grid # 3      211    195    214    184    185    184    185
184
  grid # 4      214    342    212    329    329    328    328
328

  number of gridpoints:
     atom       H16    H17    H18    H19    H20     C1     H5
H6
  grid # 1       72     73     73     73     73     82     70
69
  grid # 2      113    115    118    118    118     88    109
104
  grid # 3      214    215    223    222    224    165    217
207
  grid # 4      221    215    224    222    224    290    217
203

  number of gridpoints:
     atom        C3    C26    C27    C28    C29     H1     H2
H3
  grid # 1       89     89     88     89     89     72     71
73
  grid # 2       97     95     96     97     97    115    114
118
  grid # 3      185    182    185    184    184    218    214
223
  grid # 4      328    328    330    328    329    218    210
223

  number of gridpoints:
     atom        H9    H10    H25  total
  grid # 1       73     73     72   3517
  grid # 2      118    118    115   4490
  grid # 3      223    223    220   8640
  grid # 4      223    224    224  12258

  end of program grid


  start of program rwr
 recomputing RwR matrix   1    grid:  4
  end of program rwr


  start of program scf
```

```
       i  u  d  i  g
       t  p  i  c  r                                        RMS    maximum
       e  d  i  u  i                           energy  density    DIIS
       r  t  s  t  d     total energy          change  change     error

etot   1  N  N  2  U  -990.33510685770                 3.7E-04  3.1E-02
etot   2  Y  Y  6  M  -990.38314472816  4.8E-02  1.8E-04  1.2E-02
etot   3  N  Y  2  U  -990.38935537066  6.2E-03  5.3E-05  3.1E-03
etot   4  Y  Y  6  M  -990.38969679674  3.4E-04  2.3E-05  1.1E-03
etot   5  Y  Y  6  M  -990.38977611385  7.9E-05  7.6E-06  2.8E-04
etot   6  N  Y  2  U  -990.38979109239  1.5E-05  3.0E-06  6.9E-05
etot   7  Y  N  6  M  -990.38979365506  2.6E-06  0.0E+00  0.0E+00


 Energy components, in hartrees:
   (A)  Nuclear repulsion............  1967.21099465467
   (E)  Total one-electron terms..... -5252.71557057016
   (I)  Total two-electron terms.....  2295.11478226043
   (L)  Electronic energy............ -2957.60078830974  (E+I)
   (N)  Total energy.................  -990.38979365506  (A+L)

 SCFE: SCF energy: HF    -990.38979365506 hartrees   iterations:
7


 HOMO energy:    -0.26875
 LUMO energy:     0.09679

  Orbital energies:
  -15.60440  -15.56408  -11.29484  -11.28605  -11.27171  -11.25310
  -11.25060  -11.24821  -11.24599  -11.24090  -11.23981  -11.23932
  -11.23928  -11.23863  -11.23847  -11.23780  -11.23743  -11.23721
  -11.23472  -11.23260  -11.22506  -11.21958  -11.21917  -11.21594
  -11.21230   -1.32371   -1.25138   -1.16194   -1.15811   -1.13746
   -1.07380   -1.06529   -1.03784   -1.02261   -1.01706   -1.01675
   -0.97106   -0.95358   -0.93345   -0.86016   -0.83584   -0.83204
   -0.82722   -0.80690   -0.79592   -0.76979   -0.73260   -0.71689
   -0.70107   -0.69646   -0.66553   -0.65782   -0.64254   -0.63657
   -0.63323   -0.62328   -0.62039   -0.61546   -0.59597   -0.59273
   -0.59071   -0.58733   -0.57653   -0.55793   -0.55254   -0.54806
   -0.54230   -0.51743   -0.50677   -0.50276   -0.50055   -0.49573
   -0.49322   -0.49115   -0.48789   -0.48019   -0.42914   -0.40421
   -0.36715   -0.34259   -0.34204   -0.33775   -0.32820   -0.28241
   -0.26875    0.09679    0.12767    0.13691    0.13716    0.14724
    0.17920    0.20956    0.22641    0.23420    0.24136

  end of program scf


  start of program der1a
  end of program der1a
```

```
 start of program rwr
recomputing RwR matrix   1    grid:  4
 end of program rwr


 start of program der1b

 forces (hartrees/bohr) : total

atom  label          x             y             z
----  -----   -------------  -------------  -------------
  1    N1      1.305964E-03  -1.677270E-03  -2.594790E-03
  2    N2     -7.405171E-03  -1.586511E-03   6.320733E-03
  3    C4      3.028731E-03   1.790473E-03  -3.798228E-04
  4    C5      7.226851E-03   4.524737E-03  -7.098855E-03
  5    C6      2.476199E-04  -3.135427E-03   2.297075E-04
  6    C7     -2.330886E-03   2.478329E-03   3.273788E-04
  7    C13     2.227097E-04   1.311746E-03  -4.798910E-03
  8    H4     -1.002454E-03  -3.214195E-03  -8.533394E-05
  9    C8      2.159221E-03  -4.853058E-03  -3.527892E-03
 10    C11    -3.302127E-03  -1.233147E-03   3.282272E-03
 11    H7     -7.362198E-04   1.749372E-03   1.319340E-03
 12    C9      3.320533E-03   4.276770E-03  -1.934309E-03
 13    H8     -1.912353E-03  -7.144657E-04   1.822387E-03
 14    C10     8.315593E-04   4.548646E-03   7.271826E-04
 15    C12     4.548962E-03  -1.450949E-03  -2.371630E-03
 16    C2     -9.938152E-04   7.535157E-04   1.267906E-03
 17    H13     1.386542E-03  -4.258552E-04   9.927676E-04
 18    C14     2.299989E-03  -7.682804E-05   1.238775E-03
 19    H15     1.984307E-04  -8.884950E-04  -8.118002E-04
 20    C15    -6.451323E-04  -1.027048E-03   4.774511E-03
 21    C16    -2.632256E-04  -3.149773E-04   3.356515E-03
 22    C17     1.008954E-03   6.183636E-05  -1.888416E-03
 23    C18     1.484312E-04  -5.973317E-04  -2.098115E-03
 24    C19    -1.156244E-03  -3.591191E-04   4.042049E-04
 25    H16    -7.120642E-03  -2.020923E-03  -6.701640E-04
 26    H17     3.236541E-03   1.487269E-03  -1.213183E-03
 27    H18     7.301887E-03   2.526935E-03   1.127166E-03
 28    H19    -4.266701E-03  -1.422013E-03   1.600621E-03
 29    H20    -5.797366E-04   3.861398E-04  -2.246165E-03
 30    C1     -5.155529E-03  -8.921110E-04   1.159786E-03
 31    H5      2.282436E-03   2.852896E-04   2.405521E-03
 32    H6     -2.782347E-04   1.257850E-03  -1.502622E-03
 33    C3     -3.641709E-03  -3.629131E-03   3.069671E-03
 34    C26    -2.578335E-03  -3.574081E-03  -3.583493E-04
 35    C27    -8.629869E-04  -1.384270E-03   3.235411E-03
 36    C28     1.635804E-03   2.327281E-03   9.648920E-04
 37    C29     8.904502E-04   7.644047E-04  -3.194343E-03
 38    H1     -1.706450E-03  -2.318215E-04  -2.241600E-03
 39    H2      4.690082E-04   6.810360E-04   2.172640E-03
 40    H3      8.078606E-04   8.518771E-04   1.530140E-03
```

```
  41    H9     -1.100102E-03  -1.113211E-03  -2.495881E-03
  42    H10     3.825774E-03   3.254866E-03  -2.412596E-03
  43    H25    -2.808875E-04   1.593320E-03   4.916000E-04
 -----------   -------------  -------------  -------------
    total       1.065317E-03   1.089457E-03  -1.036505E-04

  end of program der1b


  start of program geopt   3

  geometry optimization step  3
  reading input hessian of dimension   129
   in five columns format
  reading input hessian of dimension   129
   in five columns format


  Level shifts adjusted to satisfy step-size constraints
   Step size:    0.3000236
   Cos(theta):   0.4953472

   Final level shift:  -1.6630388E-02

  energy change:          -2.3810E-03 .  (  5.0000E-05 )
  gradient maximum:        9.9076E-03 .  (  4.5000E-04 )
  gradient rms:            2.3236E-03 .  (  3.0000E-04 )
  step size:   0.29975  trust radius:   0.30000
  displacement maximum:    1.0837E-01 .  (  1.8000E-03 )
  displacement rms:        2.3551E-02 .  (  1.2000E-03 )
  predicted energy change:    -2.9446E-03    geom step:   2.9975E-
01    full step:   2.9975E-01
  molecular structure not yet converged...

  center of mass moved by:
     x:     4.5379E-03     y:     1.6225E-03     z:    -1.0573E-02

  new geometry:
                              angstroms
  atom           x                  y                  z
  N1       -0.2064093151      0.4564334872      1.1449569640
  N2       -0.9777143120     -2.2675882391      1.1401282031
  C4       -1.2067896365     -0.0074822023      1.9347114372
  C5        0.0100178696     -1.8812418506      0.4407932188
  C6       -1.6301115836     -1.3290633316      1.9647297688
  C7       -1.7180530931      1.0023503931      2.7134818099
  C13       0.7013842690     -2.8816737324     -0.4740592873
  H4       -3.0216327063     -2.6539904306      2.8557401227
  C8       -2.7796853502      0.6766421222      3.5699349035
  C11      -0.9469746435      2.1651389329      2.3678165600
  H7       -3.2396438255      1.4178307697      4.2013122414
  C9       -3.2317902692     -0.6316840549      3.6015379272
```

```
 H8       -4.0475645815     -0.8929676898      4.2526147254
 C10      -2.6592479127     -1.6425581119      2.8070396413
 C12      -0.0282154727      1.7951337785      1.4350689770
 C2        0.9934644979      2.6280168532      0.7795619441
 H13       1.7730430366     -2.7575478083     -0.4156955215
 C14       0.2865859920     -2.7249268767     -1.9051731340
 H15       0.4713121897     -3.8629015133     -0.0943843922
 C15      -0.5615655542     -2.4619627032     -4.5190042806
 C16       1.1125815133     -2.1862381413     -2.8583626252
 C17      -0.9661916103     -3.1291782413     -2.2777042664
 C18      -1.3890096252     -3.0026868085     -3.5700862667
 C19       0.6893320833     -2.0524259860     -4.1584209800
 H16       2.0979129012     -1.8591107039     -2.5945051992
 H17      -1.6160289056     -3.5619217481     -1.5421987308
 H18      -2.3733434532     -3.3581172536     -3.8357831773
 H19       1.3434543159     -1.6264018218     -4.8889259482
 H20      -0.9021702747     -2.3804723563     -5.5318037396
 C1        0.5974414175     -0.4754948750      0.3889408228
 H5        1.6054220101     -0.5235796038      0.7857568676
 H6        0.6751609178     -0.1866596432     -0.6452941877
 C3        2.9244707837      4.2594570176     -0.3923566804
 C26       1.7907979932      3.4485799055      1.5329596121
 C27       1.1713262529      2.6405355796     -0.5729922605
 C28       2.1366910125      3.4439199169     -1.1538018568
 C29       2.7452329284      4.2656384654      0.9532276351
 H1        1.6745334301      3.4364257238      2.5857229416
 H2        0.5529801450      2.0327292614     -1.1904461656
 H3        2.2687279040      3.4308993892     -2.2062196320
 H9        3.3448720185      4.8980738147      1.5650329855
 H10       3.6696029435      4.8876836769     -0.8403675527
 H25      -1.0676334116      3.1643232144      2.7358050805

 nuclear repulsion energy.......  1978.365603056 hartrees
 -------------------------------------------------
/  end of geometry optimization iteration   3   /
-------------------------------------------------

 end of program geopt


 start of program onee
 smallest eigenvalue of S:    2.790E-04
 number of canonical orbitals.....          461
 end of program onee


 start of program probe
 end of program probe


 start of program grid
```

```
  number of gridpoints:
     atom         N1     N2      C4      C5      C6      C7     C13
H4
  grid # 1        96    102      86      88      90      88      84
73
  grid # 2       104    112      95      95      99      96      92
118
  grid # 3       214    227     194     188     192     198     164
224
  grid # 4       389    414     321     321     322     344     298
223

  number of gridpoints:
     atom         C8    C11      H7      C9      H8     C10     C12
C2
  grid # 1        89     87      73      87      73      89      86
88
  grid # 2        97     94     118      97     118      97      94
99
  grid # 3       185    184     223     185     222     184     195
191
  grid # 4       331    331     226     330     223     326     320
339

  number of gridpoints:
     atom        H13    C14     H15     C15     C16     C17     C18
C19
  grid # 1        69     93      69      87      86      87      87
87
  grid # 2       109    101     109      97      96      96      97
97
  grid # 3       211    191     213     182     182     181     182
182
  grid # 4       209    339     212     325     327     327     328
327

  number of gridpoints:
     atom        H16    H17     H18     H19     H20      C1      H5
H6
  grid # 1        72     72      73      73      73      82      70
69
  grid # 2       113    114     118     118     118      87     108
103
  grid # 3       213    215     222     221     223     163     217
204
  grid # 4       213    214     223     222     224     290     217
202

  number of gridpoints:
     atom         C3    C26     C27     C28     C29      H1      H2
H3
```

```
  grid # 1       89     87     86     87     87     72     70
73
  grid # 2       97     96     96     97     97    115    113
118
  grid # 3      182    181    182    182    183    217    213
222
  grid # 4      328    326    327    327    324    216    210
222

  number of gridpoints:
     atom        H9    H10    H25  total
  grid # 1       73     73     72   3497
  grid # 2      118    118    115   4486
  grid # 3      222    222    219   8597
  grid # 4      223    223    222  12205

  end of program grid


  start of program rwr
  end of program rwr


  start of program scf

       i  u  d  i  g
       t  p  i  c  r                                    RMS    maximum
       e  d  i  u  i                        energy  density   DIIS
       r  t  s  t  d     total energy     change  change    error

etot   1  N  N  2  U  -990.32090183004            4.0E-04  2.7E-02
etot   2  Y  Y  6  M  -990.37602444074  5.5E-02  1.9E-04  1.1E-02
etot   3  N  Y  2  U  -990.38279174353  6.8E-03  5.6E-05  2.9E-03
etot   4  Y  Y  6  M  -990.38330673990  5.1E-04  2.8E-05  1.2E-03
etot   5  Y  Y  6  M  -990.38339751676  9.1E-05  7.9E-06  2.7E-04
etot   6  N  Y  2  U  -990.38336351948 -3.4E-05  3.5E-06  8.1E-05
etot   7  Y  N  6  M  -990.38336784090  4.3E-06  0.0E+00  0.0E+00


 Energy components, in hartrees:
   (A)  Nuclear repulsion............  1978.36560305571
   (E)  Total one-electron terms..... -5274.81755689824
   (I)  Total two-electron terms.....  2306.06858600163
   (L)  Electronic energy............ -2968.74897089661  (E+I)
   (N)  Total energy.................  -990.38336784090  (A+L)

 SCFE: SCF energy: HF    -990.38336784090 hartrees   iterations:
7


 HOMO energy:    -0.26814
 LUMO energy:     0.09639
```

```
Orbital energies:
-15.60547  -15.56550  -11.29770  -11.28619  -11.27078  -11.25022
-11.24528  -11.24450  -11.23825  -11.23745  -11.23449  -11.23301
-11.23221  -11.23109  -11.23069  -11.23034  -11.22948  -11.22838
-11.22673  -11.22459  -11.22253  -11.21876  -11.21686  -11.21593
-11.21192   -1.32742   -1.24676   -1.17463   -1.17229   -1.14078
 -1.07809   -1.07260   -1.04756   -1.02329   -1.02291   -1.02144
 -0.97578   -0.95637   -0.93529   -0.86214   -0.84435   -0.83597
 -0.83375   -0.80916   -0.79677   -0.77365   -0.73422   -0.72183
 -0.70571   -0.70299   -0.66886   -0.65951   -0.64726   -0.64126
 -0.63446   -0.62424   -0.62327   -0.61823   -0.59835   -0.59393
 -0.59044   -0.58890   -0.58373   -0.56063   -0.55582   -0.54996
 -0.54261   -0.52147   -0.51054   -0.50427   -0.50252   -0.49989
 -0.49515   -0.49181   -0.48895   -0.48236   -0.42870   -0.40579
 -0.37068   -0.34887   -0.34027   -0.33992   -0.33265   -0.28268
 -0.26814    0.09639    0.13129    0.13834    0.14272    0.15301
  0.18158    0.21027    0.22587    0.23576    0.24199

end of program scf


start of program der1a
end of program der1a


start of program rwr
end of program rwr


start of program der1b

forces (hartrees/bohr) : total

atom  label         x             y             z
----  -----   ------------- ------------- -------------
  1    N1      2.088108E-03 -1.816608E-03 -3.641703E-03
  2    N2      1.359145E-02  2.603072E-03 -9.392358E-03
  3    C4      4.660404E-04 -1.020443E-02 -2.510234E-03
  4    C5     -1.216198E-02 -6.585574E-03  6.161446E-03
  5    C6      7.087680E-03 -4.398158E-04 -6.447246E-03
  6    C7     -3.899426E-03  5.933573E-03  2.324081E-03
  7    C13     3.821605E-03  5.890252E-03  1.357044E-02
  8    H4      2.973135E-04  4.132462E-04  7.230307E-05
  9    C8     -6.397915E-03 -7.377681E-03  4.298384E-03
 10    C11     3.207322E-03  5.420828E-03  1.334689E-03
 11    H7      6.085201E-04  2.998135E-05 -7.440425E-04
 12    C9     -3.822426E-04  8.286041E-03  2.243006E-03
 13    H8      2.340008E-04  7.691603E-04  4.906764E-04
 14    C10    -1.938760E-03 -6.468270E-04  1.244062E-03
 15    C12    -6.102382E-03 -1.064691E-03  1.815286E-03
 16    C2     -8.753000E-03 -6.468478E-03  1.178688E-02
```

```
 17    H13      5.162556E-03   1.641355E-03   3.634586E-03
 18    C14      3.407200E-03  -1.591991E-03   8.898751E-03
 19    H15     -2.336800E-03  -4.255029E-03  -1.094404E-03
 20    C15     -8.884359E-03  -7.898080E-04  -1.509099E-02
 21    C16      1.333100E-02   6.137570E-03   1.627549E-03
 22    C17     -1.655284E-02  -9.413400E-03   8.281097E-03
 23    C18     -2.099264E-02  -9.634396E-03  -4.550000E-03
 24    C19      1.119810E-02   6.856261E-03  -8.067226E-03
 25    H16      4.778835E-03   6.395915E-04   1.815445E-03
 26    H17     -2.269290E-03  -3.734078E-04   2.039251E-03
 27    H18      1.898389E-03   2.441004E-03  -7.736249E-04
 28    H19      4.019581E-03   1.995855E-03  -3.556696E-03
 29    H20      5.225823E-04   1.660109E-03  -3.684042E-03
 30    C1      -1.526528E-04  -1.293907E-03   5.029993E-03
 31    H5       3.804775E-03   3.806220E-04   3.734436E-04
 32    H6       1.802152E-04   1.858511E-03  -4.200778E-03
 33    C3       1.062426E-02   8.644818E-03  -1.207225E-02
 34    C26      1.147070E-02   1.162699E-02   1.033083E-02
 35    C27      4.957450E-03  -1.858617E-04  -2.536308E-02
 36    C28     -9.660113E-03  -1.041083E-02  -8.949314E-03
 37    C29     -5.181304E-03  -1.111879E-03   2.398876E-02
 38    H1      -3.259171E-04   1.476531E-03   1.312592E-02
 39    H2      -4.697105E-03  -3.791650E-03  -6.595315E-03
 40    H3      -5.870500E-04  -1.164633E-03  -1.251753E-02
 41    H9       4.371697E-03   4.237461E-03   6.763454E-03
 42    H10      1.379739E-03   7.800523E-04  -2.571094E-03
 43    H25     -3.342425E-04  -4.287707E-04   6.210274E-04
-----------   -------------  -------------  -------------
   total        8.991054E-04   6.732157E-04   4.943495E-05

 end of program der1b


 start of program geopt   4

 geometry optimization step  4
 reading input hessian of dimension   129
  in five columns format
 reading input hessian of dimension   129
  in five columns format
 ** restarting optimization from step    3 **


 Level shifts adjusted to satisfy step-size constraints
  Step size:    0.3000460
  Cos(theta):   0.3075896

  Final level shift:  -4.9937562E-03

 energy change:            6.4258E-03 .  (  5.0000E-05 )
 gradient maximum:         9.9076E-03 .  (  4.5000E-04 )
 gradient rms:             2.3236E-03 .  (  3.0000E-04 )
```

```
  step size:   0.29978  trust radius:   0.30000
  displacement maximum:    1.5805E-01 .  (  1.8000E-03 )
  displacement rms:        2.3553E-02 .  (  1.2000E-03 )
  predicted energy change:    -1.5876E-03    geom step:   2.9978E-
01    full step:   2.9978E-01
  molecular structure not yet converged...

  center of mass moved by:
     x:    -2.4980E-16     y:    -3.4694E-17     z:    -6.1062E-16

  new geometry:
                              angstroms
  atom            x                 y                  z
  N1       -0.2605037549      0.4125439612      1.1900034197
  N2       -1.0201882848     -2.3117451892      1.2286465763
  C4       -1.2291547382     -0.0501813353      2.0213210471
  C5       -0.0584840655     -1.9323738777      0.5045877913
  C6       -1.6433405541     -1.3707651498      2.0724302477
  C7       -1.7196322432      0.9707097685      2.8115399710
  C13       0.6123641543     -2.9395135162     -0.4045486213
  H4       -3.0004875650     -2.6843033639      3.0337672096
  C8       -2.7502677659      0.6466074665      3.7128944205
  C11      -0.9641692572      2.1368330189      2.4248916539
  H7       -3.1870956384      1.3938929758      4.3523390576
  C9       -3.1923609494     -0.6577264470      3.7717764284
  H8       -3.9791920871     -0.9171763497      4.4577173169
  C10      -2.6430824576     -1.6735110866      2.9607130298
  C12      -0.0894151548      1.7589272383      1.4540514090
  C2        0.8975931598      2.5804167511      0.7178403216
  H13       1.6900885460     -2.9175445528     -0.2351375024
  C14       0.3269913213     -2.6769735270     -1.8741990189
  H15       0.2546697460     -3.9228910664     -0.1143387386
  C15      -0.2180137009     -2.2191864228     -4.5767173828
  C16       1.3496559462     -2.4227648804     -2.7664884238
  C17      -0.9764471114     -2.6992027704     -2.3557447632
  C18      -1.2462393835     -2.4742543646     -3.6931624843
  C19       1.0776231861     -2.1937356607     -4.1071135875
  H16       2.3658535888     -2.4078532060     -2.4199820995
  H17      -1.7766859001     -2.8933381124     -1.6738564557
  H18      -2.2487902734     -2.4928964715     -4.0444386495
  H19       1.8875121812     -1.9993966839     -4.7814773829
  H20      -0.4271057620     -2.0495195336     -5.6159740449
  C1        0.5363502956     -0.5262371704      0.4297996206
  H5        1.5569005697     -0.5698600523      0.8077454585
  H6        0.5969268301     -0.2400532601     -0.6132951547
  C3        2.7366658910      4.1635728363     -0.6570139302
  C26       1.8494198086      3.3298911691      1.4147477126
  C27       0.8738252674      2.6383115327     -0.6774631325
  C28       1.7919425574      3.4195085202     -1.3586689873
  C29       2.7597734096      4.1195096692      0.7311384976
  H1        1.8863612505      3.2798815381      2.4949515492
  H2        0.1256541849      2.0873284863     -1.2299565889
```

```
  H3        1.7610423948      3.4526209625     -2.4364084657
  H9        3.4896654651      4.6919589348      1.2854978074
  H10       3.4478987066      4.7719686917     -1.1860693938
  H25      -1.0742926529      3.1373751310      2.7923517727

  nuclear repulsion energy.......  1974.668059347 hartrees
  -------------------------------------------------
 /  end of geometry optimization iteration   4   /
 -------------------------------------------------

  end of program geopt


  start of program onee
  smallest eigenvalue of S:    2.817E-04
  number of canonical orbitals.....          461
  end of program onee


  start of program probe
  end of program probe


  start of program grid

  number of gridpoints:
     atom        N1     N2     C4     C5     C6     C7    C13
H4
  grid # 1       97    102     87     88     90     88     84
73
  grid # 2      104    112     95     95     99     98     92
118
  grid # 3      214    227    195    186    191    200    164
224
  grid # 4      389    414    320    320    324    347    299
224

  number of gridpoints:
     atom        C8    C11     H7     C9     H8    C10    C12
C2
  grid # 1       89     86     73     87     73     89     84
92
  grid # 2       97     94    118     97    118     97     95
100
  grid # 3      184    185    223    184    222    184    195
196
  grid # 4      330    330    226    331    223    327    321
343

  number of gridpoints:
     atom       H13    C14    H15    C15    C16    C17    C18
C19
```

```
  grid # 1       69     91     69     89     88     86     89
87
  grid # 2      110    100    110     97     96     96     97
96
  grid # 3      210    196    218    182    183    183    183
182
  grid # 4      215    342    217    327    327    327    327
327

  number of gridpoints:
     atom       H16    H17    H18    H19    H20     C1     H5
H6
  grid # 1       73     73     73     73     73     82     70
69
  grid # 2      113    115    118    118    118     88    109
105
  grid # 3      214    216    222    221    223    165    217
208
  grid # 4      213    214    223    222    224    290    217
206

  number of gridpoints:
     atom        C3    C26    C27    C28    C29     H1     H2
H3
  grid # 1       89     89     88     89     89     73     71
73
  grid # 2       97     95     96     97     97    115    114
118
  grid # 3      185    182    185    184    184    218    216
223
  grid # 4      330    328    329    328    329    219    214
223

  number of gridpoints:
     atom        H9    H10    H25  total
  grid # 1       73     73     73   3516
  grid # 2      118    118    115   4495
  grid # 3      223    224    221   8642
  grid # 4      224    224    224  12258

  end of program grid


  start of program rwr
  end of program rwr


  start of program scf

       i  u  d  i  g
       t  p  i  c  r                               RMS    maximum
       e  d  i  u  i                   energy  density    DIIS
```

```
      r  t  s  t  d     total energy     change  change    error

etot   1  N  N  2  U  -990.14145563547           7.8E-04  3.9E-02
etot   2  Y  Y  6  M  -990.35861329482  2.2E-01  3.9E-04  1.6E-02
etot   3  N  Y  2  U  -990.38809039062  2.9E-02  1.2E-04  3.9E-03
etot   4  Y  Y  6  M  -990.38982038619  1.7E-03  4.1E-05  1.2E-03
etot   5  Y  Y  6  M  -990.39009100052  2.7E-04  1.6E-05  3.2E-04
etot   6  N  Y  2  U  -990.39013660923  4.6E-05  5.8E-06  1.0E-04
etot   7  Y  Y  6  M  -990.39013977366  3.2E-06  2.4E-06  3.6E-05
etot   8  Y  N  6  M  -990.39014043744  6.6E-07  0.0E+00  0.0E+00


 Energy components, in hartrees:
   (A)  Nuclear repulsion............  1974.66805934711
   (E)  Total one-electron terms..... -5267.59739614095
   (I)  Total two-electron terms.....  2302.53919635640
   (L)  Electronic energy............ -2965.05819978455  (E+I)
   (N)  Total energy.................  -990.39014043744  (A+L)

 SCFE: SCF energy: HF    -990.39014043744 hartrees   iterations:
8


 HOMO energy:    -0.26872
 LUMO energy:     0.09755

  Orbital energies:
  -15.60449  -15.56529  -11.29607  -11.28604  -11.27093  -11.25222
  -11.25039  -11.24527  -11.24459  -11.24123  -11.24070  -11.24044
  -11.24005  -11.23923  -11.23844  -11.23434  -11.23294  -11.23235
  -11.23098  -11.22932  -11.22452  -11.21957  -11.21815  -11.21574
  -11.21231   -1.32548   -1.24923   -1.16438   -1.16096   -1.13857
   -1.07363   -1.06865   -1.03835   -1.02173   -1.02055   -1.01749
   -0.97069   -0.95517   -0.93360   -0.86029   -0.83635   -0.83387
   -0.83017   -0.80766   -0.79576   -0.76982   -0.73217   -0.71750
   -0.70155   -0.70000   -0.66622   -0.65729   -0.64350   -0.63778
   -0.63371   -0.62436   -0.62014   -0.61563   -0.59577   -0.59197
   -0.59045   -0.58757   -0.58039   -0.55733   -0.55239   -0.54828
   -0.54513   -0.51672   -0.50646   -0.50304   -0.50031   -0.49689
   -0.49432   -0.49097   -0.48728   -0.48190   -0.42913   -0.40459
   -0.36595   -0.34214   -0.34166   -0.33817   -0.33118   -0.28287
   -0.26872    0.09755    0.12906    0.13686    0.13760    0.14839
    0.17922    0.20764    0.22771    0.23529    0.24258

  end of program scf


  start of program der1a
  end of program der1a


  start of program rwr
```

```
 end of program rwr


 start of program der1b

 forces (hartrees/bohr) : total

atom  label          x              y              z
----  -----    -------------  -------------  -------------
  1    N1       1.722647E-04  -1.371273E-03  -2.097232E-03
  2    N2       2.307619E-03   2.645434E-04  -1.429536E-03
  3    C4       9.233719E-04  -1.909727E-03  -3.397651E-04
  4    C5      -2.472205E-03  -1.364835E-03   1.074612E-03
  5    C6       9.104396E-04  -8.696098E-04  -8.667630E-04
  6    C7      -2.264358E-03   1.987665E-03   9.229630E-04
  7    C13      1.704094E-05  -1.512183E-03   1.932501E-03
  8    H4      -1.303564E-04  -3.601330E-04   6.584231E-05
  9    C8      -2.402673E-04  -8.558610E-04   6.577247E-04
 10    C11      1.082298E-03   1.304216E-03   5.319032E-04
 11    H7       2.368012E-04   2.173179E-04  -3.217904E-04
 12    C9       5.433283E-04   1.494368E-03  -2.250984E-04
 13    H8      -7.318402E-06   9.919423E-05   2.294528E-04
 14    C10     -3.416522E-04  -3.416849E-04   3.024806E-05
 15    C12      5.998397E-04   6.585509E-04   7.717878E-04
 16    C2       1.338325E-03   2.564232E-03   7.609119E-04
 17    H13     -2.445629E-03   3.019296E-04  -7.005633E-04
 18    C14     -1.006014E-03  -2.287806E-03   2.671755E-03
 19    H15      3.635537E-04   1.884244E-03  -9.943853E-04
 20    C15      2.027062E-03   2.500076E-04  -2.559217E-03
 21    C16      2.727088E-03   1.063025E-03  -3.560983E-03
 22    C17     -9.564304E-04  -6.952268E-05  -1.579709E-04
 23    C18      2.482024E-03  -4.635361E-04   6.616306E-03
 24    C19      5.817905E-03   2.160421E-04  -5.459483E-04
 25    H16      2.531985E-03   4.057878E-04   1.288178E-04
 26    H17     -4.824063E-03  -1.082739E-03   2.997523E-03
 27    H18     -1.047403E-02  -7.737787E-04  -2.242630E-03
 28    H19      2.831240E-03   6.714518E-04  -2.296848E-03
 29    H20      2.548070E-04   7.177312E-04  -1.647014E-03
 30    C1       4.326349E-04   1.117342E-03   1.134530E-03
 31    H5       3.099164E-04   3.869422E-04  -1.953429E-05
 32    H6      -4.484517E-05   5.228931E-04   6.438714E-04
 33    C3      -2.488043E-03  -2.181722E-03   6.024058E-04
 34    C26     -2.683586E-03  -2.958448E-03   1.442213E-03
 35    C27      2.666601E-04  -4.104175E-04   1.506358E-03
 36    C28      2.305602E-03   2.215407E-03   4.983410E-04
 37    C29      8.628620E-04   7.523044E-04  -1.843323E-03
 38    H1      -1.674844E-03  -2.410790E-04  -4.984929E-03
 39    H2       2.525584E-03   1.790540E-03   2.833707E-03
 40    H3       8.433839E-04   7.493248E-04   2.107534E-03
 41    H9      -2.068308E-03  -1.653712E-03  -2.587104E-03
 42    H10      8.221141E-05   1.717202E-04  -7.402267E-04
 43    H25     -1.315526E-04  -2.308952E-04  -4.026906E-05
```

```
 -----------   -------------  -------------  -------------
    total       5.423385E-04   8.678193E-04  -3.982191E-05

  end of program der1b


  start of program geopt   5

  geometry optimization step  5
  reading input hessian of dimension   129
   in five columns format
  reading input hessian of dimension   129
   in five columns format


  Level shifts adjusted to satisfy step-size constraints
   Step size:    0.3001034
   Cos(theta):   0.4746771

   Final level shift:  -1.8111015E-02

  energy change:          -3.4678E-04 .  (  5.0000E-05 )
  gradient maximum:        1.0656E-02 .  (  4.5000E-04 )
  gradient rms:            2.1734E-03 .  (  3.0000E-04 )
  step size:   0.29956  trust radius:   0.30000
  displacement maximum:    1.2495E-01 .  (  1.8000E-03 )
  displacement rms:        2.3536E-02 .  (  1.2000E-03 )
  predicted energy change:    -2.7815E-03    geom step:   2.9956E-
01    full step:   2.9956E-01
  molecular structure not yet converged...

  center of mass moved by:
     x:     8.1464E-03     y:     6.0566E-03     z:    -8.7285E-03

  new geometry:
                              angstroms
  atom            x                 y                 z
  N1      -0.2292024469      0.4427015271      1.1628880103
  N2      -0.9782568628     -2.2878207032      1.1779266582
  C4      -1.2094337002     -0.0303642055      1.9757531149
  C5      -0.0161249279     -1.9029247248      0.4674816553
  C6      -1.6194223744     -1.3523145475      2.0109279641
  C7      -1.7211646352      0.9868621882      2.7632723068
  C13      0.6544591552     -2.9143021590     -0.4408657883
  H4      -2.9999077625     -2.6718770241      2.9344446436
  C8      -2.7718199836      0.6572998838      3.6401084653
  C11     -0.9491404552      2.1543222696      2.4120276559
  H7      -3.2211900614      1.4029180697      4.2716980948
  C9      -3.2132989134     -0.6466971041      3.6786741679
  H8      -4.0163262814     -0.9105012423      4.3442732707
  C10     -2.6416683415     -1.6597368984      2.8743855662
  C12     -0.0562922792      1.7860587709      1.4560571753
```

```
 C2        0.9488951143      2.6311226377      0.7701830808
 H13       1.7281674582     -2.8468821058     -0.3270846920
 C14       0.2937960086     -2.7261589105     -1.9088007454
 H15       0.3445375745     -3.8922911028     -0.1088900635
 C15      -0.4402906415     -2.3952792758     -4.5794029238
 C16       1.2395116030     -2.3428910048     -2.8630371971
 C17      -1.0270889510     -2.9533277298     -2.3085099819
 C18      -1.3921717180     -2.7920309200     -3.6337066705
 C19       0.8788052648     -2.1725843519     -4.1956505663
 H16       2.2638244085     -2.1880678707     -2.5683467508
 H17      -1.7675239977     -3.2630084538     -1.5767242550
 H18      -2.4257099259     -2.9929594166     -3.9302355350
 H19       1.6217050002     -1.8773108746     -4.9256653884
 H20      -0.7280733175     -2.2675932286     -5.6111620858
 C1        0.5821138301     -0.4915229124      0.4118188533
 H5        1.5989978845     -0.5325329495      0.8024327801
 H6        0.6562234427     -0.2012124645     -0.6287278123
 C3        2.8551861345      4.2505727705     -0.4749461088
 C26       1.7771405951      3.4522098043      1.5201834275
 C27       1.0866007330      2.6404146539     -0.6152847579
 C28       2.0341472297      3.4391158849     -1.2329113044
 C29       2.7192766740      4.2587160999      0.9026633710
 H1        1.6846480152      3.4442277353      2.5858043452
 H2        0.4363216332      2.0366757013     -1.2116377193
 H3        2.1295952060      3.4333746931     -2.3030217836
 H9        3.3514260736      4.8860237979      1.4956835722
 H10       3.5921450310      4.8707133033     -0.9544874007
 H25      -1.0689547267      3.1499012113      2.7877233660

 nuclear repulsion energy.......  1967.972340116 hartrees
 -------------------------------------------------
/  end of geometry optimization iteration   5   /
-------------------------------------------------

 end of program geopt


 start of program onee
 smallest eigenvalue of S:    2.829E-04
 number of canonical orbitals.....          461
 end of program onee


 start of program probe
 end of program probe


 start of program grid

 number of gridpoints:
    atom         N1     N2     C4     C5     C6     C7    C13
H4
```

```
  grid # 1        95     102      87      88      90      90      84
73
  grid # 2       103     112      95      95      99      98      92
118
  grid # 3       212     227     195     188     190     199     163
224
  grid # 4       386     413     321     320     321     348     300
224

  number of gridpoints:
     atom         C8     C11      H7      C9      H8     C10     C12
C2
  grid # 1        89      87      73      87      73      89      86
92
  grid # 2        97      94     118      97     118      97      95
100
  grid # 3       184     185     223     184     222     184     195
195
  grid # 4       328     330     226     331     223     327     320
340

  number of gridpoints:
     atom        H13     C14     H15     C15     C16     C17     C18
C19
  grid # 1        69      93      69      89      88      88      89
89
  grid # 2       109     100     110      97      96      96      97
96
  grid # 3       211     198     213     185     185     184     185
184
  grid # 4       210     342     212     330     329     328     329
329

  number of gridpoints:
     atom        H16     H17     H18     H19     H20      C1      H5
H6
  grid # 1        72      72      73      73      73      82      70
69
  grid # 2       115     114     118     118     118      88     110
104
  grid # 3       214     215     223     222     224     163     217
206
  grid # 4       214     215     232     223     224     292     217
204

  number of gridpoints:
     atom         C3     C26     C27     C28     C29      H1      H2
H3
  grid # 1        89      87      88      89      89      72      70
73
  grid # 2        97      95      96      97      97     115     113
118
```

```
  grid # 3      184    182    184    184    185    217    213
222
  grid # 4      328    327    329    327    328    215    211
224

  number of gridpoints:
     atom        H9    H10    H25  total
  grid # 1       73     73     72   3518
  grid # 2      118    118    115   4493
  grid # 3      222    223    219   8634
  grid # 4      224    224    223  12248

  end of program grid


  start of program rwr
  end of program rwr


  start of program scf

       i  u  d  i  g
       t  p  i  c  r                                  RMS    maximum
       e  d  i  u  i                        energy  density   DIIS
       r  t  s  t  d     total energy    change  change    error

etot   1  N  N  2  U  -990.30892225319             4.4E-04  2.7E-02
etot   2  Y  Y  6  M  -990.37951017403  7.1E-02  2.2E-04  1.1E-02
etot   3  N  Y  2  U  -990.38894674829  9.4E-03  6.5E-05  2.8E-03
etot   4  Y  Y  6  M  -990.38948696771  5.4E-04  2.2E-05  8.5E-04
etot   5  Y  Y  6  M  -990.38957252266  8.6E-05  9.0E-06  2.1E-04
etot   6  N  Y  2  U  -990.38957720340  4.7E-06  3.0E-06  4.3E-05
etot   7  Y  N  6  M  -990.38957709405 -1.1E-07  0.0E+00  0.0E+00


 Energy components, in hartrees:
   (A)  Nuclear repulsion............  1967.97234011558
   (E)  Total one-electron terms..... -5254.19895519368
   (I)  Total two-electron terms.....  2295.83703798405
   (L)  Electronic energy............ -2958.36191720963  (E+I)
   (N)  Total energy.................  -990.38957709405  (A+L)

 SCFE: SCF energy: HF    -990.38957709405 hartrees   iterations:
7


 HOMO energy:    -0.26884
 LUMO energy:     0.09597

  Orbital energies:
  -15.60543  -15.56487  -11.29622  -11.28765  -11.27212  -11.25108
  -11.25053  -11.24792  -11.24652  -11.23892  -11.23891  -11.23883
```

```
 -11.23801  -11.23777  -11.23716  -11.23711  -11.23637  -11.23587
 -11.23553  -11.23260  -11.22496  -11.22078  -11.21925  -11.21651
 -11.21329   -1.32543   -1.25168   -1.16443   -1.15738   -1.13804
  -1.07535   -1.06529   -1.04001   -1.02194   -1.01727   -1.01663
  -0.97152   -0.95322   -0.93415   -0.85977   -0.83914   -0.83253
  -0.82686   -0.80677   -0.79549   -0.77050   -0.73321   -0.71759
  -0.70203   -0.69559   -0.66640   -0.65820   -0.64265   -0.63725
  -0.63362   -0.62320   -0.62133   -0.61564   -0.59719   -0.59253
  -0.59025   -0.58763   -0.57566   -0.55800   -0.55276   -0.54852
  -0.54404   -0.51815   -0.50817   -0.50261   -0.50009   -0.49525
  -0.49276   -0.49132   -0.48786   -0.48039   -0.42993   -0.40500
  -0.36914   -0.34440   -0.34069   -0.33787   -0.32708   -0.28199
  -0.26884    0.09597    0.12827    0.13658    0.13747    0.14798
   0.17910    0.21010    0.22715    0.23493    0.24011

 end of program scf


 start of program der1a
 end of program der1a


 start of program rwr
 end of program rwr


 start of program der1b

 forces (hartrees/bohr) : total

atom  label          x              y              z
----  -----   -------------  -------------  -------------
  1    N1      1.630734E-03   4.147837E-04  -2.328007E-04
  2    N2     -4.394325E-03  -1.352038E-03   2.472753E-03
  3    C4     -1.011245E-03   1.568492E-03   3.045752E-05
  4    C5      4.998187E-03   1.221166E-03  -4.075432E-03
  5    C6     -2.039398E-03  -4.311267E-04   1.780415E-03
  6    C7      6.101085E-05  -2.060345E-04   7.720866E-04
  7    C13     4.203179E-04   3.755701E-03  -3.272974E-03
  8    H4      5.925810E-05   2.078679E-04   8.546451E-05
  9    C8      1.534280E-03  -1.965932E-04  -1.082552E-03
 10    C11    -1.931759E-03  -9.908874E-04   6.215361E-04
 11    H7     -1.775814E-04   1.464387E-04   1.739668E-04
 12    C9      7.213503E-04  -1.263375E-03  -1.016812E-03
 13    H8      1.282530E-04  -1.587411E-04   7.876069E-06
 14    C10    -2.470540E-04   1.260490E-03   7.692993E-04
 15    C12     1.453405E-03   1.325890E-03  -3.933628E-03
 16    C2     -1.019141E-03  -2.748754E-03  -8.869362E-04
 17    H13     4.139215E-03   1.790964E-04   9.518011E-04
 18    C14    -1.890313E-03   5.889275E-04  -2.220388E-03
 19    H15    -5.789694E-04  -2.664568E-03   1.096563E-03
 20    C15    -1.086856E-03  -8.299807E-04   2.186525E-03
```

```
  21    C16    -5.200446E-03  -1.943274E-03   4.012439E-03
  22    C17     1.011256E-03  -1.508706E-04   6.361840E-04
  23    C18    -3.123879E-03  -7.158290E-04  -6.583892E-03
  24    C19    -7.063939E-03  -2.062728E-03   8.090705E-04
  25    H16    -2.103718E-04   4.707942E-04   8.553744E-04
  26    H17     5.839874E-03   2.613085E-03  -4.175018E-03
  27    H18     1.242488E-02   3.786442E-03   1.561713E-03
  28    H19    -3.859295E-03  -1.242690E-03   3.275097E-03
  29    H20     2.965080E-04  -4.598800E-04   1.965477E-03
  30    C1      1.066212E-04  -1.197430E-03   2.415771E-04
  31    H5     -6.245271E-04  -2.326934E-04   4.699059E-05
  32    H6     -9.647908E-05   1.200227E-04   3.096240E-06
  33    C3      2.194370E-03   2.154896E-03   1.522594E-03
  34    C26     2.819150E-03   2.857859E-03  -7.306075E-04
  35    C27    -1.348596E-04  -3.131549E-04  -4.776516E-04
  36    C28    -2.416145E-03  -2.441204E-03  -9.400507E-04
  37    C29    -1.201260E-03  -6.874415E-04   9.218558E-06
  38    H1      7.430163E-04   7.420055E-04   4.313428E-03
  39    H2     -2.921308E-03  -2.500431E-03  -3.182915E-03
  40    H3     -1.090762E-03  -7.223903E-04  -1.231965E-03
  41    H9      2.552610E-03   2.455616E-03   3.038091E-03
  42    H10     7.688328E-05   2.110463E-04   6.463850E-04
  43    H25     1.247264E-05   2.472986E-04   8.043307E-05
 -----------   -------------  -------------  -------------
    total       9.037392E-04   8.158031E-04  -7.771103E-05

  end of program der1b


  start of program geopt   6

  geometry optimization step  6
  reading input hessian of dimension   129
   in five columns format
  reading input hessian of dimension   129
   in five columns format


  Level shifts adjusted to satisfy step-size constraints
   Step size:    0.3005785
   Cos(theta):   0.4030506

   Final level shift:  -1.2859045E-02

  energy change:           5.6334E-04 .  (  5.0000E-05 )
  gradient maximum:        1.2826E-02 .  (  4.5000E-04 )
  gradient rms:            2.6475E-03 .  (  3.0000E-04 )
  step size:   0.30039  trust radius:   0.30000
  displacement maximum:    1.4866E-01 .  (  1.8000E-03 )
  displacement rms:        2.3601E-02 .  (  1.2000E-03 )
  predicted energy change:    -2.6208E-03    geom step:   3.0039E-
01    full step:   3.0039E-01
```

```
molecular structure not yet converged...

center of mass moved by:
   x:    -7.0070E-03     y:     3.1695E-03     z:    -3.9849E-03

new geometry:
                              angstroms
atom            x                  y                  z
N1       -0.2431388835      0.4556736239      1.1723735471
N2       -1.0052221025     -2.2638064326      1.1711775865
C4       -1.2354149005     -0.0068382249      1.9686217617
C5       -0.0219368988     -1.8840112724      0.4769055945
C6       -1.6547168498     -1.3251930492      1.9985456785
C7       -1.7404984224      1.0066322943      2.7505185979
C13       0.6709126692     -2.8977538263     -0.4172186213
H4       -3.0356419670     -2.6439761971      2.9042709974
C8       -2.7930706857      0.6804961350      3.6197611198
C11      -0.9650101197      2.1686595222      2.4024960703
H7       -3.2425652691      1.4227454725      4.2536593268
C9       -3.2422765659     -0.6233719281      3.6535786031
H8       -4.0458719809     -0.8841748627      4.3157805202
C10      -2.6766502685     -1.6346558742      2.8502873309
C12      -0.0710715297      1.7970748741      1.4546947917
C2        0.9503376395      2.6145402001      0.7646068978
H13       1.7459317870     -2.8218826796     -0.2973025660
C14       0.3175412762     -2.7210862854     -1.8808548888
H15       0.3721363370     -3.8731171893     -0.0645970713
C15      -0.4054882560     -2.4055350093     -4.5510673450
C16       1.2049840844     -2.1961843098     -2.8003720449
C17      -0.9385119890     -3.0863025612     -2.3151953733
C18      -1.2985482642     -2.9334100012     -3.6363835117
C19       0.8468309784     -2.0394783637     -4.1317416864
H16       2.1814549757     -1.8985296468     -2.4799685659
H17      -1.6351318329     -3.4951433926     -1.6093012692
H18      -2.2805077802     -3.2390864388     -3.9568211189
H19       1.5442219641     -1.6284067831     -4.8322649240
H20      -0.6918667664     -2.2895583892     -5.5817584789
C1        0.5775477845     -0.4792363854      0.4348181138
H5        1.5772891761     -0.5317014840      0.8498355316
H6        0.6798600143     -0.1840634649     -0.5932120927
C3        2.8710077594      4.1851297153     -0.5136427992
C26       1.9198910499      3.2990087678      1.5101069134
C27       0.9496482049      2.7305127321     -0.6306199707
C28       1.9063251470      3.5077111519     -1.2645504751
C29       2.8739200353      4.0827381339      0.8755772877
H1        1.9381200101      3.2016452634      2.5905377595
H2        0.1888699186      2.2286821548     -1.2204008413
H3        1.8906486427      3.5909148714     -2.3446780704
H9        3.6213149051      4.5998757976      1.4699197044
H10       3.6131544908      4.7887646367     -1.0058448436
H25      -1.0749029726      3.1646160247      2.7777097600
```

```
  nuclear repulsion energy.......  1972.328492632 hartrees
  -------------------------------------------------
 /  end of geometry optimization iteration   6   /
 -------------------------------------------------

  end of program geopt


  start of program onee
  smallest eigenvalue of S:    2.771E-04
  number of canonical orbitals.....          461
  end of program onee


  start of program probe
  end of program probe


  start of program grid

  number of gridpoints:
     atom        N1     N2      C4      C5      C6      C7     C13
H4
  grid # 1       95    102      85      88      89      88      84
73
  grid # 2      103    112      95      95      97      96      92
118
  grid # 3      212    227     194     188     190     198     164
224
  grid # 4      387    415     320     320     327     345     300
223

  number of gridpoints:
     atom        C8    C11      H7      C9      H8     C10     C12
C2
  grid # 1       89     86      73      87      73      89      86
92
  grid # 2       97     94     118      97     118      97      94
100
  grid # 3      184    185     223     184     222     184     193
196
  grid # 4      330    330     226     329     223     326     316
343

  number of gridpoints:
     atom       H13    C14     H15     C15     C16     C17     C18
C19
  grid # 1       69     93      69      89      88      87      89
89
  grid # 2      110    101     109      97      96      96      97
96
```

```
  grid # 3      211    191    213    182    183    182    184
182
  grid # 4      213    338    212    327    327    327    327
327

  number of gridpoints:
     atom        H16    H17    H18    H19    H20     C1     H5
H6
  grid # 1       72     72     73     73     73     82     70
69
  grid # 2      113    115    118    118    118     87    108
103
  grid # 3      214    215    222    221    222    162    217
208
  grid # 4      214    215    224    223    224    290    215
205

  number of gridpoints:
     atom         C3    C26    C27    C28    C29     H1     H2
H3
  grid # 1       89     89     88     89     89     73     71
73
  grid # 2       97     95     96     97     97    115    115
118
  grid # 3      186    184    185    185    185    218    218
222
  grid # 4      331    328    330    328    329    219    215
222

  number of gridpoints:
     atom         H9    H10    H25  total
  grid # 1       73     73     73   3516
  grid # 2      118    118    115   4486
  grid # 3      223    224    221   8628
  grid # 4      224    224    224  12242

  end of program grid


  start of program rwr
  end of program rwr


  start of program scf

       i  u  d  i  g
       t  p  i  c  r                               RMS    maximum
       e  d  i  u  i                      energy  density   DIIS
       r  t  s  t  d     total energy     change  change    error

etot   1  N  N  2  U  -990.31135910434            4.3E-04  4.0E-02
etot   2  Y  Y  6  M  -990.37781182905  6.6E-02  2.3E-04  1.6E-02
```

```
etot   3  N  Y  2  U  -990.38806340056  1.0E-02  6.8E-05  3.7E-03
etot   4  Y  Y  6  M  -990.38873042054  6.7E-04  2.3E-05  1.0E-03
etot   5  Y  Y  6  M  -990.38880803844  7.8E-05  9.7E-06  2.1E-04
etot   6  N  Y  2  U  -990.38880825493  2.2E-07  3.3E-06  4.7E-05
etot   7  Y  N  6  M  -990.38881016479  1.9E-06  0.0E+00  0.0E+00


 Energy components, in hartrees:
   (A)  Nuclear repulsion............  1972.32849263240
   (E)  Total one-electron terms..... -5262.93232379671
   (I)  Total two-electron terms.....  2300.21502099953
   (L)  Electronic energy............ -2962.71730279719  (E+I)
   (N)  Total energy.................  -990.38881016479  (A+L)

 SCFE: SCF energy: HF    -990.38881016479 hartrees   iterations:
7


 HOMO energy:    -0.26932
 LUMO energy:     0.09840

  Orbital energies:
  -15.60367  -15.56424  -11.29601  -11.28477  -11.26958  -11.25339
  -11.24904  -11.24380  -11.24330  -11.24251  -11.24217  -11.24206
  -11.24138  -11.24062  -11.23899  -11.23339  -11.23226  -11.23184
  -11.22993  -11.22656  -11.22352  -11.21808  -11.21636  -11.21470
  -11.21091   -1.32713   -1.24880   -1.16550   -1.15964   -1.13970
   -1.07482   -1.06839   -1.03820   -1.02143   -1.02048   -1.01816
   -0.97093   -0.95526   -0.93500   -0.86046   -0.83462   -0.83327
   -0.83082   -0.80788   -0.79619   -0.76992   -0.73305   -0.71766
   -0.70058   -0.69957   -0.66673   -0.65810   -0.64423   -0.63655
   -0.63289   -0.62318   -0.62001   -0.61738   -0.59598   -0.59206
   -0.59102   -0.58737   -0.58058   -0.55931   -0.55282   -0.54918
   -0.54135   -0.51709   -0.50650   -0.50245   -0.50066   -0.49748
   -0.49337   -0.49087   -0.48836   -0.48092   -0.42887   -0.40467
   -0.36567   -0.34214   -0.34177   -0.33713   -0.33161   -0.28300
   -0.26932    0.09840    0.12812    0.13581    0.14035    0.14932
    0.17951    0.20872    0.22741    0.23483    0.24185

  end of program scf


  start of program der1a
  end of program der1a


  start of program rwr
  end of program rwr


  start of program der1b
```

forces (hartrees/bohr) : total

atom	label	x	y	z
1	N1	2.996888E-03	-1.092300E-03	-5.075419E-03
2	N2	2.902098E-03	9.995089E-05	-2.104699E-03
3	C4	5.384872E-04	-5.237104E-03	-1.331028E-03
4	C5	-2.465605E-03	-1.986060E-03	6.611523E-04
5	C6	4.699496E-03	-2.649344E-03	-4.655830E-03
6	C7	-3.021900E-03	4.173873E-03	3.348288E-03
7	C13	1.016495E-03	3.817286E-03	3.204766E-03
8	H4	-8.333256E-04	-1.623874E-03	3.098643E-04
9	C8	-2.602591E-03	-3.503070E-03	1.654229E-03
10	C11	-1.632059E-03	4.019113E-03	2.604804E-03
11	H7	-3.480026E-04	8.474685E-04	4.877169E-04
12	C9	2.348395E-04	3.465178E-03	6.491069E-04
13	H8	-1.358836E-03	-2.713855E-04	1.230370E-03
14	C10	-2.396643E-03	4.501606E-04	2.552408E-03
15	C12	4.320330E-03	-4.826731E-04	-1.817665E-03
16	C2	4.569580E-03	4.725283E-03	-7.136536E-05
17	H13	2.265698E-03	1.215287E-03	1.281866E-03
18	C14	4.469088E-03	5.048013E-04	5.703955E-03
19	H15	-8.762473E-04	-2.464998E-03	-1.072243E-03
20	C15	-8.066367E-03	-2.185489E-03	-6.728433E-03
21	C16	3.580633E-03	2.454246E-03	-1.704632E-03
22	C17	-1.006307E-02	-3.935131E-03	-1.505949E-03
23	C18	-8.697968E-03	-3.668085E-03	-2.236322E-03
24	C19	7.507799E-03	2.895454E-03	4.149845E-03
25	H16	5.818461E-03	8.205617E-04	1.129787E-03
26	H17	-2.640653E-03	-8.339735E-04	1.123124E-03
27	H18	3.887211E-04	9.312185E-04	7.660585E-05
28	H19	3.923565E-03	1.582982E-03	-1.724279E-03
29	H20	2.529764E-05	3.214141E-05	1.544815E-05
30	C1	-2.579334E-03	-1.237971E-03	4.679537E-03
31	H5	3.899026E-03	3.218813E-04	1.595166E-03
32	H6	1.944755E-04	2.244129E-03	-6.101903E-03
33	C3	-4.312188E-03	-3.167459E-03	1.263702E-03
34	C26	-3.044044E-03	-3.147202E-03	-9.892063E-05
35	C27	-2.661653E-04	-6.060645E-04	2.329980E-03
36	C28	4.226828E-03	3.422783E-03	1.485505E-03
37	C29	3.350305E-04	-9.021500E-06	-3.264345E-03
38	H1	-2.026984E-03	-3.716618E-06	-7.193914E-03
39	H2	4.612104E-03	2.770042E-03	5.079830E-03
40	H3	1.355729E-03	4.917203E-04	5.686416E-03
41	H9	-4.759377E-03	-2.903258E-03	-5.357919E-03
42	H10	-3.207912E-04	-1.602736E-04	-6.972213E-04
43	H25	-5.459823E-04	8.909836E-04	6.012465E-04
total		1.022540E-03	1.008090E-03	1.626298E-04

end of program der1b

```
start of program geopt   7

geometry optimization step  7
reading input hessian of dimension   129
 in five columns format
reading input hessian of dimension   129
 in five columns format
reading input hessian of dimension   129
 in five columns format


Level shifts adjusted to satisfy step-size constraints
 Step size:    0.3001756
 Cos(theta):   0.3481846

 Final level shift:  -5.8225096E-03

energy change:            7.6693E-04 .  (  5.0000E-05 )
gradient maximum:         1.7603E-02 .  (  4.5000E-04 )
gradient rms:             3.4045E-03 .  (  3.0000E-04 )
step size:   0.29981  trust radius:   0.30000
displacement maximum:     1.6540E-01 .  (  1.8000E-03 )
displacement rms:         2.3556E-02 .  (  1.2000E-03 )
predicted energy change:    -2.5296E-03    geom step:   2.9981E-
01    full step:   2.9981E-01
molecular structure not yet converged...

center of mass moved by:
   x:    -3.4490E-03     y:    -8.1566E-03     z:     4.8314E-03

new geometry:
                              angstroms
atom           x                 y                 z
N1       -0.2623089590      0.4311251804      1.1716991713
N2       -1.0180143788     -2.2950059180      1.2042298173
C4       -1.2360309063     -0.0332939736      1.9960257998
C5       -0.0491760452     -1.9161106272      0.4898652496
C6       -1.6476617970     -1.3546394568      2.0462415395
C7       -1.7230911186      0.9831467707      2.7922374034
C13       0.6280306553     -2.9273737531     -0.4121209218
H4       -2.9937724088     -2.6736769475      3.0115780294
C8       -2.7457092522      0.6539618582      3.6975262155
C11      -0.9667588878      2.1493399444      2.4114356404
H7       -3.1781071145      1.3980009698      4.3414501000
C9       -3.1860027373     -0.6506742671      3.7535912964
H8       -3.9650269465     -0.9130115590      4.4450494557
C10      -2.6416558807     -1.6625876251      2.9369875099
C12      -0.0857743078      1.7755955971      1.4454283139
C2        0.9164503009      2.6009974180      0.7343857052
H13       1.7123800183     -2.8842131896     -0.2571412082
```

```
  C14      0.3088978040    -2.7056385440    -1.8863538501
  H15      0.2866736531    -3.9117867885    -0.0961463804
  C15     -0.2973622557    -2.3040914497    -4.5907098684
  C16      1.3065628895    -2.3700351452    -2.7900211464
  C17     -0.9983945440    -2.8282694144    -2.3525013671
  C18     -1.2991393561    -2.6286610044    -3.6904363154
  C19      1.0036021505    -2.1732391689    -4.1341691423
  H16      2.3304900741    -2.2499797277    -2.4490130079
  H17     -1.7797248649    -3.0692236040    -1.6584418267
  H18     -2.3091793966    -2.7128159017    -4.0314102359
  H19      1.7931017057    -1.9116780263    -4.8188293061
  H20     -0.5306398208    -2.1492911389    -5.6298028759
  C1       0.5501654299    -0.5106191206     0.4295419250
  H5       1.5613035628    -0.5554938071     0.8239873713
  H6       0.6303048976    -0.2234538860    -0.6093977434
  C3       2.8015088368     4.1881714110    -0.5810245152
  C26      1.8234927411     3.3659516372     1.4559907329
  C27      0.9590649824     2.6466221398    -0.6560625455
  C28      1.8988761714     3.4310895081    -1.3074873104
  C29      2.7564483373     4.1586935309     0.8023034085
  H1       1.8043237458     3.3261495512     2.5316351329
  H2       0.2432097047     2.0877393438    -1.2297362427
  H3       1.9178440967     3.4562601140    -2.3820556926
  H9       3.4514125005     4.7412314670     1.3756845720
  H10      3.5280434798     4.7976541261    -1.0885472588
  H25     -1.0762728724     3.1476511252     2.7825837903

  nuclear repulsion energy.......  1971.760476131 hartrees
  -------------------------------------------------
 /  end of geometry optimization iteration   7   /
 -------------------------------------------------

  end of program geopt


  start of program onee
  smallest eigenvalue of S:    2.799E-04
  number of canonical orbitals.....          461
  end of program onee


  start of program probe
  end of program probe


  start of program grid

  number of gridpoints:
     atom        N1     N2     C4     C5     C6     C7    C13
H4
  grid # 1       96    102     87     88     89     88     84
73
```

```
  grid # 2      104    112     95     95     97     98     92
118
  grid # 3      214    227    195    187    190    199    164
224
  grid # 4      388    414    320    320    327    348    301
224

  number of gridpoints:
     atom         C8    C11     H7     C9     H8    C10    C12
C2
  grid # 1       89     86     73     87     73     89     84
92
  grid # 2       97     94    118     97    118     97     95
100
  grid # 3      184    185    223    184    222    184    195
195
  grid # 4      330    331    226    329    223    326    321
343

  number of gridpoints:
     atom        H13    C14    H15    C15    C16    C17    C18
C19
  grid # 1       69     91     69     89     88     87     89
88
  grid # 2      110    100    110     97     96     96     97
96
  grid # 3      211    194    215    183    183    183    184
183
  grid # 4      216    340    217    327    329    329    328
327

  number of gridpoints:
     atom        H16    H17    H18    H19    H20     C1     H5
H6
  grid # 1       72     73     73     73     73     82     70
69
  grid # 2      114    114    118    118    118     88    108
105
  grid # 3      214    216    222    222    223    164    217
207
  grid # 4      214    215    224    222    224    290    217
204

  number of gridpoints:
     atom         C3    C26    C27    C28    C29     H1     H2
H3
  grid # 1       89     88     88     89     89     72     71
73
  grid # 2       97     95     96     97     97    115    114
118
  grid # 3      184    182    184    184    184    217    214
222
```

```
  grid # 4      328    327    329    327    328    217    212
224

  number of gridpoints:
     atom        H9    H10    H25  total
  grid # 1       73     73     72   3512
  grid # 2      118    118    115   4492
  grid # 3      222    223    220   8629
  grid # 4      224    224    224  12258

  end of program grid


  start of program rwr
  end of program rwr


  start of program scf

       i  u  d  i  g
       t  p  i  c  r                                     RMS    maximum
       e  d  i  u  i                         energy  density   DIIS
       r  t  s  t  d     total energy    change  change    error

etot   1  N  N  2  U  -990.30270768203            4.6E-04  3.1E-02
etot   2  Y  Y  6  M  -990.37847298562  7.6E-02  2.4E-04  1.2E-02
etot   3  N  Y  2  U  -990.38974615112  1.1E-02  7.3E-05  3.0E-03
etot   4  Y  Y  6  M  -990.39043838018  6.9E-04  2.1E-05  7.2E-04
etot   5  Y  Y  6  M  -990.39051259464  7.4E-05  9.9E-06  1.8E-04
etot   6  N  Y  2  U  -990.39052152447  8.9E-06  3.4E-06  8.4E-05
etot   7  Y  N  6  M  -990.39052390496  2.4E-06  0.0E+00  0.0E+00


 Energy components, in hartrees:
   (A)  Nuclear repulsion............  1971.76047613143
   (E)  Total one-electron terms..... -5261.77830707936
   (I)  Total two-electron terms.....  2299.62730704297
   (L)  Electronic energy............ -2962.15100003639  (E+I)
   (N)  Total energy.................  -990.39052390496  (A+L)

 SCFE: SCF energy: HF    -990.39052390496 hartrees   iterations:
7


 HOMO energy:    -0.26911
 LUMO energy:     0.09715

  Orbital energies:
  -15.60451  -15.56524  -11.29616  -11.28590  -11.27118  -11.25063
  -11.25053  -11.24661  -11.24537  -11.24207  -11.23978  -11.23885
  -11.23840  -11.23770  -11.23689  -11.23687  -11.23575  -11.23433
  -11.23300  -11.23088  -11.22421  -11.21944  -11.21799  -11.21554
```

```
 -11.21243   -1.32546   -1.24901   -1.16385   -1.16049   -1.13916
  -1.07447   -1.06674   -1.04044   -1.02137   -1.01939   -1.01613
  -0.97215   -0.95419   -0.93387   -0.86020   -0.83767   -0.83371
  -0.82913   -0.80726   -0.79554   -0.77026   -0.73264   -0.71762
  -0.70168   -0.69844   -0.66645   -0.65787   -0.64274   -0.63728
  -0.63416   -0.62358   -0.62128   -0.61619   -0.59608   -0.59210
  -0.59055   -0.58653   -0.57915   -0.55835   -0.55307   -0.54811
  -0.54314   -0.51759   -0.50730   -0.50317   -0.50057   -0.49639
  -0.49350   -0.49125   -0.48704   -0.48151   -0.42958   -0.40467
  -0.36720   -0.34424   -0.34058   -0.33732   -0.32999   -0.28301
  -0.26911    0.09715    0.12930    0.13663    0.13689    0.14839
   0.17926    0.20825    0.22812    0.23474    0.24177

 end of program scf


 start of program der1a
 end of program der1a


 start of program rwr
 end of program rwr


 start of program der1b

 forces (hartrees/bohr) : total

atom  label          x              y              z
----  -----   -------------  -------------  -------------
  1    N1      1.716334E-03  -1.943601E-03  -3.246573E-03
  2    N2      1.508741E-03   7.372569E-04  -8.449564E-04
  3    C4      1.732777E-03  -2.550928E-03  -1.144846E-04
  4    C5     -2.258395E-03  -1.157807E-03   1.364650E-03
  5    C6      3.010108E-03  -1.559524E-03  -2.676497E-03
  6    C7     -2.119800E-03   2.378055E-03   8.088183E-05
  7    C13     7.949520E-04  -3.166784E-03   1.193967E-03
  8    H4     -5.137608E-04  -1.262150E-03   5.166284E-05
  9    C8     -1.260196E-03  -1.009633E-03   7.377051E-04
 10    C11     1.581835E-03   1.944482E-03   1.100216E-03
 11    H7     -2.844006E-04   7.476233E-04   4.649418E-04
 12    C9      3.021764E-04   2.034644E-03   3.016599E-04
 13    H8     -9.191824E-04  -1.596108E-04   8.422822E-04
 14    C10    -2.885543E-04  -3.647628E-05   3.084839E-04
 15    C12    -1.002256E-04   1.528865E-04   8.546479E-05
 16    C2     -1.824596E-03  -8.798194E-04   7.008701E-05
 17    H13    -5.907107E-03   4.474195E-05  -1.786917E-03
 18    C14     2.117880E-03   2.467810E-04   1.794266E-03
 19    H15     1.613655E-03   3.598184E-03  -1.510856E-03
 20    C15     3.515496E-03   5.380807E-04   1.351633E-03
 21    C16     3.599045E-03   1.791191E-03  -2.863792E-03
 22    C17     6.384644E-04   6.449569E-04  -1.226921E-03
```

```
  23    C18      3.274344E-03   3.831445E-04   3.904487E-03
  24    C19      3.397778E-03   6.246062E-04   2.548436E-04
  25    H16     -6.033188E-03  -1.648501E-03  -2.241051E-03
  26    H17     -2.244134E-03  -1.119039E-03   1.633890E-03
  27    H18     -5.095790E-03  -1.462176E-03  -5.193377E-04
  28    H19     -7.132674E-04  -3.366359E-04   6.699522E-05
  29    H20      5.473843E-04   5.484700E-05   2.894166E-04
  30    C1      -2.121480E-03   9.323276E-04   7.055723E-04
  31    H5       1.935195E-03   3.195981E-04   1.225099E-03
  32    H6       1.335999E-04   6.505389E-04  -1.350591E-03
  33    C3       2.237467E-04   7.332118E-04   8.228264E-04
  34    C26      2.934216E-03   2.259092E-03   1.769155E-03
  35    C27      1.749225E-03   1.423862E-03  -2.428323E-03
  36    C28     -3.039822E-03  -2.449444E-03  -8.296198E-04
  37    C29     -1.534705E-03  -1.836397E-03   1.746854E-03
  38    H1       1.639685E-04   5.612689E-04  -9.703912E-04
  39    H2      -6.178164E-04  -7.198783E-04  -5.945158E-04
  40    H3      -2.726356E-04  -1.448398E-04  -7.139410E-04
  41    H9       1.428431E-03   1.266800E-03   1.288079E-03
  42    H10      1.793635E-04   8.168534E-05   1.338084E-04
  43    H25     -2.945761E-04   3.841792E-04   3.416161E-04
 -----------   ------------   ------------   ------------
    total        6.550833E-04   1.090803E-03   1.177413E-05

  end of program der1b


  start of program geopt   8

  geometry optimization step  8
  reading input hessian of dimension   129
   in five columns format
  reading input hessian of dimension   129
   in five columns format
  reading input hessian of dimension   129
   in five columns format


  Level shifts adjusted to satisfy step-size constraints
   Step size:    0.3001625
   Cos(theta):   0.3290489

   Final level shift:  -3.2712361E-03

  energy change:          -1.7137E-03 .  (  5.0000E-05 )
  gradient maximum:        6.5993E-03 .  (  4.5000E-04 )
  gradient rms:            1.5880E-03 .  (  3.0000E-04 )
  step size:   0.30014  trust radius:   0.30000
  displacement maximum:    1.3289E-01 .  (  1.8000E-03 )
  displacement rms:        2.3581E-02 .  (  1.2000E-03 )
  predicted energy change:    -1.1454E-03    geom step:   3.0014E-
01    full step:   3.0014E-01
```

molecular structure not yet converged...

center of mass moved by:
x: 1.4339E-03 y: 3.9888E-03 z: 1.3736E-03

new geometry:

	angstroms		
atom	x	y	z
N1	-0.2427301136	0.4133581705	1.1722777583
N2	-1.0454939928	-2.3112638158	1.1699656111
C4	-1.2161957562	-0.0597207232	2.0064760689
C5	-0.0696132821	-1.9370852449	0.4798595573
C6	-1.6544570973	-1.3757803348	2.0263648965
C7	-1.6847856453	0.9623542639	2.8316875226
C13	0.5944122868	-2.9384071474	-0.4387964649
H4	-3.0378534973	-2.6801972707	2.9787940568
C8	-2.7195283903	0.6400467042	3.7315827985
C11	-0.8978500352	2.1256728813	2.4741738095
H7	-3.1321827458	1.3838331907	4.3903874062
C9	-3.1856697575	-0.6615094865	3.7551713096
H8	-3.9748299789	-0.9205218030	4.4383849881
C10	-2.6599327111	-1.6753768988	2.9171144248
C12	-0.0449844390	1.7563976980	1.4791878795
C2	0.9261767262	2.6151140566	0.7518235711
H13	1.6652174605	-2.8719758706	-0.3024610873
C14	0.2876181649	-2.7034242241	-1.9136085293
H15	0.2724733448	-3.9275784756	-0.1477724137
C15	-0.2818249606	-2.3156026969	-4.6110105067
C16	1.3116920078	-2.5254385140	-2.8427232568
C17	-1.0214564856	-2.6804795857	-2.3448902825
C18	-1.3074367587	-2.4976491583	-3.6888738098
C19	1.0337227845	-2.3208152467	-4.1837294462
H16	2.3428974456	-2.5809231360	-2.5252940373
H17	-1.8180053491	-2.8390751217	-1.6358051292
H18	-2.3411635228	-2.5448754781	-4.0117834742
H19	1.8504547007	-2.2045671815	-4.8863974827
H20	-0.5093223464	-2.1903514419	-5.6550411487
C1	0.5725384418	-0.5359270224	0.4467237410
H5	1.5825600909	-0.6025572202	0.8639586010
H6	0.6849443477	-0.2439441492	-0.5977493705
C3	2.7427643680	4.2718109037	-0.5786007634
C26	1.7583262170	3.4655134300	1.4748050376
C27	1.0258000879	2.6093370968	-0.6475070512
C28	1.9286490508	3.4252015088	-1.3062477428
C29	2.6507103176	4.2926508665	0.8143808905
H1	1.7064457766	3.4685024593	2.5540630146
H2	0.3832676468	1.9769465517	-1.2258047756
H3	1.9981625157	3.3991783059	-2.3867378260
H9	3.2735304395	4.9523138350	1.3873712355
H10	3.4343293803	4.9068564617	-1.0932588436
H25	-0.9916197529	3.1137132967	2.8763314713

```
  nuclear repulsion energy.......  1966.909747028 hartrees
  ------------------------------------------------
 /  end of geometry optimization iteration   8   /
 ------------------------------------------------

  end of program geopt


  start of program onee
  smallest eigenvalue of S:    2.927E-04
  number of canonical orbitals.....          461
  end of program onee


  start of program probe
  end of program probe


  start of program grid

  number of gridpoints:
     atom        N1     N2      C4      C5      C6      C7     C13
H4
  grid # 1       96    102      86      88      88      90      84
73
  grid # 2      104    112      94      95     101      98      92
118
  grid # 3      215    228     195     186     189     198     163
223
  grid # 4      388    413     323     321     327     351     300
224

  number of gridpoints:
     atom        C8    C11      H7      C9      H8     C10     C12
C2
  grid # 1       89     87      73      87      73      89      86
91
  grid # 2       97     94     118      97     118      97      95
100
  grid # 3      184    185     223     185     222     184     194
195
  grid # 4      329    331     226     331     224     328     318
343

  number of gridpoints:
     atom       H13    C14     H15     C15     C16     C17     C18
C19
  grid # 1       69     90      69      89      87      88      88
88
  grid # 2      107     98     111      97      96      96      97
97
```

```
  grid # 3      209    195    218    183    184    184    184
182
  grid # 4      207    339    213    329    329    330    331
330

  number of gridpoints:
     atom        H16    H17    H18    H19    H20     C1     H5
H6
  grid # 1       73     73     73     73     73     82     70
69
  grid # 2      114    115    118    118    118     90    110
105
  grid # 3      214    215    223    222    224    166    217
205
  grid # 4      213    215    223    222    224    293    220
207

  number of gridpoints:
     atom         C3    C26    C27    C28    C29     H1     H2
H3
  grid # 1       89     89     88     89     89     72     70
73
  grid # 2       97     96     96     97     97    115    113
118
  grid # 3      186    182    185    184    186    217    213
222
  grid # 4      330    327    328    327    330    217    210
224

  number of gridpoints:
     atom         H9    H10    H25  total
  grid # 1       73     73     72   3513
  grid # 2      118    118    115   4497
  grid # 3      223    223    219   8634
  grid # 4      223    223    222  12263

  end of program grid


  start of program rwr
  end of program rwr


  start of program scf

     i  u  d  i  g
     t  p  i  c  r                                     RMS    maximum
     e  d  i  u  i                          energy  density   DIIS
     r  t  s  t  d     total energy        change  change    error

etot   1  N  N  2  U  -990.33991144147            3.6E-04  2.8E-02
etot   2  Y  Y  6  M  -990.38172607361  4.2E-02  1.8E-04  1.1E-02
```

```
etot   3  N  Y  2  U  -990.38813338998  6.4E-03  5.5E-05  2.7E-03
etot   4  Y  Y  6  M  -990.38854871108  4.2E-04  2.2E-05  8.5E-04
etot   5  Y  Y  6  M  -990.38862044730  7.2E-05  8.4E-06  1.6E-04
etot   6  N  Y  2  U  -990.38863299439  1.3E-05  3.2E-06  5.7E-05
etot   7  Y  N  6  M  -990.38863307052  7.6E-08  0.0E+00  0.0E+00


 Energy components, in hartrees:
   (A)  Nuclear repulsion............  1966.90974702809
   (E)  Total one-electron terms..... -5252.08828716800
   (I)  Total two-electron terms.....  2294.78990706939
   (L)  Electronic energy............ -2957.29838009861  (E+I)
   (N)  Total energy.................  -990.38863307052  (A+L)

 SCFE: SCF energy: HF    -990.38863307052 hartrees   iterations:
7


 HOMO energy:    -0.26918
 LUMO energy:     0.09483

  Orbital energies:
  -15.60546  -15.56553  -11.29617  -11.28888  -11.27440  -11.25323
  -11.25291  -11.24932  -11.24466  -11.23989  -11.23899  -11.23849
  -11.23820  -11.23781  -11.23695  -11.23662  -11.23581  -11.23470
  -11.23345  -11.22918  -11.22676  -11.22253  -11.22247  -11.21821
  -11.21580   -1.32205   -1.25283   -1.16216   -1.16049   -1.13639
   -1.07414   -1.06661   -1.03656   -1.02137   -1.01847   -1.01697
   -0.96944   -0.95479   -0.93227   -0.85933   -0.83776   -0.83195
   -0.82823   -0.80624   -0.79536   -0.76922   -0.73264   -0.71663
   -0.70030   -0.69768   -0.66473   -0.65767   -0.64239   -0.63624
   -0.63253   -0.62481   -0.62019   -0.61335   -0.59730   -0.59249
   -0.58848   -0.58801   -0.57615   -0.55734   -0.55154   -0.54848
   -0.54556   -0.51729   -0.50702   -0.50198   -0.49950   -0.49588
   -0.49449   -0.49099   -0.48779   -0.47966   -0.42997   -0.40493
   -0.36948   -0.34394   -0.33991   -0.33752   -0.32983   -0.28137
   -0.26918    0.09483    0.12818    0.13771    0.13869    0.14741
    0.17628    0.20896    0.22759    0.23471    0.23973

  end of program scf


  start of program der1a
  end of program der1a


  start of program rwr
  end of program rwr


  start of program der1b
```

```
 forces (hartrees/bohr) : total

atom  label          x              y              z
----  -----   -------------  -------------  -------------
  1    N1     -3.608445E-03   2.818146E-03   4.387853E-03
  2    N2     -8.257060E-03  -1.291490E-03   6.873558E-03
  3    C4      2.927401E-04   9.089283E-03   2.291217E-03
  4    C5      1.159396E-02   4.274925E-03  -1.002984E-02
  5    C6     -2.111425E-03   3.526585E-03   5.173623E-03
  6    C7      3.146159E-03  -4.915515E-03  -4.361290E-03
  7    C13     1.197140E-03   3.152175E-03  -4.853313E-03
  8    H4      6.158857E-05   2.208153E-04  -3.754693E-04
  9    C8      3.232912E-03  -4.388872E-03  -3.687738E-03
 10    C11    -2.930359E-03  -7.516295E-03  -1.095850E-03
 11    H7     -5.246514E-04  -5.339326E-04   3.142423E-05
 12    C9      2.211024E-03   7.245822E-04  -2.076187E-03
 13    H8     -9.756688E-05  -4.122068E-04   2.025975E-04
 14    C10     2.685113E-04   4.837745E-03   1.404553E-03
 15    C12     1.505465E-03   8.606128E-04  -3.784882E-03
 16    C2      4.596320E-04  -4.228012E-03  -4.301454E-03
 17    H13     4.376286E-03  -2.059499E-03   1.720359E-03
 18    C14     1.184396E-02   2.108409E-03   5.874802E-03
 19    H15    -6.450204E-04  -1.250607E-03   1.770200E-03
 20    C15    -5.854112E-03   1.356985E-04  -5.815791E-04
 21    C16     3.009072E-03  -1.664308E-03   4.118831E-03
 22    C17    -8.790402E-03  -1.738000E-03  -6.298458E-03
 23    C18    -4.719041E-03  -2.976537E-03  -2.898018E-03
 24    C19     3.419037E-03  -2.514676E-03  -7.224967E-04
 25    H16    -2.647010E-03   1.940020E-03   9.827487E-04
 26    H17     7.504203E-04   1.171925E-03  -1.479661E-03
 27    H18     6.000360E-03   3.165718E-03  -3.106612E-04
 28    H19    -5.276927E-03   3.874991E-04   2.823860E-03
 29    H20    -6.458594E-04   6.314977E-04   1.160412E-04
 30    C1      4.753900E-04  -1.944511E-03  -4.062617E-03
 31    H5     -3.832564E-03  -2.580860E-04  -2.786851E-04
 32    H6     -7.710760E-04  -1.574150E-03   5.124757E-03
 33    C3     -8.483867E-04   5.126358E-04   1.028467E-02
 34    C26    -3.375860E-04   6.706250E-04   2.040083E-04
 35    C27    -2.580503E-03  -2.382582E-03   7.317669E-03
 36    C28    -4.311198E-04  -1.818641E-03  -1.976063E-03
 37    C29     3.356317E-04   9.286614E-04  -7.586848E-03
 38    H1      4.426202E-04   2.672569E-04  -4.159578E-03
 39    H2     -3.082192E-03  -2.219428E-03  -4.993021E-04
 40    H3     -1.537561E-03   6.603144E-05   5.377819E-03
 41    H9      2.343965E-03   1.305606E-03   1.437853E-04
 42    H10     3.434594E-03   3.036993E-03   3.538105E-04
 43    H25     3.444813E-05   5.165315E-04  -1.111761E-03
-----------   -------------  -------------  -------------
   total       9.060454E-04   6.626275E-04   4.644130E-05

 end of program der1b
```

```
start of program geopt   9

geometry optimization step  9
reading input hessian of dimension   129
 in five columns format
reading input hessian of dimension   129
 in five columns format
reading input hessian of dimension   129
 in five columns format
** restarting optimization from step    8 **


Level shifts adjusted to satisfy step-size constraints
 Step size:    0.3007076
 Cos(theta):   0.3121536

 Final level shift:  -6.2152375E-03

energy change:           1.8908E-03 .  (  5.0000E-05 )
gradient maximum:        6.5993E-03 .  (  4.5000E-04 )
gradient rms:            1.5880E-03 .  (  3.0000E-04 )
step size:   0.29990  trust radius:   0.30000
displacement maximum:    1.3848E-01 .  (  1.8000E-03 )
displacement rms:        2.3562E-02 .  (  1.2000E-03 )
predicted energy change:    -1.2255E-03    geom step:   2.9990E-
01    full step:   2.9990E-01
molecular structure not yet converged...

center of mass moved by:
   x:    -7.4940E-16     y:     2.6368E-16     z:    -8.3267E-16

new geometry:
                              angstroms
atom           x                 y                 z
N1       -0.2112213224      0.4759821970      1.1509370987
N2       -0.9304654889     -2.2596315619      1.1557263606
C4       -1.2007566870     -0.0050996186      1.9468199390
C5        0.0308354325     -1.8599684763      0.4447847412
C6       -1.5893298383     -1.3330206181      1.9833821552
C7       -1.7559182331      1.0130893177      2.7060893243
C13       0.7195427103     -2.8544750054     -0.4654951884
H4       -2.9732939203     -2.6636627946      2.8871238361
C8       -2.8165407677      0.6740276669      3.5624798977
C11      -0.9876468060      2.1851605542      2.3748675911
H7       -3.2934365683      1.4196419607      4.1781118649
C9       -3.2309842879     -0.6335021660      3.6104914752
H8       -4.0426550097     -0.9068067006      4.2655024799
C10      -2.6247327918     -1.6447328748      2.8300957907
C12      -0.0663221250      1.8221726220      1.4414763503
C2        0.9615610706      2.6573510475      0.7929901780
H13       1.7804240263     -2.7291419038     -0.3977998181
```

```
C14       0.2904550036     -2.7194671495     -1.9078212042
H15       0.4874628272     -3.8353817165     -0.1033936907
C15      -0.5195728220     -2.5100998311     -4.5600986738
C16       1.1731723675     -2.2603581915     -2.8876463572
C17      -1.0114333568     -3.0801698853     -2.2799362236
C18      -1.4107209530     -2.9767515754     -3.5926183840
C19       0.7731816258     -2.1547371391     -4.2060163333
H16       2.1848694620     -1.9871622113     -2.6169976829
H17      -1.6947814125     -3.4329355076     -1.5285602629
H18      -2.3991425622     -3.2559857461     -3.8614828367
H19       1.4638084288     -1.7938641349     -4.9522904025
H20      -0.8298119621     -2.4271286947     -5.5831759553
C1        0.6128319456     -0.4474688200      0.4042533197
H5        1.6225608382     -0.4852424998      0.8284411699
H6        0.7094088190     -0.1466392262     -0.6361783628
C3        2.9218539781      4.2333037928     -0.3978184205
C26       1.8547642164      3.3951618772      1.5732849714
C27       1.0554555784      2.7250390584     -0.5942672449
C28       2.0276025330      3.5107060596     -1.1848132236
C29       2.8296541634      4.1778589369      0.9812369472
H1        1.7838933136      3.3413965331      2.6388352230
H2        0.3516514934      2.1776688845     -1.2016643395
H3        2.0892544843      3.5634944072     -2.2542289962
H9        3.5239664473      4.7363520807      1.5935527668
H10       3.6885398335      4.8392355334     -0.8576710935
H25      -1.1255993547      3.1803096411      2.7475409393

nuclear repulsion energy.......  1969.045876041 hartrees
-------------------------------------------------
/  end of geometry optimization iteration   9   /
-------------------------------------------------

end of program geopt


start of program onee
smallest eigenvalue of S:    2.808E-04
number of canonical orbitals.....          461
end of program onee


start of program probe
end of program probe


start of program grid

number of gridpoints:
   atom        N1     N2     C4     C5     C6     C7    C13
H4
grid # 1       95    102     84     87     89     90     84
73
```

```
  grid # 2      104    112     95     95    101     98     92
118
  grid # 3      212    227    193    187    192    198    163
224
  grid # 4      386    414    324    320    324    350    294
224

  number of gridpoints:
     atom         C8    C11     H7     C9     H8    C10    C12
C2
  grid # 1       89     86     73     87     73     89     86
90
  grid # 2       97     94    118     97    118     97     94
100
  grid # 3      184    184    223    184    222    184    195
194
  grid # 4      328    332    226    328    223    330    317
341

  number of gridpoints:
     atom        H13    C14    H15    C15    C16    C17    C18
C19
  grid # 1       69     91     69     89     88     87     89
89
  grid # 2      108    100    109     97     96     96     97
97
  grid # 3      211    196    214    186    184    182    183
184
  grid # 4      210    341    212    329    328    326    329
327

  number of gridpoints:
     atom        H16    H17    H18    H19    H20     C1     H5
H6
  grid # 1       72     72     73     73     73     82     70
69
  grid # 2      114    115    118    118    118     90    108
104
  grid # 3      213    215    223    222    223    164    217
206
  grid # 4      214    215    223    223    224    293    219
208

  number of gridpoints:
     atom         C3    C26    C27    C28    C29     H1     H2
H3
  grid # 1       89     87     87     89     89     72     71
73
  grid # 2       97     95     96     97     97    115    114
118
  grid # 3      185    181    182    184    185    217    213
223
```

```
  grid # 4      330    328    330    327    328    216    212
224

  number of gridpoints:
     atom         H9    H10    H25  total
  grid # 1       73     73     72   3507
  grid # 2      118    118    115   4495
  grid # 3      223    223    220   8625
  grid # 4      224    224    224  12249

  end of program grid


  start of program rwr
  end of program rwr


  start of program scf

       i  u  d  i  g
       t  p  i  c  r                                        RMS    maximum
       e  d  i  u  i                              energy  density   DIIS
       r  t  s  t  d     total energy    change  change    error

etot   1  N  N  2  U  -990.19143961399            6.9E-04  5.1E-02
etot   2  Y  Y  6  M  -990.36304368943  1.7E-01  3.6E-04  2.1E-02
etot   3  N  Y  2  U  -990.38756589331  2.5E-02  1.1E-04  4.9E-03
etot   4  Y  Y  6  M  -990.38902919237  1.5E-03  3.0E-05  1.2E-03
etot   5  Y  Y  6  M  -990.38919073874  1.6E-04  1.6E-05  3.2E-04
etot   6  N  Y  2  U  -990.38919602691  5.3E-06  5.3E-06  8.1E-05
etot   7  Y  Y  6  M  -990.38919546120 -5.7E-07  2.6E-06  3.1E-05
etot   8  Y  N  6  M  -990.38919647219  1.0E-06  0.0E+00  0.0E+00


 Energy components, in hartrees:
   (A)  Nuclear repulsion............  1969.04587604074
   (E)  Total one-electron terms..... -5256.35681247047
   (I)  Total two-electron terms.....  2296.92173995754
   (L)  Electronic energy............ -2959.43507251293  (E+I)
   (N)  Total energy.................  -990.38919647219  (A+L)

 SCFE: SCF energy: HF    -990.38919647219 hartrees   iterations:
8


 HOMO energy:    -0.26840
 LUMO energy:     0.09701

  Orbital energies:
  -15.60534  -15.56470  -11.29636  -11.28875  -11.27084  -11.25072
  -11.25052  -11.24617  -11.24576  -11.24057  -11.23986  -11.23892
  -11.23842  -11.23745  -11.23597  -11.23589  -11.23480  -11.23326
```

```
 -11.23202  -11.22987  -11.22361  -11.22021  -11.21809  -11.21556
 -11.21238   -1.32578   -1.25026   -1.16340   -1.15935   -1.13851
  -1.07486   -1.06779   -1.03915   -1.02304   -1.01954   -1.01789
  -0.96974   -0.95431   -0.93406   -0.86044   -0.83631   -0.83390
  -0.82845   -0.80772   -0.79588   -0.77036   -0.73270   -0.71782
  -0.70108   -0.69798   -0.66655   -0.65810   -0.64342   -0.63833
  -0.63434   -0.62250   -0.62043   -0.61751   -0.59675   -0.59349
  -0.59179   -0.58934   -0.57738   -0.55766   -0.55188   -0.54826
  -0.54421   -0.51763   -0.50773   -0.50313   -0.50021   -0.49540
  -0.49235   -0.49068   -0.48759   -0.48192   -0.42965   -0.40506
  -0.36691   -0.34326   -0.34192   -0.33901   -0.32575   -0.28176
  -0.26840    0.09701    0.12789    0.13689    0.13892    0.14831
   0.17873    0.20902    0.22650    0.23488    0.24146

 end of program scf


 start of program der1a
 end of program der1a


 start of program rwr
 end of program rwr


 start of program der1b

 forces (hartrees/bohr) : total

atom  label         x              y              z
----  -----   -------------  -------------  -------------
  1    N1     -1.847904E-03   1.350870E-03   1.372186E-03
  2    N2      8.004859E-04  -1.184322E-03  -1.290199E-03
  3    C4     -2.990647E-03   1.894084E-03  -1.153339E-03
  4    C5     -1.267694E-03  -1.405869E-03   2.168808E-03
  5    C6     -3.781687E-03   1.063700E-03   1.466086E-03
  6    C7      3.701565E-03  -2.358705E-03   9.208506E-04
  7    C13    -6.358814E-03   5.686112E-03   1.117194E-03
  8    H4      7.566979E-04   2.267632E-03   4.118942E-04
  9    C8      3.487603E-04   4.660037E-03   1.792745E-03
 10    C11    -4.761810E-04  -4.721753E-05  -2.385896E-03
 11    H7      6.406881E-04  -9.788330E-04  -1.030604E-03
 12    C9     -1.838024E-03  -6.112861E-03   1.573878E-04
 13    H8      1.321968E-03   2.841587E-04  -1.158728E-03
 14    C10    -9.988378E-04  -2.699473E-03  -2.337905E-04
 15    C12    -3.644818E-04   5.292972E-04   1.985804E-03
 16    C2      4.214487E-03   2.571180E-03   2.054306E-03
 17    H13     1.220174E-02   2.129938E-03   9.413491E-04
 18    C14    -1.971814E-03  -2.257252E-04  -1.000693E-02
 19    H15    -2.739856E-03  -7.545521E-03   3.261445E-03
 20    C15    -3.750255E-04  -1.032296E-03   2.979952E-03
 21    C16     3.971553E-04  -1.694801E-03   1.180222E-02
```

```
  22    C17     1.081296E-02   3.319375E-03   5.677630E-03
  23    C18     1.275942E-02   5.331517E-03  -6.004976E-03
  24    C19    -6.135291E-03  -1.513302E-04  -5.275360E-03
  25    H16    -3.575445E-03  -1.293270E-03   1.989000E-04
  26    H17    -2.282103E-04  -2.076355E-04   5.184705E-04
  27    H18    -1.003847E-02  -2.388316E-03  -4.271375E-03
  28    H19    -1.686569E-03  -1.077089E-03   1.228071E-03
  29    H20    -1.715323E-03  -1.767050E-04  -2.514137E-03
  30    C1      6.153916E-03   1.649965E-03   1.097077E-03
  31    H5     -3.027084E-03  -3.480673E-04  -3.342055E-03
  32    H6     -1.486703E-04  -3.556652E-04   2.982007E-03
  33    C3      1.936388E-06   2.107970E-04  -5.988076E-03
  34    C26    -4.239919E-03  -2.750183E-03  -4.310837E-03
  35    C27    -6.213987E-04  -7.833312E-05   4.417728E-04
  36    C28     4.704144E-03   3.029595E-03   2.767092E-03
  37    C29     2.147902E-03   1.746275E-03   9.650488E-04
  38    H1     -4.030953E-04  -8.930180E-04   4.530742E-03
  39    H2      1.885956E-03   1.855455E-03   5.925202E-04
  40    H3      5.723930E-04   3.930392E-04  -2.575663E-03
  41    H9     -2.986811E-03  -2.049155E-03  -1.829814E-03
  42    H10    -2.808888E-03  -1.906220E-03  -3.199569E-05
  43    H25     3.854478E-04  -1.625851E-04  -2.576043E-04
 -----------   ------------  ------------  ------------
    total       1.181474E-03   8.498306E-04  -2.298122E-04

  end of program der1b


  start of program geopt  10

  geometry optimization step 10
  [ turning on trust-radius adjustment ]
  reading input hessian of dimension   129
   in five columns format
  reading input hessian of dimension   129
   in five columns format
  reading input hessian of dimension   129
   in five columns format
  ** restarting optimization from step    8 **


  energy change:           1.3274E-03 .  (  5.0000E-05 )
  gradient maximum:        6.5993E-03 .  (  4.5000E-04 )
  gradient rms:            1.5880E-03 .  (  3.0000E-04 )
  step size:   0.12023  trust radius:   0.15000
  displacement maximum:    4.6109E-02 .  (  1.8000E-03 )
  displacement rms:        9.4462E-03 .  (  1.2000E-03 )
  predicted energy change:    -6.6309E-04    geom step:   1.2023E-
01    full step:   1.2023E-01
  molecular structure not yet converged...

  center of mass moved by:
```

```
   x:     5.8287E-16     y:     2.6368E-16     z:     5.5511E-17

new geometry:
                              angstroms
atom           x                  y                  z
N1       -0.2503467923      0.4313387940       1.1728975667
N2       -0.9922353568     -2.3008642645       1.2068479080
C4       -1.2177846926     -0.0412413961       2.0027340636
C5       -0.0328950456     -1.9152046665       0.4874677885
C6       -1.6207580303     -1.3658403732       2.0516318574
C7       -1.7256576704      0.9802469690       2.7898913510
C13       0.6345154182     -2.9213500262      -0.4256979858
H4       -2.9784638181     -2.6865579845       3.0093481473
C8       -2.7573880472      0.6486468955       3.6876420923
C11      -0.9686088796      2.1508712541       2.4150585939
H7       -3.2011472190      1.3958055134       4.3235415609
C9       -3.1880965526     -0.6579308626       3.7452763889
H8       -3.9761108933     -0.9234283427       4.4292771187
C10      -2.6259600579     -1.6723713900       2.9368264804
C12      -0.0840044705      1.7791562074       1.4502481760
C2        0.9094340782      2.6151448582       0.7396605597
H13       1.7117184108     -2.8786891597      -0.2812033952
C14       0.3106147337     -2.6930411706      -1.8952436050
H15       0.2973278585     -3.9045733242      -0.1220199652
C15      -0.3176308144     -2.3047012879      -4.5894802502
C16       1.3058774411     -2.4094783712      -2.8200443949
C17      -1.0094991762     -2.7875081323      -2.3386183614
C18      -1.3208168237     -2.5954695037      -3.6744560256
C19       0.9966496806     -2.2130779832      -4.1595138305
H16       2.3268560103     -2.3606962160      -2.4970780760
H17      -1.7899818400     -3.0122134213      -1.6310120439
H18      -2.3448732588     -2.6750402869      -4.0018765889
H19       1.7816422461     -2.0017592358      -4.8628995476
H20      -0.5601115265     -2.1562329340      -5.6282763720
C1        0.5677714222     -0.5074561645       0.4336493303
H5        1.5802783124     -0.5505269015       0.8402390685
H6        0.6558736629     -0.2175416456      -0.6088435807
C3        2.8070428603      4.1983759363      -0.5604794150
C26       1.7923657031      3.4110711813       1.4677798769
C27       0.9824585662      2.6308465889      -0.6519969273
C28       1.9254954757      3.4145041743      -1.2967484498
C29       2.7309857208      4.2007778211       0.8223821304
H1        1.7459445767      3.3977291757       2.5379712574
H2        0.2862125744      2.0466037166      -1.2287363733
H3        1.9713578203      3.4151297035      -2.3708451625
H9        3.4096713505      4.8073562604       1.3990774035
H10       3.5437855062      4.8042079101      -1.0617698905
H25      -1.0891245347      3.1494999185       2.7854402047

nuclear repulsion energy.......  1970.392406452 hartrees
-------------------------------------------------
/  end of geometry optimization iteration  10   /
```

```
 ------------------------------------------------

  end of program geopt


  start of program onee
  smallest eigenvalue of S:    2.841E-04
  number of canonical orbitals.....          461
  end of program onee


  start of program probe
  end of program probe


  start of program grid

  number of gridpoints:
     atom        N1     N2      C4      C5      C6      C7    C13
H4
  grid # 1       96    102      87      88      90      89      84
73
  grid # 2      103    112      95      95      99      97      92
118
  grid # 3      213    227     195     187     191     200     163
224
  grid # 4      389    414     323     320     321     351     300
224

  number of gridpoints:
     atom        C8    C11      H7      C9      H8     C10     C12
C2
  grid # 1       89     86      73      87      73      89      86
92
  grid # 2       97     94     118      97     118      97      94
100
  grid # 3      184    185     223     184     222     184     195
195
  grid # 4      328    331     226     331     223     329     318
342

  number of gridpoints:
     atom       H13    C14     H15     C15     C16     C17     C18
C19
  grid # 1       69     91      69      89      88      88      89
89
  grid # 2      109    100     110      97      96      96      97
96
  grid # 3      210    197     216     183     184     183     184
183
  grid # 4      213    342     213     327     329     327     327
329
```

```
  number of gridpoints:
     atom       H16    H17    H18    H19    H20     C1     H5
H6
  grid # 1       73     73     73     73     73     82     70
69
  grid # 2      113    115    118    118    118     89    110
104
  grid # 3      214    216    223    222    224    165    217
207
  grid # 4      214    214    224    223    224    293    219
206

  number of gridpoints:
     atom        C3    C26    C27    C28    C29     H1     H2
H3
  grid # 1       89     88     88     89     89     72     71
73
  grid # 2       97     95     96     97     97    115    114
118
  grid # 3      184    182    184    184    185    217    212
223
  grid # 4      329    328    329    327    329    216    212
224

  number of gridpoints:
     atom        H9    H10    H25  total
  grid # 1       73     73     72   3519
  grid # 2      118    118    115   4492
  grid # 3      222    223    220   8636
  grid # 4      224    224    224  12260

  end of program grid


  start of program rwr
  end of program rwr


  start of program scf

       i  u  d  i  g
       t  p  i  c  r                                 RMS    maximum
       e  d  i  u  i                     energy  density    DIIS
       r  t  s  t  d     total energy    change  change     error

etot   1  N  N  2  U  -990.29802142608           4.7E-04  3.6E-02
etot   2  Y  Y  6  M  -990.37914076540  8.1E-02  2.4E-04  1.5E-02
etot   3  N  Y  2  U  -990.39011714517  1.1E-02  7.3E-05  3.6E-03
etot   4  Y  Y  6  M  -990.39075170470  6.3E-04  1.8E-05  8.3E-04
etot   5  Y  Y  6  M  -990.39080716088  5.5E-05  9.2E-06  2.4E-04
etot   6  N  Y  2  U  -990.39081625073  9.1E-06  3.2E-06  6.3E-05
```

```
etot   7  Y  N  6  M  -990.39082059817  4.3E-06  0.0E+00  0.0E+00


 Energy components, in hartrees:
   (A)  Nuclear repulsion............  1970.39240645220
   (E)  Total one-electron terms..... -5259.04946585476
   (I)  Total two-electron terms.....  2298.26623880439
   (L)  Electronic energy............ -2960.78322705037  (E+I)
   (N)  Total energy.................  -990.39082059817  (A+L)

 SCFE: SCF energy: HF    -990.39082059817 hartrees   iterations:
7


 HOMO energy:    -0.26884
 LUMO energy:     0.09625

  Orbital energies:
  -15.60520  -15.56542  -11.29634  -11.28757  -11.27197  -11.25137
  -11.25137  -11.24663  -11.24624  -11.24028  -11.24014  -11.23939
  -11.23864  -11.23804  -11.23717  -11.23626  -11.23525  -11.23435
  -11.23339  -11.23131  -11.22496  -11.22092  -11.21941  -11.21652
  -11.21351   -1.32467   -1.25038   -1.16266   -1.16009   -1.13785
   -1.07415   -1.06682   -1.03876   -1.02160   -1.01902   -1.01676
   -0.97049   -0.95423   -0.93360   -0.85986   -0.83666   -0.83382
   -0.82912   -0.80693   -0.79529   -0.76986   -0.73238   -0.71725
   -0.70084   -0.69840   -0.66614   -0.65779   -0.64278   -0.63724
   -0.63391   -0.62371   -0.62052   -0.61551   -0.59631   -0.59205
   -0.59042   -0.58760   -0.57799   -0.55757   -0.55200   -0.54823
   -0.54488   -0.51728   -0.50751   -0.50254   -0.49998   -0.49565
   -0.49359   -0.49112   -0.48733   -0.48118   -0.42972   -0.40500
   -0.36774   -0.34301   -0.34117   -0.33775   -0.32916   -0.28213
   -0.26884    0.09625    0.12876    0.13660    0.13709    0.14832
    0.17835    0.20865    0.22790    0.23513    0.24117

  end of program scf


  start of program der1a
  end of program der1a


  start of program rwr
  end of program rwr


  start of program der1b

  forces (hartrees/bohr) : total

 atom  label          x              y              z
 ----  -----   -------------  -------------  -------------
```

1	N1	3.176707E-04	-7.109467E-05	1.405262E-05
2	N2	-1.075429E-03	-2.083377E-04	7.417913E-04
3	C4	-9.732657E-04	1.912845E-03	4.219490E-04
4	C5	9.390608E-04	-3.118005E-04	-6.400202E-04
5	C6	-1.872859E-03	6.288692E-04	1.567042E-03
6	C7	8.574249E-04	-1.220477E-03	-4.534514E-04
7	C13	4.990676E-05	2.619141E-04	-1.067732E-03
8	H4	2.212391E-04	7.436348E-04	4.175358E-05
9	C8	9.716663E-04	1.126067E-03	-2.443932E-04
10	C11	-3.058807E-04	-1.016420E-03	-2.792513E-05
11	H7	2.030616E-04	-3.912862E-04	-4.531555E-04
12	C9	3.885238E-04	-1.817448E-03	-8.541092E-04
13	H8	5.647280E-04	1.033803E-04	-4.236539E-04
14	C10	1.787747E-04	1.629413E-04	-1.130833E-04
15	C12	-2.993285E-04	9.293299E-04	-8.305882E-04
16	C2	2.106919E-03	3.714443E-04	1.529857E-03
17	H13	-8.453989E-06	-1.369249E-05	-3.866099E-04
18	C14	-2.620975E-03	-8.478820E-04	-2.750261E-04
19	H15	1.703486E-04	2.476269E-04	5.668823E-04
20	C15	1.348587E-03	3.170487E-04	-3.143626E-04
21	C16	-3.287963E-03	-8.576557E-04	-7.679380E-04
22	C17	9.274778E-04	5.528706E-04	2.811386E-05
23	C18	-2.653106E-04	-1.756854E-04	1.189448E-03
24	C19	-2.378424E-03	-1.170097E-03	5.057107E-05
25	H16	3.742774E-03	1.309982E-03	7.701638E-04
26	H17	6.077310E-04	2.216130E-04	-3.997413E-04
27	H18	1.487305E-03	2.292942E-04	4.715166E-04
28	H19	5.016746E-04	5.351337E-04	-4.906618E-04
29	H20	3.420834E-04	-6.617536E-05	7.707523E-04
30	C1	1.310155E-03	1.015916E-03	-6.323303E-04
31	H5	-1.427620E-03	-7.688246E-05	-7.480952E-04
32	H6	-2.814998E-05	-3.196540E-04	2.202996E-03
33	C3	-1.512850E-03	-7.323179E-04	-2.557105E-03
34	C26	-1.040007E-03	-4.215451E-04	-2.994571E-03
35	C27	2.310941E-04	6.193161E-04	-3.498692E-04
36	C28	1.620128E-03	8.085342E-04	2.072791E-03
37	C29	2.143792E-04	-9.341659E-05	4.237368E-04
38	H1	-3.535759E-05	-1.816722E-04	3.011021E-03
39	H2	6.170067E-04	4.683717E-04	1.307347E-04
40	H3	-3.642736E-05	2.655682E-04	-5.930004E-04
41	H9	-9.794912E-04	-8.053914E-04	-4.013531E-04
42	H10	-1.162548E-03	-8.092648E-04	1.701950E-04
43	H25	1.251612E-04	-4.338929E-04	-3.335687E-04
total		7.345388E-04	7.896115E-04	-1.769761E-04

end of program der1b

start of program geopt 11

geometry optimization step 11

```
  reading input hessian of dimension   129
   in five columns format
  reading input hessian of dimension   129
   in five columns format
  reading input hessian of dimension   129
   in five columns format


  Level shifts adjusted to satisfy step-size constraints
   Step size:    0.0750415
   Cos(theta):   0.6886741

   Final level shift:  -2.7962975E-02

  energy change:          -2.9669E-04 .  (  5.0000E-05 )
  gradient maximum:        3.8324E-03 .  (  4.5000E-04 )
  gradient rms:            8.9140E-04 .  (  3.0000E-04 )
  step size:   0.07504  trust radius:   0.07500
  displacement maximum:    2.2233E-02 .  (  1.8000E-03 )
  displacement rms:        5.8958E-03 .  (  1.2000E-03 )
  predicted energy change:    -3.7189E-04    geom step:   7.5041E-
02    full step:   7.5041E-02
  molecular structure not yet converged...

  center of mass moved by:
     x:    -1.1335E-03     y:    -2.5596E-03     z:    -2.5985E-03

  new geometry:
                              angstroms
  atom           x                 y                 z
  N1       -0.2525386544     0.4310493893      1.1683995301
  N2       -1.0138740281    -2.2939256517      1.1975942074
  C4       -1.2252479678    -0.0341646614      1.9952733135
  C5       -0.0436572270    -1.9157862179      0.4851284149
  C6       -1.6413071326    -1.3541973725      2.0405928362
  C7       -1.7145415536     0.9821590281      2.7923186325
  C13       0.6288624322    -2.9244715472     -0.4211405469
  H4       -2.9999644294    -2.6702187494      2.9946387869
  C8       -2.7426672956     0.6546811637      3.6920735521
  C11      -0.9497183169     2.1459198615      2.4208127750
  H7       -3.1762743134     1.3992173904      4.3355281353
  C9       -3.1879856910    -0.6483570844      3.7409436743
  H8       -3.9728895093    -0.9095416004      4.4272694645
  C10      -2.6429704192    -1.6600851796      2.9242590216
  C12      -0.0716241692     1.7744356070      1.4512907646
  C2        0.9269144697     2.6056206604      0.7453112864
  H13       1.7074422886    -2.8817125614     -0.2753663900
  C14       0.3076938336    -2.7045928872     -1.8905668910
  H15       0.2923378719    -3.9050788522     -0.1052980764
  C15      -0.3053244872    -2.3158522487     -4.5878771164
  C16       1.3069751907    -2.4063165422     -2.8077883962
  C17      -1.0079025385    -2.8063076055     -2.3422753578
```

```
  C18      -1.3113854749     -2.6134522525     -3.6776129076
  C19       1.0025465289     -2.2143970874     -4.1491423166
  H16       2.3309432563     -2.3188574629     -2.4786269976
  H17      -1.7917111891     -3.0332743005     -1.6406619581
  H18      -2.3295554842     -2.6925171880     -4.0106917005
  H19       1.7907633063     -1.9834667927     -4.8460183616
  H20      -0.5422240742     -2.1661899720     -5.6255384105
  C1        0.5644095491     -0.5120677085      0.4353968420
  H5        1.5708233822     -0.5629812851      0.8428439319
  H6        0.6581057935     -0.2218487346     -0.5998173568
  C3        2.7889171356      4.2151427700     -0.5660685035
  C26       1.8162112152      3.3890211368      1.4702869848
  C27       0.9775928654      2.6431062201     -0.6456130349
  C28       1.9045321762      3.4402657775     -1.2949648747
  C29       2.7395960663      4.1914658022      0.8181678154
  H1        1.7871549220      3.3588554939      2.5468422945
  H2        0.2728790876      2.0718618690     -1.2204641568
  H3        1.9264167737      3.4627840599     -2.3698183664
  H9        3.4212751180      4.7898184578      1.3932077598
  H10       3.5069237148      4.8342820442     -1.0716438394
  H25      -1.0577771051      3.1427356635      2.7959918279

  nuclear repulsion energy.......  1971.488097852 hartrees
  ------------------------------------------------
 /  end of geometry optimization iteration  11   /
 ------------------------------------------------

  end of program geopt


  start of program onee
  smallest eigenvalue of S:    2.800E-04
  number of canonical orbitals.....          461
  end of program onee


  start of program probe
  end of program probe


  start of program grid

  number of gridpoints:
     atom        N1     N2      C4      C5      C6      C7     C13
H4
  grid # 1       96    102      86      88      89      88      84
73
  grid # 2      103    112      95      95      98      98      92
118
  grid # 3      214    227     195     186     190     199     164
224
```

```
  grid # 4      389    414    322    321    325    347    300
224

  number of gridpoints:
     atom         C8    C11     H7     C9     H8    C10    C12
C2
  grid # 1       89     86     73     87     73     89     86
92
  grid # 2       97     94    118     97    118     97     94
100
  grid # 3      184    184    223    184    222    184    194
195
  grid # 4      330    331    226    329    223    326    317
343

  number of gridpoints:
     atom        H13    C14    H15    C15    C16    C17    C18
C19
  grid # 1       69     91     69     89     88     88     89
89
  grid # 2      110    100    110     97     96     96     97
96
  grid # 3      211    196    214    183    184    183    184
183
  grid # 4      214    340    215    327    327    327    327
327

  number of gridpoints:
     atom        H16    H17    H18    H19    H20     C1     H5
H6
  grid # 1       72     73     73     73     73     82     70
69
  grid # 2      114    115    118    118    118     88    110
104
  grid # 3      214    216    222    222    224    163    217
207
  grid # 4      214    214    224    223    224    292    217
202

  number of gridpoints:
     atom         C3    C26    C27    C28    C29     H1     H2
H3
  grid # 1       89     89     88     89     89     72     71
73
  grid # 2       97     95     96     97     97    115    114
118
  grid # 3      185    182    184    185    184    217    214
222
  grid # 4      328    327    331    327    328    217    212
224

  number of gridpoints:
```

```
   atom        H9    H10    H25  total
 grid # 1       73     73     72   3516
 grid # 2      118    118    115   4493
 grid # 3      223    223    220   8631
 grid # 4      224    224    224  12247

 end of program grid


 start of program scf

      i  u  d  i  g
      t  p  i  c  r                                   RMS    maximum
      e  d  i  u  i                        energy  density   DIIS
      r  t  s  t  d     total energy       change  change     error

etot   1  N  N  1  U  -990.38859773819            8.1E-05  3.6E-03
etot   2  Y  Y  4  M  -990.39048554750  1.9E-03  3.8E-05  1.4E-03
etot   3  Y  Y  4  M  -990.39075521604  2.7E-04  1.1E-05  3.8E-04
etot   4  N  Y  1  U  -990.39078219283  2.7E-05  6.5E-06  1.5E-04
etot   5  Y  Y  4  M  -990.39078684366  4.7E-06  1.8E-06  3.2E-05
etot   6  Y  N  4  M  -990.39078874833  1.9E-06  0.0E+00  0.0E+00


Energy components, in hartrees:
  (A)  Nuclear repulsion............  1971.48809785247
  (E)  Total one-electron terms..... -5261.23708829039
  (I)  Total two-electron terms.....  2299.35820168958
  (L)  Electronic energy............ -2961.87888660080  (E+I)
  (N)  Total energy.................  -990.39078874833  (A+L)

SCFE: SCF energy: HF    -990.39078874833 hartrees   iterations:
6


HOMO energy:    -0.26900
LUMO energy:     0.09681

 Orbital energies:
 -15.60450  -15.56520  -11.29613  -11.28583  -11.27124  -11.25060
 -11.25060  -11.24629  -11.24564  -11.24013  -11.23934  -11.23862
 -11.23812  -11.23741  -11.23665  -11.23611  -11.23489  -11.23442
 -11.23272  -11.23078  -11.22433  -11.21958  -11.21829  -11.21571
 -11.21257   -1.32518   -1.24924   -1.16401   -1.16055   -1.13914
  -1.07477   -1.06714   -1.04013   -1.02193   -1.01901   -1.01706
  -0.97170   -0.95432   -0.93398   -0.86039   -0.83766   -0.83356
  -0.82897   -0.80722   -0.79561   -0.77030   -0.73280   -0.71772
  -0.70154   -0.69847   -0.66672   -0.65798   -0.64297   -0.63737
```

```
 -0.63423   -0.62375   -0.62103   -0.61586   -0.59622   -0.59244
 -0.59059   -0.58747   -0.57852   -0.55830   -0.55278   -0.54840
 -0.54412   -0.51758   -0.50739   -0.50284   -0.50056   -0.49599
 -0.49355   -0.49122   -0.48722   -0.48146   -0.42973   -0.40470
 -0.36763   -0.34405   -0.34100   -0.33769   -0.32924   -0.28262
 -0.26900    0.09681    0.12911    0.13706    0.13737    0.14842
  0.17898    0.20870    0.22842    0.23520    0.24150

end of program scf


start of program der1a
end of program der1a


start of program rwr
end of program rwr


start of program der1b

forces (hartrees/bohr) : total

atom  label          x              y              z
----  -----   -------------  -------------  -------------
  1     N1      7.004627E-04  -5.345029E-04  -8.693736E-04
  2     N2      1.325105E-03   4.490218E-04  -1.207276E-03
  3     C4      6.466353E-04  -1.748891E-03  -5.276414E-04
  4     C5     -1.583901E-03  -5.770104E-05   1.464054E-03
  5     C6      2.273493E-03  -1.141576E-03  -2.212237E-03
  6     C7     -6.504121E-04   1.484241E-03   1.389573E-04
  7     C13    -1.049263E-04  -1.124955E-03   1.171673E-03
  8     H4     -4.026407E-04  -9.691760E-04  -5.464798E-05
  9     C8     -9.303924E-04  -6.391797E-04   4.551451E-04
 10     C11     6.920090E-04   1.520404E-03   3.671516E-04
 11     H7     -7.863954E-05   4.226999E-04   2.440591E-04
 12     C9     -2.025401E-04   1.476436E-03   5.741089E-04
 13     H8     -5.400360E-04  -1.340263E-04   4.904071E-04
 14     C10    -7.800840E-05  -1.362858E-04   6.780081E-04
 15     C12    -2.323231E-04  -3.858693E-04   8.130474E-04
 16     C2     -1.650918E-03  -1.050059E-03  -4.870314E-04
 17     H13    -1.249323E-03   3.298007E-04  -3.456024E-04
 18     C14     3.372735E-04   3.524076E-05  -8.065477E-04
 19     H15     3.415639E-04   3.736382E-04  -5.501722E-04
 20     C15    -7.211408E-05  -4.291507E-04   1.165413E-03
 21     C16     1.485377E-04   5.669822E-04   3.571922E-04
 22     C17     9.294633E-04   4.954721E-05   8.698192E-04
 23     C18     1.506381E-03   3.791037E-04  -2.957498E-04
 24     C19     1.342766E-03   5.454351E-04  -6.709570E-04
 25     H16    -1.552766E-03  -6.560598E-04  -2.441234E-04
 26     H17     1.700841E-04  -9.802381E-05   1.903320E-04
 27     H18    -1.365794E-03  -2.753192E-04  -2.401760E-04
```

```
  28    H19    -6.056477E-04  -1.969351E-04   3.560983E-04
  29    H20    -1.099160E-04   1.214393E-04  -7.279111E-04
  30    C1     -1.851382E-03  -1.238949E-04   1.290145E-03
  31    H5      1.574782E-03   1.314021E-04   1.035441E-03
  32    H6      3.969414E-04   6.577154E-04  -2.503026E-03
  33    C3      1.115385E-03   1.071782E-03   1.756491E-03
  34    C26     1.717762E-03   1.497857E-03   2.251613E-03
  35    C27     3.012996E-04   7.204426E-04  -9.322043E-04
  36    C28    -1.972431E-03  -1.672589E-03  -1.659435E-03
  37    C29    -8.471681E-04  -7.644455E-04   8.396384E-04
  38    H1      2.871811E-04   2.205505E-04  -1.629586E-03
  39    H2     -7.152317E-04  -9.510178E-04  -5.023593E-04
  40    H3      1.101136E-05  -8.687548E-05  -4.894680E-04
  41    H9      1.039112E-03   8.927797E-04   5.902413E-04
  42    H10     7.541013E-04   6.428744E-04  -3.083269E-04
  43    H25    -2.161468E-04   5.428347E-04   2.594211E-04
 -----------   -------------  -------------  -------------
    total       5.986926E-04   9.556962E-04   9.460540E-05

  end of program der1b


  start of program geopt  12

  geometry optimization step 12
  reading input hessian of dimension   129
   in five columns format
  reading input hessian of dimension   129
   in five columns format


  Level shifts adjusted to satisfy step-size constraints
   Step size:    0.0381814
   Cos(theta):   0.6358626

   Final level shift:  -2.2947896E-02

  energy change:            3.1850E-05 *  (  5.0000E-05 )
  gradient maximum:         3.7159E-03 .  (  4.5000E-04 )
  gradient rms:             7.8551E-04 .  (  3.0000E-04 )
  step size:   0.03818  trust radius:   0.03750
  displacement maximum:     1.1555E-02 .  (  1.8000E-03 )
  displacement rms:         2.9998E-03 .  (  1.2000E-03 )
  predicted energy change:    -1.3809E-04    geom step:   3.8181E-
02    full step:   3.8181E-02
  molecular structure not yet converged...

  center of mass moved by:
     x:     4.5426E-04     y:     8.1147E-04     z:    -6.7642E-04

  new geometry:
                               angstroms
```

```
atom           x                  y                  z
N1       -0.2481100487      0.4334566292      1.1690590843
N2       -1.0032005669     -2.2934581620      1.1949409475
C4       -1.2219414340     -0.0343838020      1.9923563545
C5       -0.0339796710     -1.9120602342      0.4839591872
C6       -1.6327378826     -1.3563177585      2.0371288910
C7       -1.7214706152      0.9842720805      2.7832559656
C13       0.6387689584     -2.9205192316     -0.4241415240
H4       -2.9960643356     -2.6736608684      2.9862523038
C8       -2.7543266162      0.6556694707      3.6773710238
C11      -0.9567330317      2.1503186557      2.4169640341
H7       -3.1942470497      1.4021628828      4.3154141299
C9       -3.1938710405     -0.6480229029      3.7284797792
H8       -3.9830026483     -0.9107663027      4.4110346409
C10      -2.6387220303     -1.6617110159      2.9180186584
C12      -0.0739060768      1.7781436478      1.4520699323
C2        0.9254724195      2.6102272532      0.7486369541
H13       1.7151095547     -2.8707079086     -0.2840967415
C14       0.3101739106     -2.7047976511     -1.8919547337
H15       0.3108881878     -3.9021230759     -0.1082165064
C15      -0.3249288630     -2.3285963028     -4.5840286299
C16       1.2990880581     -2.4022115814     -2.8172990363
C17      -1.0064246650     -2.8179513158     -2.3322450242
C18      -1.3210023891     -2.6315174078     -3.6652839004
C19       0.9856382061     -2.2157562838     -4.1570604658
H16       2.3222336572     -2.3129300127     -2.4940718207
H17      -1.7807423369     -3.0503320668     -1.6229685692
H18      -2.3412419940     -2.7221754401     -3.9894950791
H19       1.7641119017     -1.9818956103     -4.8615237321
H20      -0.5712857690     -2.1834408899     -5.6215168996
C1        0.5743863458     -0.5077215009      0.4405346425
H5        1.5783628755     -0.5595077770      0.8633906677
H6        0.6828551014     -0.2141085169     -0.5964581000
C3        2.7976584351      4.2167944296     -0.5536768887
C26       1.8195941604      3.3876491689      1.4793453416
C27       0.9766033142      2.6525116204     -0.6445526402
C28       1.9079513036      3.4480386227     -1.2902513713
C29       2.7477220187      4.1880060271      0.8321252429
H1        1.7898857173      3.3527087763      2.5543504826
H2        0.2700912980      2.0817515541     -1.2208892319
H3        1.9329210192      3.4750874837     -2.3661874570
H9        3.4352847020      4.7809146017      1.4097776804
H10       3.5220132279      4.8352318702     -1.0559521854
H25      -1.0710358057      3.1480250996      2.7899724688

nuclear repulsion energy.......  1971.062170283 hartrees
------------------------------------------------
/  end of geometry optimization iteration  12   /
------------------------------------------------

end of program geopt
```

```
  start of program onee
  smallest eigenvalue of S:    2.810E-04
  number of canonical orbitals.....           461
  end of program onee


  start of program probe
  end of program probe


  start of program grid

  number of gridpoints:
     atom        N1     N2      C4      C5      C6      C7     C13
H4
  grid # 1       95    102      87      87      90      89      84
73
  grid # 2      103    112      95      95      99      98      92
118
  grid # 3      214    227     195     187     191     199     163
224
  grid # 4      385    414     321     320     323     348     299
224

  number of gridpoints:
     atom        C8    C11      H7      C9      H8     C10     C12
C2
  grid # 1       89     86      73      87      73      89      86
92
  grid # 2       97     94     118      97     118      97      94
100
  grid # 3      184    185     223     184     222     184     194
195
  grid # 4      328    331     226     331     223     329     317
343

  number of gridpoints:
     atom       H13    C14     H15     C15     C16     C17     C18
C19
  grid # 1       69     91      69      89      88      87      89
89
  grid # 2      109    100     110      97      96      96      97
96
  grid # 3      211    196     214     183     184     182     184
183
  grid # 4      214    340     213     327     327     327     327
327

  number of gridpoints:
     atom       H16    H17     H18     H19     H20      C1      H5
H6
```

```
  grid # 1       72     73     73     73     73     82     70
69
  grid # 2      114    114    118    118    118     88    109
104
  grid # 3      214    216    222    221    224    164    217
207
  grid # 4      214    214    224    223    224    292    219
206

  number of gridpoints:
     atom        C3    C26    C27    C28    C29     H1     H2
H3
  grid # 1       89     88     88     89     89     72     71
73
  grid # 2       97     95     96     97     97    115    114
118
  grid # 3      185    182    184    185    185    217    213
223
  grid # 4      328    327    330    328    328    217    212
224

  number of gridpoints:
     atom        H9    H10    H25  total
  grid # 1       73     73     72   3515
  grid # 2      118    118    115   4491
  grid # 3      223    224    220   8634
  grid # 4      224    224    224  12246

  end of program grid


  start of program rwr
  end of program rwr


  start of program scf

       i  u  d  i  g
       t  p  i  c  r                                RMS    maximum
       e  d  i  u  i                      energy  density   DIIS
       r  t  s  t  d     total energy     change  change    error

etot   1  N  N  1  U  -990.39028009303           4.3E-05  2.4E-03
etot   2  Y  Y  4  M  -990.39078089231  5.0E-04  1.8E-05  5.5E-04
etot   3  Y  Y  4  M  -990.39083518771  5.4E-05  4.9E-06  1.2E-04
etot   4  Y  N  4  M  -990.39084155577  6.4E-06  0.0E+00  0.0E+00


 Energy components, in hartrees:
   (A)  Nuclear repulsion............  1971.06217028341
   (E)  Total one-electron terms..... -5260.39454868744
   (I)  Total two-electron terms.....  2298.94153684826
```

```
   (L)  Electronic energy........... -2961.45301183918  (E+I)
   (N)  Total energy................  -990.39084155577  (A+L)

 SCFE: SCF energy: HF    -990.39084155577 hartrees   iterations:
4


 HOMO energy:    -0.26906
 LUMO energy:     0.09673

  Orbital energies:
  -15.60462  -15.56496  -11.29644  -11.28704  -11.27124  -11.25104
  -11.25071  -11.24585  -11.24537  -11.24021  -11.23959  -11.23927
  -11.23874  -11.23808  -11.23742  -11.23573  -11.23438  -11.23415
  -11.23269  -11.23001  -11.22431  -11.22021  -11.21853  -11.21599
  -11.21287   -1.32532   -1.24973   -1.16308   -1.16069   -1.13877
   -1.07464   -1.06716   -1.03922   -1.02184   -1.01901   -1.01724
   -0.97091   -0.95440   -0.93382   -0.86015   -0.83698   -0.83354
   -0.82928   -0.80716   -0.79551   -0.77003   -0.73266   -0.71746
   -0.70102   -0.69855   -0.66633   -0.65789   -0.64293   -0.63722
   -0.63387   -0.62365   -0.62070   -0.61586   -0.59633   -0.59238
   -0.59043   -0.58757   -0.57842   -0.55802   -0.55233   -0.54821
   -0.54447   -0.51739   -0.50720   -0.50256   -0.50022   -0.49593
   -0.49341   -0.49120   -0.48742   -0.48125   -0.42975   -0.40484
   -0.36746   -0.34335   -0.34121   -0.33758   -0.32931   -0.28234
   -0.26906    0.09673    0.12890    0.13698    0.13758    0.14860
    0.17856    0.20869    0.22823    0.23521    0.24124

  end of program scf


  start of program der1a
  end of program der1a


  start of program rwr
  end of program rwr


  start of program der1b

  forces (hartrees/bohr) : total

 atom  label         x             y             z
 ----  -----   ------------- ------------- -------------
   1    N1      2.227998E-04  3.002457E-04 -7.633866E-04
   2    N2      8.313377E-04  6.278822E-05 -8.329915E-04
   3    C4     -2.749466E-04 -4.216903E-04 -5.263447E-07
   4    C5     -6.186970E-04 -7.144881E-04  3.670361E-04
   5    C6      2.786252E-04  3.974446E-05 -3.989161E-04
   6    C7      9.204291E-05  2.064961E-04  1.809267E-04
   7    C13    -2.647782E-04  7.202521E-04  7.337563E-04
```

```
   8    H4       4.755602E-05   2.153163E-04   1.133755E-04
   9    C8      -3.968861E-04   6.246385E-04   6.455230E-04
  10    C11      1.871446E-05   4.167742E-04  -1.449800E-04
  11    H7       4.661710E-05  -2.622866E-05   2.075457E-05
  12    C9      -4.135986E-04  -8.043030E-04   2.383556E-04
  13    H8       9.219046E-05   1.817128E-05  -3.487273E-05
  14    C10     -5.487297E-04  -5.031939E-04   3.007736E-04
  15    C12      4.545402E-04   3.326283E-05   3.764629E-06
  16    C2       3.716824E-04   4.474287E-04  -1.073648E-03
  17    H13      6.660705E-04   2.713936E-04  -4.580298E-05
  18    C14     -9.524221E-06  -1.503978E-05  -1.155393E-04
  19    H15     -2.589289E-04  -4.741291E-04   1.596626E-04
  20    C15     -3.296994E-04  -8.513451E-05  -3.637118E-04
  21    C16      5.059299E-04   2.273868E-04   1.343442E-04
  22    C17      1.784530E-04   6.295302E-06   3.790300E-04
  23    C18      4.746219E-04   1.823099E-04  -5.954331E-04
  24    C19     -1.022632E-04   1.979797E-04   6.156296E-05
  25    H16      3.316220E-04  -1.320570E-04   5.700000E-06
  26    H17     -4.687600E-04  -9.797374E-05   2.750276E-04
  27    H18     -1.205098E-03  -6.438215E-05  -3.681211E-04
  28    H19      4.370449E-04  -4.587109E-06  -2.768543E-05
  29    H20      1.076385E-05  -4.651129E-05   2.680799E-04
  30    C1       1.139672E-03   1.536346E-04   3.587566E-04
  31    H5      -4.346405E-04  -9.406025E-07  -3.907024E-04
  32    H6      -1.517462E-04  -9.902310E-05   3.357495E-04
  33    C3       1.185490E-04   2.269726E-04   5.102438E-04
  34    C26     -2.577180E-06   8.957851E-05  -5.609457E-05
  35    C27      1.261047E-04   2.099169E-04   7.317389E-04
  36    C28      2.097073E-04   1.577945E-04   1.716548E-04
  37    C29      3.597928E-05  -6.029637E-05  -9.796868E-04
  38    H1       9.699556E-05   7.995279E-05  -5.711064E-04
  39    H2       8.836115E-05  -5.681230E-05   5.451637E-05
  40    H3      -2.236567E-05  -8.217920E-05   5.481682E-04
  41    H9      -1.316816E-04   3.301862E-05  -1.580912E-04
  42    H10     -5.437240E-04  -3.859753E-04   3.434680E-04
  43    H25     -4.214022E-06  -3.049285E-05   3.941865E-06
-----------   -------------  -------------  -------------
   total         6.931218E-04   8.159137E-04   2.461458E-05

 end of program der1b


 start of program geopt  13

 geometry optimization step 13
 reading input hessian of dimension   129
  in five columns format
 reading input hessian of dimension   129
  in five columns format


 Level shifts adjusted to satisfy step-size constraints
```

```
   Step size:    0.0192086
   Cos(theta):   0.7007821

   Final level shift:  -6.0023224E-02

  energy change:          -5.2807E-05 .  (  5.0000E-05 )
  gradient maximum:        1.3227E-03 .  (  4.5000E-04 )
  gradient rms:            3.7901E-04 .  (  3.0000E-04 )
  step size:   0.01921  trust radius:   0.01875
  displacement maximum:    6.9080E-03 .  (  1.8000E-03 )
  displacement rms:        1.5092E-03 .  (  1.2000E-03 )
  predicted energy change:    -4.3542E-05    geom step:   1.9209E-
02    full step:   1.9209E-02
  molecular structure not yet converged...

  center of mass moved by:
     x:    -2.9109E-04     y:     4.6312E-04     z:     3.9301E-04

  new geometry:
                               angstroms
  atom          x                 y                 z
  N1       -0.2488255692      0.4322339843      1.1698872896
  N2       -1.0058226391     -2.2950440448      1.1945544518
  C4       -1.2229240041     -0.0361424909      1.9939013709
  C5       -0.0365007616     -1.9141015496      0.4855354067
  C6       -1.6347093114     -1.3580296563      2.0375911084
  C7       -1.7199951420      0.9823189968      2.7870807318
  C13       0.6348421100     -2.9216660268     -0.4233610698
  H4       -2.9973932708     -2.6761090969      2.9877417657
  C8       -2.7524946331      0.6532233934      3.6829849331
  C11      -0.9547024201      2.1488365611      2.4190560905
  H7       -3.1905897249      1.3985912480      4.3231430452
  C9       -3.1932086574     -0.6517690880      3.7325641745
  H8       -3.9812949477     -0.9149495298      4.4155551603
  C10      -2.6401022769     -1.6646647698      2.9195236102
  C12      -0.0728836370      1.7767980278      1.4534342970
  C2        0.9248616477      2.6094547063      0.7465477863
  H13       1.7116541383     -2.8719037968     -0.2842566294
  C14       0.3090127646     -2.7017285669     -1.8919217197
  H15       0.3057087418     -3.9036615388     -0.1088356875
  C15      -0.3181517155     -2.3211174476     -4.5844573536
  C16       1.3022806045     -2.4054300452     -2.8151364504
  C17      -1.0074098438     -2.8056006886     -2.3339389563
  C18      -1.3187367817     -2.6172398029     -3.6681293975
  C19       0.9926701615     -2.2159193741     -4.1546871043
  H16       2.3260666979     -2.3274211924     -2.4914155295
  H17      -1.7860704955     -3.0349791182     -1.6266254226
  H18      -2.3425831130     -2.7043182588     -3.9940814180
  H19       1.7761371458     -1.9889515630     -4.8563274949
  H20      -0.5618666539     -2.1757307323     -5.6219403179
  C1        0.5740559454     -0.5100177170      0.4417086690
  H5        1.5784350739     -0.5618273663      0.8611698811
```

```
  H6        0.6798161828     -0.2186286872     -0.5959096950
  C3        2.7946536701      4.2153968164     -0.5592303079
  C26       1.8165501131      3.3931684312      1.4740306347
  C27       0.9780419294      2.6451051479     -0.6454457865
  C28       1.9082602778      3.4400244963     -1.2927166344
  C29       2.7429412688      4.1934511473      0.8251154766
  H1        1.7882002850      3.3627714017      2.5482192846
  H2        0.2755486666      2.0683734653     -1.2209902818
  H3        1.9348576060      3.4600611274     -2.3678965230
  H9        3.4273881104      4.7918441078      1.4013512856
  H10       3.5166904418      4.8328913912     -1.0628356014
  H25      -1.0679447669      3.1461658281      2.7930681050

  nuclear repulsion energy.......  1971.091366916 hartrees
  ------------------------------------------------
 /  end of geometry optimization iteration  13   /
 ------------------------------------------------

  end of program geopt


  start of program onee
  smallest eigenvalue of S:    2.824E-04
  number of canonical orbitals.....          461
  end of program onee


  start of program probe
  end of program probe


  start of program grid

  number of gridpoints:
     atom        N1     N2      C4      C5      C6      C7    C13
H4
  grid # 1       96    102      87      87      90      90     84
73
  grid # 2      103    112      95      95      99      98     92
118
  grid # 3      214    227     195     187     191     199    163
224
  grid # 4      389    414     321     320     323     347    299
224

  number of gridpoints:
     atom        C8    C11      H7      C9      H8     C10    C12
C2
  grid # 1       89     86      73      87      73      89     86
92
  grid # 2       97     94     118      97     118      97     94
100
```

```
  grid # 3      184    185    223    184    222    184    194
196
  grid # 4      329    331    226    331    223    329    317
341

  number of gridpoints:
     atom       H13    C14    H15    C15    C16    C17    C18
C19
  grid # 1       69     93     69     89     88     88     89
89
  grid # 2      109    100    110     97     96     96     97
96
  grid # 3      210    195    214    183    184    183    184
183
  grid # 4      214    340    213    327    327    327    327
327

  number of gridpoints:
     atom       H16    H17    H18    H19    H20     C1     H5
H6
  grid # 1       72     73     73     73     73     82     70
69
  grid # 2      114    115    118    118    118     88    110
104
  grid # 3      214    216    223    222    224    164    217
207
  grid # 4      214    214    224    223    224    293    217
205

  number of gridpoints:
     atom        C3    C26    C27    C28    C29     H1     H2
H3
  grid # 1       89     88     88     89     89     72     71
73
  grid # 2       97     95     96     97     97    115    114
118
  grid # 3      185    182    184    184    185    217    213
223
  grid # 4      328    328    329    328    328    217    212
224

  number of gridpoints:
     atom        H9    H10    H25  total
  grid # 1       73     73     72   3520
  grid # 2      118    118    115   4493
  grid # 3      223    223    220   8634
  grid # 4      224    224    224  12246

  end of program grid


  start of program rwr
```

```
  end of program rwr


  start of program scf

       i  u  d  i  g
       t  p  i  c  r                                 RMS    maximum
       e  d  i  u  i                       energy  density   DIIS
       r  t  s  t  d     total energy     change  change    error

etot   1  N  N  1  U  -990.39066938504           2.3E-05  1.1E-03
etot   2  Y  Y  4  M  -990.39082701468  1.6E-04  1.1E-05  4.2E-04
etot   3  Y  Y  4  M  -990.39084593688  1.9E-05  3.2E-06  1.1E-04
etot   4  Y  N  4  M  -990.39084898231  3.0E-06  0.0E+00  0.0E+00


 Energy components, in hartrees:
   (A)  Nuclear repulsion............  1971.09136691580
   (E)  Total one-electron terms..... -5260.45116671076
   (I)  Total two-electron terms.....  2298.96895081265
   (L)  Electronic energy............ -2961.48221589810  (E+I)
   (N)  Total energy.................  -990.39084898231  (A+L)

 SCFE: SCF energy: HF    -990.39084898231 hartrees   iterations:
4


 HOMO energy:    -0.26909
 LUMO energy:     0.09670

  Orbital energies:
  -15.60482  -15.56511  -11.29607  -11.28679  -11.27163  -11.25115
  -11.25090  -11.24617  -11.24561  -11.23984  -11.23956  -11.23889
  -11.23843  -11.23775  -11.23701  -11.23573  -11.23460  -11.23444
  -11.23276  -11.23041  -11.22479  -11.22011  -11.21902  -11.21611
  -11.21311   -1.32505   -1.25012   -1.16329   -1.16072   -1.13844
   -1.07455   -1.06701   -1.03926   -1.02193   -1.01882   -1.01722
   -0.97108   -0.95429   -0.93372   -0.86021   -0.83691   -0.83360
   -0.82903   -0.80715   -0.79555   -0.77004   -0.73267   -0.71741
   -0.70115   -0.69841   -0.66628   -0.65791   -0.64291   -0.63702
   -0.63380   -0.62376   -0.62069   -0.61579   -0.59625   -0.59229
   -0.59055   -0.58752   -0.57825   -0.55807   -0.55233   -0.54821
   -0.54447   -0.51744   -0.50724   -0.50258   -0.50023   -0.49595
   -0.49354   -0.49120   -0.48746   -0.48116   -0.42969   -0.40485
   -0.36758   -0.34333   -0.34129   -0.33767   -0.32932   -0.28244
   -0.26909    0.09670    0.12895    0.13716    0.13754    0.14851
    0.17843    0.20872    0.22830    0.23522    0.24111

  end of program scf


  start of program der1a
```

```
 end of program der1a


 start of program rwr
 end of program rwr


 start of program der1b

 forces (hartrees/bohr) : total
```

atom	label	x	y	z
1	N1	4.528739E-04	-2.150721E-04	-7.189734E-04
2	N2	-5.179471E-04	-3.795031E-05	3.540438E-04
3	C4	1.453513E-04	-6.415916E-05	2.186439E-05
4	C5	7.956954E-04	-7.125645E-05	-6.055630E-04
5	C6	1.674864E-04	-8.425208E-05	-1.081652E-04
6	C7	-2.512909E-04	2.681225E-04	1.706745E-04
7	C13	2.264142E-04	5.762714E-04	8.823663E-05
8	H4	-6.045948E-05	-1.622022E-04	5.346281E-05
9	C8	-9.811810E-06	-6.163186E-04	-1.033681E-04
10	C11	-3.140914E-04	-5.043245E-07	3.741302E-04
11	H7	-7.779658E-05	9.048357E-05	3.931211E-05
12	C9	2.767482E-04	5.658957E-04	-1.033924E-04
13	H8	-1.429809E-04	-1.901601E-05	1.548469E-04
14	C10	-7.750078E-05	3.688975E-04	2.521305E-04
15	C12	4.616740E-04	5.257927E-06	-2.829912E-04
16	C2	2.944823E-04	2.233180E-04	5.568139E-04
17	H13	4.427948E-04	6.578201E-05	1.726383E-04
18	C14	1.282378E-04	-1.306465E-04	5.130233E-04
19	H15	-1.131664E-04	-2.810631E-04	-3.533440E-05
20	C15	-5.253545E-04	1.215568E-05	-8.190267E-05
21	C16	1.228580E-04	-7.128547E-05	5.101624E-04
22	C17	-7.451867E-04	-2.526086E-04	2.209580E-04
23	C18	-1.326991E-03	-2.850276E-04	-7.335060E-04
24	C19	-1.692902E-04	6.245857E-06	-3.167455E-04
25	H16	3.328469E-04	1.496603E-04	2.129530E-04
26	H17	3.554518E-04	1.287242E-04	-1.716810E-04
27	H18	1.486137E-03	2.577115E-04	2.009026E-04
28	H19	1.714175E-05	8.756748E-05	-1.598401E-04
29	H20	3.361176E-05	4.889786E-05	-2.258726E-04
30	C1	-2.352300E-04	1.325569E-04	1.607766E-04
31	H5	-2.663504E-05	8.592286E-06	1.148176E-04
32	H6	-4.109229E-05	8.430998E-05	1.882357E-04
33	C3	-4.420181E-04	-1.741977E-04	-2.983395E-04
34	C26	-2.316693E-04	-2.309980E-04	-1.002232E-04
35	C27	-8.113392E-05	-3.491728E-05	-1.261326E-04
36	C28	2.378795E-04	1.576131E-04	7.887497E-05
37	C29	1.155601E-04	5.628839E-05	2.498595E-04
38	H1	-1.391838E-04	-3.540240E-05	1.268414E-04
39	H2	6.131107E-05	1.420514E-04	1.187152E-04

```
  40    H3      5.722184E-05   1.176396E-04  -1.489399E-04
  41    H9     -1.997639E-04  -2.093410E-04  -2.637846E-04
  42    H10     2.310080E-04   2.058152E-04  -3.046466E-04
  43    H25    -5.723237E-05   6.307406E-05  -1.828532E-05
 -----------   -------------  -------------  -------------
   total        6.569597E-04   8.467135E-04   2.658720E-05

  end of program der1b


  start of program geopt  14

  geometry optimization step 14
  reading input hessian of dimension   129
   in five columns format
  reading input hessian of dimension   129
   in five columns format
  reading input hessian of dimension   129
   in five columns format


  Level shifts adjusted to satisfy step-size constraints
   Step size:    0.0108550
   Cos(theta):   0.7082419

   Final level shift:  -3.0472439E-02

  energy change:          -7.4265E-06 *  (  5.0000E-05 )
  gradient maximum:        1.4699E-03 .  (  4.5000E-04 )
  gradient rms:            2.7737E-04 *  (  3.0000E-04 )
  step size:   0.01085  trust radius:   0.01000
  displacement maximum:    4.5549E-03 .  (  1.8000E-03 )
  displacement rms:        8.5285E-04 *  (  1.2000E-03 )
  predicted energy change:    -1.5366E-05    geom step:   1.0855E-
02    full step:   1.0855E-02
  molecular structure not yet converged...

  center of mass moved by:
     x:     2.3234E-04     y:    -3.9719E-04     z:     1.1836E-04

  new geometry:
                              angstroms
  atom            x                 y                 z
  N1       -0.2476098628      0.4319499494      1.1700896461
  N2       -1.0050850757     -2.2950871682      1.1949182325
  C4       -1.2225555532     -0.0359392069      1.9934979632
  C5       -0.0345337388     -1.9145591457      0.4863206158
  C6       -1.6345629750     -1.3576518558      2.0372932256
  C7       -1.7207638142      0.9827487410      2.7857085019
  C13       0.6372167911     -2.9214974519     -0.4226269266
  H4       -2.9991767135     -2.6746254046      2.9867461727
  C8       -2.7544283972      0.6541992122      3.6802520196
```

```
 C11      -0.9547075557      2.1487129569      2.4185234965
 H7       -3.1936858699      1.4000793918      4.3191782963
 C9       -3.1953417522     -0.6499826706      3.7301011813
 H8       -3.9846205275     -0.9126483273      4.4122075724
 C10      -2.6412076664     -1.6632555907      2.9184523073
 C12      -0.0714118196      1.7763193928      1.4539254438
 C2        0.9267126827      2.6085697080      0.7475086448
 H13       1.7145636018     -2.8715088035     -0.2838423517
 C14       0.3089199593     -2.7020981095     -1.8909152593
 H15       0.3081077662     -3.9039526830     -0.1083403583
 C15      -0.3215924809     -2.3201267580     -4.5836616469
 C16       1.3004540244     -2.3992437756     -2.8137797881
 C17      -1.0077278059     -2.8118948597     -2.3339058106
 C18      -1.3203617236     -2.6227459376     -3.6680394462
 C19       0.9888784831     -2.2093360982     -4.1534576988
 H16       2.3240667900     -2.3146888619     -2.4895233992
 H17      -1.7849137076     -3.0449393883     -1.6268919447
 H18      -2.3419771432     -2.7122708466     -3.9947783125
 H19       1.7707403444     -1.9764961643     -4.8551652860
 H20      -0.5656277175     -2.1736956496     -5.6211053749
 C1        0.5760456600     -0.5105838268      0.4433549677
 H5        1.5800429573     -0.5626136306      0.8641513090
 H6        0.6825437933     -0.2189447287     -0.5941242795
 C3        2.7951744578      4.2145617192     -0.5601377830
 C26       1.8187572576      3.3915699332      1.4744469707
 C27       0.9785171969      2.6447421378     -0.6446715605
 C28       1.9082206454      3.4396720163     -1.2930044553
 C29       2.7448249516      4.1917280945      0.8244421729
 H1        1.7902754511      3.3610162274      2.5486052296
 H2        0.2747696491      2.0686681731     -1.2192937084
 H3        1.9337718424      3.4605773143     -2.3682340620
 H9        3.4301063300      4.7894624992      1.3997519889
 H10       3.5171655374      4.8322369654     -1.0645143063
 H25      -1.0688078963      3.1463685995      2.7914697600

 nuclear repulsion energy.......  1971.109269844 hartrees
 ------------------------------------------------
/  end of geometry optimization iteration  14   /
------------------------------------------------

 end of program geopt


 start of program onee
 smallest eigenvalue of S:    2.820E-04
 number of canonical orbitals.....          461
 end of program onee


 start of program probe
 end of program probe
```

```
  start of program grid

  number of gridpoints:
     atom        N1     N2     C4     C5     C6     C7    C13
H4
  grid # 1       96    102     87     87     90     90     84
73
  grid # 2      103    112     95     95     99     98     92
118
  grid # 3      214    227    195    187    191    199    163
224
  grid # 4      389    414    322    320    323    347    299
224

  number of gridpoints:
     atom        C8    C11     H7     C9     H8    C10    C12
C2
  grid # 1       89     86     73     87     73     89     86
92
  grid # 2       97     94    118     97    118     97     94
100
  grid # 3      184    185    223    184    222    184    194
195
  grid # 4      329    331    226    331    223    329    317
341

  number of gridpoints:
     atom       H13    C14    H15    C15    C16    C17    C18
C19
  grid # 1       69     91     69     89     88     88     89
89
  grid # 2      110    100    110     97     96     96     97
96
  grid # 3      211    197    214    183    184    183    184
183
  grid # 4      214    340    213    327    327    327    327
327

  number of gridpoints:
     atom       H16    H17    H18    H19    H20     C1     H5
H6
  grid # 1       72     73     73     73     73     82     70
69
  grid # 2      114    115    118    118    118     88    110
104
  grid # 3      214    216    223    222    224    164    217
207
  grid # 4      214    214    224    223    224    293    217
205

  number of gridpoints:
```

```
     atom        C3    C26    C27    C28    C29     H1     H2
H3
  grid # 1       89     88     88     89     89     72     71
73
  grid # 2       97     95     96     97     97    115    114
118
  grid # 3      185    182    184    185    185    217    213
223
  grid # 4      328    328    329    328    328    217    212
224

  number of gridpoints:
     atom        H9    H10    H25  total
  grid # 1       73     73     72   3518
  grid # 2      118    118    115   4494
  grid # 3      223    224    220   8638
  grid # 4      224    224    224  12247

  end of program grid


  start of program rwr
  end of program rwr


  start of program scf

       i  u  d  i  g
       t  p  i  c  r                                RMS    maximum
       e  d  i  u  i                       energy  density   DIIS
       r  t  s  t  d     total energy      change  change    error

etot   1  N  N  1  U  -990.39080246980             1.4E-05  9.2E-04
etot   2  Y  Y  4  M  -990.39085334491  5.1E-05  6.4E-06  3.6E-04
etot   3  Y  Y  4  M  -990.39085990967  6.6E-06  2.0E-06  9.6E-05
etot   4  Y  N  4  M  -990.39085924579 -6.6E-07  0.0E+00  0.0E+00


 Energy components, in hartrees:
   (A)  Nuclear repulsion............  1971.10926984366
   (E)  Total one-electron terms..... -5260.48968808571
   (I)  Total two-electron terms.....  2298.98955899626
   (L)  Electronic energy............ -2961.50012908945  (E+I)
   (N)  Total energy.................  -990.39085924579  (A+L)

 SCFE: SCF energy: HF    -990.39085924579 hartrees   iterations:
4


 HOMO energy:    -0.26901
 LUMO energy:     0.09668
```

```
Orbital energies:
-15.60463  -15.56524  -11.29626  -11.28681  -11.27141  -11.25098
-11.25089  -11.24604  -11.24583  -11.23989  -11.23981  -11.23902
-11.23846  -11.23783  -11.23708  -11.23595  -11.23451  -11.23447
-11.23271  -11.23045  -11.22455  -11.22026  -11.21879  -11.21610
-11.21302   -1.32494   -1.24990   -1.16327   -1.16057   -1.13849
 -1.07446   -1.06705   -1.03929   -1.02190   -1.01890   -1.01702
 -0.97103   -0.95434   -0.93367   -0.86016   -0.83696   -0.83361
 -0.82905   -0.80707   -0.79550   -0.76999   -0.73262   -0.71741
 -0.70112   -0.69838   -0.66629   -0.65789   -0.64288   -0.63712
 -0.63384   -0.62367   -0.62072   -0.61582   -0.59621   -0.59233
 -0.59050   -0.58743   -0.57834   -0.55809   -0.55227   -0.54820
 -0.54435   -0.51743   -0.50724   -0.50258   -0.50021   -0.49593
 -0.49348   -0.49117   -0.48739   -0.48119   -0.42972   -0.40488
 -0.36749   -0.34336   -0.34123   -0.33760   -0.32929   -0.28245
 -0.26901    0.09668    0.12894    0.13711    0.13740    0.14856
  0.17846    0.20869    0.22827    0.23519    0.24116

end of program scf


start of program der1a
end of program der1a


start of program rwr
end of program rwr


start of program der1b

forces (hartrees/bohr) : total

atom  label         x              y              z
----  -----   -------------  -------------  -------------
  1    N1      3.594168E-04  -3.247900E-04  -4.707368E-04
  2    N2      1.456478E-04   1.299541E-04  -1.590589E-04
  3    C4      1.259234E-04  -1.305667E-04  -7.187211E-05
  4    C5     -3.678037E-05  -4.521296E-05   1.199478E-04
  5    C6      5.984738E-05  -1.658855E-04  -5.162803E-05
  6    C7     -6.725914E-05   2.139092E-04   6.314595E-05
  7    C13    -3.445068E-05   1.086910E-04   1.095968E-04
  8    H4      1.233470E-05  -3.123613E-05   3.656539E-05
  9    C8     -2.053567E-05   3.892458E-05   6.619510E-05
 10    C11    -3.836909E-05   2.069748E-04   1.279422E-04
 11    H7     -2.571961E-06   9.001533E-05   2.737180E-05
 12    C9      4.406467E-05  -4.942249E-05  -3.711664E-05
 13    H8     -1.489958E-05   3.325105E-06   5.832352E-05
 14    C10    -2.354330E-05   2.627673E-05   4.444407E-05
 15    C12     1.173535E-04   8.037980E-05  -1.135315E-05
 16    C2      3.023010E-05   1.143481E-04   1.144053E-04
 17    H13     4.746262E-05   8.375568E-05   4.709878E-06
```

```
 18    C14    -2.570906E-04  -7.682341E-05   2.962546E-05
 19    H15     4.472624E-05  -6.375860E-05  -2.023663E-05
 20    C15     1.891440E-04   6.801696E-05  -1.397037E-04
 21    C16     8.780006E-05   7.855990E-05  -3.361955E-05
 22    C17     7.344717E-05  -2.688328E-05   3.500123E-04
 23    C18    -2.375303E-04  -1.002662E-04  -2.346247E-05
 24    C19     3.475516E-05   4.269829E-05  -7.942340E-05
 25    H16    -1.546063E-05  -3.301204E-05  -2.106096E-05
 26    H17     6.240769E-05   4.272218E-05   5.193411E-05
 27    H18     2.079249E-04   6.398798E-05   5.979060E-05
 28    H19    -1.512320E-05  -2.486948E-05  -5.670209E-05
 29    H20     3.073742E-05   1.633282E-05  -8.591535E-05
 30    C1     -1.156034E-04   1.492051E-04   1.091582E-04
 31    H5     -1.311443E-04   5.783864E-05   4.118251E-05
 32    H6      4.583649E-05   3.741885E-05   1.991567E-04
 33    C3     -1.431507E-04  -3.099268E-05  -1.181485E-04
 34    C26     4.632667E-05   6.386637E-05  -1.655493E-04
 35    C27     1.736270E-05   4.420336E-05  -2.361738E-04
 36    C28     3.924001E-05   3.200689E-05   1.369748E-04
 37    C29     1.549265E-05   1.514235E-05   4.802569E-05
 38    H1     -3.310335E-05   2.030411E-05   1.784773E-04
 39    H2      9.751055E-05   8.306954E-05   8.534773E-05
 40    H3      2.527376E-05   2.668486E-05  -9.347572E-05
 41    H9     -9.749903E-05  -4.736787E-05  -9.930280E-05
 42    H10    -2.819321E-06   2.249804E-05  -7.816079E-05
 43    H25    -1.728472E-05   5.798267E-05   1.449746E-05
-----------   -------------  -------------  -------------
   total       6.560473E-04   8.680061E-04   2.412989E-05

 end of program der1b


 start of program geopt  15

 geometry optimization step 15
 reading input hessian of dimension   129
  in five columns format
 reading input hessian of dimension   129
  in five columns format



 Level shifts adjusted to satisfy step-size constraints
  Step size:    0.0143612
  Cos(theta):   0.4919231

  Final level shift:  -2.7000810E-02

 energy change:          -1.0263E-05 *  (  5.0000E-05 )
 gradient maximum:        4.0569E-04 *  (  4.5000E-04 )
 gradient rms:            1.0457E-04 *  (  3.0000E-04 )
 step size:   0.01436  trust radius:   0.01414
 displacement maximum:    8.4903E-03 .  (  1.8000E-03 )
```

```
  displacement rms:        1.1283E-03 *  (  1.2000E-03 )
  predicted energy change:    -7.4859E-06    geom step:   1.4361E-
02    full step:   1.4361E-02
  molecular structure not yet converged...

  center of mass moved by:
     x:     2.6501E-05     y:    -2.4923E-04     z:     6.1267E-05

  new geometry:
                              angstroms
  atom           x                 y                 z
  N1       -0.2463607370      0.4314193906      1.1710338958
  N2       -1.0035424221     -2.2955233045      1.1947806590
  C4       -1.2216235933     -0.0368540479      1.9940347638
  C5       -0.0326064789     -1.9145180546      0.4866450499
  C6       -1.6331040260     -1.3588415492      2.0376289344
  C7       -1.7214275643      0.9821252034      2.7857401080
  C13       0.6387952323     -2.9207111820     -0.4230303496
  H4       -2.9983933254     -2.6759482577      2.9865390107
  C8       -2.7554322781      0.6531437848      3.6795071530
  C11      -0.9556687926      2.1484771315      2.4194464699
  H7       -3.1957362911      1.3993275357      4.3177668929
  C9       -3.1954005477     -0.6509580953      3.7294178623
  H8       -3.9851392350     -0.9139827639      4.4112945459
  C10      -2.6402354285     -1.6642884757      2.9184542048
  C12      -0.0716231059      1.7762215315      1.4549835704
  C2        0.9257296648      2.6089482628      0.7484190091
  H13       1.7160363204     -2.8699892414     -0.2843745120
  C14       0.3092857777     -2.7015111760     -1.8910420198
  H15       0.3107659365     -3.9038972276     -0.1098048464
  C15      -0.3210182211     -2.3198001555     -4.5845563047
  C16       1.3026267989     -2.4078132613     -2.8158087619
  C17      -1.0093417649     -2.8017601438     -2.3324540938
  C18      -1.3215507168     -2.6128445964     -3.6669555125
  C19       0.9912426335     -2.2182211833     -4.1556558124
  H16       2.3277109198     -2.3309831513     -2.4925549566
  H17      -1.7872243266     -3.0262248434     -1.6238607485
  H18      -2.3426567575     -2.6932803270     -3.9926346211
  H19       1.7741739528     -1.9932912445     -4.8591528825
  H20      -0.5645529054     -2.1734405864     -5.6223463359
  C1        0.5782555737     -0.5107568800      0.4460053859
  H5        1.5811573473     -0.5631828239      0.8704649311
  H6        0.6880840404     -0.2177615274     -0.5909622482
  C3        2.7922215595      4.2150771119     -0.5617697159
  C26       1.8178585798      3.3922403513      1.4746958238
  C27       0.9762379671      2.6449102789     -0.6443115909
  C28       1.9050145102      3.4399273512     -1.2938231691
  C29       2.7430458157      4.1923393067      0.8232302794
  H1        1.7893807507      3.3619023049      2.5491513072
  H2        0.2718921784      2.0685313456     -1.2175644237
  H3        1.9300026211      3.4609305987     -2.3692287604
  H9        3.4287854490      4.7902046058      1.3973483110
```

```
  H10       3.5140165769      4.8329081285     -1.0672737690
  H25      -1.0711083010      3.1462171349      2.7921150838

  nuclear repulsion energy.......  1971.034696625 hartrees
  ------------------------------------------------
 /  end of geometry optimization iteration  15   /
 ------------------------------------------------

  end of program geopt


  start of program onee
  smallest eigenvalue of S:    2.821E-04
  number of canonical orbitals.....          461
  end of program onee


  start of program probe
  end of program probe


  start of program grid

  number of gridpoints:
     atom        N1     N2     C4     C5     C6     C7    C13
H4
  grid # 1       96    102     87     87     90     89     84
73
  grid # 2      103    112     95     95     99     98     92
118
  grid # 3      214    227    195    187    191    199    163
224
  grid # 4      388    414    321    320    322    348    299
224

  number of gridpoints:
     atom        C8    C11     H7     C9     H8    C10    C12
C2
  grid # 1       89     86     73     87     73     89     86
92
  grid # 2       97     94    118     97    118     97     94
100
  grid # 3      184    185    223    184    222    184    194
195
  grid # 4      328    331    226    331    223    329    317
343

  number of gridpoints:
     atom       H13    C14    H15    C15    C16    C17    C18
C19
  grid # 1       69     91     69     89     88     88     89
89
```

```
  grid # 2      109    100    110     97     96     96     97
96
  grid # 3      210    196    214    183    184    183    184
183
  grid # 4      214    340    213    327    327    327    327
327

  number of gridpoints:
     atom        H16    H17    H18    H19    H20     C1     H5
H6
  grid # 1       72     73     73     73     73     82     70
69
  grid # 2      114    115    118    118    118     88    109
104
  grid # 3      214    216    222    222    224    164    217
207
  grid # 4      214    214    224    223    224    293    219
205

  number of gridpoints:
     atom         C3    C26    C27    C28    C29     H1     H2
H3
  grid # 1       89     88     88     89     89     72     71
73
  grid # 2       97     95     96     97     97    115    114
118
  grid # 3      185    182    183    185    185    217    213
223
  grid # 4      328    327    329    327    328    217    212
224

  number of gridpoints:
     atom         H9    H10    H25  total
  grid # 1       73     73     72   3517
  grid # 2      118    118    115   4492
  grid # 3      222    224    220   8633
  grid # 4      224    224    224  12246

  end of program grid


  start of program rwr
  end of program rwr


  start of program scf

       i  u  d  i  g
       t  p  i  c  r                                RMS    maximum
       e  d  i  u  i                      energy  density   DIIS
       r  t  s  t  d     total energy     change  change    error
```

```
etot   1  N  N  1  U  -990.39072743546            2.7E-05  1.6E-03
etot   2  Y  Y  4  M  -990.39084046479  1.1E-04  1.0E-05  6.3E-04
etot   3  Y  Y  4  M  -990.39085660547  1.6E-05  2.9E-06  1.5E-04
etot   4  Y  N  4  M  -990.39085842189  1.8E-06  0.0E+00  0.0E+00


 Energy components, in hartrees:
   (A)  Nuclear repulsion............  1971.03469662474
   (E)  Total one-electron terms..... -5260.33898399291
   (I)  Total two-electron terms.....  2298.91342894628
   (L)  Electronic energy............ -2961.42555504663  (E+I)
   (N)  Total energy.................  -990.39085842189  (A+L)

 SCFE: SCF energy: HF    -990.39085842189 hartrees   iterations:
4


 HOMO energy:    -0.26904
 LUMO energy:     0.09663

  Orbital energies:
  -15.60476  -15.56511  -11.29628  -11.28688  -11.27173  -11.25112
  -11.25088  -11.24616  -11.24601  -11.23993  -11.23979  -11.23905
  -11.23848  -11.23784  -11.23711  -11.23607  -11.23466  -11.23444
  -11.23292  -11.23053  -11.22458  -11.22023  -11.21899  -11.21606
  -11.21326   -1.32501   -1.24984   -1.16321   -1.16033   -1.13852
   -1.07453   -1.06701   -1.03926   -1.02190   -1.01887   -1.01693
   -0.97093   -0.95430   -0.93367   -0.86015   -0.83703   -0.83361
   -0.82898   -0.80705   -0.79545   -0.77000   -0.73261   -0.71741
   -0.70108   -0.69829   -0.66635   -0.65790   -0.64282   -0.63719
   -0.63386   -0.62365   -0.62070   -0.61581   -0.59629   -0.59238
   -0.59044   -0.58747   -0.57825   -0.55807   -0.55220   -0.54819
   -0.54450   -0.51739   -0.50722   -0.50256   -0.50019   -0.49584
   -0.49341   -0.49117   -0.48733   -0.48121   -0.42975   -0.40483
   -0.36754   -0.34337   -0.34122   -0.33756   -0.32914   -0.28243
   -0.26904    0.09663    0.12889    0.13707    0.13733    0.14845
    0.17836    0.20861    0.22838    0.23520    0.24116

  end of program scf


  start of program der1a
  end of program der1a


  start of program rwr
  end of program rwr


  start of program der1b

  forces (hartrees/bohr) : total
```

atom	label	x	y	z
1	N1	-2.481303E-04	2.161363E-04	3.038174E-05
2	N2	2.836382E-04	-2.499438E-05	-2.728610E-04
3	C4	-5.332666E-05	1.576006E-04	1.308833E-04
4	C5	-3.595149E-04	-1.387013E-04	2.963041E-04
5	C6	2.019594E-05	1.908855E-04	2.396059E-05
6	C7	2.466402E-04	-1.423657E-04	-1.552636E-04
7	C13	-1.276988E-04	5.612039E-06	6.448695E-05
8	H4	8.439654E-05	1.557089E-04	3.881350E-05
9	C8	1.165608E-05	5.058858E-04	1.509718E-04
10	C11	1.471085E-04	-4.822498E-05	-3.124871E-04
11	H7	5.901722E-05	-5.856555E-05	-6.083047E-05
12	C9	-2.081620E-04	-4.628287E-04	1.114154E-04
13	H8	1.113241E-04	3.132659E-05	-7.909427E-05
14	C10	-1.274255E-04	-2.859290E-04	1.870702E-05
15	C12	-5.235787E-06	8.359514E-05	2.010785E-04
16	C2	2.011226E-05	1.154169E-04	-3.679946E-04
17	H13	1.231949E-04	6.717329E-05	-9.936772E-05
18	C14	-2.239323E-04	1.957682E-04	-5.369846E-04
19	H15	3.610993E-06	-2.367943E-05	1.405502E-04
20	C15	4.528455E-04	8.163430E-06	3.393146E-04
21	C16	-5.301138E-05	-4.748777E-05	2.288187E-04
22	C17	9.188483E-04	1.208919E-04	1.826117E-04
23	C18	1.202191E-03	1.978553E-04	5.774334E-05
24	C19	-4.165740E-04	-3.910772E-05	-2.787547E-04
25	H16	-3.998587E-04	2.166071E-05	-1.106724E-04
26	H17	-1.196398E-04	-1.085782E-04	1.297513E-04
27	H18	-9.010150E-04	-1.633065E-04	-2.194062E-04
28	H19	-1.346353E-04	2.261628E-05	1.123854E-04
29	H20	4.154382E-06	-5.819682E-06	1.013341E-04
30	C1	5.219134E-04	1.699161E-04	-4.100770E-06
31	H5	-3.070221E-04	-1.084423E-05	-1.892583E-04
32	H6	6.403176E-05	-1.027693E-04	3.230711E-04
33	C3	1.449365E-04	1.660298E-04	1.228439E-04
34	C26	-3.787139E-07	5.421162E-05	-1.973404E-04
35	C27	1.942710E-05	4.496688E-05	2.562209E-04
36	C28	-4.419795E-05	-3.799500E-05	5.523642E-05
37	C29	-1.078932E-06	-5.617927E-06	-2.284443E-04
38	H1	8.125850E-05	7.200215E-05	-6.136742E-05
39	H2	4.595660E-05	3.363527E-05	-2.715897E-05
40	H3	-3.156831E-05	-9.218442E-06	3.647842E-06
41	H9	3.691536E-05	8.898917E-05	2.667129E-05
42	H10	-2.160082E-04	-1.433661E-04	1.416099E-04
43	H25	3.817919E-05	-4.036847E-05	-5.326570E-05
total		6.631381E-04	8.262798E-04	3.416091E-05

end of program der1b

```
start of program geopt  16

geometry optimization step 16
reading input hessian of dimension   129
 in five columns format
reading input hessian of dimension   129
 in five columns format
** restarting optimization from step   15 **


Level shifts adjusted to satisfy step-size constraints
 Step size:    0.0104342
 Cos(theta):   0.4965050

 Final level shift:  -2.6262214E-02

energy change:           8.2390E-07 #  (  5.0000E-05 )
gradient maximum:        4.0569E-04 *  (  4.5000E-04 )
gradient rms:            1.0457E-04 *  (  3.0000E-04 )
step size:   0.01043  trust radius:   0.01000
displacement maximum:    6.0223E-03 .  (  1.8000E-03 )
displacement rms:        8.1979E-04 *  (  1.2000E-03 )
predicted energy change:    -4.8790E-06    geom step:   1.0434E-
02    full step:   1.0434E-02

********************************************************
**            Geometry optimization complete          **
********************************************************

center of mass moved by:
   x:     0.0000E+00     y:    -6.8695E-16     z:     3.8858E-16

final geometry:
                              angstroms
atom           x                  y                  z
N1       -0.2476098628      0.4319499494      1.1700896461
N2       -1.0050850757     -2.2950871682      1.1949182325
C4       -1.2225555532     -0.0359392069      1.9934979632
C5       -0.0345337388     -1.9145591457      0.4863206158
C6       -1.6345629750     -1.3576518558      2.0372932256
C7       -1.7207638142      0.9827487410      2.7857085019
C13       0.6372167911     -2.9214974519     -0.4226269266
H4       -2.9991767135     -2.6746254046      2.9867461727
C8       -2.7544283972      0.6541992122      3.6802520196
C11      -0.9547075557      2.1487129569      2.4185234965
H7       -3.1936858699      1.4000793918      4.3191782963
C9       -3.1953417522     -0.6499826706      3.7301011813
H8       -3.9846205275     -0.9126483273      4.4122075724
C10      -2.6412076664     -1.6632555907      2.9184523073
C12      -0.0714118196      1.7763193928      1.4539254438
C2        0.9267126827      2.6085697080      0.7475086448
H13       1.7145636018     -2.8715088035     -0.2838423517
```

```
 C14       0.3089199593     -2.7020981095     -1.8909152593
 H15       0.3081077662     -3.9039526830     -0.1083403583
 C15      -0.3215924809     -2.3201267580     -4.5836616469
 C16       1.3004540244     -2.3992437756     -2.8137797881
 C17      -1.0077278059     -2.8118948597     -2.3339058106
 C18      -1.3203617236     -2.6227459376     -3.6680394462
 C19       0.9888784831     -2.2093360982     -4.1534576988
 H16       2.3240667900     -2.3146888619     -2.4895233992
 H17      -1.7849137076     -3.0449393883     -1.6268919447
 H18      -2.3419771432     -2.7122708466     -3.9947783125
 H19       1.7707403444     -1.9764961643     -4.8551652860
 H20      -0.5656277175     -2.1736956496     -5.6211053749
 C1        0.5760456600     -0.5105838268      0.4433549677
 H5        1.5800429573     -0.5626136306      0.8641513090
 H6        0.6825437933     -0.2189447287     -0.5941242795
 C3        2.7951744578      4.2145617192     -0.5601377830
 C26       1.8187572576      3.3915699332      1.4744469707
 C27       0.9785171969      2.6447421378     -0.6446715605
 C28       1.9082206454      3.4396720163     -1.2930044553
 C29       2.7448249516      4.1917280945      0.8244421729
 H1        1.7902754511      3.3610162274      2.5486052296
 H2        0.2747696491      2.0686681731     -1.2192937084
 H3        1.9337718424      3.4605773143     -2.3682340620
 H9        3.4301063300      4.7894624992      1.3997519889
 H10       3.5171655374      4.8322369654     -1.0645143063
 H25      -1.0688078963      3.1463685995      2.7914697600

 nuclear repulsion energy.......  1971.109269844 hartrees
 --------------------------------------------------
/  end of geometry optimization iteration  16   /
--------------------------------------------------

 end of program geopt


 start of program post
 Writing a SPARTAN archive file
 end of program post

Total cpu seconds     user:    1761.812   user+sys:    1761.812
```

Compound 2'a

```
+-----------------------------------------------------------------
---+
  |  Jaguar version 3.5, release  42
|
  |
|
  |  Copyright 1991-1998 Schrodinger, Inc.
|
  |  All Rights Reserved.
|
  |
|
  |  Use of this program should be acknowledged in publications
as:    |
  |  Jaguar 3.5, Schrodinger, Inc., Portland, Oregon, 1998.
|
  +-----------------------------------------------------------------
-----+

  start of program pre
  Job name: WF3654
  Executables used: D:\TITAN
  Temporary files : C:\Users\gfabr\AppData\Local\Temp\WF3654

  Input file comments:
    Molecule001
    This file created by Spartan

  basis set:             6-31G**
  net molecular charge:    0
  multiplicity:            1

  number of basis functions....          465

 Input geometry:
                              angstroms
  atom           x                 y                 z
  N1       -0.1550260000      0.4374990000      1.1210390000
  N2       -1.0086730000     -2.3256400000      1.1332130000
  C4       -1.1860510000     -0.0350800000      1.9315790000
  C5       -0.0104550000     -1.9237530000      0.4028750000
  C6       -1.6456290000     -1.3760560000      1.9632780000
  C7       -1.7070180000      1.0262900000      2.7388420000
  C13       0.7199200000     -2.8803430000     -0.5063790000
  H4       -3.1185930000     -2.6496450000      2.9223950000
  C8       -2.7656460000      0.7093510000      3.5969080000
  C11      -0.9132890000      2.1852800000      2.3994340000
  H7       -3.2133600000      1.4833470000      4.2359600000
  C9       -3.2381490000     -0.5972350000      3.6279590000
```

```
H8       -4.0721010000     -0.8455450000      4.3029390000
C10      -2.7017240000     -1.6349910000      2.8394980000
C12       0.0268720000      1.8011420000      1.4273890000
C2        1.0263770000      2.6560040000      0.8075810000
H13       1.8204060000     -2.6663390000     -0.4286580000
C14       0.2610050000     -2.7449900000     -1.9177470000
H15       0.5612370000     -3.9425620000     -0.1705390000
C15      -0.5895550000     -2.5433530000     -4.5760680000
C16       1.1246910000     -2.2456410000     -2.8988340000
C17      -1.0320590000     -3.1444790000     -2.2771670000
C18      -1.4542200000     -3.0419090000     -3.6012120000
C19       0.6998040000     -2.1465450000     -4.2231290000
H16       2.1442750000     -1.9343460000     -2.6271240000
H17      -1.7134540000     -3.5365780000     -1.5059970000
H18      -2.4719630000     -3.3556810000     -3.8767030000
H19       1.3846780000     -1.7555640000     -4.9897570000
H20      -0.9239070000     -2.4642400000     -5.6207450000
C1        0.5776390000     -0.5062530000      0.3222060000
H5        1.6528730000     -0.5444960000      0.6633050000
H6        0.5619750000     -0.1829560000     -0.7593680000
C3        2.9246780000      4.3652120000     -0.3378590000
C26       1.7455110000      3.5549090000      1.6101580000
C27       1.2625130000      2.6251890000     -0.5732690000
C28       2.2088580000      3.4770190000     -1.1402160000
C29       2.6895760000      4.4036320000      1.0366540000
H1        1.5580180000      3.5843390000      2.6943120000
H2        0.6891520000      1.9402470000     -1.2156630000
H3        2.3868000000      3.4502800000     -2.2254160000
H9        3.2505630000      5.1053430000      1.6714530000
H10       3.6710440000      5.0358360000     -0.7881560000
H25      -1.0275920000      3.1833010000      2.8110280000

Molecular weight:    322.15 amu

Stoichiometry:        C23N2H18
Molecular Point Group: C1
Point Group used: C1
nuclear repulsion energy.......  1952.777623249 hartrees

Non-default options chosen:
  Geometry will be optimized in redundant internal coordinates
  Initial Hessian: from previous calculation

end of program pre


start of program onee
smallest eigenvalue of S:    3.218E-04
number of canonical orbitals.....          462
end of program onee
```

```
  start of program hfig
  initial wavefunction generated automatically from atomic
wavefunctions

  Irreducible     Total no   No of occupied orbitals
  representation  orbitals   Shell_1  Shell_2    ...
  No Symm          462         85
  ------------------------
  Orbital occupation/shell    1.000

  end of program hfig


  start of program probe
  end of program probe


  start of program grid

  number of gridpoints:
     atom        N1     N2      C4      C5      C6      C7     C13
H4
  grid # 1       97    102      88      88      88      90      84
73
  grid # 2      104    112      97      95      98      98      92
118
  grid # 3      214    229     199     184     197     196     170
223
  grid # 4      391    414     331     322     327     347     304
232

  number of gridpoints:
     atom        C8    C11      H7      C9      H8     C10     C12
C2
  grid # 1       89     87      73      87      73      89      86
90
  grid # 2       97     95     118      97     118      97      97
98
  grid # 3      186    182     224     184     222     186     191
195
  grid # 4      332    341     234     329     231     332     331
341

  number of gridpoints:
     atom       H13    C14     H15     C15     C16     C17     C18
C19
  grid # 1       69     93      69      89      88      89      89
89
  grid # 2      110    100     112      97      96      96      97
97
  grid # 3      210    195     216     187     186     185     186
185
```

```
  grid # 4      215    342    223    331    330    331    331
330

  number of gridpoints:
     atom       H16    H17    H18    H19    H20     C1     H5
H6
  grid # 1       72     72     73     73     73     82     70
69
  grid # 2      112    114    118    118    118     90    112
102
  grid # 3      214    217    224    223    224    167    216
205
  grid # 4      221    223    232    231    232    299    217
206

  number of gridpoints:
     atom        C3    C26    C27    C28    C29     H1     H2
H3
  grid # 1       89     89     88     89     89     72     71
73
  grid # 2       97     96     96     97     97    115    111
118
  grid # 3      187    184    186    186    186    218    212
224
  grid # 4      332    330    330    329    330    224    217
232

  number of gridpoints:
     atom        H9    H10    H25  total
  grid # 1       73     73     72   3521
  grid # 2      118    118    115   4498
  grid # 3      224    224    219   8672
  grid # 4      232    232    222  12443

  end of program grid


  start of program rwr
  end of program rwr


  start of program scf
 number of electrons..........        170
 number of alpha electrons....         85
 number of beta electrons.....         85
 number of orbitals, total....        462
 number of core orbitals......         85
 number of open shell orbs....          0
 number of occupied orbitals..         85
 number of virtual orbitals...        377
 number of hamiltonians.......          1
 number of shells.............          1
```

```
 SCF type: HF

       i  u  d  i  g
       t  p  i  c  r                                          RMS    maximum
       e  d  i  u  i                              energy  density   DIIS
       r  t  s  t  d     total energy     change  change    error

etot   1  N  N  5  M  -987.21080013367             3.9E-03  9.8E-02
etot   2  Y  Y  6  M  -989.98476104696  2.8E+00  1.7E-03  5.5E-02
etot   3  Y  Y  6  M  -990.27640419982  2.9E-01  7.8E-04  2.7E-02
etot   4  N  Y  2  U  -990.34536478481  6.9E-02  3.8E-04  1.5E-02
etot   5  Y  Y  6  M  -990.35620931742  1.1E-02  1.0E-03  1.0E-02
etot   6  N  Y  2  U  -990.36578188544  9.6E-03  1.2E-04  2.2E-03
etot   7  Y  Y  6  M  -990.36633784606  5.6E-04  4.0E-05  5.6E-04
etot   8  Y  Y  6  M  -990.36641184175  7.4E-05  1.2E-05  2.3E-04
etot   9  N  Y  2  U  -990.36623705176 -1.7E-04  5.8E-06  1.1E-04
etot  10  Y  Y  6  M  -990.36624305181  6.0E-06  4.9E-06  5.8E-05
etot  11  Y  N  6  M  -990.36624587679  2.8E-06  0.0E+00  0.0E+00


 Energy components, in hartrees:
   (A)  Nuclear repulsion............  1952.77762324898
   (E)  Total one-electron terms..... -5223.87627037117
   (I)  Total two-electron terms.....  2280.73240124540
   (L)  Electronic energy............ -2943.14386912577  (E+I)
   (N)  Total energy.................  -990.36624587679  (A+L)

 SCFE: SCF energy: HF    -990.36624587679 hartrees   iterations:
11


 HOMO energy:    -0.26238
 LUMO energy:     0.08458

  Orbital energies:
  -15.61225  -15.57394  -11.30699  -11.29988  -11.28257  -11.26176
  -11.25976  -11.25489  -11.25065  -11.24883  -11.24706  -11.24570
  -11.24527  -11.24438  -11.24359  -11.24267  -11.24138  -11.24124
  -11.23937  -11.23733  -11.22956  -11.22835  -11.22767  -11.22333
  -11.22233   -1.30891   -1.23521   -1.15989   -1.15811   -1.13132
   -1.07044   -1.06879   -1.03483   -1.01840   -1.01489   -1.01367
   -0.96165   -0.95090   -0.92391   -0.85859   -0.83575   -0.83045
   -0.82627   -0.79990   -0.78600   -0.76574   -0.72486   -0.71505
   -0.69233   -0.69090   -0.66232   -0.65281   -0.64039   -0.63511
   -0.63125   -0.61793   -0.61598   -0.61045   -0.59401   -0.58832
   -0.58652   -0.58354   -0.57683   -0.55347   -0.54722   -0.54082
   -0.53565   -0.51410   -0.50599   -0.49981   -0.49872   -0.49409
   -0.49103   -0.48832   -0.48378   -0.47850   -0.42632   -0.40843
   -0.36790   -0.34360   -0.34155   -0.33783   -0.32751   -0.27573
   -0.26238    0.08458    0.11895    0.13358    0.13414    0.14570
    0.17403    0.20148    0.21421    0.22686    0.23450
```

```
 end of program scf


 start of program der1a
 end of program der1a


 start of program rwr
recomputing RwR matrix   2    grid:  4
 end of program rwr


 start of program der1b

 forces (hartrees/bohr) : total

atom  label          x              y              z
----  -----   -------------  -------------  -------------
  1    N1     -3.240872E-02   9.749688E-03   3.179943E-02
  2    N2      3.994841E-02   1.589784E-02  -2.744144E-02
  3    C4     -4.648588E-03   1.109702E-02   6.253104E-03
  4    C5     -3.592959E-02  -8.241299E-03   3.439027E-02
  5    C6      2.414258E-03   2.916011E-02   4.891493E-03
  6    C7      2.172547E-02  -2.498377E-02  -2.655680E-02
  7    C13     1.776470E-02  -2.489095E-02   2.423915E-02
  8    H4      8.099431E-03   1.571016E-02  -2.748483E-03
  9    C8     -8.989727E-03   2.813047E-03   8.737148E-03
 10    C11     2.073446E-02  -1.403474E-02  -2.135011E-02
 11    H7      5.617432E-03  -1.253712E-02  -8.781297E-03
 12    C9     -1.066947E-02   1.935235E-03   1.014118E-02
 13    H8      1.338669E-02   4.465009E-03  -1.056642E-02
 14    C10     7.534698E-03  -9.961889E-03  -9.389467E-03
 15    C12    -4.116184E-02  -1.291056E-02   3.163420E-02
 16    C2      1.304881E-02   8.436034E-03  -7.725578E-03
 17    H13    -2.288695E-02  -5.875229E-03  -2.856836E-03
 18    C14    -3.001289E-05   9.115626E-04  -2.375293E-02
 19    H15     2.454104E-03   2.667852E-02  -3.093814E-03
 20    C15    -8.755934E-04   1.284549E-03  -8.246274E-03
 21    C16     1.904926E-03   8.743392E-04  -2.548369E-04
 22    C17    -5.241454E-03  -1.814770E-03   2.230640E-03
 23    C18    -7.258278E-03  -2.346623E-03  -2.041341E-04
 24    C19     3.649640E-03   3.130199E-03  -7.320720E-03
 25    H16    -1.529594E-02  -5.126618E-03  -3.849080E-03
 26    H17     1.141142E-02   6.616599E-03  -1.234182E-02
 27    H18     1.586235E-02   4.779575E-03   4.278800E-03
 28    H19    -1.052798E-02  -5.951830E-03   1.156894E-02
 29    H20     5.229882E-03  -1.242672E-03   1.606278E-02
 30    C1      2.113894E-02   9.772674E-03  -1.632786E-02
 31    H5     -2.245682E-02  -6.894493E-04  -1.106135E-02
 32    H6      4.269849E-03  -1.216673E-02   2.603804E-02
 33    C3      5.645640E-03   4.899727E-03  -3.657363E-03
 34    C26     4.904907E-04   2.576969E-03   4.717966E-03
```

```
  35    C27    -4.000485E-04  -2.665549E-03  -5.344084E-03
  36    C28     1.194079E-03  -8.729806E-04  -7.925486E-03
  37    C29     2.773262E-03   5.466727E-03   5.063842E-03
  38    H1      3.964567E-03  -1.650721E-03  -1.828743E-02
  39    H2      8.982905E-03   1.280343E-02   1.060548E-02
  40    H3     -2.910655E-03   4.951888E-04   1.678907E-02
  41    H9     -8.627735E-03  -1.102210E-02  -9.843115E-03
  42    H10    -1.152274E-02  -1.049380E-02   6.921504E-03
  43    H25     2.708121E-03  -9.050340E-03  -6.693826E-03
 -----------   -------------  -------------  -------------
    total       1.123978E-04   1.024489E-03   7.424633E-04

  end of program der1b


  start of program geopt   1

  geometry optimization step  1
  reading input hessian of dimension   129
   in five columns format


  Level shifts adjusted to satisfy step-size constraints
   Step size:    0.3000165
   Cos(theta):   0.8700386

   Final level shift:  -9.0459602E-02

  gradient maximum:        5.6400E-02 .  (  4.5000E-04 )
  gradient rms:            1.1269E-02 .  (  3.0000E-04 )
  step size:   0.30001  trust radius:   0.30000
  displacement maximum:    7.9070E-02 .  (  1.8000E-03 )
  displacement rms:        2.3571E-02 .  (  1.2000E-03 )
  predicted energy change:    -2.2790E-02    geom step:   3.0001E-
01    full step:   3.0001E-01
  molecular structure not yet converged...

  center of mass moved by:
     x:     1.7900E-03     y:     7.9249E-03     z:     5.2280E-03

  new geometry:
                              angstroms
  atom           x                 y                 z
  N1       -0.1851238607      0.4641584805      1.1496398783
  N2       -0.9624306185     -2.2803275995      1.1391335864
  C4       -1.1923369589     -0.0180925749      1.9481977188
  C5        0.0003838455     -1.8866472314      0.4304247849
  C6       -1.6131242204     -1.3444054000      1.9677332987
  C7       -1.7143322129      1.0107834641      2.7508560527
  C13       0.6888484121     -2.8743700008     -0.4705903976
  H4       -3.0326107871     -2.6499595048      2.8909113274
  C8       -2.7758076385      0.6768625265      3.6172856202
```

```
C11      -0.9256685288      2.1808482739      2.4168546928
H7       -3.2270829119      1.4262828740      4.2504106118
C9       -3.2256779072     -0.6251283660      3.6377251029
H8       -4.0393570567     -0.8900121801      4.2984882981
C10      -2.6561623240     -1.6393208618      2.8310281808
C12      -0.0161416855      1.8156812458      1.4563175807
C2        1.0081011308      2.6662618384      0.8151804166
H13       1.7673251388     -2.7356321765     -0.3960304160
C14       0.2466641767     -2.7462605278     -1.9265734159
H15       0.4459377797     -3.8754027525     -0.1219375680
C15      -0.5453795077     -2.5531842005     -4.6103164906
C16       1.1228101928     -2.2821169158     -2.9004344929
C17      -1.0389296276     -3.1210357258     -2.3122887225
C18      -1.4308521122     -3.0220378034     -3.6483671644
C19       0.7311661555     -2.1855070811     -4.2360240634
H16       2.1179862862     -2.0041675136     -2.6156208078
H17      -1.7271848709     -3.4681454408     -1.5658608400
H18      -2.4200252396     -3.2868875229     -3.9394618092
H19       1.4207969829     -1.8273231853     -4.9765738859
H20      -0.8415814914     -2.4643443665     -5.6434658446
C1        0.5813167973     -0.4636626778      0.3433044617
H5        1.6400268303     -0.4816687726      0.6523424559
H6        0.5606286448     -0.1702832088     -0.7202931642
C3        2.9112386307      4.3243859975     -0.3688081175
C26       1.7641520157      3.5436729388      1.5963361301
C27       1.2174113891      2.6356426600     -0.5675864813
C28       2.1639530001      3.4567854144     -1.1533354185
C29       2.7066639823      4.3656913408      1.0056564678
H1        1.6123726912      3.5626682239      2.6583667114
H2        0.6171678014      1.9866834111     -1.1807727574
H3        2.3092217370      3.4311451005     -2.2201183904
H9        3.2922168597      5.0359612586      1.6129911517
H10       3.6489292197      4.9615184331     -0.8293143894
H25      -1.0462406649      3.1705625677      2.8156287502

nuclear repulsion energy.......  1960.025589350 hartrees
 -------------------------------------------------
/  end of geometry optimization iteration   1   /
-------------------------------------------------

end of program geopt


start of program onee
smallest eigenvalue of S:    2.977E-04
number of canonical orbitals.....          461
end of program onee


start of program probe
end of program probe
```

```
  start of program grid

  number of gridpoints:
     atom        N1     N2      C4      C5      C6      C7     C13
H4
  grid # 1        97    102      87      88      88      92      84
73
  grid # 2       103    112      95      95      96     101      92
118
  grid # 3       214    227     197     186     193     197     164
223
  grid # 4       393    413     326     321     328     351     300
224

  number of gridpoints:
     atom        C8    C11      H7      C9      H8     C10     C12
C2
  grid # 1        89     87      73      87      73      89      86
93
  grid # 2        97     94     118      97     118      97      96
100
  grid # 3       184    184     223     184     222     185     195
197
  grid # 4       328    336     226     331     224     332     319
344

  number of gridpoints:
     atom       H13    C14     H15     C15     C16     C17     C18
C19
  grid # 1        69     93      69      89      88      89      89
89
  grid # 2       109    100     109      97      96      96      97
97
  grid # 3       211    195     214     184     184     184     186
182
  grid # 4       216    340     216     328     328     330     331
328

  number of gridpoints:
     atom       H16    H17     H18     H19     H20      C1      H5
H6
  grid # 1        72     72      73      73      73      82      72
69
  grid # 2       112    115     118     118     118      90     112
102
  grid # 3       213    216     223     222     223     166     217
205
  grid # 4       214    215     224     222     223     294     219
207

  number of gridpoints:
```

```
     atom         C3     C26     C27     C28     C29      H1      H2
H3
  grid # 1        89      87      87      89      89      72      70
73
  grid # 2        97      97      96      97      97     115     111
118
  grid # 3       185     181     183     184     184     218     213
222
  grid # 4       329     328     330     328     328     217     210
224

  number of gridpoints:
     atom         H9     H10     H25  total
  grid # 1        73      73      72   3523
  grid # 2       118     118     115   4494
  grid # 3       223     223     219   8635
  grid # 4       224     224     222  12295

  end of program grid


  start of program rwr
  end of program rwr


  start of program scf

       i  u  d  i  g
       t  p  i  c  r                                      RMS    maximum
       e  d  i  u  i                           energy  density    DIIS
       r  t  s  t  d     total energy          change  change     error

etot   1  N  N  2  U  -990.37561875819                 2.2E-04  5.2E-03
etot   2  Y  Y  6  M  -990.38629314976  1.1E-02  8.4E-05  2.1E-03
etot   3  N  Y  2  U  -990.38722199985  9.3E-04  3.1E-05  7.2E-04
etot   4  Y  Y  6  M  -990.38736835388  1.5E-04  1.3E-05  3.4E-04
etot   5  Y  Y  6  M  -990.38740323781  3.5E-05  5.6E-06  1.3E-04
etot   6  Y  Y  6  M  -990.38740916528  5.9E-06  2.4E-06  4.7E-05
etot   7  Y  N  6  M  -990.38741265890  3.5E-06  0.0E+00  0.0E+00


 Energy components, in hartrees:
   (A)  Nuclear repulsion............  1960.02558935013
   (E)  Total one-electron terms..... -5238.29315543660
   (I)  Total two-electron terms.....  2287.88015342757
   (L)  Electronic energy............ -2950.41300200903  (E+I)
   (N)  Total energy.................  -990.38741265890  (A+L)

 SCFE: SCF energy: HF    -990.38741265890 hartrees   iterations:
7
```

```
HOMO energy:    -0.26625
LUMO energy:     0.09232

 Orbital energies:
 -15.60805  -15.56700  -11.29787  -11.29337  -11.27638  -11.25511
 -11.25341  -11.25179  -11.24692  -11.24146  -11.24113  -11.24000
 -11.23941  -11.23867  -11.23797  -11.23592  -11.23504  -11.23469
 -11.23278  -11.23229  -11.22657  -11.22384  -11.22377  -11.21820
 -11.21692   -1.31817   -1.24994   -1.16238   -1.15889   -1.13461
  -1.07262   -1.06754   -1.03658   -1.02296   -1.01802   -1.01474
  -0.96749   -0.95529   -0.93114   -0.85928   -0.83758   -0.83378
  -0.83067   -0.80421   -0.79354   -0.76882   -0.73077   -0.71744
  -0.69958   -0.69803   -0.66500   -0.65657   -0.64316   -0.63792
  -0.63536   -0.62308   -0.61992   -0.61392   -0.59697   -0.59327
  -0.58930   -0.58645   -0.57884   -0.55677   -0.55072   -0.54533
  -0.54289   -0.51775   -0.50819   -0.50301   -0.50024   -0.49594
  -0.49263   -0.49143   -0.48732   -0.48017   -0.42932   -0.40604
  -0.36912   -0.34308   -0.34234   -0.33503   -0.33025   -0.28019
  -0.26625    0.09232    0.12603    0.13625    0.13684    0.14868
   0.17722    0.20851    0.22157    0.23301    0.24218

 end of program scf


 start of program der1a
 end of program der1a


 start of program rwr
 end of program rwr


 start of program der1b

 forces (hartrees/bohr) : total
```

atom	label	x	y	z
1	N1	-9.539155E-03	2.745592E-03	1.044822E-02
2	N2	-2.673465E-03	1.727028E-03	2.906341E-03
3	C4	-1.885556E-04	1.245403E-02	3.193744E-03
4	C5	2.271285E-03	6.211561E-03	1.516907E-03
5	C6	-6.464198E-03	7.479387E-03	7.554108E-03
6	C7	8.793345E-03	-1.029557E-02	-9.843759E-03
7	C13	-5.471678E-04	-7.069051E-03	-5.718790E-03
8	H4	1.852086E-03	3.212933E-03	-7.082379E-04
9	C8	4.338852E-03	4.033060E-03	-3.262937E-03
10	C11	1.606909E-03	-1.006096E-02	-6.249112E-03
11	H7	9.730113E-04	-2.564706E-03	-1.338833E-03
12	C9	-5.458253E-04	-4.327700E-03	-5.240229E-04
13	H8	2.968983E-03	5.464179E-04	-2.555816E-03
14	C10	4.804096E-03	2.217867E-04	-3.885245E-03

```
 15    C12    -7.171547E-03  -3.974109E-03   7.707933E-03
 16    C2      9.332330E-04   2.740879E-04  -3.181994E-03
 17    H13    -2.015010E-03  -1.526317E-03  -1.690651E-03
 18    C14     3.524810E-03  -3.916218E-04  -2.663166E-03
 19    H15     1.889224E-03   3.787146E-03   1.319866E-03
 20    C15    -1.027612E-03   6.505881E-05   3.781739E-04
 21    C16    -4.116869E-03  -8.483180E-04  -5.317073E-03
 22    C17    -1.277121E-04   3.725580E-03  -1.022516E-02
 23    C18     8.848389E-03   3.067855E-03   9.831513E-03
 24    C19     3.253650E-03  -3.767333E-04   8.365422E-03
 25    H16     4.090188E-03   1.430527E-03  -2.015014E-04
 26    H17    -2.307131E-03  -1.447538E-03   4.755238E-04
 27    H18    -8.359663E-03  -3.799457E-03  -4.288966E-04
 28    H19     2.299917E-03   9.846778E-04  -2.783170E-04
 29    H20    -1.387235E-03  -1.403497E-03   2.321291E-03
 30    C1      3.683293E-03   3.676776E-03  -8.195002E-03
 31    H5     -9.851720E-03  -8.517076E-04  -2.436073E-03
 32    H6      1.473065E-03  -4.295533E-03   1.252041E-02
 33    C3      3.914041E-04  -3.266543E-04   4.963776E-04
 34    C26    -2.384326E-03  -3.429782E-03  -4.918934E-03
 35    C27    -1.975575E-03  -5.180976E-04   5.429357E-03
 36    C28     1.047866E-03   1.965373E-03   9.943780E-04
 37    C29     2.585439E-03   2.168460E-03  -3.629454E-03
 38    H1      4.052696E-04   7.061260E-05   1.388971E-03
 39    H2      5.731526E-04   1.449645E-03   2.603645E-05
 40    H3      3.735787E-05  -1.572279E-04   8.376755E-04
 41    H9     -9.939832E-04  -7.438282E-04  -2.855378E-04
 42    H10    -1.057111E-03  -6.022944E-04   1.457865E-03
 43    H25     1.116333E-03  -1.534056E-03  -1.866734E-03
-----------   -------------  -------------  -------------
   total       1.027296E-03   7.528330E-04  -2.351347E-04

 end of program der1b


 start of program geopt   2

 geometry optimization step  2
 reading input hessian of dimension   129
  in five columns format


 Level shifts adjusted to satisfy step-size constraints
  Step size:     0.3001262
  Cos(theta):    0.4522028

  Final level shift:  -7.1431608E-03

 energy change:           -2.1167E-02 .  (  5.0000E-05 )
 gradient maximum:         2.0310E-02 .  (  4.5000E-04 )
 gradient rms:             3.9611E-03 .  (  3.0000E-04 )
 step size:   0.29998  trust radius:   0.30000
```

displacement maximum: 1.2227E-01 . (1.8000E-03)
displacement rms: 2.3569E-02 . (1.2000E-03)
predicted energy change: -3.7426E-03 geom step: 2.9998E-01 full step: 2.9998E-01
molecular structure not yet converged...

center of mass moved by:
x: -9.2535E-03 y: 3.1123E-03 z: 1.4212E-03

new geometry:

atom	x	y (angstroms)	z
N1	-0.2322091003	0.4425447477	1.1722376588
N2	-1.0170271355	-2.2784648229	1.1730995628
C4	-1.2206159278	-0.0226657246	1.9837450839
C5	-0.0448348209	-1.9052512670	0.4751983779
C6	-1.6507777180	-1.3371163143	2.0094539253
C7	-1.7024716670	0.9919018866	2.7899381630
C13	0.6283020713	-2.9170533017	-0.4248611566
H4	-3.0346295842	-2.6472027368	2.9359624232
C8	-2.7497248744	0.6679486212	3.6759761067
C11	-0.9219400908	2.1551243638	2.4291375123
H7	-3.1822925194	1.4081076944	4.3208527754
C9	-3.2122659889	-0.6338983252	3.7021230748
H8	-4.0092368660	-0.8922566392	4.3712351952
C10	-2.6664125428	-1.6434906978	2.8798451206
C12	-0.0462186113	1.7852092098	1.4609796706
C2	0.9639603762	2.6172556086	0.7603660644
H13	1.7049014864	-2.8504962231	-0.3042157835
C14	0.2872845063	-2.7186247711	-1.9011082558
H15	0.3111604572	-3.8957135626	-0.0910075502
C15	-0.3637160763	-2.3879002288	-4.5957973831
C16	1.2521389896	-2.2939910017	-2.8167452985
C17	-1.0099224983	-2.9730715129	-2.3497803317
C18	-1.3345315137	-2.8115555909	-3.6881685682
C19	0.9281516443	-2.1274223633	-4.1580106912
H16	2.2689730521	-2.0919467686	-2.4900654971
H17	-1.7651731246	-3.3056021719	-1.6519350479
H18	-2.3449269541	-3.0307603123	-4.0214737620
H19	1.6928080246	-1.7993395561	-4.8496324883
H20	-0.6146895035	-2.2677049661	-5.6318950361
C1	0.5593460744	-0.4968540721	0.4025037161
H5	1.5835558214	-0.5417582018	0.7564692615
H6	0.6038790034	-0.2150061772	-0.6400473898
C3	2.8215489000	4.2490688071	-0.5283409458
C26	1.8492457635	3.4064361869	1.4997536269
C27	1.0238745658	2.6583201916	-0.6349113427
C28	1.9515451393	3.4616543589	-1.2728168602
C29	2.7672368925	4.2206654051	0.8601900607
H1	1.8310545892	3.3653506796	2.5769048131
H2	0.3330469792	2.0787631427	-1.2253750449
H3	1.9815361995	3.4802193197	-2.3500591580

```
  H9        3.4413282681      4.8236548923      1.4492434236
  H10       3.5316247441      4.8745490259     -1.0248311658
  H25      -1.0230567326      3.1472274108      2.8145615026

  nuclear repulsion energy.......  1967.210994655 hartrees
  ------------------------------------------------
 /  end of geometry optimization iteration   2   /
 ------------------------------------------------

  end of program geopt


  start of program onee
  smallest eigenvalue of S:    2.843E-04
  number of canonical orbitals.....          461
  end of program onee


  start of program probe
  end of program probe


  start of program grid

  number of gridpoints:
     atom        N1     N2     C4     C5     C6     C7    C13
H4
  grid # 1       97    102     84     88     87     89     84
73
  grid # 2      104    112     96     95     97     97     92
118
  grid # 3      214    227    196    186    189    198    163
223
  grid # 4      391    413    320    320    324    348    300
224

  number of gridpoints:
     atom        C8    C11     H7     C9     H8    C10    C12
C2
  grid # 1       89     87     73     87     73     89     86
92
  grid # 2       97     94    118     97    118     97     94
100
  grid # 3      184    185    223    186    222    184    194
196
  grid # 4      330    331    226    331    223    327    316
343

  number of gridpoints:
     atom       H13    C14    H15    C15    C16    C17    C18
C19
```

```
  grid # 1        69     93     69     89     88     88     89
89
  grid # 2       109    101    109     97     96     96     97
96
  grid # 3       211    195    214    184    185    184    185
184
  grid # 4       214    342    212    329    329    328    328
328

  number of gridpoints:
     atom        H16    H17    H18    H19    H20     C1     H5
H6
  grid # 1        72     73     73     73     73     82     70
69
  grid # 2       113    115    118    118    118     88    109
104
  grid # 3       214    215    223    222    224    165    217
207
  grid # 4       221    215    224    222    224    290    217
203

  number of gridpoints:
     atom         C3    C26    C27    C28    C29     H1     H2
H3
  grid # 1        89     89     88     89     89     72     71
73
  grid # 2        97     95     96     97     97    115    114
118
  grid # 3       185    182    185    184    184    218    214
223
  grid # 4       328    328    330    328    329    218    210
223

  number of gridpoints:
     atom         H9    H10    H25  total
  grid # 1        73     73     72   3517
  grid # 2       118    118    115   4490
  grid # 3       223    223    220   8640
  grid # 4       223    224    224  12258

  end of program grid


  start of program rwr
 recomputing RwR matrix   1    grid:  4
  end of program rwr


  start of program scf

       i  u  d  i  g
       t  p  i  c  r                                 RMS    maximum
```

```
      e  d  i  u  i                        energy  density   DIIS
      r  t  s  t  d     total energy       change  change    error

etot   1  N  N  2  U  -990.33510685770            3.7E-04  3.1E-02
etot   2  Y  Y  6  M  -990.38314472816  4.8E-02  1.8E-04  1.2E-02
etot   3  N  Y  2  U  -990.38935537066  6.2E-03  5.3E-05  3.1E-03
etot   4  Y  Y  6  M  -990.38969679674  3.4E-04  2.3E-05  1.1E-03
etot   5  Y  Y  6  M  -990.38977611385  7.9E-05  7.6E-06  2.8E-04
etot   6  N  Y  2  U  -990.38979109239  1.5E-05  3.0E-06  6.9E-05
etot   7  Y  N  6  M  -990.38979365506  2.6E-06  0.0E+00  0.0E+00


 Energy components, in hartrees:
   (A)  Nuclear repulsion............  1967.21099465467
   (E)  Total one-electron terms..... -5252.71557057016
   (I)  Total two-electron terms.....  2295.11478226043
   (L)  Electronic energy............ -2957.60078830974  (E+I)
   (N)  Total energy.................  -990.38979365506  (A+L)

 SCFE: SCF energy: HF    -990.38979365506 hartrees   iterations:
7


 HOMO energy:    -0.26875
 LUMO energy:     0.09679

  Orbital energies:
  -15.60440  -15.56408  -11.29484  -11.28605  -11.27171  -11.25310
  -11.25060  -11.24821  -11.24599  -11.24090  -11.23981  -11.23932
  -11.23928  -11.23863  -11.23847  -11.23780  -11.23743  -11.23721
  -11.23472  -11.23260  -11.22506  -11.21958  -11.21917  -11.21594
  -11.21230   -1.32371   -1.25138   -1.16194   -1.15811   -1.13746
   -1.07380   -1.06529   -1.03784   -1.02261   -1.01706   -1.01675
   -0.97106   -0.95358   -0.93345   -0.86016   -0.83584   -0.83204
   -0.82722   -0.80690   -0.79592   -0.76979   -0.73260   -0.71689
   -0.70107   -0.69646   -0.66553   -0.65782   -0.64254   -0.63657
   -0.63323   -0.62328   -0.62039   -0.61546   -0.59597   -0.59273
   -0.59071   -0.58733   -0.57653   -0.55793   -0.55254   -0.54806
   -0.54230   -0.51743   -0.50677   -0.50276   -0.50055   -0.49573
   -0.49322   -0.49115   -0.48789   -0.48019   -0.42914   -0.40421
   -0.36715   -0.34259   -0.34204   -0.33775   -0.32820   -0.28241
   -0.26875    0.09679    0.12767    0.13691    0.13716    0.14724
    0.17920    0.20956    0.22641    0.23420    0.24136

  end of program scf


  start of program der1a
  end of program der1a


  start of program rwr
```

recomputing RwR matrix 1 grid: 4
end of program rwr

start of program der1b

forces (hartrees/bohr) : total

atom	label	x	y	z
1	N1	1.305964E-03	-1.677270E-03	-2.594790E-03
2	N2	-7.405171E-03	-1.586511E-03	6.320733E-03
3	C4	3.028731E-03	1.790473E-03	-3.798228E-04
4	C5	7.226851E-03	4.524737E-03	-7.098855E-03
5	C6	2.476199E-04	-3.135427E-03	2.297075E-04
6	C7	-2.330886E-03	2.478329E-03	3.273788E-04
7	C13	2.227097E-04	1.311746E-03	-4.798910E-03
8	H4	-1.002454E-03	-3.214195E-03	-8.533394E-05
9	C8	2.159221E-03	-4.853058E-03	-3.527892E-03
10	C11	-3.302127E-03	-1.233147E-03	3.282272E-03
11	H7	-7.362198E-04	1.749372E-03	1.319340E-03
12	C9	3.320533E-03	4.276770E-03	-1.934309E-03
13	H8	-1.912353E-03	-7.144657E-04	1.822387E-03
14	C10	8.315593E-04	4.548646E-03	7.271826E-04
15	C12	4.548962E-03	-1.450949E-03	-2.371630E-03
16	C2	-9.938152E-04	7.535157E-04	1.267906E-03
17	H13	1.386542E-03	-4.258552E-04	9.927676E-04
18	C14	2.299989E-03	-7.682804E-05	1.238775E-03
19	H15	1.984307E-04	-8.884950E-04	-8.118002E-04
20	C15	-6.451323E-04	-1.027048E-03	4.774511E-03
21	C16	-2.632256E-04	-3.149773E-04	3.356515E-03
22	C17	1.008954E-03	6.183636E-05	-1.888416E-03
23	C18	1.484312E-04	-5.973317E-04	-2.098115E-03
24	C19	-1.156244E-03	-3.591191E-04	4.042049E-04
25	H16	-7.120642E-03	-2.020923E-03	-6.701640E-04
26	H17	3.236541E-03	1.487269E-03	-1.213183E-03
27	H18	7.301887E-03	2.526935E-03	1.127166E-03
28	H19	-4.266701E-03	-1.422013E-03	1.600621E-03
29	H20	-5.797366E-04	3.861398E-04	-2.246165E-03
30	C1	-5.155529E-03	-8.921110E-04	1.159786E-03
31	H5	2.282436E-03	2.852896E-04	2.405521E-03
32	H6	-2.782347E-04	1.257850E-03	-1.502622E-03
33	C3	-3.641709E-03	-3.629131E-03	3.069671E-03
34	C26	-2.578335E-03	-3.574081E-03	-3.583493E-04
35	C27	-8.629869E-04	-1.384270E-03	3.235411E-03
36	C28	1.635804E-03	2.327281E-03	9.648920E-04
37	C29	8.904502E-04	7.644047E-04	-3.194343E-03
38	H1	-1.706450E-03	-2.318215E-04	-2.241600E-03
39	H2	4.690082E-04	6.810360E-04	2.172640E-03
40	H3	8.078606E-04	8.518771E-04	1.530140E-03
41	H9	-1.100102E-03	-1.113211E-03	-2.495881E-03
42	H10	3.825774E-03	3.254866E-03	-2.412596E-03

```
 43    H25    -2.808875E-04   1.593320E-03   4.916000E-04
-----------   -------------  -------------  -------------
   total       1.065317E-03   1.089457E-03  -1.036505E-04

 end of program der1b


 start of program geopt   3

 geometry optimization step  3
 reading input hessian of dimension   129
  in five columns format
 reading input hessian of dimension   129
  in five columns format



 Level shifts adjusted to satisfy step-size constraints
  Step size:    0.3000236
  Cos(theta):   0.4953472

  Final level shift:  -1.6630388E-02

 energy change:          -2.3810E-03 .  (  5.0000E-05 )
 gradient maximum:        9.9076E-03 .  (  4.5000E-04 )
 gradient rms:            2.3236E-03 .  (  3.0000E-04 )
 step size:   0.29975  trust radius:   0.30000
 displacement maximum:    1.0837E-01 .  (  1.8000E-03 )
 displacement rms:        2.3551E-02 .  (  1.2000E-03 )
 predicted energy change:    -2.9446E-03    geom step:   2.9975E-
01    full step:   2.9975E-01
 molecular structure not yet converged...

 center of mass moved by:
    x:     4.5379E-03     y:     1.6225E-03     z:    -1.0573E-02

 new geometry:
                              angstroms
 atom           x                 y                 z
 N1       -0.2064093151      0.4564334872      1.1449569640
 N2       -0.9777143120     -2.2675882391      1.1401282031
 C4       -1.2067896365     -0.0074822023      1.9347114372
 C5        0.0100178696     -1.8812418506      0.4407932188
 C6       -1.6301115836     -1.3290633316      1.9647297688
 C7       -1.7180530931      1.0023503931      2.7134818099
 C13       0.7013842690     -2.8816737324     -0.4740592873
 H4       -3.0216327063     -2.6539904306      2.8557401227
 C8       -2.7796853502      0.6766421222      3.5699349035
 C11      -0.9469746435      2.1651389329      2.3678165600
 H7       -3.2396438255      1.4178307697      4.2013122414
 C9       -3.2317902692     -0.6316840549      3.6015379272
 H8       -4.0475645815     -0.8929676898      4.2526147254
 C10      -2.6592479127     -1.6425581119      2.8070396413
```

```
C12      -0.0282154727     1.7951337785     1.4350689770
C2        0.9934644979     2.6280168532     0.7795619441
H13       1.7730430366    -2.7575478083    -0.4156955215
C14       0.2865859920    -2.7249268767    -1.9051731340
H15       0.4713121897    -3.8629015133    -0.0943843922
C15      -0.5615655542    -2.4619627032    -4.5190042806
C16       1.1125815133    -2.1862381413    -2.8583626252
C17      -0.9661916103    -3.1291782413    -2.2777042664
C18      -1.3890096252    -3.0026868085    -3.5700862667
C19       0.6893320833    -2.0524259860    -4.1584209800
H16       2.0979129012    -1.8591107039    -2.5945051992
H17      -1.6160289056    -3.5619217481    -1.5421987308
H18      -2.3733434532    -3.3581172536    -3.8357831773
H19       1.3434543159    -1.6264018218    -4.8889259482
H20      -0.9021702747    -2.3804723563    -5.5318037396
C1        0.5974414175    -0.4754948750     0.3889408228
H5        1.6054220101    -0.5235796038     0.7857568676
H6        0.6751609178    -0.1866596432    -0.6452941877
C3        2.9244707837     4.2594570176    -0.3923566804
C26       1.7907979932     3.4485799055     1.5329596121
C27       1.1713262529     2.6405355796    -0.5729922605
C28       2.1366910125     3.4439199169    -1.1538018568
C29       2.7452329284     4.2656384654     0.9532276351
H1        1.6745334301     3.4364257238     2.5857229416
H2        0.5529801450     2.0327292614    -1.1904461656
H3        2.2687279040     3.4308993892    -2.2062196320
H9        3.3448720185     4.8980738147     1.5650329855
H10       3.6696029435     4.8876836769    -0.8403675527
H25      -1.0676334116     3.1643232144     2.7358050805

nuclear repulsion energy.......  1978.365603056 hartrees
 --------------------------------------------------
/  end of geometry optimization iteration   3   /
--------------------------------------------------

end of program geopt


start of program onee
smallest eigenvalue of S:    2.790E-04
number of canonical orbitals.....          461
end of program onee


start of program probe
end of program probe


start of program grid

number of gridpoints:
```

atom	N1	N2	C4	C5	C6	C7	C13	H4
grid # 1	96	102	86	88	90	88	84	73
grid # 2	104	112	95	95	99	96	92	118
grid # 3	214	227	194	188	192	198	164	224
grid # 4	389	414	321	321	322	344	298	223

number of gridpoints:

atom	C8	C11	H7	C9	H8	C10	C12	C2
grid # 1	89	87	73	87	73	89	86	88
grid # 2	97	94	118	97	118	97	94	99
grid # 3	185	184	223	185	222	184	195	191
grid # 4	331	331	226	330	223	326	320	339

number of gridpoints:

atom	H13	C14	H15	C15	C16	C17	C18	C19
grid # 1	69	93	69	87	86	87	87	87
grid # 2	109	101	109	97	96	96	97	97
grid # 3	211	191	213	182	182	181	182	182
grid # 4	209	339	212	325	327	327	328	327

number of gridpoints:

atom	H16	H17	H18	H19	H20	C1	H5	H6
grid # 1	72	72	73	73	73	82	70	69
grid # 2	113	114	118	118	118	87	108	103
grid # 3	213	215	222	221	223	163	217	204
grid # 4	213	214	223	222	224	290	217	202

number of gridpoints:

atom	C3	C26	C27	C28	C29	H1	H2	H3
grid # 1	89	87	86	87	87	72	70	73

```
  grid # 2       97     96     96     97     97    115    113
118
  grid # 3      182    181    182    182    183    217    213
222
  grid # 4      328    326    327    327    324    216    210
222

  number of gridpoints:
     atom        H9    H10    H25  total
  grid # 1       73     73     72   3497
  grid # 2      118    118    115   4486
  grid # 3      222    222    219   8597
  grid # 4      223    223    222  12205

  end of program grid


  start of program rwr
  end of program rwr


  start of program scf

       i  u  d  i  g
       t  p  i  c  r                                   RMS    maximum
       e  d  i  u  i                         energy  density   DIIS
       r  t  s  t  d     total energy        change  change    error

etot   1  N  N  2  U  -990.32090183004              4.0E-04  2.7E-02
etot   2  Y  Y  6  M  -990.37602444074  5.5E-02  1.9E-04  1.1E-02
etot   3  N  Y  2  U  -990.38279174353  6.8E-03  5.6E-05  2.9E-03
etot   4  Y  Y  6  M  -990.38330673990  5.1E-04  2.8E-05  1.2E-03
etot   5  Y  Y  6  M  -990.38339751676  9.1E-05  7.9E-06  2.7E-04
etot   6  N  Y  2  U  -990.38336351948 -3.4E-05  3.5E-06  8.1E-05
etot   7  Y  N  6  M  -990.38336784090  4.3E-06  0.0E+00  0.0E+00


 Energy components, in hartrees:
   (A)  Nuclear repulsion............  1978.36560305571
   (E)  Total one-electron terms..... -5274.81755689824
   (I)  Total two-electron terms.....  2306.06858600163
   (L)  Electronic energy............ -2968.74897089661  (E+I)
   (N)  Total energy.................  -990.38336784090  (A+L)

 SCFE: SCF energy: HF    -990.38336784090 hartrees   iterations:
7


 HOMO energy:    -0.26814
 LUMO energy:     0.09639

  Orbital energies:
```

```
-15.60547  -15.56550  -11.29770  -11.28619  -11.27078  -11.25022
-11.24528  -11.24450  -11.23825  -11.23745  -11.23449  -11.23301
-11.23221  -11.23109  -11.23069  -11.23034  -11.22948  -11.22838
-11.22673  -11.22459  -11.22253  -11.21876  -11.21686  -11.21593
-11.21192   -1.32742   -1.24676   -1.17463   -1.17229   -1.14078
 -1.07809   -1.07260   -1.04756   -1.02329   -1.02291   -1.02144
 -0.97578   -0.95637   -0.93529   -0.86214   -0.84435   -0.83597
 -0.83375   -0.80916   -0.79677   -0.77365   -0.73422   -0.72183
 -0.70571   -0.70299   -0.66886   -0.65951   -0.64726   -0.64126
 -0.63446   -0.62424   -0.62327   -0.61823   -0.59835   -0.59393
 -0.59044   -0.58890   -0.58373   -0.56063   -0.55582   -0.54996
 -0.54261   -0.52147   -0.51054   -0.50427   -0.50252   -0.49989
 -0.49515   -0.49181   -0.48895   -0.48236   -0.42870   -0.40579
 -0.37068   -0.34887   -0.34027   -0.33992   -0.33265   -0.28268
 -0.26814    0.09639    0.13129    0.13834    0.14272    0.15301
  0.18158    0.21027    0.22587    0.23576    0.24199

end of program scf


start of program der1a
end of program der1a


start of program rwr
end of program rwr


start of program der1b

forces (hartrees/bohr) : total

atom  label         x              y              z
----  -----   -------------  -------------  -------------
  1    N1      2.088108E-03  -1.816608E-03  -3.641703E-03
  2    N2      1.359145E-02   2.603072E-03  -9.392358E-03
  3    C4      4.660404E-04  -1.020443E-02  -2.510234E-03
  4    C5     -1.216198E-02  -6.585574E-03   6.161446E-03
  5    C6      7.087680E-03  -4.398158E-04  -6.447246E-03
  6    C7     -3.899426E-03   5.933573E-03   2.324081E-03
  7    C13     3.821605E-03   5.890252E-03   1.357044E-02
  8    H4      2.973135E-04   4.132462E-04   7.230307E-05
  9    C8     -6.397915E-03  -7.377681E-03   4.298384E-03
 10    C11     3.207322E-03   5.420828E-03   1.334689E-03
 11    H7      6.085201E-04   2.998135E-05  -7.440425E-04
 12    C9     -3.822426E-04   8.286041E-03   2.243006E-03
 13    H8      2.340008E-04   7.691603E-04   4.906764E-04
 14    C10    -1.938760E-03  -6.468270E-04   1.244062E-03
 15    C12    -6.102382E-03  -1.064691E-03   1.815286E-03
 16    C2     -8.753000E-03  -6.468478E-03   1.178688E-02
 17    H13     5.162556E-03   1.641355E-03   3.634586E-03
 18    C14     3.407200E-03  -1.591991E-03   8.898751E-03
```

```
 19    H15    -2.336800E-03  -4.255029E-03  -1.094404E-03
 20    C15    -8.884359E-03  -7.898080E-04  -1.509099E-02
 21    C16     1.333100E-02   6.137570E-03   1.627549E-03
 22    C17    -1.655284E-02  -9.413400E-03   8.281097E-03
 23    C18    -2.099264E-02  -9.634396E-03  -4.550000E-03
 24    C19     1.119810E-02   6.856261E-03  -8.067226E-03
 25    H16     4.778835E-03   6.395915E-04   1.815445E-03
 26    H17    -2.269290E-03  -3.734078E-04   2.039251E-03
 27    H18     1.898389E-03   2.441004E-03  -7.736249E-04
 28    H19     4.019581E-03   1.995855E-03  -3.556696E-03
 29    H20     5.225823E-04   1.660109E-03  -3.684042E-03
 30    C1     -1.526528E-04  -1.293907E-03   5.029993E-03
 31    H5      3.804775E-03   3.806220E-04   3.734436E-04
 32    H6      1.802152E-04   1.858511E-03  -4.200778E-03
 33    C3      1.062426E-02   8.644818E-03  -1.207225E-02
 34    C26     1.147070E-02   1.162699E-02   1.033083E-02
 35    C27     4.957450E-03  -1.858617E-04  -2.536308E-02
 36    C28    -9.660113E-03  -1.041083E-02  -8.949314E-03
 37    C29    -5.181304E-03  -1.111879E-03   2.398876E-02
 38    H1     -3.259171E-04   1.476531E-03   1.312592E-02
 39    H2     -4.697105E-03  -3.791650E-03  -6.595315E-03
 40    H3     -5.870500E-04  -1.164633E-03  -1.251753E-02
 41    H9      4.371697E-03   4.237461E-03   6.763454E-03
 42    H10     1.379739E-03   7.800523E-04  -2.571094E-03
 43    H25    -3.342425E-04  -4.287707E-04   6.210274E-04
-----------   ------------   ------------   ------------
   total       8.991054E-04   6.732157E-04   4.943495E-05

 end of program der1b


 start of program geopt   4

 geometry optimization step  4
 reading input hessian of dimension   129
  in five columns format
 reading input hessian of dimension   129
  in five columns format
 ** restarting optimization from step    3 **


 Level shifts adjusted to satisfy step-size constraints
  Step size:    0.3000460
  Cos(theta):   0.3075896

  Final level shift:  -4.9937562E-03

 energy change:           6.4258E-03 .  (  5.0000E-05 )
 gradient maximum:        9.9076E-03 .  (  4.5000E-04 )
 gradient rms:            2.3236E-03 .  (  3.0000E-04 )
 step size:   0.29978  trust radius:   0.30000
 displacement maximum:    1.5805E-01 .  (  1.8000E-03 )
```

displacement rms: 2.3553E-02 . (1.2000E-03)
predicted energy change: -1.5876E-03 geom step: 2.9978E-01 full step: 2.9978E-01
molecular structure not yet converged...

center of mass moved by:
x: -2.4980E-16 y: -3.4694E-17 z: -6.1062E-16

new geometry:

	angstroms		
atom	x	y	z
N1	-0.2605037549	0.4125439612	1.1900034197
N2	-1.0201882848	-2.3117451892	1.2286465763
C4	-1.2291547382	-0.0501813353	2.0213210471
C5	-0.0584840655	-1.9323738777	0.5045877913
C6	-1.6433405541	-1.3707651498	2.0724302477
C7	-1.7196322432	0.9707097685	2.8115399710
C13	0.6123641543	-2.9395135162	-0.4045486213
H4	-3.0004875650	-2.6843033639	3.0337672096
C8	-2.7502677659	0.6466074665	3.7128944205
C11	-0.9641692572	2.1368330189	2.4248916539
H7	-3.1870956384	1.3938929758	4.3523390576
C9	-3.1923609494	-0.6577264470	3.7717764284
H8	-3.9791920871	-0.9171763497	4.4577173169
C10	-2.6430824576	-1.6735110866	2.9607130298
C12	-0.0894151548	1.7589272383	1.4540514090
C2	0.8975931598	2.5804167511	0.7178403216
H13	1.6900885460	-2.9175445528	-0.2351375024
C14	0.3269913213	-2.6769735270	-1.8741990189
H15	0.2546697460	-3.9228910664	-0.1143387386
C15	-0.2180137009	-2.2191864228	-4.5767173828
C16	1.3496559462	-2.4227648804	-2.7664884238
C17	-0.9764471114	-2.6992027704	-2.3557447632
C18	-1.2462393835	-2.4742543646	-3.6931624843
C19	1.0776231861	-2.1937356607	-4.1071135875
H16	2.3658535888	-2.4078532060	-2.4199820995
H17	-1.7766859001	-2.8933381124	-1.6738564557
H18	-2.2487902734	-2.4928964715	-4.0444386495
H19	1.8875121812	-1.9993966839	-4.7814773829
H20	-0.4271057620	-2.0495195336	-5.6159740449
C1	0.5363502956	-0.5262371704	0.4297996206
H5	1.5569005697	-0.5698600523	0.8077454585
H6	0.5969268301	-0.2400532601	-0.6132951547
C3	2.7366658910	4.1635728363	-0.6570139302
C26	1.8494198086	3.3298911691	1.4147477126
C27	0.8738252674	2.6383115327	-0.6774631325
C28	1.7919425574	3.4195085202	-1.3586689873
C29	2.7597734096	4.1195096692	0.7311384976
H1	1.8863612505	3.2798815381	2.4949515492
H2	0.1256541849	2.0873284863	-1.2299565889
H3	1.7610423948	3.4526209625	-2.4364084657
H9	3.4896654651	4.6919589348	1.2854978074

```
  H10       3.4478987066      4.7719686917     -1.1860693938
  H25      -1.0742926529      3.1373751310      2.7923517727

  nuclear repulsion energy.......  1974.668059347 hartrees
  ------------------------------------------------------
 /  end of geometry optimization iteration   4   /
 ------------------------------------------------------

  end of program geopt


  start of program onee
  smallest eigenvalue of S:    2.817E-04
  number of canonical orbitals.....          461
  end of program onee


  start of program probe
  end of program probe


  start of program grid

  number of gridpoints:
     atom        N1     N2      C4      C5      C6      C7     C13
H4
  grid # 1       97    102      87      88      90      88      84
73
  grid # 2      104    112      95      95      99      98      92
118
  grid # 3      214    227     195     186     191     200     164
224
  grid # 4      389    414     320     320     324     347     299
224

  number of gridpoints:
     atom        C8    C11      H7      C9      H8     C10     C12
C2
  grid # 1       89     86      73      87      73      89      84
92
  grid # 2       97     94     118      97     118      97      95
100
  grid # 3      184    185     223     184     222     184     195
196
  grid # 4      330    330     226     331     223     327     321
343

  number of gridpoints:
     atom       H13    C14     H15     C15     C16     C17     C18
C19
  grid # 1       69     91      69      89      88      86      89
87
```

```
  grid # 2      110    100    110     97     96     96     97
96
  grid # 3      210    196    218    182    183    183    183
182
  grid # 4      215    342    217    327    327    327    327
327

  number of gridpoints:
     atom       H16    H17    H18    H19    H20     C1     H5
H6
  grid # 1       73     73     73     73     73     82     70
69
  grid # 2      113    115    118    118    118     88    109
105
  grid # 3      214    216    222    221    223    165    217
208
  grid # 4      213    214    223    222    224    290    217
206

  number of gridpoints:
     atom        C3    C26    C27    C28    C29     H1     H2
H3
  grid # 1       89     89     88     89     89     73     71
73
  grid # 2       97     95     96     97     97    115    114
118
  grid # 3      185    182    185    184    184    218    216
223
  grid # 4      330    328    329    328    329    219    214
223

  number of gridpoints:
     atom        H9    H10    H25  total
  grid # 1       73     73     73   3516
  grid # 2      118    118    115   4495
  grid # 3      223    224    221   8642
  grid # 4      224    224    224  12258

  end of program grid


  start of program rwr
  end of program rwr


  start of program scf

       i  u  d  i  g
       t  p  i  c  r                                RMS    maximum
       e  d  i  u  i                      energy  density   DIIS
       r  t  s  t  d     total energy     change  change    error
```

```
etot   1  N  N  2  U  -990.14145563547            7.8E-04  3.9E-02
etot   2  Y  Y  6  M  -990.35861329482  2.2E-01  3.9E-04  1.6E-02
etot   3  N  Y  2  U  -990.38809039062  2.9E-02  1.2E-04  3.9E-03
etot   4  Y  Y  6  M  -990.38982038619  1.7E-03  4.1E-05  1.2E-03
etot   5  Y  Y  6  M  -990.39009100052  2.7E-04  1.6E-05  3.2E-04
etot   6  N  Y  2  U  -990.39013660923  4.6E-05  5.8E-06  1.0E-04
etot   7  Y  Y  6  M  -990.39013977366  3.2E-06  2.4E-06  3.6E-05
etot   8  Y  N  6  M  -990.39014043744  6.6E-07  0.0E+00  0.0E+00


 Energy components, in hartrees:
   (A)  Nuclear repulsion............  1974.66805934711
   (E)  Total one-electron terms..... -5267.59739614095
   (I)  Total two-electron terms.....  2302.53919635640
   (L)  Electronic energy............ -2965.05819978455  (E+I)
   (N)  Total energy.................  -990.39014043744  (A+L)

 SCFE: SCF energy: HF    -990.39014043744 hartrees   iterations:
8


 HOMO energy:    -0.26872
 LUMO energy:     0.09755

  Orbital energies:
  -15.60449  -15.56529  -11.29607  -11.28604  -11.27093  -11.25222
  -11.25039  -11.24527  -11.24459  -11.24123  -11.24070  -11.24044
  -11.24005  -11.23923  -11.23844  -11.23434  -11.23294  -11.23235
  -11.23098  -11.22932  -11.22452  -11.21957  -11.21815  -11.21574
  -11.21231   -1.32548   -1.24923   -1.16438   -1.16096   -1.13857
   -1.07363   -1.06865   -1.03835   -1.02173   -1.02055   -1.01749
   -0.97069   -0.95517   -0.93360   -0.86029   -0.83635   -0.83387
   -0.83017   -0.80766   -0.79576   -0.76982   -0.73217   -0.71750
   -0.70155   -0.70000   -0.66622   -0.65729   -0.64350   -0.63778
   -0.63371   -0.62436   -0.62014   -0.61563   -0.59577   -0.59197
   -0.59045   -0.58757   -0.58039   -0.55733   -0.55239   -0.54828
   -0.54513   -0.51672   -0.50646   -0.50304   -0.50031   -0.49689
   -0.49432   -0.49097   -0.48728   -0.48190   -0.42913   -0.40459
   -0.36595   -0.34214   -0.34166   -0.33817   -0.33118   -0.28287
   -0.26872    0.09755    0.12906    0.13686    0.13760    0.14839
    0.17922    0.20764    0.22771    0.23529    0.24258

  end of program scf


  start of program der1a
  end of program der1a


  start of program rwr
  end of program rwr
```

start of program der1b

forces (hartrees/bohr) : total

atom	label	x	y	z
1	N1	1.722647E-04	-1.371273E-03	-2.097232E-03
2	N2	2.307619E-03	2.645434E-04	-1.429536E-03
3	C4	9.233719E-04	-1.909727E-03	-3.397651E-04
4	C5	-2.472205E-03	-1.364835E-03	1.074612E-03
5	C6	9.104396E-04	-8.696098E-04	-8.667630E-04
6	C7	-2.264358E-03	1.987665E-03	9.229630E-04
7	C13	1.704094E-05	-1.512183E-03	1.932501E-03
8	H4	-1.303564E-04	-3.601330E-04	6.584231E-05
9	C8	-2.402673E-04	-8.558610E-04	6.577247E-04
10	C11	1.082298E-03	1.304216E-03	5.319032E-04
11	H7	2.368012E-04	2.173179E-04	-3.217904E-04
12	C9	5.433283E-04	1.494368E-03	-2.250984E-04
13	H8	-7.318402E-06	9.919423E-05	2.294528E-04
14	C10	-3.416522E-04	-3.416849E-04	3.024806E-05
15	C12	5.998397E-04	6.585509E-04	7.717878E-04
16	C2	1.338325E-03	2.564232E-03	7.609119E-04
17	H13	-2.445629E-03	3.019296E-04	-7.005633E-04
18	C14	-1.006014E-03	-2.287806E-03	2.671755E-03
19	H15	3.635537E-04	1.884244E-03	-9.943853E-04
20	C15	2.027062E-03	2.500076E-04	-2.559217E-03
21	C16	2.727088E-03	1.063025E-03	-3.560983E-03
22	C17	-9.564304E-04	-6.952268E-05	-1.579709E-04
23	C18	2.482024E-03	-4.635361E-04	6.616306E-03
24	C19	5.817905E-03	2.160421E-04	-5.459483E-04
25	H16	2.531985E-03	4.057878E-04	1.288178E-04
26	H17	-4.824063E-03	-1.082739E-03	2.997523E-03
27	H18	-1.047403E-02	-7.737787E-04	-2.242630E-03
28	H19	2.831240E-03	6.714518E-04	-2.296848E-03
29	H20	2.548070E-04	7.177312E-04	-1.647014E-03
30	C1	4.326349E-04	1.117342E-03	1.134530E-03
31	H5	3.099164E-04	3.869422E-04	-1.953429E-05
32	H6	-4.484517E-05	5.228931E-04	6.438714E-04
33	C3	-2.488043E-03	-2.181722E-03	6.024058E-04
34	C26	-2.683586E-03	-2.958448E-03	1.442213E-03
35	C27	2.666601E-04	-4.104175E-04	1.506358E-03
36	C28	2.305602E-03	2.215407E-03	4.983410E-04
37	C29	8.628620E-04	7.523044E-04	-1.843323E-03
38	H1	-1.674844E-03	-2.410790E-04	-4.984929E-03
39	H2	2.525584E-03	1.790540E-03	2.833707E-03
40	H3	8.433839E-04	7.493248E-04	2.107534E-03
41	H9	-2.068308E-03	-1.653712E-03	-2.587104E-03
42	H10	8.221141E-05	1.717202E-04	-7.402267E-04
43	H25	-1.315526E-04	-2.308952E-04	-4.026906E-05
total		5.423385E-04	8.678193E-04	-3.982191E-05

```
end of program der1b


start of program geopt   5

geometry optimization step  5
reading input hessian of dimension   129
 in five columns format
reading input hessian of dimension   129
 in five columns format


Level shifts adjusted to satisfy step-size constraints
 Step size:    0.3001034
 Cos(theta):   0.4746771

 Final level shift:  -1.8111015E-02

energy change:          -3.4678E-04 .  (  5.0000E-05 )
gradient maximum:        1.0656E-02 .  (  4.5000E-04 )
gradient rms:            2.1734E-03 .  (  3.0000E-04 )
step size:   0.29956  trust radius:   0.30000
displacement maximum:    1.2495E-01 .  (  1.8000E-03 )
displacement rms:        2.3536E-02 .  (  1.2000E-03 )
predicted energy change:    -2.7815E-03    geom step:   2.9956E-
01    full step:   2.9956E-01
molecular structure not yet converged...

center of mass moved by:
   x:     8.1464E-03     y:     6.0566E-03     z:    -8.7285E-03

new geometry:
                              angstroms
atom           x                 y                 z
N1       -0.2292024469      0.4427015271      1.1628880103
N2       -0.9782568628     -2.2878207032      1.1779266582
C4       -1.2094337002     -0.0303642055      1.9757531149
C5       -0.0161249279     -1.9029247248      0.4674816553
C6       -1.6194223744     -1.3523145475      2.0109279641
C7       -1.7211646352      0.9868621882      2.7632723068
C13       0.6544591552     -2.9143021590     -0.4408657883
H4       -2.9999077625     -2.6718770241      2.9344446436
C8       -2.7718199836      0.6572998838      3.6401084653
C11      -0.9491404552      2.1543222696      2.4120276559
H7       -3.2211900614      1.4029180697      4.2716980948
C9       -3.2132989134     -0.6466971041      3.6786741679
H8       -4.0163262814     -0.9105012423      4.3442732707
C10      -2.6416683415     -1.6597368984      2.8743855662
C12      -0.0562922792      1.7860587709      1.4560571753
C2        0.9488951143      2.6311226377      0.7701830808
H13       1.7281674582     -2.8468821058     -0.3270846920
```

```
 C14       0.2937960086    -2.7261589105    -1.9088007454
 H15       0.3445375745    -3.8922911028    -0.1088900635
 C15      -0.4402906415    -2.3952792758    -4.5794029238
 C16       1.2395116030    -2.3428910048    -2.8630371971
 C17      -1.0270889510    -2.9533277298    -2.3085099819
 C18      -1.3921717180    -2.7920309200    -3.6337066705
 C19       0.8788052648    -2.1725843519    -4.1956505663
 H16       2.2638244085    -2.1880678707    -2.5683467508
 H17      -1.7675239977    -3.2630084538    -1.5767242550
 H18      -2.4257099259    -2.9929594166    -3.9302355350
 H19       1.6217050002    -1.8773108746    -4.9256653884
 H20      -0.7280733175    -2.2675932286    -5.6111620858
 C1        0.5821138301    -0.4915229124     0.4118188533
 H5        1.5989978845    -0.5325329495     0.8024327801
 H6        0.6562234427    -0.2012124645    -0.6287278123
 C3        2.8551861345     4.2505727705    -0.4749461088
 C26       1.7771405951     3.4522098043     1.5201834275
 C27       1.0866007330     2.6404146539    -0.6152847579
 C28       2.0341472297     3.4391158849    -1.2329113044
 C29       2.7192766740     4.2587160999     0.9026633710
 H1        1.6846480152     3.4442277353     2.5858043452
 H2        0.4363216332     2.0366757013    -1.2116377193
 H3        2.1295952060     3.4333746931    -2.3030217836
 H9        3.3514260736     4.8860237979     1.4956835722
 H10       3.5921450310     4.8707133033    -0.9544874007
 H25      -1.0689547267     3.1499012113     2.7877233660

 nuclear repulsion energy.......  1967.972340116 hartrees
 -------------------------------------------------
/  end of geometry optimization iteration   5   /
-------------------------------------------------

 end of program geopt


 start of program onee
 smallest eigenvalue of S:    2.829E-04
 number of canonical orbitals.....           461
 end of program onee


 start of program probe
 end of program probe


 start of program grid

 number of gridpoints:
    atom        N1     N2     C4     C5     C6     C7    C13
H4
 grid # 1       95    102     87     88     90     90     84
73
```

```
  grid # 2      103    112     95     95     99     98     92
118
  grid # 3      212    227    195    188    190    199    163
224
  grid # 4      386    413    321    320    321    348    300
224

  number of gridpoints:
     atom         C8    C11     H7     C9     H8    C10    C12
C2
  grid # 1       89     87     73     87     73     89     86
92
  grid # 2       97     94    118     97    118     97     95
100
  grid # 3      184    185    223    184    222    184    195
195
  grid # 4      328    330    226    331    223    327    320
340

  number of gridpoints:
     atom        H13    C14    H15    C15    C16    C17    C18
C19
  grid # 1       69     93     69     89     88     88     89
89
  grid # 2      109    100    110     97     96     96     97
96
  grid # 3      211    198    213    185    185    184    185
184
  grid # 4      210    342    212    330    329    328    329
329

  number of gridpoints:
     atom        H16    H17    H18    H19    H20     C1     H5
H6
  grid # 1       72     72     73     73     73     82     70
69
  grid # 2      115    114    118    118    118     88    110
104
  grid # 3      214    215    223    222    224    163    217
206
  grid # 4      214    215    232    223    224    292    217
204

  number of gridpoints:
     atom         C3    C26    C27    C28    C29     H1     H2
H3
  grid # 1       89     87     88     89     89     72     70
73
  grid # 2       97     95     96     97     97    115    113
118
  grid # 3      184    182    184    184    185    217    213
222
```

```
  grid # 4      328    327    329    327    328    215    211
224

  number of gridpoints:
     atom        H9    H10    H25  total
  grid # 1       73     73     72   3518
  grid # 2      118    118    115   4493
  grid # 3      222    223    219   8634
  grid # 4      224    224    223  12248

  end of program grid


  start of program rwr
  end of program rwr


  start of program scf

       i  u  d  i  g
       t  p  i  c  r                                    RMS    maximum
       e  d  i  u  i                         energy  density   DIIS
       r  t  s  t  d     total energy    change  change    error

etot   1  N  N  2  U  -990.30892225319           4.4E-04  2.7E-02
etot   2  Y  Y  6  M  -990.37951017403  7.1E-02  2.2E-04  1.1E-02
etot   3  N  Y  2  U  -990.38894674829  9.4E-03  6.5E-05  2.8E-03
etot   4  Y  Y  6  M  -990.38948696771  5.4E-04  2.2E-05  8.5E-04
etot   5  Y  Y  6  M  -990.38957252266  8.6E-05  9.0E-06  2.1E-04
etot   6  N  Y  2  U  -990.38957720340  4.7E-06  3.0E-06  4.3E-05
etot   7  Y  N  6  M  -990.38957709405 -1.1E-07  0.0E+00  0.0E+00


 Energy components, in hartrees:
   (A)  Nuclear repulsion............  1967.97234011558
   (E)  Total one-electron terms..... -5254.19895519368
   (I)  Total two-electron terms.....  2295.83703798405
   (L)  Electronic energy............ -2958.36191720963  (E+I)
   (N)  Total energy.................  -990.38957709405  (A+L)

 SCFE: SCF energy: HF    -990.38957709405 hartrees   iterations:
7


 HOMO energy:    -0.26884
 LUMO energy:     0.09597

  Orbital energies:
  -15.60543  -15.56487  -11.29622  -11.28765  -11.27212  -11.25108
  -11.25053  -11.24792  -11.24652  -11.23892  -11.23891  -11.23883
  -11.23801  -11.23777  -11.23716  -11.23711  -11.23637  -11.23587
  -11.23553  -11.23260  -11.22496  -11.22078  -11.21925  -11.21651
```

```
-11.21329   -1.32543   -1.25168   -1.16443   -1.15738   -1.13804
 -1.07535   -1.06529   -1.04001   -1.02194   -1.01727   -1.01663
 -0.97152   -0.95322   -0.93415   -0.85977   -0.83914   -0.83253
 -0.82686   -0.80677   -0.79549   -0.77050   -0.73321   -0.71759
 -0.70203   -0.69559   -0.66640   -0.65820   -0.64265   -0.63725
 -0.63362   -0.62320   -0.62133   -0.61564   -0.59719   -0.59253
 -0.59025   -0.58763   -0.57566   -0.55800   -0.55276   -0.54852
 -0.54404   -0.51815   -0.50817   -0.50261   -0.50009   -0.49525
 -0.49276   -0.49132   -0.48786   -0.48039   -0.42993   -0.40500
 -0.36914   -0.34440   -0.34069   -0.33787   -0.32708   -0.28199
 -0.26884    0.09597    0.12827    0.13658    0.13747    0.14798
  0.17910    0.21010    0.22715    0.23493    0.24011

end of program scf


start of program der1a
end of program der1a


start of program rwr
end of program rwr


start of program der1b

forces (hartrees/bohr) : total

atom  label         x              y              z
----  -----   -------------  -------------  -------------
  1    N1      1.630734E-03   4.147837E-04  -2.328007E-04
  2    N2     -4.394325E-03  -1.352038E-03   2.472753E-03
  3    C4     -1.011245E-03   1.568492E-03   3.045752E-05
  4    C5      4.998187E-03   1.221166E-03  -4.075432E-03
  5    C6     -2.039398E-03  -4.311267E-04   1.780415E-03
  6    C7      6.101085E-05  -2.060345E-04   7.720866E-04
  7    C13     4.203179E-04   3.755701E-03  -3.272974E-03
  8    H4      5.925810E-05   2.078679E-04   8.546451E-05
  9    C8      1.534280E-03  -1.965932E-04  -1.082552E-03
 10    C11    -1.931759E-03  -9.908874E-04   6.215361E-04
 11    H7     -1.775814E-04   1.464387E-04   1.739668E-04
 12    C9      7.213503E-04  -1.263375E-03  -1.016812E-03
 13    H8      1.282530E-04  -1.587411E-04   7.876069E-06
 14    C10    -2.470540E-04   1.260490E-03   7.692993E-04
 15    C12     1.453405E-03   1.325890E-03  -3.933628E-03
 16    C2     -1.019141E-03  -2.748754E-03  -8.869362E-04
 17    H13     4.139215E-03   1.790964E-04   9.518011E-04
 18    C14    -1.890313E-03   5.889275E-04  -2.220388E-03
 19    H15    -5.789694E-04  -2.664568E-03   1.096563E-03
 20    C15    -1.086856E-03  -8.299807E-04   2.186525E-03
 21    C16    -5.200446E-03  -1.943274E-03   4.012439E-03
 22    C17     1.011256E-03  -1.508706E-04   6.361840E-04
```

```
  23    C18    -3.123879E-03  -7.158290E-04  -6.583892E-03
  24    C19    -7.063939E-03  -2.062728E-03   8.090705E-04
  25    H16    -2.103718E-04   4.707942E-04   8.553744E-04
  26    H17     5.839874E-03   2.613085E-03  -4.175018E-03
  27    H18     1.242488E-02   3.786442E-03   1.561713E-03
  28    H19    -3.859295E-03  -1.242690E-03   3.275097E-03
  29    H20     2.965080E-04  -4.598800E-04   1.965477E-03
  30    C1      1.066212E-04  -1.197430E-03   2.415771E-04
  31    H5     -6.245271E-04  -2.326934E-04   4.699059E-05
  32    H6     -9.647908E-05   1.200227E-04   3.096240E-06
  33    C3      2.194370E-03   2.154896E-03   1.522594E-03
  34    C26     2.819150E-03   2.857859E-03  -7.306075E-04
  35    C27    -1.348596E-04  -3.131549E-04  -4.776516E-04
  36    C28    -2.416145E-03  -2.441204E-03  -9.400507E-04
  37    C29    -1.201260E-03  -6.874415E-04   9.218558E-06
  38    H1      7.430163E-04   7.420055E-04   4.313428E-03
  39    H2     -2.921308E-03  -2.500431E-03  -3.182915E-03
  40    H3     -1.090762E-03  -7.223903E-04  -1.231965E-03
  41    H9      2.552610E-03   2.455616E-03   3.038091E-03
  42    H10     7.688328E-05   2.110463E-04   6.463850E-04
  43    H25     1.247264E-05   2.472986E-04   8.043307E-05
 -----------   ------------  ------------  ------------
    total       9.037392E-04   8.158031E-04  -7.771103E-05

  end of program der1b


  start of program geopt   6

  geometry optimization step  6
  reading input hessian of dimension   129
   in five columns format
  reading input hessian of dimension   129
   in five columns format


  Level shifts adjusted to satisfy step-size constraints
   Step size:    0.3005785
   Cos(theta):   0.4030506

   Final level shift:  -1.2859045E-02

  energy change:           5.6334E-04 .  (  5.0000E-05 )
  gradient maximum:        1.2826E-02 .  (  4.5000E-04 )
  gradient rms:            2.6475E-03 .  (  3.0000E-04 )
  step size:   0.30039  trust radius:   0.30000
  displacement maximum:    1.4866E-01 .  (  1.8000E-03 )
  displacement rms:        2.3601E-02 .  (  1.2000E-03 )
  predicted energy change:    -2.6208E-03    geom step:   3.0039E-
01    full step:   3.0039E-01
  molecular structure not yet converged...
```

```
center of mass moved by:
   x:    -7.0070E-03     y:     3.1695E-03     z:    -3.9849E-03

new geometry:
                              angstroms
atom            x                  y                  z
N1       -0.2431388835      0.4556736239      1.1723735471
N2       -1.0052221025     -2.2638064326      1.1711775865
C4       -1.2354149005     -0.0068382249      1.9686217617
C5       -0.0219368988     -1.8840112724      0.4769055945
C6       -1.6547168498     -1.3251930492      1.9985456785
C7       -1.7404984224      1.0066322943      2.7505185979
C13       0.6709126692     -2.8977538263     -0.4172186213
H4       -3.0356419670     -2.6439761971      2.9042709974
C8       -2.7930706857      0.6804961350      3.6197611198
C11      -0.9650101197      2.1686595222      2.4024960703
H7       -3.2425652691      1.4227454725      4.2536593268
C9       -3.2422765659     -0.6233719281      3.6535786031
H8       -4.0458719809     -0.8841748627      4.3157805202
C10      -2.6766502685     -1.6346558742      2.8502873309
C12      -0.0710715297      1.7970748741      1.4546947917
C2        0.9503376395      2.6145402001      0.7646068978
H13       1.7459317870     -2.8218826796     -0.2973025660
C14       0.3175412762     -2.7210862854     -1.8808548888
H15       0.3721363370     -3.8731171893     -0.0645970713
C15      -0.4054882560     -2.4055350093     -4.5510673450
C16       1.2049840844     -2.1961843098     -2.8003720449
C17      -0.9385119890     -3.0863025612     -2.3151953733
C18      -1.2985482642     -2.9334100012     -3.6363835117
C19       0.8468309784     -2.0394783637     -4.1317416864
H16       2.1814549757     -1.8985296468     -2.4799685659
H17      -1.6351318329     -3.4951433926     -1.6093012692
H18      -2.2805077802     -3.2390864388     -3.9568211189
H19       1.5442219641     -1.6284067831     -4.8322649240
H20      -0.6918667664     -2.2895583892     -5.5817584789
C1        0.5775477845     -0.4792363854      0.4348181138
H5        1.5772891761     -0.5317014840      0.8498355316
H6        0.6798600143     -0.1840634649     -0.5932120927
C3        2.8710077594      4.1851297153     -0.5136427992
C26       1.9198910499      3.2990087678      1.5101069134
C27       0.9496482049      2.7305127321     -0.6306199707
C28       1.9063251470      3.5077111519     -1.2645504751
C29       2.8739200353      4.0827381339      0.8755772877
H1        1.9381200101      3.2016452634      2.5905377595
H2        0.1888699186      2.2286821548     -1.2204008413
H3        1.8906486427      3.5909148714     -2.3446780704
H9        3.6213149051      4.5998757976      1.4699197044
H10       3.6131544908      4.7887646367     -1.0058448436
H25      -1.0749029726      3.1646160247      2.7777097600

nuclear repulsion energy.......  1972.328492632 hartrees
------------------------------------------------
```

```
 /  end of geometry optimization iteration   6   /
 ------------------------------------------------

  end of program geopt


  start of program onee
  smallest eigenvalue of S:    2.771E-04
  number of canonical orbitals.....          461
  end of program onee


  start of program probe
  end of program probe


  start of program grid

  number of gridpoints:
     atom         N1     N2      C4      C5      C6      C7     C13
H4
  grid # 1        95    102      85      88      89      88      84
73
  grid # 2       103    112      95      95      97      96      92
118
  grid # 3       212    227     194     188     190     198     164
224
  grid # 4       387    415     320     320     327     345     300
223

  number of gridpoints:
     atom         C8    C11      H7      C9      H8     C10     C12
C2
  grid # 1        89     86      73      87      73      89      86
92
  grid # 2        97     94     118      97     118      97      94
100
  grid # 3       184    185     223     184     222     184     193
196
  grid # 4       330    330     226     329     223     326     316
343

  number of gridpoints:
     atom        H13    C14     H15     C15     C16     C17     C18
C19
  grid # 1        69     93      69      89      88      87      89
89
  grid # 2       110    101     109      97      96      96      97
96
  grid # 3       211    191     213     182     183     182     184
182
```

```
  grid # 4      213    338    212    327    327    327    327
327

  number of gridpoints:
     atom        H16    H17    H18    H19    H20     C1     H5
H6
  grid # 1        72     72     73     73     73     82     70
69
  grid # 2       113    115    118    118    118     87    108
103
  grid # 3       214    215    222    221    222    162    217
208
  grid # 4       214    215    224    223    224    290    215
205

  number of gridpoints:
     atom         C3    C26    C27    C28    C29     H1     H2
H3
  grid # 1        89     89     88     89     89     73     71
73
  grid # 2        97     95     96     97     97    115    115
118
  grid # 3       186    184    185    185    185    218    218
222
  grid # 4       331    328    330    328    329    219    215
222

  number of gridpoints:
     atom         H9    H10    H25  total
  grid # 1        73     73     73   3516
  grid # 2       118    118    115   4486
  grid # 3       223    224    221   8628
  grid # 4       224    224    224  12242

  end of program grid


  start of program rwr
  end of program rwr


  start of program scf

       i  u  d  i  g
       t  p  i  c  r                                      RMS    maximum
       e  d  i  u  i                          energy  density   DIIS
       r  t  s  t  d     total energy         change  change    error

etot   1  N  N  2  U  -990.31135910434              4.3E-04  4.0E-02
etot   2  Y  Y  6  M  -990.37781182905  6.6E-02  2.3E-04  1.6E-02
etot   3  N  Y  2  U  -990.38806340056  1.0E-02  6.8E-05  3.7E-03
etot   4  Y  Y  6  M  -990.38873042054  6.7E-04  2.3E-05  1.0E-03
```

```
etot   5  Y  Y  6  M  -990.38880803844  7.8E-05  9.7E-06  2.1E-04
etot   6  N  Y  2  U  -990.38880825493  2.2E-07  3.3E-06  4.7E-05
etot   7  Y  N  6  M  -990.38881016479  1.9E-06  0.0E+00  0.0E+00


 Energy components, in hartrees:
   (A)  Nuclear repulsion............  1972.32849263240
   (E)  Total one-electron terms..... -5262.93232379671
   (I)  Total two-electron terms.....  2300.21502099953
   (L)  Electronic energy............ -2962.71730279719  (E+I)
   (N)  Total energy.................  -990.38881016479  (A+L)

 SCFE: SCF energy: HF    -990.38881016479 hartrees   iterations:
7


 HOMO energy:    -0.26932
 LUMO energy:     0.09840

  Orbital energies:
  -15.60367  -15.56424  -11.29601  -11.28477  -11.26958  -11.25339
  -11.24904  -11.24380  -11.24330  -11.24251  -11.24217  -11.24206
  -11.24138  -11.24062  -11.23899  -11.23339  -11.23226  -11.23184
  -11.22993  -11.22656  -11.22352  -11.21808  -11.21636  -11.21470
  -11.21091   -1.32713   -1.24880   -1.16550   -1.15964   -1.13970
   -1.07482   -1.06839   -1.03820   -1.02143   -1.02048   -1.01816
   -0.97093   -0.95526   -0.93500   -0.86046   -0.83462   -0.83327
   -0.83082   -0.80788   -0.79619   -0.76992   -0.73305   -0.71766
   -0.70058   -0.69957   -0.66673   -0.65810   -0.64423   -0.63655
   -0.63289   -0.62318   -0.62001   -0.61738   -0.59598   -0.59206
   -0.59102   -0.58737   -0.58058   -0.55931   -0.55282   -0.54918
   -0.54135   -0.51709   -0.50650   -0.50245   -0.50066   -0.49748
   -0.49337   -0.49087   -0.48836   -0.48092   -0.42887   -0.40467
   -0.36567   -0.34214   -0.34177   -0.33713   -0.33161   -0.28300
   -0.26932    0.09840    0.12812    0.13581    0.14035    0.14932
    0.17951    0.20872    0.22741    0.23483    0.24185

  end of program scf


  start of program der1a
  end of program der1a


  start of program rwr
  end of program rwr


  start of program der1b

  forces (hartrees/bohr) : total
```

atom	label	x	y	z
1	N1	2.996888E-03	-1.092300E-03	-5.075419E-03
2	N2	2.902098E-03	9.995089E-05	-2.104699E-03
3	C4	5.384872E-04	-5.237104E-03	-1.331028E-03
4	C5	-2.465605E-03	-1.986060E-03	6.611523E-04
5	C6	4.699496E-03	-2.649344E-03	-4.655830E-03
6	C7	-3.021900E-03	4.173873E-03	3.348288E-03
7	C13	1.016495E-03	3.817286E-03	3.204766E-03
8	H4	-8.333256E-04	-1.623874E-03	3.098643E-04
9	C8	-2.602591E-03	-3.503070E-03	1.654229E-03
10	C11	-1.632059E-03	4.019113E-03	2.604804E-03
11	H7	-3.480026E-04	8.474685E-04	4.877169E-04
12	C9	2.348395E-04	3.465178E-03	6.491069E-04
13	H8	-1.358836E-03	-2.713855E-04	1.230370E-03
14	C10	-2.396643E-03	4.501606E-04	2.552408E-03
15	C12	4.320330E-03	-4.826731E-04	-1.817665E-03
16	C2	4.569580E-03	4.725283E-03	-7.136536E-05
17	H13	2.265698E-03	1.215287E-03	1.281866E-03
18	C14	4.469088E-03	5.048013E-04	5.703955E-03
19	H15	-8.762473E-04	-2.464998E-03	-1.072243E-03
20	C15	-8.066367E-03	-2.185489E-03	-6.728433E-03
21	C16	3.580633E-03	2.454246E-03	-1.704632E-03
22	C17	-1.006307E-02	-3.935131E-03	-1.505949E-03
23	C18	-8.697968E-03	-3.668085E-03	-2.236322E-03
24	C19	7.507799E-03	2.895454E-03	4.149845E-03
25	H16	5.818461E-03	8.205617E-04	1.129787E-03
26	H17	-2.640653E-03	-8.339735E-04	1.123124E-03
27	H18	3.887211E-04	9.312185E-04	7.660585E-05
28	H19	3.923565E-03	1.582982E-03	-1.724279E-03
29	H20	2.529764E-05	3.214141E-05	1.544815E-05
30	C1	-2.579334E-03	-1.237971E-03	4.679537E-03
31	H5	3.899026E-03	3.218813E-04	1.595166E-03
32	H6	1.944755E-04	2.244129E-03	-6.101903E-03
33	C3	-4.312188E-03	-3.167459E-03	1.263702E-03
34	C26	-3.044044E-03	-3.147202E-03	-9.892063E-05
35	C27	-2.661653E-04	-6.060645E-04	2.329980E-03
36	C28	4.226828E-03	3.422783E-03	1.485505E-03
37	C29	3.350305E-04	-9.021500E-06	-3.264345E-03
38	H1	-2.026984E-03	-3.716618E-06	-7.193914E-03
39	H2	4.612104E-03	2.770042E-03	5.079830E-03
40	H3	1.355729E-03	4.917203E-04	5.686416E-03
41	H9	-4.759377E-03	-2.903258E-03	-5.357919E-03
42	H10	-3.207912E-04	-1.602736E-04	-6.972213E-04
43	H25	-5.459823E-04	8.909836E-04	6.012465E-04
total		1.022540E-03	1.008090E-03	1.626298E-04

end of program der1b

```
start of program geopt   7

geometry optimization step  7
reading input hessian of dimension   129
 in five columns format
reading input hessian of dimension   129
 in five columns format
reading input hessian of dimension   129
 in five columns format


Level shifts adjusted to satisfy step-size constraints
 Step size:    0.3001756
 Cos(theta):   0.3481846

 Final level shift:  -5.8225096E-03

energy change:           7.6693E-04 .  (  5.0000E-05 )
gradient maximum:        1.7603E-02 .  (  4.5000E-04 )
gradient rms:            3.4045E-03 .  (  3.0000E-04 )
step size:   0.29981  trust radius:   0.30000
displacement maximum:    1.6540E-01 .  (  1.8000E-03 )
displacement rms:        2.3556E-02 .  (  1.2000E-03 )
predicted energy change:    -2.5296E-03    geom step:   2.9981E-
01    full step:   2.9981E-01
molecular structure not yet converged...

center of mass moved by:
   x:    -3.4490E-03     y:    -8.1566E-03     z:     4.8314E-03

new geometry:
                              angstroms
atom           x                 y                 z
N1       -0.2623089590      0.4311251804      1.1716991713
N2       -1.0180143788     -2.2950059180      1.2042298173
C4       -1.2360309063     -0.0332939736      1.9960257998
C5       -0.0491760452     -1.9161106272      0.4898652496
C6       -1.6476617970     -1.3546394568      2.0462415395
C7       -1.7230911186      0.9831467707      2.7922374034
C13       0.6280306553     -2.9273737531     -0.4121209218
H4       -2.9937724088     -2.6736769475      3.0115780294
C8       -2.7457092522      0.6539618582      3.6975262155
C11      -0.9667588878      2.1493399444      2.4114356404
H7       -3.1781071145      1.3980009698      4.3414501000
C9       -3.1860027373     -0.6506742671      3.7535912964
H8       -3.9650269465     -0.9130115590      4.4450494557
C10      -2.6416558807     -1.6625876251      2.9369875099
C12      -0.0857743078      1.7755955971      1.4454283139
C2        0.9164503009      2.6009974180      0.7343857052
H13       1.7123800183     -2.8842131896     -0.2571412082
C14       0.3088978040     -2.7056385440     -1.8863538501
H15       0.2866736531     -3.9117867885     -0.0961463804
```

```
 C15     -0.2973622557    -2.3040914497    -4.5907098684
 C16      1.3065628895    -2.3700351452    -2.7900211464
 C17     -0.9983945440    -2.8282694144    -2.3525013671
 C18     -1.2991393561    -2.6286610044    -3.6904363154
 C19      1.0036021505    -2.1732391689    -4.1341691423
 H16      2.3304900741    -2.2499797277    -2.4490130079
 H17     -1.7797248649    -3.0692236040    -1.6584418267
 H18     -2.3091793966    -2.7128159017    -4.0314102359
 H19      1.7931017057    -1.9116780263    -4.8188293061
 H20     -0.5306398208    -2.1492911389    -5.6298028759
 C1       0.5501654299    -0.5106191206     0.4295419250
 H5       1.5613035628    -0.5554938071     0.8239873713
 H6       0.6303048976    -0.2234538860    -0.6093977434
 C3       2.8015088368     4.1881714110    -0.5810245152
 C26      1.8234927411     3.3659516372     1.4559907329
 C27      0.9590649824     2.6466221398    -0.6560625455
 C28      1.8988761714     3.4310895081    -1.3074873104
 C29      2.7564483373     4.1586935309     0.8023034085
 H1       1.8043237458     3.3261495512     2.5316351329
 H2       0.2432097047     2.0877393438    -1.2297362427
 H3       1.9178440967     3.4562601140    -2.3820556926
 H9       3.4514125005     4.7412314670     1.3756845720
 H10      3.5280434798     4.7976541261    -1.0885472588
 H25     -1.0762728724     3.1476511252     2.7825837903

 nuclear repulsion energy.......  1971.760476131 hartrees
 ------------------------------------------------
/  end of geometry optimization iteration   7   /
------------------------------------------------

 end of program geopt


 start of program onee
 smallest eigenvalue of S:    2.799E-04
 number of canonical orbitals.....          461
 end of program onee


 start of program probe
 end of program probe


 start of program grid

 number of gridpoints:
    atom        N1     N2      C4      C5      C6      C7     C13
H4
 grid # 1       96    102      87      88      89      88      84
73
 grid # 2      104    112      95      95      97      98      92
118
```

```
  grid # 3      214    227    195    187    190    199    164
224
  grid # 4      388    414    320    320    327    348    301
224

  number of gridpoints:
     atom         C8    C11     H7     C9     H8    C10    C12
C2
  grid # 1       89     86     73     87     73     89     84
92
  grid # 2       97     94    118     97    118     97     95
100
  grid # 3      184    185    223    184    222    184    195
195
  grid # 4      330    331    226    329    223    326    321
343

  number of gridpoints:
     atom        H13    C14    H15    C15    C16    C17    C18
C19
  grid # 1       69     91     69     89     88     87     89
88
  grid # 2      110    100    110     97     96     96     97
96
  grid # 3      211    194    215    183    183    183    184
183
  grid # 4      216    340    217    327    329    329    328
327

  number of gridpoints:
     atom        H16    H17    H18    H19    H20     C1     H5
H6
  grid # 1       72     73     73     73     73     82     70
69
  grid # 2      114    114    118    118    118     88    108
105
  grid # 3      214    216    222    222    223    164    217
207
  grid # 4      214    215    224    222    224    290    217
204

  number of gridpoints:
     atom         C3    C26    C27    C28    C29     H1     H2
H3
  grid # 1       89     88     88     89     89     72     71
73
  grid # 2       97     95     96     97     97    115    114
118
  grid # 3      184    182    184    184    184    217    214
222
  grid # 4      328    327    329    327    328    217    212
224
```

```
 number of gridpoints:
    atom         H9    H10    H25  total
 grid # 1        73     73     72   3512
 grid # 2       118    118    115   4492
 grid # 3       222    223    220   8629
 grid # 4       224    224    224  12258

 end of program grid


 start of program rwr
 end of program rwr


 start of program scf

      i  u  d  i  g
      t  p  i  c  r                                  RMS    maximum
      e  d  i  u  i                         energy  density   DIIS
      r  t  s  t  d      total energy       change  change    error

etot   1  N  N  2  U  -990.30270768203            4.6E-04  3.1E-02
etot   2  Y  Y  6  M  -990.37847298562  7.6E-02  2.4E-04  1.2E-02
etot   3  N  Y  2  U  -990.38974615112  1.1E-02  7.3E-05  3.0E-03
etot   4  Y  Y  6  M  -990.39043838018  6.9E-04  2.1E-05  7.2E-04
etot   5  Y  Y  6  M  -990.39051259464  7.4E-05  9.9E-06  1.8E-04
etot   6  N  Y  2  U  -990.39052152447  8.9E-06  3.4E-06  8.4E-05
etot   7  Y  N  6  M  -990.39052390496  2.4E-06  0.0E+00  0.0E+00


 Energy components, in hartrees:
   (A)  Nuclear repulsion............  1971.76047613143
   (E)  Total one-electron terms..... -5261.77830707936
   (I)  Total two-electron terms.....  2299.62730704297
   (L)  Electronic energy............ -2962.15100003639  (E+I)
   (N)  Total energy.................  -990.39052390496  (A+L)

 SCFE: SCF energy: HF    -990.39052390496 hartrees   iterations:
7


 HOMO energy:    -0.26911
 LUMO energy:     0.09715

  Orbital energies:
  -15.60451  -15.56524  -11.29616  -11.28590  -11.27118  -11.25063
  -11.25053  -11.24661  -11.24537  -11.24207  -11.23978  -11.23885
  -11.23840  -11.23770  -11.23689  -11.23687  -11.23575  -11.23433
  -11.23300  -11.23088  -11.22421  -11.21944  -11.21799  -11.21554
  -11.21243   -1.32546   -1.24901   -1.16385   -1.16049   -1.13916
   -1.07447   -1.06674   -1.04044   -1.02137   -1.01939   -1.01613
```

```
 -0.97215   -0.95419   -0.93387   -0.86020   -0.83767   -0.83371
 -0.82913   -0.80726   -0.79554   -0.77026   -0.73264   -0.71762
 -0.70168   -0.69844   -0.66645   -0.65787   -0.64274   -0.63728
 -0.63416   -0.62358   -0.62128   -0.61619   -0.59608   -0.59210
 -0.59055   -0.58653   -0.57915   -0.55835   -0.55307   -0.54811
 -0.54314   -0.51759   -0.50730   -0.50317   -0.50057   -0.49639
 -0.49350   -0.49125   -0.48704   -0.48151   -0.42958   -0.40467
 -0.36720   -0.34424   -0.34058   -0.33732   -0.32999   -0.28301
 -0.26911    0.09715    0.12930    0.13663    0.13689    0.14839
  0.17926    0.20825    0.22812    0.23474    0.24177

end of program scf


start of program der1a
end of program der1a


start of program rwr
end of program rwr


start of program der1b

forces (hartrees/bohr) : total

atom  label          x              y              z
----  -----   -------------  -------------  -------------
  1    N1      1.716334E-03  -1.943601E-03  -3.246573E-03
  2    N2      1.508741E-03   7.372569E-04  -8.449564E-04
  3    C4      1.732777E-03  -2.550928E-03  -1.144846E-04
  4    C5     -2.258395E-03  -1.157807E-03   1.364650E-03
  5    C6      3.010108E-03  -1.559524E-03  -2.676497E-03
  6    C7     -2.119800E-03   2.378055E-03   8.088183E-05
  7    C13     7.949520E-04  -3.166784E-03   1.193967E-03
  8    H4     -5.137608E-04  -1.262150E-03   5.166284E-05
  9    C8     -1.260196E-03  -1.009633E-03   7.377051E-04
 10    C11     1.581835E-03   1.944482E-03   1.100216E-03
 11    H7     -2.844006E-04   7.476233E-04   4.649418E-04
 12    C9      3.021764E-04   2.034644E-03   3.016599E-04
 13    H8     -9.191824E-04  -1.596108E-04   8.422822E-04
 14    C10    -2.885543E-04  -3.647628E-05   3.084839E-04
 15    C12    -1.002256E-04   1.528865E-04   8.546479E-05
 16    C2     -1.824596E-03  -8.798194E-04   7.008701E-05
 17    H13    -5.907107E-03   4.474195E-05  -1.786917E-03
 18    C14     2.117880E-03   2.467810E-04   1.794266E-03
 19    H15     1.613655E-03   3.598184E-03  -1.510856E-03
 20    C15     3.515496E-03   5.380807E-04   1.351633E-03
 21    C16     3.599045E-03   1.791191E-03  -2.863792E-03
 22    C17     6.384644E-04   6.449569E-04  -1.226921E-03
 23    C18     3.274344E-03   3.831445E-04   3.904487E-03
 24    C19     3.397778E-03   6.246062E-04   2.548436E-04
```

```
  25    H16    -6.033188E-03  -1.648501E-03  -2.241051E-03
  26    H17    -2.244134E-03  -1.119039E-03   1.633890E-03
  27    H18    -5.095790E-03  -1.462176E-03  -5.193377E-04
  28    H19    -7.132674E-04  -3.366359E-04   6.699522E-05
  29    H20     5.473843E-04   5.484700E-05   2.894166E-04
  30    C1     -2.121480E-03   9.323276E-04   7.055723E-04
  31    H5      1.935195E-03   3.195981E-04   1.225099E-03
  32    H6      1.335999E-04   6.505389E-04  -1.350591E-03
  33    C3      2.237467E-04   7.332118E-04   8.228264E-04
  34    C26     2.934216E-03   2.259092E-03   1.769155E-03
  35    C27     1.749225E-03   1.423862E-03  -2.428323E-03
  36    C28    -3.039822E-03  -2.449444E-03  -8.296198E-04
  37    C29    -1.534705E-03  -1.836397E-03   1.746854E-03
  38    H1      1.639685E-04   5.612689E-04  -9.703912E-04
  39    H2     -6.178164E-04  -7.198783E-04  -5.945158E-04
  40    H3     -2.726356E-04  -1.448398E-04  -7.139410E-04
  41    H9      1.428431E-03   1.266800E-03   1.288079E-03
  42    H10     1.793635E-04   8.168534E-05   1.338084E-04
  43    H25    -2.945761E-04   3.841792E-04   3.416161E-04
 -----------   -------------  -------------  -------------
    total       6.550833E-04   1.090803E-03   1.177413E-05

  end of program der1b


  start of program geopt   8

  geometry optimization step  8
  reading input hessian of dimension   129
   in five columns format
  reading input hessian of dimension   129
   in five columns format
  reading input hessian of dimension   129
   in five columns format


  Level shifts adjusted to satisfy step-size constraints
   Step size:    0.3001625
   Cos(theta):   0.3290489

   Final level shift:  -3.2712361E-03

  energy change:          -1.7137E-03 .  (  5.0000E-05 )
  gradient maximum:        6.5993E-03 .  (  4.5000E-04 )
  gradient rms:            1.5880E-03 .  (  3.0000E-04 )
  step size:   0.30014  trust radius:   0.30000
  displacement maximum:    1.3289E-01 .  (  1.8000E-03 )
  displacement rms:        2.3581E-02 .  (  1.2000E-03 )
  predicted energy change:    -1.1454E-03    geom step:   3.0014E-
01    full step:   3.0014E-01
  molecular structure not yet converged...
```

```
center of mass moved by:
   x:     1.4339E-03     y:     3.9888E-03     z:     1.3736E-03

new geometry:
                              angstroms
atom            x                  y                  z
N1       -0.2427301136      0.4133581705      1.1722777583
N2       -1.0454939928     -2.3112638158      1.1699656111
C4       -1.2161957562     -0.0597207232      2.0064760689
C5       -0.0696132821     -1.9370852449      0.4798595573
C6       -1.6544570973     -1.3757803348      2.0263648965
C7       -1.6847856453      0.9623542639      2.8316875226
C13       0.5944122868     -2.9384071474     -0.4387964649
H4       -3.0378534973     -2.6801972707      2.9787940568
C8       -2.7195283903      0.6400467042      3.7315827985
C11      -0.8978500352      2.1256728813      2.4741738095
H7       -3.1321827458      1.3838331907      4.3903874062
C9       -3.1856697575     -0.6615094865      3.7551713096
H8       -3.9748299789     -0.9205218030      4.4383849881
C10      -2.6599327111     -1.6753768988      2.9171144248
C12      -0.0449844390      1.7563976980      1.4791878795
C2        0.9261767262      2.6151140566      0.7518235711
H13       1.6652174605     -2.8719758706     -0.3024610873
C14       0.2876181649     -2.7034242241     -1.9136085293
H15       0.2724733448     -3.9275784756     -0.1477724137
C15      -0.2818249606     -2.3156026969     -4.6110105067
C16       1.3116920078     -2.5254385140     -2.8427232568
C17      -1.0214564856     -2.6804795857     -2.3448902825
C18      -1.3074367587     -2.4976491583     -3.6888738098
C19       1.0337227845     -2.3208152467     -4.1837294462
H16       2.3428974456     -2.5809231360     -2.5252940373
H17      -1.8180053491     -2.8390751217     -1.6358051292
H18      -2.3411635228     -2.5448754781     -4.0117834742
H19       1.8504547007     -2.2045671815     -4.8863974827
H20      -0.5093223464     -2.1903514419     -5.6550411487
C1        0.5725384418     -0.5359270224      0.4467237410
H5        1.5825600909     -0.6025572202      0.8639586010
H6        0.6849443477     -0.2439441492     -0.5977493705
C3        2.7427643680      4.2718109037     -0.5786007634
C26       1.7583262170      3.4655134300      1.4748050376
C27       1.0258000879      2.6093370968     -0.6475070512
C28       1.9286490508      3.4252015088     -1.3062477428
C29       2.6507103176      4.2926508665      0.8143808905
H1        1.7064457766      3.4685024593      2.5540630146
H2        0.3832676468      1.9769465517     -1.2258047756
H3        1.9981625157      3.3991783059     -2.3867378260
H9        3.2735304395      4.9523138350      1.3873712355
H10       3.4343293803      4.9068564617     -1.0932588436
H25      -0.9916197529      3.1137132967      2.8763314713

nuclear repulsion energy.......  1966.909747028 hartrees
------------------------------------------------
```

```
 /  end of geometry optimization iteration   8   /
 -------------------------------------------------

  end of program geopt


  start of program onee
  smallest eigenvalue of S:    2.927E-04
  number of canonical orbitals.....          461
  end of program onee


  start of program probe
  end of program probe


  start of program grid

  number of gridpoints:
     atom         N1     N2      C4      C5      C6      C7     C13
H4
  grid # 1        96    102      86      88      88      90      84
73
  grid # 2       104    112      94      95     101      98      92
118
  grid # 3       215    228     195     186     189     198     163
223
  grid # 4       388    413     323     321     327     351     300
224

  number of gridpoints:
     atom         C8    C11      H7      C9      H8     C10     C12
C2
  grid # 1        89     87      73      87      73      89      86
91
  grid # 2        97     94     118      97     118      97      95
100
  grid # 3       184    185     223     185     222     184     194
195
  grid # 4       329    331     226     331     224     328     318
343

  number of gridpoints:
     atom        H13    C14     H15     C15     C16     C17     C18
C19
  grid # 1        69     90      69      89      87      88      88
88
  grid # 2       107     98     111      97      96      96      97
97
  grid # 3       209    195     218     183     184     184     184
182
```

```
  grid # 4      207    339    213    329    329    330    331
330

  number of gridpoints:
     atom       H16    H17    H18    H19    H20     C1     H5
H6
  grid # 1       73     73     73     73     73     82     70
69
  grid # 2      114    115    118    118    118     90    110
105
  grid # 3      214    215    223    222    224    166    217
205
  grid # 4      213    215    223    222    224    293    220
207

  number of gridpoints:
     atom        C3    C26    C27    C28    C29     H1     H2
H3
  grid # 1       89     89     88     89     89     72     70
73
  grid # 2       97     96     96     97     97    115    113
118
  grid # 3      186    182    185    184    186    217    213
222
  grid # 4      330    327    328    327    330    217    210
224

  number of gridpoints:
     atom        H9    H10    H25  total
  grid # 1       73     73     72   3513
  grid # 2      118    118    115   4497
  grid # 3      223    223    219   8634
  grid # 4      223    223    222  12263

  end of program grid


  start of program rwr
  end of program rwr


  start of program scf

       i  u  d  i  g
       t  p  i  c  r                               RMS    maximum
       e  d  i  u  i                     energy  density   DIIS
       r  t  s  t  d     total energy    change  change    error

etot   1  N  N  2  U  -990.33991144147           3.6E-04  2.8E-02
etot   2  Y  Y  6  M  -990.38172607361  4.2E-02  1.8E-04  1.1E-02
etot   3  N  Y  2  U  -990.38813338998  6.4E-03  5.5E-05  2.7E-03
etot   4  Y  Y  6  M  -990.38854871108  4.2E-04  2.2E-05  8.5E-04
```

```
etot   5  Y  Y  6  M  -990.38862044730  7.2E-05  8.4E-06  1.6E-04
etot   6  N  Y  2  U  -990.38863299439  1.3E-05  3.2E-06  5.7E-05
etot   7  Y  N  6  M  -990.38863307052  7.6E-08  0.0E+00  0.0E+00


 Energy components, in hartrees:
   (A)  Nuclear repulsion............  1966.90974702809
   (E)  Total one-electron terms..... -5252.08828716800
   (I)  Total two-electron terms.....  2294.78990706939
   (L)  Electronic energy............ -2957.29838009861  (E+I)
   (N)  Total energy.................  -990.38863307052  (A+L)

 SCFE: SCF energy: HF    -990.38863307052 hartrees   iterations:
7


 HOMO energy:    -0.26918
 LUMO energy:     0.09483

  Orbital energies:
  -15.60546  -15.56553  -11.29617  -11.28888  -11.27440  -11.25323
  -11.25291  -11.24932  -11.24466  -11.23989  -11.23899  -11.23849
  -11.23820  -11.23781  -11.23695  -11.23662  -11.23581  -11.23470
  -11.23345  -11.22918  -11.22676  -11.22253  -11.22247  -11.21821
  -11.21580   -1.32205   -1.25283   -1.16216   -1.16049   -1.13639
   -1.07414   -1.06661   -1.03656   -1.02137   -1.01847   -1.01697
   -0.96944   -0.95479   -0.93227   -0.85933   -0.83776   -0.83195
   -0.82823   -0.80624   -0.79536   -0.76922   -0.73264   -0.71663
   -0.70030   -0.69768   -0.66473   -0.65767   -0.64239   -0.63624
   -0.63253   -0.62481   -0.62019   -0.61335   -0.59730   -0.59249
   -0.58848   -0.58801   -0.57615   -0.55734   -0.55154   -0.54848
   -0.54556   -0.51729   -0.50702   -0.50198   -0.49950   -0.49588
   -0.49449   -0.49099   -0.48779   -0.47966   -0.42997   -0.40493
   -0.36948   -0.34394   -0.33991   -0.33752   -0.32983   -0.28137
   -0.26918    0.09483    0.12818    0.13771    0.13869    0.14741
    0.17628    0.20896    0.22759    0.23471    0.23973

  end of program scf


  start of program der1a
  end of program der1a


  start of program rwr
  end of program rwr


  start of program der1b

  forces (hartrees/bohr) : total
```

atom	label	x	y	z
1	N1	-3.608445E-03	2.818146E-03	4.387853E-03
2	N2	-8.257060E-03	-1.291490E-03	6.873558E-03
3	C4	2.927401E-04	9.089283E-03	2.291217E-03
4	C5	1.159396E-02	4.274925E-03	-1.002984E-02
5	C6	-2.111425E-03	3.526585E-03	5.173623E-03
6	C7	3.146159E-03	-4.915515E-03	-4.361290E-03
7	C13	1.197140E-03	3.152175E-03	-4.853313E-03
8	H4	6.158857E-05	2.208153E-04	-3.754693E-04
9	C8	3.232912E-03	-4.388872E-03	-3.687738E-03
10	C11	-2.930359E-03	-7.516295E-03	-1.095850E-03
11	H7	-5.246514E-04	-5.339326E-04	3.142423E-05
12	C9	2.211024E-03	7.245822E-04	-2.076187E-03
13	H8	-9.756688E-05	-4.122068E-04	2.025975E-04
14	C10	2.685113E-04	4.837745E-03	1.404553E-03
15	C12	1.505465E-03	8.606128E-04	-3.784882E-03
16	C2	4.596320E-04	-4.228012E-03	-4.301454E-03
17	H13	4.376286E-03	-2.059499E-03	1.720359E-03
18	C14	1.184396E-02	2.108409E-03	5.874802E-03
19	H15	-6.450204E-04	-1.250607E-03	1.770200E-03
20	C15	-5.854112E-03	1.356985E-04	-5.815791E-04
21	C16	3.009072E-03	-1.664308E-03	4.118831E-03
22	C17	-8.790402E-03	-1.738000E-03	-6.298458E-03
23	C18	-4.719041E-03	-2.976537E-03	-2.898018E-03
24	C19	3.419037E-03	-2.514676E-03	-7.224967E-04
25	H16	-2.647010E-03	1.940020E-03	9.827487E-04
26	H17	7.504203E-04	1.171925E-03	-1.479661E-03
27	H18	6.000360E-03	3.165718E-03	-3.106612E-04
28	H19	-5.276927E-03	3.874991E-04	2.823860E-03
29	H20	-6.458594E-04	6.314977E-04	1.160412E-04
30	C1	4.753900E-04	-1.944511E-03	-4.062617E-03
31	H5	-3.832564E-03	-2.580860E-04	-2.786851E-04
32	H6	-7.710760E-04	-1.574150E-03	5.124757E-03
33	C3	-8.483867E-04	5.126358E-04	1.028467E-02
34	C26	-3.375860E-04	6.706250E-04	2.040083E-04
35	C27	-2.580503E-03	-2.382582E-03	7.317669E-03
36	C28	-4.311198E-04	-1.818641E-03	-1.976063E-03
37	C29	3.356317E-04	9.286614E-04	-7.586848E-03
38	H1	4.426202E-04	2.672569E-04	-4.159578E-03
39	H2	-3.082192E-03	-2.219428E-03	-4.993021E-04
40	H3	-1.537561E-03	6.603144E-05	5.377819E-03
41	H9	2.343965E-03	1.305606E-03	1.437853E-04
42	H10	3.434594E-03	3.036993E-03	3.538105E-04
43	H25	3.444813E-05	5.165315E-04	-1.111761E-03
total		9.060454E-04	6.626275E-04	4.644130E-05

end of program der1b

start of program geopt 9

```
geometry optimization step  9
reading input hessian of dimension   129
 in five columns format
reading input hessian of dimension   129
 in five columns format
reading input hessian of dimension   129
 in five columns format
** restarting optimization from step    8 **


Level shifts adjusted to satisfy step-size constraints
 Step size:    0.3007076
 Cos(theta):   0.3121536

 Final level shift:  -6.2152375E-03

energy change:           1.8908E-03 .  (  5.0000E-05 )
gradient maximum:        6.5993E-03 .  (  4.5000E-04 )
gradient rms:            1.5880E-03 .  (  3.0000E-04 )
step size:   0.29990  trust radius:   0.30000
displacement maximum:    1.3848E-01 .  (  1.8000E-03 )
displacement rms:        2.3562E-02 .  (  1.2000E-03 )
predicted energy change:    -1.2255E-03    geom step:   2.9990E-
01    full step:   2.9990E-01
molecular structure not yet converged...

center of mass moved by:
   x:    -7.4940E-16     y:     2.6368E-16     z:    -8.3267E-16

new geometry:
                               angstroms
atom            x                 y                 z
N1       -0.2112213224      0.4759821970      1.1509370987
N2       -0.9304654889     -2.2596315619      1.1557263606
C4       -1.2007566870     -0.0050996186      1.9468199390
C5        0.0308354325     -1.8599684763      0.4447847412
C6       -1.5893298383     -1.3330206181      1.9833821552
C7       -1.7559182331      1.0130893177      2.7060893243
C13       0.7195427103     -2.8544750054     -0.4654951884
H4       -2.9732939203     -2.6636627946      2.8871238361
C8       -2.8165407677      0.6740276669      3.5624798977
C11      -0.9876468060      2.1851605542      2.3748675911
H7       -3.2934365683      1.4196419607      4.1781118649
C9       -3.2309842879     -0.6335021660      3.6104914752
H8       -4.0426550097     -0.9068067006      4.2655024799
C10      -2.6247327918     -1.6447328748      2.8300957907
C12      -0.0663221250      1.8221726220      1.4414763503
C2        0.9615610706      2.6573510475      0.7929901780
H13       1.7804240263     -2.7291419038     -0.3977998181
C14       0.2904550036     -2.7194671495     -1.9078212042
H15       0.4874628272     -3.8353817165     -0.1033936907
```

```
  C15      -0.5195728220     -2.5100998311     -4.5600986738
  C16       1.1731723675     -2.2603581915     -2.8876463572
  C17      -1.0114333568     -3.0801698853     -2.2799362236
  C18      -1.4107209530     -2.9767515754     -3.5926183840
  C19       0.7731816258     -2.1547371391     -4.2060163333
  H16       2.1848694620     -1.9871622113     -2.6169976829
  H17      -1.6947814125     -3.4329355076     -1.5285602629
  H18      -2.3991425622     -3.2559857461     -3.8614828367
  H19       1.4638084288     -1.7938641349     -4.9522904025
  H20      -0.8298119621     -2.4271286947     -5.5831759553
  C1        0.6128319456     -0.4474688200      0.4042533197
  H5        1.6225608382     -0.4852424998      0.8284411699
  H6        0.7094088190     -0.1466392262     -0.6361783628
  C3        2.9218539781      4.2333037928     -0.3978184205
  C26       1.8547642164      3.3951618772      1.5732849714
  C27       1.0554555784      2.7250390584     -0.5942672449
  C28       2.0276025330      3.5107060596     -1.1848132236
  C29       2.8296541634      4.1778589369      0.9812369472
  H1        1.7838933136      3.3413965331      2.6388352230
  H2        0.3516514934      2.1776688845     -1.2016643395
  H3        2.0892544843      3.5634944072     -2.2542289962
  H9        3.5239664473      4.7363520807      1.5935527668
  H10       3.6885398335      4.8392355334     -0.8576710935
  H25      -1.1255993547      3.1803096411      2.7475409393

  nuclear repulsion energy.......  1969.045876041 hartrees
  -------------------------------------------------
 /  end of geometry optimization iteration   9   /
 -------------------------------------------------

  end of program geopt


  start of program onee
  smallest eigenvalue of S:    2.808E-04
  number of canonical orbitals.....          461
  end of program onee


  start of program probe
  end of program probe


  start of program grid

  number of gridpoints:
     atom         N1     N2     C4     C5     C6     C7    C13
H4
  grid # 1        95    102     84     87     89     90     84
73
  grid # 2       104    112     95     95    101     98     92
118
```

```
  grid # 3      212    227    193    187    192    198    163
224
  grid # 4      386    414    324    320    324    350    294
224

  number of gridpoints:
     atom        C8    C11     H7     C9     H8    C10    C12
C2
  grid # 1       89     86     73     87     73     89     86
90
  grid # 2       97     94    118     97    118     97     94
100
  grid # 3      184    184    223    184    222    184    195
194
  grid # 4      328    332    226    328    223    330    317
341

  number of gridpoints:
     atom       H13    C14    H15    C15    C16    C17    C18
C19
  grid # 1       69     91     69     89     88     87     89
89
  grid # 2      108    100    109     97     96     96     97
97
  grid # 3      211    196    214    186    184    182    183
184
  grid # 4      210    341    212    329    328    326    329
327

  number of gridpoints:
     atom       H16    H17    H18    H19    H20     C1     H5
H6
  grid # 1       72     72     73     73     73     82     70
69
  grid # 2      114    115    118    118    118     90    108
104
  grid # 3      213    215    223    222    223    164    217
206
  grid # 4      214    215    223    223    224    293    219
208

  number of gridpoints:
     atom        C3    C26    C27    C28    C29     H1     H2
H3
  grid # 1       89     87     87     89     89     72     71
73
  grid # 2       97     95     96     97     97    115    114
118
  grid # 3      185    181    182    184    185    217    213
223
  grid # 4      330    328    330    327    328    216    212
224
```

```
  number of gridpoints:
     atom        H9    H10    H25  total
  grid # 1       73     73     72   3507
  grid # 2      118    118    115   4495
  grid # 3      223    223    220   8625
  grid # 4      224    224    224  12249

  end of program grid


  start of program rwr
  end of program rwr


  start of program scf

       i  u  d  i  g
       t  p  i  c  r                                       RMS    maximum
       e  d  i  u  i                          energy  density   DIIS
       r  t  s  t  d     total energy        change  change    error

etot   1  N  N  2  U  -990.19143961399              6.9E-04  5.1E-02
etot   2  Y  Y  6  M  -990.36304368943  1.7E-01  3.6E-04  2.1E-02
etot   3  N  Y  2  U  -990.38756589331  2.5E-02  1.1E-04  4.9E-03
etot   4  Y  Y  6  M  -990.38902919237  1.5E-03  3.0E-05  1.2E-03
etot   5  Y  Y  6  M  -990.38919073874  1.6E-04  1.6E-05  3.2E-04
etot   6  N  Y  2  U  -990.38919602691  5.3E-06  5.3E-06  8.1E-05
etot   7  Y  Y  6  M  -990.38919546120 -5.7E-07  2.6E-06  3.1E-05
etot   8  Y  N  6  M  -990.38919647219  1.0E-06  0.0E+00  0.0E+00


 Energy components, in hartrees:
   (A)  Nuclear repulsion............  1969.04587604074
   (E)  Total one-electron terms..... -5256.35681247047
   (I)  Total two-electron terms.....  2296.92173995754
   (L)  Electronic energy............ -2959.43507251293  (E+I)
   (N)  Total energy.................  -990.38919647219  (A+L)

 SCFE: SCF energy: HF    -990.38919647219 hartrees   iterations:
8


 HOMO energy:    -0.26840
 LUMO energy:     0.09701

  Orbital energies:
  -15.60534  -15.56470  -11.29636  -11.28875  -11.27084  -11.25072
  -11.25052  -11.24617  -11.24576  -11.24057  -11.23986  -11.23892
  -11.23842  -11.23745  -11.23597  -11.23589  -11.23480  -11.23326
  -11.23202  -11.22987  -11.22361  -11.22021  -11.21809  -11.21556
  -11.21238   -1.32578   -1.25026   -1.16340   -1.15935   -1.13851
```

```
 -1.07486   -1.06779   -1.03915   -1.02304   -1.01954   -1.01789
 -0.96974   -0.95431   -0.93406   -0.86044   -0.83631   -0.83390
 -0.82845   -0.80772   -0.79588   -0.77036   -0.73270   -0.71782
 -0.70108   -0.69798   -0.66655   -0.65810   -0.64342   -0.63833
 -0.63434   -0.62250   -0.62043   -0.61751   -0.59675   -0.59349
 -0.59179   -0.58934   -0.57738   -0.55766   -0.55188   -0.54826
 -0.54421   -0.51763   -0.50773   -0.50313   -0.50021   -0.49540
 -0.49235   -0.49068   -0.48759   -0.48192   -0.42965   -0.40506
 -0.36691   -0.34326   -0.34192   -0.33901   -0.32575   -0.28176
 -0.26840    0.09701    0.12789    0.13689    0.13892    0.14831
  0.17873    0.20902    0.22650    0.23488    0.24146

end of program scf


start of program der1a
end of program der1a


start of program rwr
end of program rwr


start of program der1b

forces (hartrees/bohr) : total
```

atom	label	x	y	z
1	N1	-1.847904E-03	1.350870E-03	1.372186E-03
2	N2	8.004859E-04	-1.184322E-03	-1.290199E-03
3	C4	-2.990647E-03	1.894084E-03	-1.153339E-03
4	C5	-1.267694E-03	-1.405869E-03	2.168808E-03
5	C6	-3.781687E-03	1.063700E-03	1.466086E-03
6	C7	3.701565E-03	-2.358705E-03	9.208506E-04
7	C13	-6.358814E-03	5.686112E-03	1.117194E-03
8	H4	7.566979E-04	2.267632E-03	4.118942E-04
9	C8	3.487603E-04	4.660037E-03	1.792745E-03
10	C11	-4.761810E-04	-4.721753E-05	-2.385896E-03
11	H7	6.406881E-04	-9.788330E-04	-1.030604E-03
12	C9	-1.838024E-03	-6.112861E-03	1.573878E-04
13	H8	1.321968E-03	2.841587E-04	-1.158728E-03
14	C10	-9.988378E-04	-2.699473E-03	-2.337905E-04
15	C12	-3.644818E-04	5.292972E-04	1.985804E-03
16	C2	4.214487E-03	2.571180E-03	2.054306E-03
17	H13	1.220174E-02	2.129938E-03	9.413491E-04
18	C14	-1.971814E-03	-2.257252E-04	-1.000693E-02
19	H15	-2.739856E-03	-7.545521E-03	3.261445E-03
20	C15	-3.750255E-04	-1.032296E-03	2.979952E-03
21	C16	3.971553E-04	-1.694801E-03	1.180222E-02
22	C17	1.081296E-02	3.319375E-03	5.677630E-03
23	C18	1.275942E-02	5.331517E-03	-6.004976E-03

```
  24    C19    -6.135291E-03  -1.513302E-04  -5.275360E-03
  25    H16    -3.575445E-03  -1.293270E-03   1.989000E-04
  26    H17    -2.282103E-04  -2.076355E-04   5.184705E-04
  27    H18    -1.003847E-02  -2.388316E-03  -4.271375E-03
  28    H19    -1.686569E-03  -1.077089E-03   1.228071E-03
  29    H20    -1.715323E-03  -1.767050E-04  -2.514137E-03
  30    C1      6.153916E-03   1.649965E-03   1.097077E-03
  31    H5     -3.027084E-03  -3.480673E-04  -3.342055E-03
  32    H6     -1.486703E-04  -3.556652E-04   2.982007E-03
  33    C3      1.936388E-06   2.107970E-04  -5.988076E-03
  34    C26    -4.239919E-03  -2.750183E-03  -4.310837E-03
  35    C27    -6.213987E-04  -7.833312E-05   4.417728E-04
  36    C28     4.704144E-03   3.029595E-03   2.767092E-03
  37    C29     2.147902E-03   1.746275E-03   9.650488E-04
  38    H1     -4.030953E-04  -8.930180E-04   4.530742E-03
  39    H2      1.885956E-03   1.855455E-03   5.925202E-04
  40    H3      5.723930E-04   3.930392E-04  -2.575663E-03
  41    H9     -2.986811E-03  -2.049155E-03  -1.829814E-03
  42    H10    -2.808888E-03  -1.906220E-03  -3.199569E-05
  43    H25     3.854478E-04  -1.625851E-04  -2.576043E-04
 -----------   -------------  -------------  -------------
    total       1.181474E-03   8.498306E-04  -2.298122E-04

  end of program der1b


  start of program geopt  10

  geometry optimization step 10
  [ turning on trust-radius adjustment ]
  reading input hessian of dimension   129
   in five columns format
  reading input hessian of dimension   129
   in five columns format
  reading input hessian of dimension   129
   in five columns format
  ** restarting optimization from step    8 **


  energy change:           1.3274E-03 .  (  5.0000E-05 )
  gradient maximum:        6.5993E-03 .  (  4.5000E-04 )
  gradient rms:            1.5880E-03 .  (  3.0000E-04 )
  step size:   0.12023  trust radius:   0.15000
  displacement maximum:    4.6109E-02 .  (  1.8000E-03 )
  displacement rms:        9.4462E-03 .  (  1.2000E-03 )
  predicted energy change:    -6.6309E-04    geom step:   1.2023E-
01    full step:   1.2023E-01
  molecular structure not yet converged...

  center of mass moved by:
     x:     5.8287E-16     y:     2.6368E-16     z:     5.5511E-17
```

new geometry:

atom	x	y	z
		angstroms	
N1	-0.2503467923	0.4313387940	1.1728975667
N2	-0.9922353568	-2.3008642645	1.2068479080
C4	-1.2177846926	-0.0412413961	2.0027340636
C5	-0.0328950456	-1.9152046665	0.4874677885
C6	-1.6207580303	-1.3658403732	2.0516318574
C7	-1.7256576704	0.9802469690	2.7898913510
C13	0.6345154182	-2.9213500262	-0.4256979858
H4	-2.9784638181	-2.6865579845	3.0093481473
C8	-2.7573880472	0.6486468955	3.6876420923
C11	-0.9686088796	2.1508712541	2.4150585939
H7	-3.2011472190	1.3958055134	4.3235415609
C9	-3.1880965526	-0.6579308626	3.7452763889
H8	-3.9761108933	-0.9234283427	4.4292771187
C10	-2.6259600579	-1.6723713900	2.9368264804
C12	-0.0840044705	1.7791562074	1.4502481760
C2	0.9094340782	2.6151448582	0.7396605597
H13	1.7117184108	-2.8786891597	-0.2812033952
C14	0.3106147337	-2.6930411706	-1.8952436050
H15	0.2973278585	-3.9045733242	-0.1220199652
C15	-0.3176308144	-2.3047012879	-4.5894802502
C16	1.3058774411	-2.4094783712	-2.8200443949
C17	-1.0094991762	-2.7875081323	-2.3386183614
C18	-1.3208168237	-2.5954695037	-3.6744560256
C19	0.9966496806	-2.2130779832	-4.1595138305
H16	2.3268560103	-2.3606962160	-2.4970780760
H17	-1.7899818400	-3.0122134213	-1.6310120439
H18	-2.3448732588	-2.6750402869	-4.0018765889
H19	1.7816422461	-2.0017592358	-4.8628995476
H20	-0.5601115265	-2.1562329340	-5.6282763720
C1	0.5677714222	-0.5074561645	0.4336493303
H5	1.5802783124	-0.5505269015	0.8402390685
H6	0.6558736629	-0.2175416456	-0.6088435807
C3	2.8070428603	4.1983759363	-0.5604794150
C26	1.7923657031	3.4110711813	1.4677798769
C27	0.9824585662	2.6308465889	-0.6519969273
C28	1.9254954757	3.4145041743	-1.2967484498
C29	2.7309857208	4.2007778211	0.8223821304
H1	1.7459445767	3.3977291757	2.5379712574
H2	0.2862125744	2.0466037166	-1.2287363733
H3	1.9713578203	3.4151297035	-2.3708451625
H9	3.4096713505	4.8073562604	1.3990774035
H10	3.5437855062	4.8042079101	-1.0617698905
H25	-1.0891245347	3.1494999185	2.7854402047

nuclear repulsion energy....... 1970.392406452 hartrees

/ end of geometry optimization iteration 10 /

```
  end of program geopt


  start of program onee
  smallest eigenvalue of S:    2.841E-04
  number of canonical orbitals.....          461
  end of program onee


  start of program probe
  end of program probe


  start of program grid

  number of gridpoints:
     atom        N1     N2     C4     C5     C6     C7    C13
H4
  grid # 1       96    102     87     88     90     89     84
73
  grid # 2      103    112     95     95     99     97     92
118
  grid # 3      213    227    195    187    191    200    163
224
  grid # 4      389    414    323    320    321    351    300
224

  number of gridpoints:
     atom        C8    C11     H7     C9     H8    C10    C12
C2
  grid # 1       89     86     73     87     73     89     86
92
  grid # 2       97     94    118     97    118     97     94
100
  grid # 3      184    185    223    184    222    184    195
195
  grid # 4      328    331    226    331    223    329    318
342

  number of gridpoints:
     atom       H13    C14    H15    C15    C16    C17    C18
C19
  grid # 1       69     91     69     89     88     88     89
89
  grid # 2      109    100    110     97     96     96     97
96
  grid # 3      210    197    216    183    184    183    184
183
  grid # 4      213    342    213    327    329    327    327
329

  number of gridpoints:
```

```
     atom        H16    H17    H18    H19    H20     C1     H5
H6
  grid # 1        73     73     73     73     73     82     70
69
  grid # 2       113    115    118    118    118     89    110
104
  grid # 3       214    216    223    222    224    165    217
207
  grid # 4       214    214    224    223    224    293    219
206

  number of gridpoints:
     atom         C3    C26    C27    C28    C29     H1     H2
H3
  grid # 1        89     88     88     89     89     72     71
73
  grid # 2        97     95     96     97     97    115    114
118
  grid # 3       184    182    184    184    185    217    212
223
  grid # 4       329    328    329    327    329    216    212
224

  number of gridpoints:
     atom         H9    H10    H25  total
  grid # 1        73     73     72   3519
  grid # 2       118    118    115   4492
  grid # 3       222    223    220   8636
  grid # 4       224    224    224  12260

  end of program grid


  start of program rwr
  end of program rwr


  start of program scf

       i  u  d  i  g
       t  p  i  c  r                                     RMS    maximum
       e  d  i  u  i                          energy  density   DIIS
       r  t  s  t  d     total energy        change   change    error

etot   1  N  N  2  U  -990.29802142608             4.7E-04  3.6E-02
etot   2  Y  Y  6  M  -990.37914076540  8.1E-02  2.4E-04  1.5E-02
etot   3  N  Y  2  U  -990.39011714517  1.1E-02  7.3E-05  3.6E-03
etot   4  Y  Y  6  M  -990.39075170470  6.3E-04  1.8E-05  8.3E-04
etot   5  Y  Y  6  M  -990.39080716088  5.5E-05  9.2E-06  2.4E-04
etot   6  N  Y  2  U  -990.39081625073  9.1E-06  3.2E-06  6.3E-05
etot   7  Y  N  6  M  -990.39082059817  4.3E-06  0.0E+00  0.0E+00
```

```
 Energy components, in hartrees:
   (A)  Nuclear repulsion............  1970.39240645220
   (E)  Total one-electron terms..... -5259.04946585476
   (I)  Total two-electron terms.....  2298.26623880439
   (L)  Electronic energy............ -2960.78322705037  (E+I)
   (N)  Total energy.................  -990.39082059817  (A+L)

 SCFE: SCF energy: HF    -990.39082059817 hartrees   iterations:
7


 HOMO energy:    -0.26884
 LUMO energy:     0.09625

  Orbital energies:
  -15.60520  -15.56542  -11.29634  -11.28757  -11.27197  -11.25137
  -11.25137  -11.24663  -11.24624  -11.24028  -11.24014  -11.23939
  -11.23864  -11.23804  -11.23717  -11.23626  -11.23525  -11.23435
  -11.23339  -11.23131  -11.22496  -11.22092  -11.21941  -11.21652
  -11.21351   -1.32467   -1.25038   -1.16266   -1.16009   -1.13785
   -1.07415   -1.06682   -1.03876   -1.02160   -1.01902   -1.01676
   -0.97049   -0.95423   -0.93360   -0.85986   -0.83666   -0.83382
   -0.82912   -0.80693   -0.79529   -0.76986   -0.73238   -0.71725
   -0.70084   -0.69840   -0.66614   -0.65779   -0.64278   -0.63724
   -0.63391   -0.62371   -0.62052   -0.61551   -0.59631   -0.59205
   -0.59042   -0.58760   -0.57799   -0.55757   -0.55200   -0.54823
   -0.54488   -0.51728   -0.50751   -0.50254   -0.49998   -0.49565
   -0.49359   -0.49112   -0.48733   -0.48118   -0.42972   -0.40500
   -0.36774   -0.34301   -0.34117   -0.33775   -0.32916   -0.28213
   -0.26884    0.09625    0.12876    0.13660    0.13709    0.14832
    0.17835    0.20865    0.22790    0.23513    0.24117

  end of program scf


  start of program der1a
  end of program der1a


  start of program rwr
  end of program rwr


  start of program der1b

  forces (hartrees/bohr) : total

 atom  label          x             y             z
 ----  -----  ------------- ------------- -------------
   1    N1      3.176707E-04  -7.109467E-05   1.405262E-05
   2    N2     -1.075429E-03  -2.083377E-04   7.417913E-04
```

```
  3    C4     -9.732657E-04   1.912845E-03   4.219490E-04
  4    C5      9.390608E-04  -3.118005E-04  -6.400202E-04
  5    C6     -1.872859E-03   6.288692E-04   1.567042E-03
  6    C7      8.574249E-04  -1.220477E-03  -4.534514E-04
  7    C13     4.990676E-05   2.619141E-04  -1.067732E-03
  8    H4      2.212391E-04   7.436348E-04   4.175358E-05
  9    C8      9.716663E-04   1.126067E-03  -2.443932E-04
 10    C11    -3.058807E-04  -1.016420E-03  -2.792513E-05
 11    H7      2.030616E-04  -3.912862E-04  -4.531555E-04
 12    C9      3.885238E-04  -1.817448E-03  -8.541092E-04
 13    H8      5.647280E-04   1.033803E-04  -4.236539E-04
 14    C10     1.787747E-04   1.629413E-04  -1.130833E-04
 15    C12    -2.993285E-04   9.293299E-04  -8.305882E-04
 16    C2      2.106919E-03   3.714443E-04   1.529857E-03
 17    H13    -8.453989E-06  -1.369249E-05  -3.866099E-04
 18    C14    -2.620975E-03  -8.478820E-04  -2.750261E-04
 19    H15     1.703486E-04   2.476269E-04   5.668823E-04
 20    C15     1.348587E-03   3.170487E-04  -3.143626E-04
 21    C16    -3.287963E-03  -8.576557E-04  -7.679380E-04
 22    C17     9.274778E-04   5.528706E-04   2.811386E-05
 23    C18    -2.653106E-04  -1.756854E-04   1.189448E-03
 24    C19    -2.378424E-03  -1.170097E-03   5.057107E-05
 25    H16     3.742774E-03   1.309982E-03   7.701638E-04
 26    H17     6.077310E-04   2.216130E-04  -3.997413E-04
 27    H18     1.487305E-03   2.292942E-04   4.715166E-04
 28    H19     5.016746E-04   5.351337E-04  -4.906618E-04
 29    H20     3.420834E-04  -6.617536E-05   7.707523E-04
 30    C1      1.310155E-03   1.015916E-03  -6.323303E-04
 31    H5     -1.427620E-03  -7.688246E-05  -7.480952E-04
 32    H6     -2.814998E-05  -3.196540E-04   2.202996E-03
 33    C3     -1.512850E-03  -7.323179E-04  -2.557105E-03
 34    C26    -1.040007E-03  -4.215451E-04  -2.994571E-03
 35    C27     2.310941E-04   6.193161E-04  -3.498692E-04
 36    C28     1.620128E-03   8.085342E-04   2.072791E-03
 37    C29     2.143792E-04  -9.341659E-05   4.237368E-04
 38    H1     -3.535759E-05  -1.816722E-04   3.011021E-03
 39    H2      6.170067E-04   4.683717E-04   1.307347E-04
 40    H3     -3.642736E-05   2.655682E-04  -5.930004E-04
 41    H9     -9.794912E-04  -8.053914E-04  -4.013531E-04
 42    H10    -1.162548E-03  -8.092648E-04   1.701950E-04
 43    H25     1.251612E-04  -4.338929E-04  -3.335687E-04
-----------   -------------  -------------  -------------
   total       7.345388E-04   7.896115E-04  -1.769761E-04

 end of program der1b


 start of program geopt  11

 geometry optimization step 11
 reading input hessian of dimension   129
  in five columns format
```

```
reading input hessian of dimension   129
 in five columns format
reading input hessian of dimension   129
 in five columns format


Level shifts adjusted to satisfy step-size constraints
 Step size:    0.0750415
 Cos(theta):   0.6886741

 Final level shift:  -2.7962975E-02

energy change:          -2.9669E-04 .  (  5.0000E-05 )
gradient maximum:        3.8324E-03 .  (  4.5000E-04 )
gradient rms:            8.9140E-04 .  (  3.0000E-04 )
step size:   0.07504  trust radius:   0.07500
displacement maximum:    2.2233E-02 .  (  1.8000E-03 )
displacement rms:        5.8958E-03 .  (  1.2000E-03 )
predicted energy change:    -3.7189E-04    geom step:   7.5041E-
02    full step:   7.5041E-02
molecular structure not yet converged...

center of mass moved by:
   x:    -1.1335E-03     y:    -2.5596E-03     z:    -2.5985E-03

new geometry:
                              angstroms
atom            x                 y                 z
N1       -0.2525386544      0.4310493893      1.1683995301
N2       -1.0138740281     -2.2939256517      1.1975942074
C4       -1.2252479678     -0.0341646614      1.9952733135
C5       -0.0436572270     -1.9157862179      0.4851284149
C6       -1.6413071326     -1.3541973725      2.0405928362
C7       -1.7145415536      0.9821590281      2.7923186325
C13       0.6288624322     -2.9244715472     -0.4211405469
H4       -2.9999644294     -2.6702187494      2.9946387869
C8       -2.7426672956      0.6546811637      3.6920735521
C11      -0.9497183169      2.1459198615      2.4208127750
H7       -3.1762743134      1.3992173904      4.3355281353
C9       -3.1879856910     -0.6483570844      3.7409436743
H8       -3.9728895093     -0.9095416004      4.4272694645
C10      -2.6429704192     -1.6600851796      2.9242590216
C12      -0.0716241692      1.7744356070      1.4512907646
C2        0.9269144697      2.6056206604      0.7453112864
H13       1.7074422886     -2.8817125614     -0.2753663900
C14       0.3076938336     -2.7045928872     -1.8905668910
H15       0.2923378719     -3.9050788522     -0.1052980764
C15      -0.3053244872     -2.3158522487     -4.5878771164
C16       1.3069751907     -2.4063165422     -2.8077883962
C17      -1.0079025385     -2.8063076055     -2.3422753578
C18      -1.3113854749     -2.6134522525     -3.6776129076
C19       1.0025465289     -2.2143970874     -4.1491423166
```

```
  H16       2.3309432563     -2.3188574629     -2.4786269976
  H17      -1.7917111891     -3.0332743005     -1.6406619581
  H18      -2.3295554842     -2.6925171880     -4.0106917005
  H19       1.7907633063     -1.9834667927     -4.8460183616
  H20      -0.5422240742     -2.1661899720     -5.6255384105
  C1        0.5644095491     -0.5120677085      0.4353968420
  H5        1.5708233822     -0.5629812851      0.8428439319
  H6        0.6581057935     -0.2218487346     -0.5998173568
  C3        2.7889171356      4.2151427700     -0.5660685035
  C26       1.8162112152      3.3890211368      1.4702869848
  C27       0.9775928654      2.6431062201     -0.6456130349
  C28       1.9045321762      3.4402657775     -1.2949648747
  C29       2.7395960663      4.1914658022      0.8181678154
  H1        1.7871549220      3.3588554939      2.5468422945
  H2        0.2728790876      2.0718618690     -1.2204641568
  H3        1.9264167737      3.4627840599     -2.3698183664
  H9        3.4212751180      4.7898184578      1.3932077598
  H10       3.5069237148      4.8342820442     -1.0716438394
  H25      -1.0577771051      3.1427356635      2.7959918279

  nuclear repulsion energy.......  1971.488097852 hartrees
  -------------------------------------------------
 /  end of geometry optimization iteration  11   /
 -------------------------------------------------

  end of program geopt


  start of program onee
  smallest eigenvalue of S:    2.800E-04
  number of canonical orbitals.....          461
  end of program onee


  start of program probe
  end of program probe


  start of program grid

  number of gridpoints:
     atom        N1     N2     C4     C5     C6     C7    C13
H4
  grid # 1       96    102     86     88     89     88     84
73
  grid # 2      103    112     95     95     98     98     92
118
  grid # 3      214    227    195    186    190    199    164
224
  grid # 4      389    414    322    321    325    347    300
224
```

```
  number of gridpoints:
     atom         C8     C11      H7      C9      H8     C10     C12
C2
  grid # 1        89      86      73      87      73      89      86
92
  grid # 2        97      94     118      97     118      97      94
100
  grid # 3       184     184     223     184     222     184     194
195
  grid # 4       330     331     226     329     223     326     317
343

  number of gridpoints:
     atom        H13     C14     H15     C15     C16     C17     C18
C19
  grid # 1        69      91      69      89      88      88      89
89
  grid # 2       110     100     110      97      96      96      97
96
  grid # 3       211     196     214     183     184     183     184
183
  grid # 4       214     340     215     327     327     327     327
327

  number of gridpoints:
     atom        H16     H17     H18     H19     H20      C1      H5
H6
  grid # 1        72      73      73      73      73      82      70
69
  grid # 2       114     115     118     118     118      88     110
104
  grid # 3       214     216     222     222     224     163     217
207
  grid # 4       214     214     224     223     224     292     217
202

  number of gridpoints:
     atom         C3     C26     C27     C28     C29      H1      H2
H3
  grid # 1        89      89      88      89      89      72      71
73
  grid # 2        97      95      96      97      97     115     114
118
  grid # 3       185     182     184     185     184     217     214
222
  grid # 4       328     327     331     327     328     217     212
224

  number of gridpoints:
     atom         H9     H10     H25   total
  grid # 1        73      73      72    3516
  grid # 2       118     118     115    4493
```

```
  grid # 3      223    223    220   8631
  grid # 4      224    224    224  12247

  end of program grid


  start of program rwr
  end of program rwr


  start of program scf

       i  u  d  i  g
       t  p  i  c  r                                  RMS    maximum
       e  d  i  u  i                        energy  density   DIIS
       r  t  s  t  d     total energy       change  change     error

etot   1  N  N  1  U  -990.38859773819              8.1E-05  3.6E-03
etot   2  Y  Y  4  M  -990.39048554750  1.9E-03  3.8E-05  1.4E-03
etot   3  Y  Y  4  M  -990.39075521604  2.7E-04  1.1E-05  3.8E-04
etot   4  N  Y  1  U  -990.39078219283  2.7E-05  6.5E-06  1.5E-04
etot   5  Y  Y  4  M  -990.39078684366  4.7E-06  1.8E-06  3.2E-05
etot   6  Y  N  4  M  -990.39078874833  1.9E-06  0.0E+00  0.0E+00


 Energy components, in hartrees:
   (A)  Nuclear repulsion............  1971.48809785247
   (E)  Total one-electron terms..... -5261.23708829039
   (I)  Total two-electron terms.....  2299.35820168958
   (L)  Electronic energy............ -2961.87888660080  (E+I)
   (N)  Total energy.................  -990.39078874833  (A+L)

 SCFE: SCF energy: HF    -990.39078874833 hartrees   iterations:
6


 HOMO energy:    -0.26900
 LUMO energy:     0.09681

  Orbital energies:
  -15.60450  -15.56520  -11.29613  -11.28583  -11.27124  -11.25060
  -11.25060  -11.24629  -11.24564  -11.24013  -11.23934  -11.23862
  -11.23812  -11.23741  -11.23665  -11.23611  -11.23489  -11.23442
  -11.23272  -11.23078  -11.22433  -11.21958  -11.21829  -11.21571
  -11.21257   -1.32518   -1.24924   -1.16401   -1.16055   -1.13914
   -1.07477   -1.06714   -1.04013   -1.02193   -1.01901   -1.01706
   -0.97170   -0.95432   -0.93398   -0.86039   -0.83766   -0.83356
   -0.82897   -0.80722   -0.79561   -0.77030   -0.73280   -0.71772
   -0.70154   -0.69847   -0.66672   -0.65798   -0.64297   -0.63737
   -0.63423   -0.62375   -0.62103   -0.61586   -0.59622   -0.59244
   -0.59059   -0.58747   -0.57852   -0.55830   -0.55278   -0.54840
   -0.54412   -0.51758   -0.50739   -0.50284   -0.50056   -0.49599
```

```
 -0.49355   -0.49122   -0.48722   -0.48146   -0.42973   -0.40470
 -0.36763   -0.34405   -0.34100   -0.33769   -0.32924   -0.28262
 -0.26900    0.09681    0.12911    0.13706    0.13737    0.14842
  0.17898    0.20870    0.22842    0.23520    0.24150

end of program scf


start of program der1a
end of program der1a


start of program rwr
end of program rwr


start of program der1b

forces (hartrees/bohr) : total
```

atom	label	x	y	z
1	N1	7.004627E-04	-5.345029E-04	-8.693736E-04
2	N2	1.325105E-03	4.490218E-04	-1.207276E-03
3	C4	6.466353E-04	-1.748891E-03	-5.276414E-04
4	C5	-1.583901E-03	-5.770104E-05	1.464054E-03
5	C6	2.273493E-03	-1.141576E-03	-2.212237E-03
6	C7	-6.504121E-04	1.484241E-03	1.389573E-04
7	C13	-1.049263E-04	-1.124955E-03	1.171673E-03
8	H4	-4.026407E-04	-9.691760E-04	-5.464798E-05
9	C8	-9.303924E-04	-6.391797E-04	4.551451E-04
10	C11	6.920090E-04	1.520404E-03	3.671516E-04
11	H7	-7.863954E-05	4.226999E-04	2.440591E-04
12	C9	-2.025401E-04	1.476436E-03	5.741089E-04
13	H8	-5.400360E-04	-1.340263E-04	4.904071E-04
14	C10	-7.800840E-05	-1.362858E-04	6.780081E-04
15	C12	-2.323231E-04	-3.858693E-04	8.130474E-04
16	C2	-1.650918E-03	-1.050059E-03	-4.870314E-04
17	H13	-1.249323E-03	3.298007E-04	-3.456024E-04
18	C14	3.372735E-04	3.524076E-05	-8.065477E-04
19	H15	3.415639E-04	3.736382E-04	-5.501722E-04
20	C15	-7.211408E-05	-4.291507E-04	1.165413E-03
21	C16	1.485377E-04	5.669822E-04	3.571922E-04
22	C17	9.294633E-04	4.954721E-05	8.698192E-04
23	C18	1.506381E-03	3.791037E-04	-2.957498E-04
24	C19	1.342766E-03	5.454351E-04	-6.709570E-04
25	H16	-1.552766E-03	-6.560598E-04	-2.441234E-04
26	H17	1.700841E-04	-9.802381E-05	1.903320E-04
27	H18	-1.365794E-03	-2.753192E-04	-2.401760E-04
28	H19	-6.056477E-04	-1.969351E-04	3.560983E-04
29	H20	-1.099160E-04	1.214393E-04	-7.279111E-04
30	C1	-1.851382E-03	-1.238949E-04	1.290145E-03

```
 31    H5      1.574782E-03   1.314021E-04   1.035441E-03
 32    H6      3.969414E-04   6.577154E-04  -2.503026E-03
 33    C3      1.115385E-03   1.071782E-03   1.756491E-03
 34    C26     1.717762E-03   1.497857E-03   2.251613E-03
 35    C27     3.012996E-04   7.204426E-04  -9.322043E-04
 36    C28    -1.972431E-03  -1.672589E-03  -1.659435E-03
 37    C29    -8.471681E-04  -7.644455E-04   8.396384E-04
 38    H1      2.871811E-04   2.205505E-04  -1.629586E-03
 39    H2     -7.152317E-04  -9.510178E-04  -5.023593E-04
 40    H3      1.101136E-05  -8.687548E-05  -4.894680E-04
 41    H9      1.039112E-03   8.927797E-04   5.902413E-04
 42    H10     7.541013E-04   6.428744E-04  -3.083269E-04
 43    H25    -2.161468E-04   5.428347E-04   2.594211E-04
-----------   -------------  -------------  -------------
   total       5.986926E-04   9.556962E-04   9.460540E-05

 end of program der1b


 start of program geopt  12

 geometry optimization step 12
 reading input hessian of dimension   129
  in five columns format
 reading input hessian of dimension   129
  in five columns format


 Level shifts adjusted to satisfy step-size constraints
  Step size:    0.0381814
  Cos(theta):   0.6358626

  Final level shift:  -2.2947896E-02

 energy change:            3.1850E-05 *  (  5.0000E-05 )
 gradient maximum:         3.7159E-03 .  (  4.5000E-04 )
 gradient rms:             7.8551E-04 .  (  3.0000E-04 )
 step size:   0.03818  trust radius:   0.03750
 displacement maximum:     1.1555E-02 .  (  1.8000E-03 )
 displacement rms:         2.9998E-03 .  (  1.2000E-03 )
 predicted energy change:    -1.3809E-04    geom step:   3.8181E-
02    full step:   3.8181E-02
 molecular structure not yet converged...

 center of mass moved by:
    x:     4.5426E-04     y:     8.1147E-04     z:    -6.7642E-04

 new geometry:
                               angstroms
 atom            x                 y                 z
 N1      -0.2481100487      0.4334566292      1.1690590843
 N2      -1.0032005669     -2.2934581620      1.1949409475
```

```
 C4       -1.2219414340     -0.0343838020      1.9923563545
 C5       -0.0339796710     -1.9120602342      0.4839591872
 C6       -1.6327378826     -1.3563177585      2.0371288910
 C7       -1.7214706152      0.9842720805      2.7832559656
 C13       0.6387689584     -2.9205192316     -0.4241415240
 H4       -2.9960643356     -2.6736608684      2.9862523038
 C8       -2.7543266162      0.6556694707      3.6773710238
 C11      -0.9567330317      2.1503186557      2.4169640341
 H7       -3.1942470497      1.4021628828      4.3154141299
 C9       -3.1938710405     -0.6480229029      3.7284797792
 H8       -3.9830026483     -0.9107663027      4.4110346409
 C10      -2.6387220303     -1.6617110159      2.9180186584
 C12      -0.0739060768      1.7781436478      1.4520699323
 C2        0.9254724195      2.6102272532      0.7486369541
 H13       1.7151095547     -2.8707079086     -0.2840967415
 C14       0.3101739106     -2.7047976511     -1.8919547337
 H15       0.3108881878     -3.9021230759     -0.1082165064
 C15      -0.3249288630     -2.3285963028     -4.5840286299
 C16       1.2990880581     -2.4022115814     -2.8172990363
 C17      -1.0064246650     -2.8179513158     -2.3322450242
 C18      -1.3210023891     -2.6315174078     -3.6652839004
 C19       0.9856382061     -2.2157562838     -4.1570604658
 H16       2.3222336572     -2.3129300127     -2.4940718207
 H17      -1.7807423369     -3.0503320668     -1.6229685692
 H18      -2.3412419940     -2.7221754401     -3.9894950791
 H19       1.7641119017     -1.9818956103     -4.8615237321
 H20      -0.5712857690     -2.1834408899     -5.6215168996
 C1        0.5743863458     -0.5077215009      0.4405346425
 H5        1.5783628755     -0.5595077770      0.8633906677
 H6        0.6828551014     -0.2141085169     -0.5964581000
 C3        2.7976584351      4.2167944296     -0.5536768887
 C26       1.8195941604      3.3876491689      1.4793453416
 C27       0.9766033142      2.6525116204     -0.6445526402
 C28       1.9079513036      3.4480386227     -1.2902513713
 C29       2.7477220187      4.1880060271      0.8321252429
 H1        1.7898857173      3.3527087763      2.5543504826
 H2        0.2700912980      2.0817515541     -1.2208892319
 H3        1.9329210192      3.4750874837     -2.3661874570
 H9        3.4352847020      4.7809146017      1.4097776804
 H10       3.5220132279      4.8352318702     -1.0559521854
 H25      -1.0710358057      3.1480250996      2.7899724688

 nuclear repulsion energy.......  1971.062170283 hartrees
 -------------------------------------------------
/  end of geometry optimization iteration  12   /
-------------------------------------------------

 end of program geopt


 start of program onee
 smallest eigenvalue of S:    2.810E-04
```

```
  number of canonical orbitals.....          461
  end of program onee


  start of program probe
  end of program probe


  start of program grid

  number of gridpoints:
     atom        N1     N2     C4     C5     C6     C7    C13
H4
  grid # 1       95    102     87     87     90     89     84
73
  grid # 2      103    112     95     95     99     98     92
118
  grid # 3      214    227    195    187    191    199    163
224
  grid # 4      385    414    321    320    323    348    299
224

  number of gridpoints:
     atom        C8    C11     H7     C9     H8    C10    C12
C2
  grid # 1       89     86     73     87     73     89     86
92
  grid # 2       97     94    118     97    118     97     94
100
  grid # 3      184    185    223    184    222    184    194
195
  grid # 4      328    331    226    331    223    329    317
343

  number of gridpoints:
     atom       H13    C14    H15    C15    C16    C17    C18
C19
  grid # 1       69     91     69     89     88     87     89
89
  grid # 2      109    100    110     97     96     96     97
96
  grid # 3      211    196    214    183    184    182    184
183
  grid # 4      214    340    213    327    327    327    327
327

  number of gridpoints:
     atom       H16    H17    H18    H19    H20     C1     H5
H6
  grid # 1       72     73     73     73     73     82     70
69
```

```
  grid # 2      114    114    118    118    118     88    109
104
  grid # 3      214    216    222    221    224    164    217
207
  grid # 4      214    214    224    223    224    292    219
206

  number of gridpoints:
     atom         C3    C26    C27    C28    C29     H1     H2
H3
  grid # 1       89     88     88     89     89     72     71
73
  grid # 2       97     95     96     97     97    115    114
118
  grid # 3      185    182    184    185    185    217    213
223
  grid # 4      328    327    330    328    328    217    212
224

  number of gridpoints:
     atom         H9    H10    H25  total
  grid # 1       73     73     72   3515
  grid # 2      118    118    115   4491
  grid # 3      223    224    220   8634
  grid # 4      224    224    224  12246

  end of program grid


  start of program rwr
  end of program rwr


  start of program scf

       i  u  d  i  g
       t  p  i  c  r                                   RMS    maximum
       e  d  i  u  i                        energy  density   DIIS
       r  t  s  t  d     total energy       change  change    error

etot   1  N  N  1  U  -990.39028009303            4.3E-05  2.4E-03
etot   2  Y  Y  4  M  -990.39078089231  5.0E-04  1.8E-05  5.5E-04
etot   3  Y  Y  4  M  -990.39083518771  5.4E-05  4.9E-06  1.2E-04
etot   4  Y  N  4  M  -990.39084155577  6.4E-06  0.0E+00  0.0E+00


 Energy components, in hartrees:
   (A)  Nuclear repulsion............  1971.06217028341
   (E)  Total one-electron terms..... -5260.39454868744
   (I)  Total two-electron terms.....  2298.94153684826
   (L)  Electronic energy............ -2961.45301183918  (E+I)
   (N)  Total energy.................  -990.39084155577  (A+L)
```

```
 SCFE: SCF energy: HF    -990.39084155577 hartrees   iterations:
4


 HOMO energy:    -0.26906
 LUMO energy:     0.09673

  Orbital energies:
  -15.60462  -15.56496  -11.29644  -11.28704  -11.27124  -11.25104
  -11.25071  -11.24585  -11.24537  -11.24021  -11.23959  -11.23927
  -11.23874  -11.23808  -11.23742  -11.23573  -11.23438  -11.23415
  -11.23269  -11.23001  -11.22431  -11.22021  -11.21853  -11.21599
  -11.21287   -1.32532   -1.24973   -1.16308   -1.16069   -1.13877
   -1.07464   -1.06716   -1.03922   -1.02184   -1.01901   -1.01724
   -0.97091   -0.95440   -0.93382   -0.86015   -0.83698   -0.83354
   -0.82928   -0.80716   -0.79551   -0.77003   -0.73266   -0.71746
   -0.70102   -0.69855   -0.66633   -0.65789   -0.64293   -0.63722
   -0.63387   -0.62365   -0.62070   -0.61586   -0.59633   -0.59238
   -0.59043   -0.58757   -0.57842   -0.55802   -0.55233   -0.54821
   -0.54447   -0.51739   -0.50720   -0.50256   -0.50022   -0.49593
   -0.49341   -0.49120   -0.48742   -0.48125   -0.42975   -0.40484
   -0.36746   -0.34335   -0.34121   -0.33758   -0.32931   -0.28234
   -0.26906    0.09673    0.12890    0.13698    0.13758    0.14860
    0.17856    0.20869    0.22823    0.23521    0.24124

  end of program scf


  start of program der1a
  end of program der1a


  start of program rwr
  end of program rwr


  start of program der1b

  forces (hartrees/bohr) : total

 atom  label         x              y              z
 ----  -----   -------------  -------------  -------------
   1    N1      2.227998E-04   3.002457E-04  -7.633866E-04
   2    N2      8.313377E-04   6.278822E-05  -8.329915E-04
   3    C4     -2.749466E-04  -4.216903E-04  -5.263447E-07
   4    C5     -6.186970E-04  -7.144881E-04   3.670361E-04
   5    C6      2.786252E-04   3.974446E-05  -3.989161E-04
   6    C7      9.204291E-05   2.064961E-04   1.809267E-04
   7    C13    -2.647782E-04   7.202521E-04   7.337563E-04
   8    H4      4.755602E-05   2.153163E-04   1.133755E-04
   9    C8     -3.968861E-04   6.246385E-04   6.455230E-04
```

```
 10    C11      1.871446E-05   4.167742E-04  -1.449800E-04
 11    H7       4.661710E-05  -2.622866E-05   2.075457E-05
 12    C9      -4.135986E-04  -8.043030E-04   2.383556E-04
 13    H8       9.219046E-05   1.817128E-05  -3.487273E-05
 14    C10     -5.487297E-04  -5.031939E-04   3.007736E-04
 15    C12      4.545402E-04   3.326283E-05   3.764629E-06
 16    C2       3.716824E-04   4.474287E-04  -1.073648E-03
 17    H13      6.660705E-04   2.713936E-04  -4.580298E-05
 18    C14     -9.524221E-06  -1.503978E-05  -1.155393E-04
 19    H15     -2.589289E-04  -4.741291E-04   1.596626E-04
 20    C15     -3.296994E-04  -8.513451E-05  -3.637118E-04
 21    C16      5.059299E-04   2.273868E-04   1.343442E-04
 22    C17      1.784530E-04   6.295302E-06   3.790300E-04
 23    C18      4.746219E-04   1.823099E-04  -5.954331E-04
 24    C19     -1.022632E-04   1.979797E-04   6.156296E-05
 25    H16      3.316220E-04  -1.320570E-04   5.700000E-06
 26    H17     -4.687600E-04  -9.797374E-05   2.750276E-04
 27    H18     -1.205098E-03  -6.438215E-05  -3.681211E-04
 28    H19      4.370449E-04  -4.587109E-06  -2.768543E-05
 29    H20      1.076385E-05  -4.651129E-05   2.680799E-04
 30    C1       1.139672E-03   1.536346E-04   3.587566E-04
 31    H5      -4.346405E-04  -9.406025E-07  -3.907024E-04
 32    H6      -1.517462E-04  -9.902310E-05   3.357495E-04
 33    C3       1.185490E-04   2.269726E-04   5.102438E-04
 34    C26     -2.577180E-06   8.957851E-05  -5.609457E-05
 35    C27      1.261047E-04   2.099169E-04   7.317389E-04
 36    C28      2.097073E-04   1.577945E-04   1.716548E-04
 37    C29      3.597928E-05  -6.029637E-05  -9.796868E-04
 38    H1       9.699556E-05   7.995279E-05  -5.711064E-04
 39    H2       8.836115E-05  -5.681230E-05   5.451637E-05
 40    H3      -2.236567E-05  -8.217920E-05   5.481682E-04
 41    H9      -1.316816E-04   3.301862E-05  -1.580912E-04
 42    H10     -5.437240E-04  -3.859753E-04   3.434680E-04
 43    H25     -4.214022E-06  -3.049285E-05   3.941865E-06
-----------    -------------  -------------  -------------
   total        6.931218E-04   8.159137E-04   2.461458E-05

 end of program der1b


 start of program geopt  13

 geometry optimization step 13
 reading input hessian of dimension   129
  in five columns format
 reading input hessian of dimension   129
  in five columns format


 Level shifts adjusted to satisfy step-size constraints
  Step size:    0.0192086
  Cos(theta):   0.7007821
```

```
 Final level shift:  -6.0023224E-02

energy change:           -5.2807E-05 .  (  5.0000E-05 )
gradient maximum:         1.3227E-03 .  (  4.5000E-04 )
gradient rms:             3.7901E-04 .  (  3.0000E-04 )
step size:   0.01921  trust radius:   0.01875
displacement maximum:     6.9080E-03 .  (  1.8000E-03 )
displacement rms:         1.5092E-03 .  (  1.2000E-03 )
predicted energy change:    -4.3542E-05    geom step:   1.9209E-
02    full step:   1.9209E-02
molecular structure not yet converged...

center of mass moved by:
   x:    -2.9109E-04     y:     4.6312E-04     z:     3.9301E-04

new geometry:
                              angstroms
atom           x                  y                  z
N1       -0.2488255692      0.4322339843      1.1698872896
N2       -1.0058226391     -2.2950440448      1.1945544518
C4       -1.2229240041     -0.0361424909      1.9939013709
C5       -0.0365007616     -1.9141015496      0.4855354067
C6       -1.6347093114     -1.3580296563      2.0375911084
C7       -1.7199951420      0.9823189968      2.7870807318
C13       0.6348421100     -2.9216660268     -0.4233610698
H4       -2.9973932708     -2.6761090969      2.9877417657
C8       -2.7524946331      0.6532233934      3.6829849331
C11      -0.9547024201      2.1488365611      2.4190560905
H7       -3.1905897249      1.3985912480      4.3231430452
C9       -3.1932086574     -0.6517690880      3.7325641745
H8       -3.9812949477     -0.9149495298      4.4155551603
C10      -2.6401022769     -1.6646647698      2.9195236102
C12      -0.0728836370      1.7767980278      1.4534342970
C2        0.9248616477      2.6094547063      0.7465477863
H13       1.7116541383     -2.8719037968     -0.2842566294
C14       0.3090127646     -2.7017285669     -1.8919217197
H15       0.3057087418     -3.9036615388     -0.1088356875
C15      -0.3181517155     -2.3211174476     -4.5844573536
C16       1.3022806045     -2.4054300452     -2.8151364504
C17      -1.0074098438     -2.8056006886     -2.3339389563
C18      -1.3187367817     -2.6172398029     -3.6681293975
C19       0.9926701615     -2.2159193741     -4.1546871043
H16       2.3260666979     -2.3274211924     -2.4914155295
H17      -1.7860704955     -3.0349791182     -1.6266254226
H18      -2.3425831130     -2.7043182588     -3.9940814180
H19       1.7761371458     -1.9889515630     -4.8563274949
H20      -0.5618666539     -2.1757307323     -5.6219403179
C1        0.5740559454     -0.5100177170      0.4417086690
H5        1.5784350739     -0.5618273663      0.8611698811
H6        0.6798161828     -0.2186286872     -0.5959096950
C3        2.7946536701      4.2153968164     -0.5592303079
```

```
  C26       1.8165501131      3.3931684312      1.4740306347
  C27       0.9780419294      2.6451051479     -0.6454457865
  C28       1.9082602778      3.4400244963     -1.2927166344
  C29       2.7429412688      4.1934511473      0.8251154766
  H1        1.7882002850      3.3627714017      2.5482192846
  H2        0.2755486666      2.0683734653     -1.2209902818
  H3        1.9348576060      3.4600611274     -2.3678965230
  H9        3.4273881104      4.7918441078      1.4013512856
  H10       3.5166904418      4.8328913912     -1.0628356014
  H25      -1.0679447669      3.1461658281      2.7930681050

  nuclear repulsion energy.......  1971.091366916 hartrees
  -------------------------------------------------
 /  end of geometry optimization iteration  13   /
 -------------------------------------------------

  end of program geopt


  start of program onee
  smallest eigenvalue of S:    2.824E-04
  number of canonical orbitals.....          461
  end of program onee


  start of program probe
  end of program probe


  start of program grid

  number of gridpoints:
     atom        N1     N2      C4      C5      C6      C7     C13
H4
  grid # 1       96    102      87      87      90      90      84
73
  grid # 2      103    112      95      95      99      98      92
118
  grid # 3      214    227     195     187     191     199     163
224
  grid # 4      389    414     321     320     323     347     299
224

  number of gridpoints:
     atom        C8    C11      H7      C9      H8     C10     C12
C2
  grid # 1       89     86      73      87      73      89      86
92
  grid # 2       97     94     118      97     118      97      94
100
  grid # 3      184    185     223     184     222     184     194
196
```

```
  grid # 4      329    331    226    331    223    329    317
341

  number of gridpoints:
     atom        H13    C14    H15    C15    C16    C17    C18
C19
  grid # 1        69     93     69     89     88     88     89
89
  grid # 2       109    100    110     97     96     96     97
96
  grid # 3       210    195    214    183    184    183    184
183
  grid # 4       214    340    213    327    327    327    327
327

  number of gridpoints:
     atom        H16    H17    H18    H19    H20     C1     H5
H6
  grid # 1        72     73     73     73     73     82     70
69
  grid # 2       114    115    118    118    118     88    110
104
  grid # 3       214    216    223    222    224    164    217
207
  grid # 4       214    214    224    223    224    293    217
205

  number of gridpoints:
     atom         C3    C26    C27    C28    C29     H1     H2
H3
  grid # 1        89     88     88     89     89     72     71
73
  grid # 2        97     95     96     97     97    115    114
118
  grid # 3       185    182    184    184    185    217    213
223
  grid # 4       328    328    329    328    328    217    212
224

  number of gridpoints:
     atom         H9    H10    H25  total
  grid # 1        73     73     72   3520
  grid # 2       118    118    115   4493
  grid # 3       223    223    220   8634
  grid # 4       224    224    224  12246

  end of program grid


  start of program rwr
  end of program rwr
```

```
  start of program scf

       i  u  d  i  g
       t  p  i  c  r                                       RMS    maximum
       e  d  i  u  i                        energy  density   DIIS
       r  t  s  t  d     total energy     change  change     error

etot   1  N  N  1  U  -990.39066938504           2.3E-05  1.1E-03
etot   2  Y  Y  4  M  -990.39082701468  1.6E-04  1.1E-05  4.2E-04
etot   3  Y  Y  4  M  -990.39084593688  1.9E-05  3.2E-06  1.1E-04
etot   4  Y  N  4  M  -990.39084898231  3.0E-06  0.0E+00  0.0E+00


 Energy components, in hartrees:
   (A)  Nuclear repulsion............  1971.09136691580
   (E)  Total one-electron terms..... -5260.45116671076
   (I)  Total two-electron terms.....  2298.96895081265
   (L)  Electronic energy............ -2961.48221589810  (E+I)
   (N)  Total energy.................  -990.39084898231  (A+L)

 SCFE: SCF energy: HF    -990.39084898231 hartrees   iterations:
4


 HOMO energy:    -0.26909
 LUMO energy:     0.09670

  Orbital energies:
  -15.60482  -15.56511  -11.29607  -11.28679  -11.27163  -11.25115
  -11.25090  -11.24617  -11.24561  -11.23984  -11.23956  -11.23889
  -11.23843  -11.23775  -11.23701  -11.23573  -11.23460  -11.23444
  -11.23276  -11.23041  -11.22479  -11.22011  -11.21902  -11.21611
  -11.21311   -1.32505   -1.25012   -1.16329   -1.16072   -1.13844
   -1.07455   -1.06701   -1.03926   -1.02193   -1.01882   -1.01722
   -0.97108   -0.95429   -0.93372   -0.86021   -0.83691   -0.83360
   -0.82903   -0.80715   -0.79555   -0.77004   -0.73267   -0.71741
   -0.70115   -0.69841   -0.66628   -0.65791   -0.64291   -0.63702
   -0.63380   -0.62376   -0.62069   -0.61579   -0.59625   -0.59229
   -0.59055   -0.58752   -0.57825   -0.55807   -0.55233   -0.54821
   -0.54447   -0.51744   -0.50724   -0.50258   -0.50023   -0.49595
   -0.49354   -0.49120   -0.48746   -0.48116   -0.42969   -0.40485
   -0.36758   -0.34333   -0.34129   -0.33767   -0.32932   -0.28244
   -0.26909    0.09670    0.12895    0.13716    0.13754    0.14851
    0.17843    0.20872    0.22830    0.23522    0.24111

  end of program scf


  start of program der1a
  end of program der1a
```

```
start of program rwr
end of program rwr


start of program der1b

forces (hartrees/bohr) : total
```

atom	label	x	y	z
1	N1	4.528739E-04	-2.150721E-04	-7.189734E-04
2	N2	-5.179471E-04	-3.795031E-05	3.540438E-04
3	C4	1.453513E-04	-6.415916E-05	2.186439E-05
4	C5	7.956954E-04	-7.125645E-05	-6.055630E-04
5	C6	1.674864E-04	-8.425208E-05	-1.081652E-04
6	C7	-2.512909E-04	2.681225E-04	1.706745E-04
7	C13	2.264142E-04	5.762714E-04	8.823663E-05
8	H4	-6.045948E-05	-1.622022E-04	5.346281E-05
9	C8	-9.811810E-06	-6.163186E-04	-1.033681E-04
10	C11	-3.140914E-04	-5.043245E-07	3.741302E-04
11	H7	-7.779658E-05	9.048357E-05	3.931211E-05
12	C9	2.767482E-04	5.658957E-04	-1.033924E-04
13	H8	-1.429809E-04	-1.901601E-05	1.548469E-04
14	C10	-7.750078E-05	3.688975E-04	2.521305E-04
15	C12	4.616740E-04	5.257927E-06	-2.829912E-04
16	C2	2.944823E-04	2.233180E-04	5.568139E-04
17	H13	4.427948E-04	6.578201E-05	1.726383E-04
18	C14	1.282378E-04	-1.306465E-04	5.130233E-04
19	H15	-1.131664E-04	-2.810631E-04	-3.533440E-05
20	C15	-5.253545E-04	1.215568E-05	-8.190267E-05
21	C16	1.228580E-04	-7.128547E-05	5.101624E-04
22	C17	-7.451867E-04	-2.526086E-04	2.209580E-04
23	C18	-1.326991E-03	-2.850276E-04	-7.335060E-04
24	C19	-1.692902E-04	6.245857E-06	-3.167455E-04
25	H16	3.328469E-04	1.496603E-04	2.129530E-04
26	H17	3.554518E-04	1.287242E-04	-1.716810E-04
27	H18	1.486137E-03	2.577115E-04	2.009026E-04
28	H19	1.714175E-05	8.756748E-05	-1.598401E-04
29	H20	3.361176E-05	4.889786E-05	-2.258726E-04
30	C1	-2.352300E-04	1.325569E-04	1.607766E-04
31	H5	-2.663504E-05	8.592286E-06	1.148176E-04
32	H6	-4.109229E-05	8.430998E-05	1.882357E-04
33	C3	-4.420181E-04	-1.741977E-04	-2.983395E-04
34	C26	-2.316693E-04	-2.309980E-04	-1.002232E-04
35	C27	-8.113392E-05	-3.491728E-05	-1.261326E-04
36	C28	2.378795E-04	1.576131E-04	7.887497E-05
37	C29	1.155601E-04	5.628839E-05	2.498595E-04
38	H1	-1.391838E-04	-3.540240E-05	1.268414E-04
39	H2	6.131107E-05	1.420514E-04	1.187152E-04
40	H3	5.722184E-05	1.176396E-04	-1.489399E-04
41	H9	-1.997639E-04	-2.093410E-04	-2.637846E-04

```
  42    H10     2.310080E-04   2.058152E-04  -3.046466E-04
  43    H25    -5.723237E-05   6.307406E-05  -1.828532E-05
 -----------   -------------  -------------  -------------
    total       6.569597E-04   8.467135E-04   2.658720E-05

  end of program der1b


  start of program geopt  14

  geometry optimization step 14
  reading input hessian of dimension   129
   in five columns format
  reading input hessian of dimension   129
   in five columns format
  reading input hessian of dimension   129
   in five columns format


  Level shifts adjusted to satisfy step-size constraints
   Step size:    0.0108550
   Cos(theta):   0.7082419

   Final level shift:  -3.0472439E-02

  energy change:          -7.4265E-06 *  (  5.0000E-05 )
  gradient maximum:        1.4699E-03 .  (  4.5000E-04 )
  gradient rms:            2.7737E-04 *  (  3.0000E-04 )
  step size:   0.01085  trust radius:   0.01000
  displacement maximum:    4.5549E-03 .  (  1.8000E-03 )
  displacement rms:        8.5285E-04 *  (  1.2000E-03 )
  predicted energy change:    -1.5366E-05    geom step:   1.0855E-
02    full step:   1.0855E-02
  molecular structure not yet converged...

  center of mass moved by:
     x:     2.3234E-04     y:    -3.9719E-04     z:     1.1836E-04

  new geometry:
                                angstroms
  atom           x                 y                 z
  N1       -0.2476098628      0.4319499494      1.1700896461
  N2       -1.0050850757     -2.2950871682      1.1949182325
  C4       -1.2225555532     -0.0359392069      1.9934979632
  C5       -0.0345337388     -1.9145591457      0.4863206158
  C6       -1.6345629750     -1.3576518558      2.0372932256
  C7       -1.7207638142      0.9827487410      2.7857085019
  C13       0.6372167911     -2.9214974519     -0.4226269266
  H4       -2.9991767135     -2.6746254046      2.9867461727
  C8       -2.7544283972      0.6541992122      3.6802520196
  C11      -0.9547075557      2.1487129569      2.4185234965
  H7       -3.1936858699      1.4000793918      4.3191782963
```

```
C9      -3.1953417522    -0.6499826706     3.7301011813
H8      -3.9846205275    -0.9126483273     4.4122075724
C10     -2.6412076664    -1.6632555907     2.9184523073
C12     -0.0714118196     1.7763193928     1.4539254438
C2       0.9267126827     2.6085697080     0.7475086448
H13      1.7145636018    -2.8715088035    -0.2838423517
C14      0.3089199593    -2.7020981095    -1.8909152593
H15      0.3081077662    -3.9039526830    -0.1083403583
C15     -0.3215924809    -2.3201267580    -4.5836616469
C16      1.3004540244    -2.3992437756    -2.8137797881
C17     -1.0077278059    -2.8118948597    -2.3339058106
C18     -1.3203617236    -2.6227459376    -3.6680394462
C19      0.9888784831    -2.2093360982    -4.1534576988
H16      2.3240667900    -2.3146888619    -2.4895233992
H17     -1.7849137076    -3.0449393883    -1.6268919447
H18     -2.3419771432    -2.7122708466    -3.9947783125
H19      1.7707403444    -1.9764961643    -4.8551652860
H20     -0.5656277175    -2.1736956496    -5.6211053749
C1       0.5760456600    -0.5105838268     0.4433549677
H5       1.5800429573    -0.5626136306     0.8641513090
H6       0.6825437933    -0.2189447287    -0.5941242795
C3       2.7951744578     4.2145617192    -0.5601377830
C26      1.8187572576     3.3915699332     1.4744469707
C27      0.9785171969     2.6447421378    -0.6446715605
C28      1.9082206454     3.4396720163    -1.2930044553
C29      2.7448249516     4.1917280945     0.8244421729
H1       1.7902754511     3.3610162274     2.5486052296
H2       0.2747696491     2.0686681731    -1.2192937084
H3       1.9337718424     3.4605773143    -2.3682340620
H9       3.4301063300     4.7894624992     1.3997519889
H10      3.5171655374     4.8322369654    -1.0645143063
H25     -1.0688078963     3.1463685995     2.7914697600

nuclear repulsion energy.......  1971.109269844 hartrees
 ------------------------------------------------
/  end of geometry optimization iteration  14   /
------------------------------------------------

end of program geopt


start of program onee
smallest eigenvalue of S:    2.820E-04
number of canonical orbitals.....          461
end of program onee


start of program probe
end of program probe


start of program grid
```

```
  number of gridpoints:
     atom         N1     N2     C4     C5     C6     C7    C13
H4
  grid # 1        96    102     87     87     90     90     84
73
  grid # 2       103    112     95     95     99     98     92
118
  grid # 3       214    227    195    187    191    199    163
224
  grid # 4       389    414    322    320    323    347    299
224

  number of gridpoints:
     atom         C8    C11     H7     C9     H8    C10    C12
C2
  grid # 1        89     86     73     87     73     89     86
92
  grid # 2        97     94    118     97    118     97     94
100
  grid # 3       184    185    223    184    222    184    194
195
  grid # 4       329    331    226    331    223    329    317
341

  number of gridpoints:
     atom        H13    C14    H15    C15    C16    C17    C18
C19
  grid # 1        69     91     69     89     88     88     89
89
  grid # 2       110    100    110     97     96     96     97
96
  grid # 3       211    197    214    183    184    183    184
183
  grid # 4       214    340    213    327    327    327    327
327

  number of gridpoints:
     atom        H16    H17    H18    H19    H20     C1     H5
H6
  grid # 1        72     73     73     73     73     82     70
69
  grid # 2       114    115    118    118    118     88    110
104
  grid # 3       214    216    223    222    224    164    217
207
  grid # 4       214    214    224    223    224    293    217
205

  number of gridpoints:
     atom         C3    C26    C27    C28    C29     H1     H2
H3
```

```
  grid # 1       89     88     88     89     89     72     71
73
  grid # 2       97     95     96     97     97    115    114
118
  grid # 3      185    182    184    185    185    217    213
223
  grid # 4      328    328    329    328    328    217    212
224

  number of gridpoints:
     atom        H9    H10    H25  total
  grid # 1       73     73     72   3518
  grid # 2      118    118    115   4494
  grid # 3      223    224    220   8638
  grid # 4      224    224    224  12247

  end of program grid


  start of program rwr
  end of program rwr


  start of program scf

       i  u  d  i  g
       t  p  i  c  r                                    RMS    maximum
       e  d  i  u  i                          energy  density   DIIS
       r  t  s  t  d     total energy         change  change    error

etot   1  N  N  1  U  -990.39080246980                1.4E-05  9.2E-04
etot   2  Y  Y  4  M  -990.39085334491  5.1E-05  6.4E-06  3.6E-04
etot   3  Y  Y  4  M  -990.39085990967  6.6E-06  2.0E-06  9.6E-05
etot   4  Y  N  4  M  -990.39085924579 -6.6E-07  0.0E+00  0.0E+00


 Energy components, in hartrees:
   (A)  Nuclear repulsion............  1971.10926984366
   (E)  Total one-electron terms..... -5260.48968808571
   (I)  Total two-electron terms.....  2298.98955899626
   (L)  Electronic energy............ -2961.50012908945  (E+I)
   (N)  Total energy.................  -990.39085924579  (A+L)

 SCFE: SCF energy: HF    -990.39085924579 hartrees   iterations:
4


 HOMO energy:    -0.26901
 LUMO energy:     0.09668

  Orbital energies:
  -15.60463  -15.56524  -11.29626  -11.28681  -11.27141  -11.25098
```

```
 -11.25089  -11.24604  -11.24583  -11.23989  -11.23981  -11.23902
 -11.23846  -11.23783  -11.23708  -11.23595  -11.23451  -11.23447
 -11.23271  -11.23045  -11.22455  -11.22026  -11.21879  -11.21610
 -11.21302   -1.32494   -1.24990   -1.16327   -1.16057   -1.13849
  -1.07446   -1.06705   -1.03929   -1.02190   -1.01890   -1.01702
  -0.97103   -0.95434   -0.93367   -0.86016   -0.83696   -0.83361
  -0.82905   -0.80707   -0.79550   -0.76999   -0.73262   -0.71741
  -0.70112   -0.69838   -0.66629   -0.65789   -0.64288   -0.63712
  -0.63384   -0.62367   -0.62072   -0.61582   -0.59621   -0.59233
  -0.59050   -0.58743   -0.57834   -0.55809   -0.55227   -0.54820
  -0.54435   -0.51743   -0.50724   -0.50258   -0.50021   -0.49593
  -0.49348   -0.49117   -0.48739   -0.48119   -0.42972   -0.40488
  -0.36749   -0.34336   -0.34123   -0.33760   -0.32929   -0.28245
  -0.26901    0.09668    0.12894    0.13711    0.13740    0.14856
   0.17846    0.20869    0.22827    0.23519    0.24116

 end of program scf


 start of program der1a
 end of program der1a


 start of program rwr
 end of program rwr


 start of program der1b

 forces (hartrees/bohr) : total
```

atom	label	x	y	z
1	N1	3.594168E-04	-3.247900E-04	-4.707368E-04
2	N2	1.456478E-04	1.299541E-04	-1.590589E-04
3	C4	1.259234E-04	-1.305667E-04	-7.187211E-05
4	C5	-3.678037E-05	-4.521296E-05	1.199478E-04
5	C6	5.984738E-05	-1.658855E-04	-5.162803E-05
6	C7	-6.725914E-05	2.139092E-04	6.314595E-05
7	C13	-3.445068E-05	1.086910E-04	1.095968E-04
8	H4	1.233470E-05	-3.123613E-05	3.656539E-05
9	C8	-2.053567E-05	3.892458E-05	6.619510E-05
10	C11	-3.836909E-05	2.069748E-04	1.279422E-04
11	H7	-2.571961E-06	9.001533E-05	2.737180E-05
12	C9	4.406467E-05	-4.942249E-05	-3.711664E-05
13	H8	-1.489958E-05	3.325105E-06	5.832352E-05
14	C10	-2.354330E-05	2.627673E-05	4.444407E-05
15	C12	1.173535E-04	8.037980E-05	-1.135315E-05
16	C2	3.023010E-05	1.143481E-04	1.144053E-04
17	H13	4.746262E-05	8.375568E-05	4.709878E-06
18	C14	-2.570906E-04	-7.682341E-05	2.962546E-05
19	H15	4.472624E-05	-6.375860E-05	-2.023663E-05

```
 20    C15      1.891440E-04   6.801696E-05  -1.397037E-04
 21    C16      8.780006E-05   7.855990E-05  -3.361955E-05
 22    C17      7.344717E-05  -2.688328E-05   3.500123E-04
 23    C18     -2.375303E-04  -1.002662E-04  -2.346247E-05
 24    C19      3.475516E-05   4.269829E-05  -7.942340E-05
 25    H16     -1.546063E-05  -3.301204E-05  -2.106096E-05
 26    H17      6.240769E-05   4.272218E-05   5.193411E-05
 27    H18      2.079249E-04   6.398798E-05   5.979060E-05
 28    H19     -1.512320E-05  -2.486948E-05  -5.670209E-05
 29    H20      3.073742E-05   1.633282E-05  -8.591535E-05
 30    C1      -1.156034E-04   1.492051E-04   1.091582E-04
 31    H5      -1.311443E-04   5.783864E-05   4.118251E-05
 32    H6       4.583649E-05   3.741885E-05   1.991567E-04
 33    C3      -1.431507E-04  -3.099268E-05  -1.181485E-04
 34    C26      4.632667E-05   6.386637E-05  -1.655493E-04
 35    C27      1.736270E-05   4.420336E-05  -2.361738E-04
 36    C28      3.924001E-05   3.200689E-05   1.369748E-04
 37    C29      1.549265E-05   1.514235E-05   4.802569E-05
 38    H1      -3.310335E-05   2.030411E-05   1.784773E-04
 39    H2       9.751055E-05   8.306954E-05   8.534773E-05
 40    H3       2.527376E-05   2.668486E-05  -9.347572E-05
 41    H9      -9.749903E-05  -4.736787E-05  -9.930280E-05
 42    H10     -2.819321E-06   2.249804E-05  -7.816079E-05
 43    H25     -1.728472E-05   5.798267E-05   1.449746E-05
-----------   ------------  ------------  ------------
   total        6.560473E-04   8.680061E-04   2.412989E-05

 end of program der1b


 start of program geopt  15

 geometry optimization step 15
 reading input hessian of dimension   129
  in five columns format
 reading input hessian of dimension   129
  in five columns format


 Level shifts adjusted to satisfy step-size constraints
  Step size:    0.0143612
  Cos(theta):   0.4919231

  Final level shift:  -2.7000810E-02

 energy change:          -1.0263E-05 *  (  5.0000E-05 )
 gradient maximum:        4.0569E-04 *  (  4.5000E-04 )
 gradient rms:            1.0457E-04 *  (  3.0000E-04 )
 step size:   0.01436  trust radius:   0.01414
 displacement maximum:    8.4903E-03 .  (  1.8000E-03 )
 displacement rms:        1.1283E-03 *  (  1.2000E-03 )
```

predicted energy change: -7.4859E-06 geom step: 1.4361E-02 full step: 1.4361E-02
molecular structure not yet converged...

center of mass moved by:
x: 2.6501E-05 y: -2.4923E-04 z: 6.1267E-05

new geometry:

	angstroms		
atom	x	y	z
N1	-0.2463607370	0.4314193906	1.1710338958
N2	-1.0035424221	-2.2955233045	1.1947806590
C4	-1.2216235933	-0.0368540479	1.9940347638
C5	-0.0326064789	-1.9145180546	0.4866450499
C6	-1.6331040260	-1.3588415492	2.0376289344
C7	-1.7214275643	0.9821252034	2.7857401080
C13	0.6387952323	-2.9207111820	-0.4230303496
H4	-2.9983933254	-2.6759482577	2.9865390107
C8	-2.7554322781	0.6531437848	3.6795071530
C11	-0.9556687926	2.1484771315	2.4194464699
H7	-3.1957362911	1.3993275357	4.3177668929
C9	-3.1954005477	-0.6509580953	3.7294178623
H8	-3.9851392350	-0.9139827639	4.4112945459
C10	-2.6402354285	-1.6642884757	2.9184542048
C12	-0.0716231059	1.7762215315	1.4549835704
C2	0.9257296648	2.6089482628	0.7484190091
H13	1.7160363204	-2.8699892414	-0.2843745120
C14	0.3092857777	-2.7015111760	-1.8910420198
H15	0.3107659365	-3.9038972276	-0.1098048464
C15	-0.3210182211	-2.3198001555	-4.5845563047
C16	1.3026267989	-2.4078132613	-2.8158087619
C17	-1.0093417649	-2.8017601438	-2.3324540938
C18	-1.3215507168	-2.6128445964	-3.6669555125
C19	0.9912426335	-2.2182211833	-4.1556558124
H16	2.3277109198	-2.3309831513	-2.4925549566
H17	-1.7872243266	-3.0262248434	-1.6238607485
H18	-2.3426567575	-2.6932803270	-3.9926346211
H19	1.7741739528	-1.9932912445	-4.8591528825
H20	-0.5645529054	-2.1734405864	-5.6223463359
C1	0.5782555737	-0.5107568800	0.4460053859
H5	1.5811573473	-0.5631828239	0.8704649311
H6	0.6880840404	-0.2177615274	-0.5909622482
C3	2.7922215595	4.2150771119	-0.5617697159
C26	1.8178585798	3.3922403513	1.4746958238
C27	0.9762379671	2.6449102789	-0.6443115909
C28	1.9050145102	3.4399273512	-1.2938231691
C29	2.7430458157	4.1923393067	0.8232302794
H1	1.7893807507	3.3619023049	2.5491513072
H2	0.2718921784	2.0685313456	-1.2175644237
H3	1.9300026211	3.4609305987	-2.3692287604
H9	3.4287854490	4.7902046058	1.3973483110
H10	3.5140165769	4.8329081285	-1.0672737690

```
  H25      -1.0711083010      3.1462171349      2.7921150838

  nuclear repulsion energy.......  1971.034696625 hartrees
  ------------------------------------------------
 /  end of geometry optimization iteration  15   /
 ------------------------------------------------

  end of program geopt


  start of program onee
  smallest eigenvalue of S:    2.821E-04
  number of canonical orbitals.....          461
  end of program onee


  start of program probe
  end of program probe


  start of program grid

  number of gridpoints:
     atom        N1     N2      C4      C5      C6      C7    C13
H4
  grid # 1       96    102      87      87      90      89     84
73
  grid # 2      103    112      95      95      99      98     92
118
  grid # 3      214    227     195     187     191     199    163
224
  grid # 4      388    414     321     320     322     348    299
224

  number of gridpoints:
     atom        C8    C11      H7      C9      H8     C10    C12
C2
  grid # 1       89     86      73      87      73      89     86
92
  grid # 2       97     94     118      97     118      97     94
100
  grid # 3      184    185     223     184     222     184    194
195
  grid # 4      328    331     226     331     223     329    317
343

  number of gridpoints:
     atom       H13    C14     H15     C15     C16     C17    C18
C19
  grid # 1       69     91      69      89      88      88     89
89
```

```
  grid # 2      109    100    110     97     96     96     97
96
  grid # 3      210    196    214    183    184    183    184
183
  grid # 4      214    340    213    327    327    327    327
327

  number of gridpoints:
     atom        H16    H17    H18    H19    H20     C1     H5
H6
  grid # 1       72     73     73     73     73     82     70
69
  grid # 2      114    115    118    118    118     88    109
104
  grid # 3      214    216    222    222    224    164    217
207
  grid # 4      214    214    224    223    224    293    219
205

  number of gridpoints:
     atom         C3    C26    C27    C28    C29     H1     H2
H3
  grid # 1       89     88     88     89     89     72     71
73
  grid # 2       97     95     96     97     97    115    114
118
  grid # 3      185    182    183    185    185    217    213
223
  grid # 4      328    327    329    327    328    217    212
224

  number of gridpoints:
     atom         H9    H10    H25  total
  grid # 1       73     73     72   3517
  grid # 2      118    118    115   4492
  grid # 3      222    224    220   8633
  grid # 4      224    224    224  12246

  end of program grid


  start of program rwr
  end of program rwr


  start of program scf

       i  u  d  i  g
       t  p  i  c  r                                  RMS    maximum
       e  d  i  u  i                        energy  density   DIIS
       r  t  s  t  d     total energy       change  change    error
```

```
etot   1  N  N  1  U  -990.39072743546            2.7E-05  1.6E-03
etot   2  Y  Y  4  M  -990.39084046479  1.1E-04  1.0E-05  6.3E-04
etot   3  Y  Y  4  M  -990.39085660547  1.6E-05  2.9E-06  1.5E-04
etot   4  Y  N  4  M  -990.39085842189  1.8E-06  0.0E+00  0.0E+00


 Energy components, in hartrees:
   (A)  Nuclear repulsion............  1971.03469662474
   (E)  Total one-electron terms..... -5260.33898399291
   (I)  Total two-electron terms.....  2298.91342894628
   (L)  Electronic energy............ -2961.42555504663  (E+I)
   (N)  Total energy.................  -990.39085842189  (A+L)

 SCFE: SCF energy: HF    -990.39085842189 hartrees   iterations:
4


 HOMO energy:    -0.26904
 LUMO energy:     0.09663

  Orbital energies:
  -15.60476  -15.56511  -11.29628  -11.28688  -11.27173  -11.25112
  -11.25088  -11.24616  -11.24601  -11.23993  -11.23979  -11.23905
  -11.23848  -11.23784  -11.23711  -11.23607  -11.23466  -11.23444
  -11.23292  -11.23053  -11.22458  -11.22023  -11.21899  -11.21606
  -11.21326   -1.32501   -1.24984   -1.16321   -1.16033   -1.13852
   -1.07453   -1.06701   -1.03926   -1.02190   -1.01887   -1.01693
   -0.97093   -0.95430   -0.93367   -0.86015   -0.83703   -0.83361
   -0.82898   -0.80705   -0.79545   -0.77000   -0.73261   -0.71741
   -0.70108   -0.69829   -0.66635   -0.65790   -0.64282   -0.63719
   -0.63386   -0.62365   -0.62070   -0.61581   -0.59629   -0.59238
   -0.59044   -0.58747   -0.57825   -0.55807   -0.55220   -0.54819
   -0.54450   -0.51739   -0.50722   -0.50256   -0.50019   -0.49584
   -0.49341   -0.49117   -0.48733   -0.48121   -0.42975   -0.40483
   -0.36754   -0.34337   -0.34122   -0.33756   -0.32914   -0.28243
   -0.26904    0.09663    0.12889    0.13707    0.13733    0.14845
    0.17836    0.20861    0.22838    0.23520    0.24116

  end of program scf


  start of program der1a
  end of program der1a


  start of program rwr
  end of program rwr


  start of program der1b

  forces (hartrees/bohr) : total
```

atom	label	x	y	z
1	N1	-2.481303E-04	2.161363E-04	3.038174E-05
2	N2	2.836382E-04	-2.499438E-05	-2.728610E-04
3	C4	-5.332666E-05	1.576006E-04	1.308833E-04
4	C5	-3.595149E-04	-1.387013E-04	2.963041E-04
5	C6	2.019594E-05	1.908855E-04	2.396059E-05
6	C7	2.466402E-04	-1.423657E-04	-1.552636E-04
7	C13	-1.276988E-04	5.612039E-06	6.448695E-05
8	H4	8.439654E-05	1.557089E-04	3.881350E-05
9	C8	1.165608E-05	5.058858E-04	1.509718E-04
10	C11	1.471085E-04	-4.822498E-05	-3.124871E-04
11	H7	5.901722E-05	-5.856555E-05	-6.083047E-05
12	C9	-2.081620E-04	-4.628287E-04	1.114154E-04
13	H8	1.113241E-04	3.132659E-05	-7.909427E-05
14	C10	-1.274255E-04	-2.859290E-04	1.870702E-05
15	C12	-5.235787E-06	8.359514E-05	2.010785E-04
16	C2	2.011226E-05	1.154169E-04	-3.679946E-04
17	H13	1.231949E-04	6.717329E-05	-9.936772E-05
18	C14	-2.239323E-04	1.957682E-04	-5.369846E-04
19	H15	3.610993E-06	-2.367943E-05	1.405502E-04
20	C15	4.528455E-04	8.163430E-06	3.393146E-04
21	C16	-5.301138E-05	-4.748777E-05	2.288187E-04
22	C17	9.188483E-04	1.208919E-04	1.826117E-04
23	C18	1.202191E-03	1.978553E-04	5.774334E-05
24	C19	-4.165740E-04	-3.910772E-05	-2.787547E-04
25	H16	-3.998587E-04	2.166071E-05	-1.106724E-04
26	H17	-1.196398E-04	-1.085782E-04	1.297513E-04
27	H18	-9.010150E-04	-1.633065E-04	-2.194062E-04
28	H19	-1.346353E-04	2.261628E-05	1.123854E-04
29	H20	4.154382E-06	-5.819682E-06	1.013341E-04
30	C1	5.219134E-04	1.699161E-04	-4.100770E-06
31	H5	-3.070221E-04	-1.084423E-05	-1.892583E-04
32	H6	6.403176E-05	-1.027693E-04	3.230711E-04
33	C3	1.449365E-04	1.660298E-04	1.228439E-04
34	C26	-3.787139E-07	5.421162E-05	-1.973404E-04
35	C27	1.942710E-05	4.496688E-05	2.562209E-04
36	C28	-4.419795E-05	-3.799500E-05	5.523642E-05
37	C29	-1.078932E-06	-5.617927E-06	-2.284443E-04
38	H1	8.125850E-05	7.200215E-05	-6.136742E-05
39	H2	4.595660E-05	3.363527E-05	-2.715897E-05
40	H3	-3.156831E-05	-9.218442E-06	3.647842E-06
41	H9	3.691536E-05	8.898917E-05	2.667129E-05
42	H10	-2.160082E-04	-1.433661E-04	1.416099E-04
43	H25	3.817919E-05	-4.036847E-05	-5.326570E-05
total		6.631381E-04	8.262798E-04	3.416091E-05

end of program der1b

```
start of program geopt  16

geometry optimization step 16
reading input hessian of dimension   129
 in five columns format
reading input hessian of dimension   129
 in five columns format
** restarting optimization from step   15 **


Level shifts adjusted to satisfy step-size constraints
 Step size:    0.0104342
 Cos(theta):   0.4965050

 Final level shift:  -2.6262214E-02

energy change:           8.2390E-07 #  (  5.0000E-05 )
gradient maximum:        4.0569E-04 *  (  4.5000E-04 )
gradient rms:            1.0457E-04 *  (  3.0000E-04 )
step size:   0.01043  trust radius:   0.01000
displacement maximum:    6.0223E-03 .  (  1.8000E-03 )
displacement rms:        8.1979E-04 *  (  1.2000E-03 )
predicted energy change:    -4.8790E-06    geom step:   1.0434E-
02    full step:   1.0434E-02

********************************************************
**            Geometry optimization complete          **
********************************************************

center of mass moved by:
   x:     0.0000E+00     y:    -6.8695E-16     z:     3.8858E-16

final geometry:
                              angstroms
atom            x                  y                  z
N1       -0.2476098628      0.4319499494      1.1700896461
N2       -1.0050850757     -2.2950871682      1.1949182325
C4       -1.2225555532     -0.0359392069      1.9934979632
C5       -0.0345337388     -1.9145591457      0.4863206158
C6       -1.6345629750     -1.3576518558      2.0372932256
C7       -1.7207638142      0.9827487410      2.7857085019
C13       0.6372167911     -2.9214974519     -0.4226269266
H4       -2.9991767135     -2.6746254046      2.9867461727
C8       -2.7544283972      0.6541992122      3.6802520196
C11      -0.9547075557      2.1487129569      2.4185234965
H7       -3.1936858699      1.4000793918      4.3191782963
C9       -3.1953417522     -0.6499826706      3.7301011813
H8       -3.9846205275     -0.9126483273      4.4122075724
C10      -2.6412076664     -1.6632555907      2.9184523073
C12      -0.0714118196      1.7763193928      1.4539254438
C2        0.9267126827      2.6085697080      0.7475086448
H13       1.7145636018     -2.8715088035     -0.2838423517
```

```
 C14       0.3089199593     -2.7020981095     -1.8909152593
 H15       0.3081077662     -3.9039526830     -0.1083403583
 C15      -0.3215924809     -2.3201267580     -4.5836616469
 C16       1.3004540244     -2.3992437756     -2.8137797881
 C17      -1.0077278059     -2.8118948597     -2.3339058106
 C18      -1.3203617236     -2.6227459376     -3.6680394462
 C19       0.9888784831     -2.2093360982     -4.1534576988
 H16       2.3240667900     -2.3146888619     -2.4895233992
 H17      -1.7849137076     -3.0449393883     -1.6268919447
 H18      -2.3419771432     -2.7122708466     -3.9947783125
 H19       1.7707403444     -1.9764961643     -4.8551652860
 H20      -0.5656277175     -2.1736956496     -5.6211053749
 C1        0.5760456600     -0.5105838268      0.4433549677
 H5        1.5800429573     -0.5626136306      0.8641513090
 H6        0.6825437933     -0.2189447287     -0.5941242795
 C3        2.7951744578      4.2145617192     -0.5601377830
 C26       1.8187572576      3.3915699332      1.4744469707
 C27       0.9785171969      2.6447421378     -0.6446715605
 C28       1.9082206454      3.4396720163     -1.2930044553
 C29       2.7448249516      4.1917280945      0.8244421729
 H1        1.7902754511      3.3610162274      2.5486052296
 H2        0.2747696491      2.0686681731     -1.2192937084
 H3        1.9337718424      3.4605773143     -2.3682340620
 H9        3.4301063300      4.7894624992      1.3997519889
 H10       3.5171655374      4.8322369654     -1.0645143063
 H25      -1.0688078963      3.1463685995      2.7914697600

 nuclear repulsion energy.......  1971.109269844 hartrees
 -------------------------------------------------
/  end of geometry optimization iteration  16   /
-------------------------------------------------

 end of program geopt


 start of program post
 Writing a SPARTAN archive file
 end of program post

Total cpu seconds     user:    1761.812   user+sys:    1761.812
```

2-benzyl-8-chloro-5-phenyl-3*H*-pyrrolo[1,2,3-*de*]quinoxaline 2a

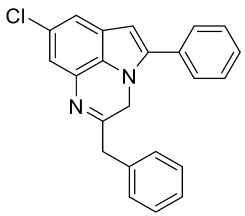

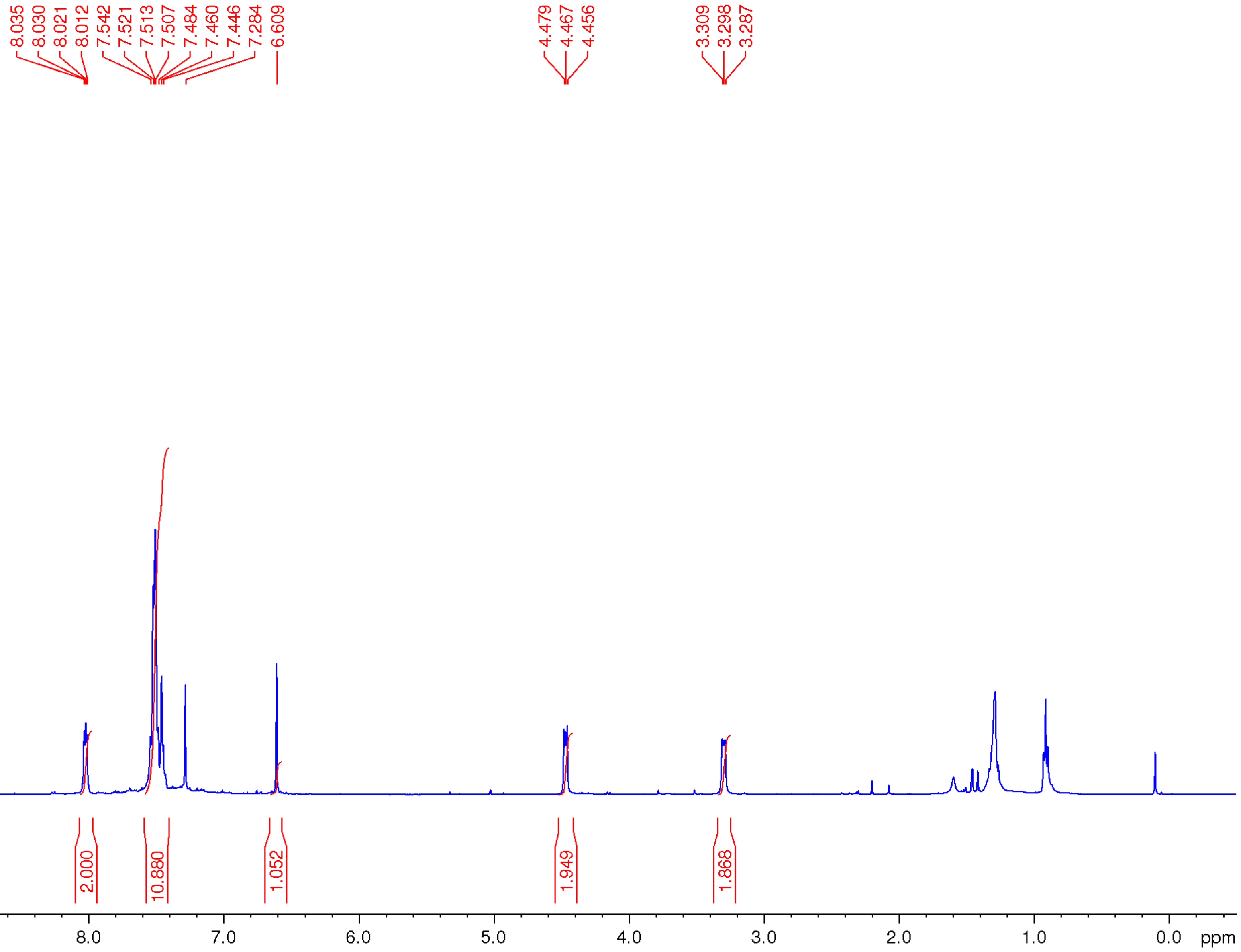

2-benzyl-8-chloro-5-phenyl-3*H*-pyrrolo[1,2,3-*de*]quinoxaline 2a

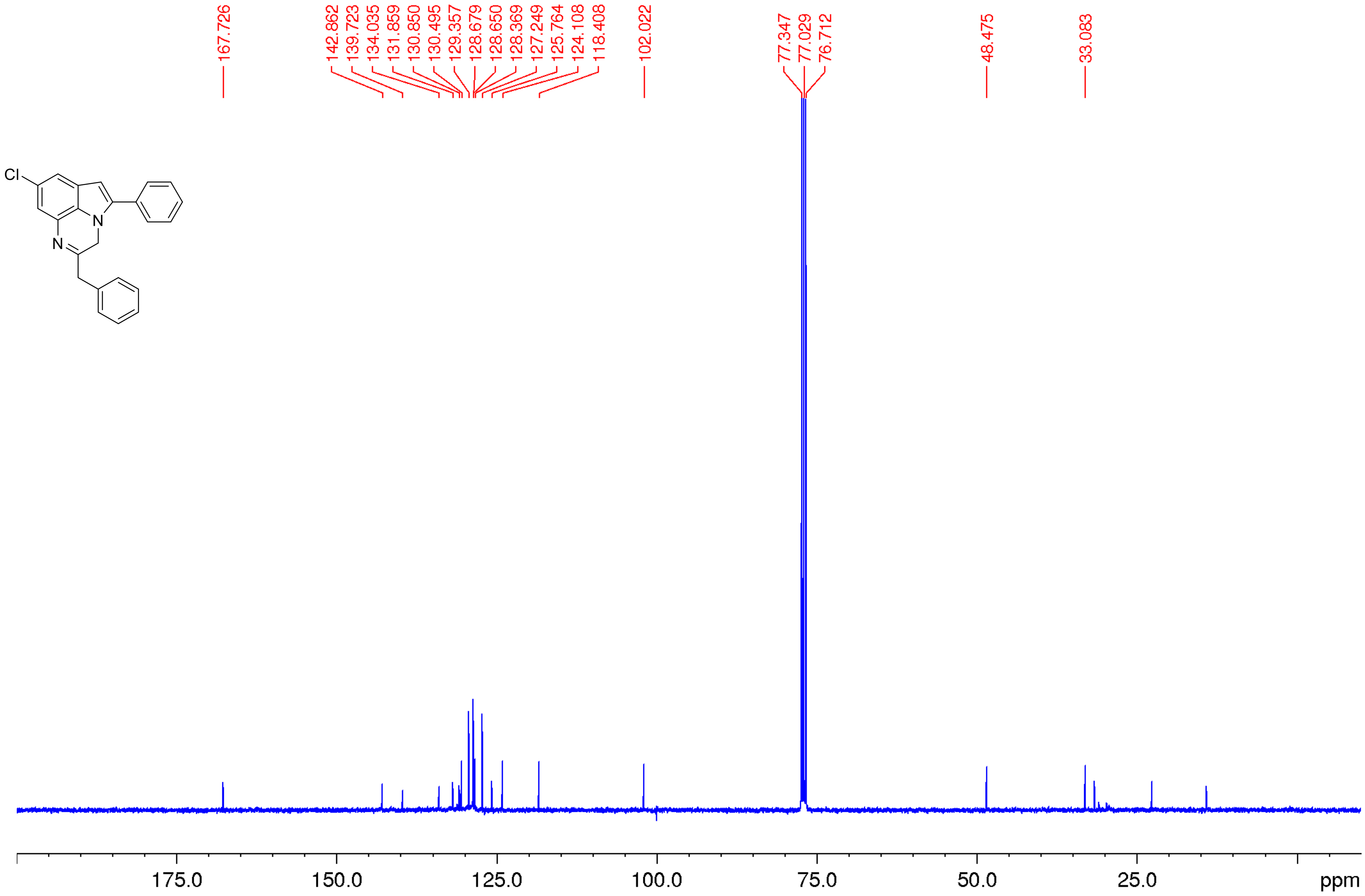

2-benzyl-8-chloro-5-phenyl-3*H*-pyrrolo[1,2,3-*de*]quinoxaline 2a

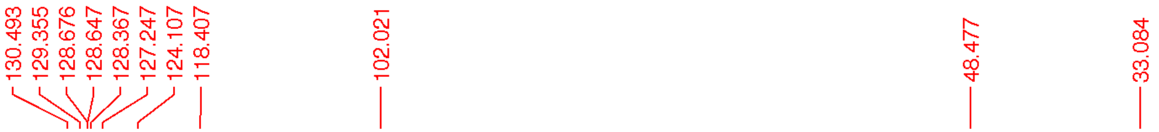

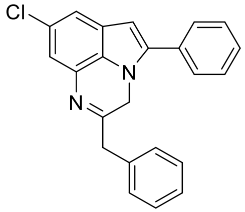

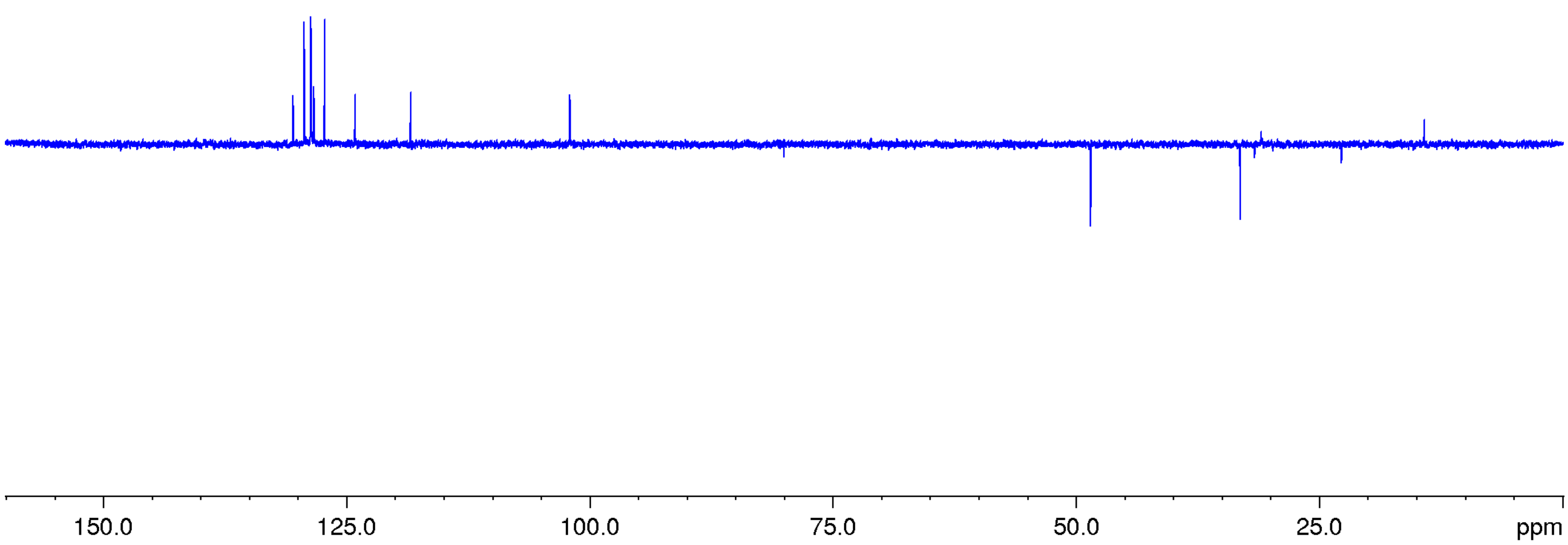

8-chloro-2-(4-chlorobenzyl)-5-phenyl-3*H*-pyrrolo[1,2,3-*de*]quinoxaline 2b

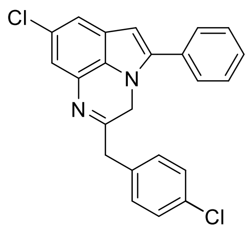

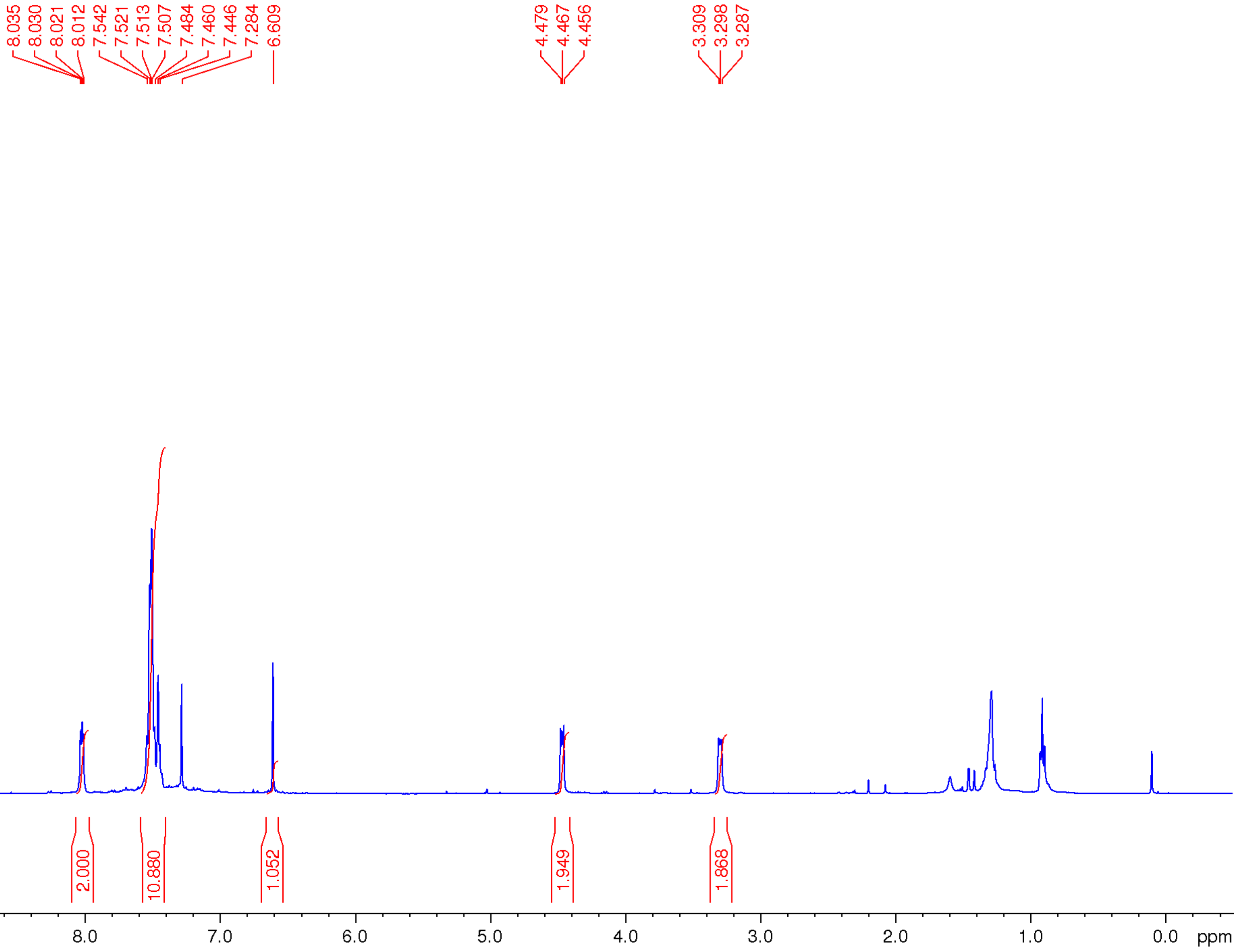

8-chloro-2-(4-chlorobenzyl)-5-phenyl-3*H*-pyrrolo[1,2,3-*de*]quinoxaline 2b

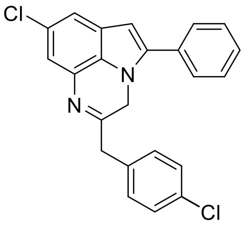

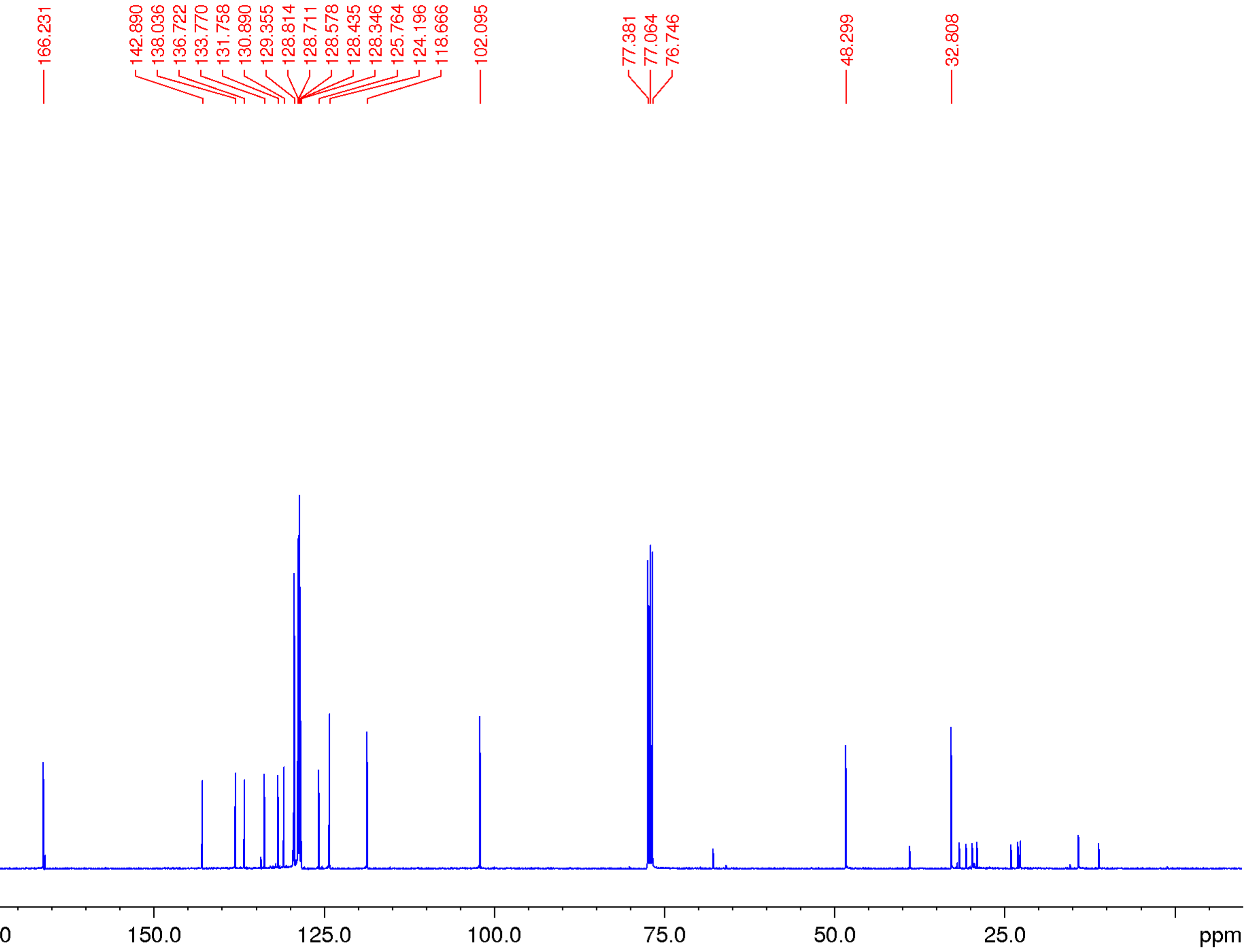

8-chloro-2-(4-chlorobenzyl)-5-phenyl-3*H*-pyrrolo[1,2,3-*de*]quinoxaline 2b

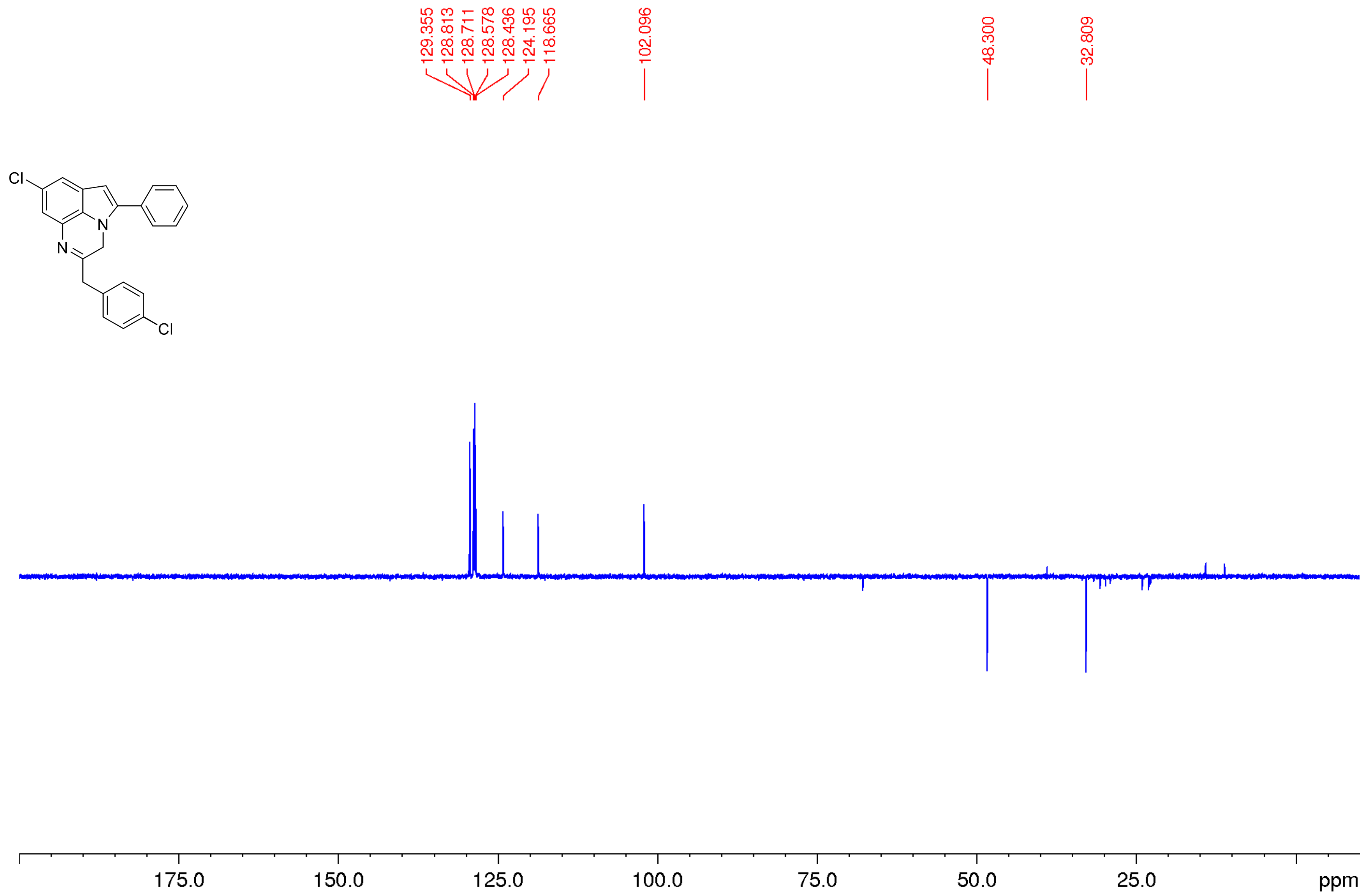

8-chloro-2-(4-methoxybenzyl)-5-phenyl-3*H*-pyrrolo[1,2,3-*de*]quinoxaline 2c

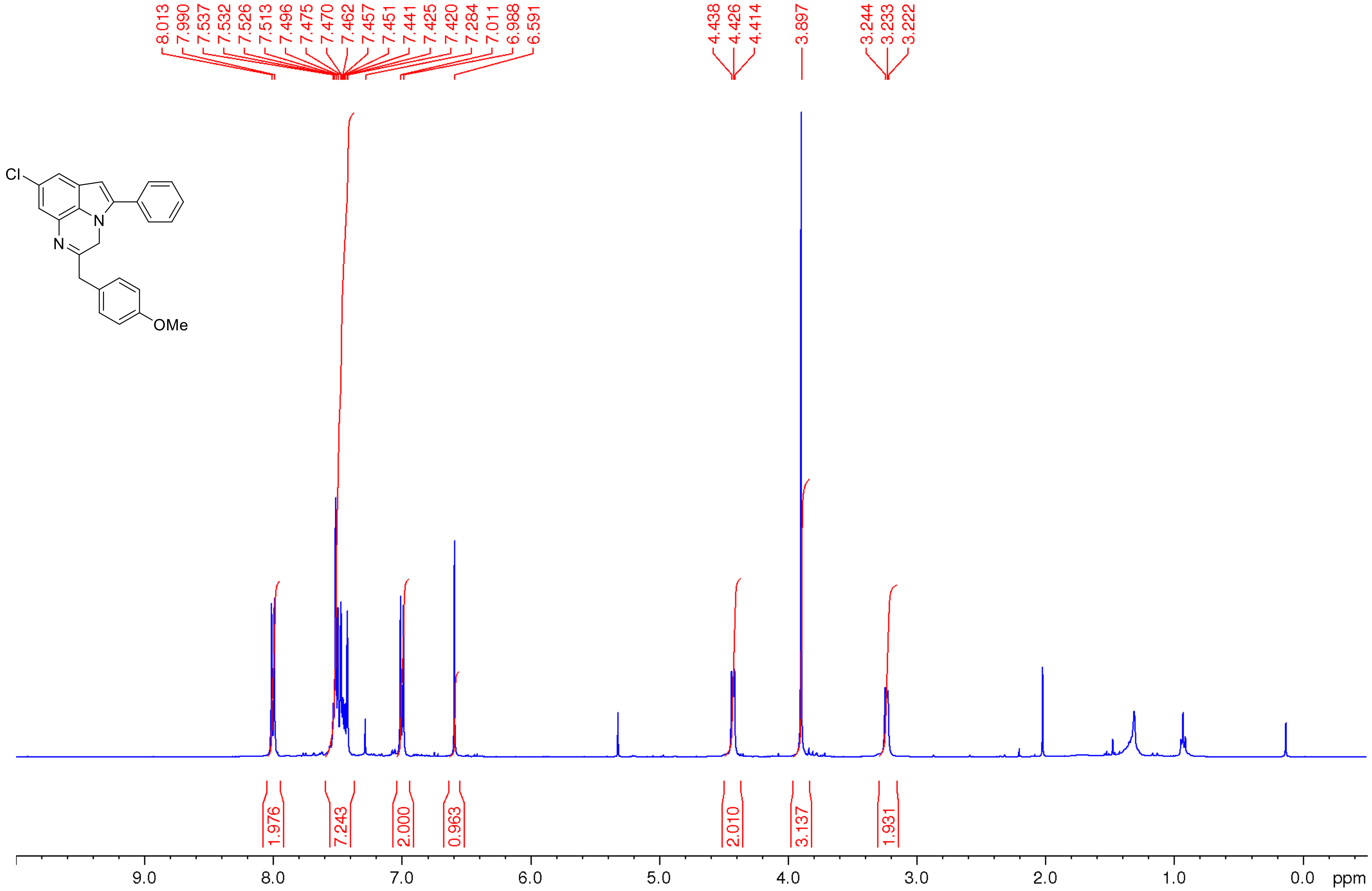

8-chloro-2-(4-methoxybenzyl)-5-phenyl-3*H*-pyrrolo[1,2,3-*de*]quinoxaline 2c

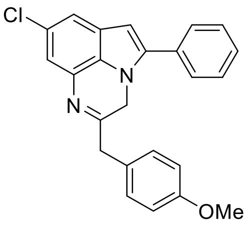

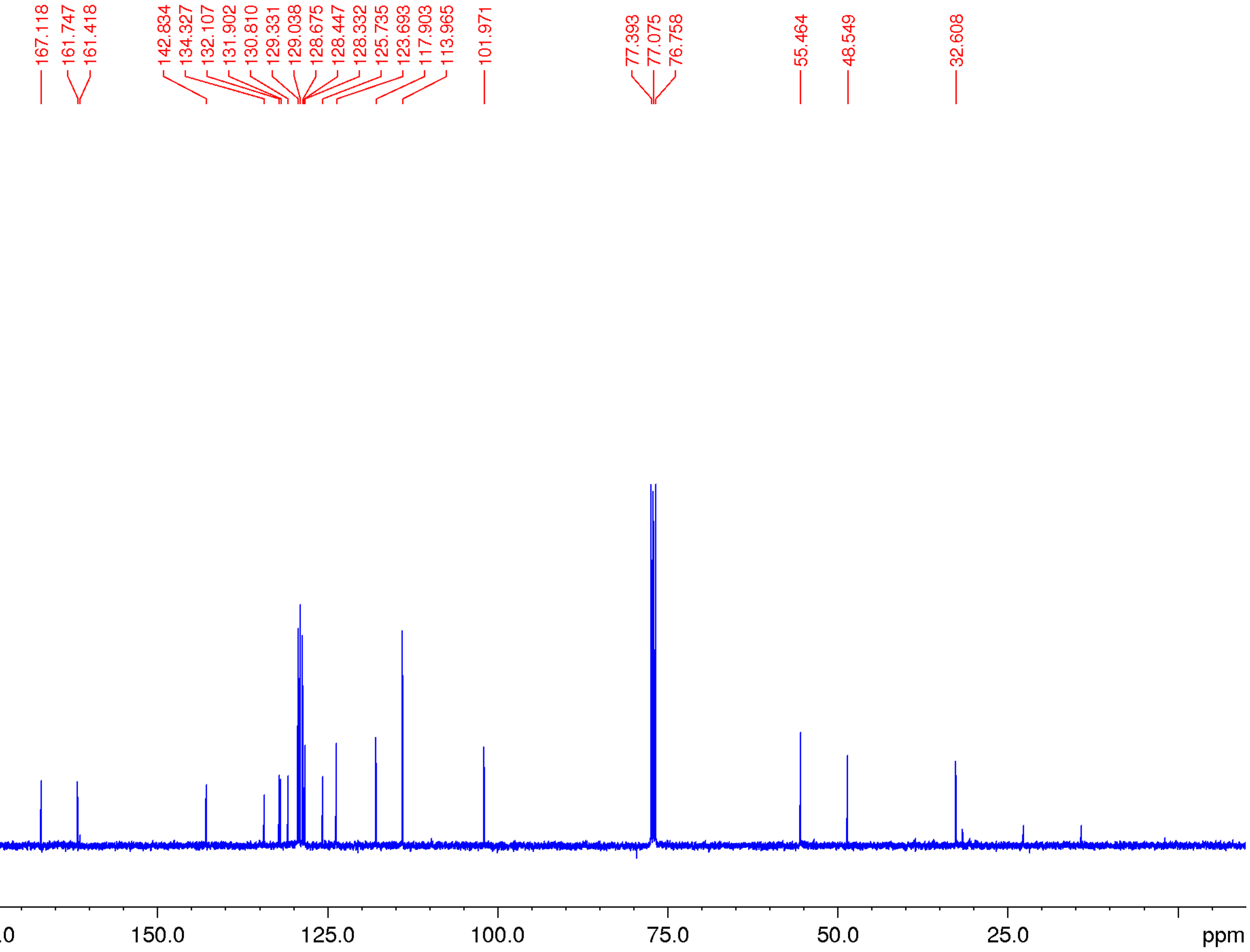

8-chloro-2-(4-methoxybenzyl)-5-phenyl-3*H*-pyrrolo[1,2,3-*de*]quinoxaline 2c

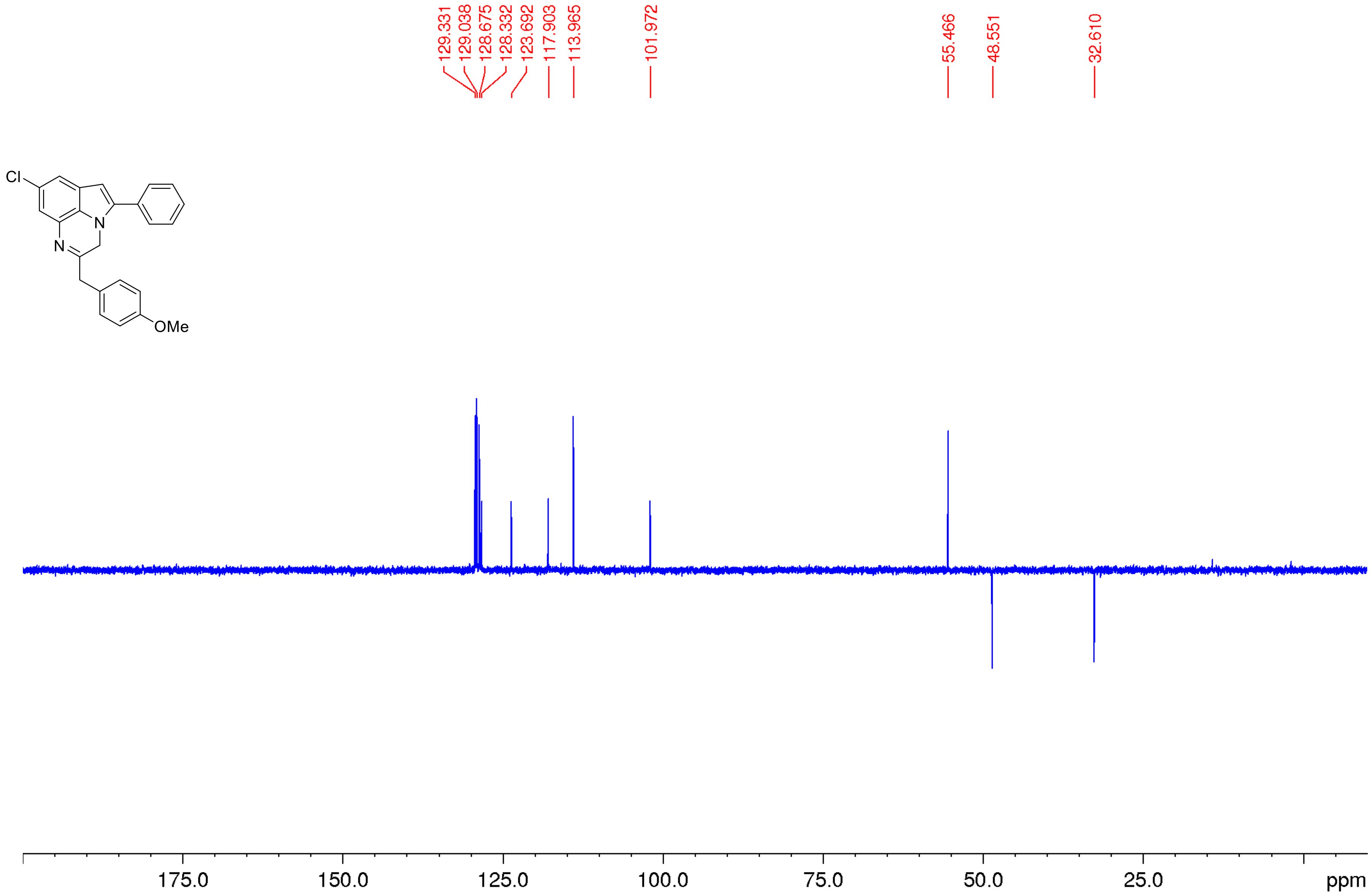

1-(4-((8-chloro-5-phenyl-3*H*-pyrrolo[1,2,3-*de*]quinoxalin-2-yl)methyl)phenyl)ethan-1-one 2d

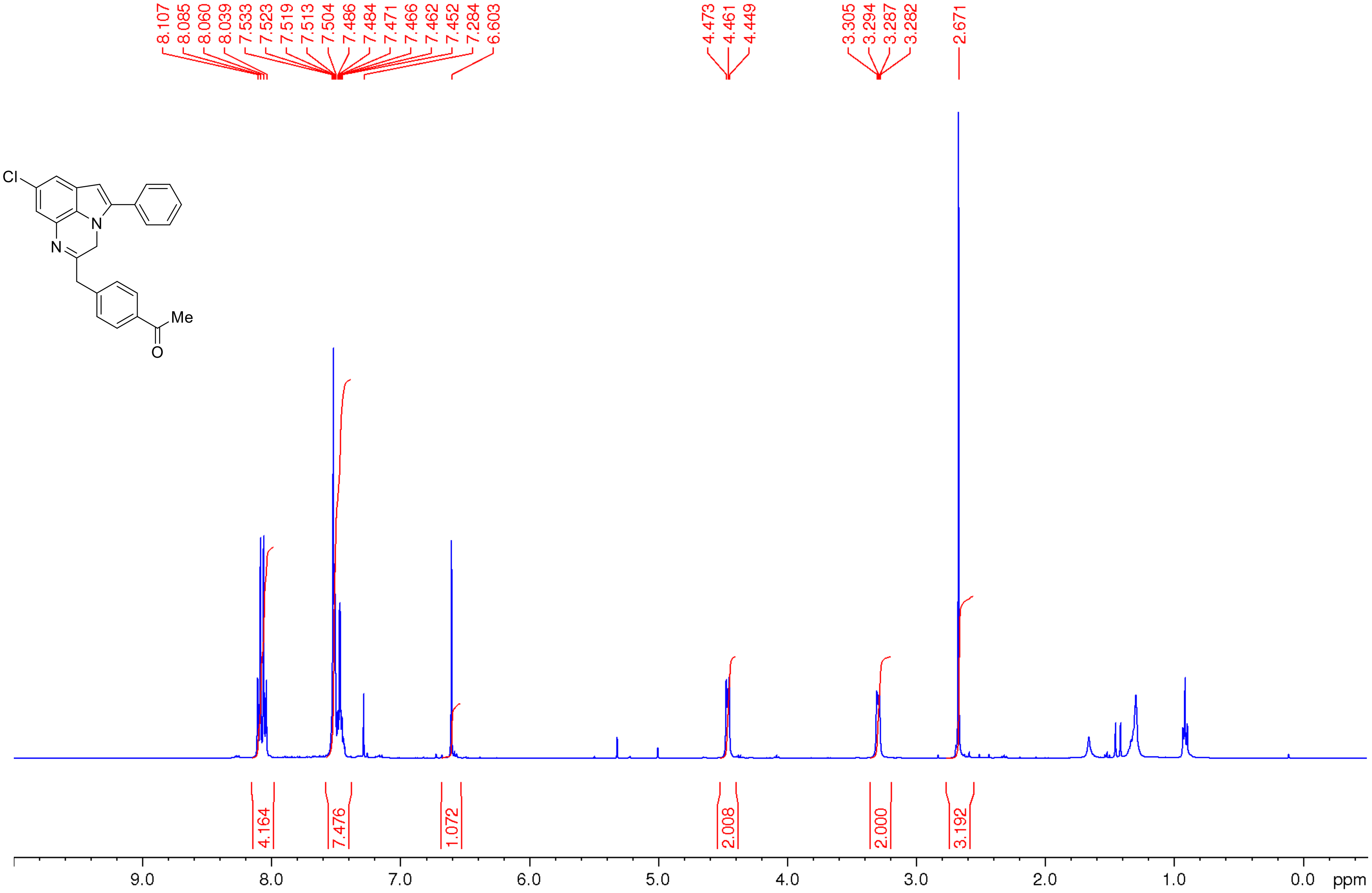

1-(4-((8-chloro-5-phenyl-3*H*-pyrrolo[1,2,3-*de*]quinoxalin-2-yl)methyl)phenyl)ethan-1-one 2d

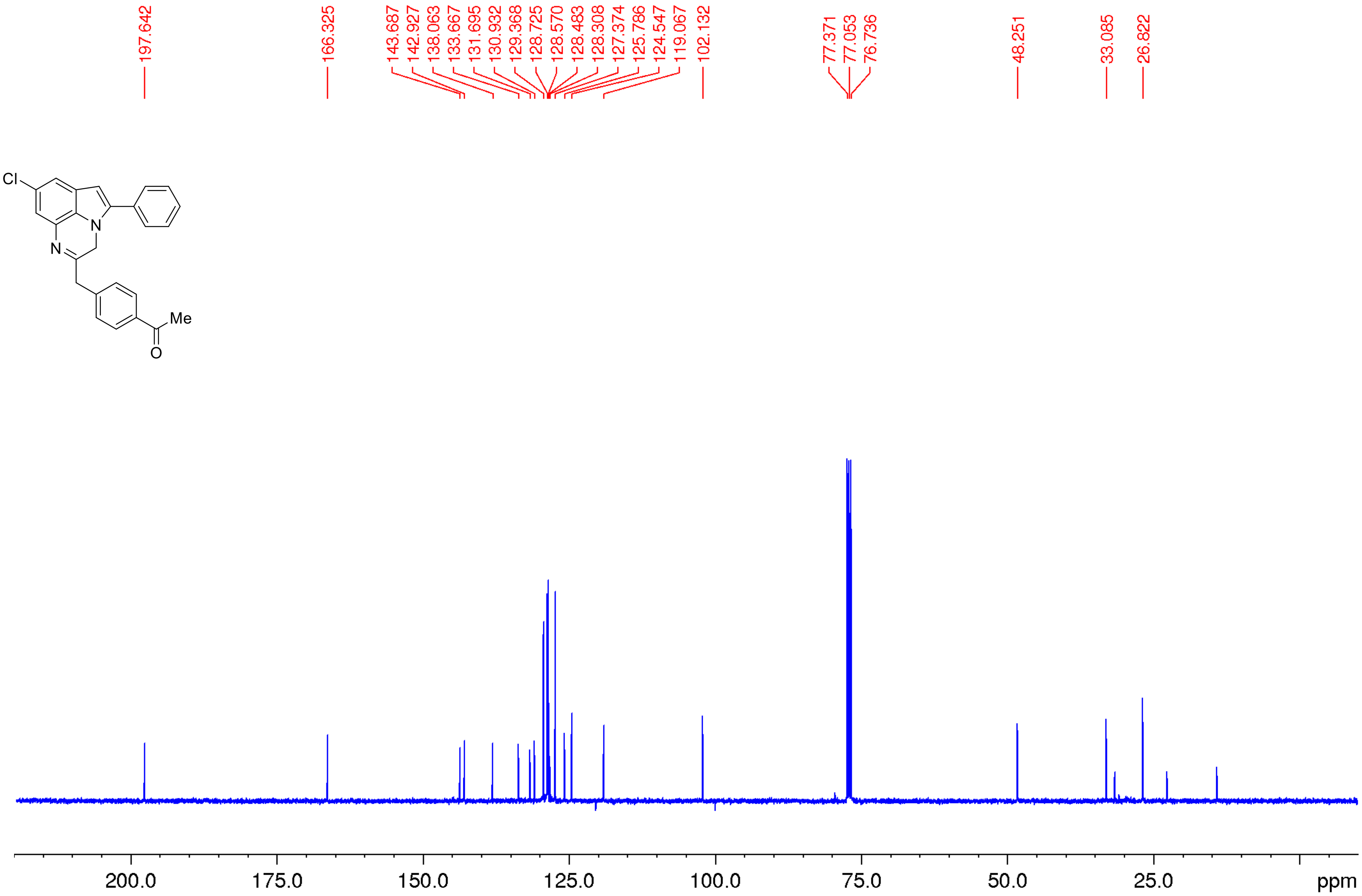

1-(4-((8-chloro-5-phenyl-3*H*-pyrrolo[1,2,3-*de*]quinoxalin-2-yl)methyl)phenyl)ethan-1-one 2d

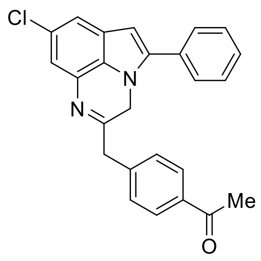

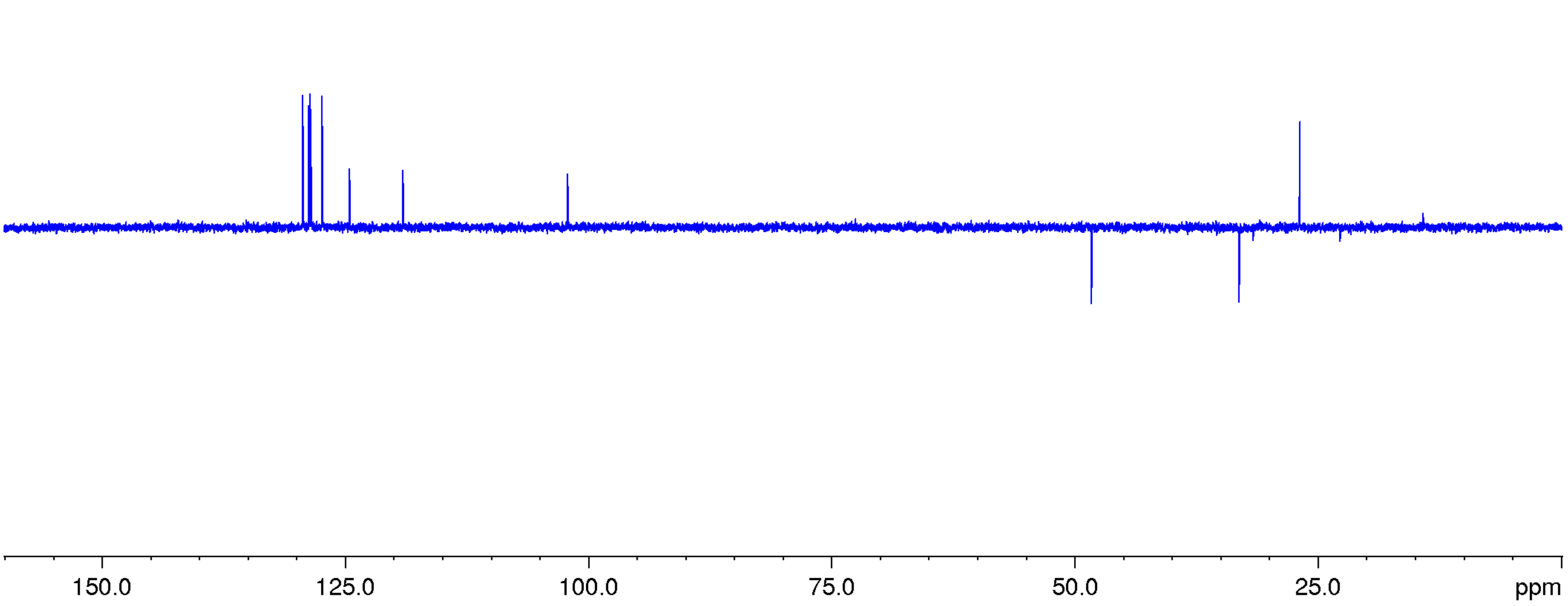

8-chloro-5-(4-methoxyphenyl)-2-(3-(trifluoromethyl)benzyl)-3*H*-pyrrolo[1,2,3-*de*]quinoxaline 2e

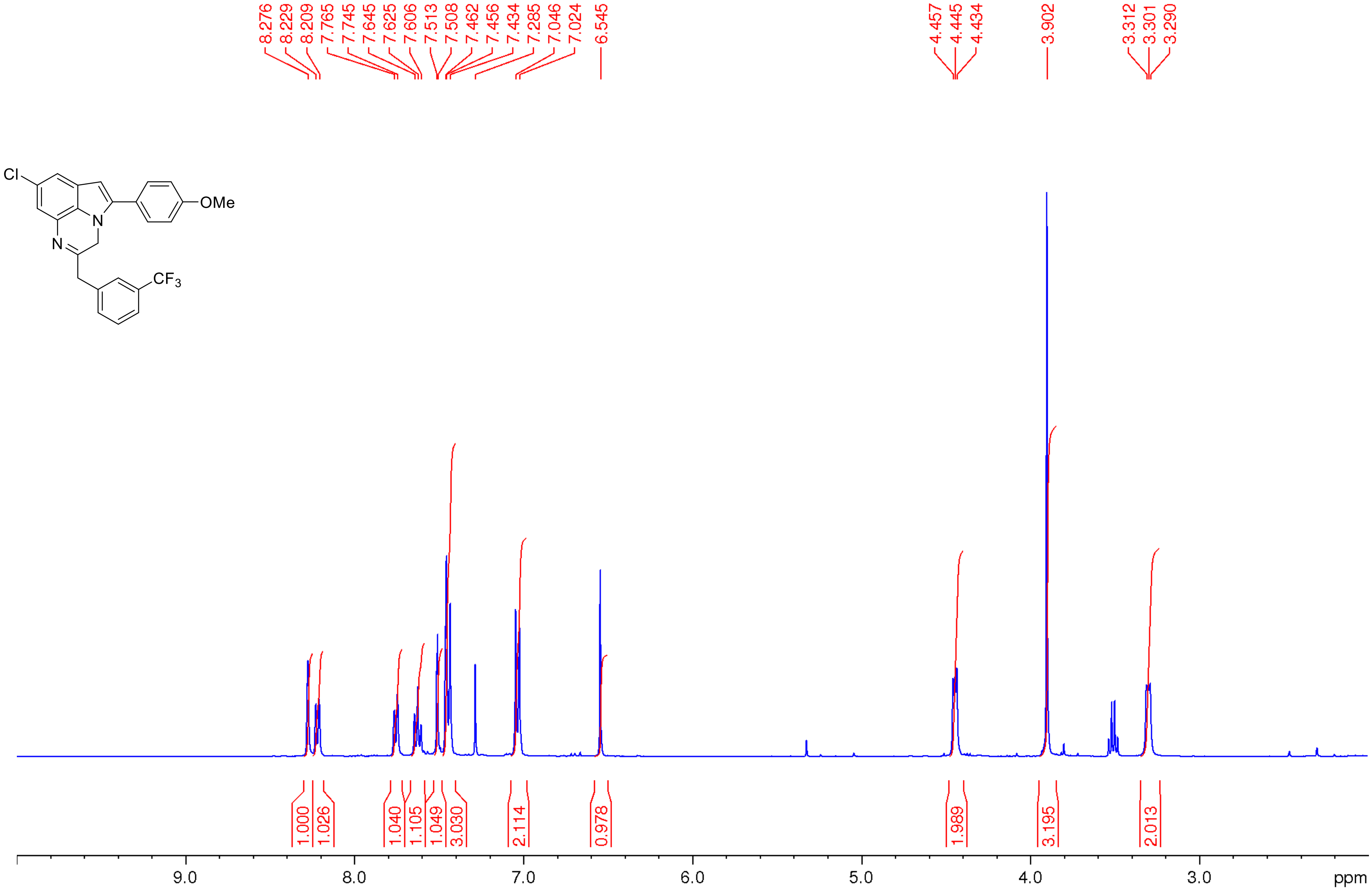

8-chloro-5-(4-methoxyphenyl)-2-(3-(trifluoromethyl)benzyl)-3*H*-pyrrolo[1,2,3-*de*]quinoxaline 2e

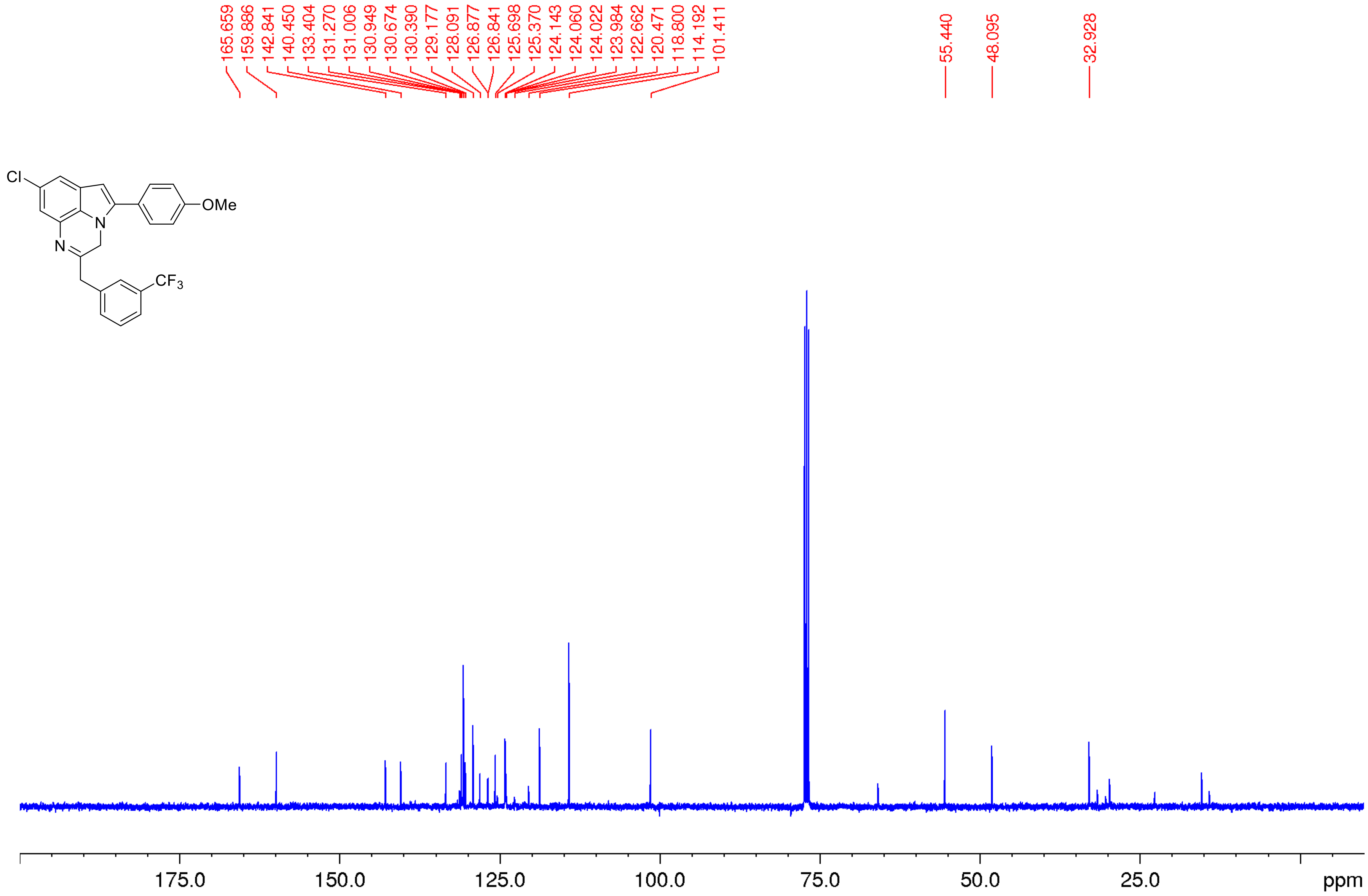

8-chloro-5-(4-methoxyphenyl)-2-(3-(trifluoromethyl)benzyl)-3*H*-pyrrolo[1,2,3-*de*]quinoxaline 2e

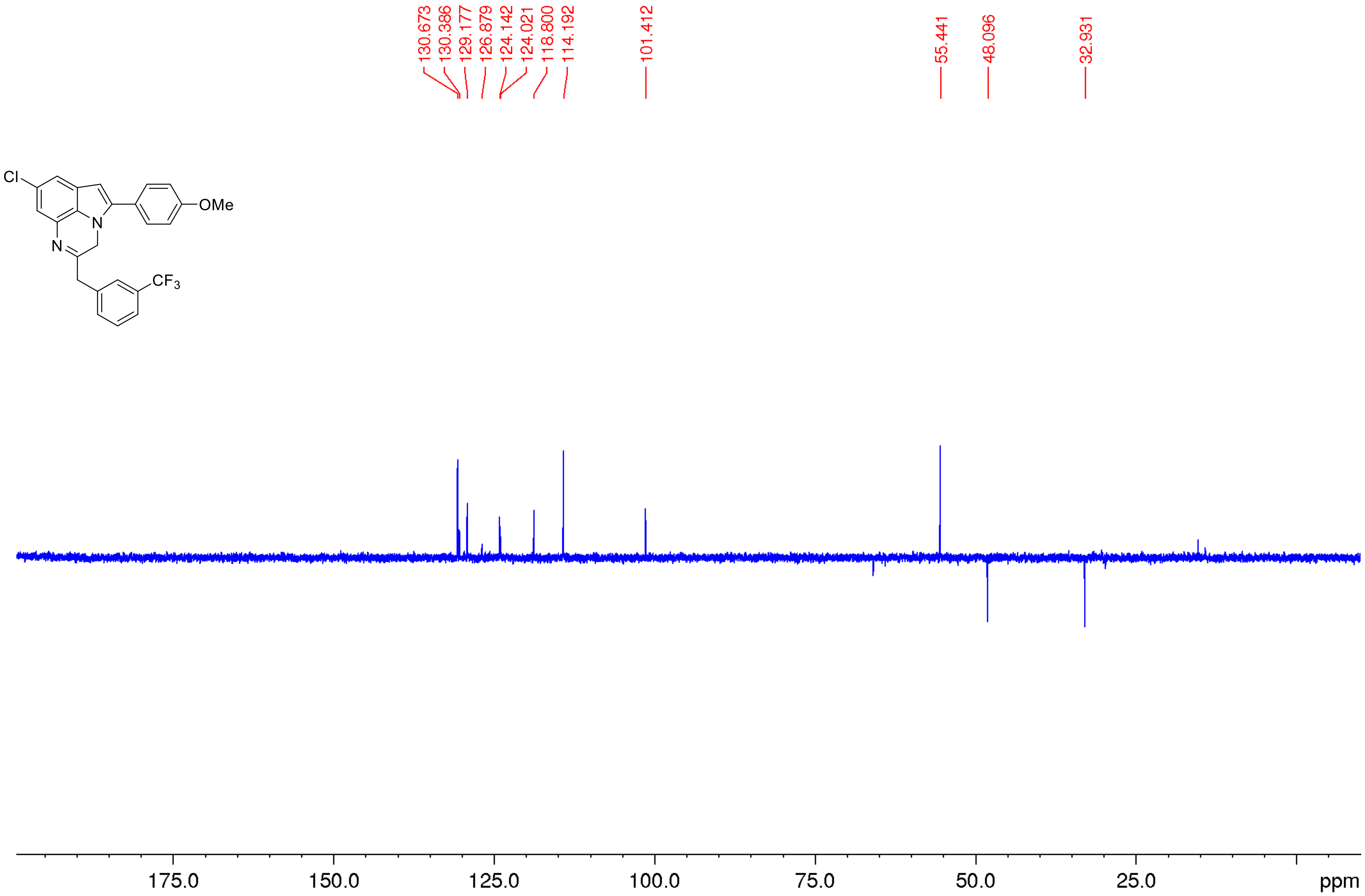

8-chloro-5-(4-methoxyphenyl)-2-(3-(trifluoromethyl)benzyl)-3*H*-pyrrolo[1,2,3-*de*]quinoxaline 2e

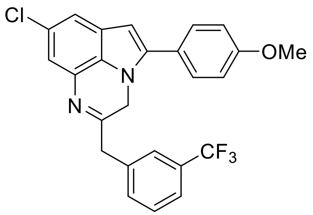

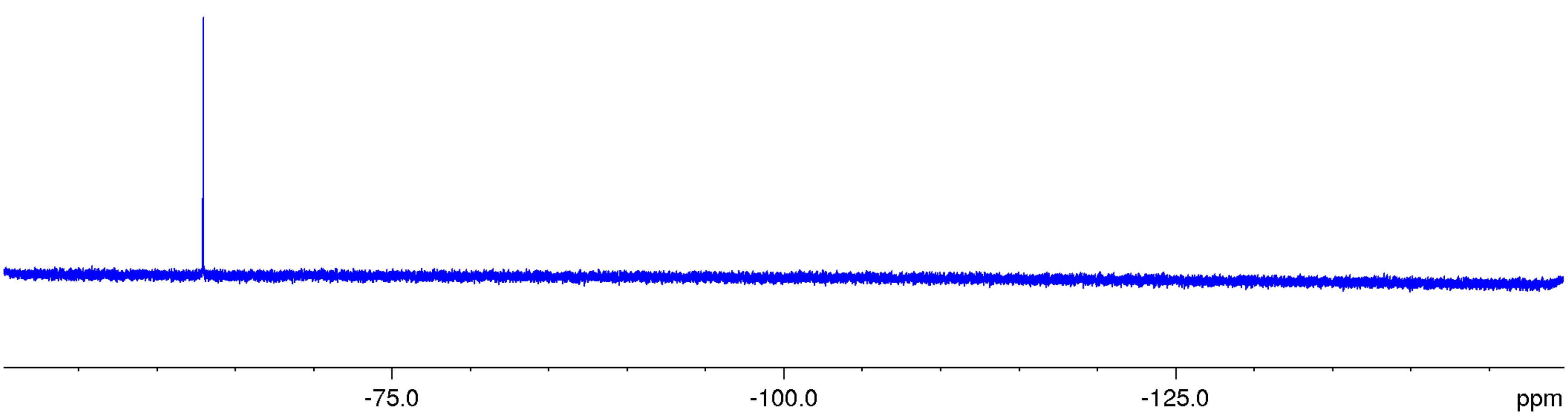

8-chloro-2-(4-methoxybenzyl)-5-(4-methoxyphenyl)-3*H*-pyrrolo[1,2,3-*de*]quinoxaline 2f

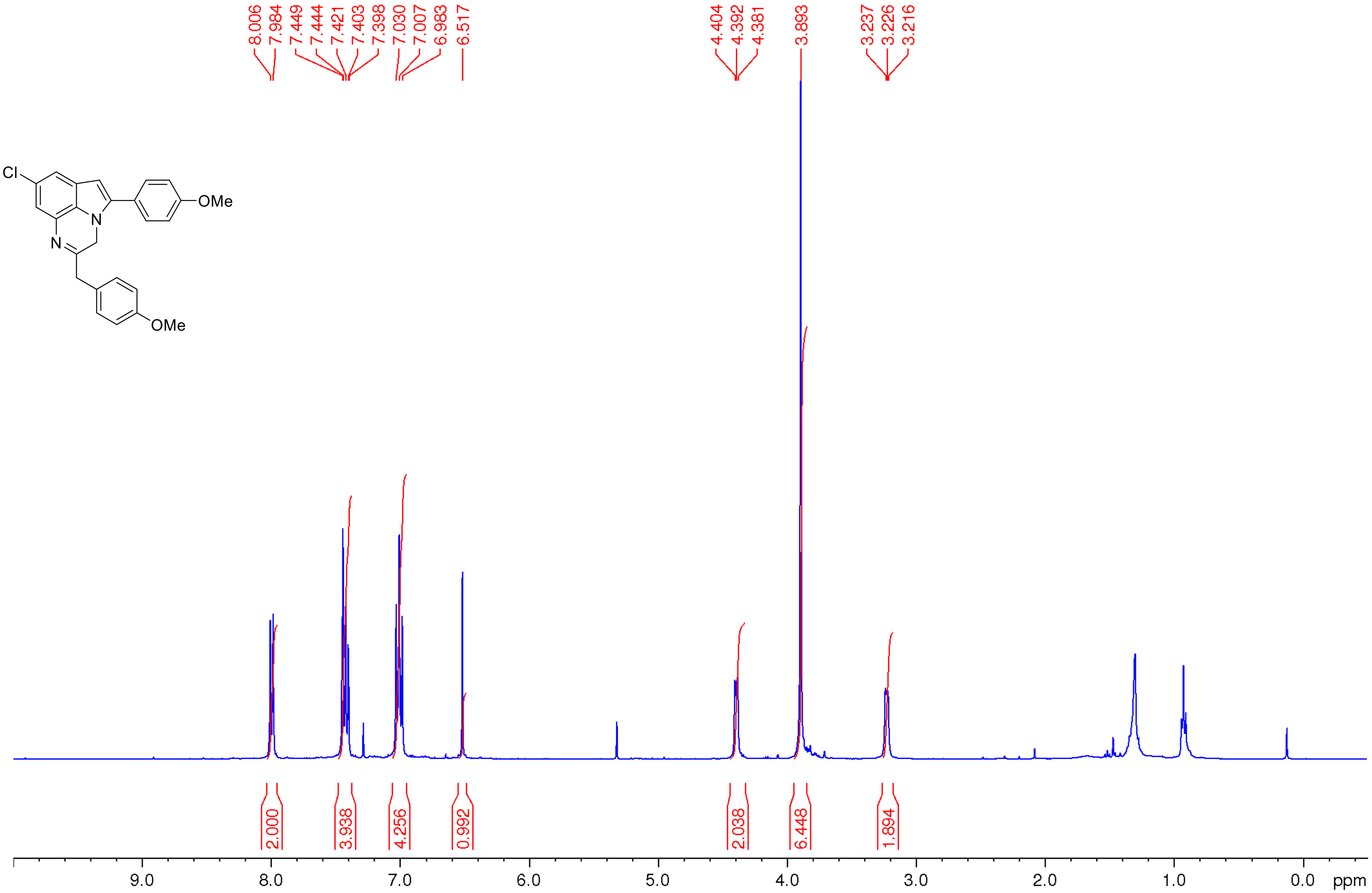

8-chloro-2-(4-methoxybenzyl)-5-(4-methoxyphenyl)-3*H*-pyrrolo[1,2,3-*de*]quinoxaline 2f

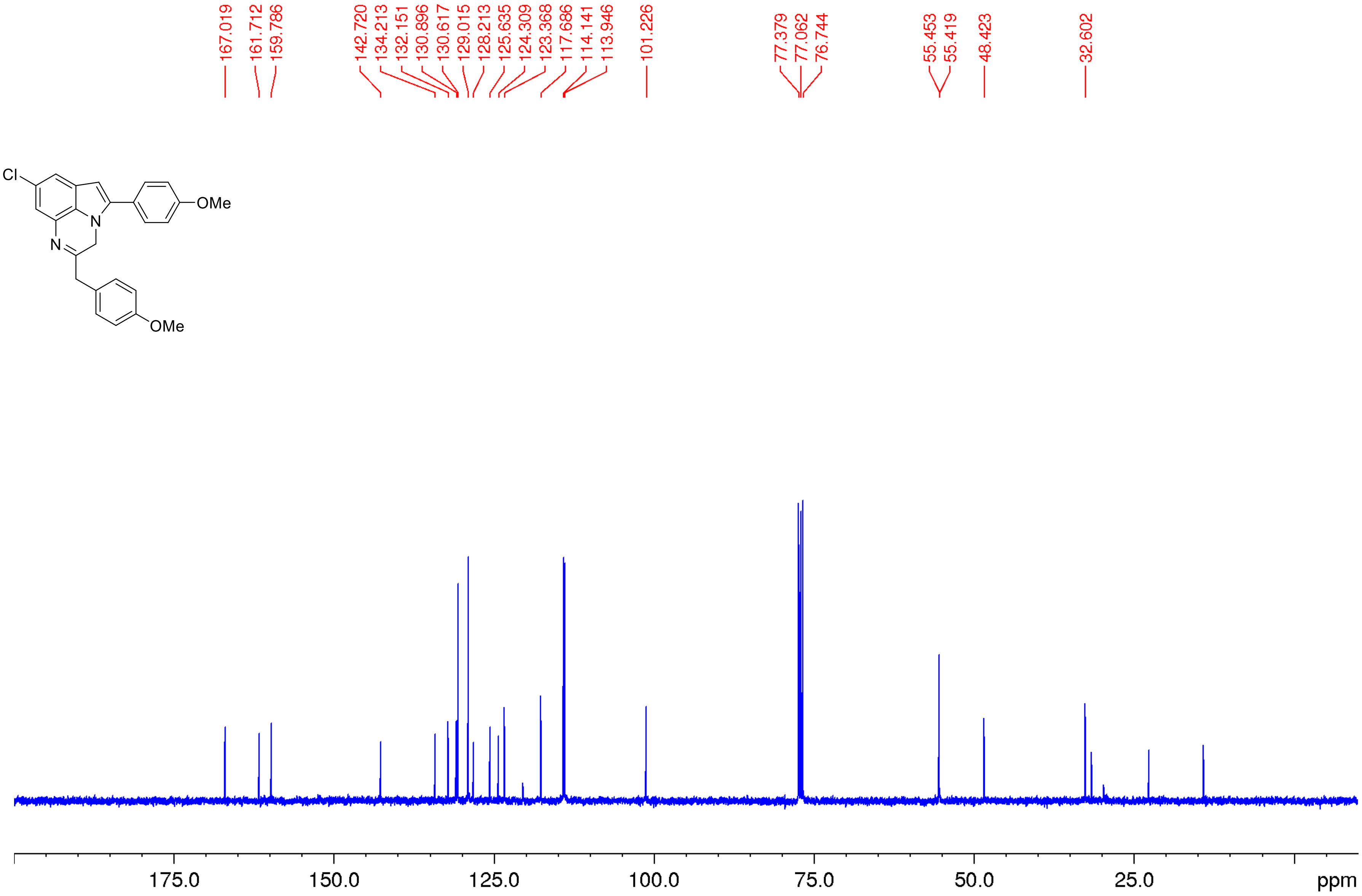

8-chloro-2-(4-methoxybenzyl)-5-(4-methoxyphenyl)-3*H*-pyrrolo[1,2,3-*de*]quinoxaline 2f

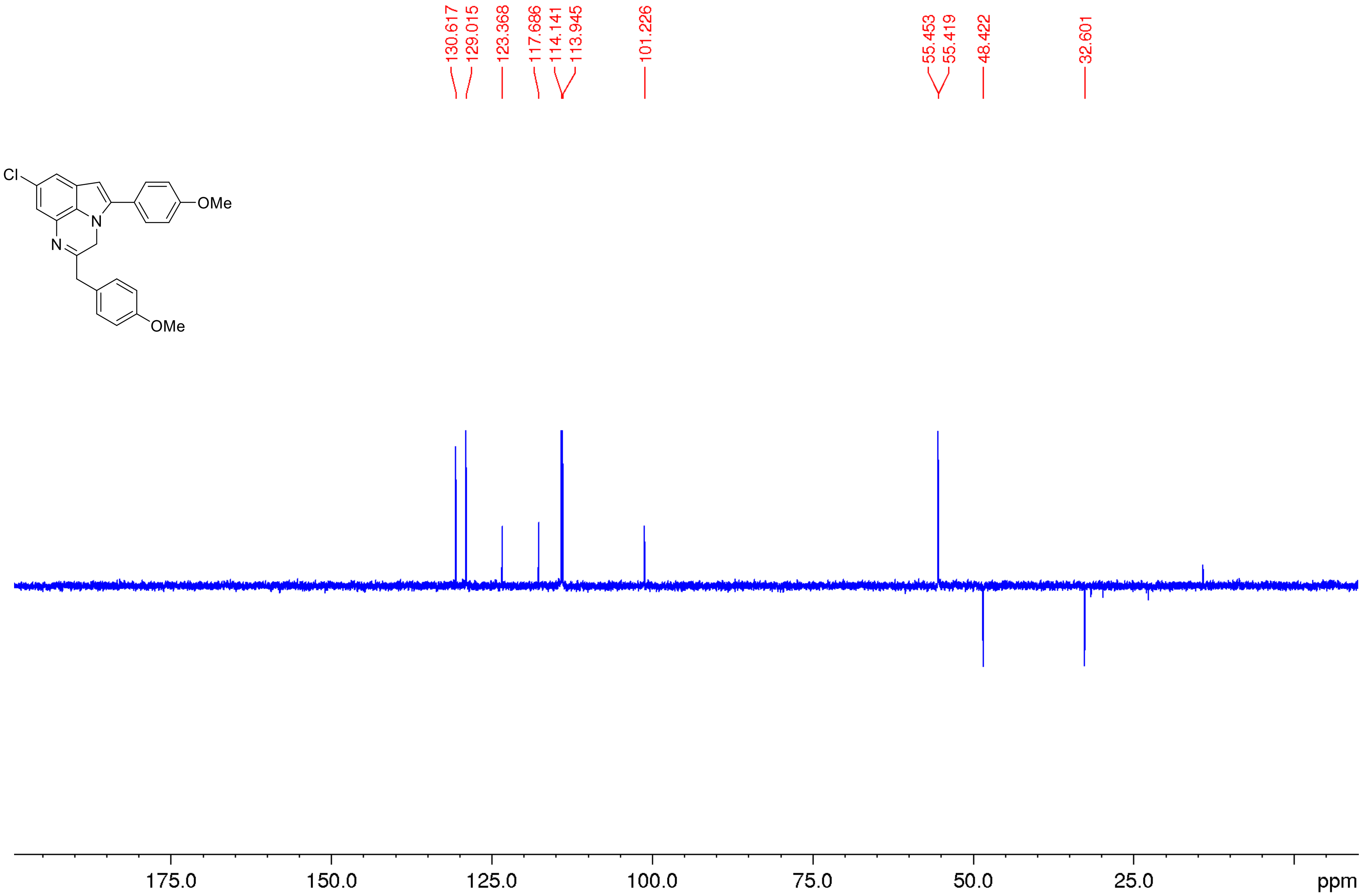

1-(4-((8-methyl-5-phenyl-3*H*-pyrrolo[1,2,3-*de*]quinoxalin-2-yl)methyl)phenyl)ethan-1-one 2g

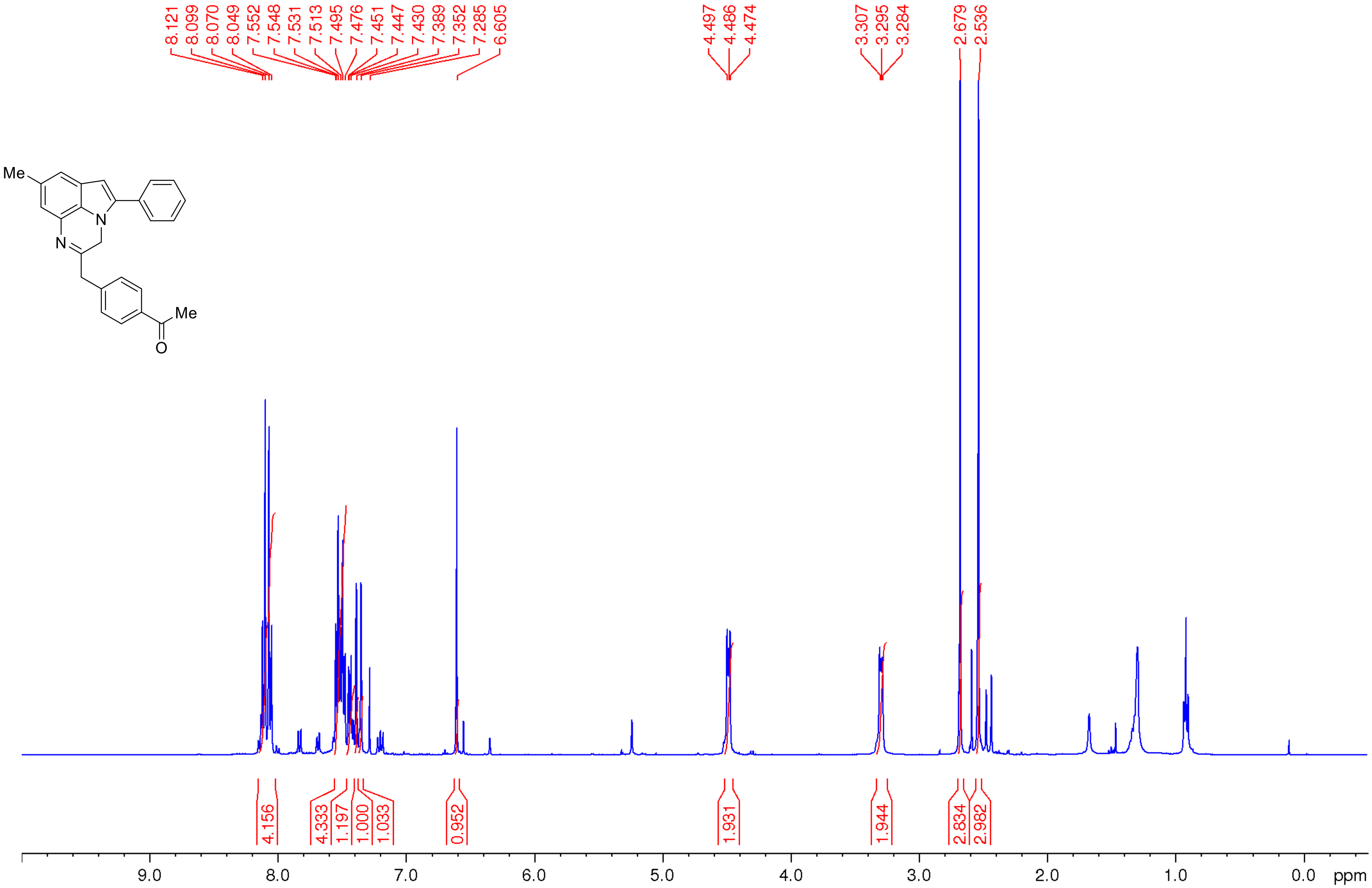

1-(4-((8-methyl-5-phenyl-3*H*-pyrrolo[1,2,3-*de*]quinoxalin-2-yl)methyl)phenyl)ethan-1-one 2g

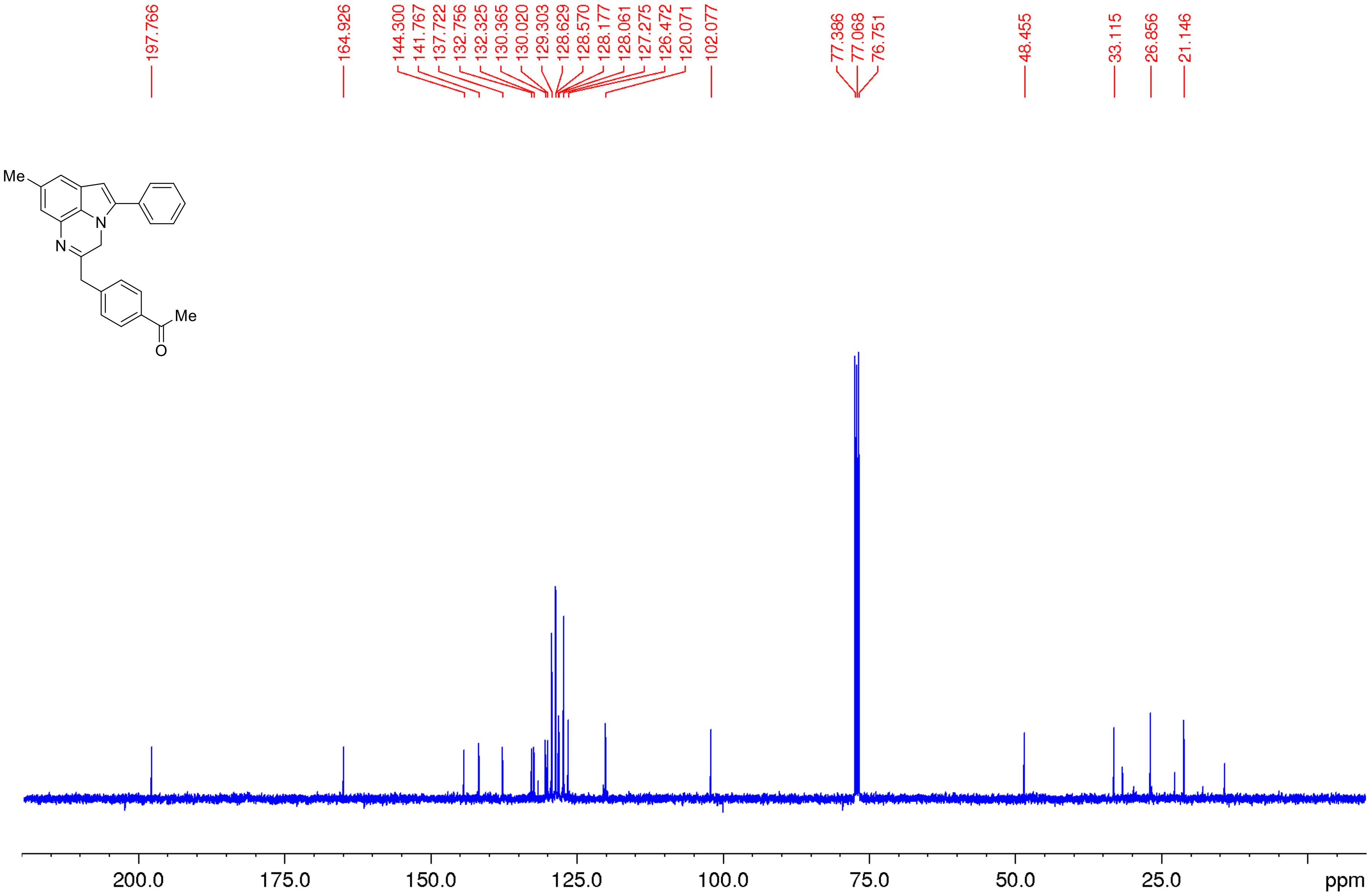

1-(4-((8-methyl-5-phenyl-3*H*-pyrrolo[1,2,3-*de*]quinoxalin-2-yl)methyl)phenyl)ethan-1-one 2g

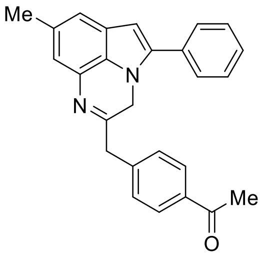

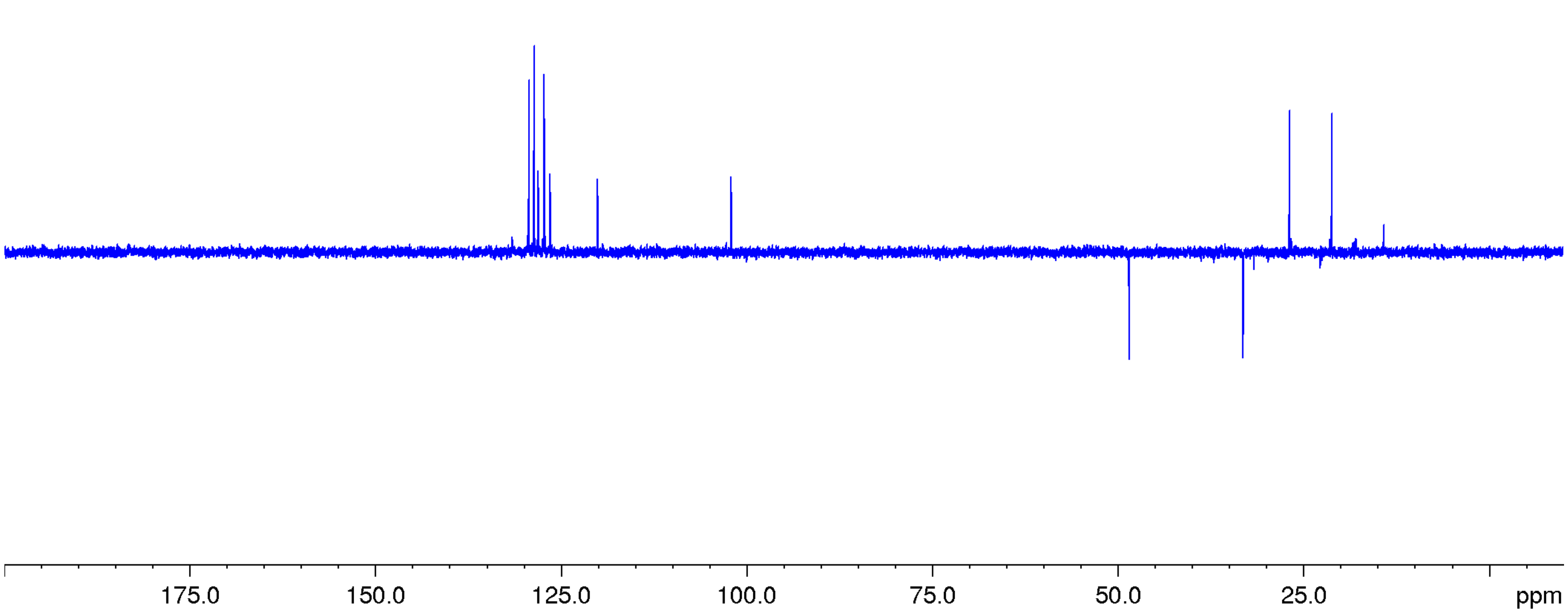

methyl 4-(2-(4-chlorobenzyl)-8-methyl-3*H*-pyrrolo[1,2,3-*de*]quinoxalin-5-yl)benzoate 2h

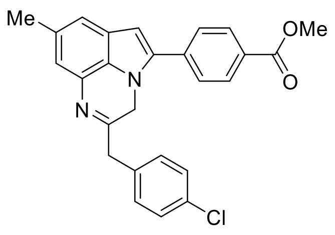

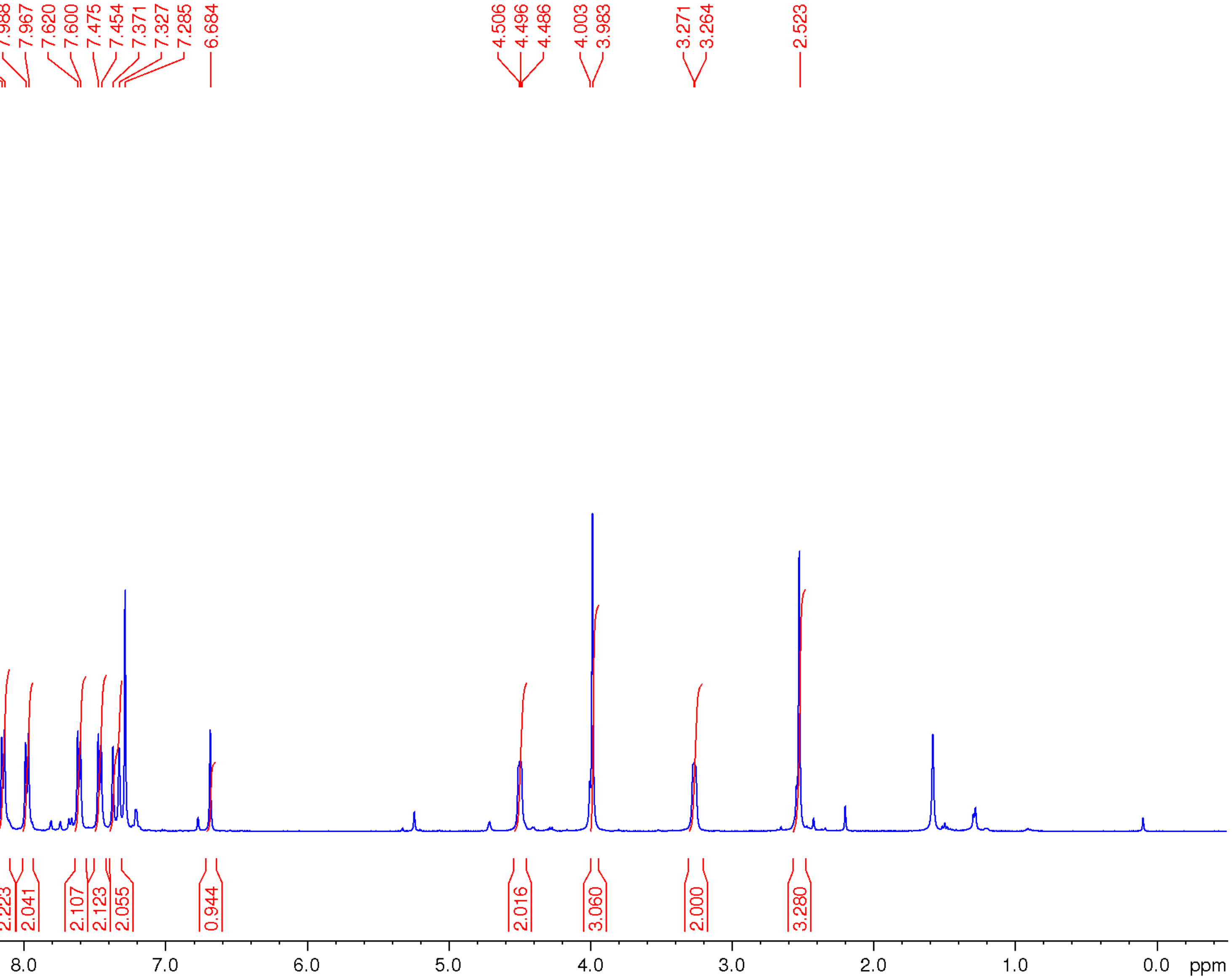

methyl 4-(2-(4-chlorobenzyl)-8-methyl-3*H*-pyrrolo[1,2,3-*de*]quinoxalin-5-yl)benzoate 2h

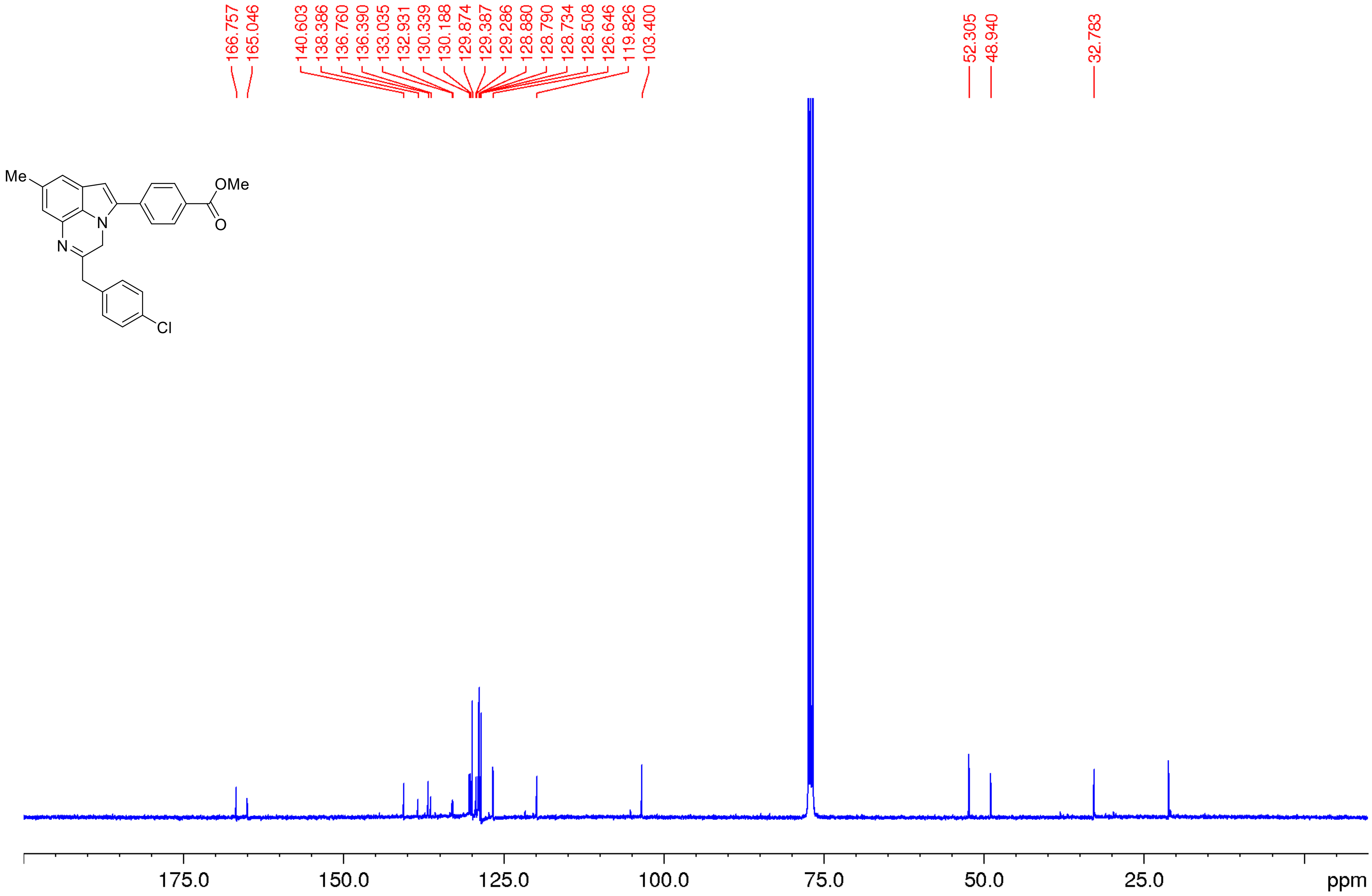

methyl 4-(2-(4-chlorobenzyl)-8-methyl-3*H*-pyrrolo[1,2,3-*de*]quinoxalin-5-yl)benzoate 2h

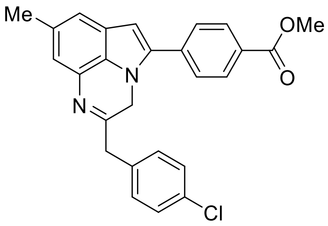

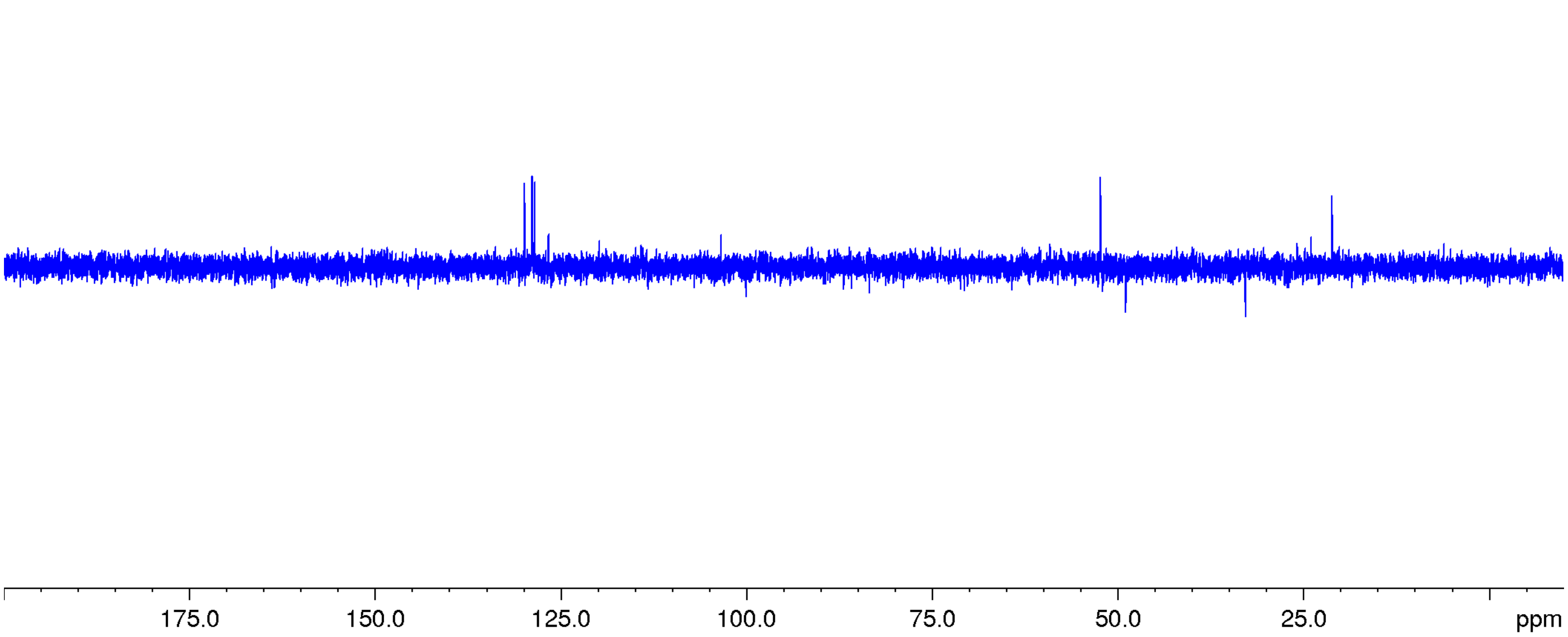

2-(4-chlorobenzyl)-8-methyl-5-phenyl-3*H*-pyrrolo[1,2,3-*de*]quinoxaline 2i

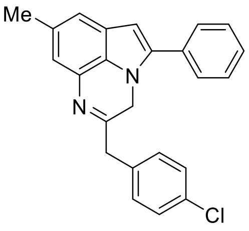

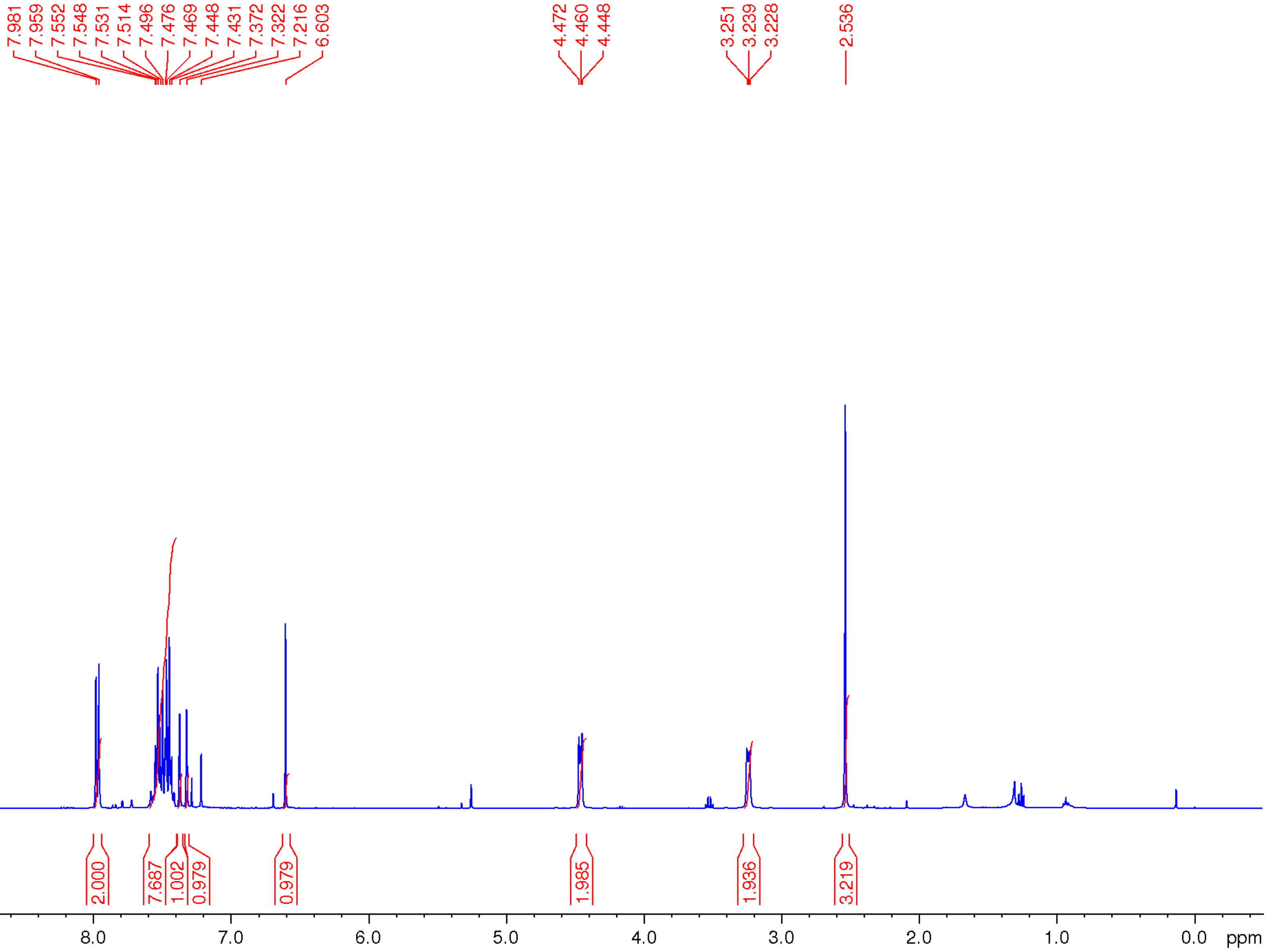

2-(4-chlorobenzyl)-8-methyl-5-phenyl-3*H*-pyrrolo[1,2,3-*de*]quinoxaline 2i

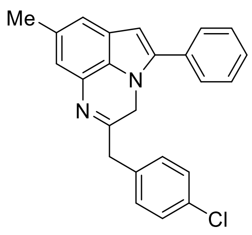

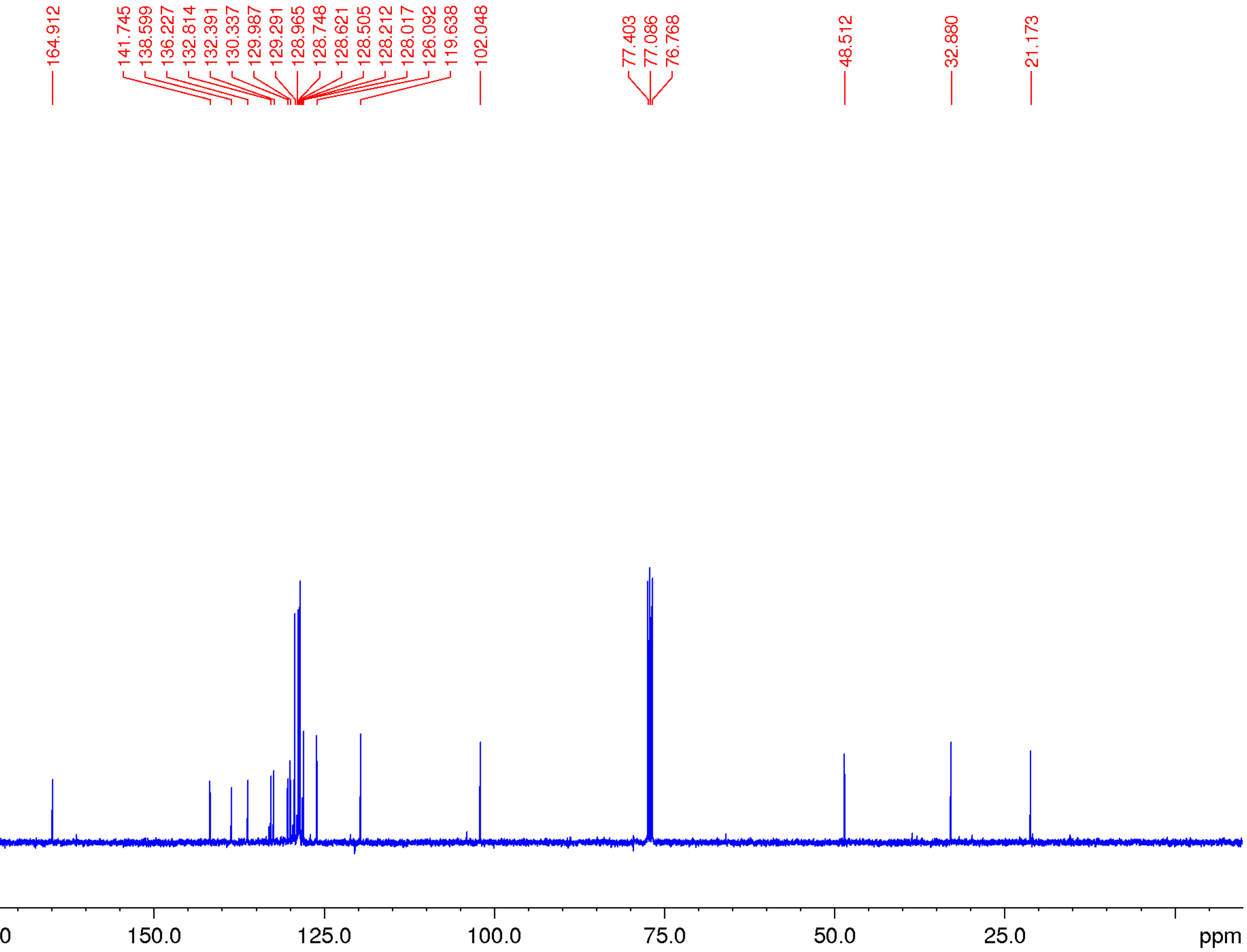

2-(4-chlorobenzyl)-8-methyl-5-phenyl-3*H*-pyrrolo[1,2,3-*de*]quinoxaline 2i

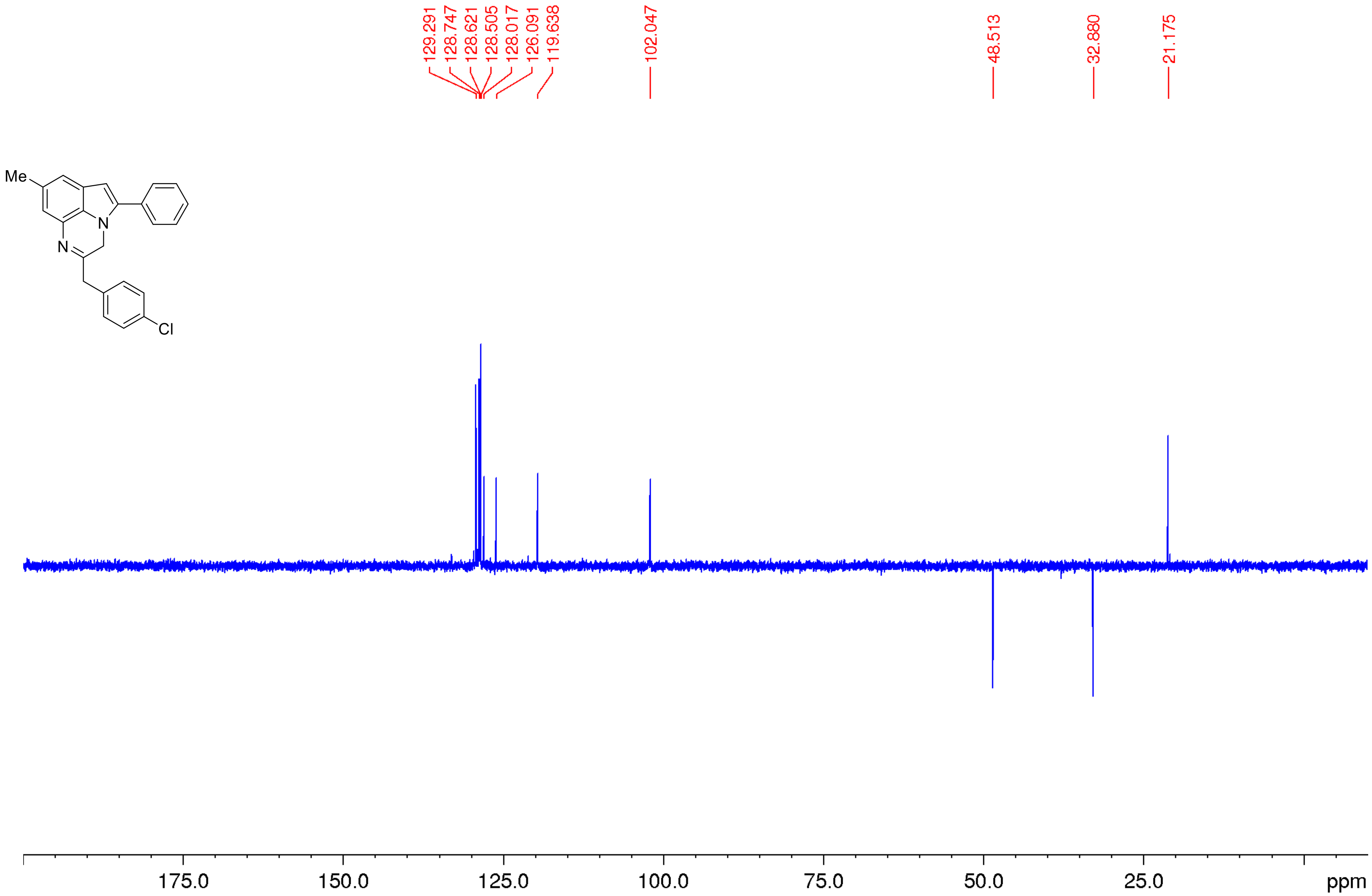

1-(4-((6-(4-methoxyphenyl)-8-methyl-5-phenyl-3*H*-pyrrolo[1,2,3-de]quinoxalin-2-yl)methyl)phenyl)ethan-1-one 2k

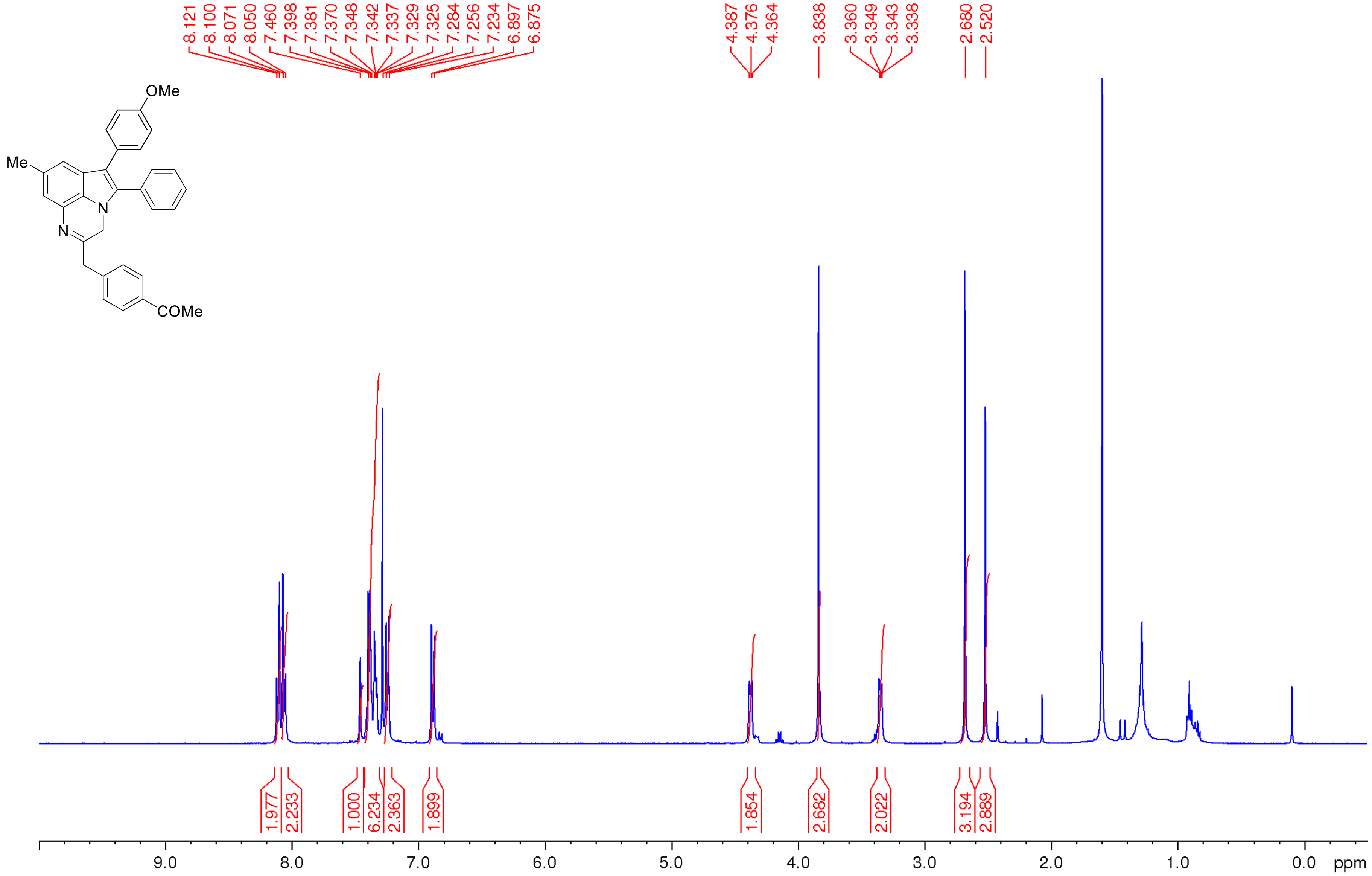

1-(4-((6-(4-methoxyphenyl)-8-methyl-5-phenyl-3*H*-pyrrolo[1,2,3-*de*]quinoxalin-2-yl)methyl)phenyl)ethan-1-one 2k

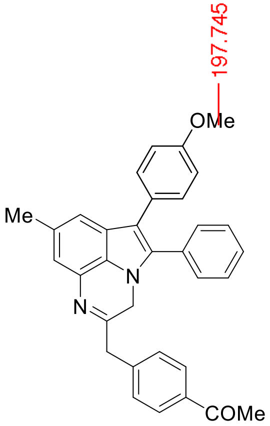

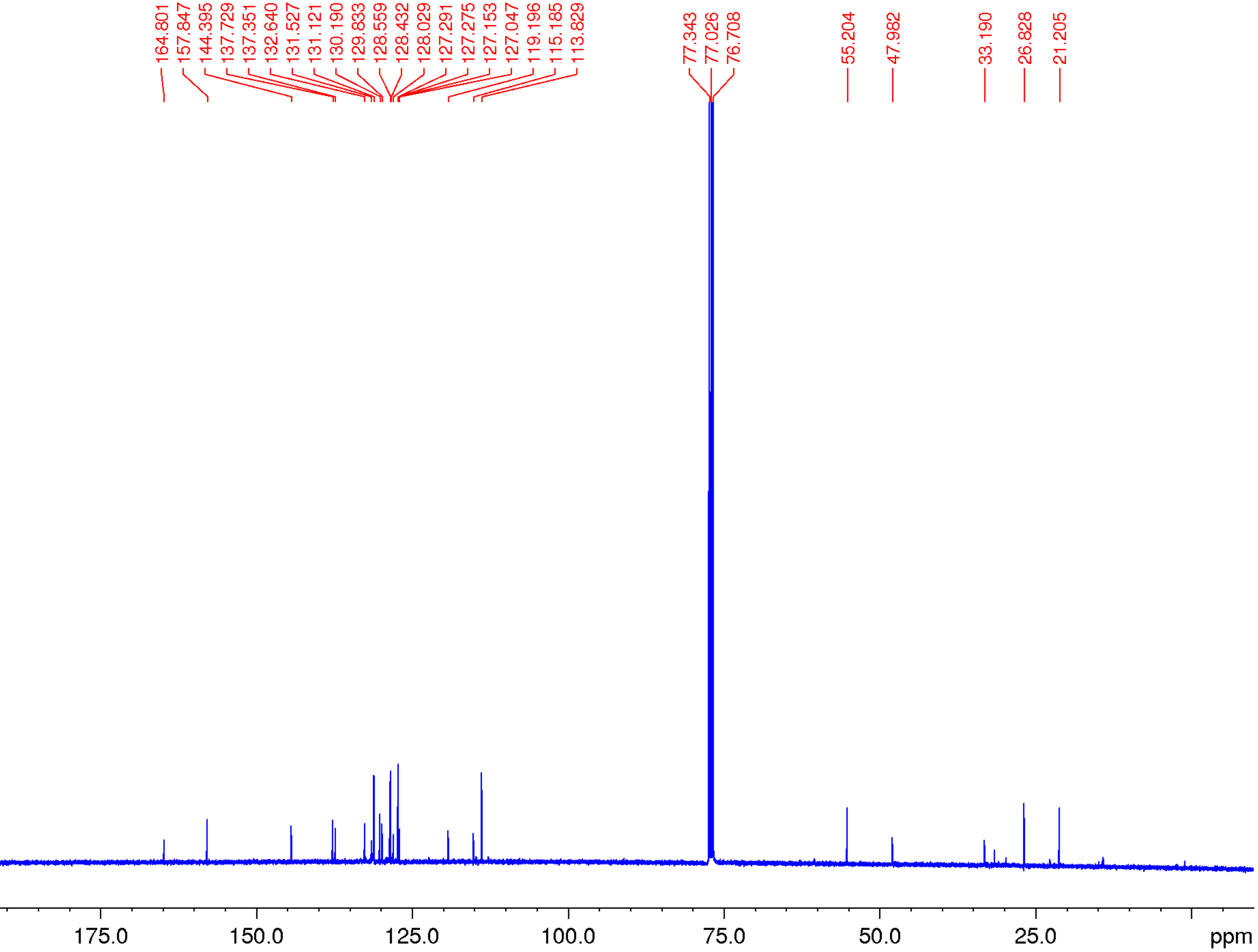

1-(4-((6-(4-methoxyphenyl)-8-methyl-5-phenyl-3*H*-pyrrolo[1,2,3-*de*]quinoxalin-2-yl)methyl)phenyl)ethan-1-one 2k

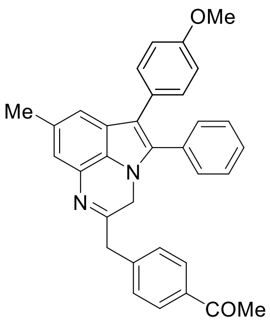

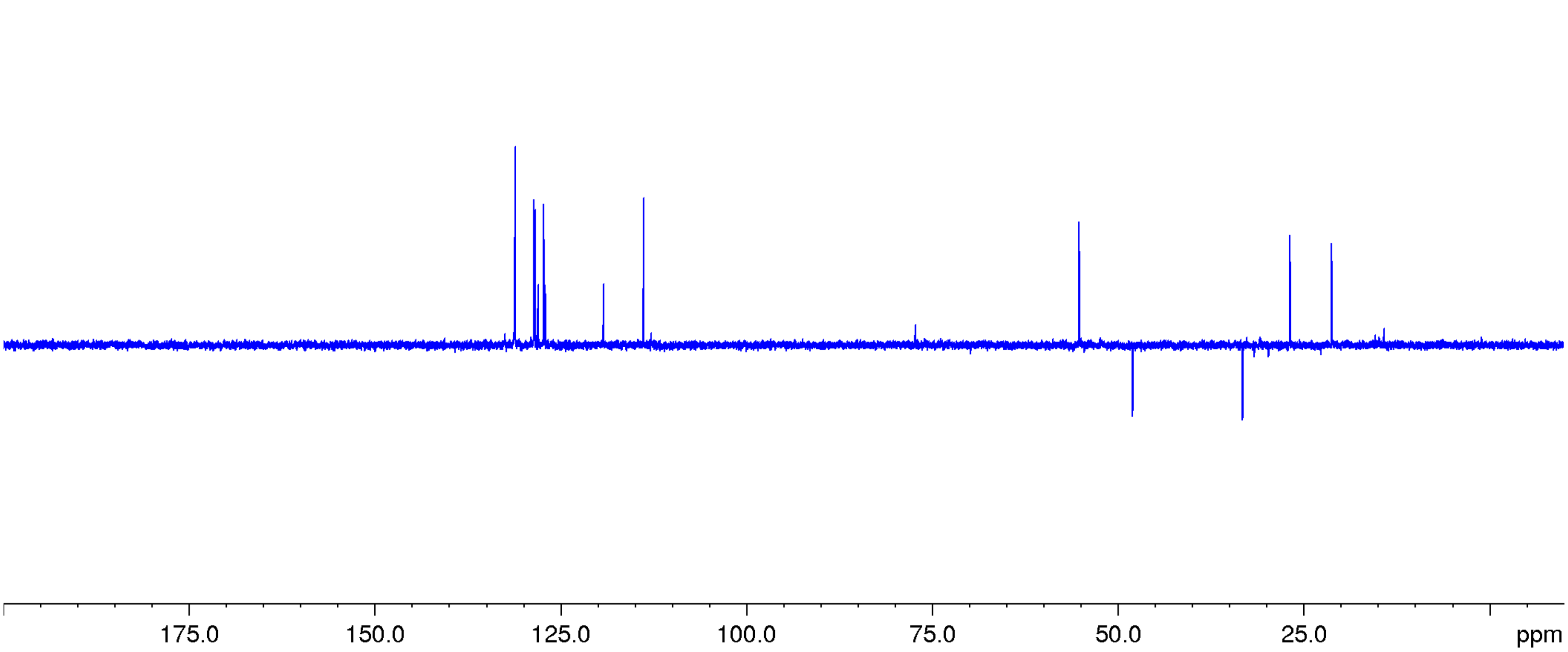

8-chloro-2-(4-methoxybenzyl)-5-phenyl-2,3-dihydro-1*H*-pyrrolo[1,2,3-*de*]quinoxaline 9d

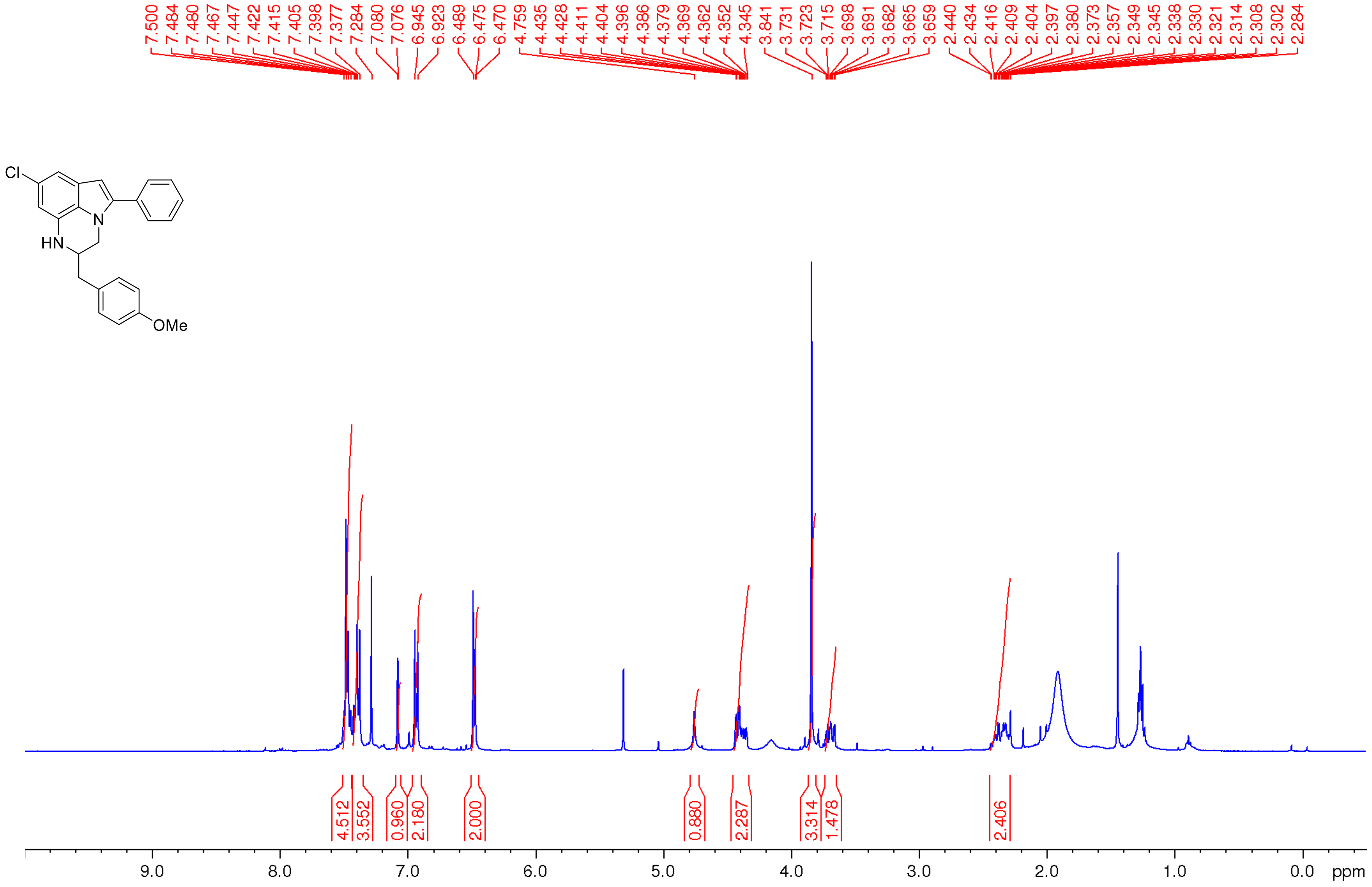

8-chloro-2-(4-methoxybenzyl)-5-phenyl-2,3-dihydro-1*H*-pyrrolo[1,2,3-*de*]quinoxaline 9d

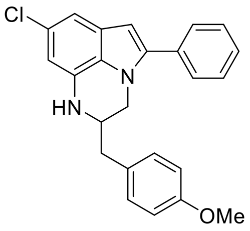

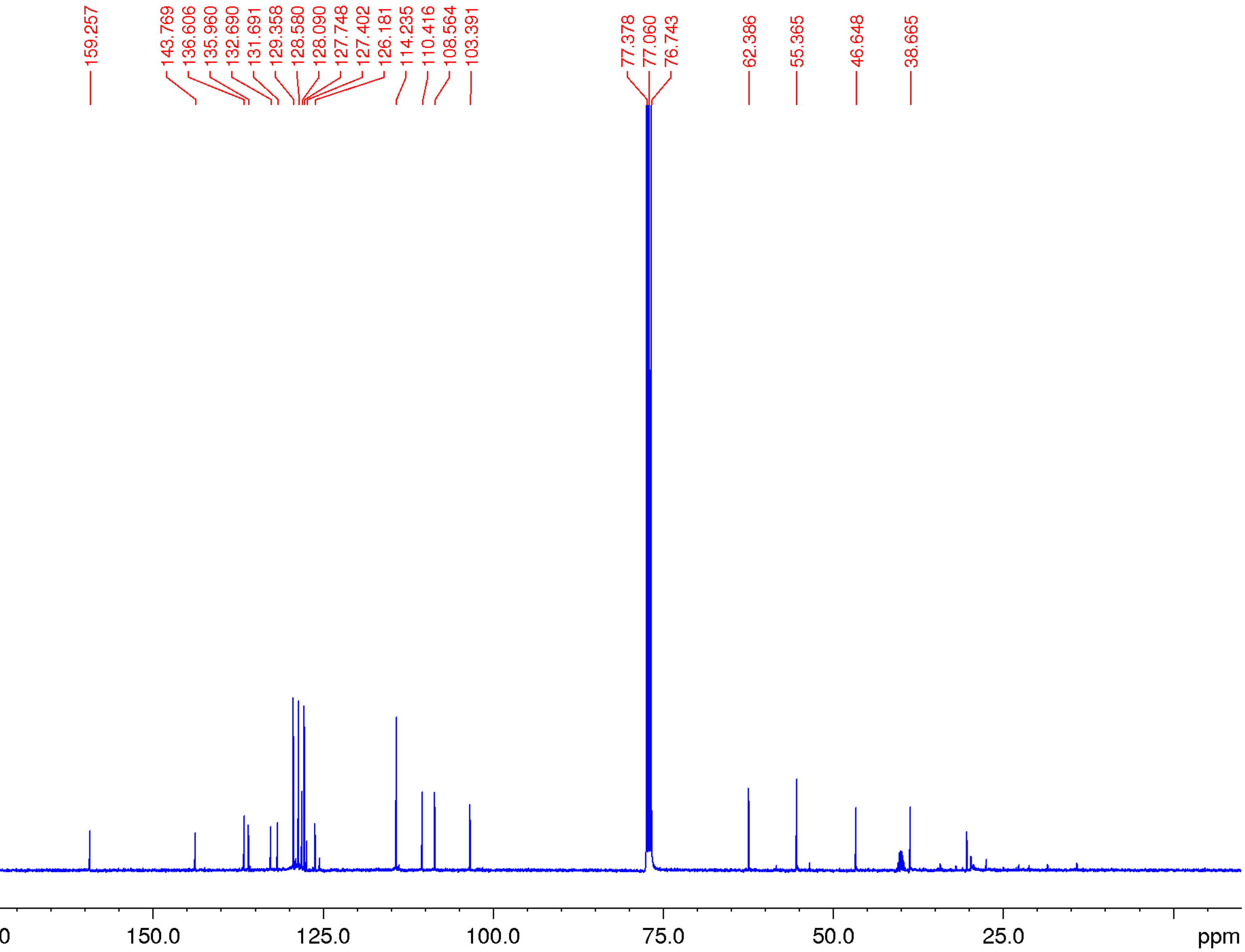

8-chloro-2-(4-methoxybenzyl)-5-phenyl-2,3-dihydro-1*H*-pyrrolo[1,2,3-*de*]quinoxaline 9d

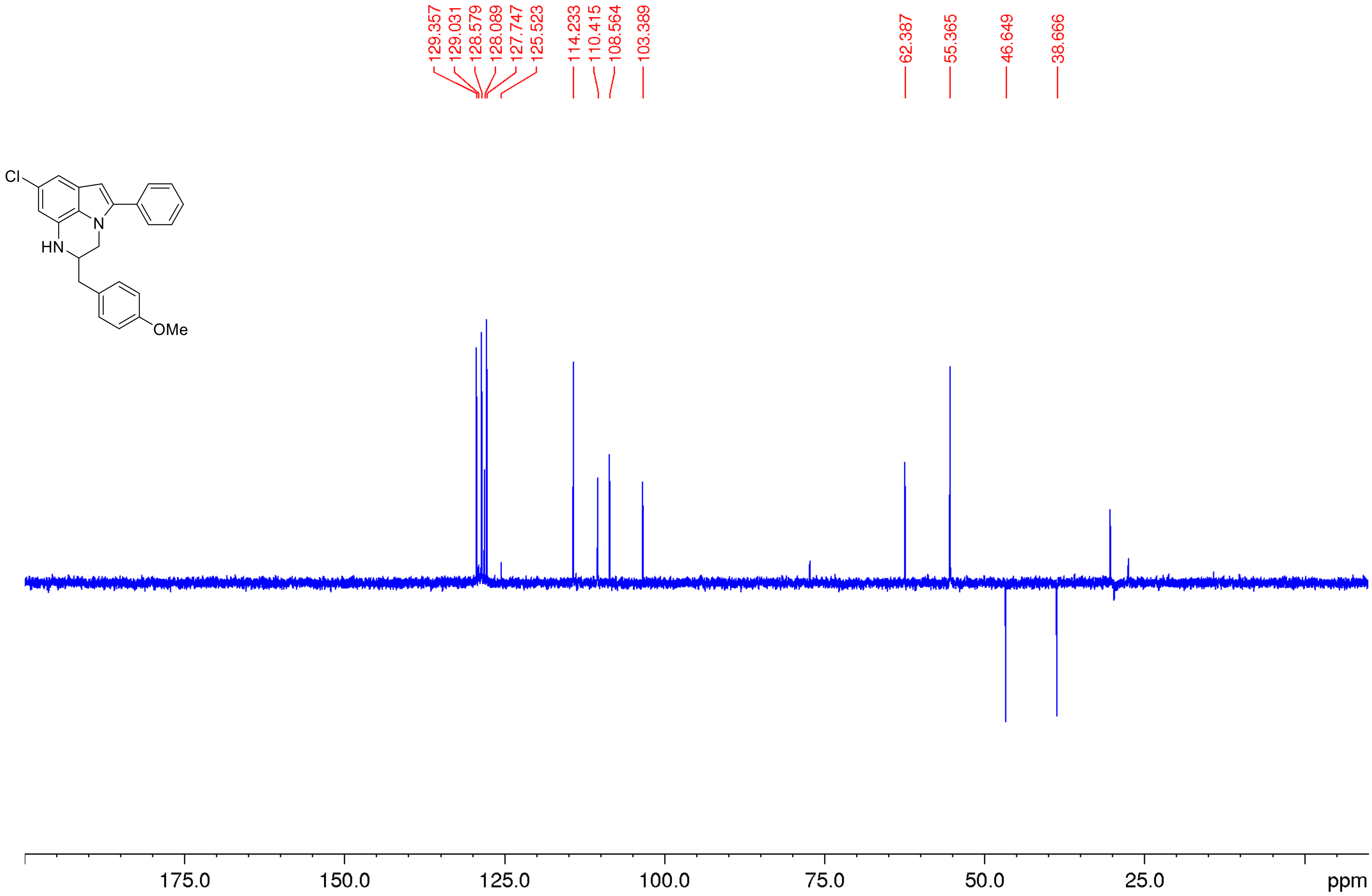

5-chloro-7-nitro-2-phenyl-1*H*-indole 5a

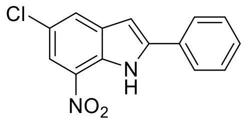

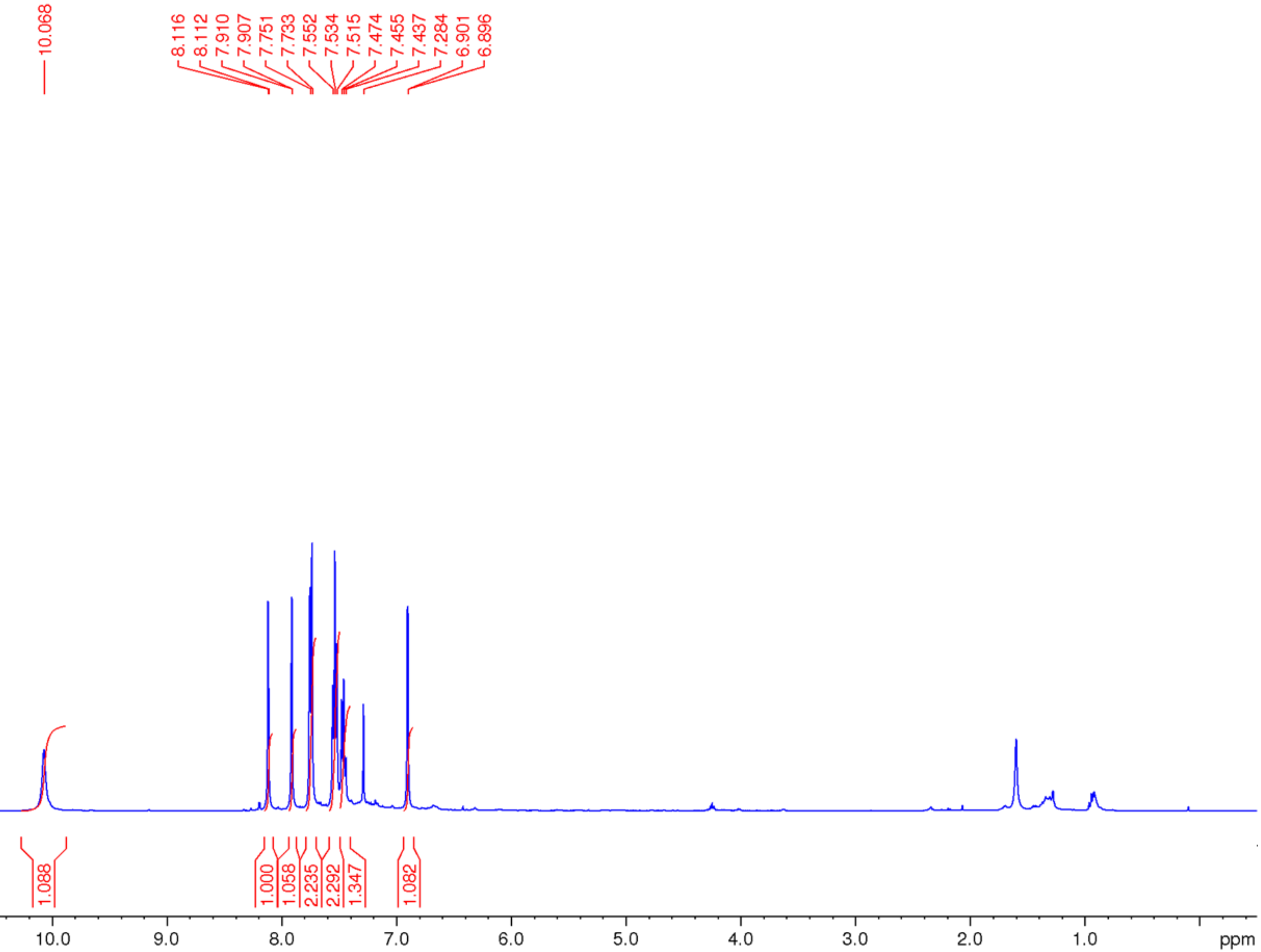

5-chloro-7-nitro-2-phenyl-1*H*-indole 5a

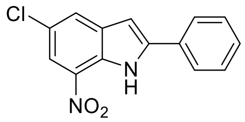

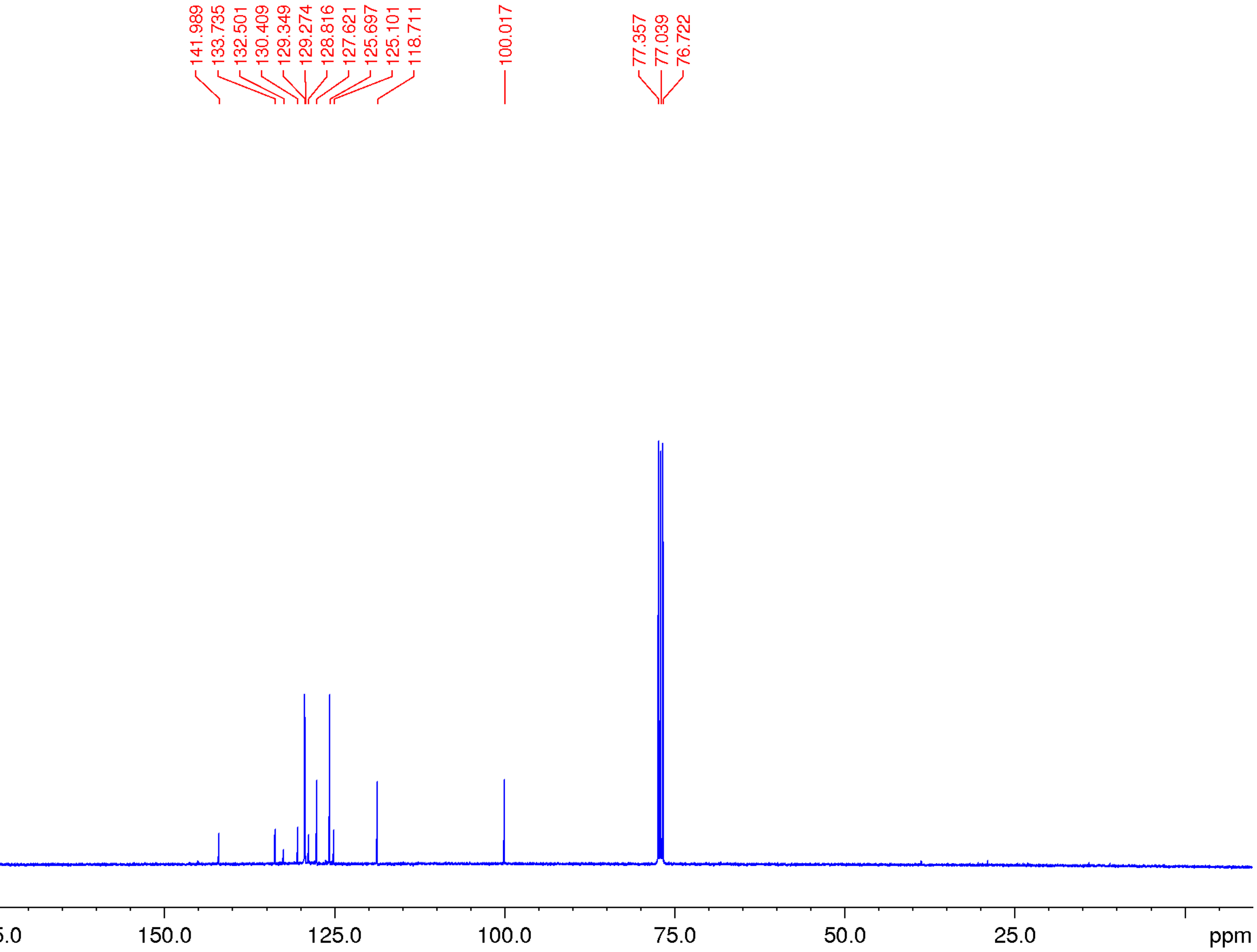

5-chloro-7-nitro-2-phenyl-1*H*-indole 5a

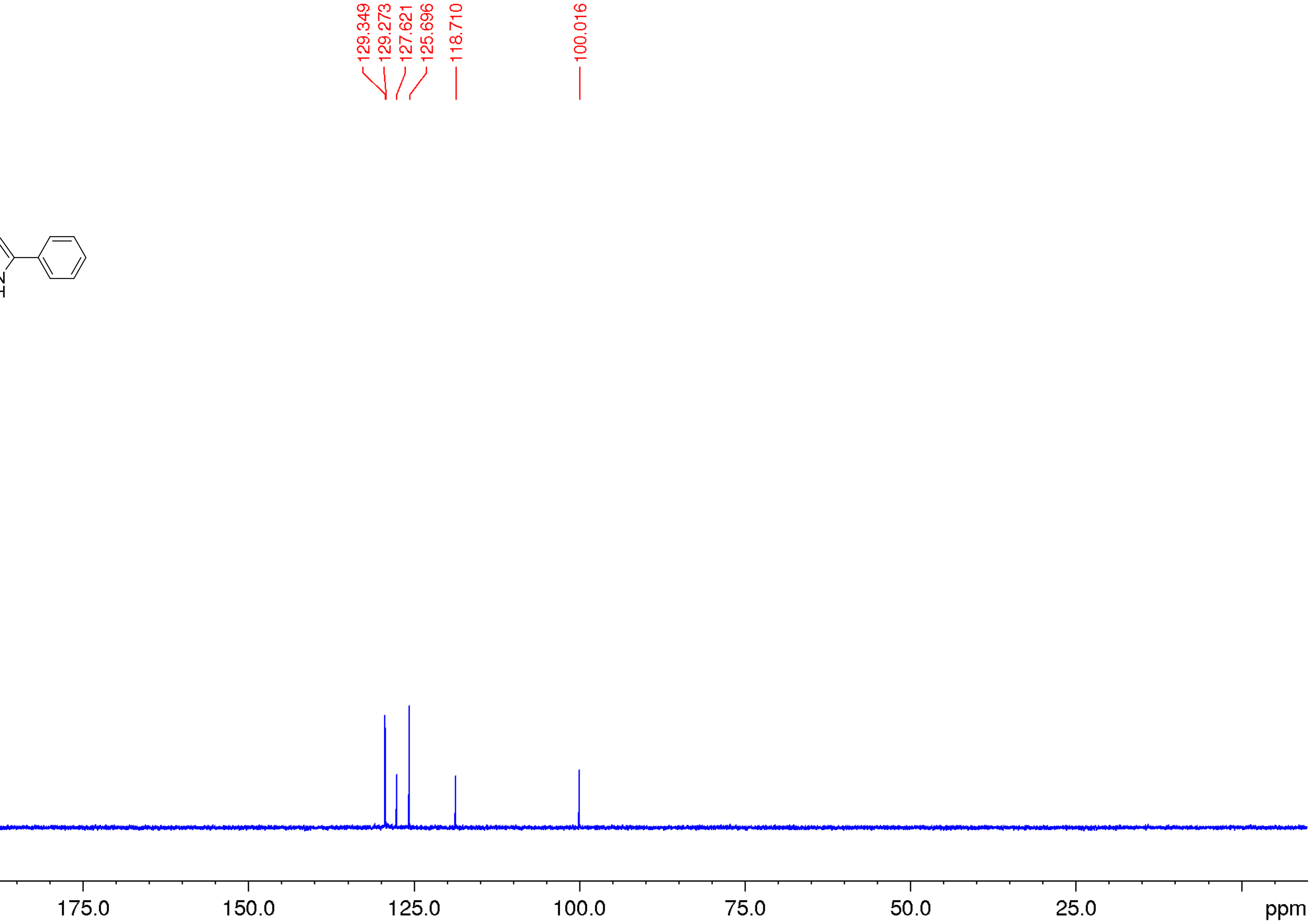

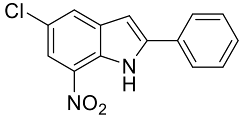

5-chloro-2-(4-methoxyphenyl)-7-nitro-1*H*-indole 5b

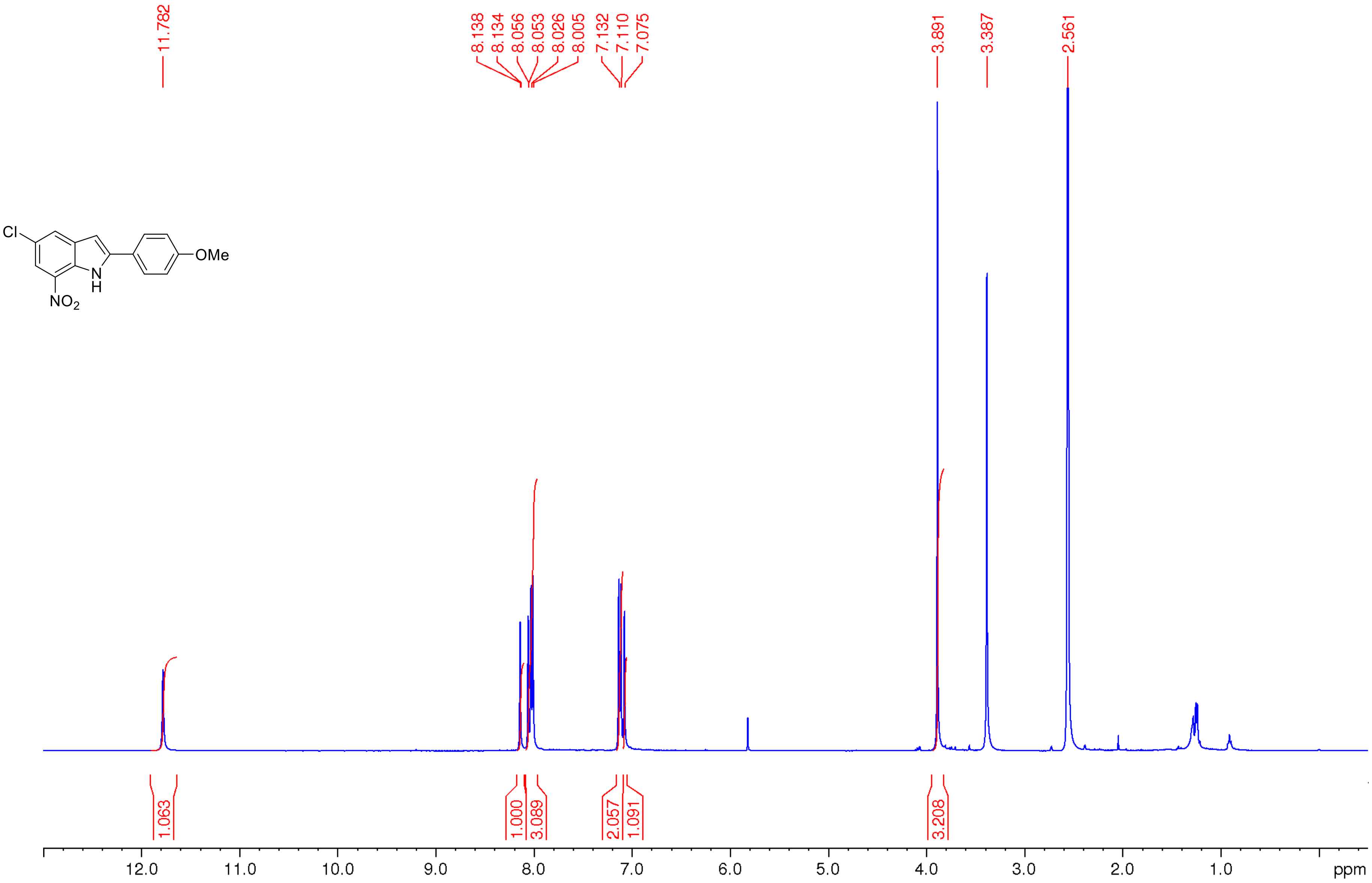

5-chloro-2-(4-methoxyphenyl)-7-nitro-1*H*-indole 5b

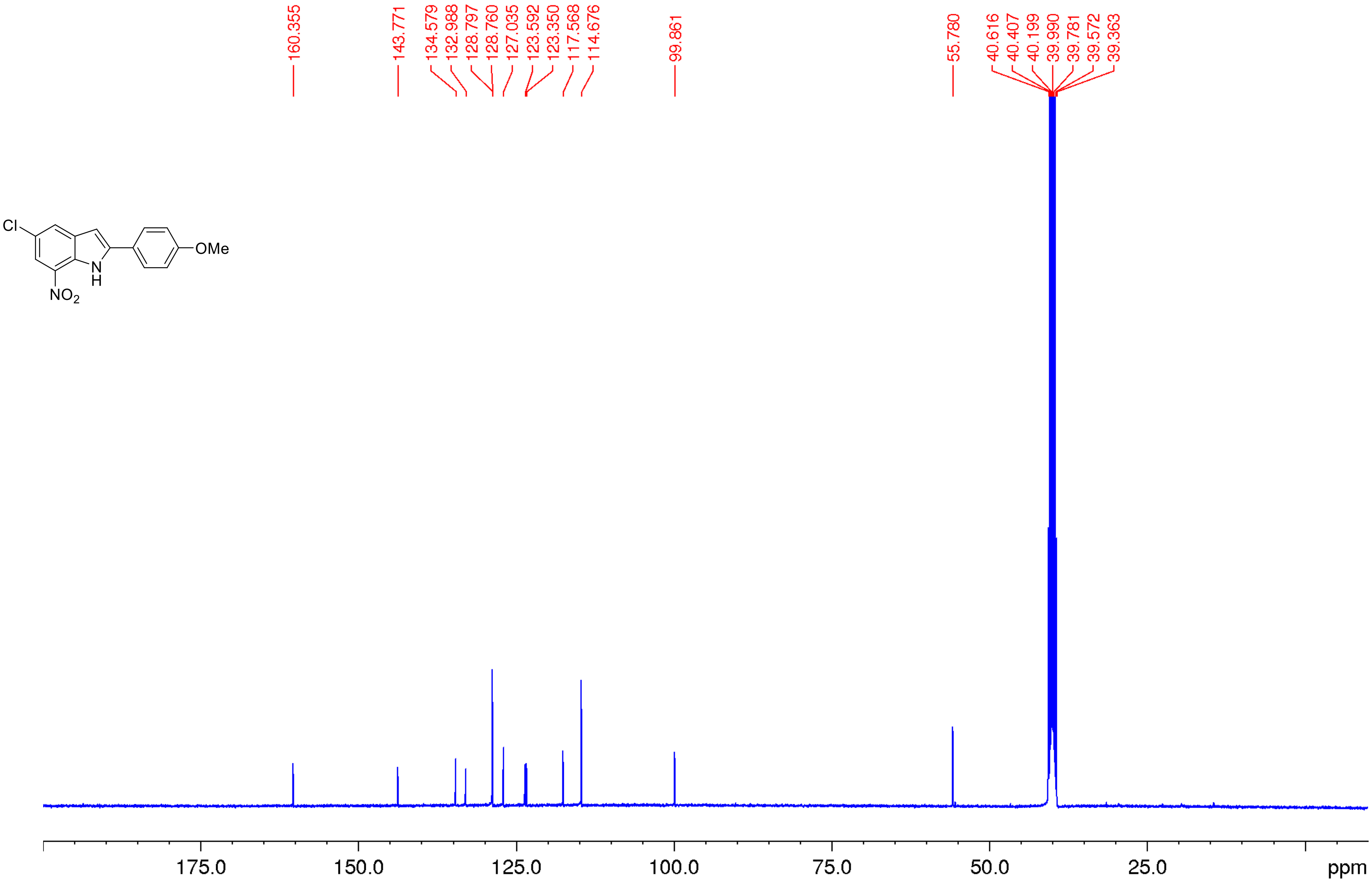

5-chloro-2-(4-methoxyphenyl)-7-nitro-1*H*-indole 5b

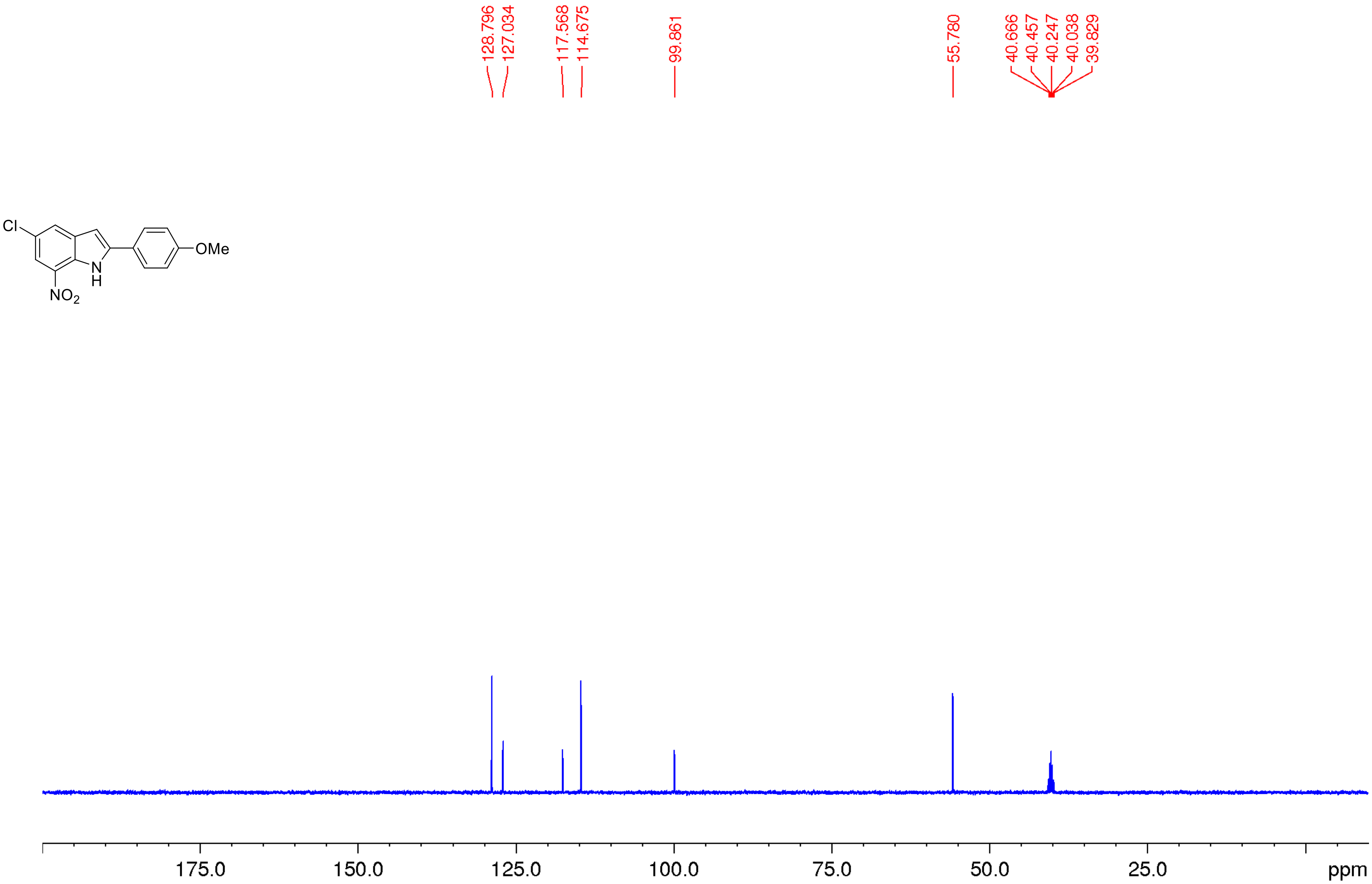

5-methyl-7-nitro-2-phenyl-1*H*-indole 5c

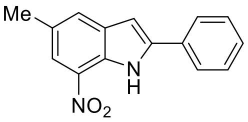

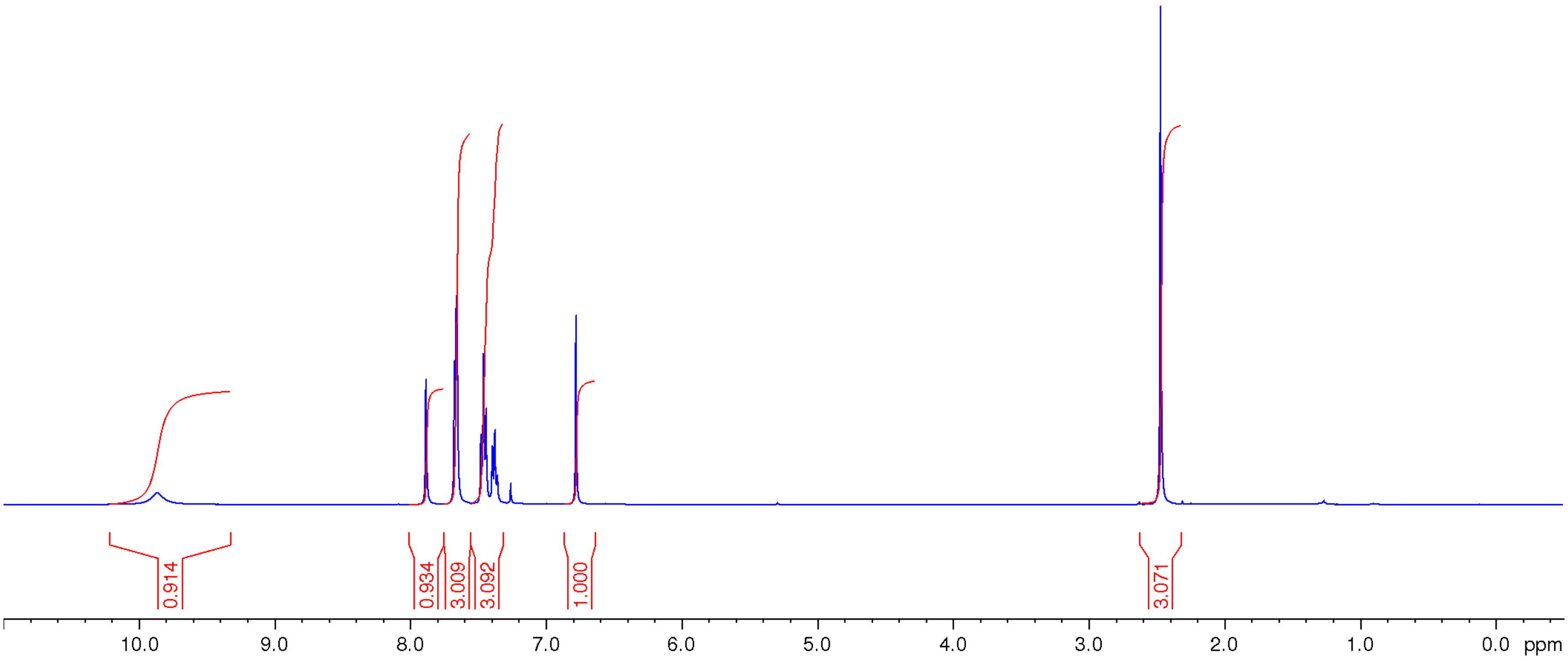

5-methyl-7-nitro-2-phenyl-1*H*-indole 5c

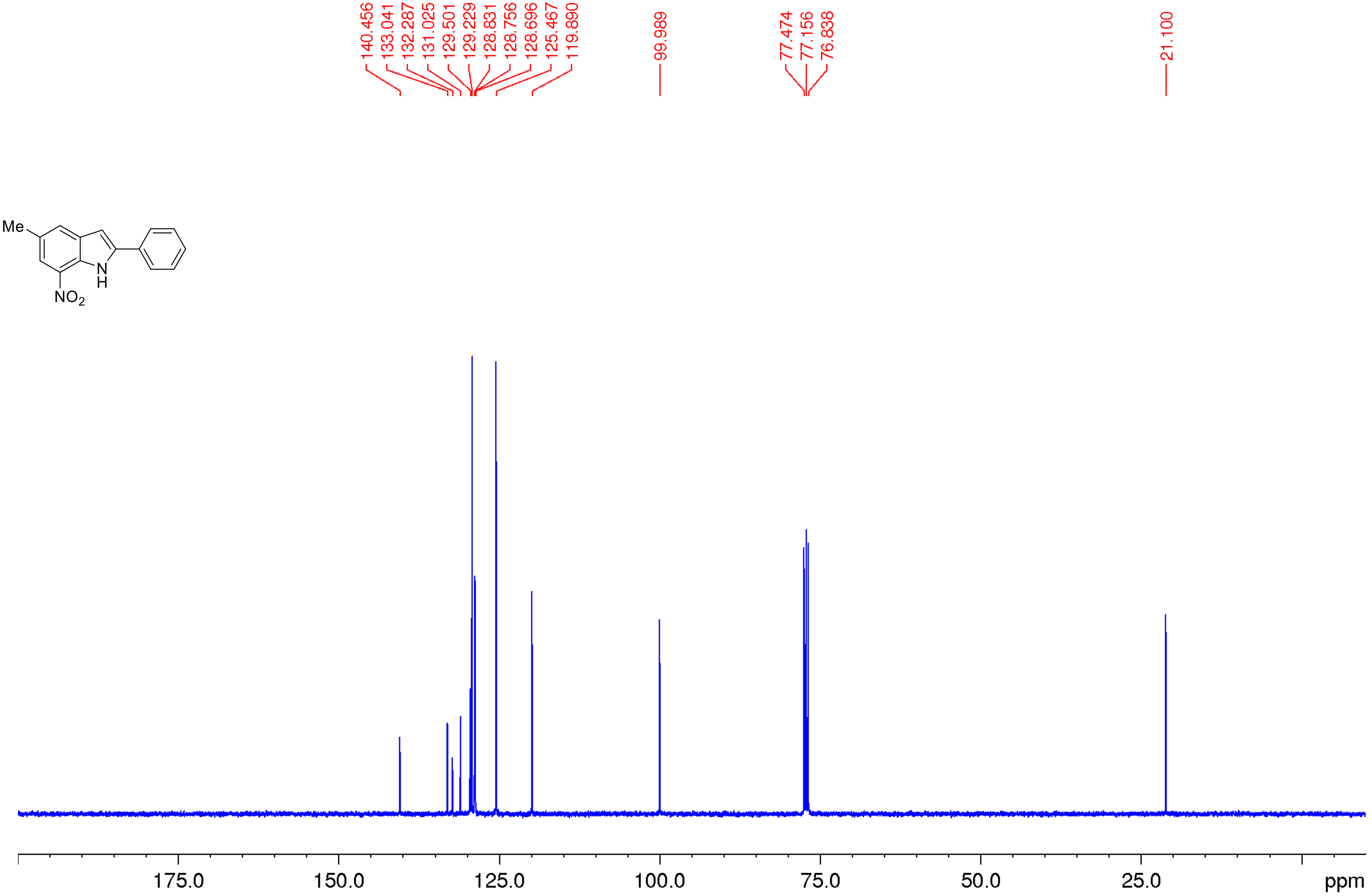

5-methyl-7-nitro-2-phenyl-1*H*-indole 5c

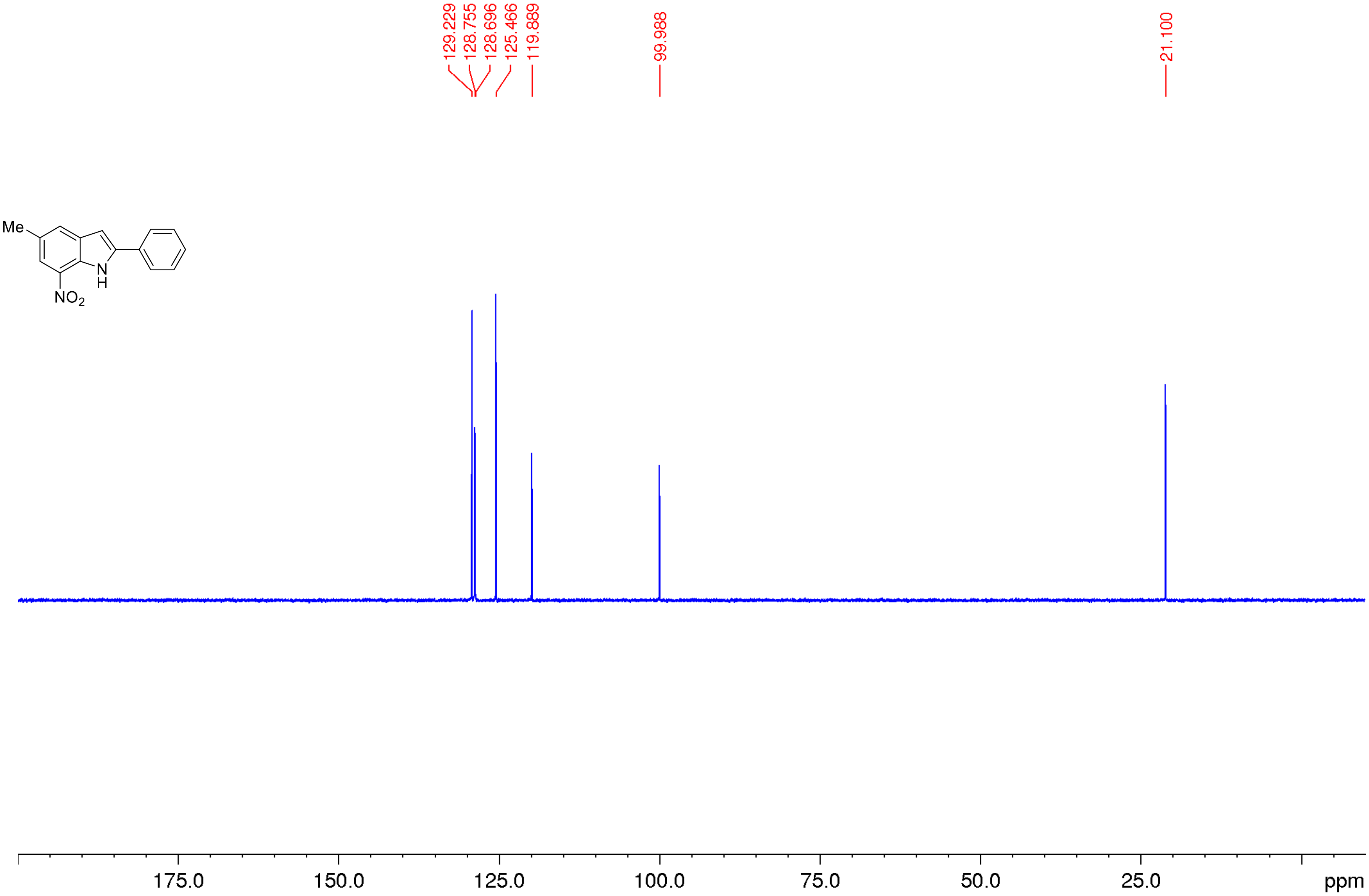

methyl 4-(5-methyl-7-nitro-1*H*-indol-2-yl)benzoate 5d

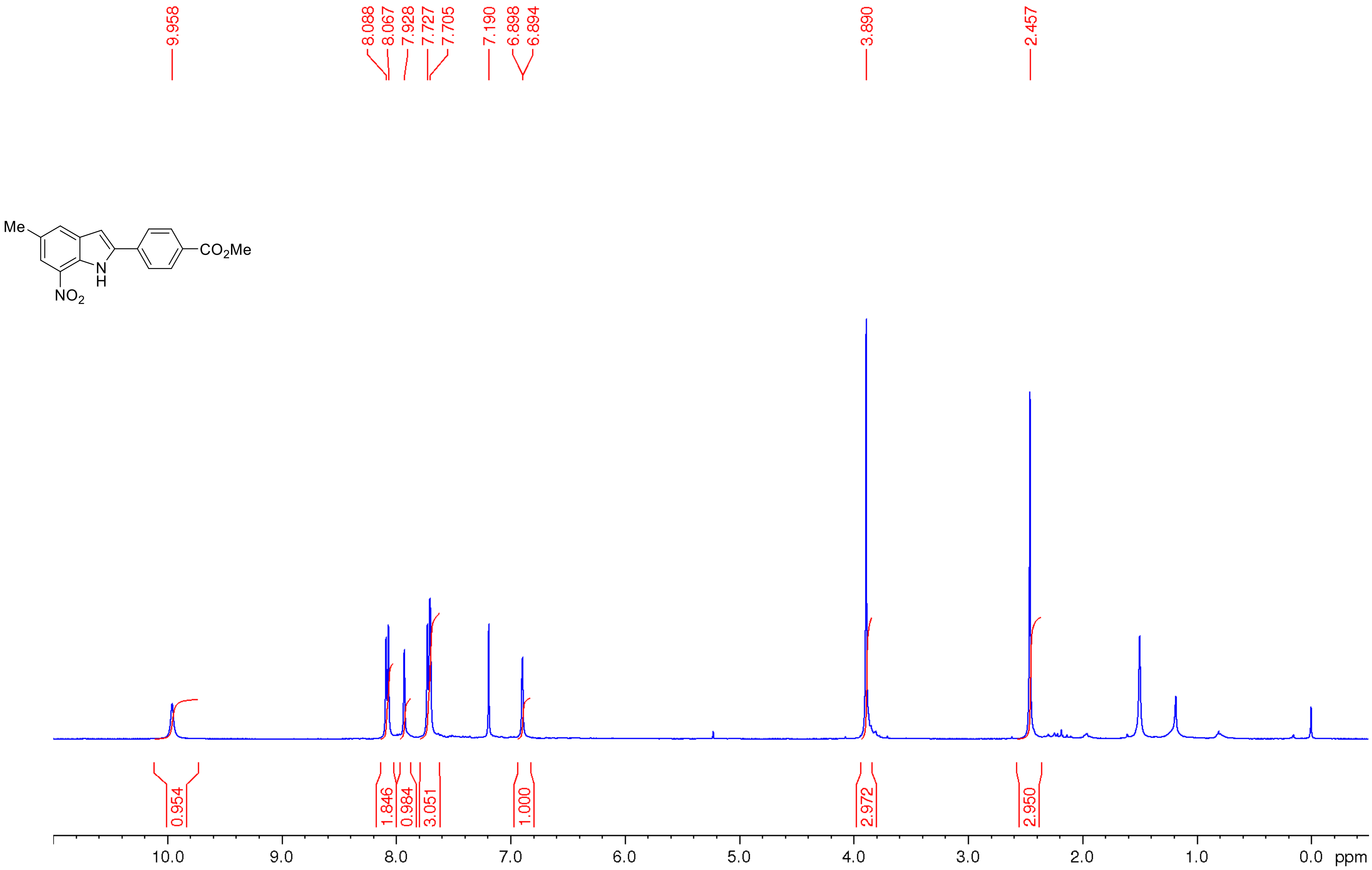

methyl 4-(5-methyl-7-nitro-1*H*-indol-2-yl)benzoate 5d

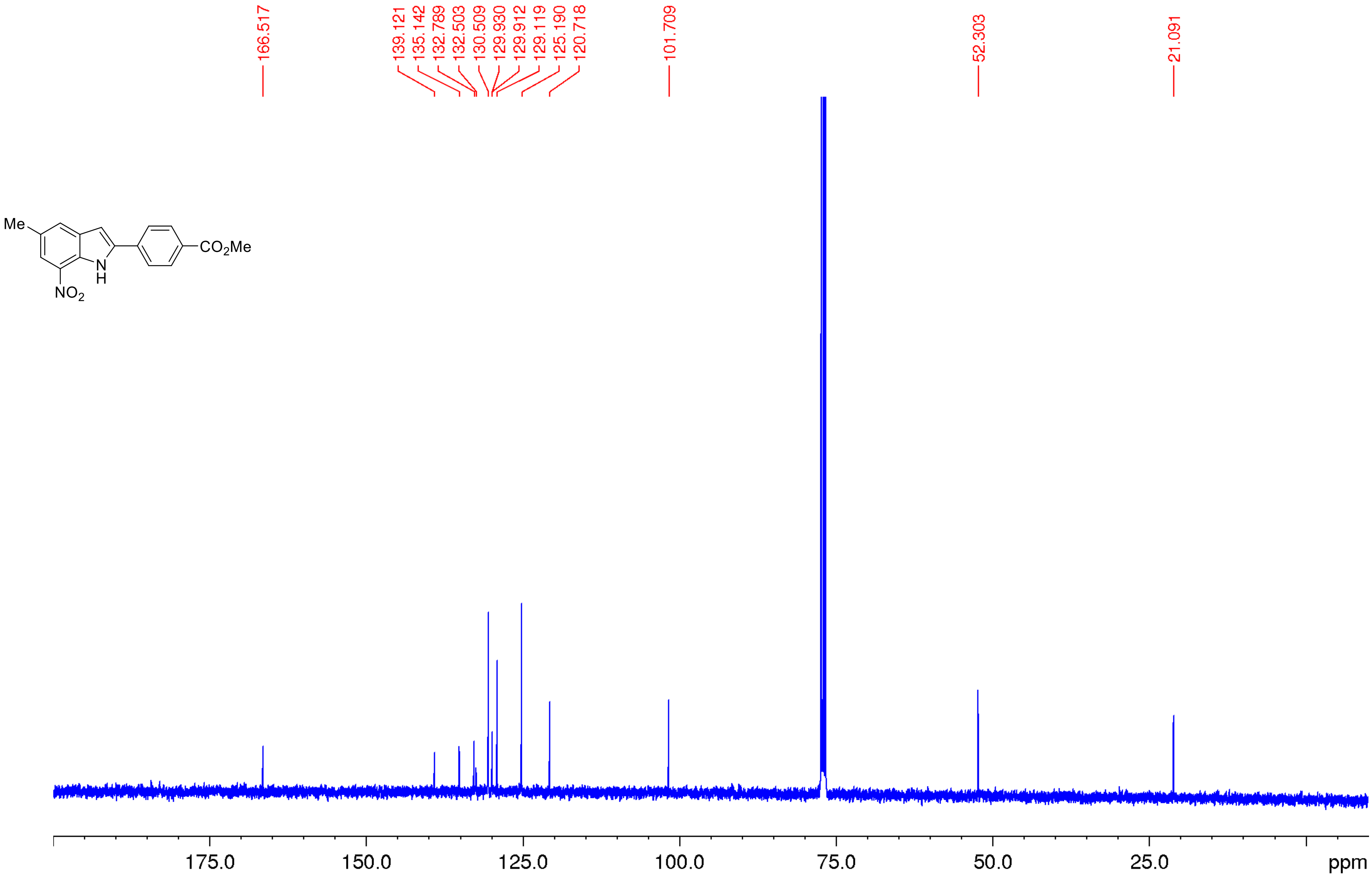

methyl 4-(5-methyl-7-nitro-1*H*-indol-2-yl)benzoate 5d

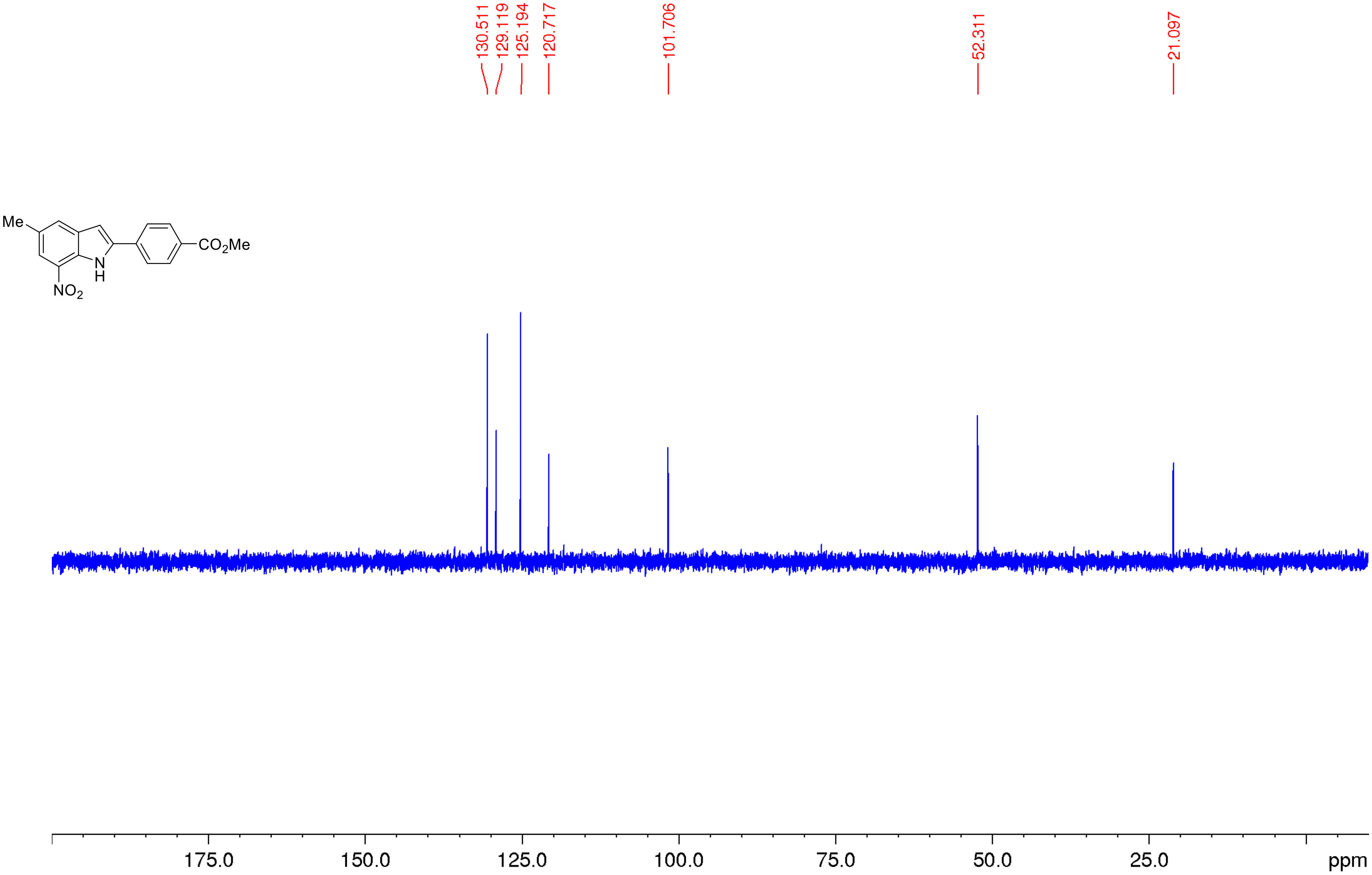

5-chloro-7-nitro-2-phenyl-1-(prop-2-yn-1-yl)-1*H*-indole 6a

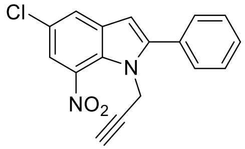

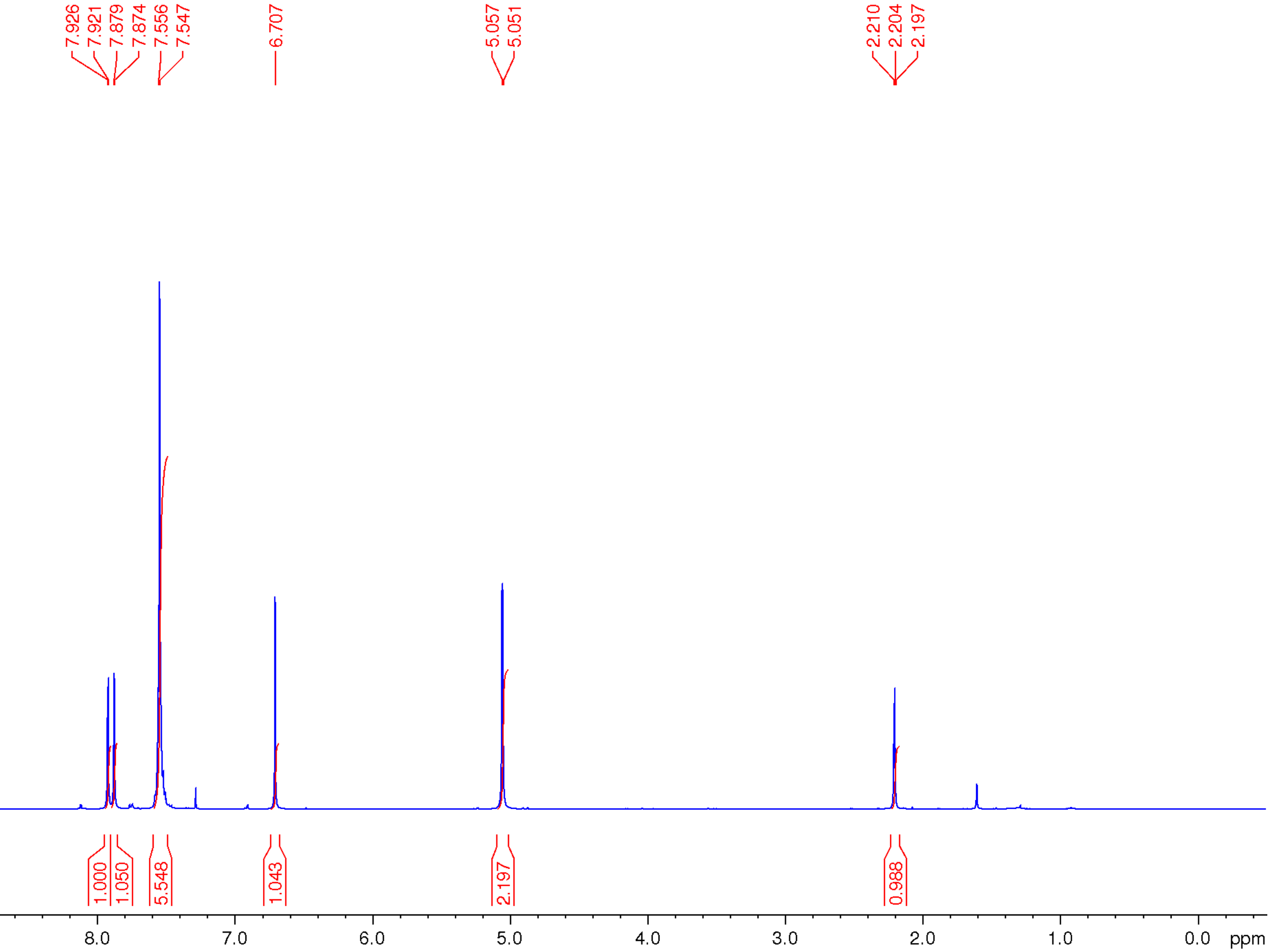

5-chloro-7-nitro-2-phenyl-1-(prop-2-yn-1-yl)-1*H*-indole 6a

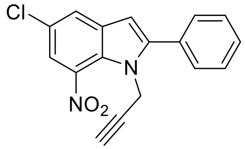

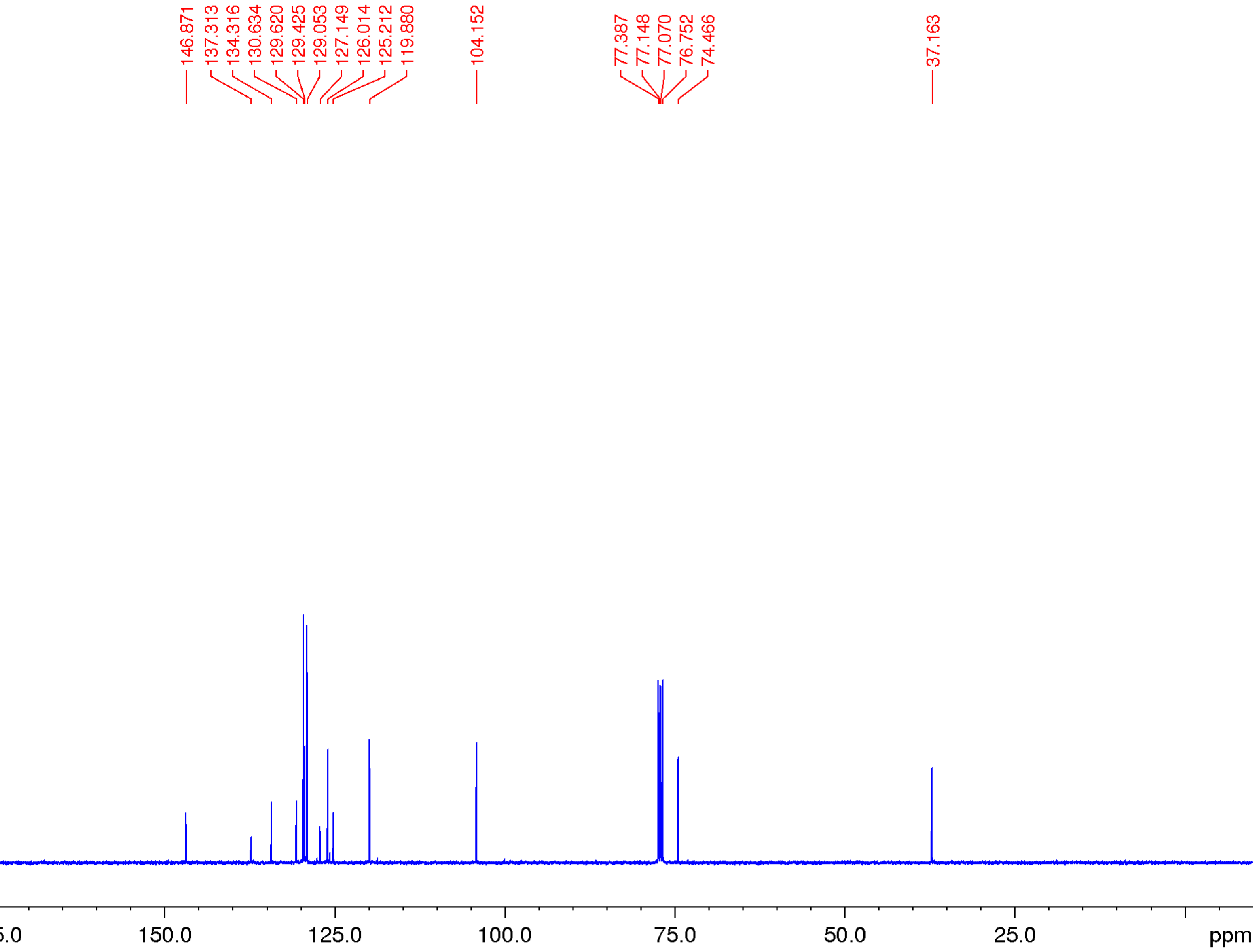

5-chloro-7-nitro-2-phenyl-1-(prop-2-yn-1-yl)-1*H*-indole 6a

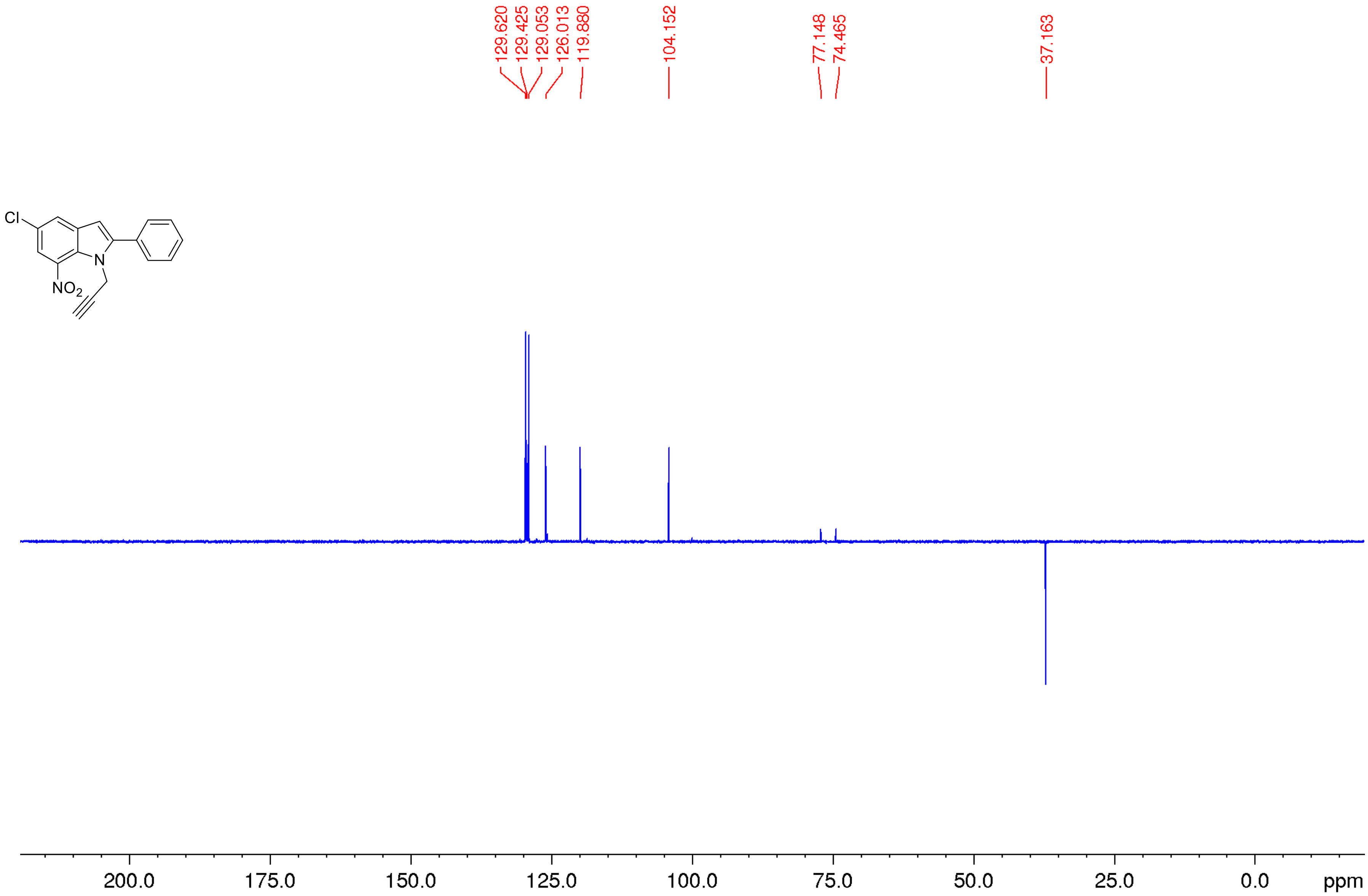

5-chloro-2-(4-methoxyphenyl)-7-nitro-1-(prop-2-yn-1-yl)-1*H*-indole 6b

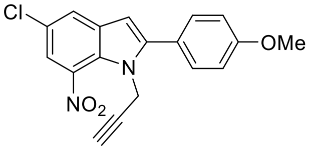

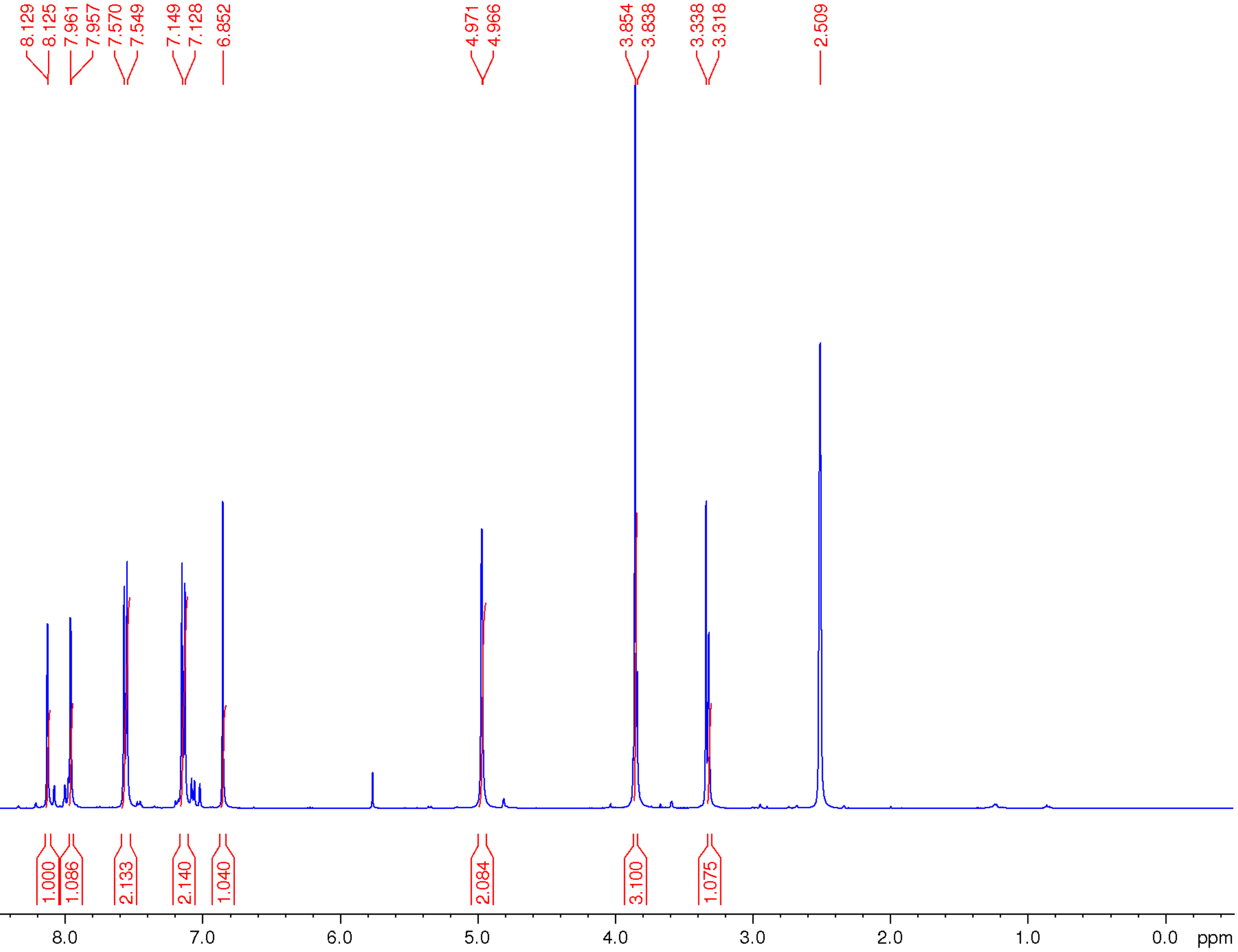

5-chloro-2-(4-methoxyphenyl)-7-nitro-1-(prop-2-yn-1-yl)-1*H*-indole 6b

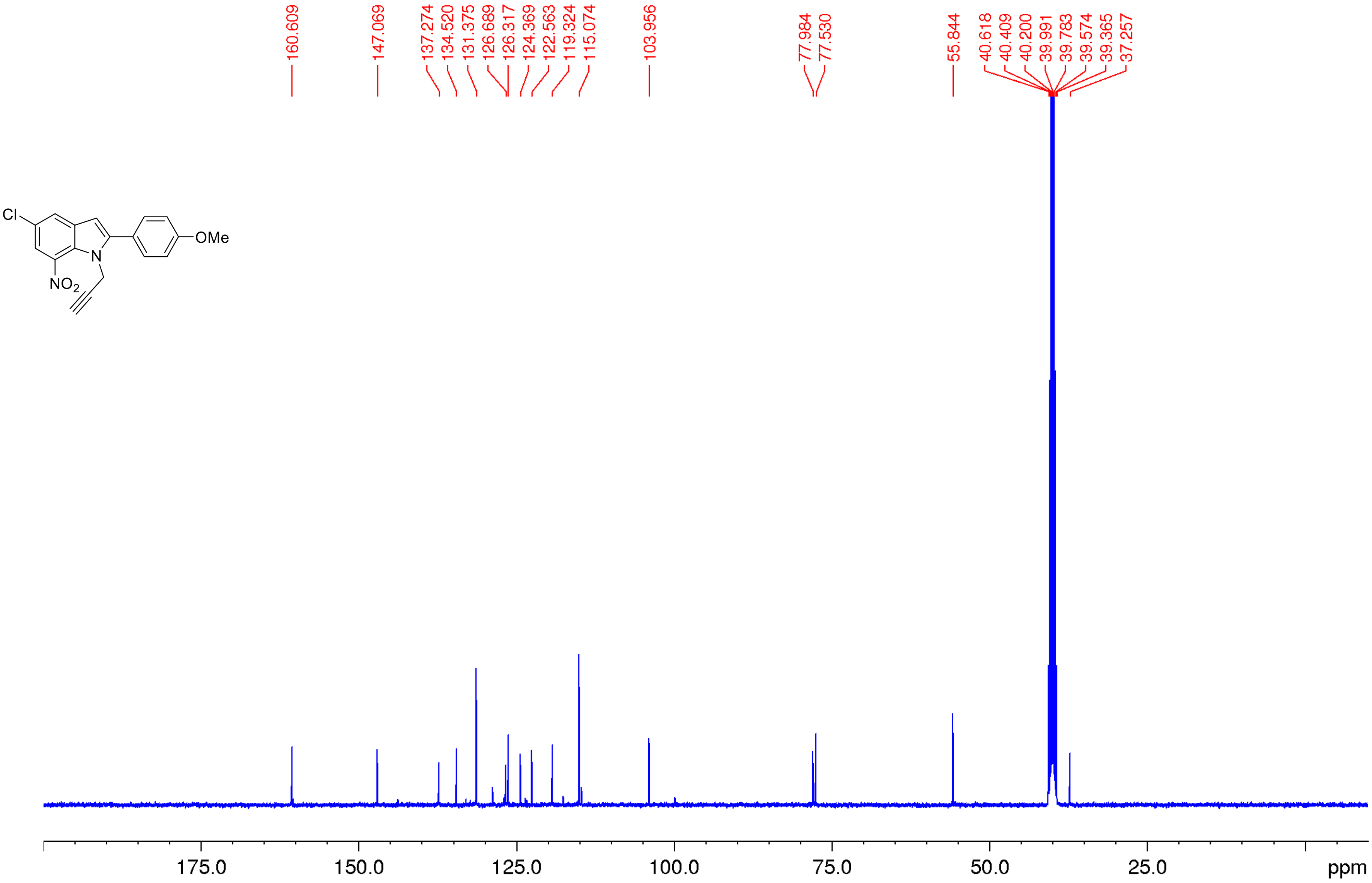

5-chloro-2-(4-methoxyphenyl)-7-nitro-1-(prop-2-yn-1-yl)-1*H*-indole 6b

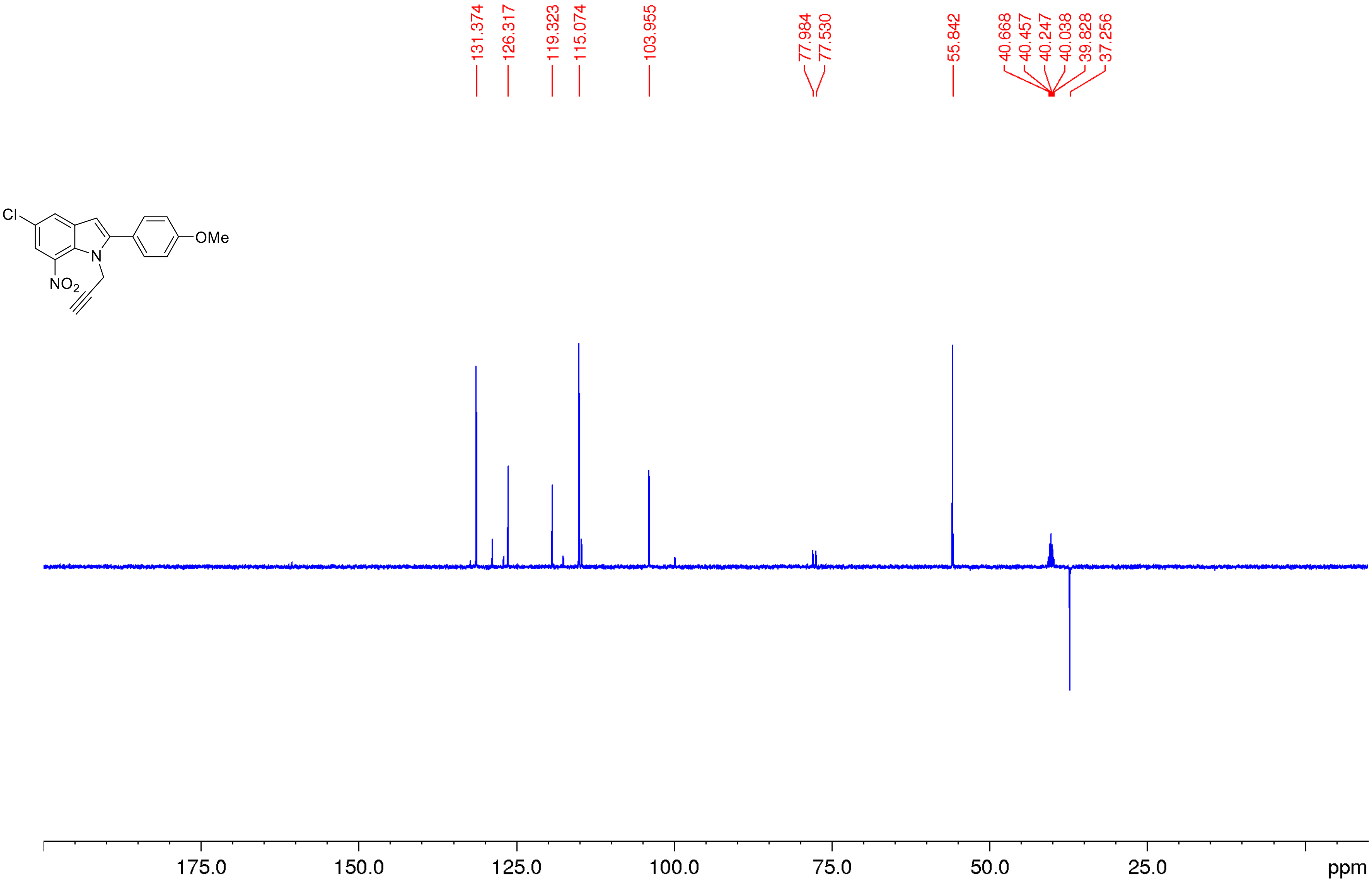

5-methyl-7-nitro-2-phenyl-1-(prop-2-yn-1-yl)-1*H*-indole 6c

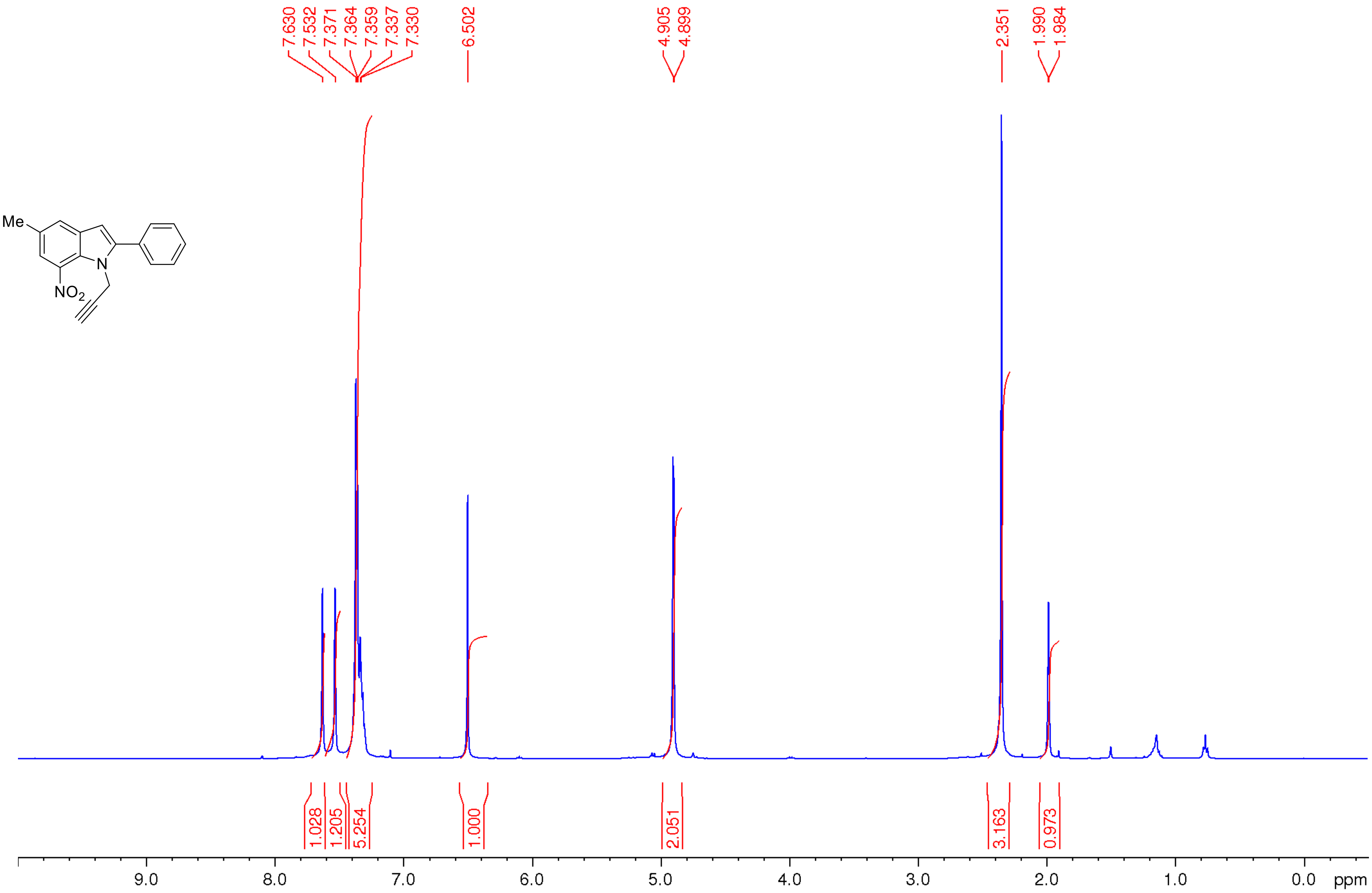

5-methyl-7-nitro-2-phenyl-1-(prop-2-yn-1-yl)-1*H*-indole 6c

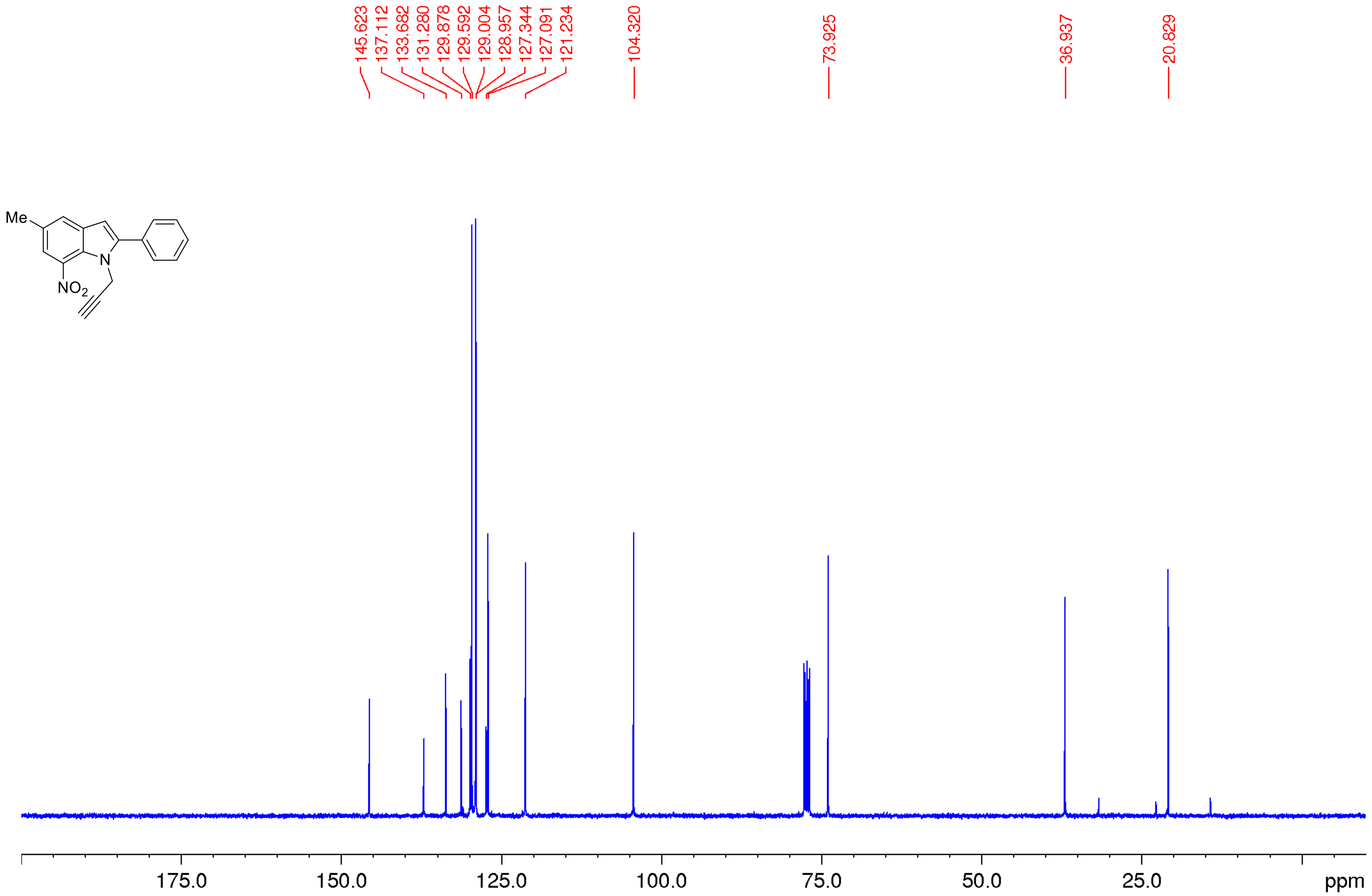

5-methyl-7-nitro-2-phenyl-1-(prop-2-yn-1-yl)-1*H*-indole 6c

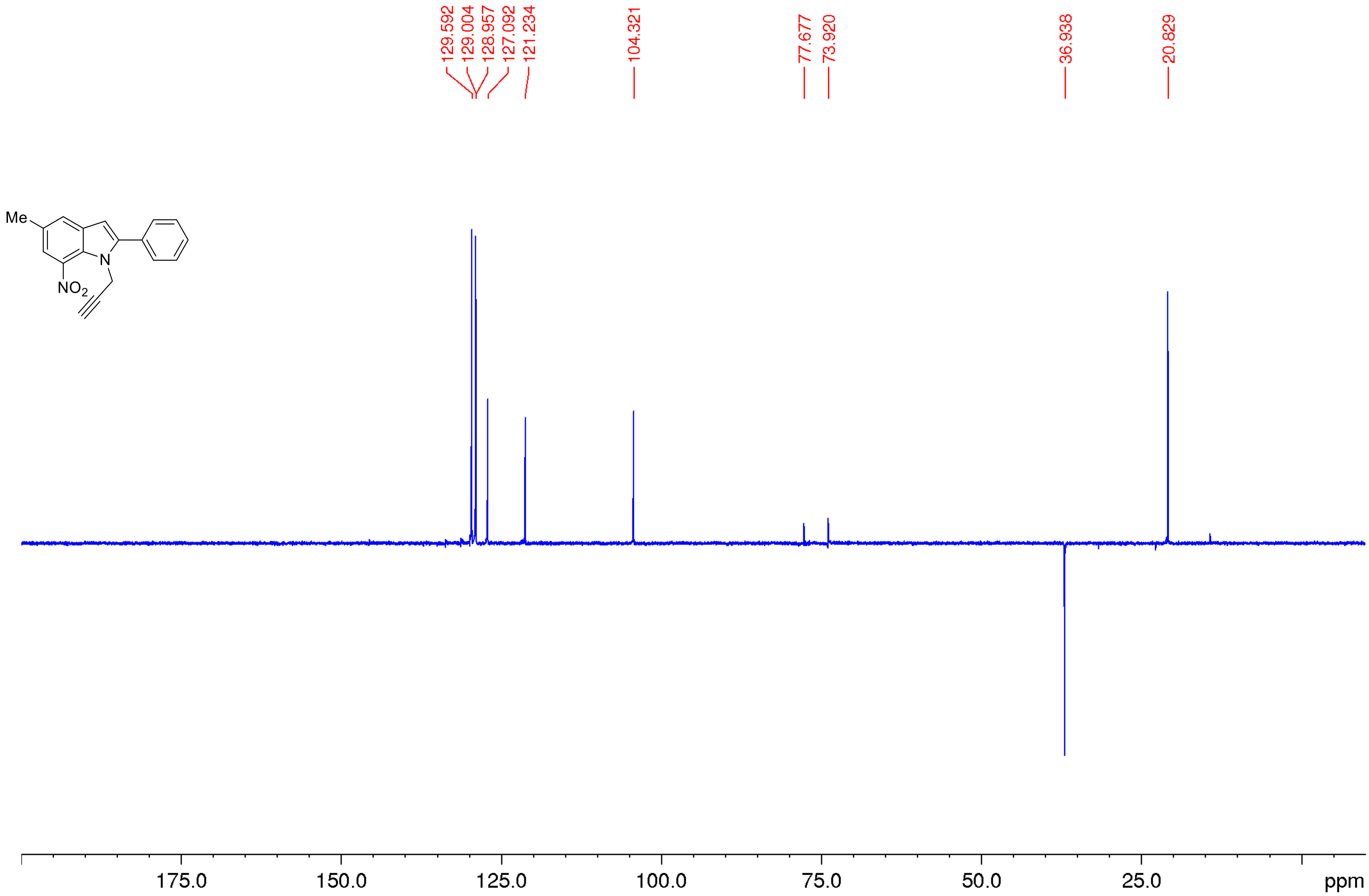

methyl 4-(5-methyl-7-nitro-1-(prop-2-yn-1-yl)-1*H*-indol-2-yl)benzoate 6d

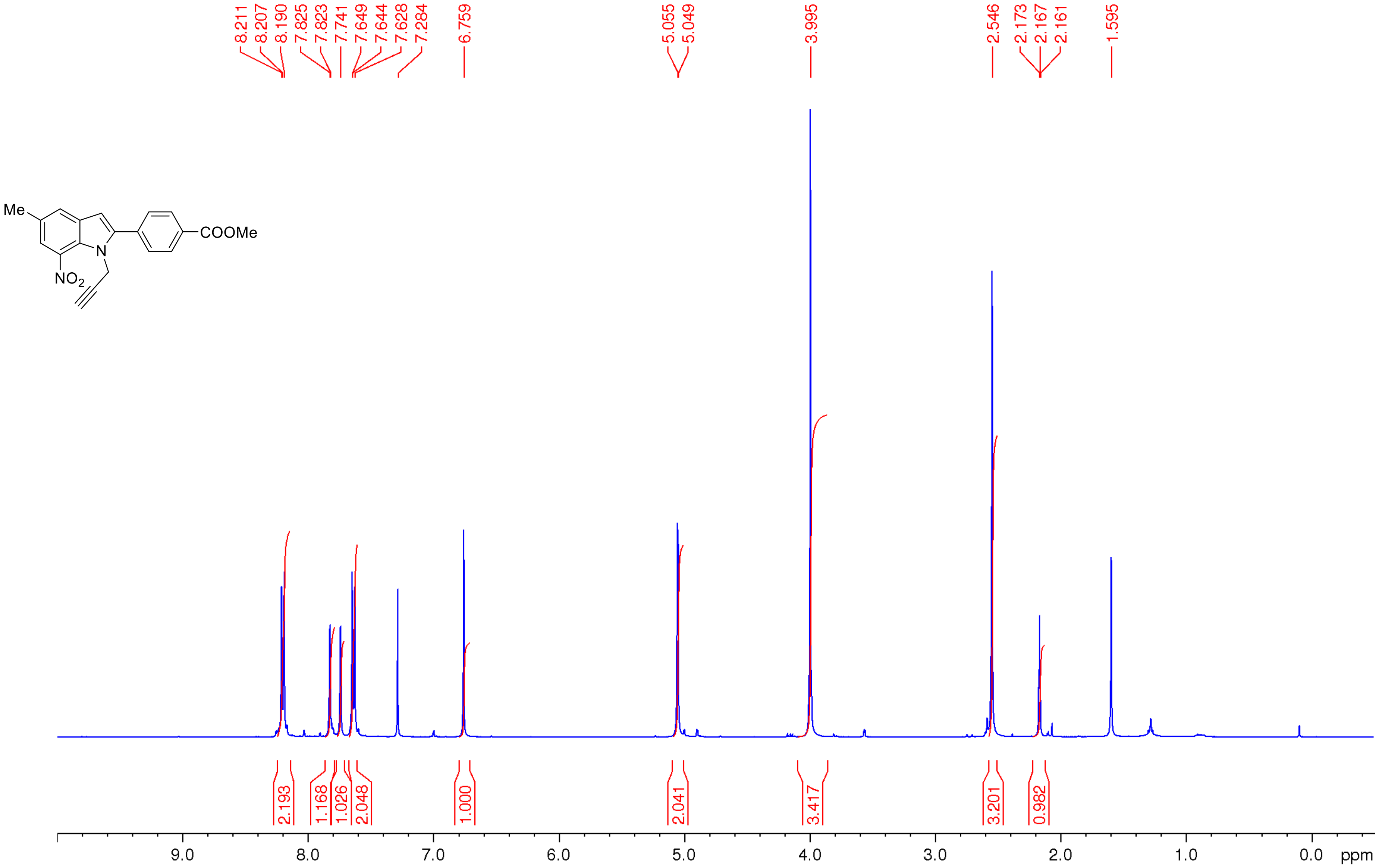

methyl 4-(5-methyl-7-nitro-1-(prop-2-yn-1-yl)-1*H*-indol-2-yl)benzoate 6d

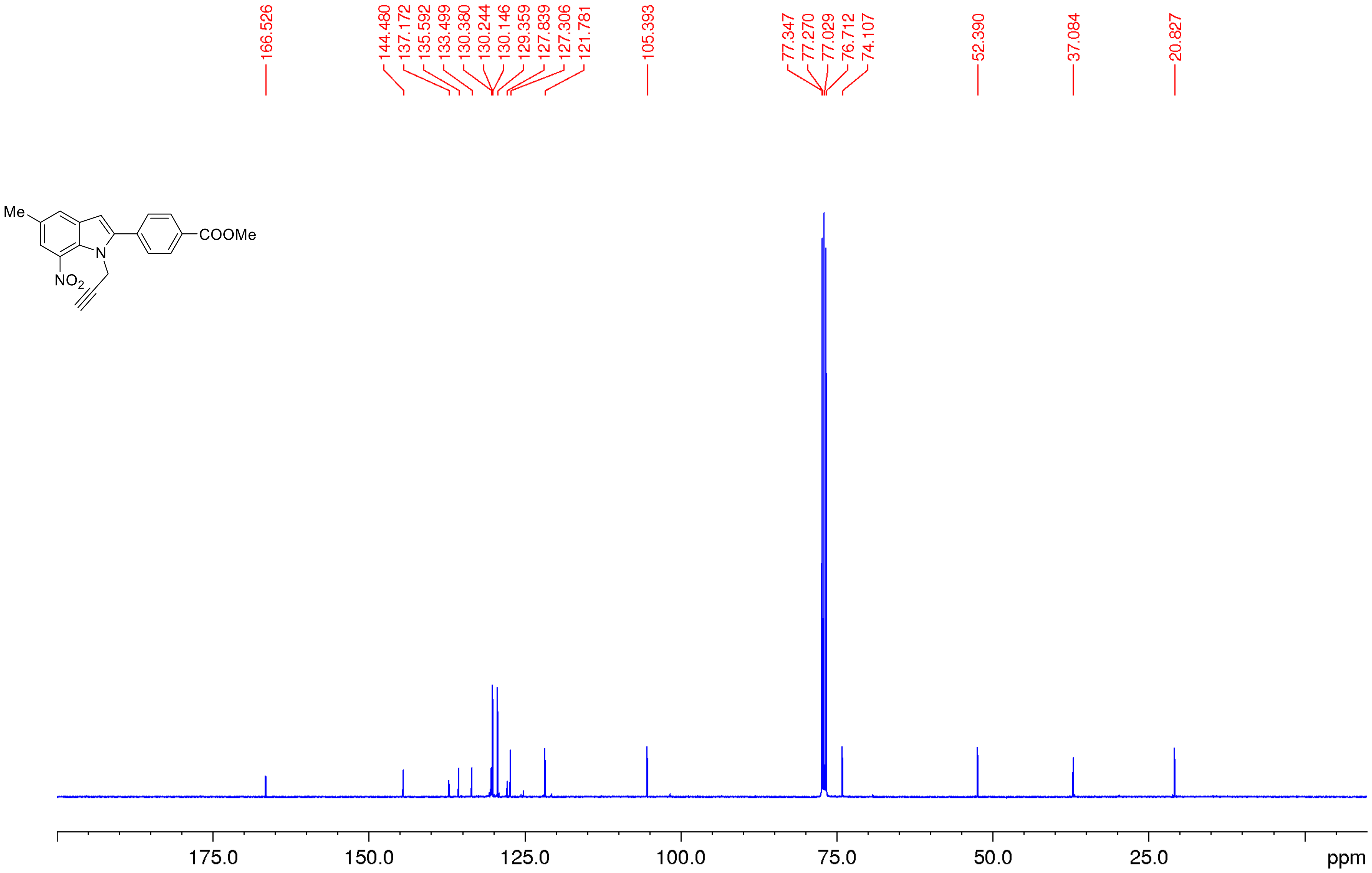

methyl 4-(5-methyl-7-nitro-1-(prop-2-yn-1-yl)-1*H*-indol-2-yl)benzoate 6d

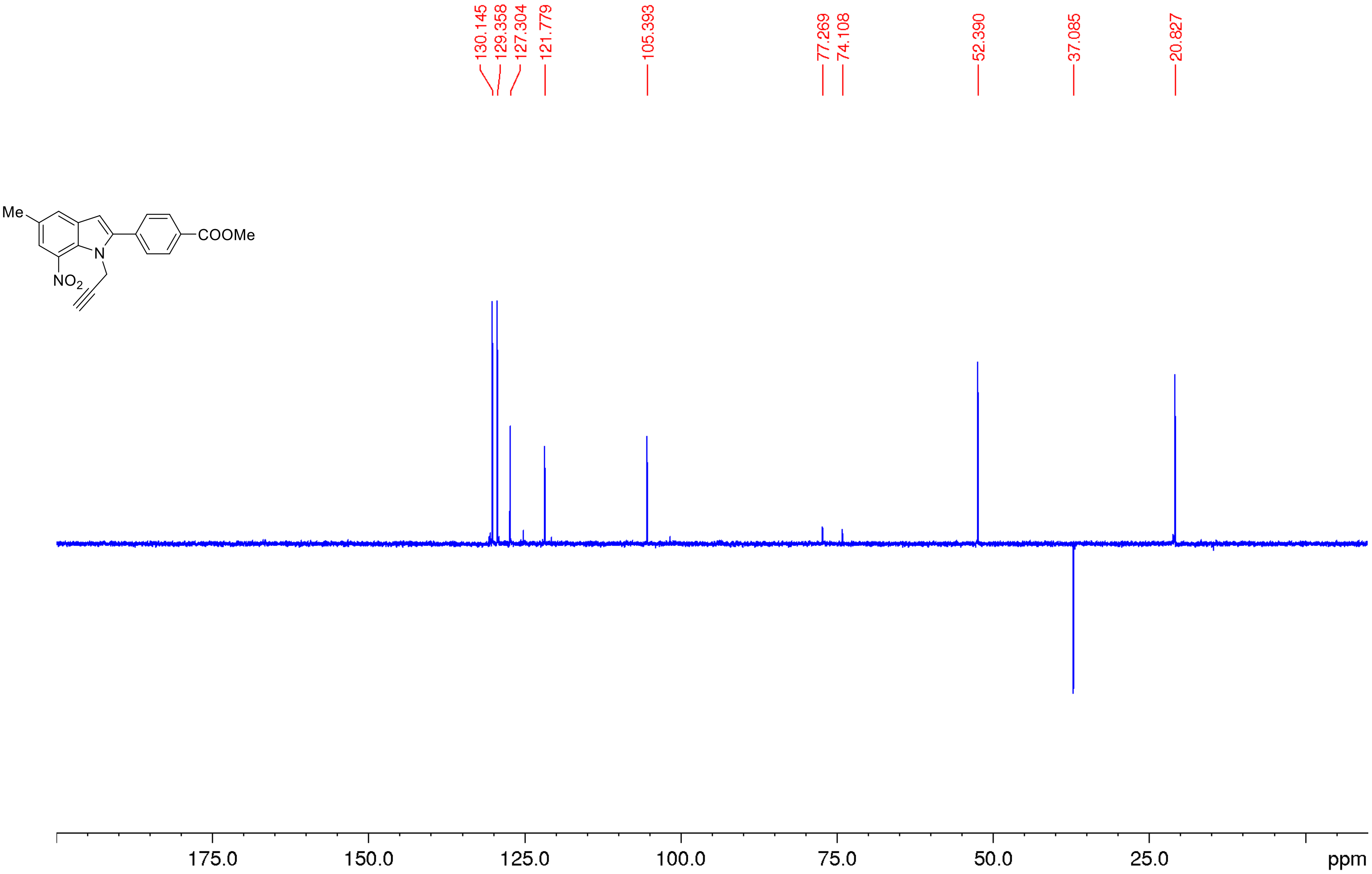

5-chloro-7-nitro-2-phenyl-1-(3-phenylprop-2-yn-1-yl)-1*H*-indole 7a

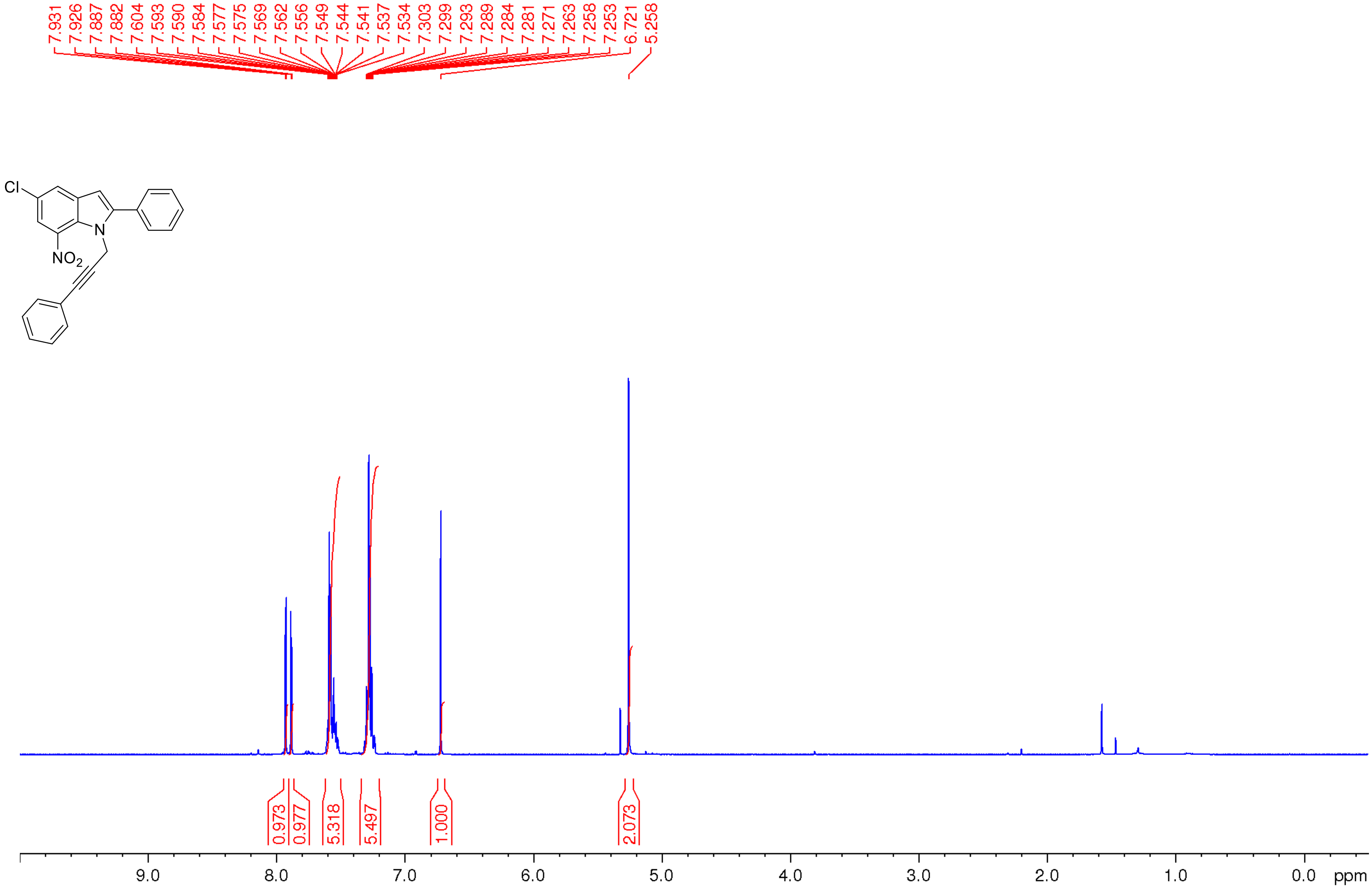

5-chloro-7-nitro-2-phenyl-1-(3-phenylprop-2-yn-1-yl)-1*H*-indole 7a

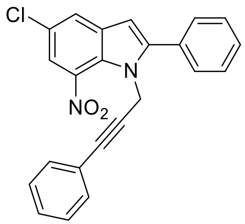

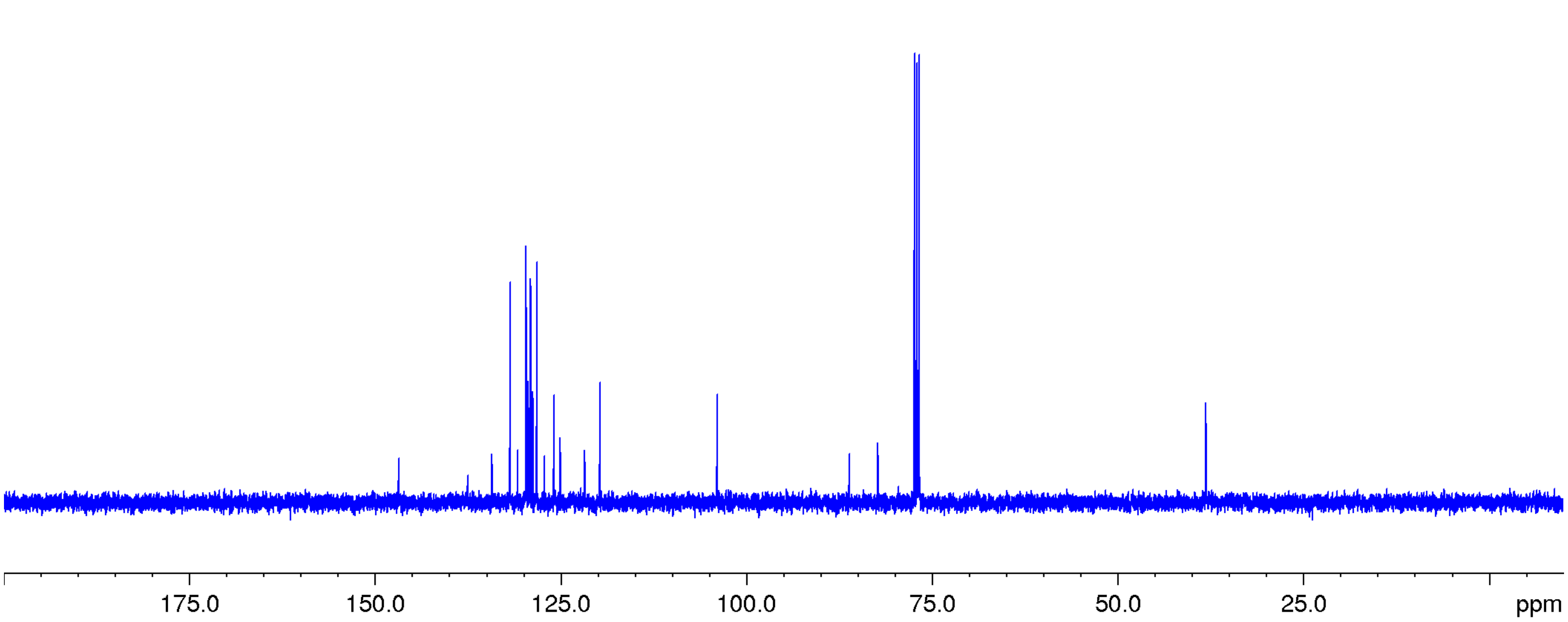

5-chloro-7-nitro-2-phenyl-1-(3-phenylprop-2-yn-1-yl)-1*H*-indole 7a

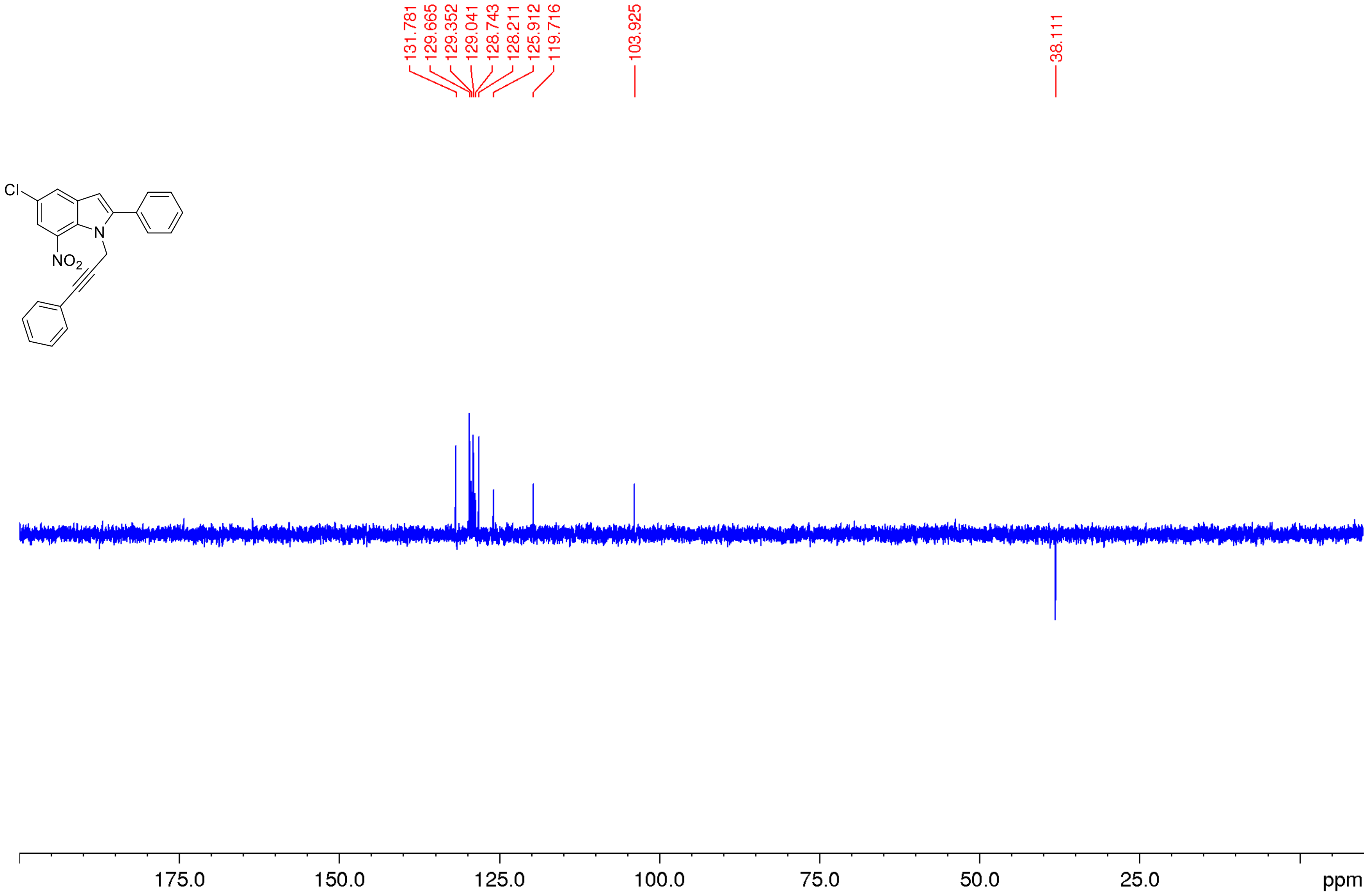

5-chloro-1-(3-(4-chlorophenyl)prop-2-yn-1-yl)-7-nitro-2-phenyl-1*H*-indole 7b

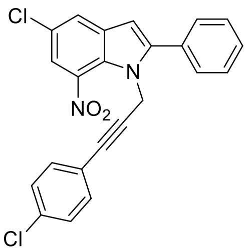

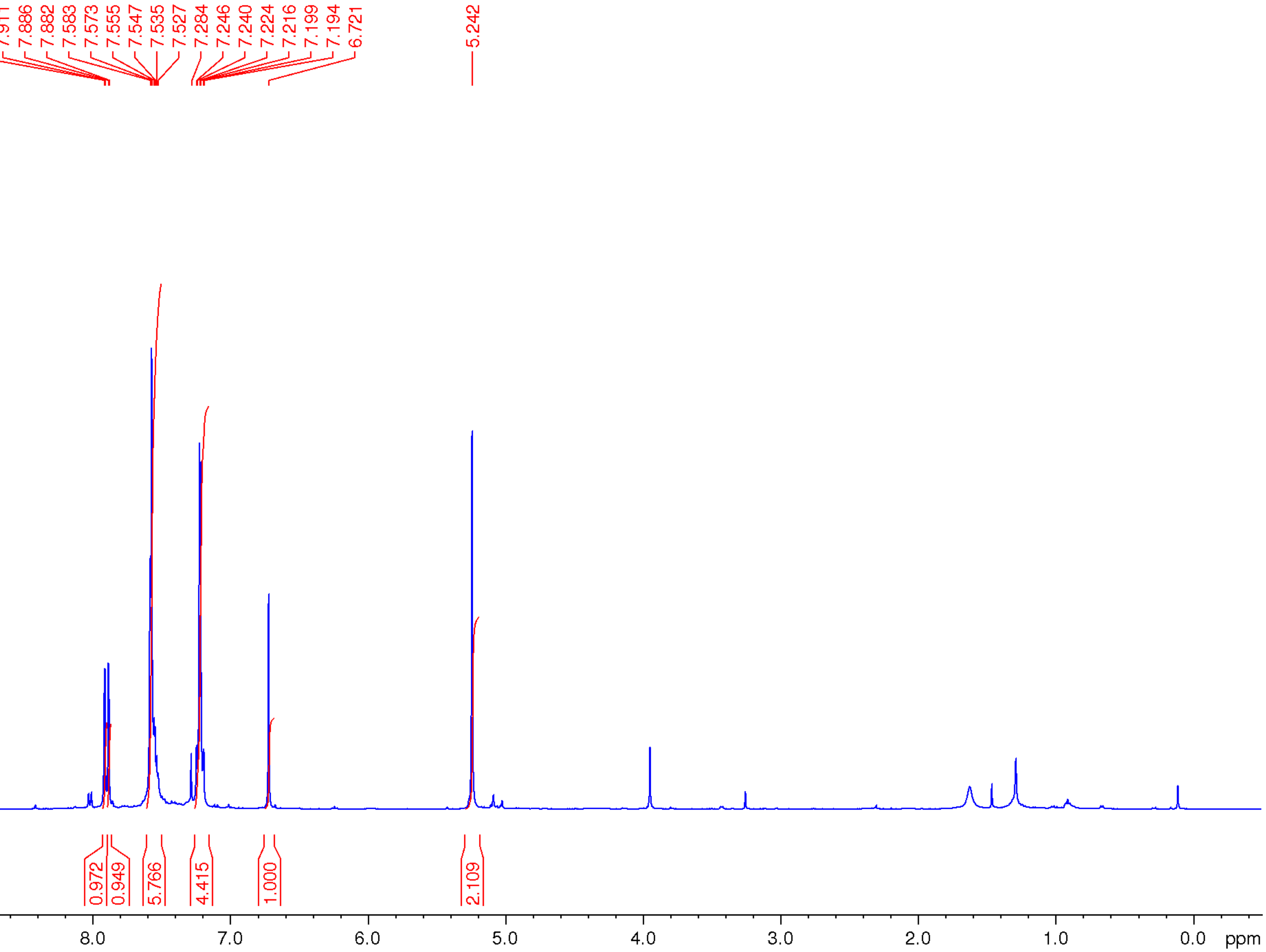

5-chloro-1-(3-(4-chlorophenyl)prop-2-yn-1-yl)-7-nitro-2-phenyl-1*H*-indole 7b

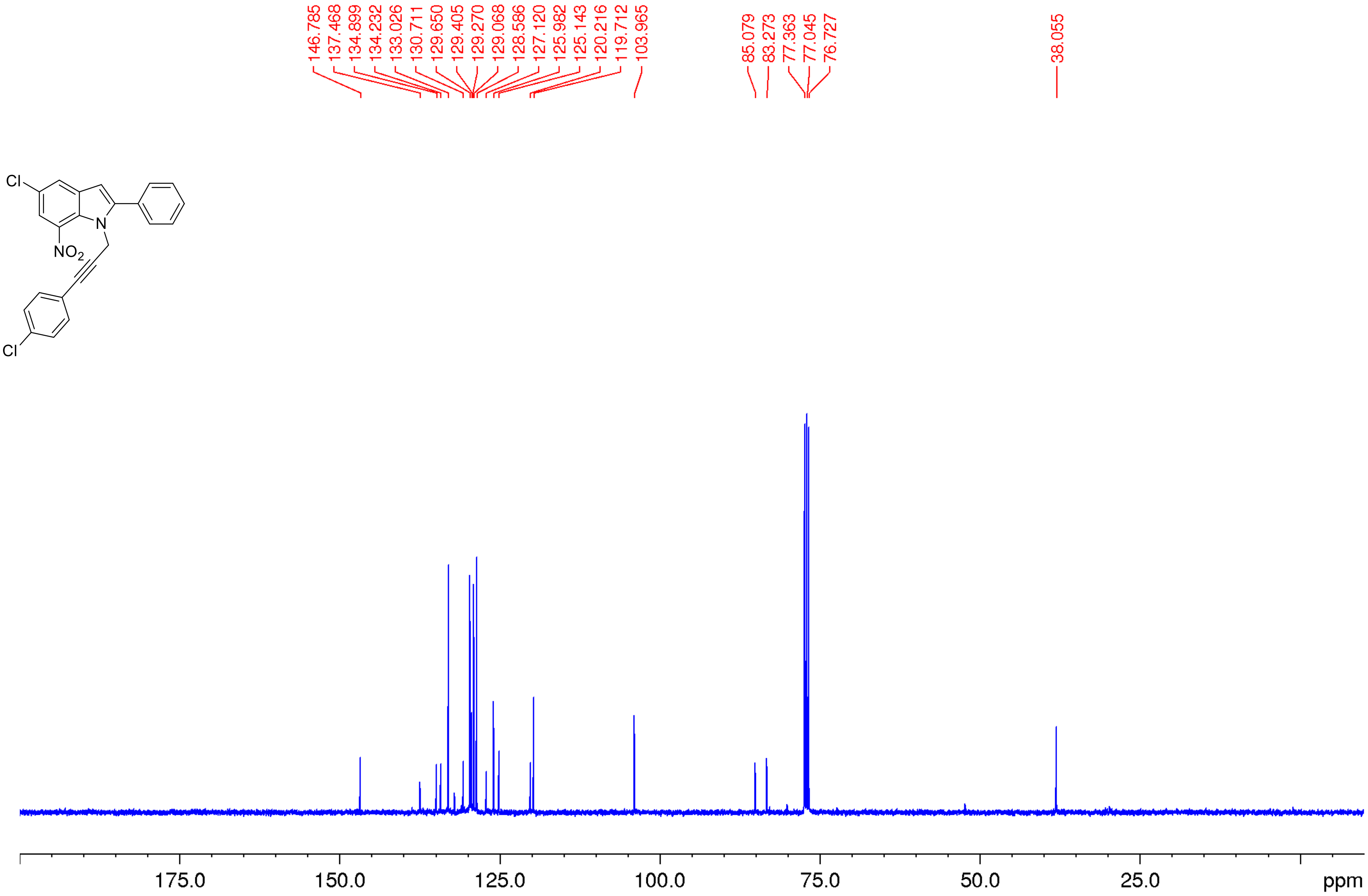

5-chloro-1-(3-(4-chlorophenyl)prop-2-yn-1-yl)-7-nitro-2-phenyl-1*H*-indole 7b

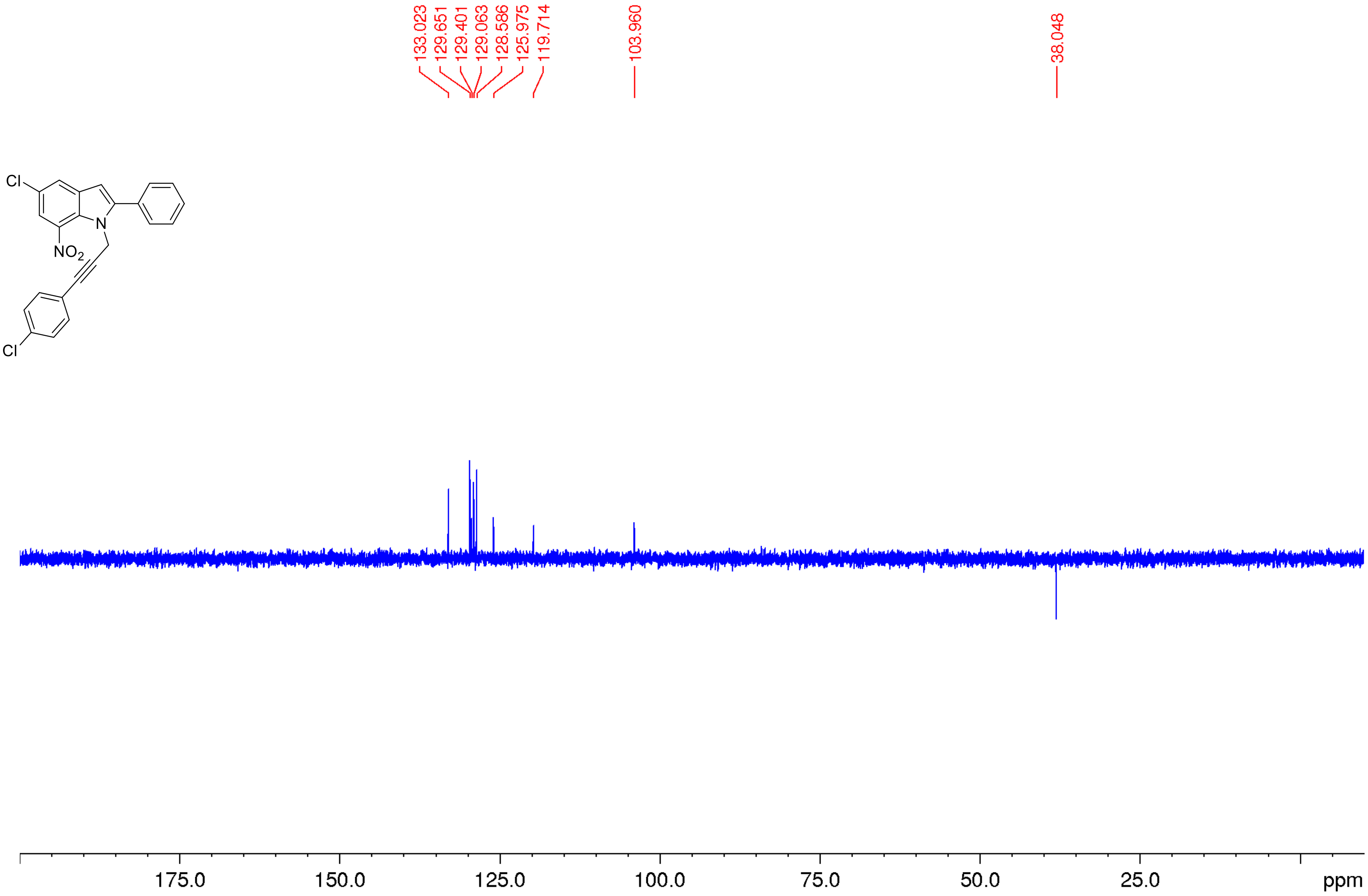

5-chloro-1-(3-(4-methoxyphenyl)prop-2-yn-1-yl)-7-nitro-2-phenyl-1*H*-indole 7c

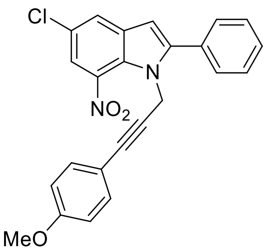

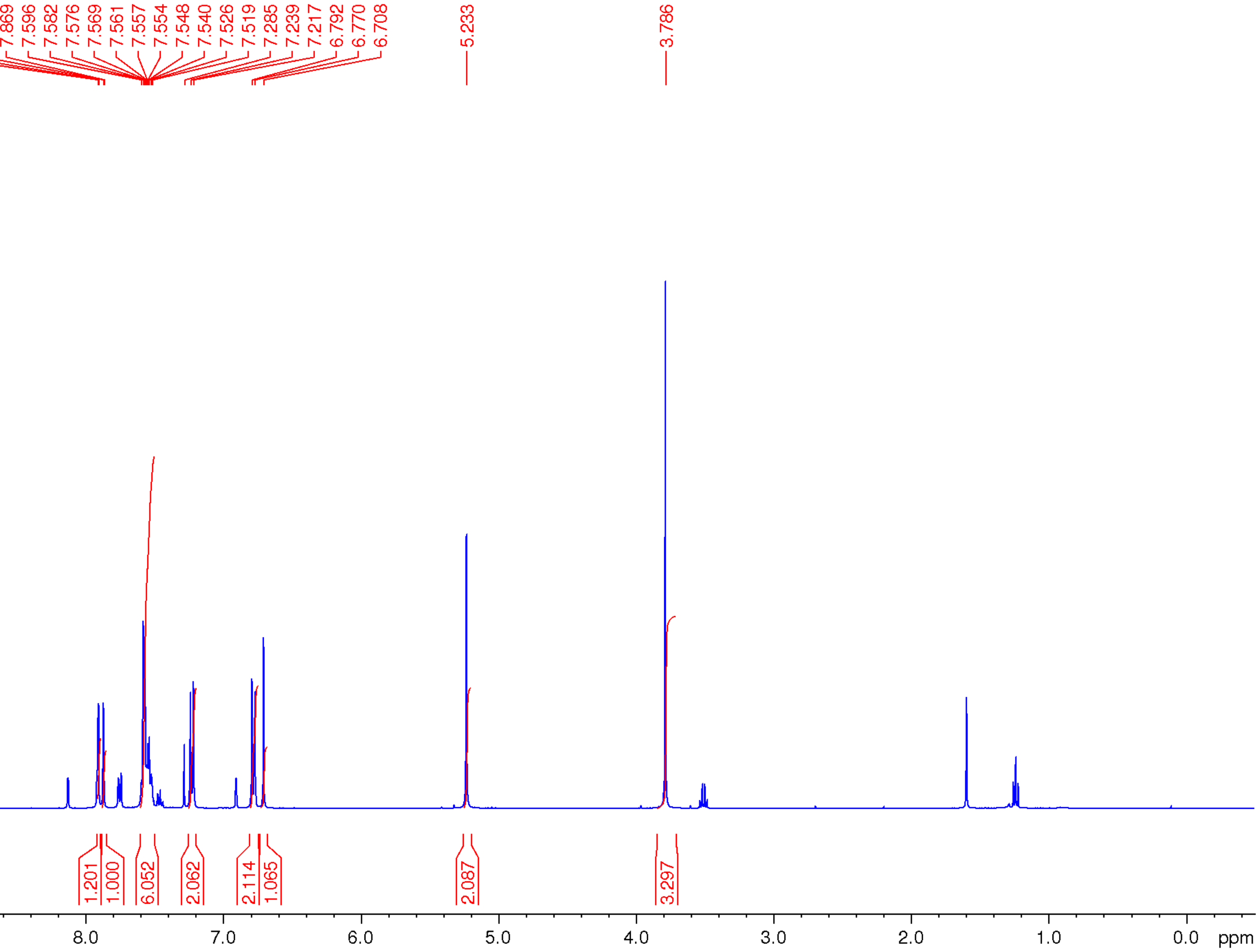

5-chloro-1-(3-(4-methoxyphenyl)prop-2-yn-1-yl)-7-nitro-2-phenyl-1*H*-indole 7c

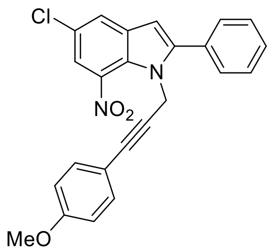

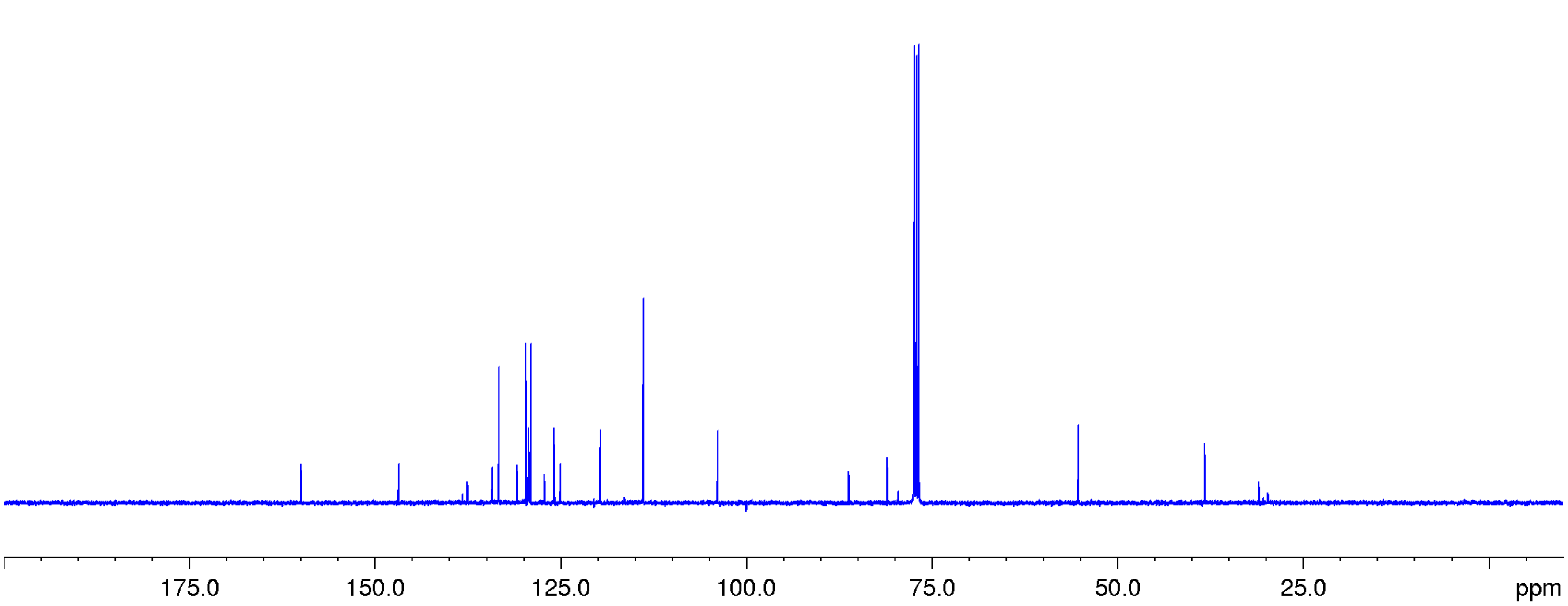

5-chloro-1-(3-(4-methoxyphenyl)prop-2-yn-1-yl)-7-nitro-2-phenyl-1*H*-indole 7c

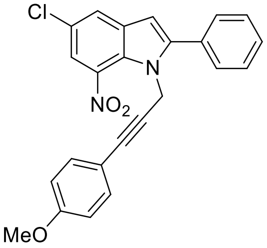

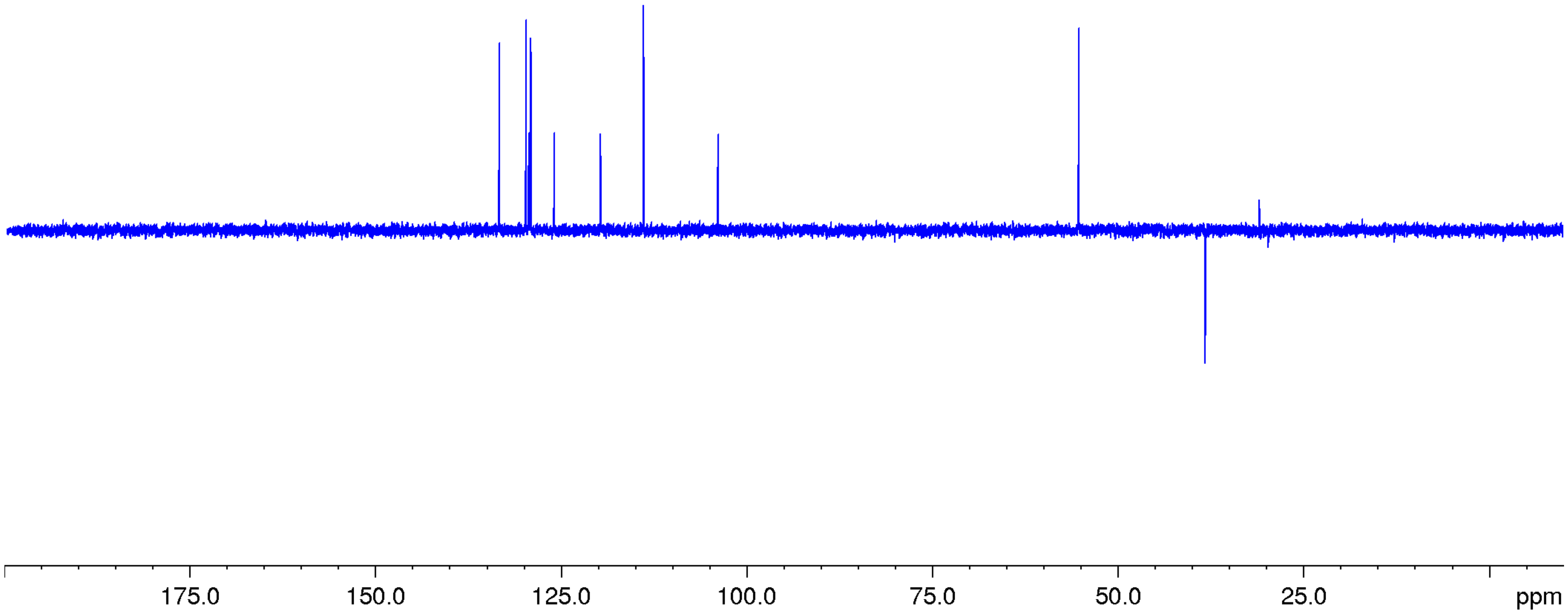

1-(4-(3-(5-chloro-7-nitro-2-phenyl-1*H*-indol-1-yl)prop-1-yn-1-yl)phenyl)ethan-1-one 7d

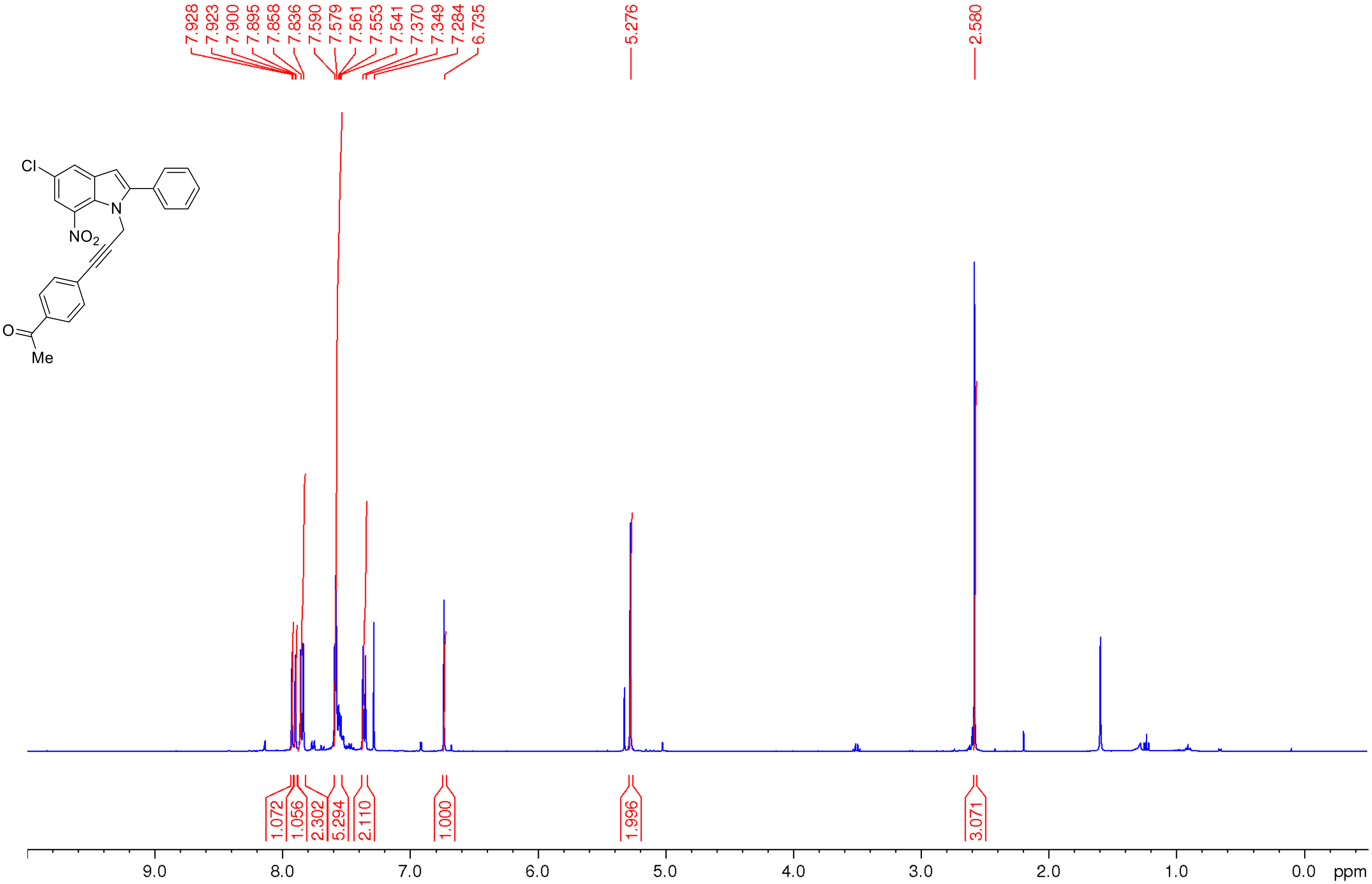

1-(4-(3-(5-chloro-7-nitro-2-phenyl-1*H*-indol-1-yl)prop-1-yn-1-yl)phenyl)ethan-1-one 7d

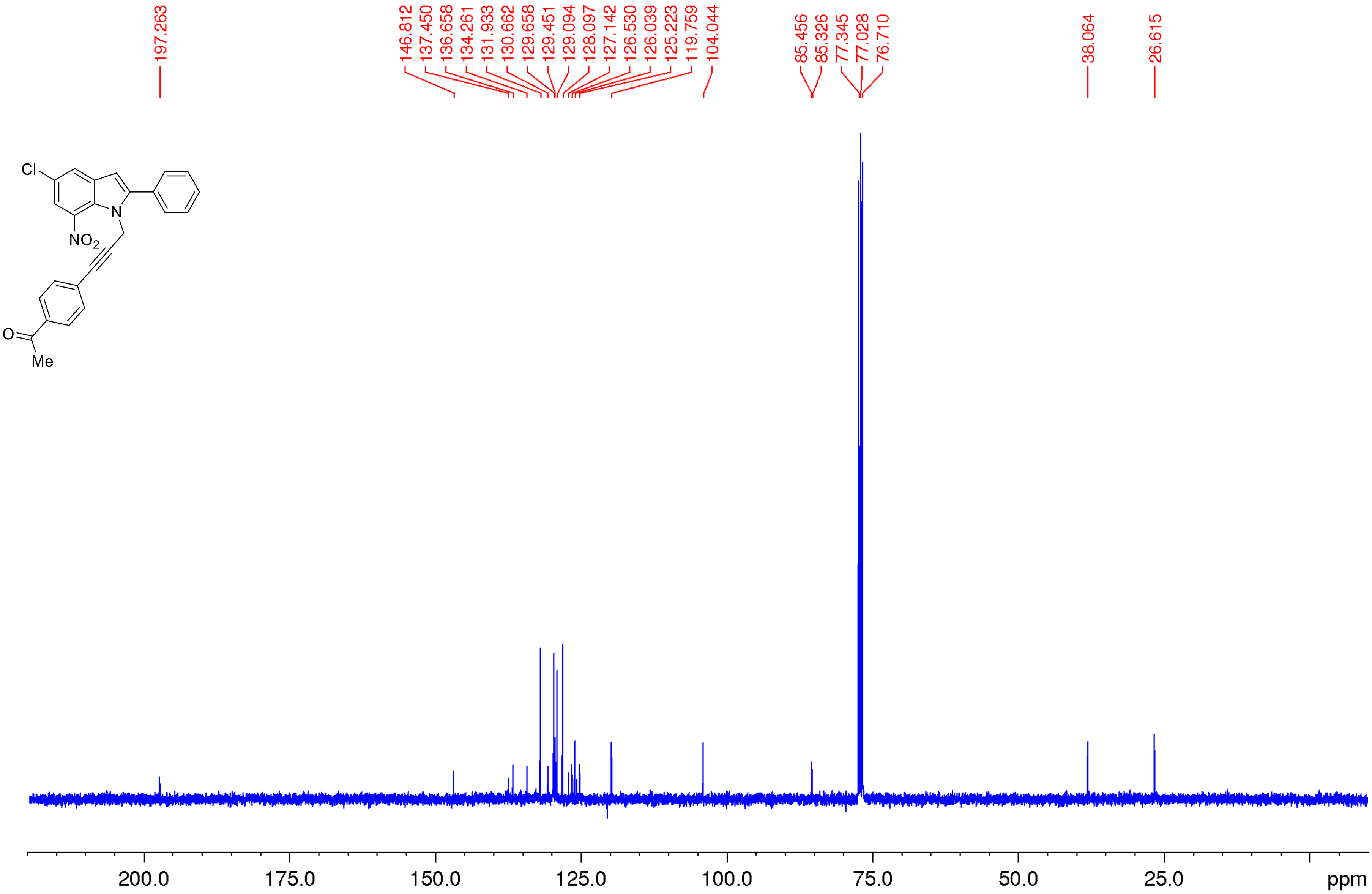

1-(4-(3-(5-chloro-7-nitro-2-phenyl-1*H*-indol-1-yl)prop-1-yn-1-yl)phenyl)ethan-1-one 7d

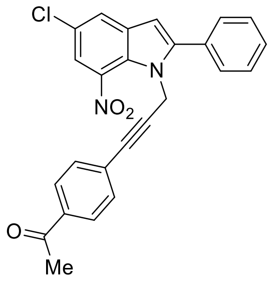

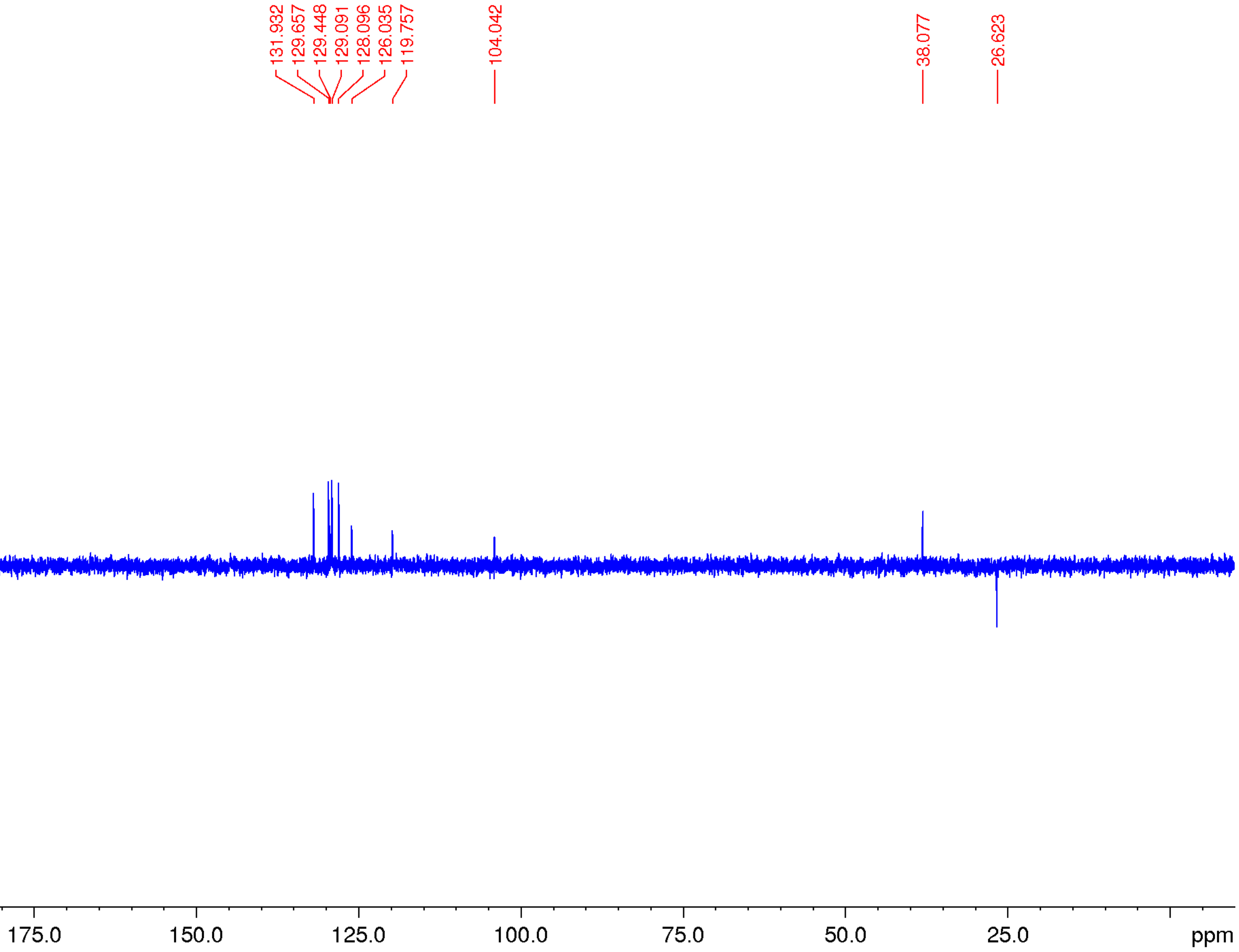

5-chloro-2-(4-methoxyphenyl)-7-nitro-1-(3-(3-(trifluoromethyl)phenyl)prop-2-yn-1-yl)-1*H*-indole 7e

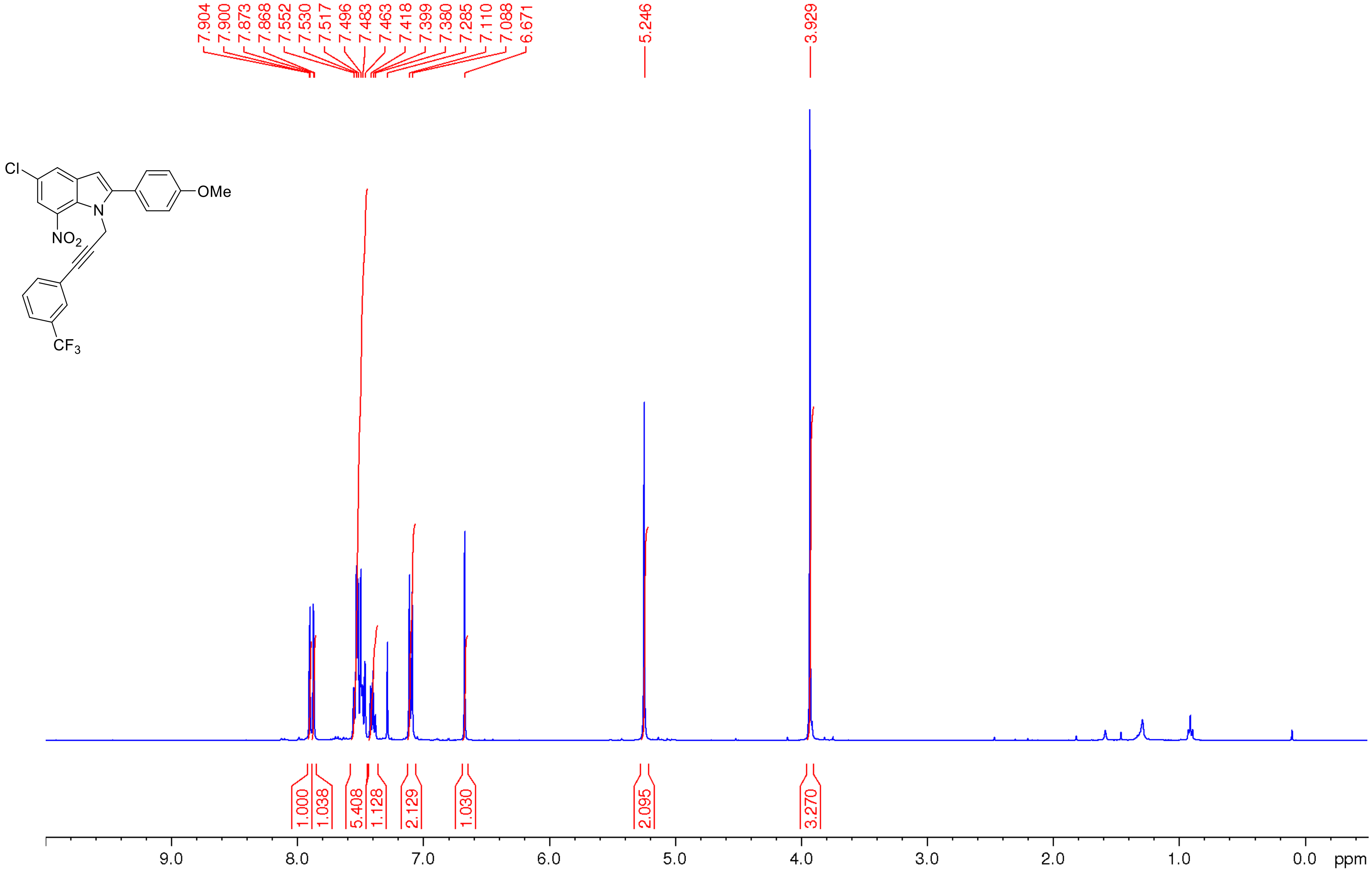

5-chloro-2-(4-methoxyphenyl)-7-nitro-1-(3-(3-(trifluoromethyl)phenyl)prop-2-yn-1-yl)-1*H*-indole 7e

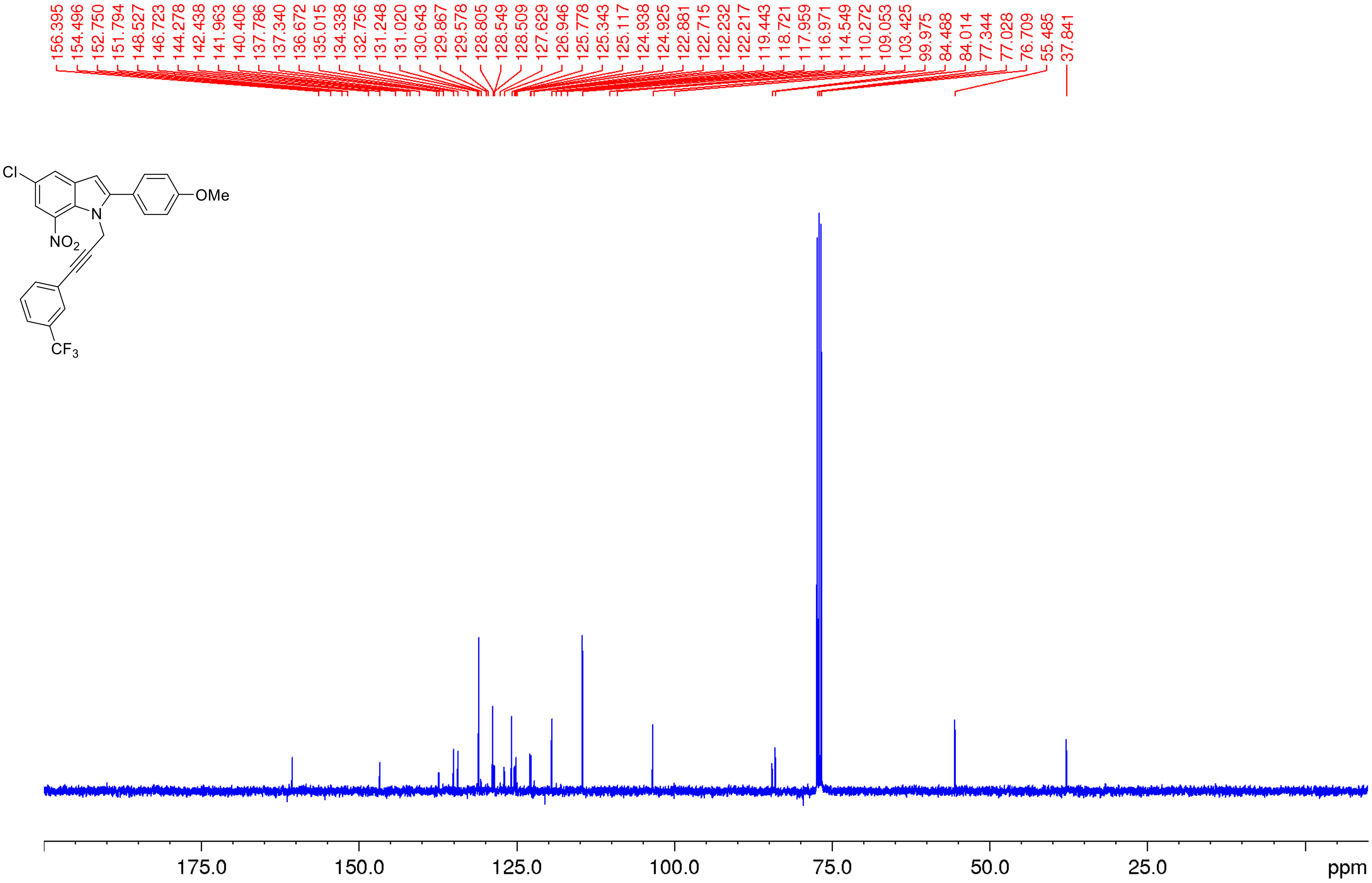

5-chloro-2-(4-methoxyphenyl)-7-nitro-1-(3-(3-(trifluoromethyl)phenyl)prop-2-yn-1-yl)-1*H*-indole 7e

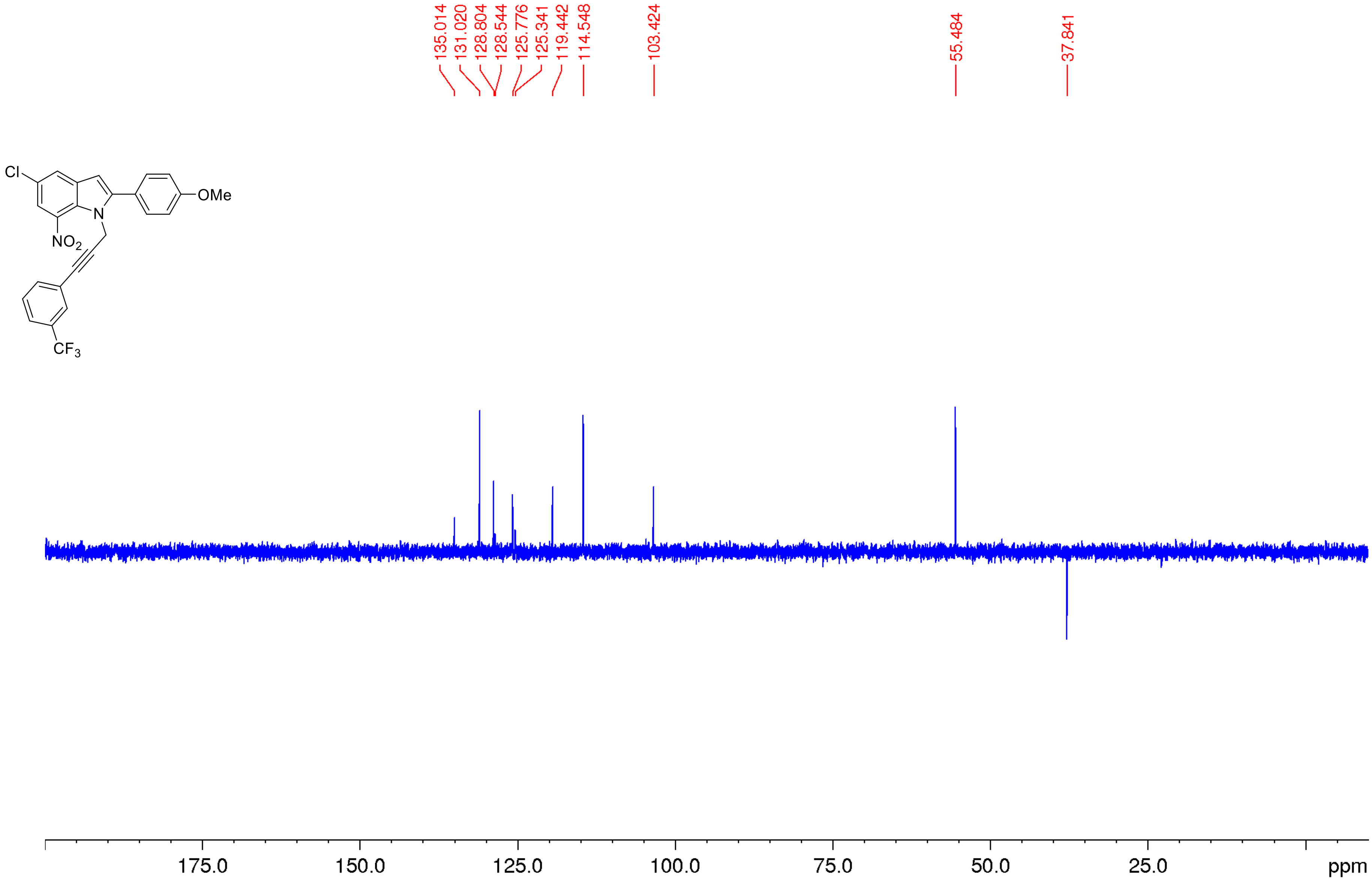

5-chloro-2-(4-methoxyphenyl)-7-nitro-1-(3-(3-(trifluoromethyl)phenyl)prop-2-yn-1-yl)-1*H*-indole 7e

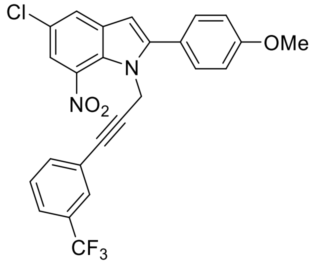

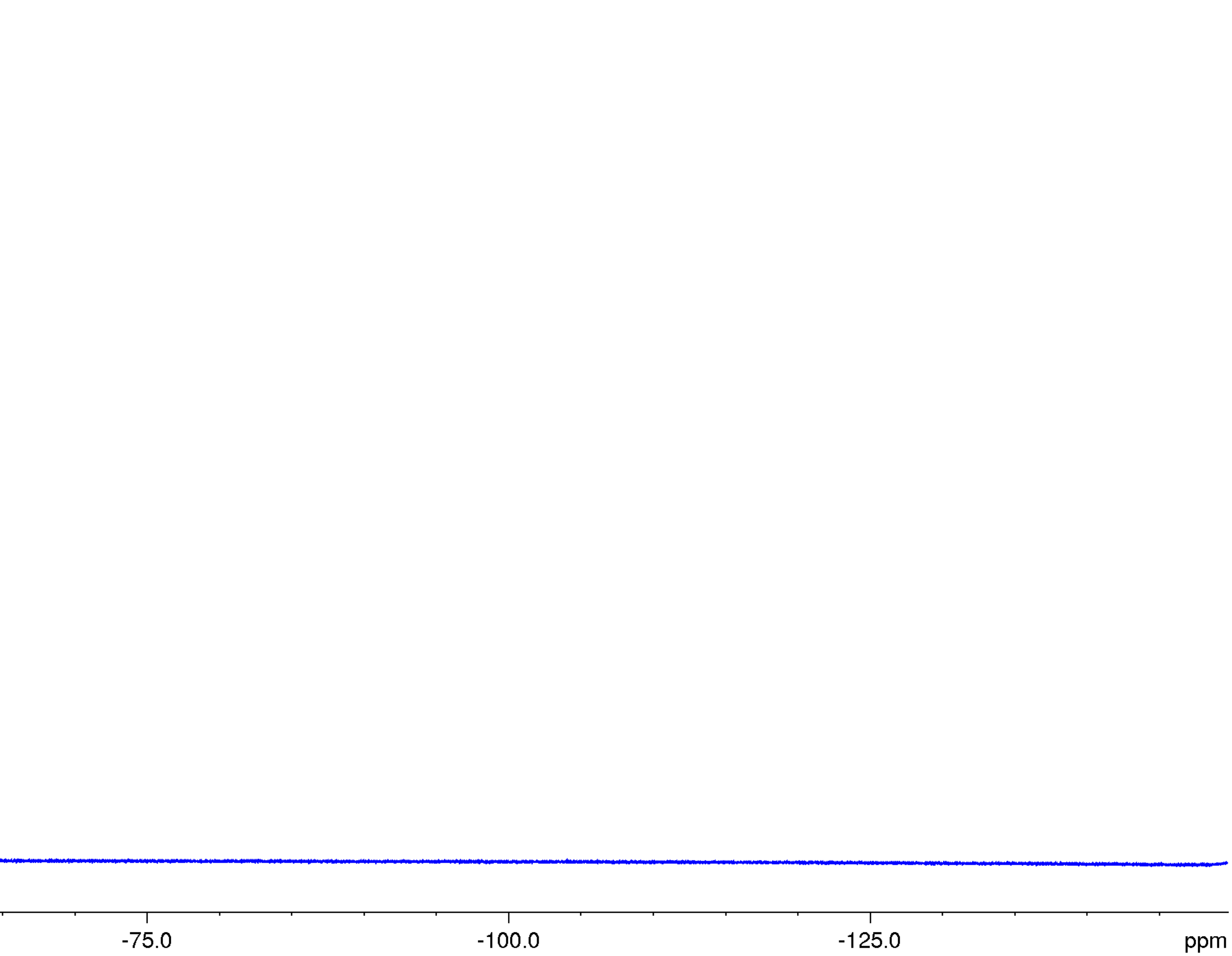

5-chloro-2-(4-methoxyphenyl)-1-(3-(4-methoxyphenyl)prop-2-yn-1-yl)-7-nitro-1*H*-indole 7f

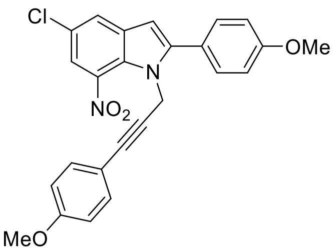

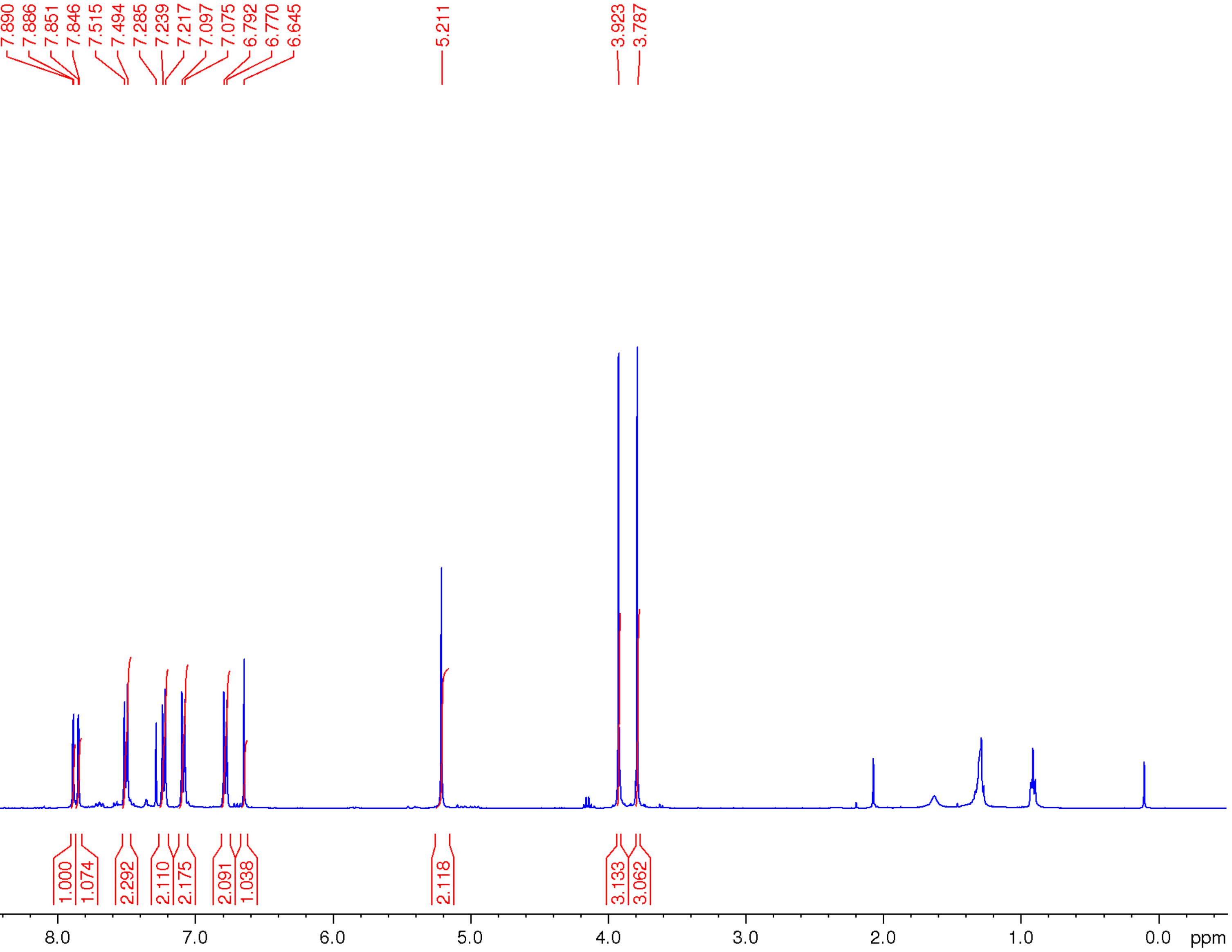

5-chloro-2-(4-methoxyphenyl)-1-(3-(4-methoxyphenyl)prop-2-yn-1-yl)-7-nitro-1*H*-indole 7f

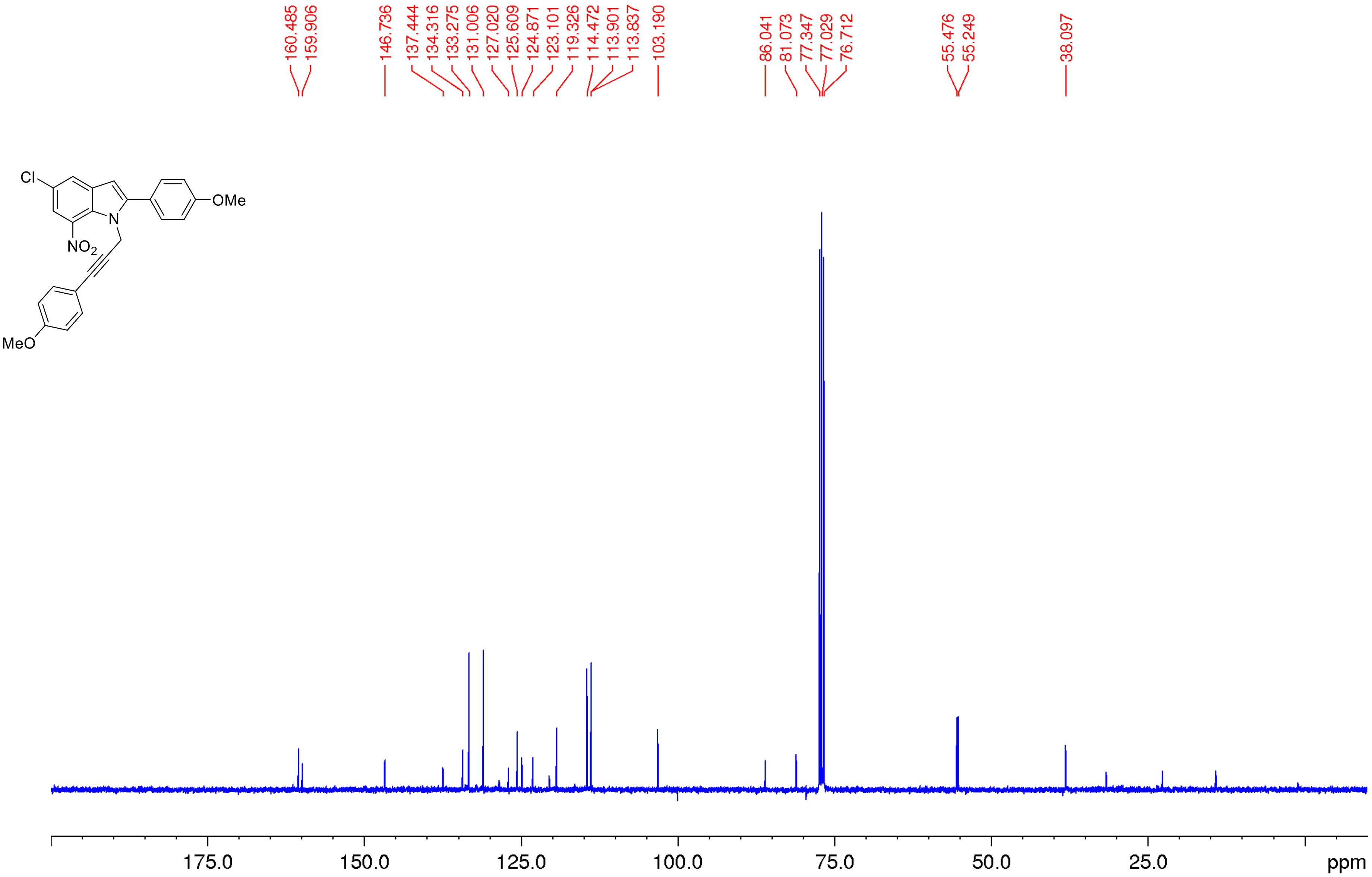

5-chloro-2-(4-methoxyphenyl)-1-(3-(4-methoxyphenyl)prop-2-yn-1-yl)-7-nitro-1*H*-indole 7f

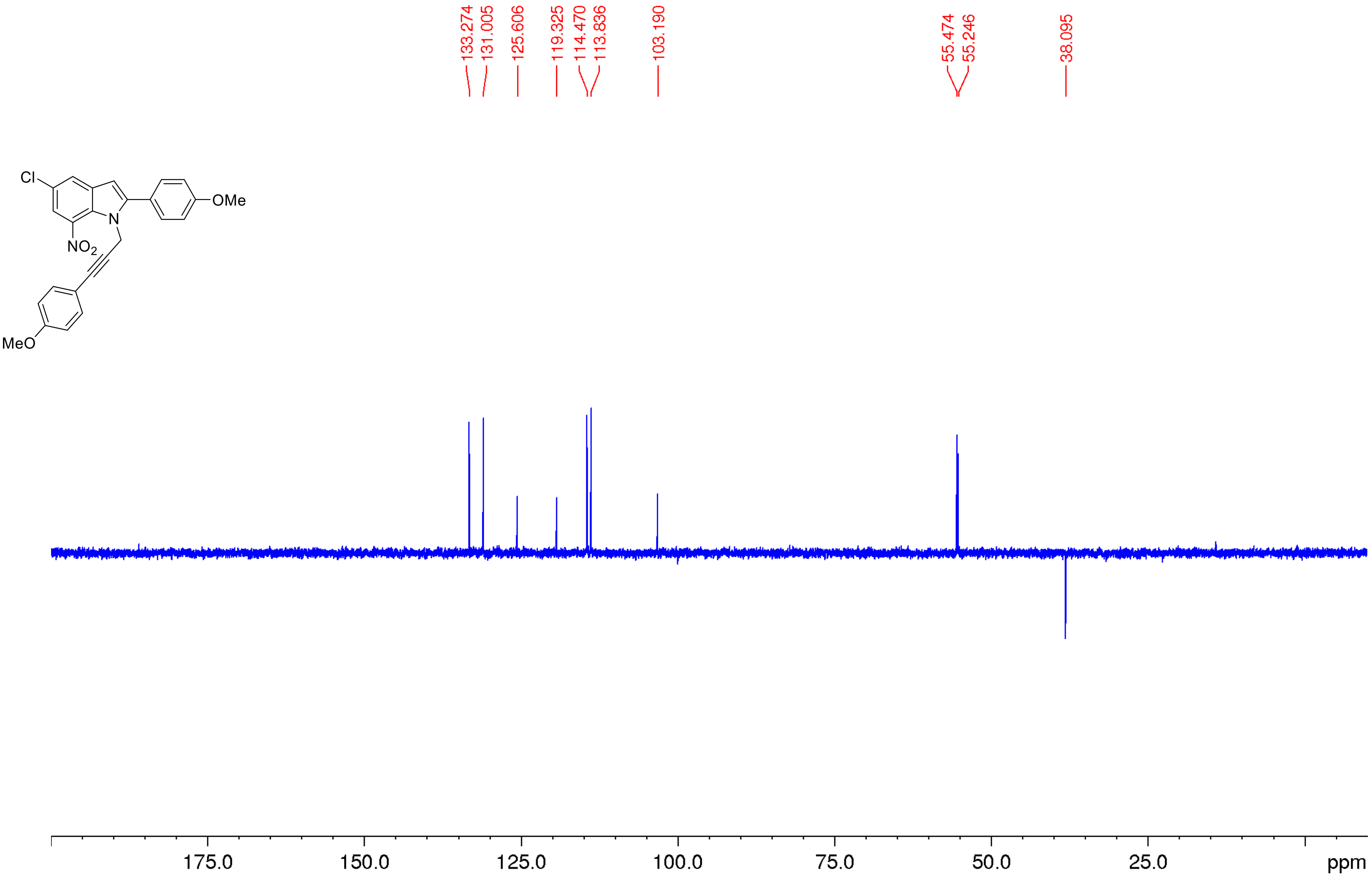

1-(4-(3-(5-methyl-7-nitro-2-phenyl-1*H*-indol-1-yl)prop-1-yn-1-yl)phenyl)ethan-1-one 7g

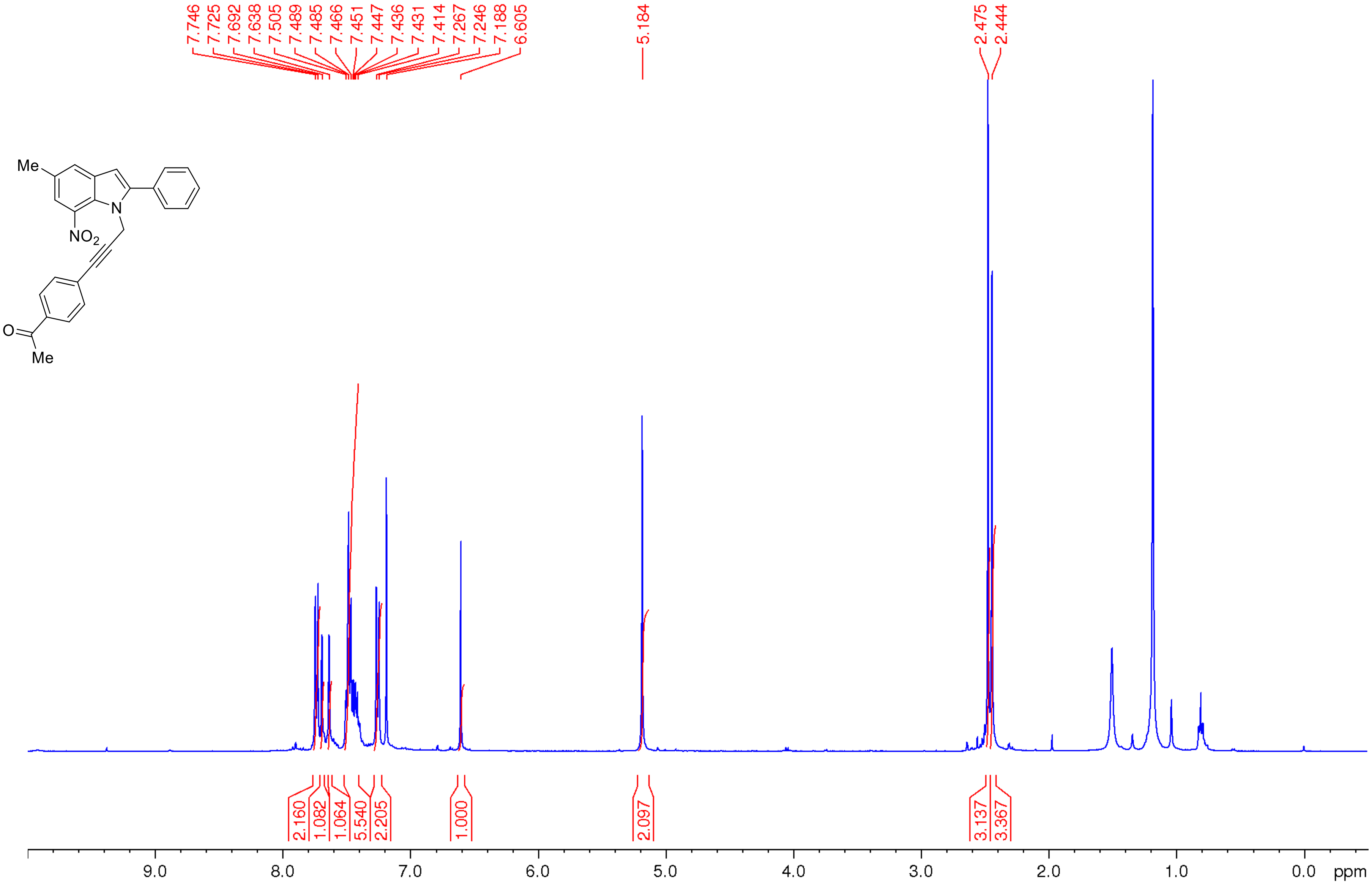

1-(4-(3-(5-methyl-7-nitro-2-phenyl-1*H*-indol-1-yl)prop-1-yn-1-yl)phenyl)ethan-1-one 7g

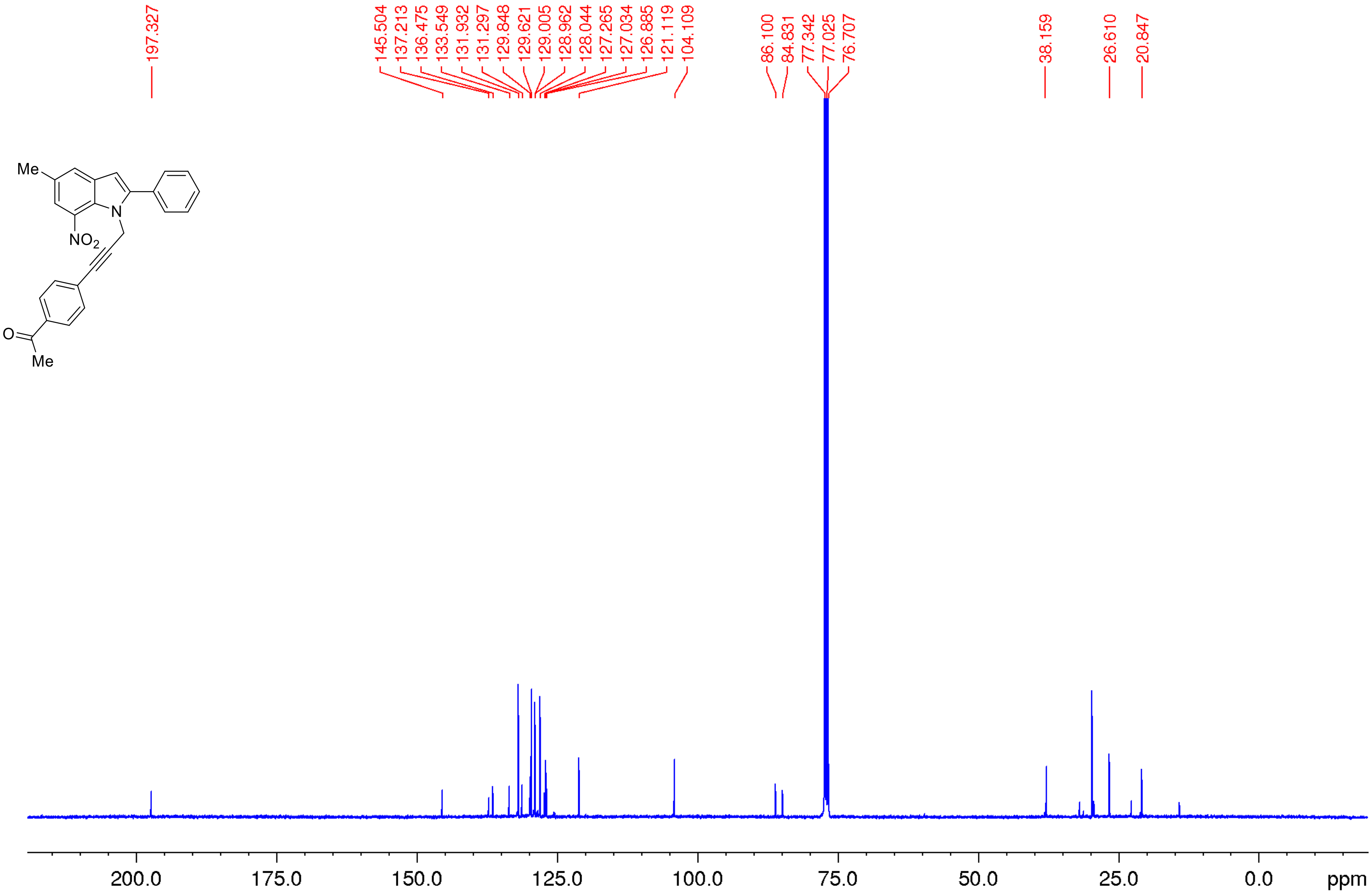

1-(4-(3-(5-methyl-7-nitro-2-phenyl-1*H*-indol-1-yl)prop-1-yn-1-yl)phenyl)ethan-1-one 7g

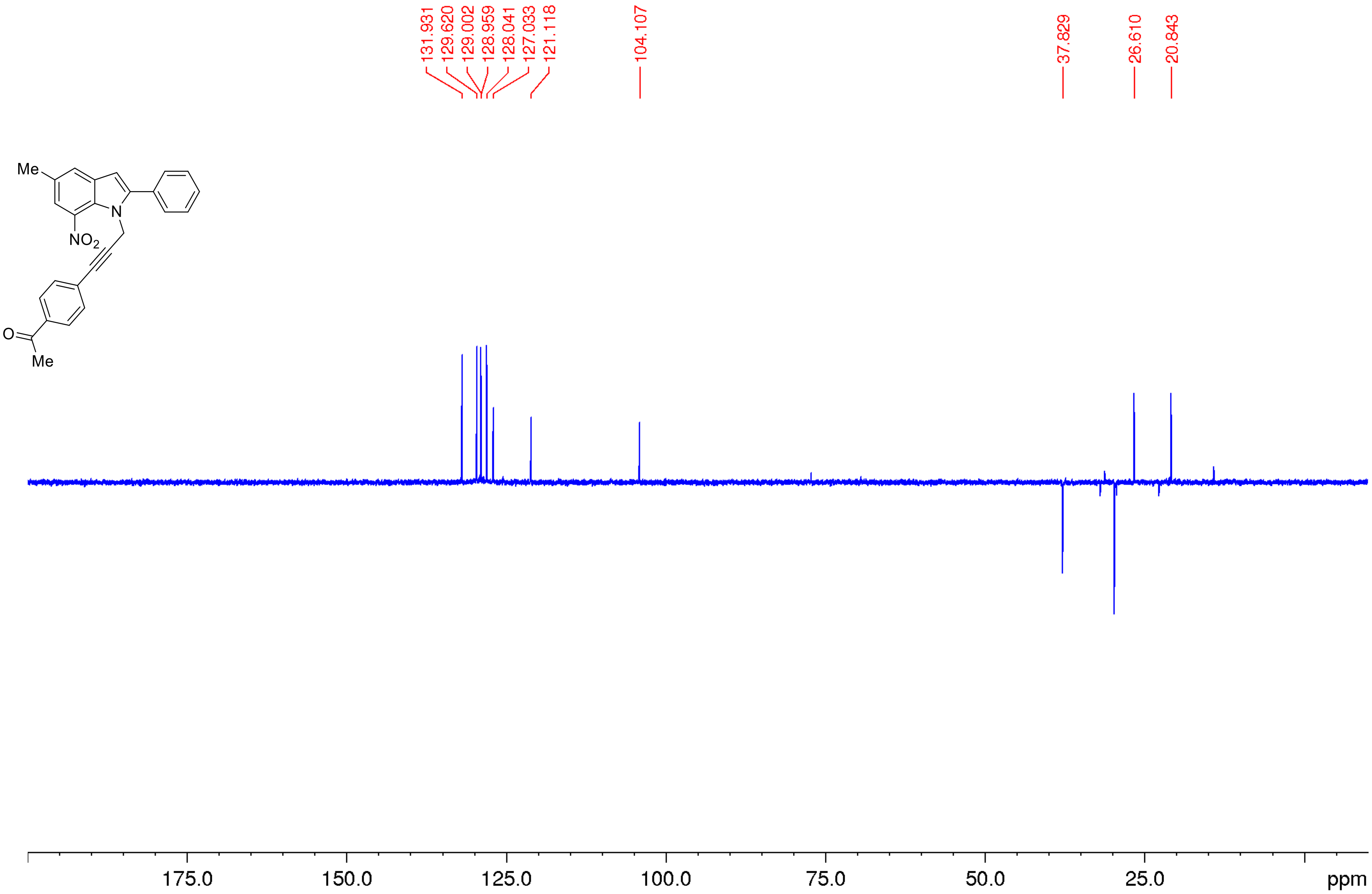

methyl 4-(1-(3-(4-chlorophenyl)prop-2-yn-1-yl)-5-methyl-7-nitro-1*H*-indol-2-yl)benzoate 7h

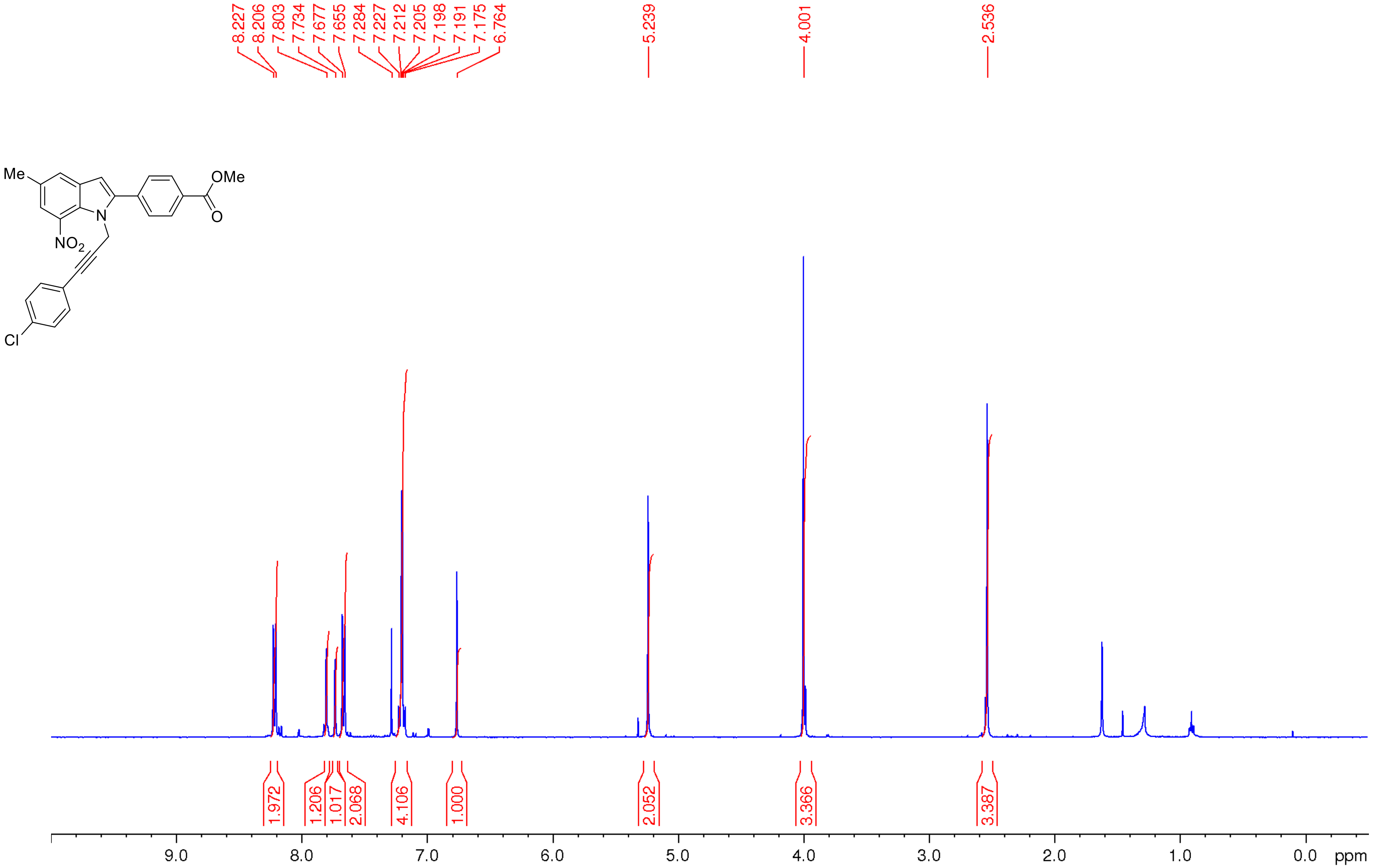

methyl 4-(1-(3-(4-chlorophenyl)prop-2-yn-1-yl)-5-methyl-7-nitro-1*H*-indol-2-yl)benzoate 7h

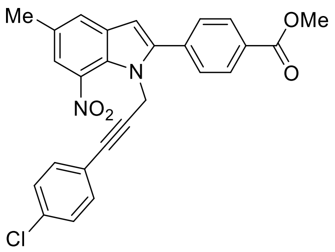

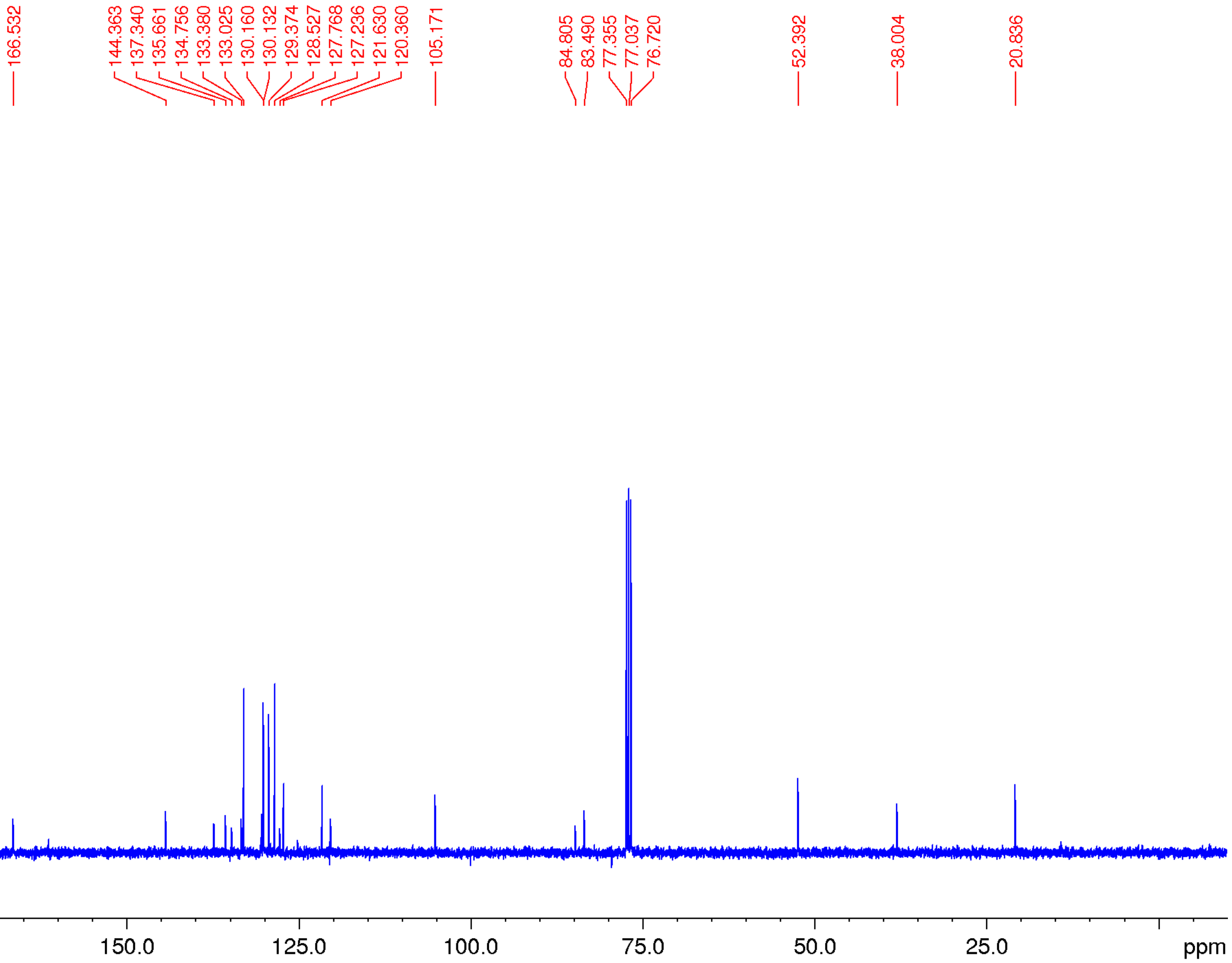

methyl 4-(1-(3-(4-chlorophenyl)prop-2-yn-1-yl)-5-methyl-7-nitro-1*H*-indol-2-yl)benzoate 7h

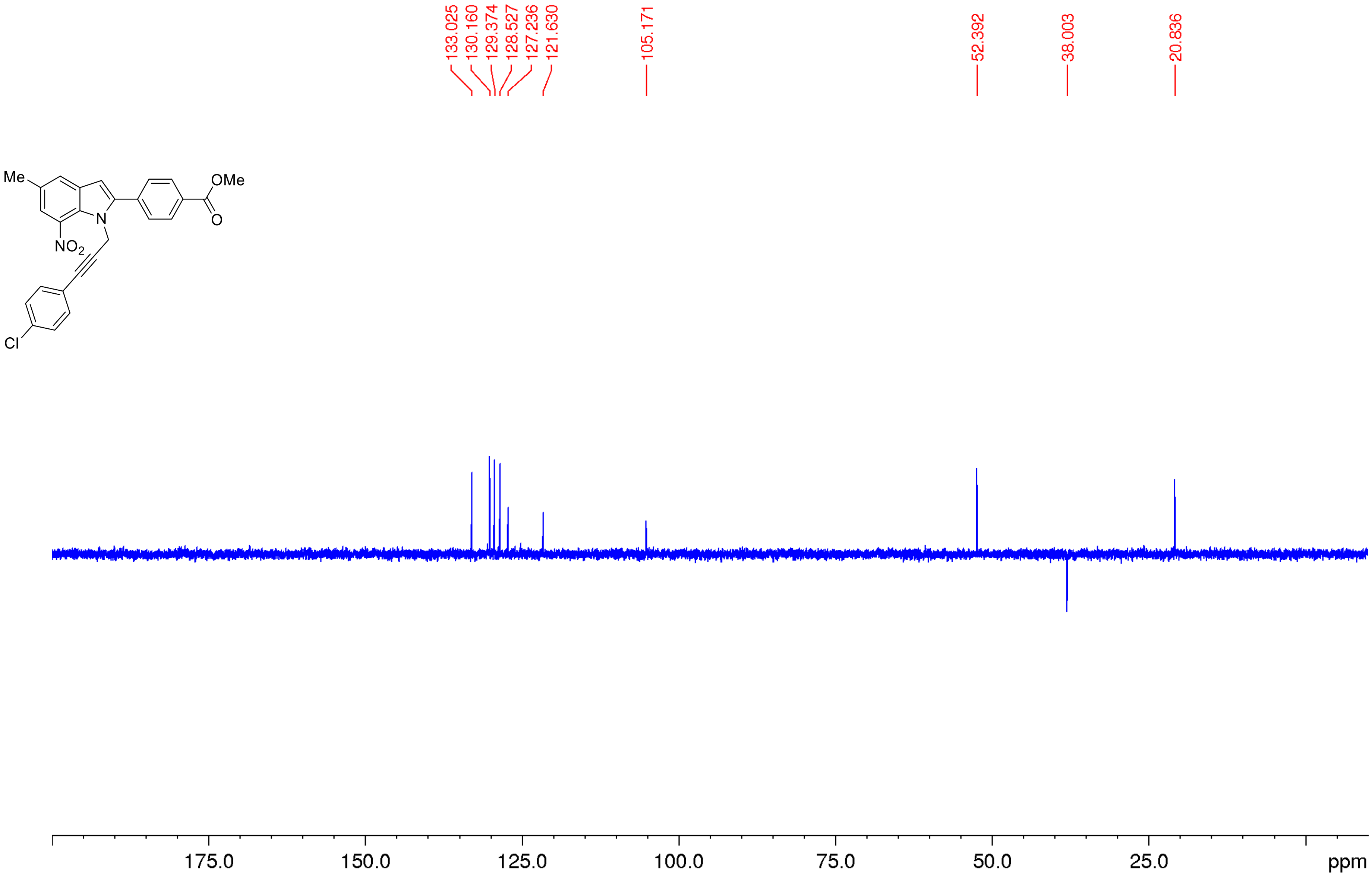

1-(3-(4-chlorophenyl)prop-2-yn-1-yl)-5-methyl-7-nitro-2-phenyl-1*H*-indole 7i

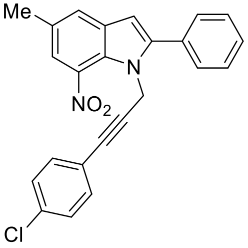

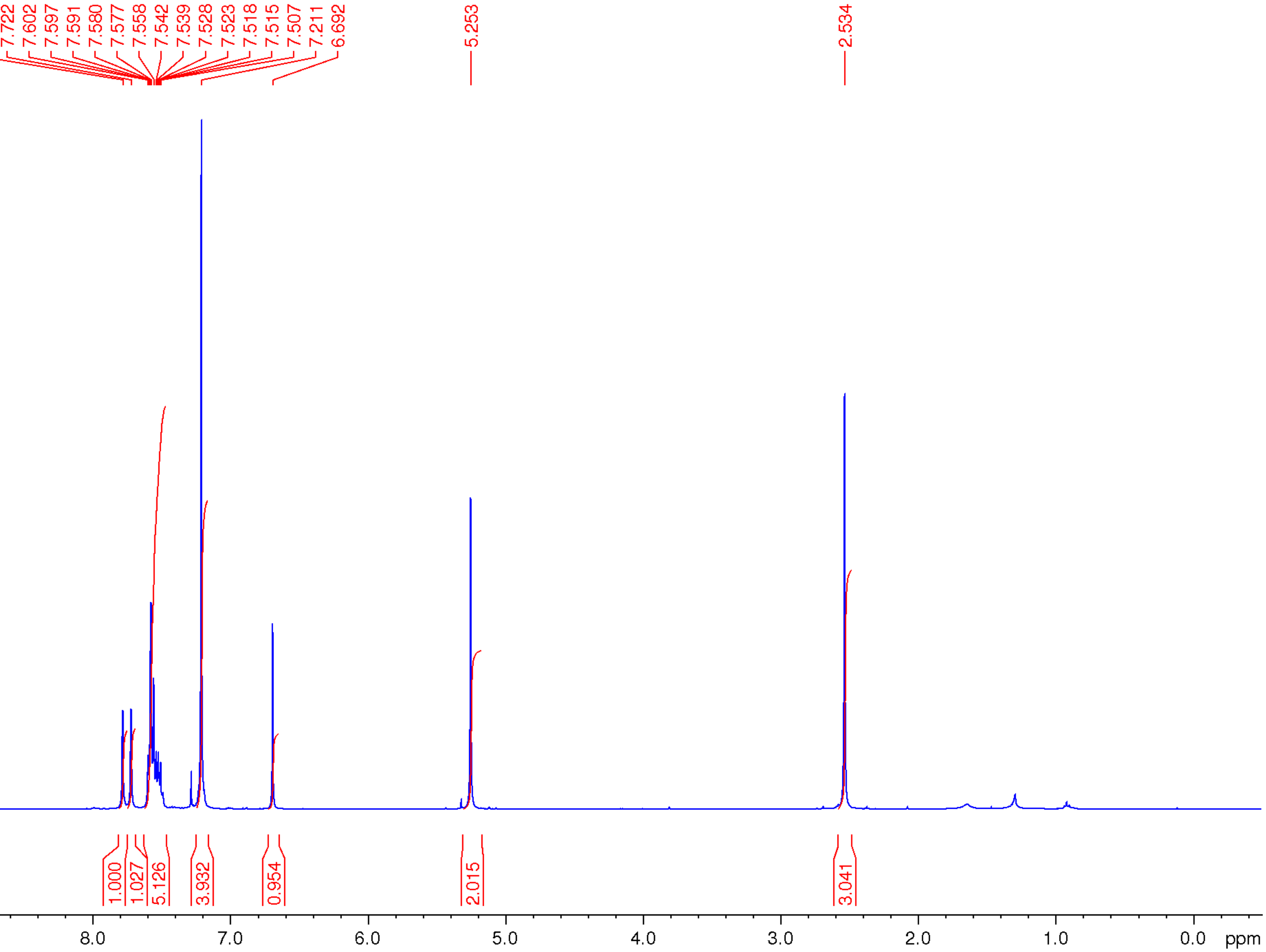

1-(3-(4-chlorophenyl)prop-2-yn-1-yl)-5-methyl-7-nitro-2-phenyl-1*H*-indole 7i

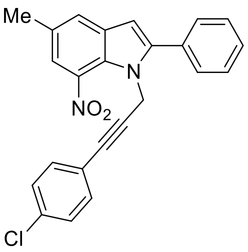

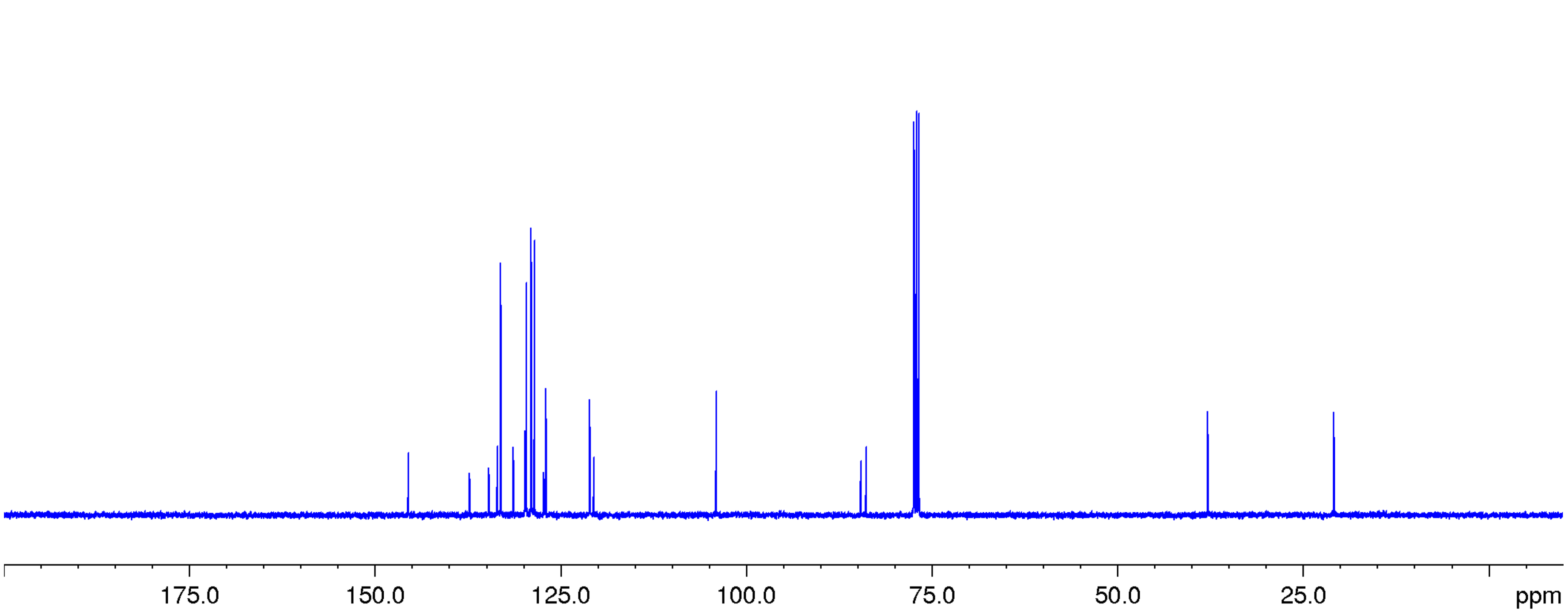

1-(3-(4-chlorophenyl)prop-2-yn-1-yl)-5-methyl-7-nitro-2-phenyl-1*H*-indole 7i

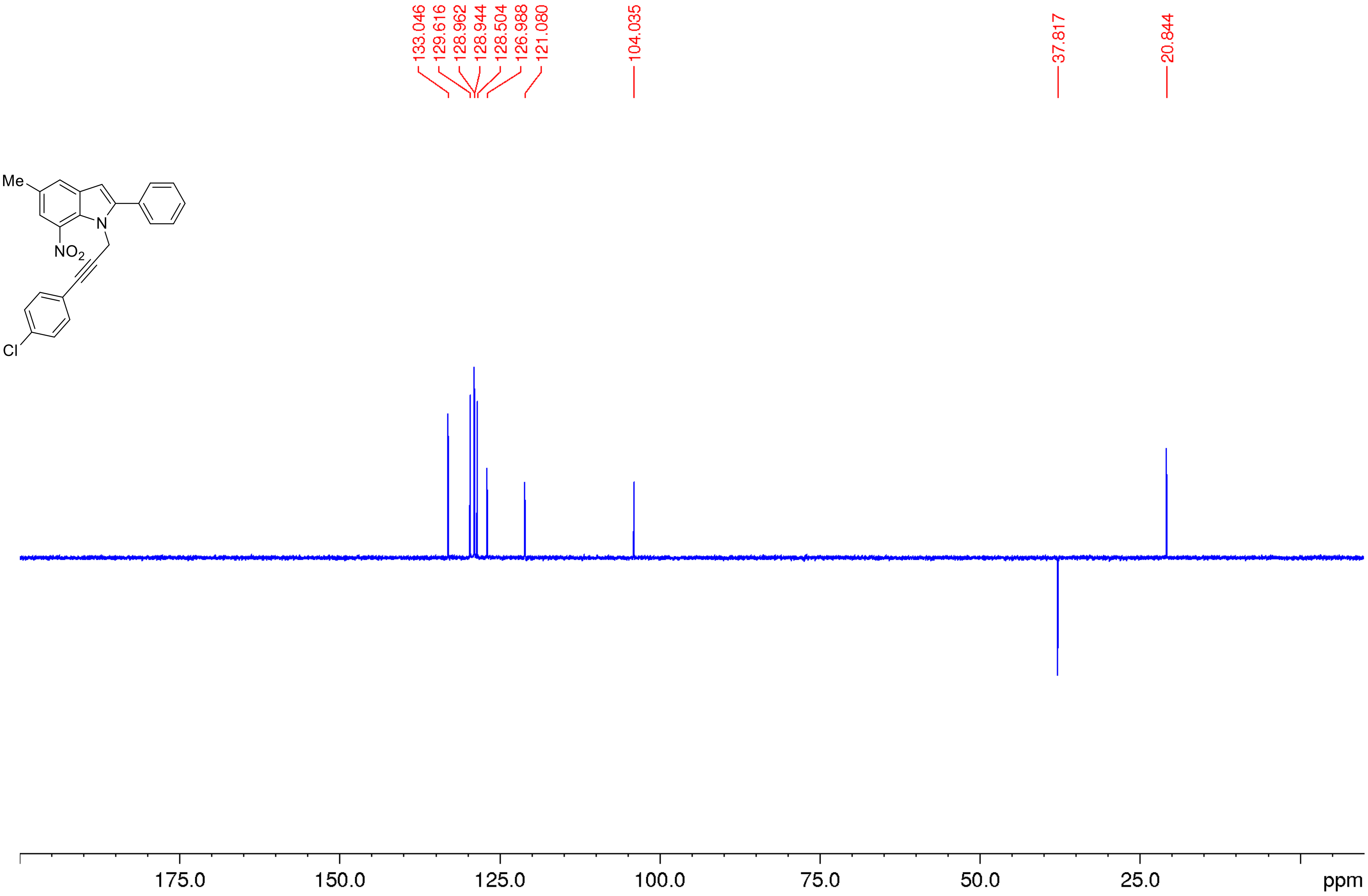

5-chloro-2-phenyl-1-(3-phenylprop-2-yn-1-yl)-1*H*-indol-7-amine 1a

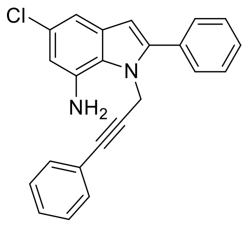

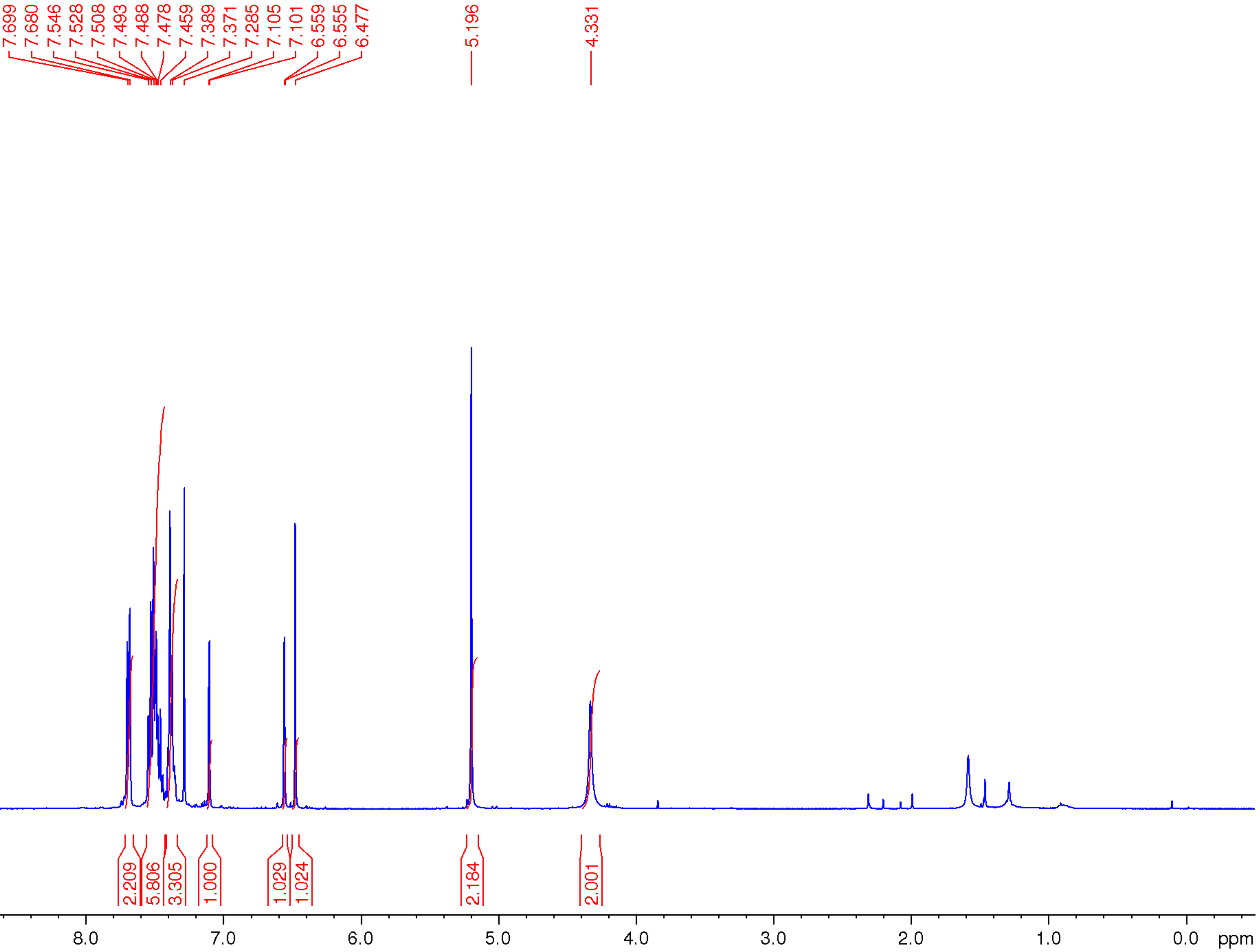

5-chloro-2-phenyl-1-(3-phenylprop-2-yn-1-yl)-1*H*-indol-7-amine 1a

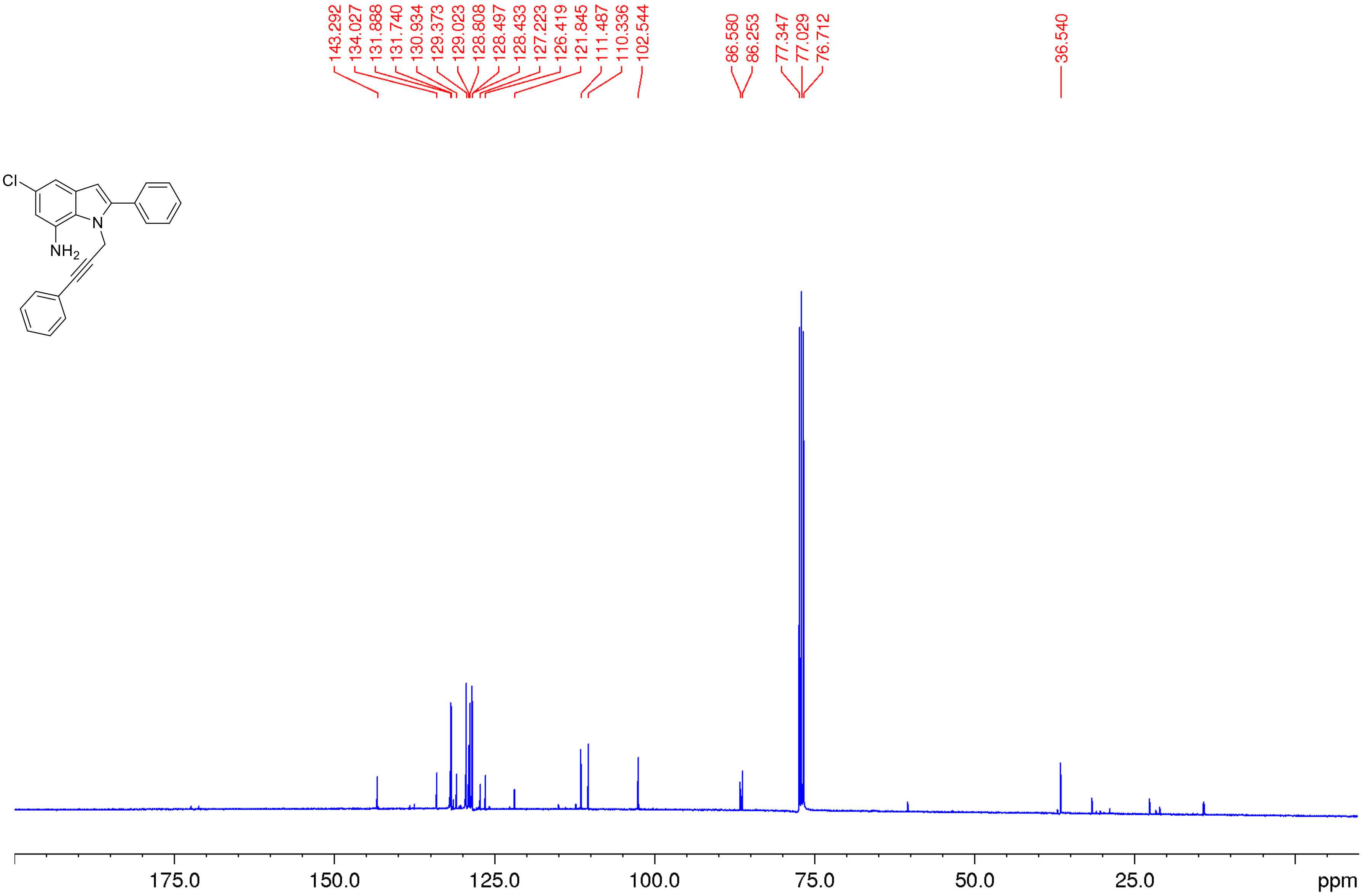

5-chloro-2-phenyl-1-(3-phenylprop-2-yn-1-yl)-1*H*-indol-7-amine 1a

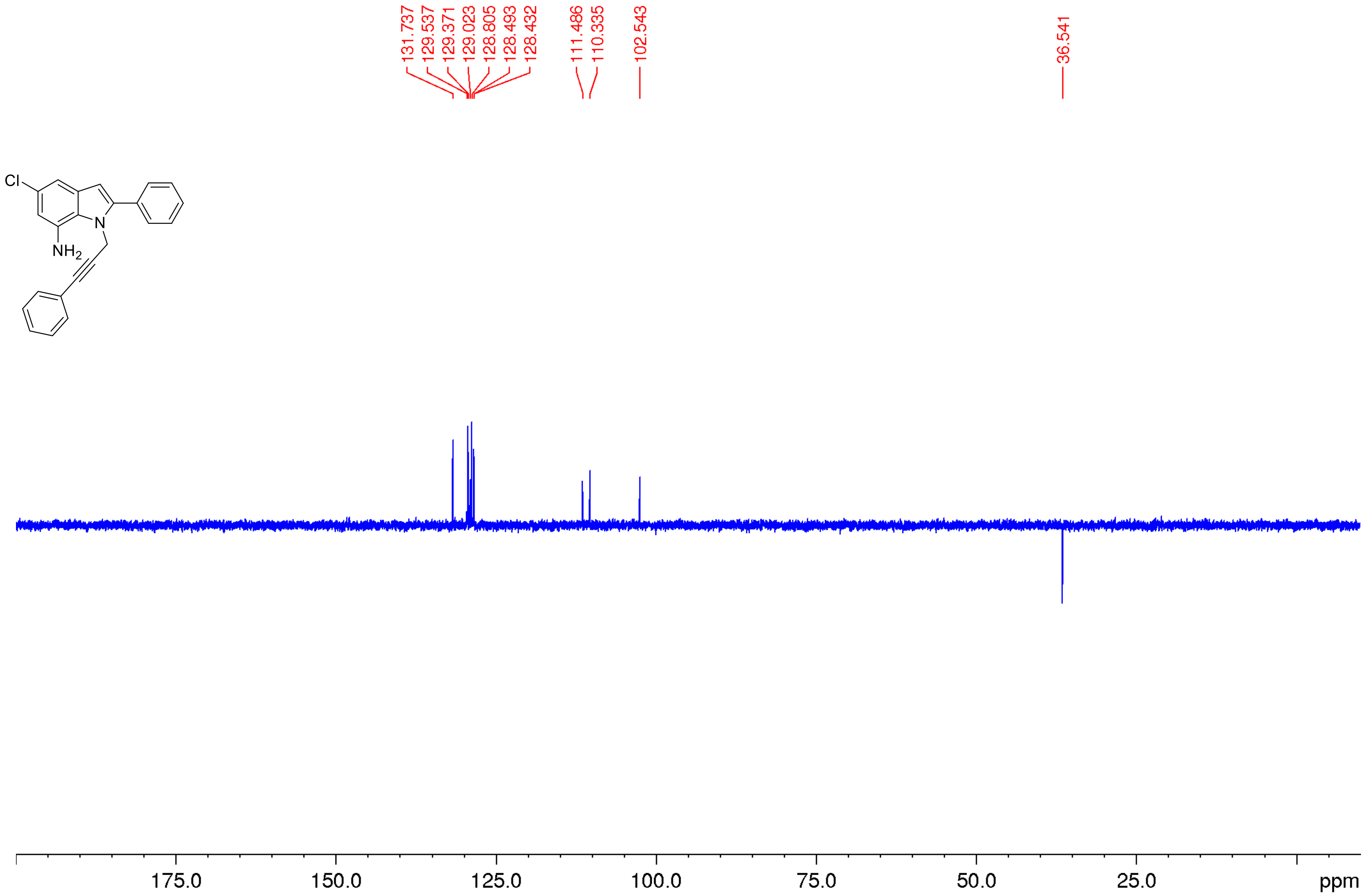

5-chloro-1-(3-(4-chlorophenyl)prop-2-yn-1-yl)-2-phenyl-1*H*-indol-7-amine 1b

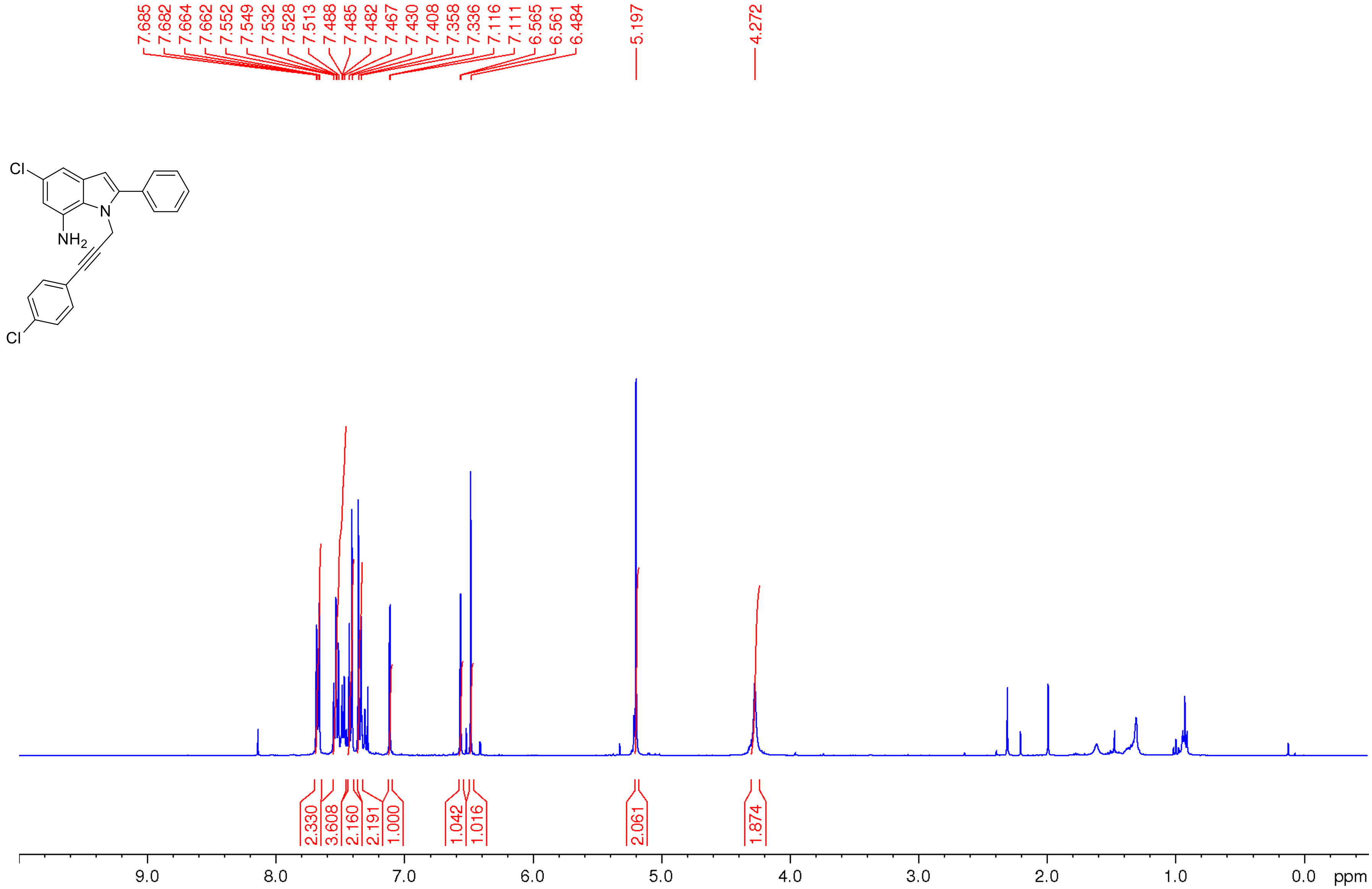

5-chloro-1-(3-(4-chlorophenyl)prop-2-yn-1-yl)-2-phenyl-1*H*-indol-7-amine 1b

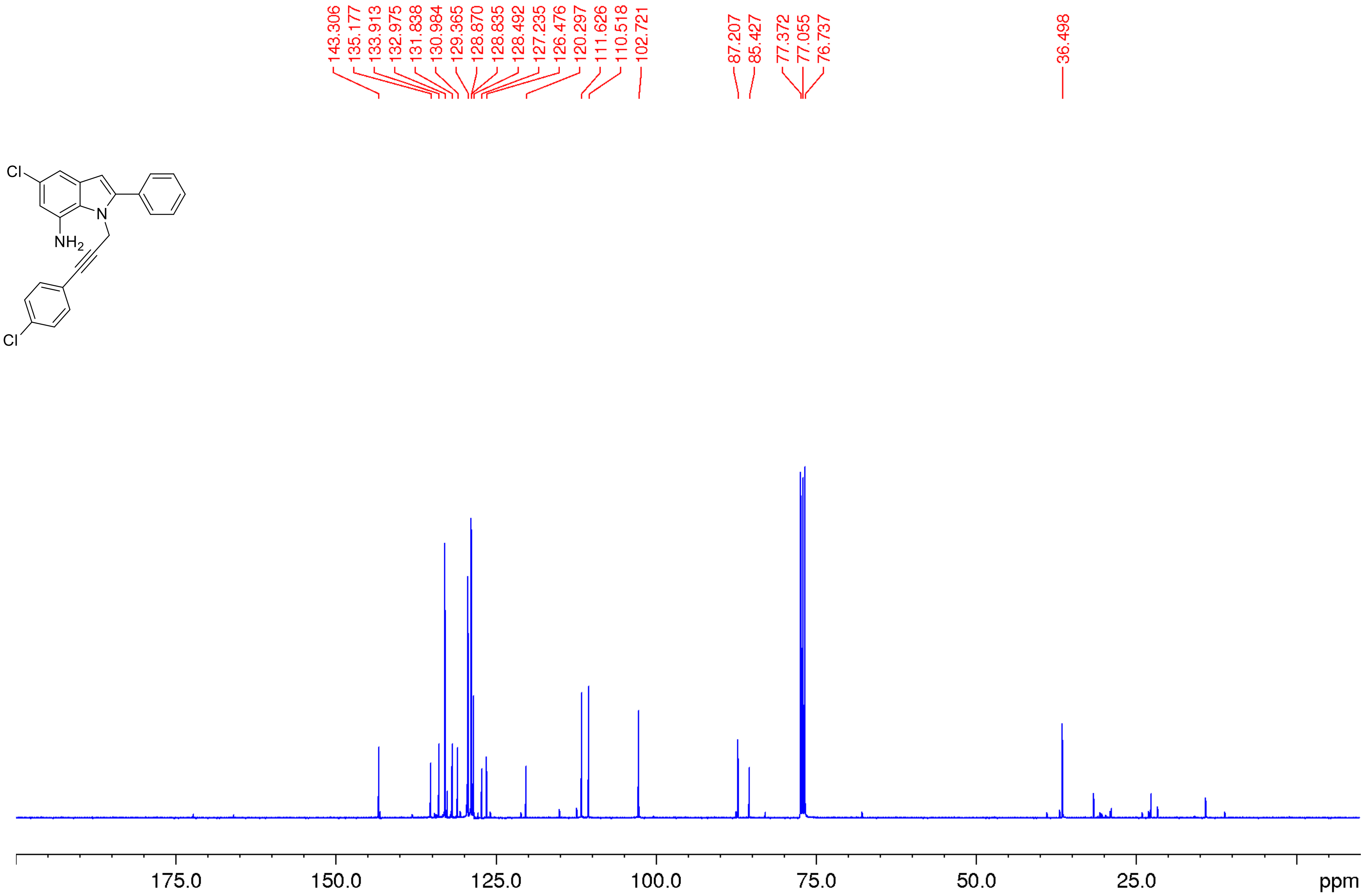

5-chloro-1-(3-(4-chlorophenyl)prop-2-yn-1-yl)-2-phenyl-1*H*-indol-7-amine 1b

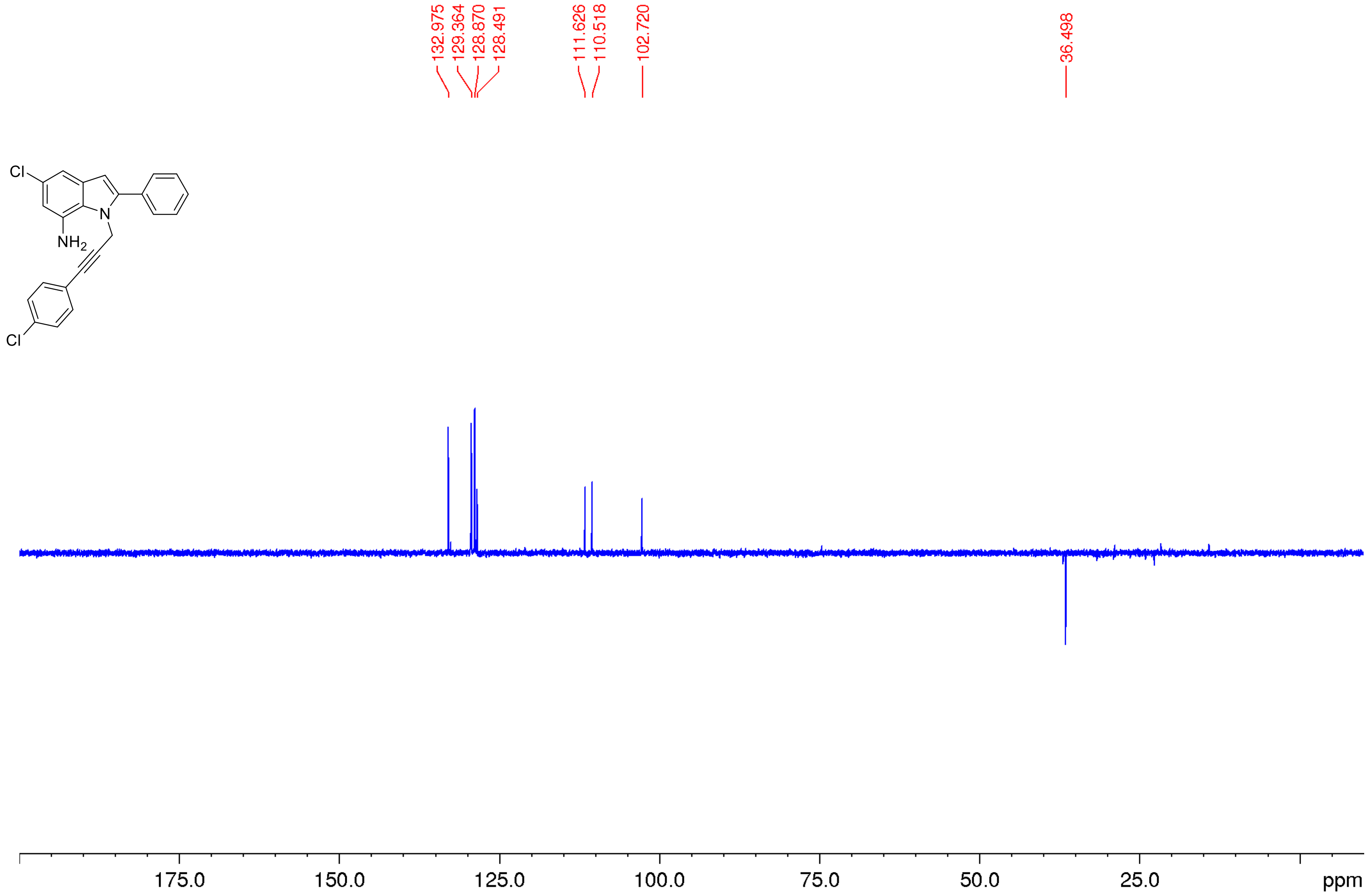

5-chloro-1-(3-(4-methoxyphenyl)prop-2-yn-1-yl)-2-phenyl-1H-indol-7-amine 1c

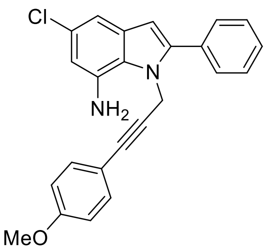

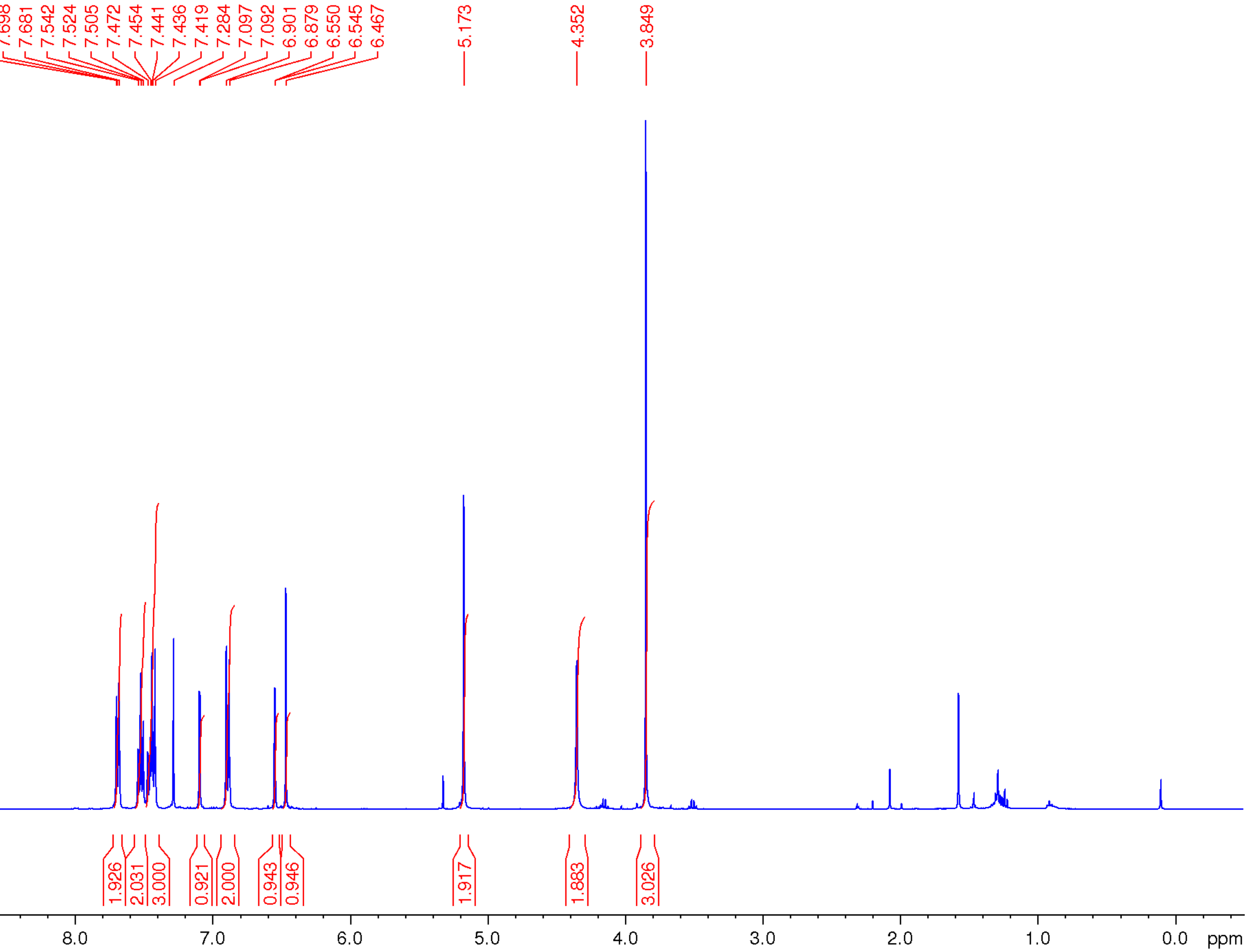

5-chloro-1-(3-(4-methoxyphenyl)prop-2-yn-1-yl)-2-phenyl-1H-indol-7-amine 1c

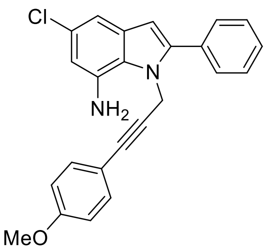

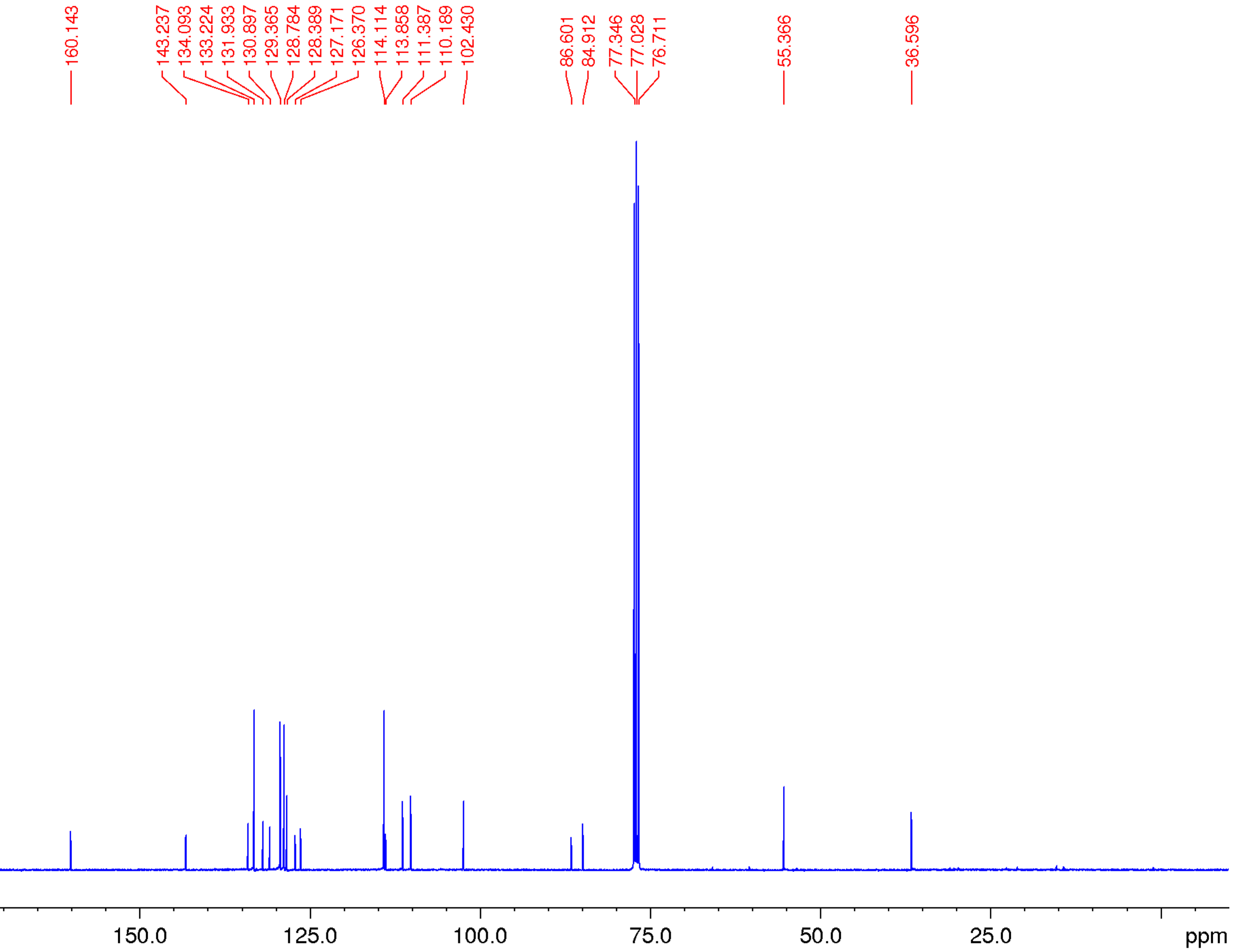

5-chloro-1-(3-(4-methoxyphenyl)prop-2-yn-1-yl)-2-phenyl-1*H*-indol-7-amine 1c

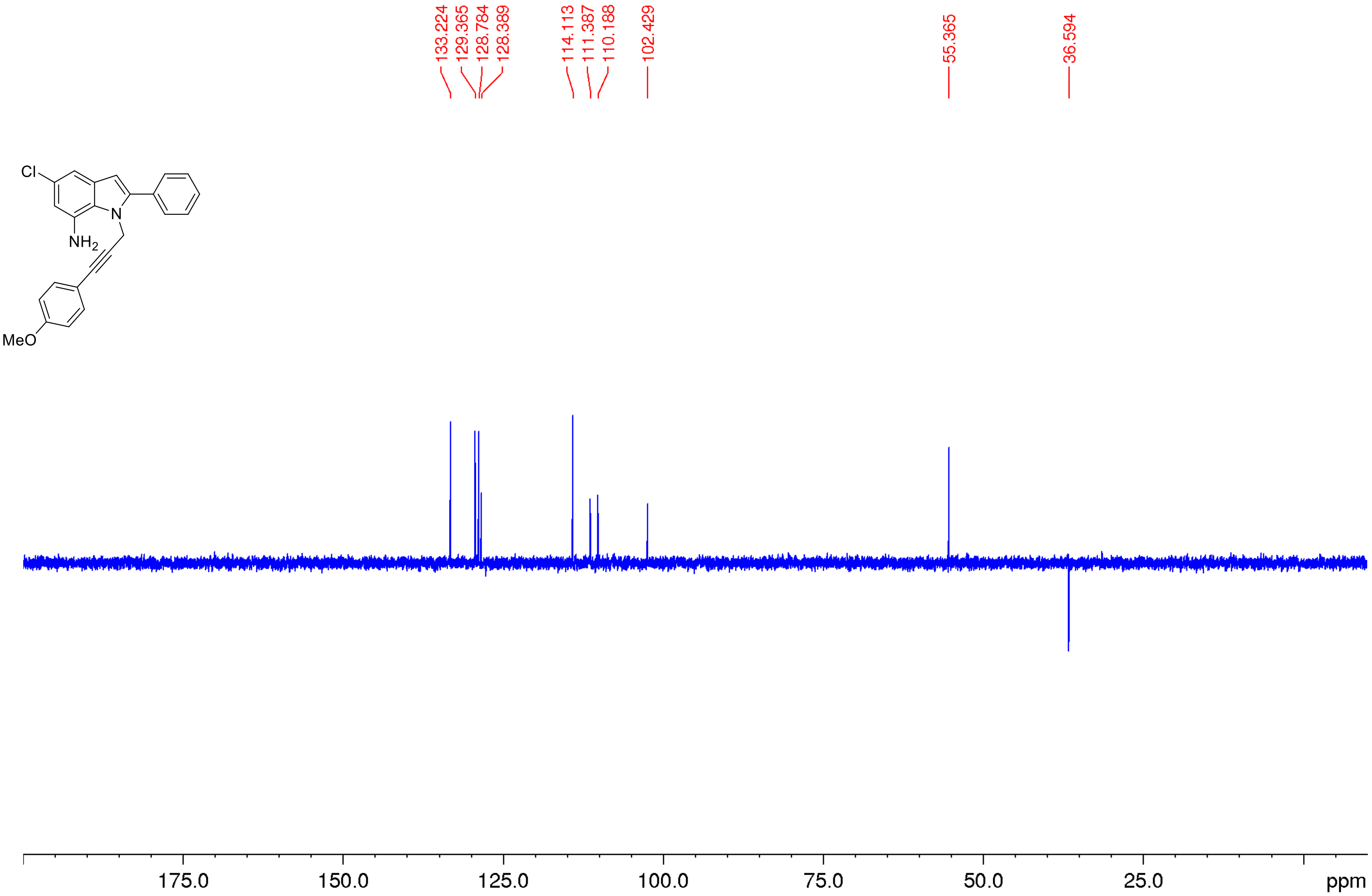

1-(4-(3-(7-amino-5-chloro-2-phenyl-1*H*-indol-1-yl)prop-1-yn-1-yl)phenyl)ethan-1-one 1d

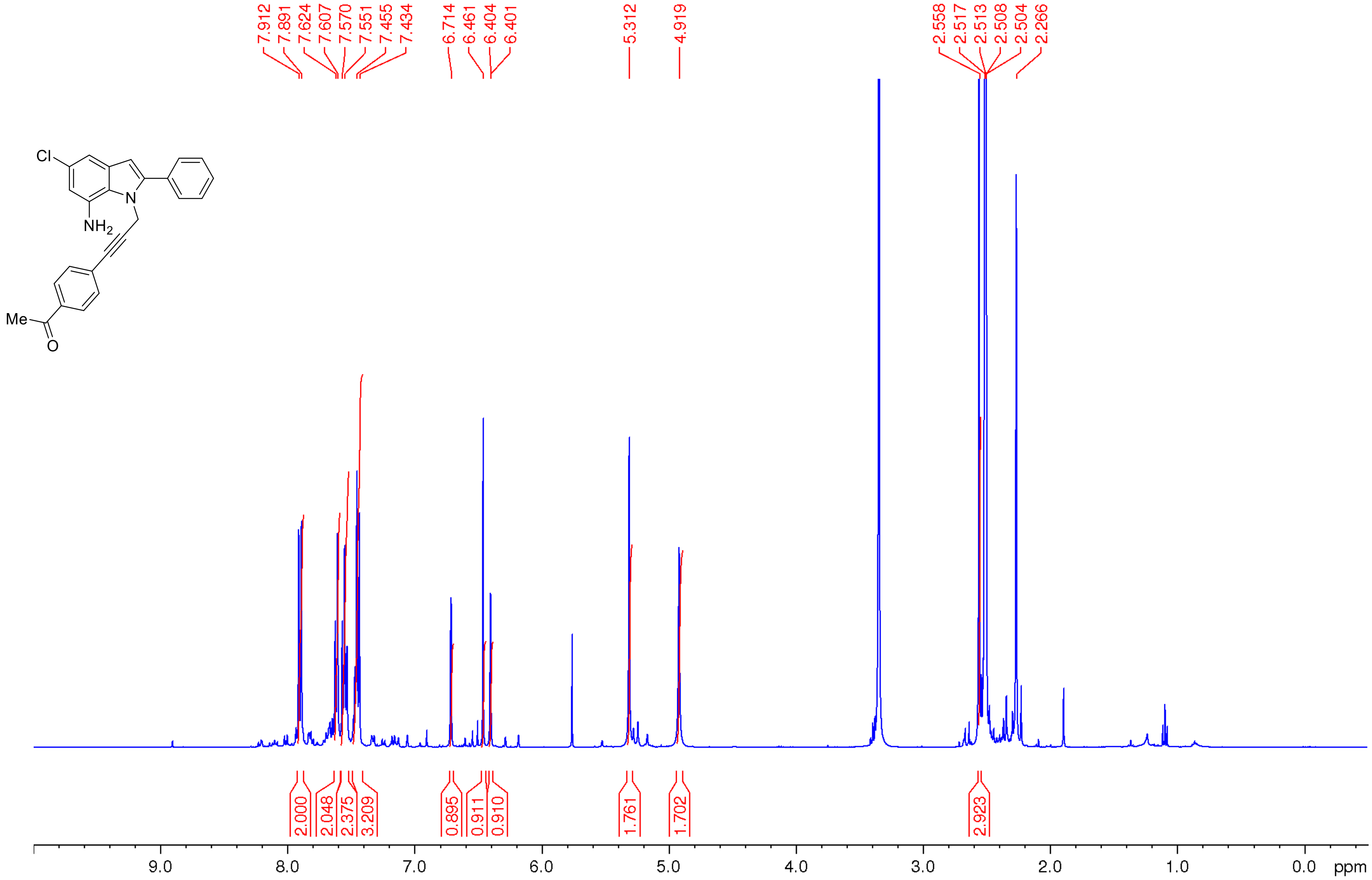

1-(4-(3-(7-amino-5-chloro-2-phenyl-1*H*-indol-1-yl)prop-1-yn-1-yl)phenyl)ethan-1-one 1d

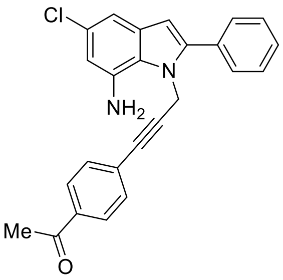

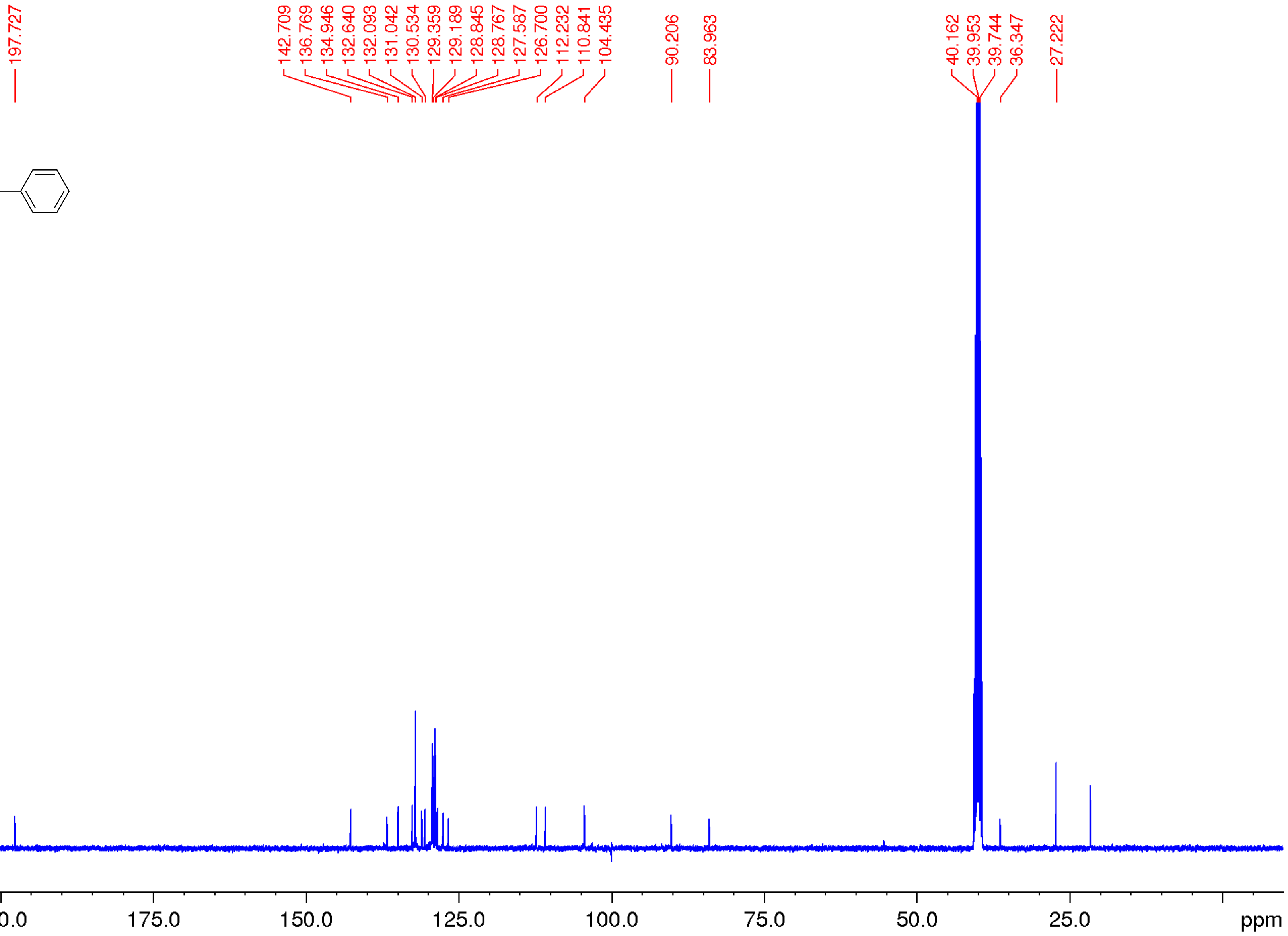

1-(4-(3-(7-amino-5-chloro-2-phenyl-1*H*-indol-1-yl)prop-1-yn-1-yl)phenyl)ethan-1-one 1d

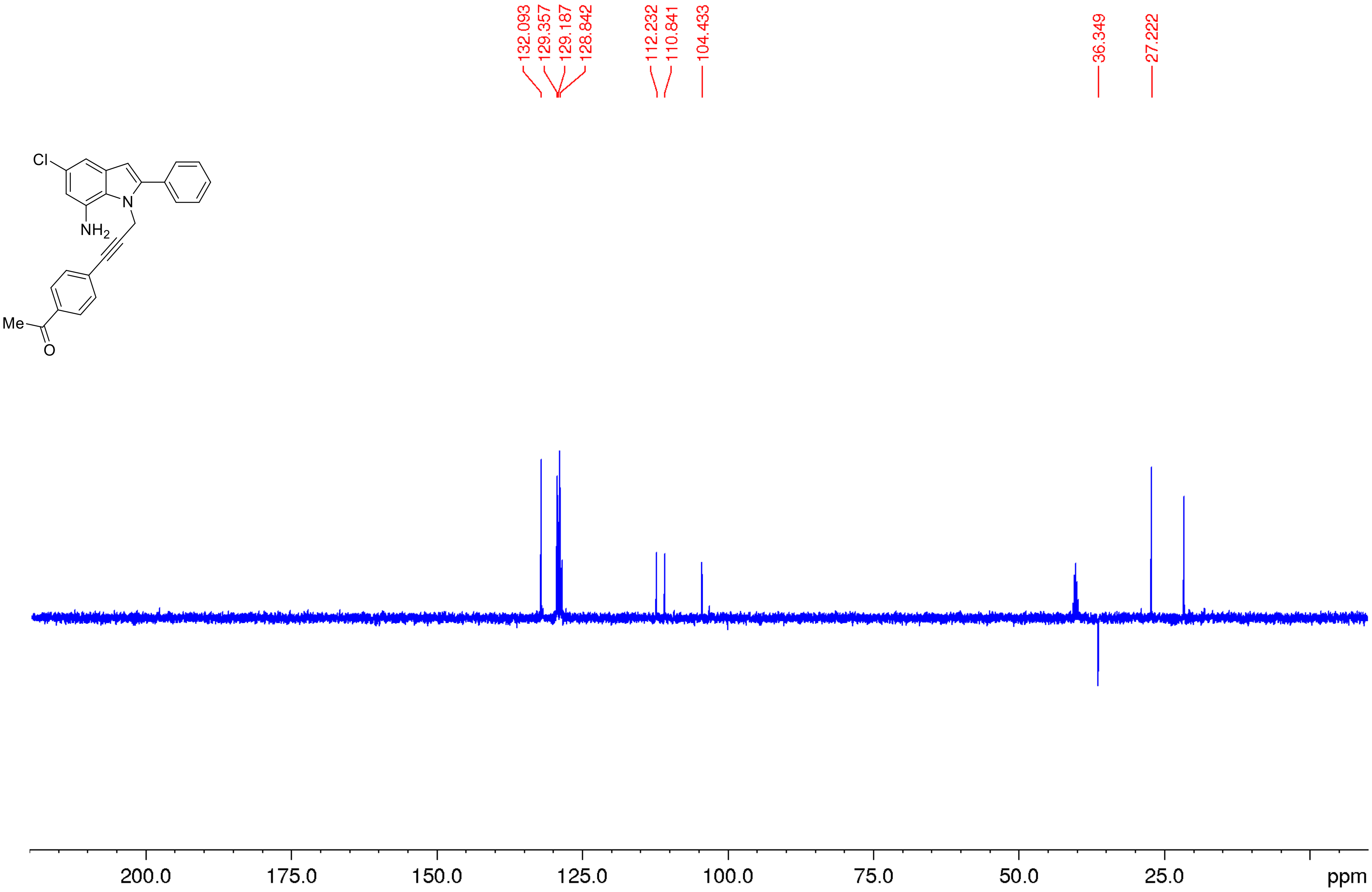

5-chloro-2-(4-methoxyphenyl)-1-(3-(3-(trifluoromethyl)phenyl)prop-2-yn-1-yl)-1*H*-indol-7-amine 1e

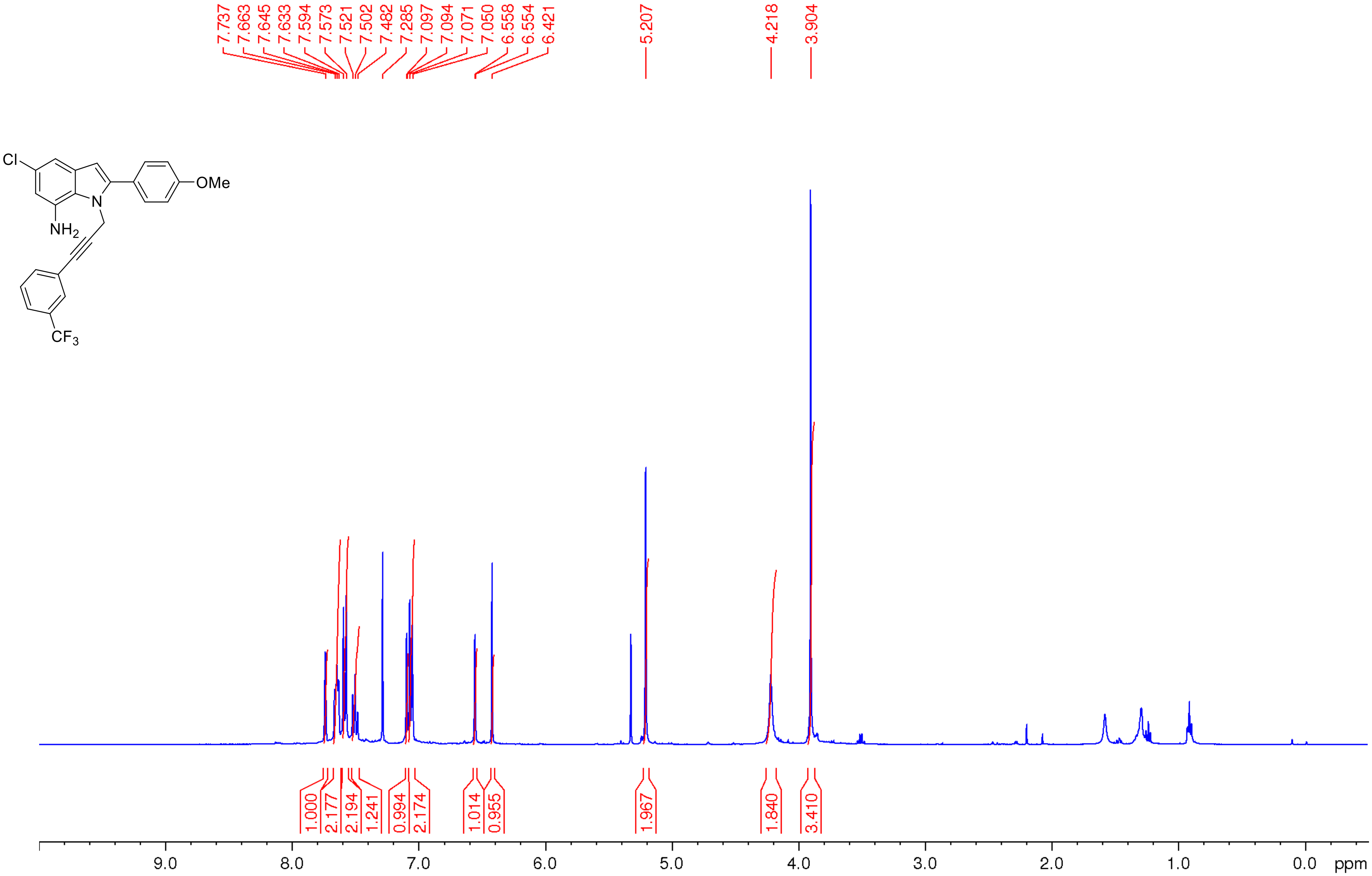

5-chloro-2-(4-methoxyphenyl)-1-(3-(3-(trifluoromethyl)phenyl)prop-2-yn-1-yl)-1*H*-indol-7-amine 1e

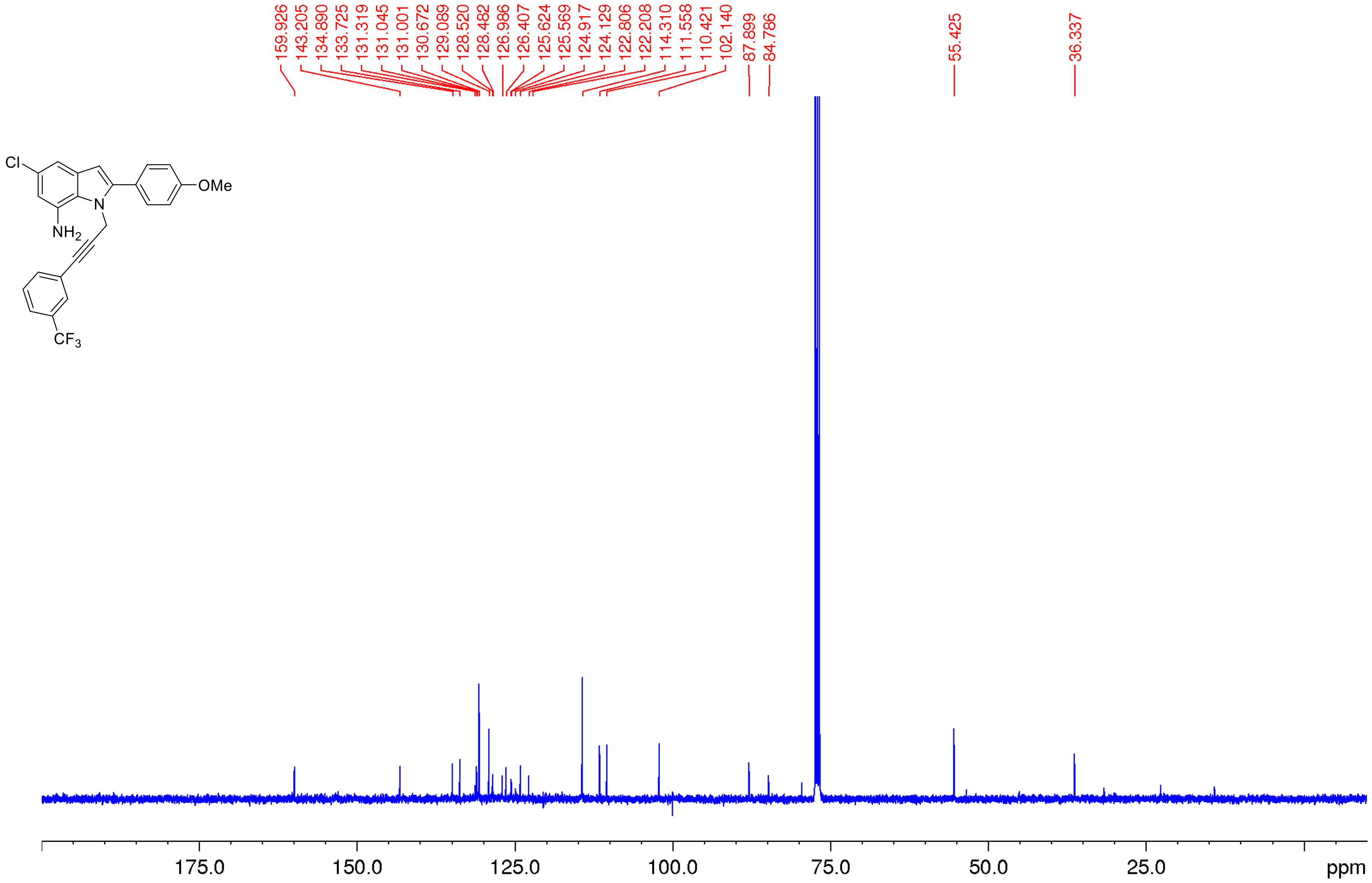

5-chloro-2-(4-methoxyphenyl)-1-(3-(3-(trifluoromethyl)phenyl)prop-2-yn-1-yl)-1*H*-indol-7-amine 1e

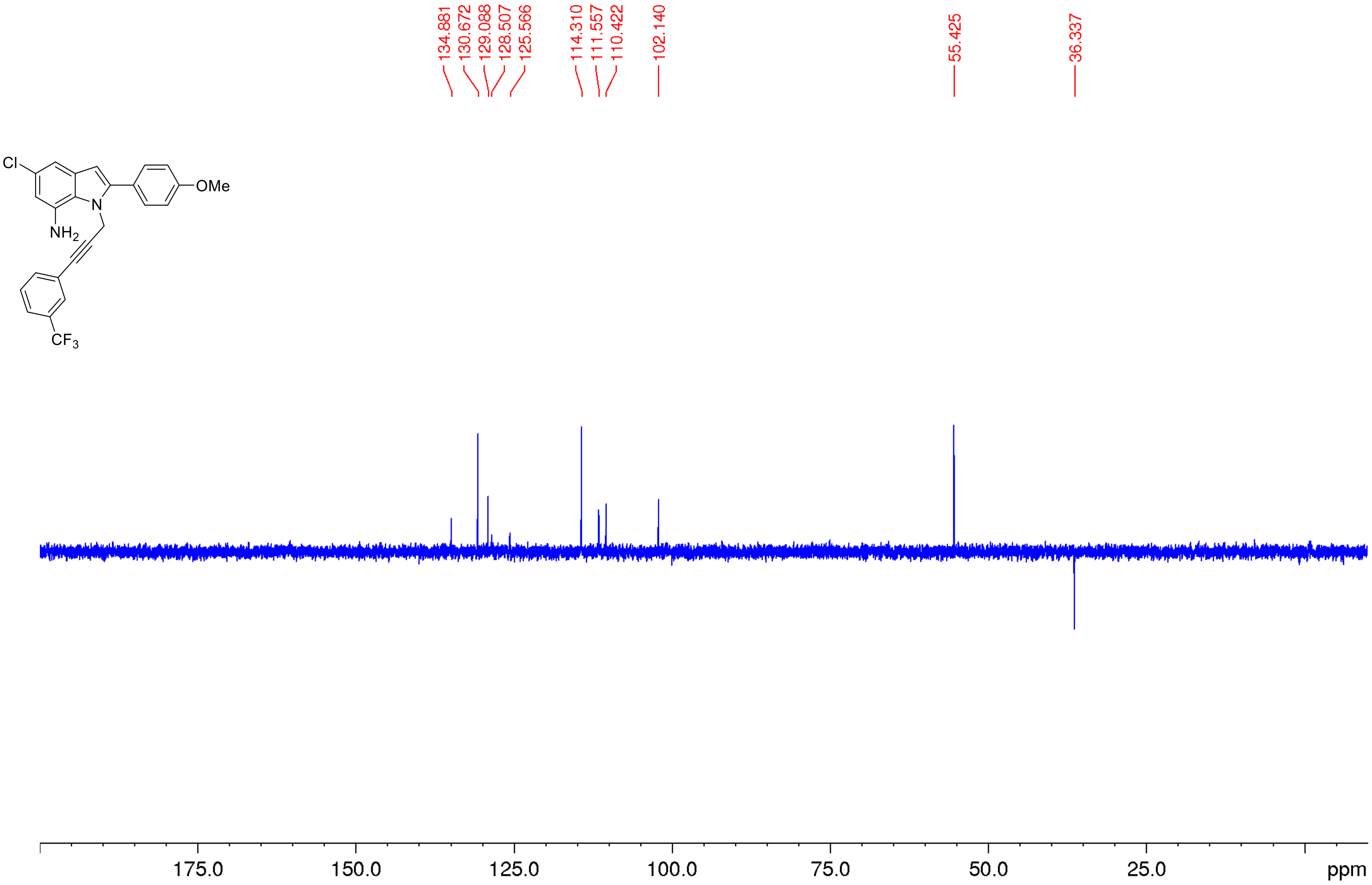

5-chloro-2-(4-methoxyphenyl)-1-(3-(3-(trifluoromethyl)phenyl)prop-2-yn-1-yl)-1*H*-indol-7-amine 1e

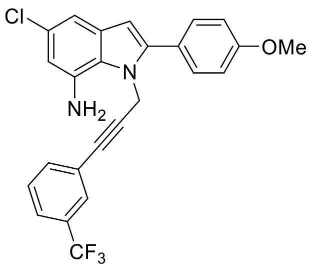

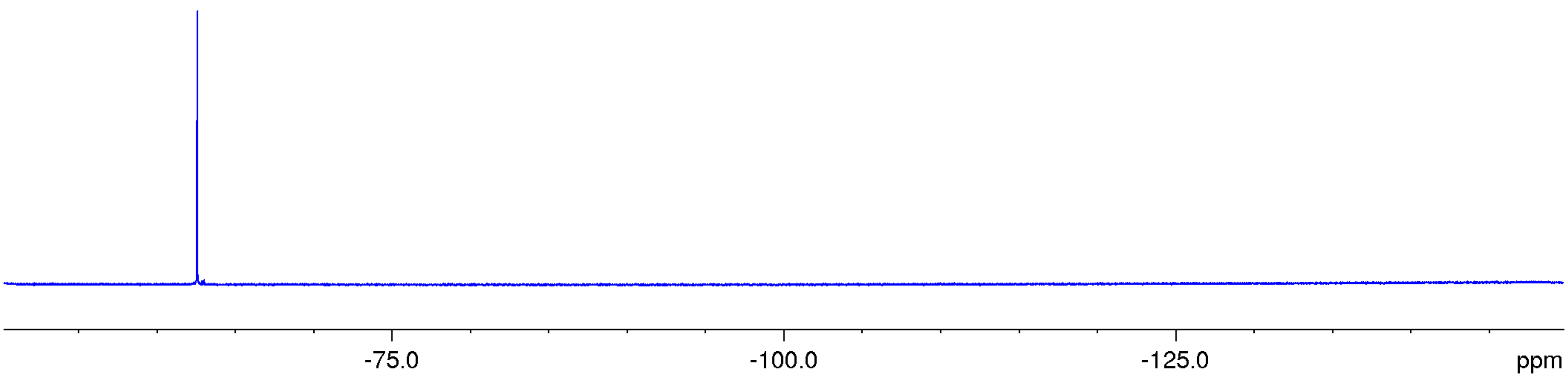

5-cloro-2-(4-metossifenil)-1-(3-(4-metossifenil)prop-2-in-1-il)-7-ammino-1*H*-indolo amine 1f

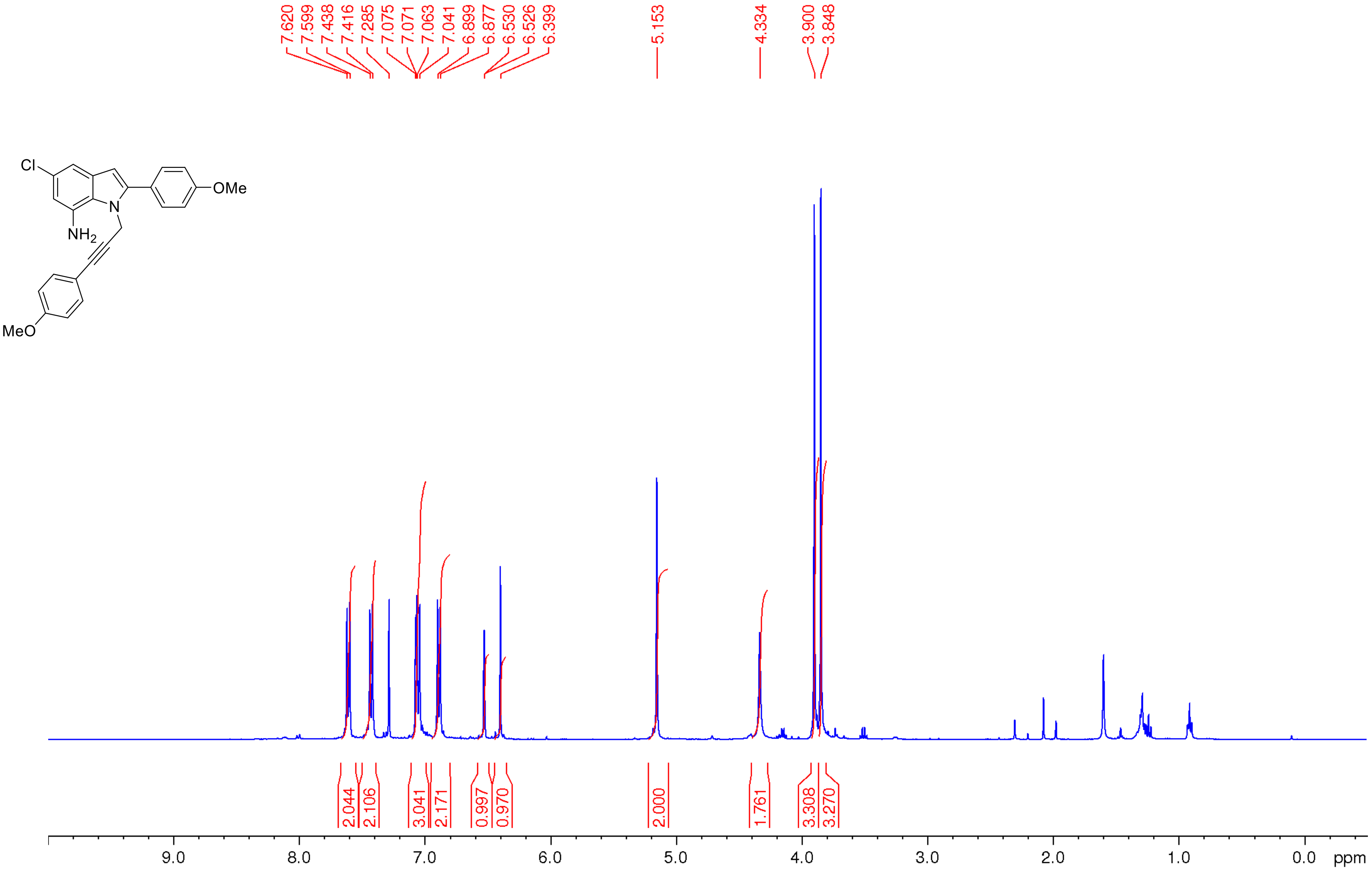

5-cloro-2-(4-metossifenil)-1-(3-(4-metossifenil)prop-2-in-1-il)-7-ammino-1*H*-indolo amine 1f

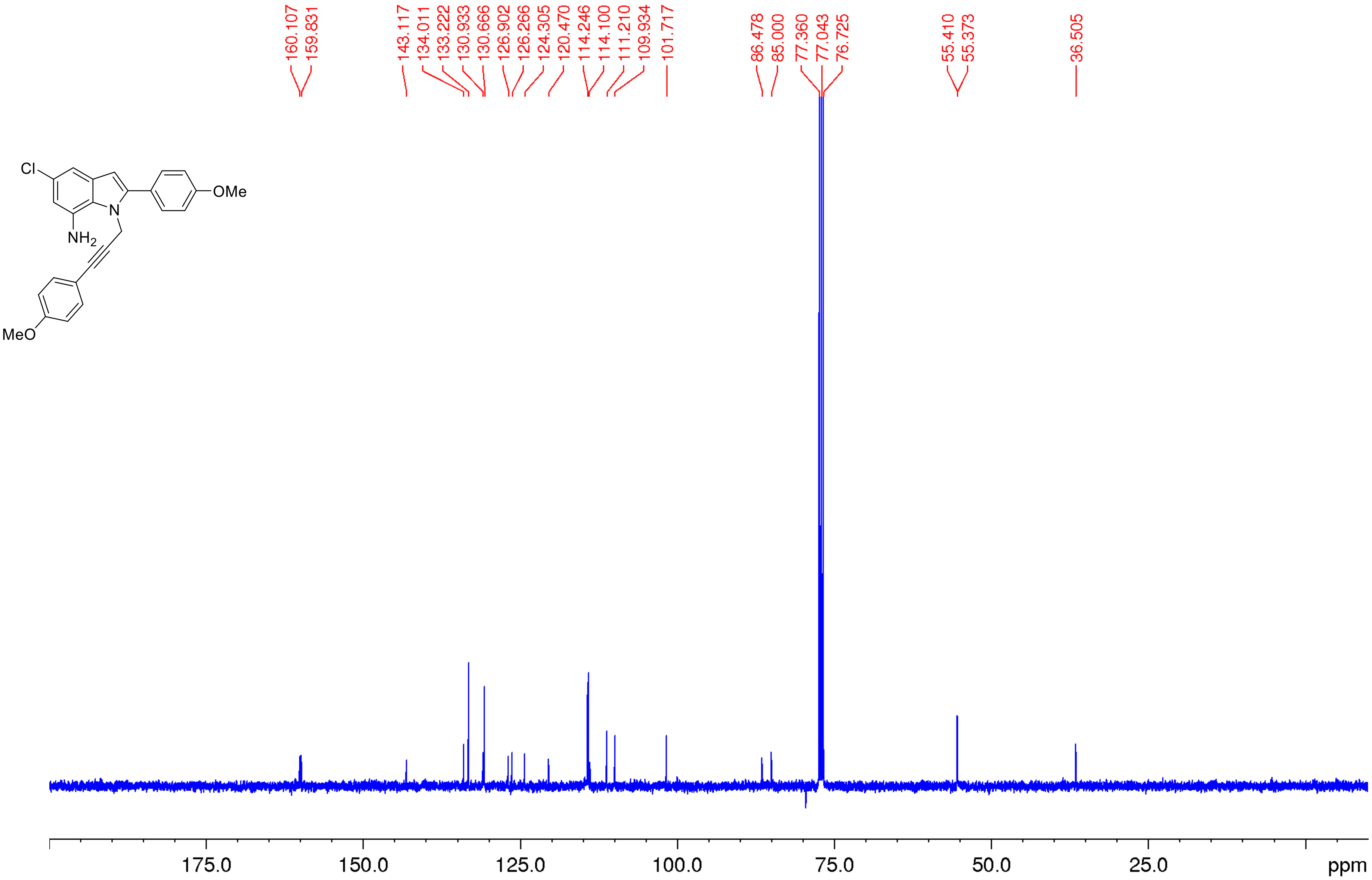

5-cloro-2-(4-metossifenil)-1-(3-(4-metossifenil)prop-2-in-1-il)-7-ammino-1*H*-indolo amine 1f

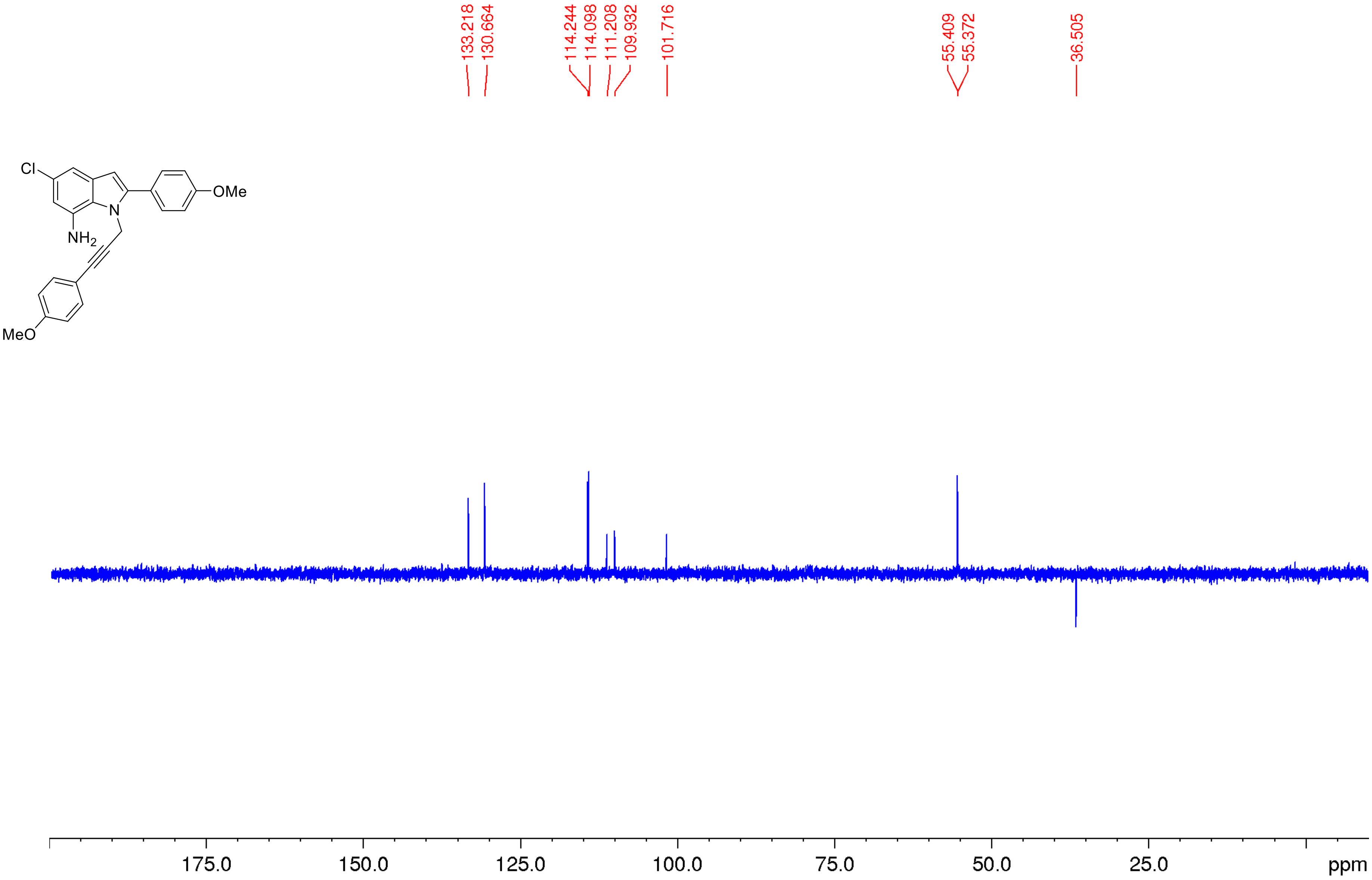

1-(4-(3-(7-amino-5-methyl-2-phenyl-1*H*-indol-1-yl)prop-1-yn-1-yl)phenyl)ethan-1-one 1g

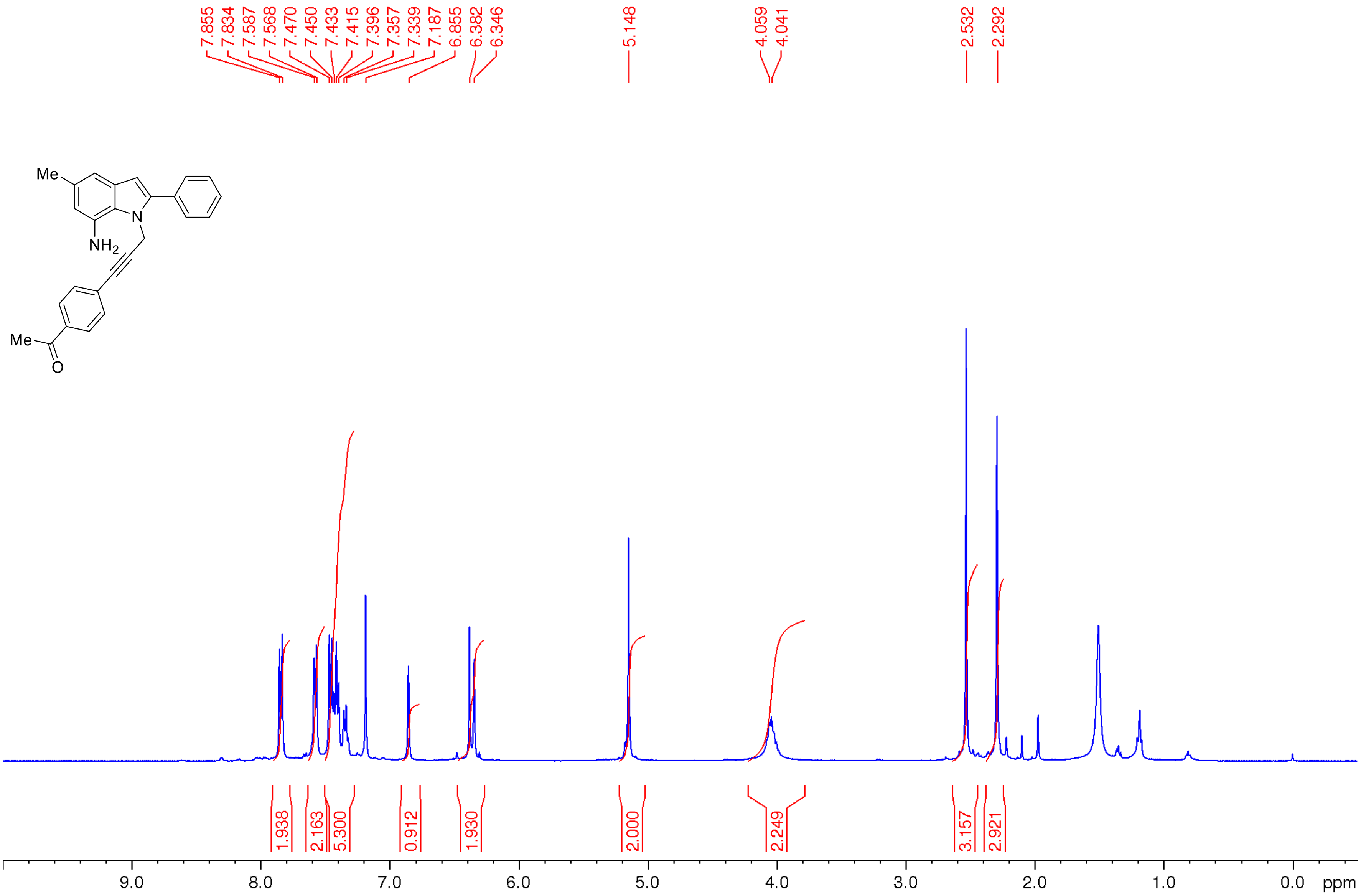

1-(4-(3-(7-amino-5-methyl-2-phenyl-1*H*-indol-1-yl)prop-1-yn-1-yl)phenyl)ethan-1-one 1g

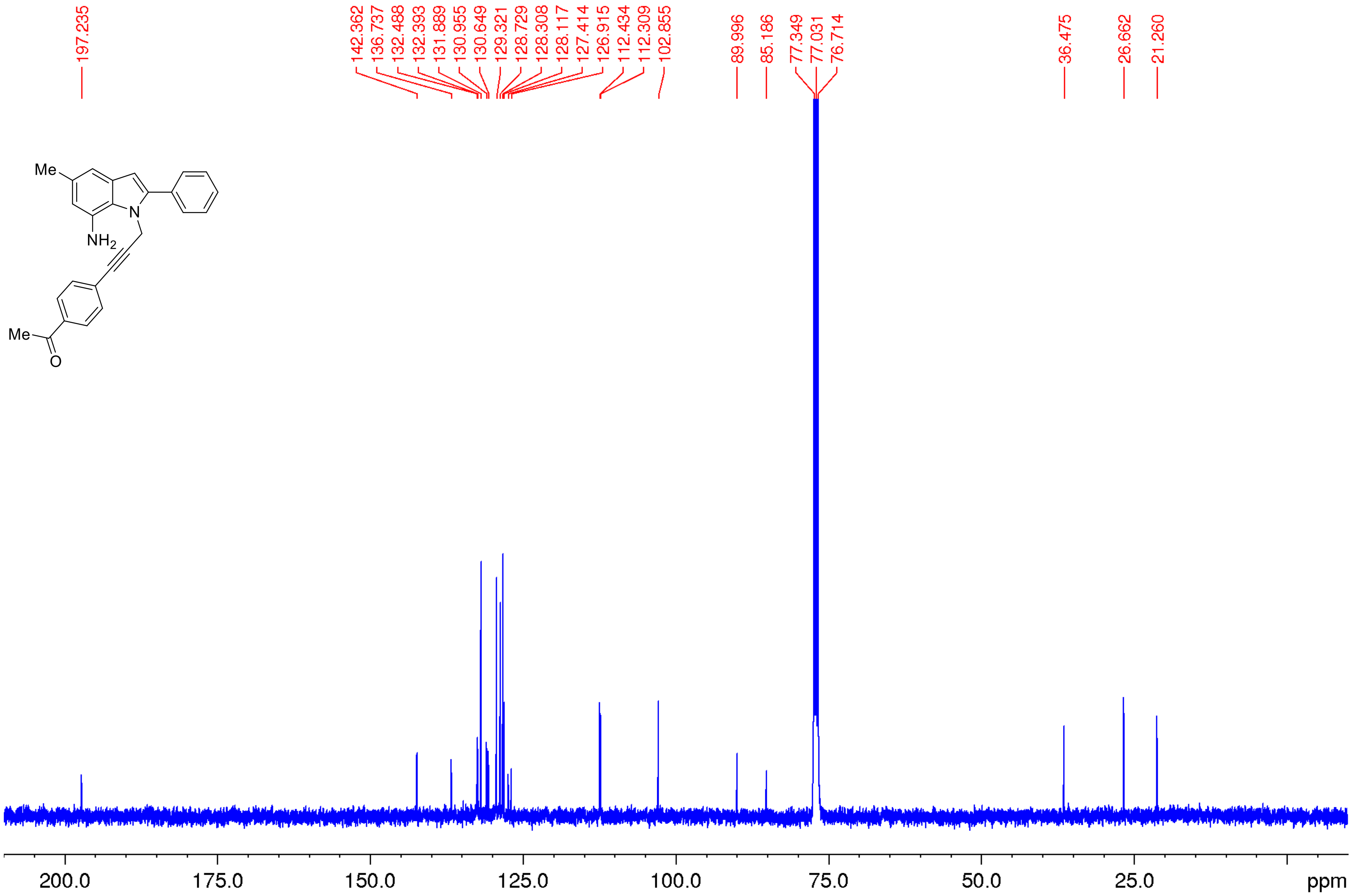

1-(4-(3-(7-amino-5-methyl-2-phenyl-1*H*-indol-1-yl)prop-1-yn-1-yl)phenyl)ethan-1-one 1g

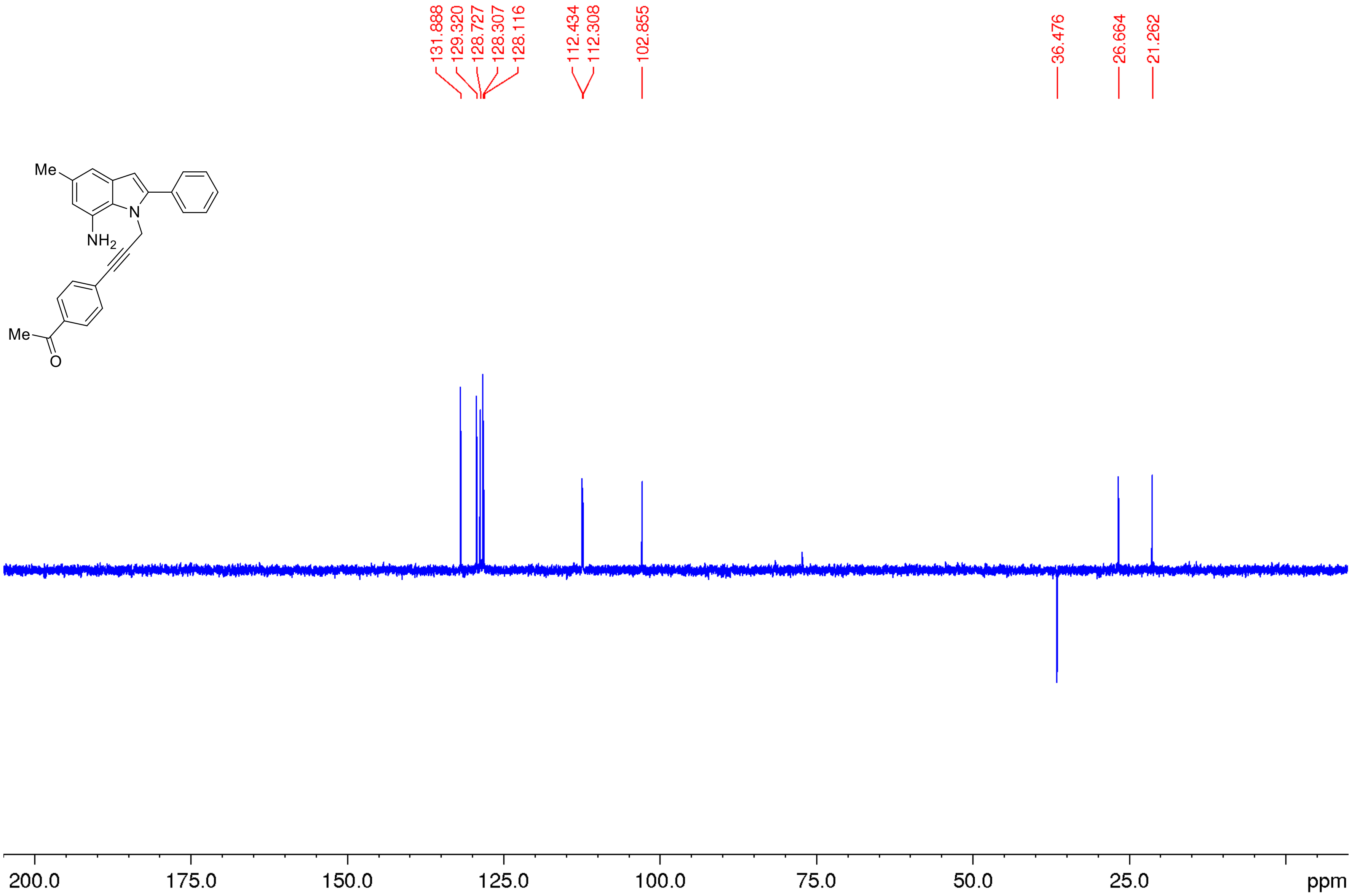

methyl 4-(7-amino-1-(3-(4-chlorophenyl)prop-2-yn-1-yl)-5-methyl-1*H*-indol-2-yl)benzoate 1h

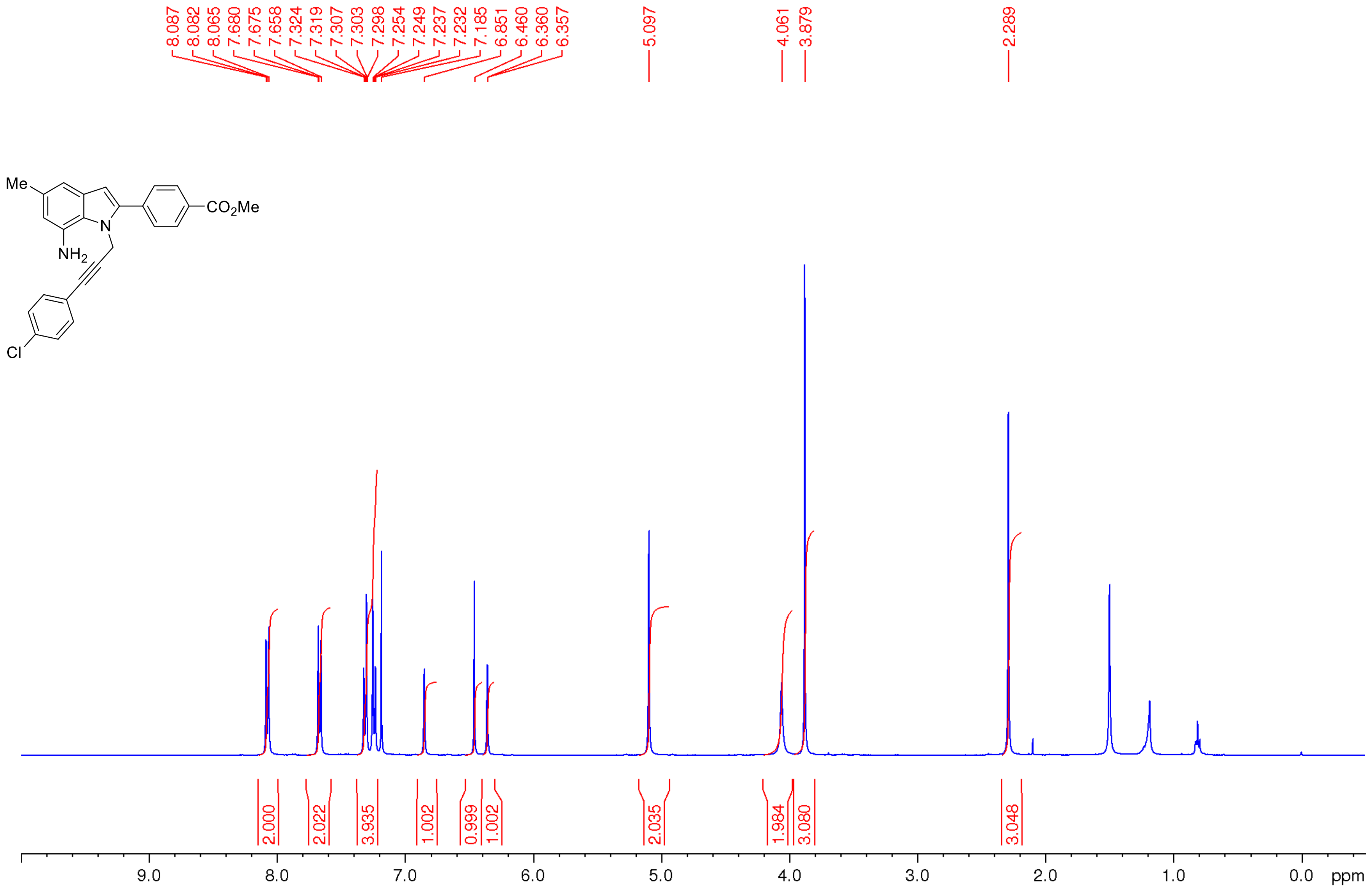

methyl 4-(7-amino-1-(3-(4-chlorophenyl)prop-2-yn-1-yl)-5-methyl-1*H*-indol-2-yl)benzoate 1h

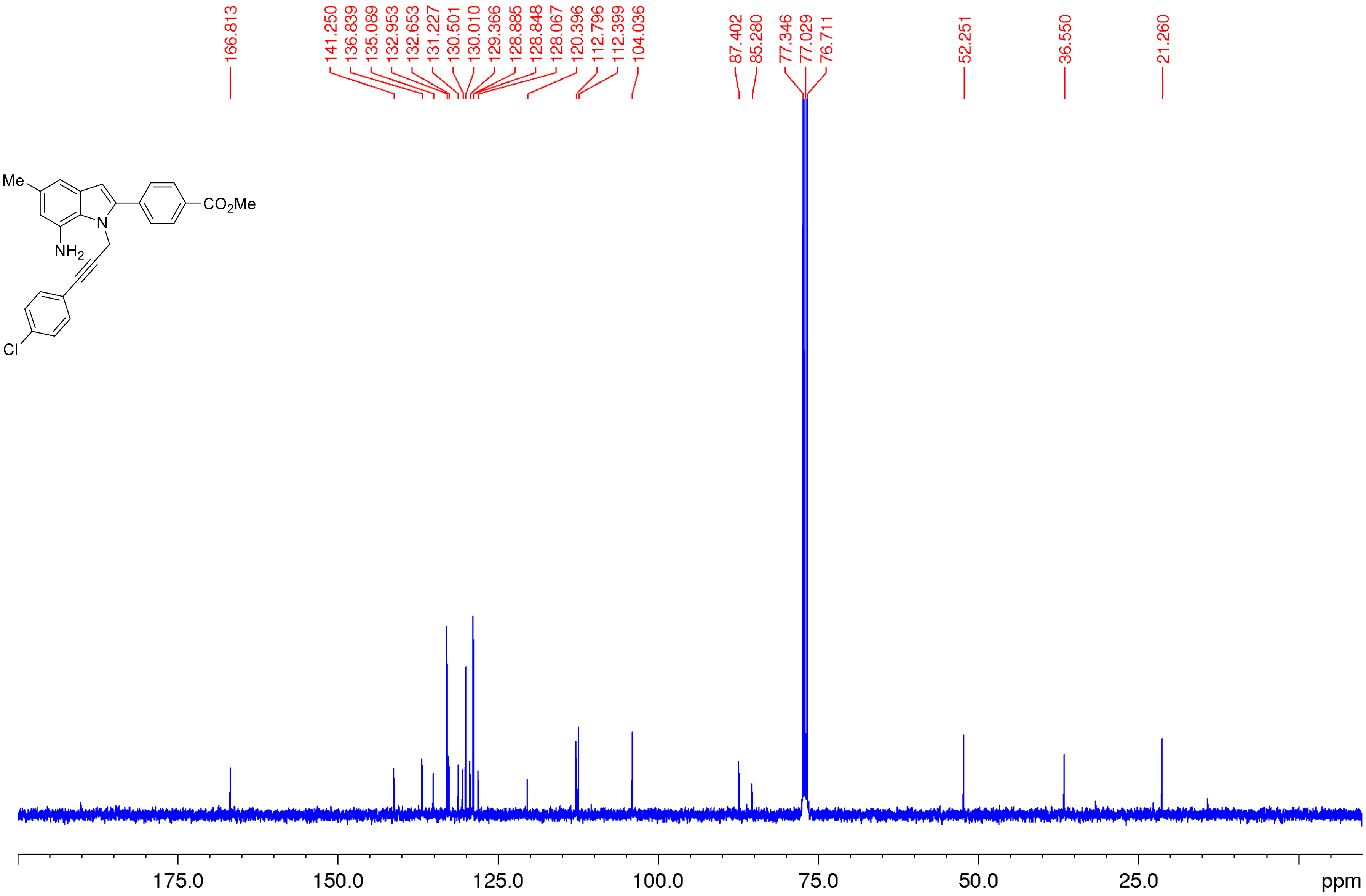

methyl 4-(7-amino-1-(3-(4-chlorophenyl)prop-2-yn-1-yl)-5-methyl-1*H*-indol-2-yl)benzoate 1h

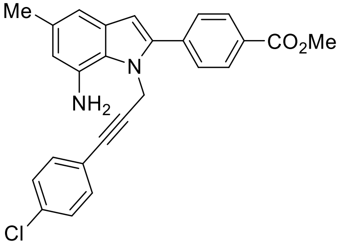

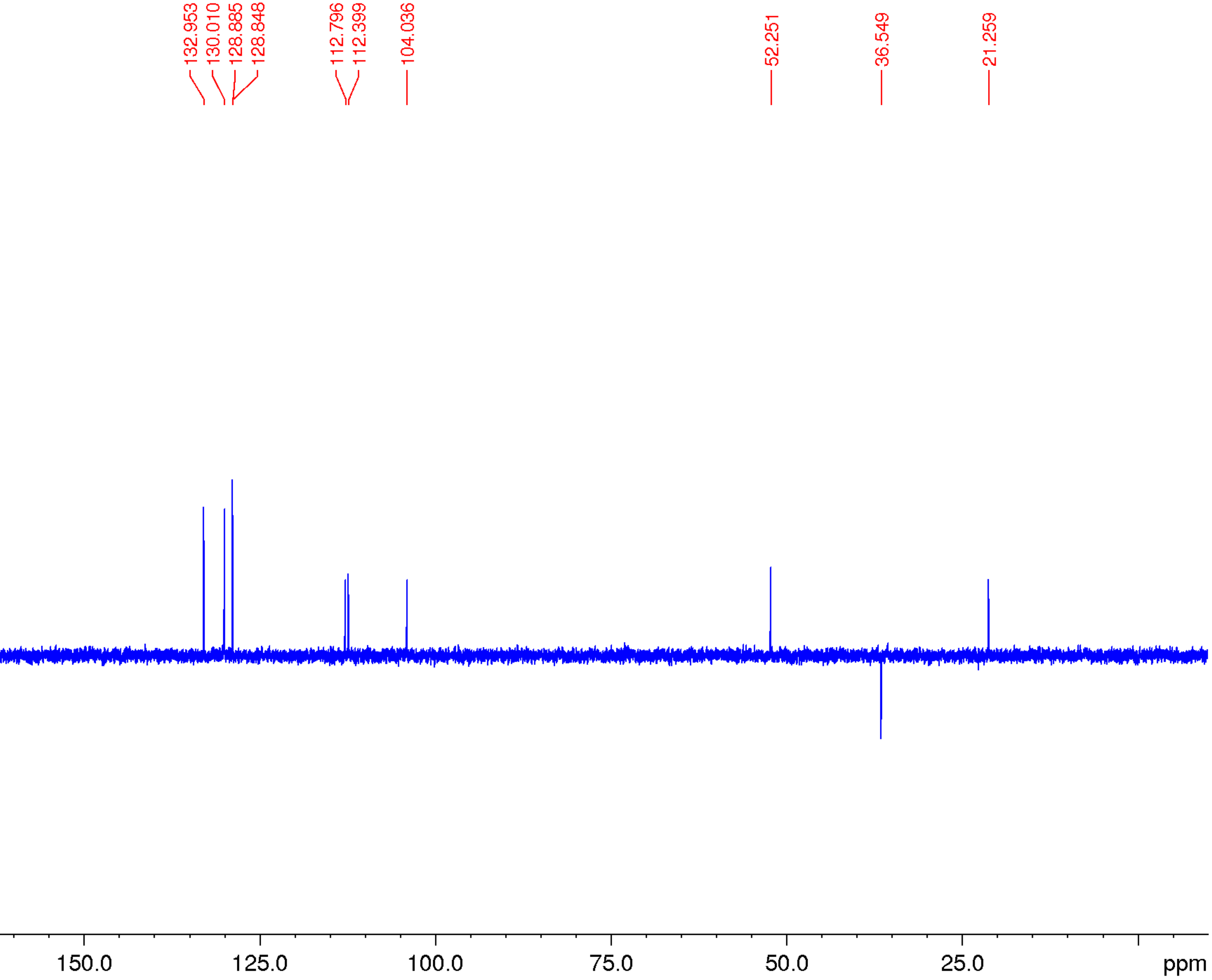

1-(3-(4-chlorophenyl)prop-2-yn-1-yl)-5-methyl-2-phenyl-1*H*-indol-7-amine 1i

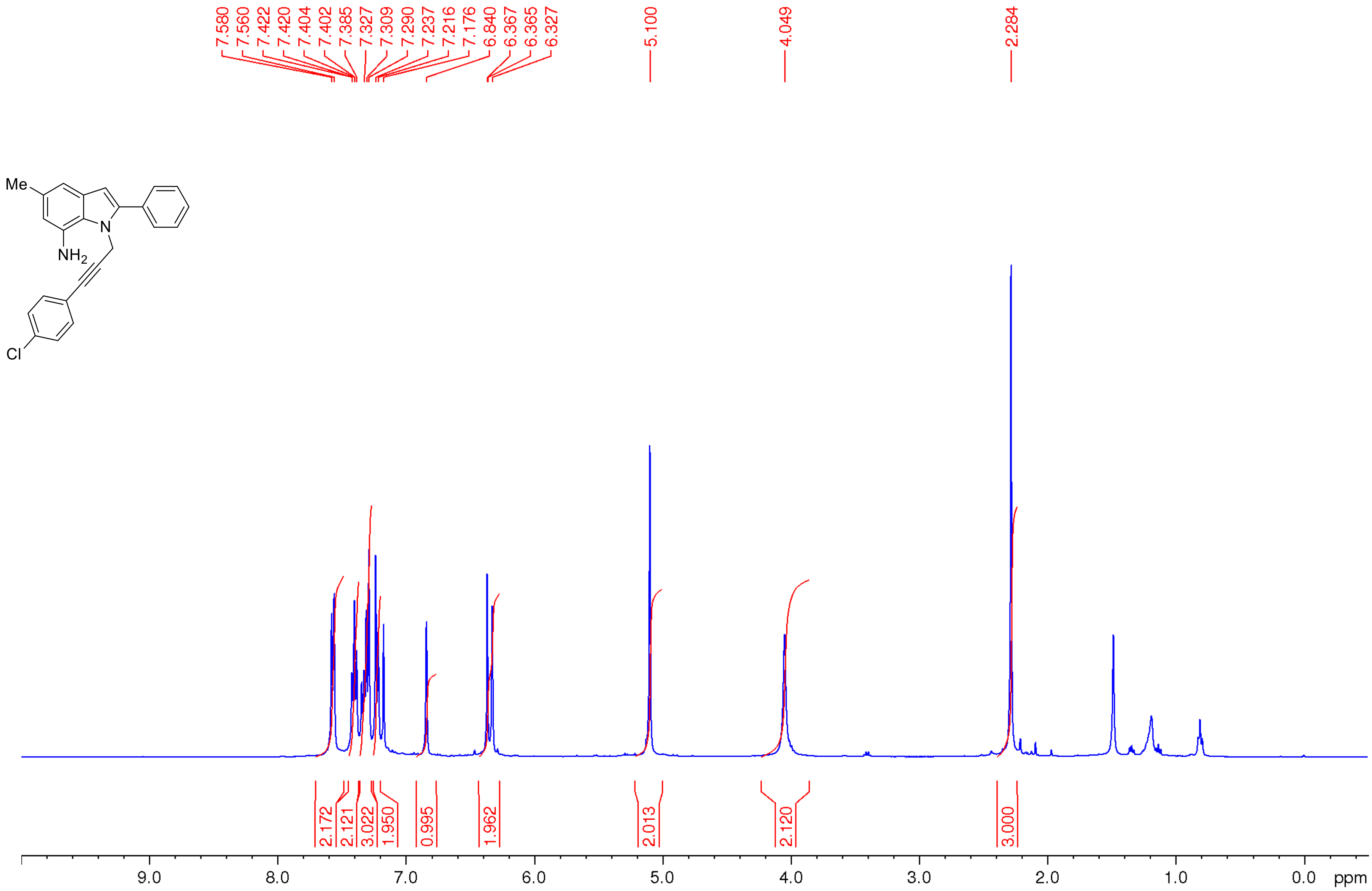

1-(3-(4-chlorophenyl)prop-2-yn-1-yl)-5-methyl-2-phenyl-1*H*-indol-7-amine 1i

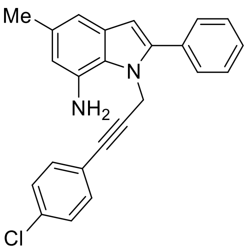

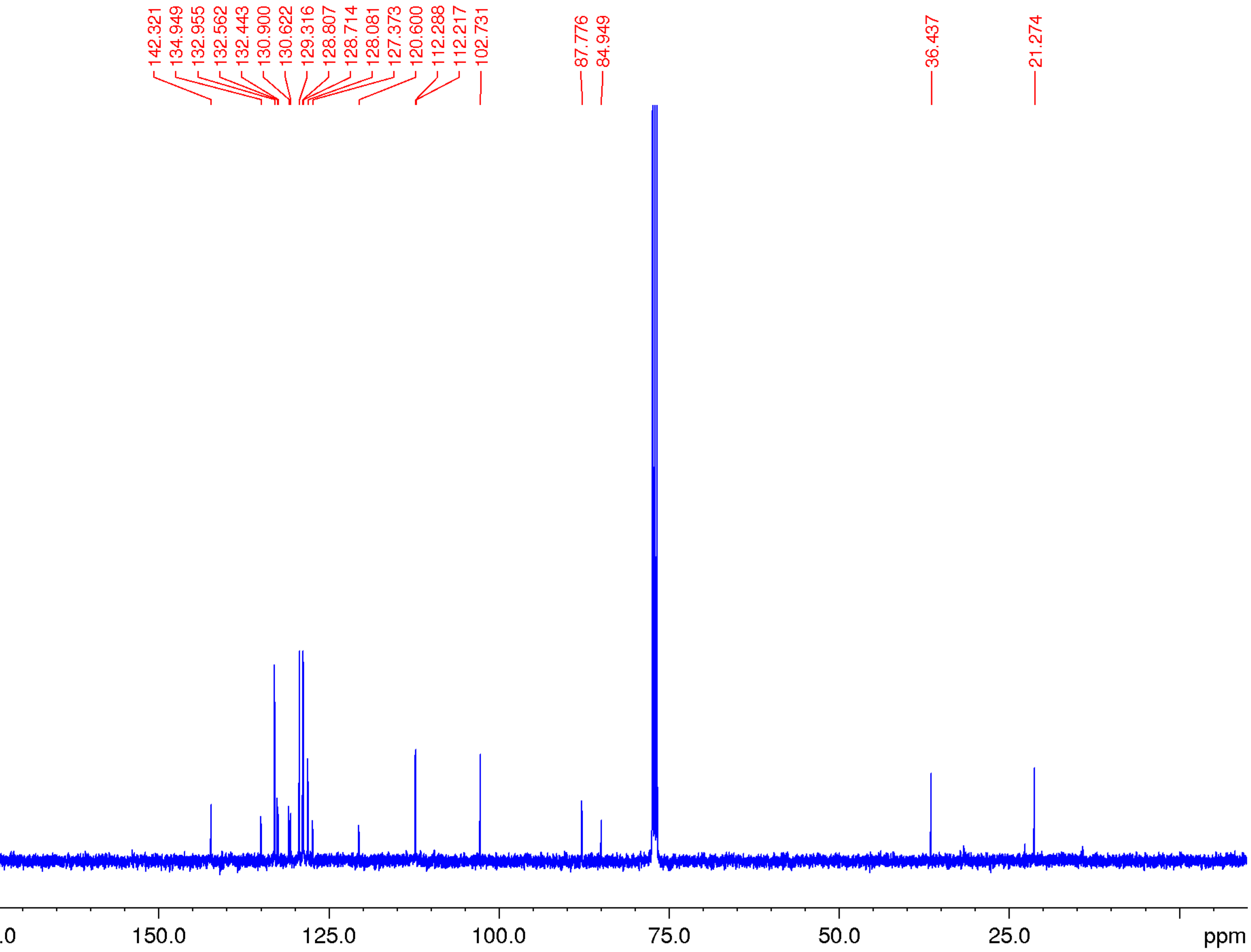

1-(3-(4-chlorophenyl)prop-2-yn-1-yl)-5-methyl-2-phenyl-1*H*-indol-7-amine 1i

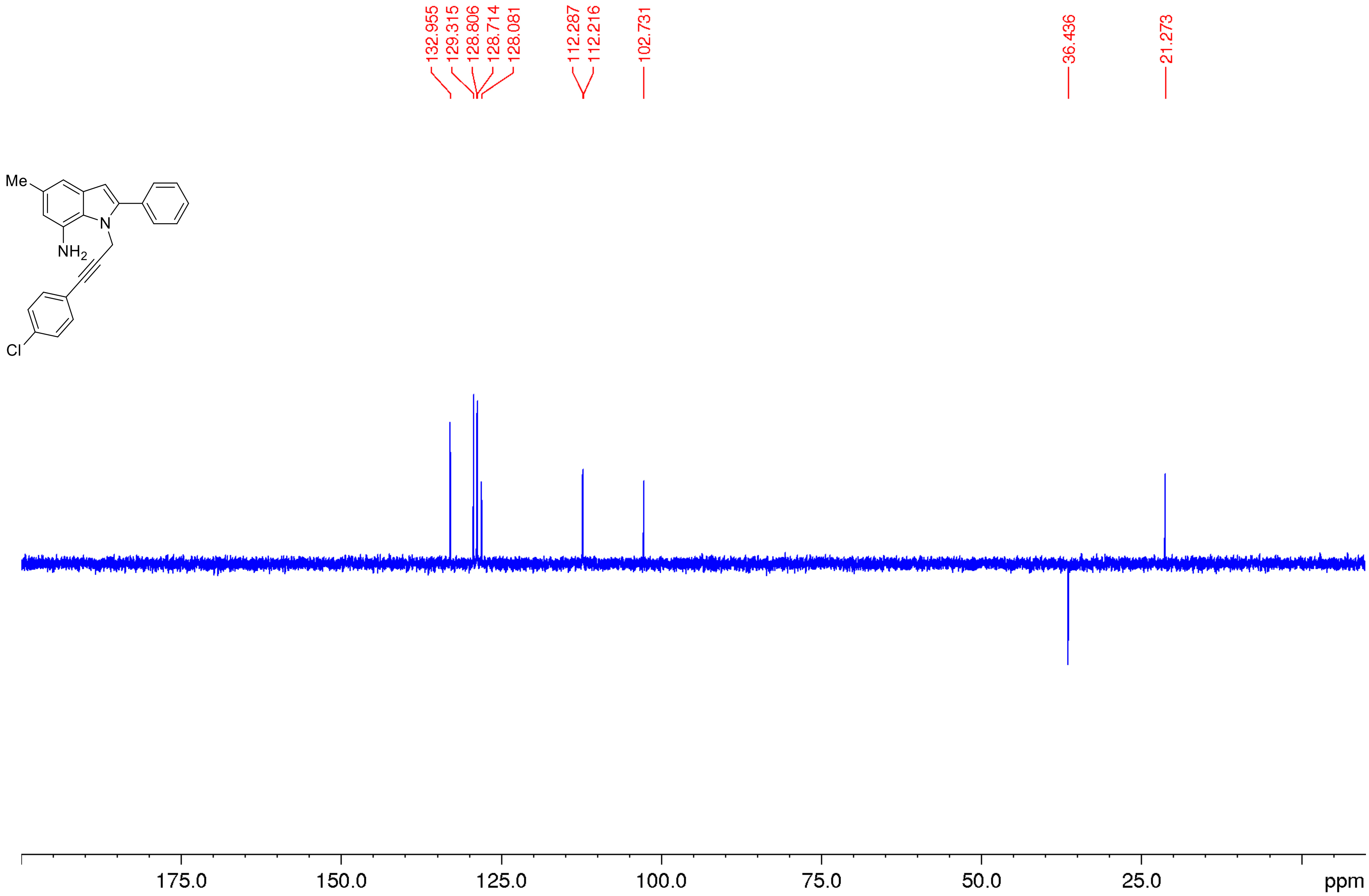

5-methyl-2-phenyl-1-(prop-2-yn-1-yl)-1*H*-indol-7-amine 1j

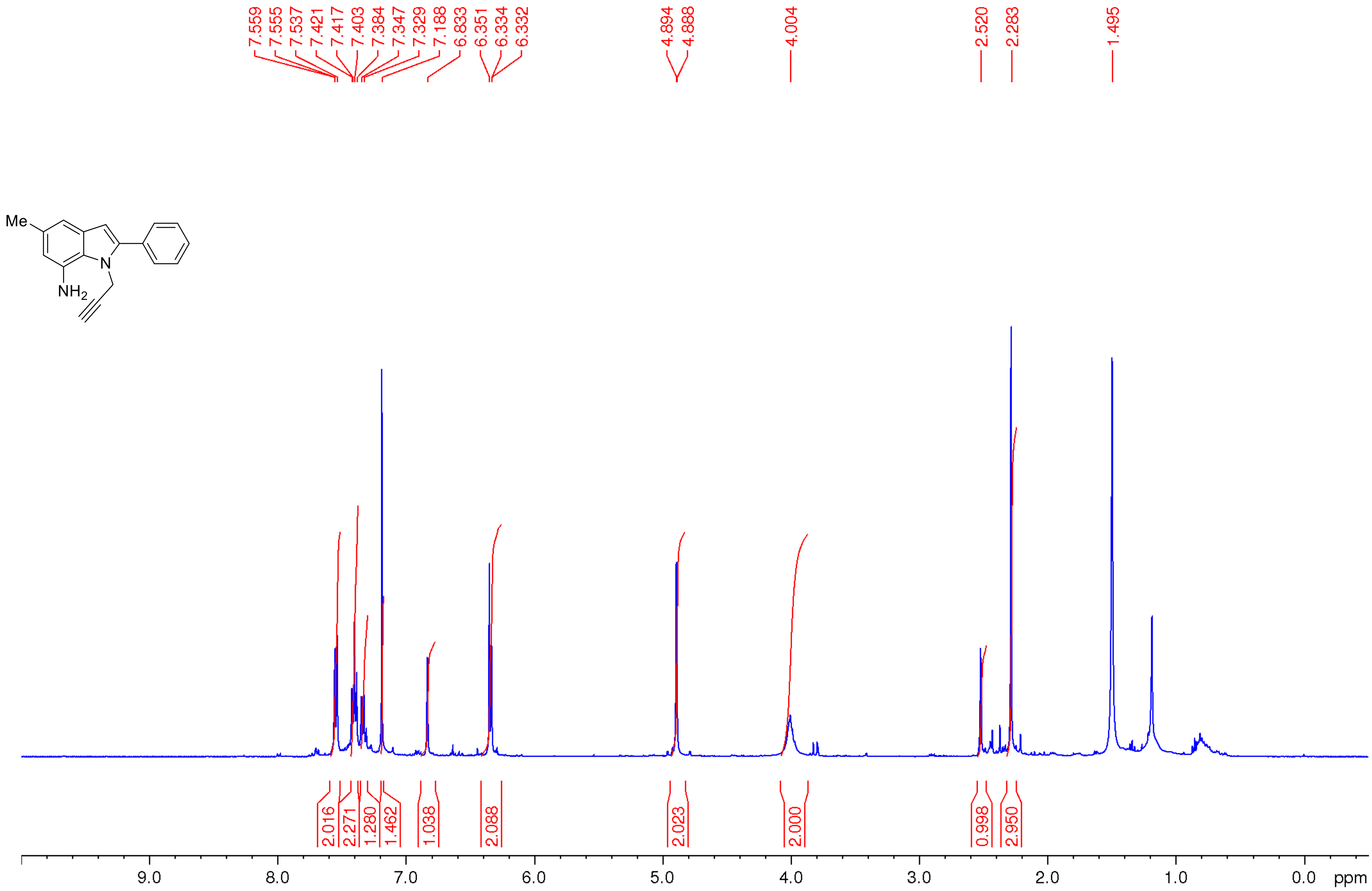

5-methyl-2-phenyl-1-(prop-2-yn-1-yl)-1*H*-indol-7-amine 1j

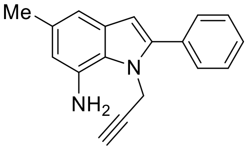

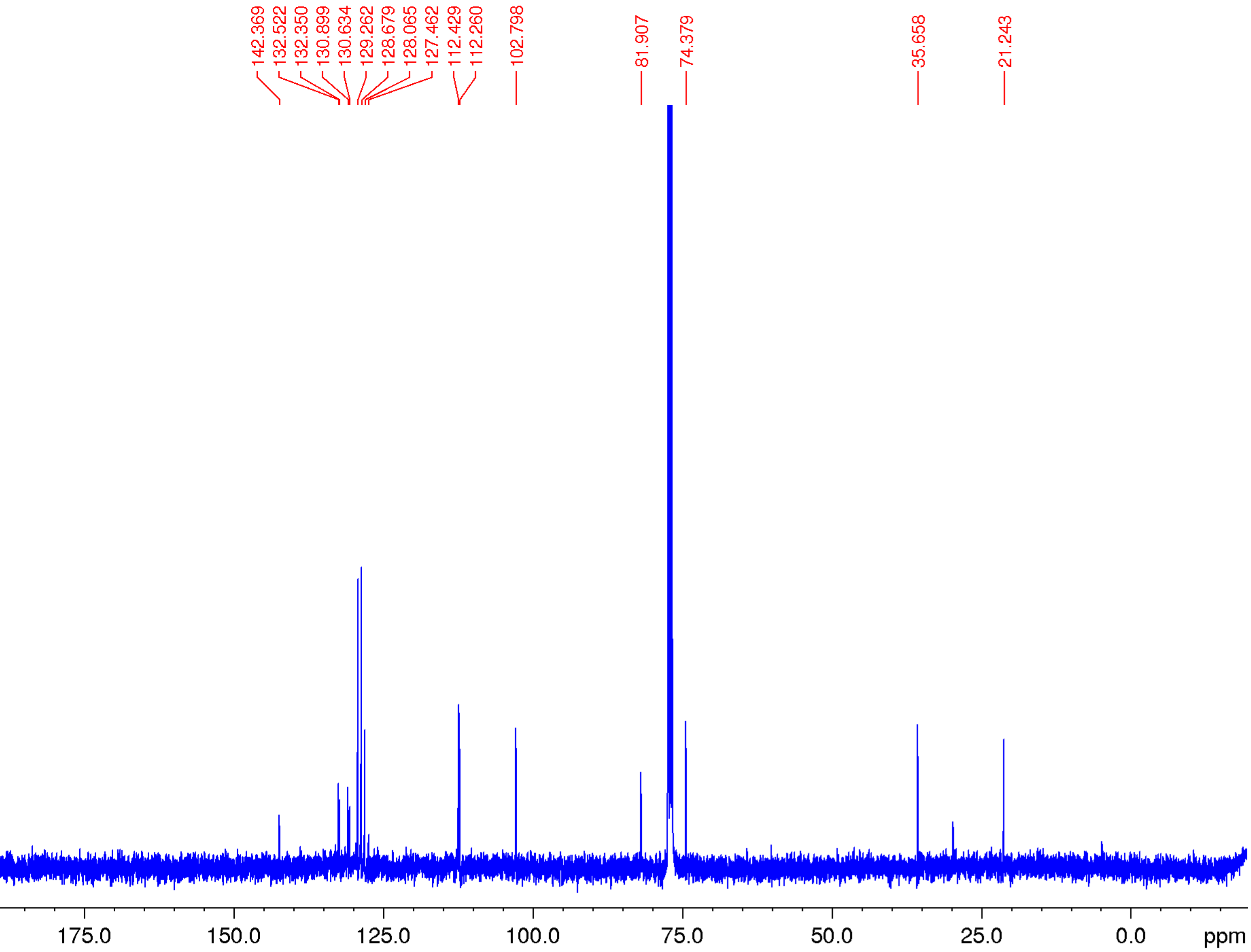

5-methyl-2-phenyl-1-(prop-2-yn-1-yl)-1*H*-indol-7-amine 1j

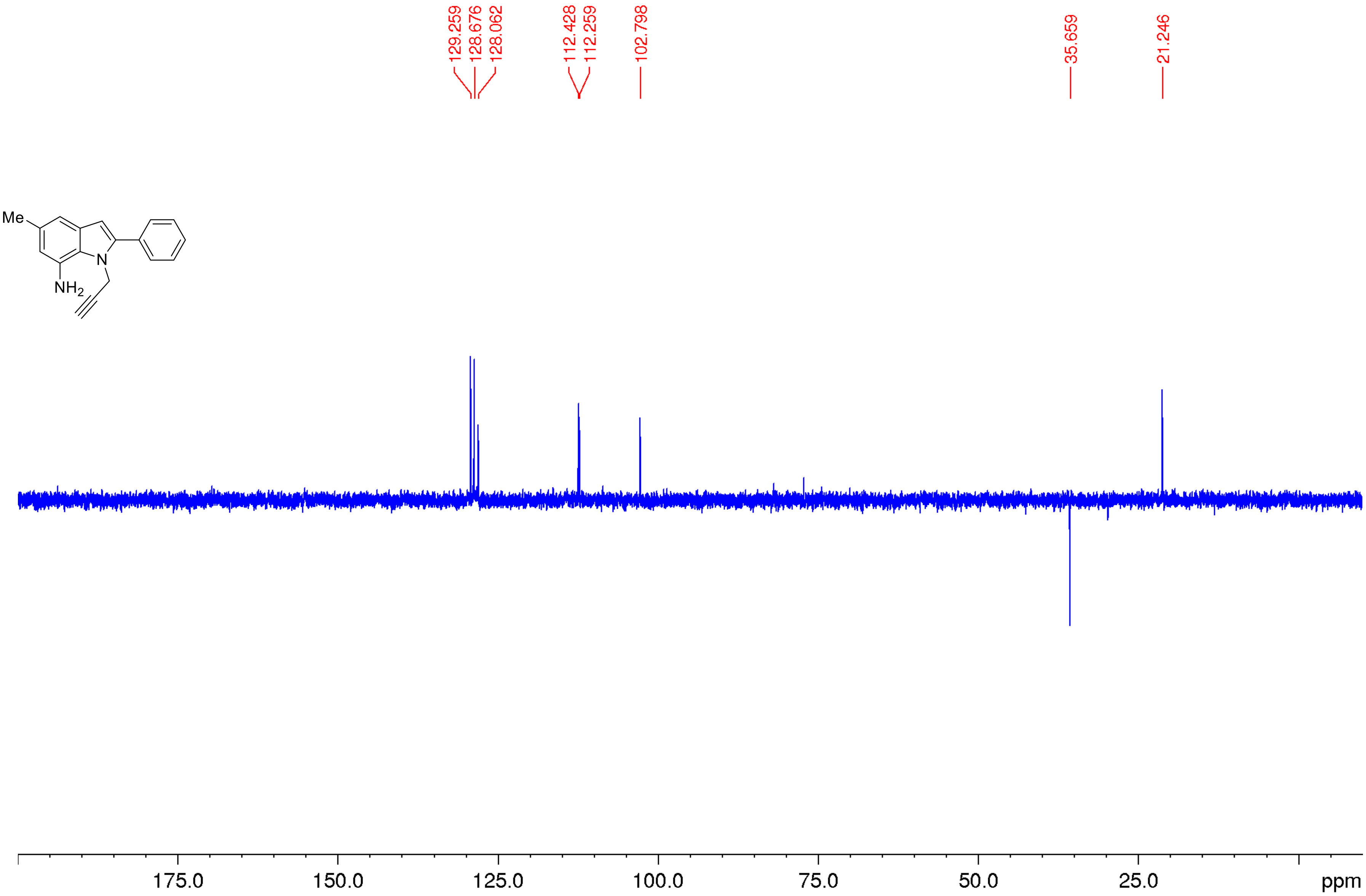

1-(4-(3-(7-amino-3-(4-methoxyphenyl)-5-methyl-2-phenyl-1*H*-indol-1-yl)prop-1-yn-1-yl)phenyl)ethan-1-one 1k

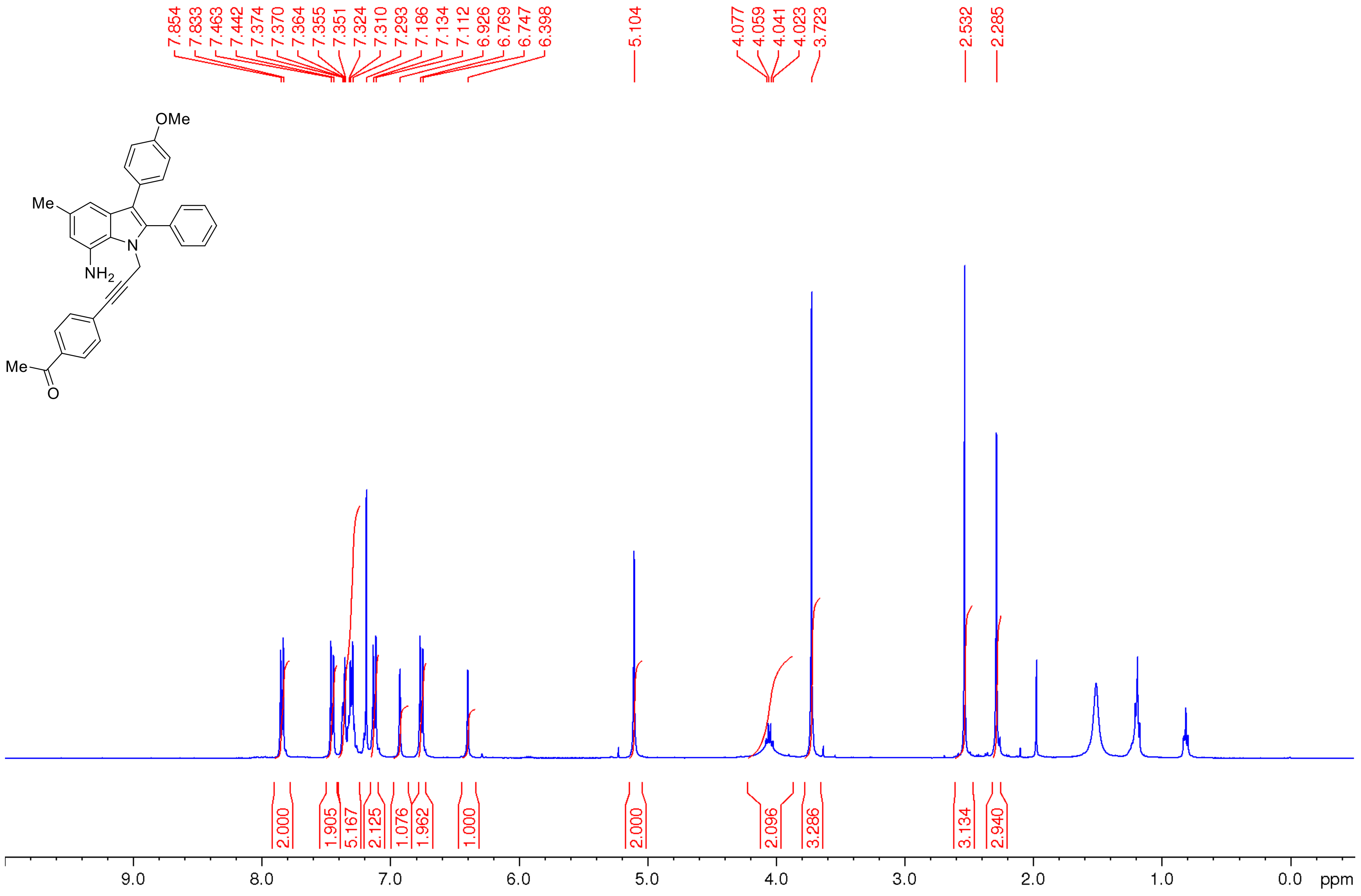

1-(4-(3-(7-amino-3-(4-methoxyphenyl)-5-methyl-2-phenyl-1*H*-indol-1-yl)prop-1-yn-1-yl)phenyl)ethan-1-one 1k

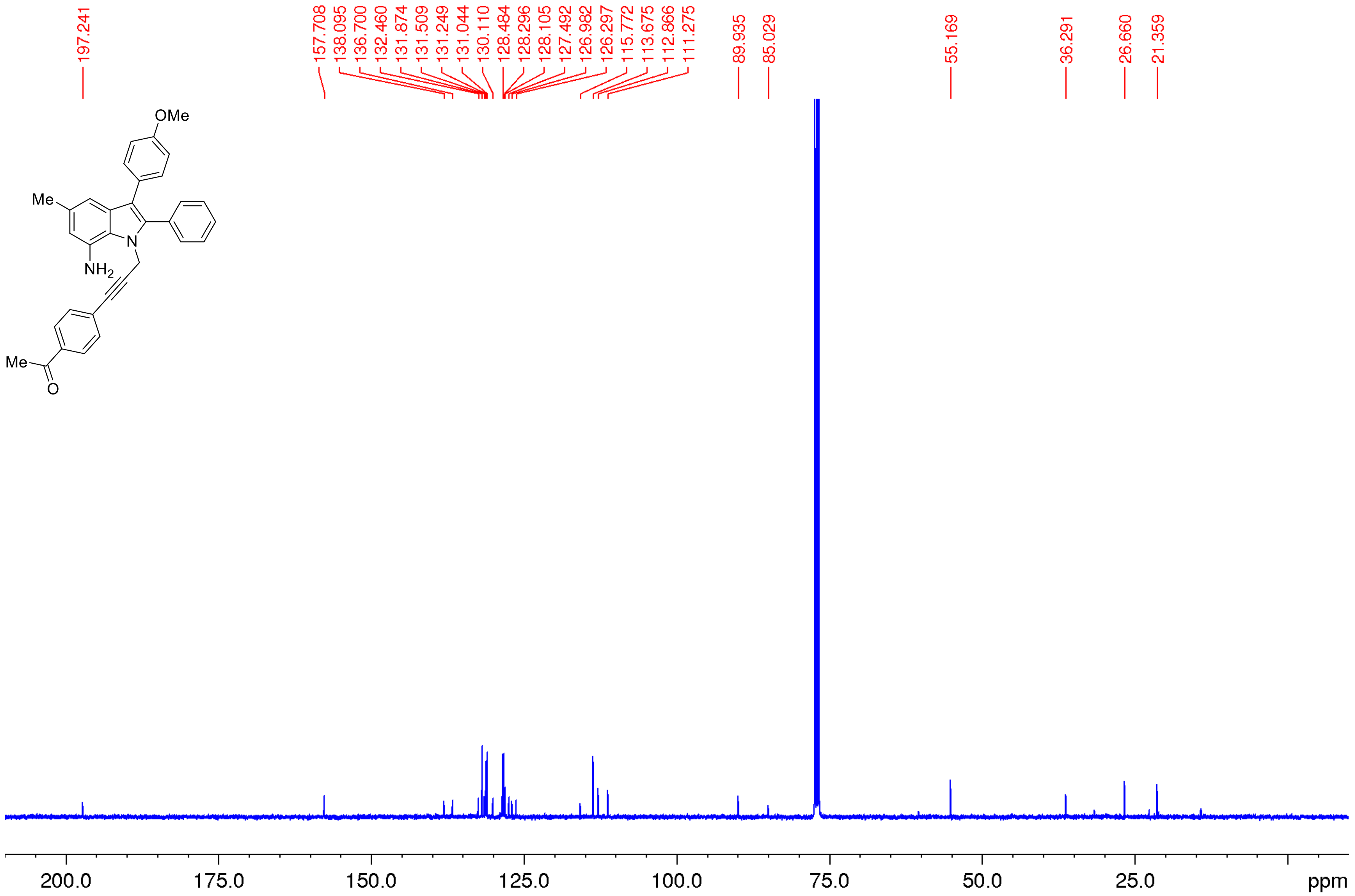

1-(4-(3-(7-amino-3-(4-methoxyphenyl)-5-methyl-2-phenyl-1*H*-indol-1-yl)prop-1-yn-1-yl)phenyl)ethan-1-one 1k

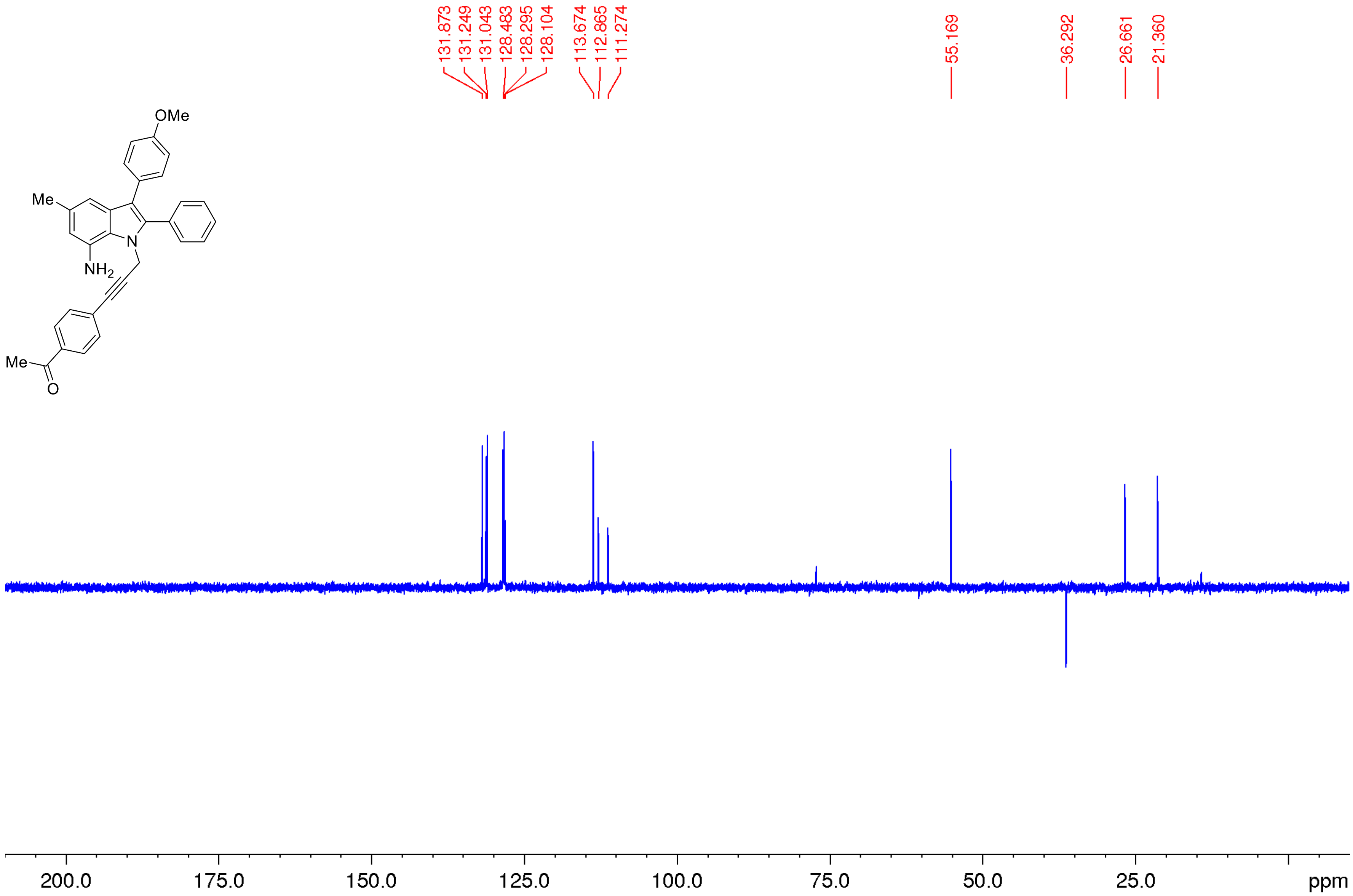

3-iodo-5-methyl-7-nitro-2-phenyl-1*H*-indole 11

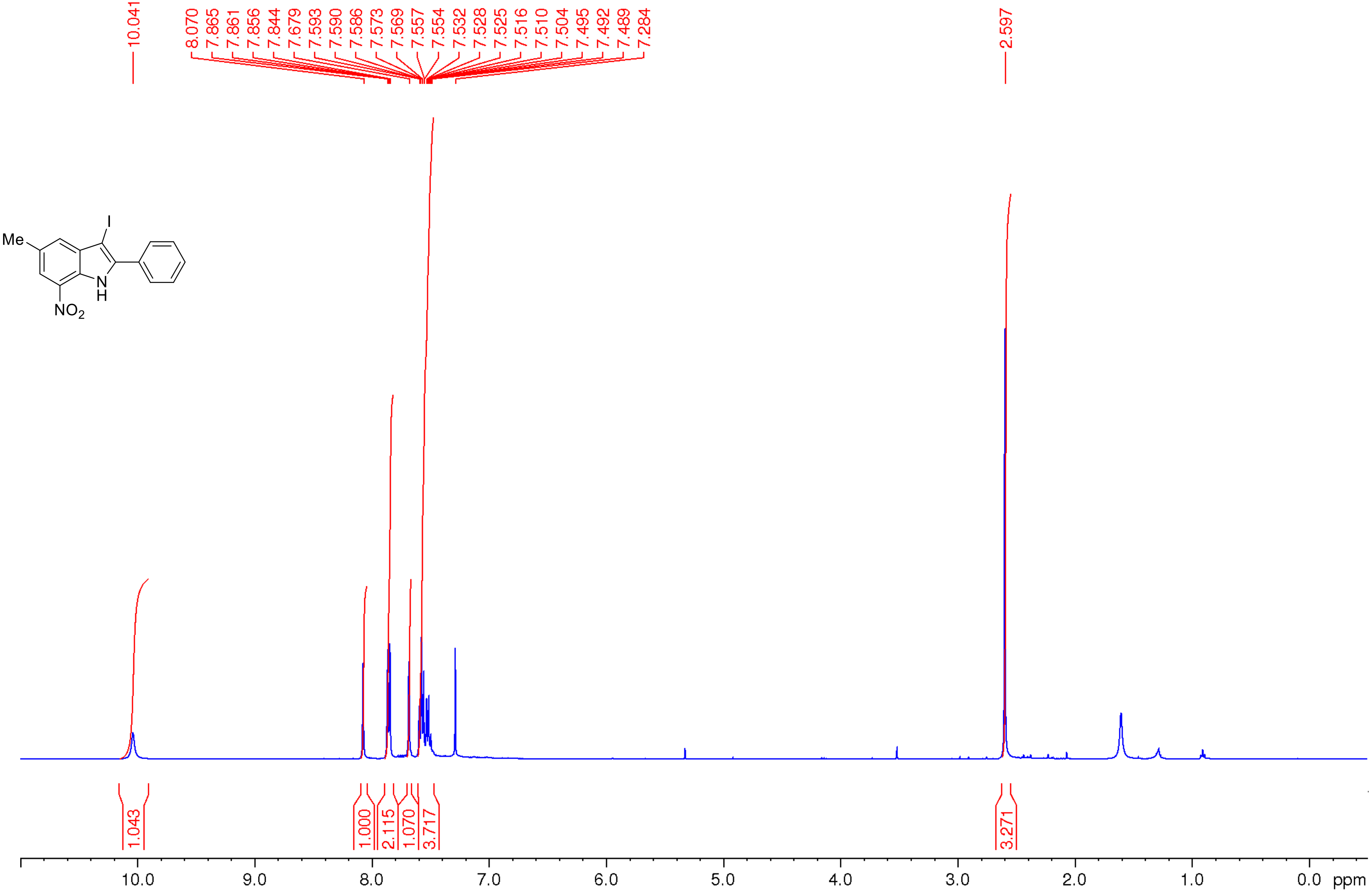

3-iodo-5-methyl-7-nitro-2-phenyl-1*H*-indole 11

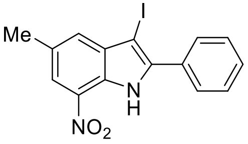

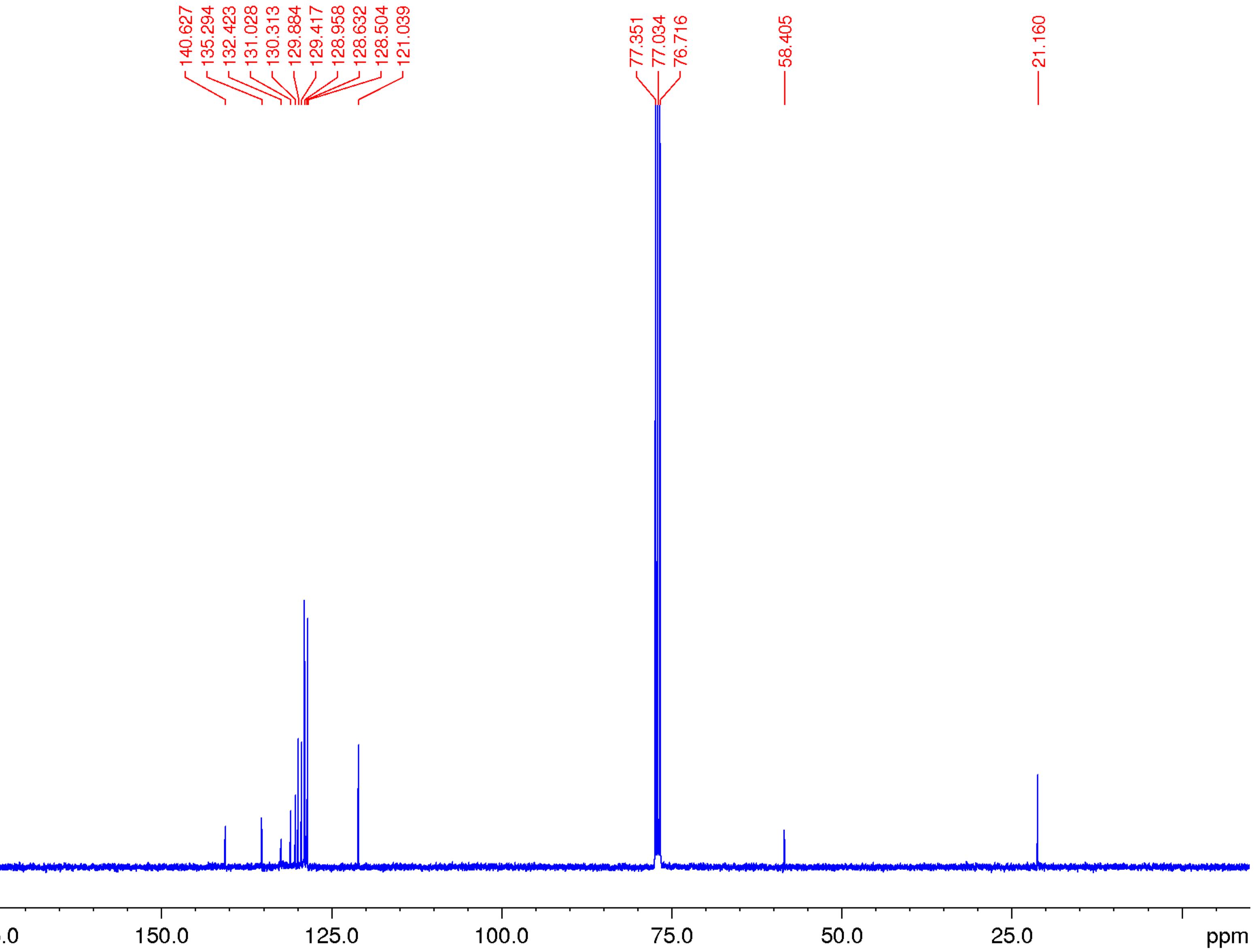

3-iodo-5-methyl-7-nitro-2-phenyl-1*H*-indole

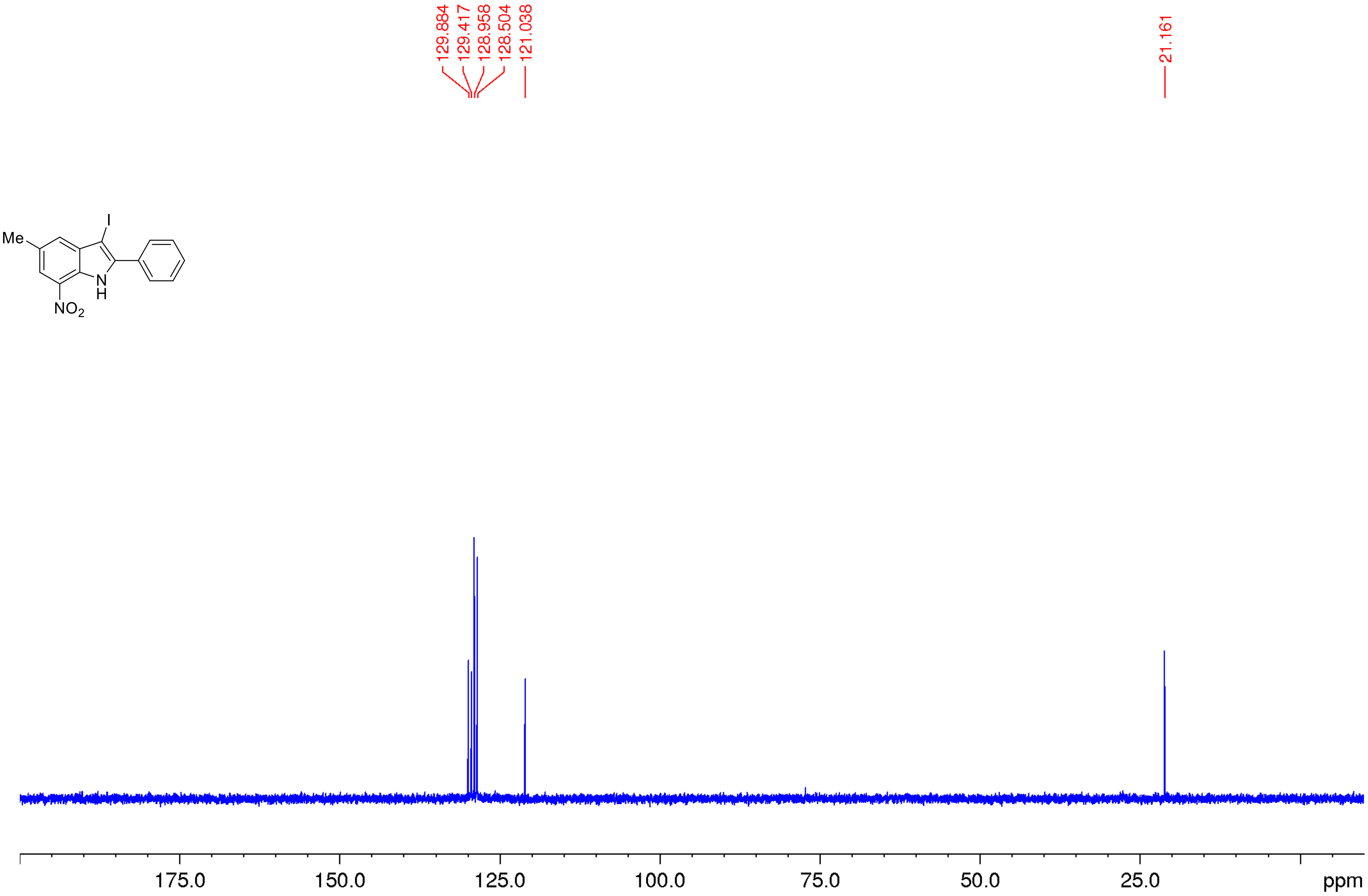

3-(4-methoxyphenyl)-5-methyl-7-nitro-2-phenyl-1*H*-indole 12

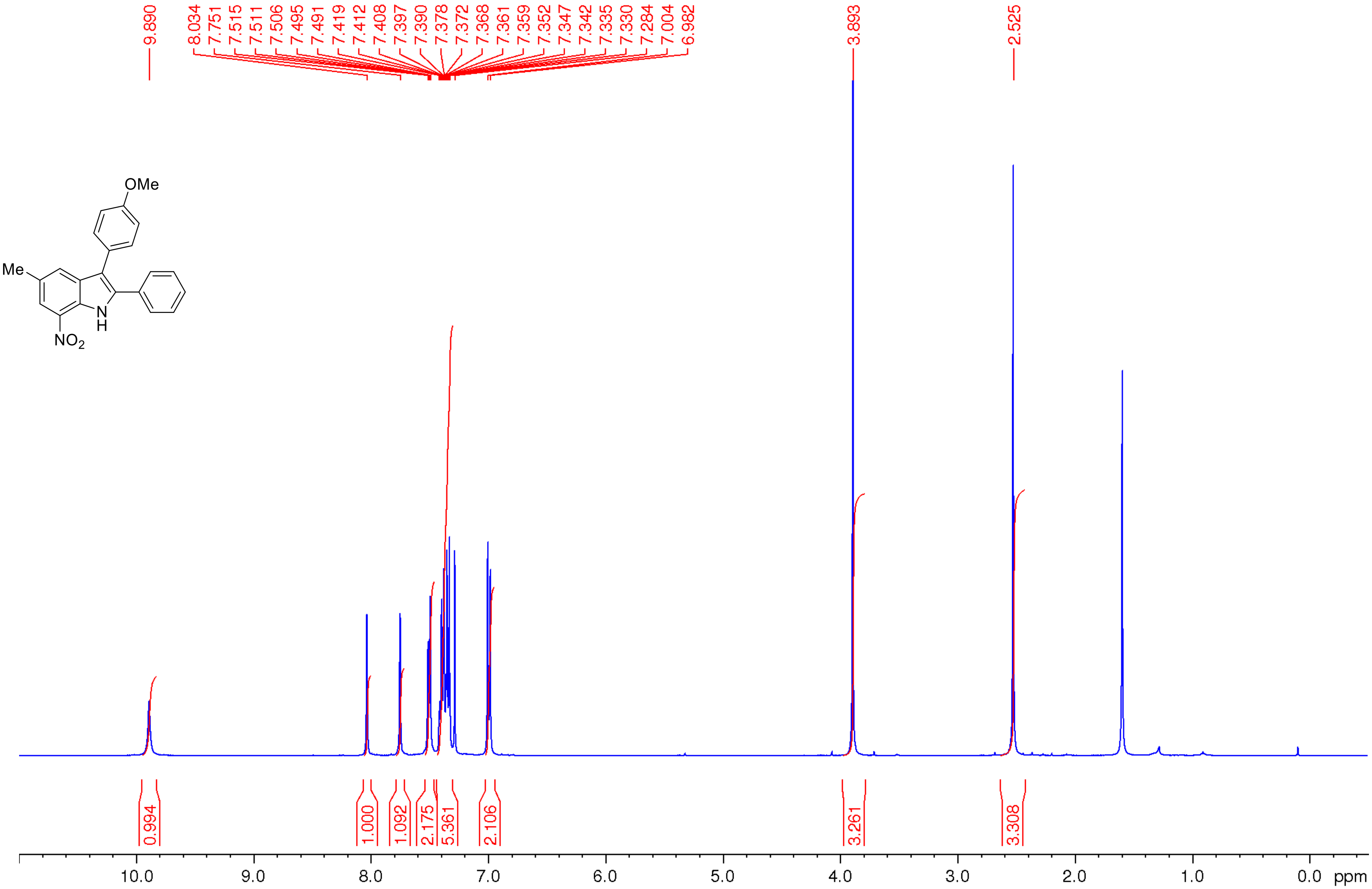

3-(4-methoxyphenyl)-5-methyl-7-nitro-2-phenyl-1*H*-indole 12

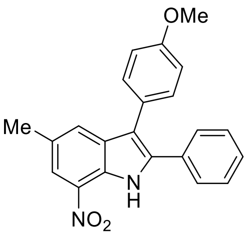

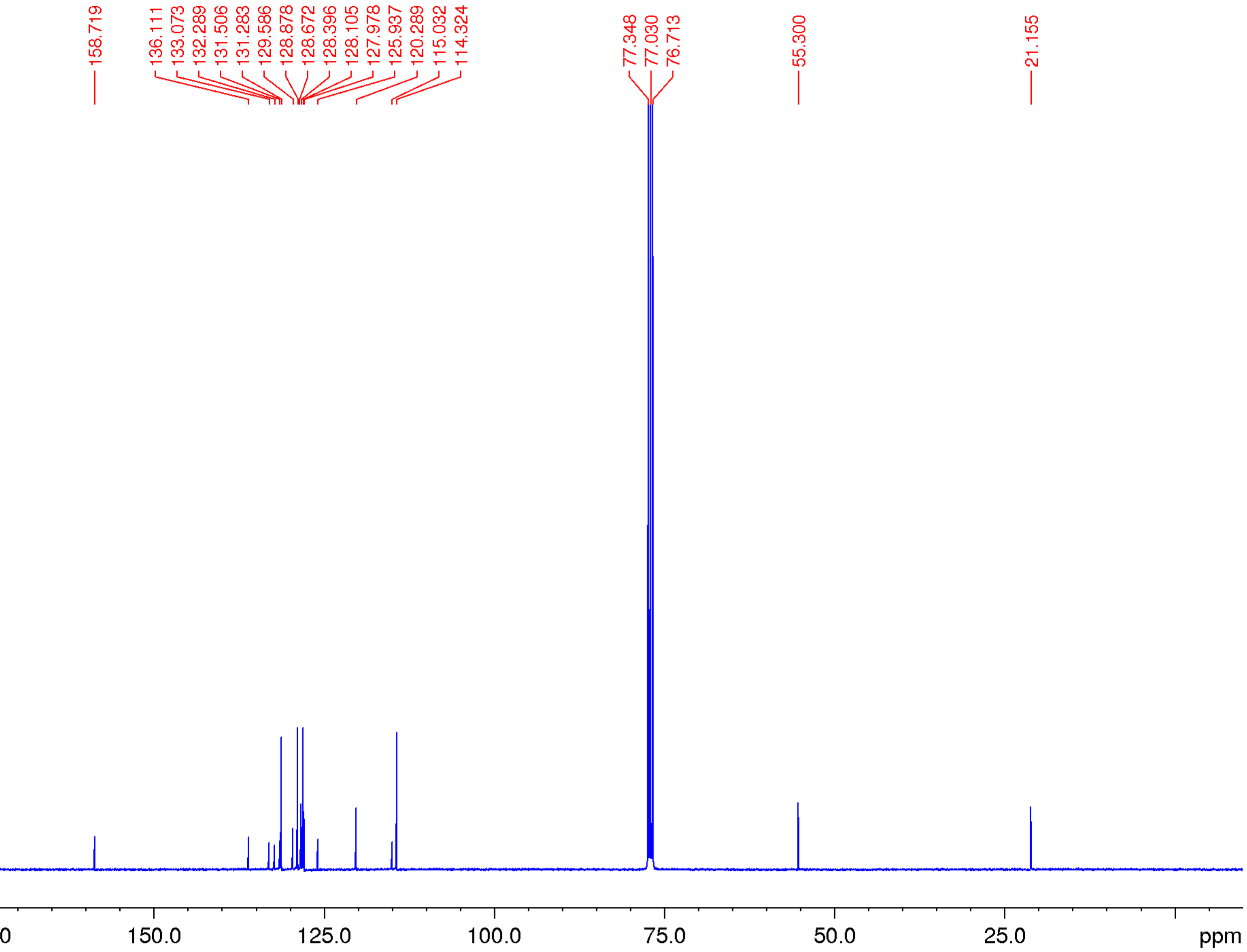

3-(4-methoxyphenyl)-5-methyl-7-nitro-2-phenyl-1*H*-indole 12

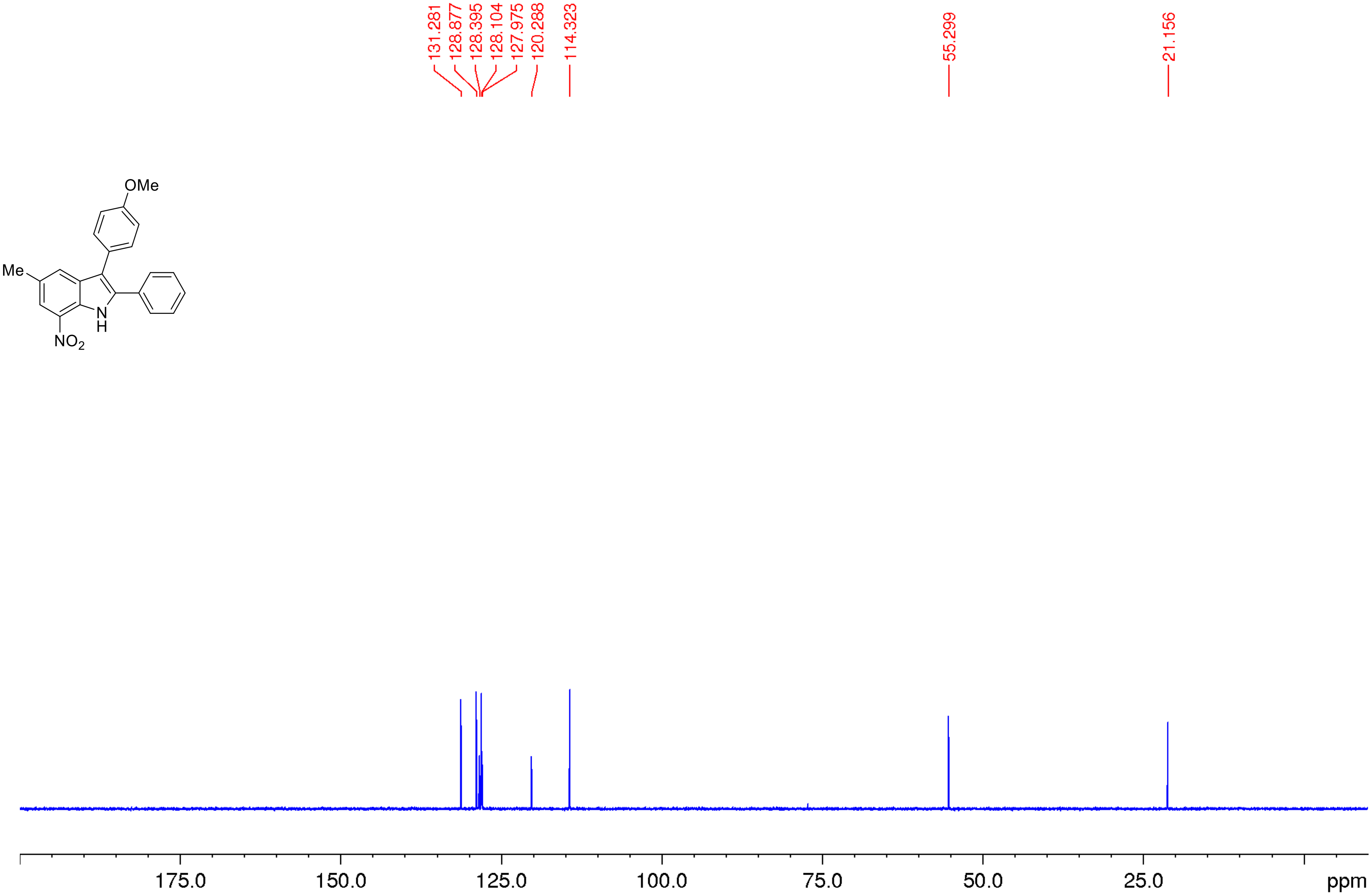

3-(4-methoxyphenyl)-5-methyl-7-nitro-2-phenyl-1-(prop-2-yn-1-yl)-1*H*-indole

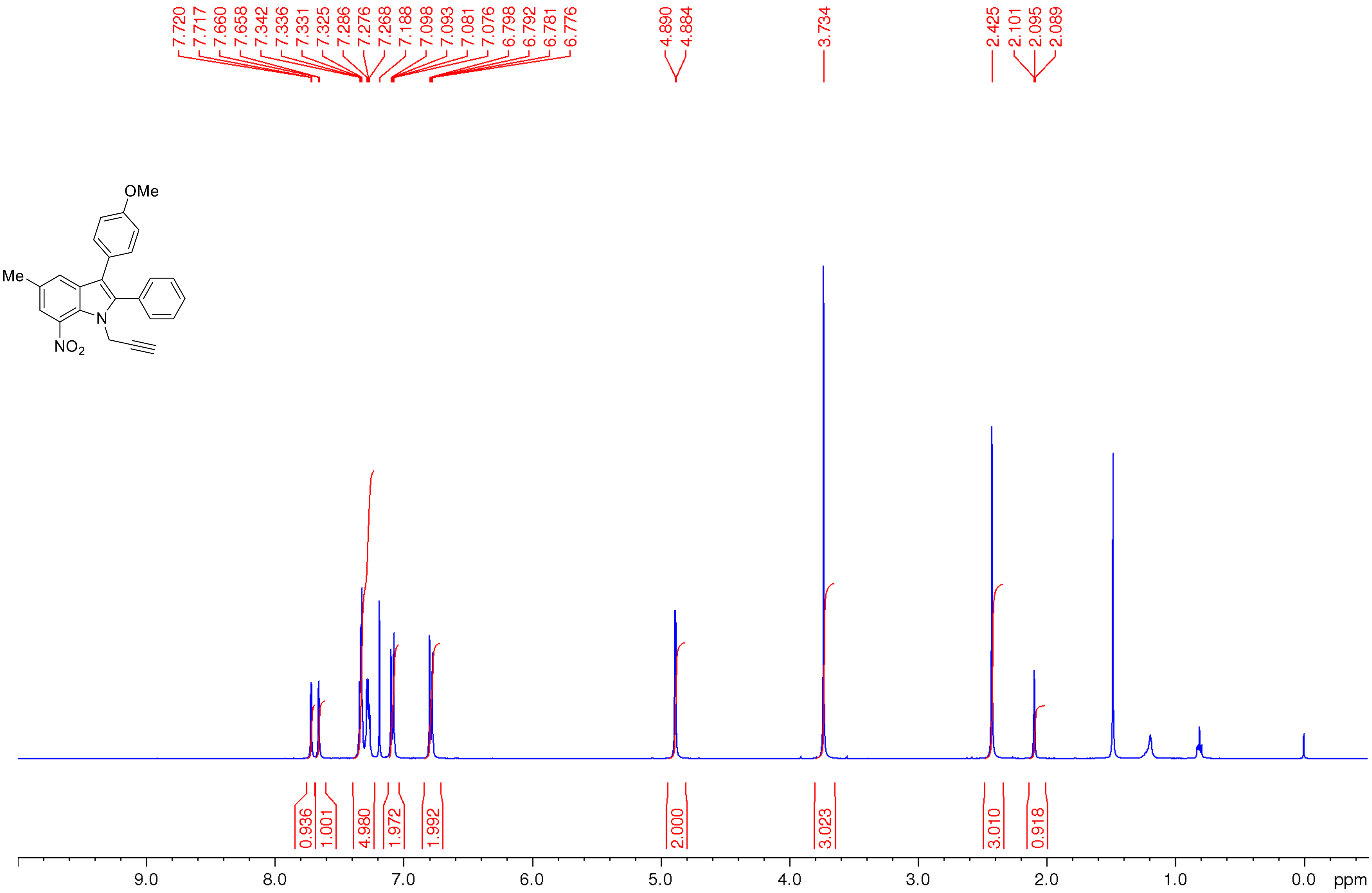

3-(4-methoxyphenyl)-5-methyl-7-nitro-2-phenyl-1-(prop-2-yn-1-yl)-1*H*-indole 13

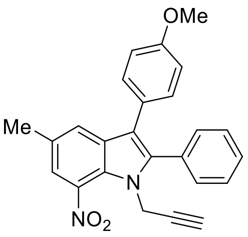

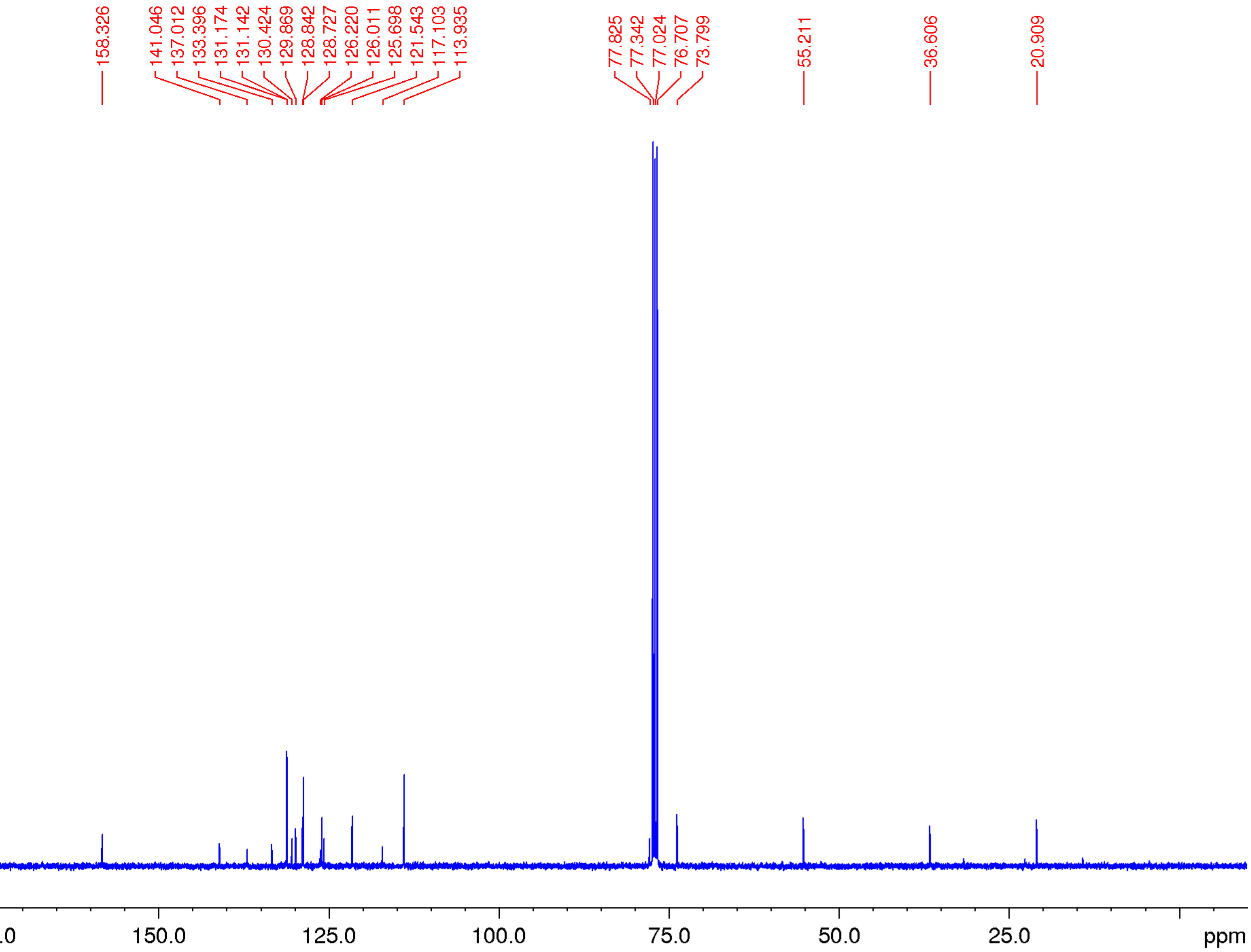

3-(4-methoxyphenyl)-5-methyl-7-nitro-2-phenyl-1-(prop-2-yn-1-yl)-1*H*-indole 13

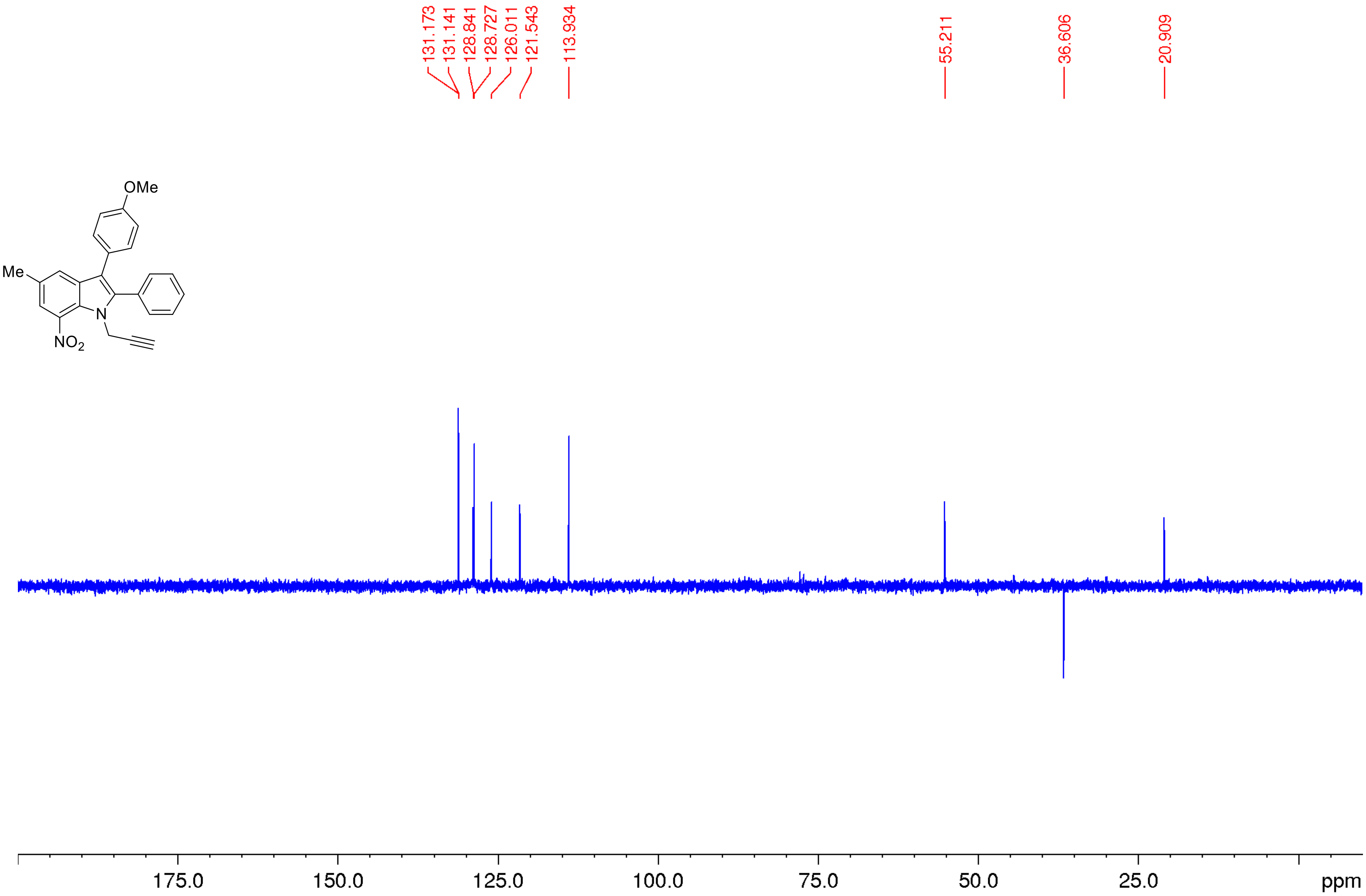

1-(4-(3-(3-(4-methoxyphenyl)-5-methyl-7-nitro-2-phenyl-1*H*-indol-1-yl)prop-1-yn-1-yl)phenyl)ethan-1-one 14

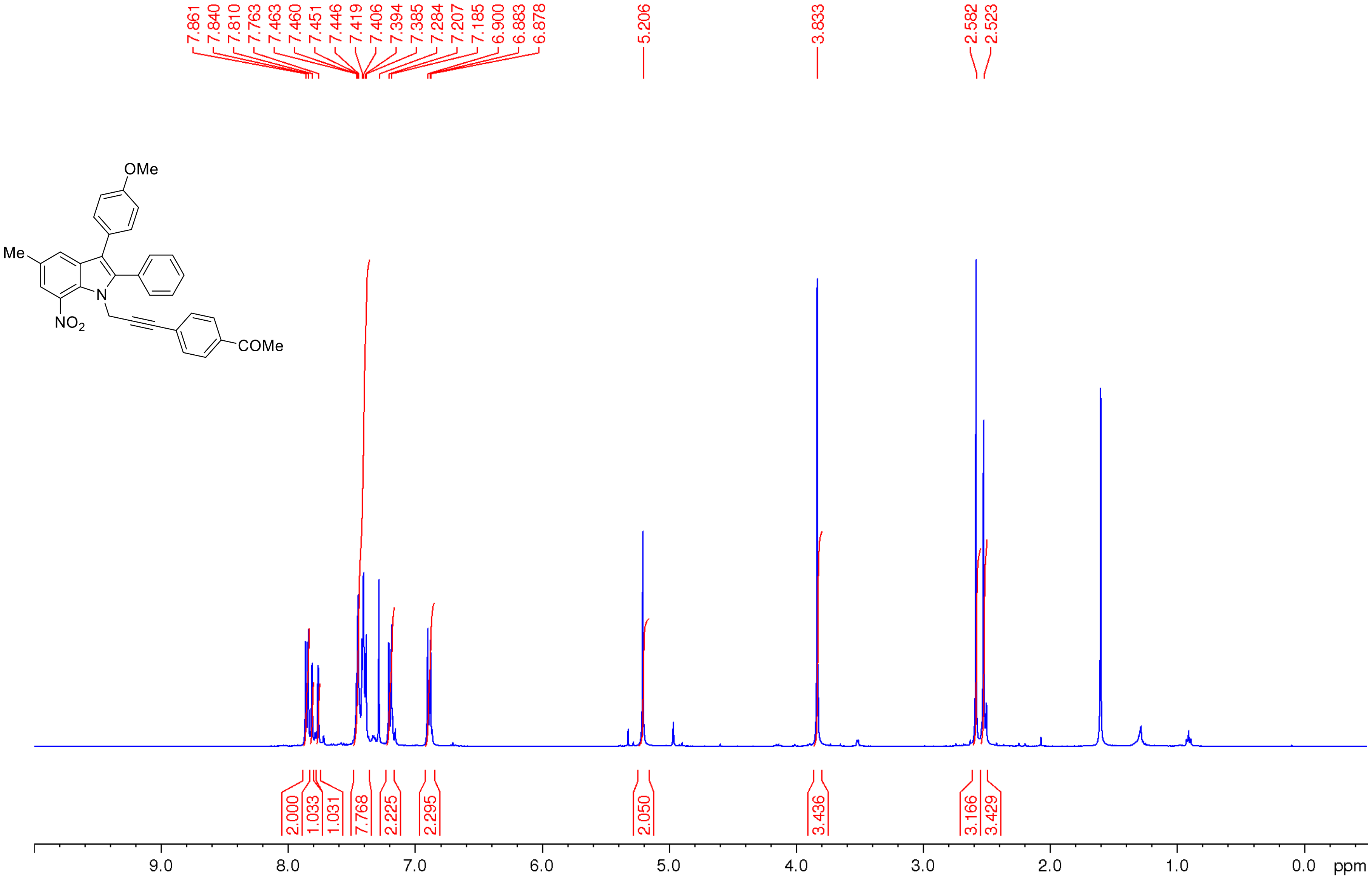

1-(4-(3-(3-(4-methoxyphenyl)-5-methyl-7-nitro-2-phenyl-1*H*-indol-1-yl)prop-1-yn-1-yl)phenyl)ethan-1-one 14

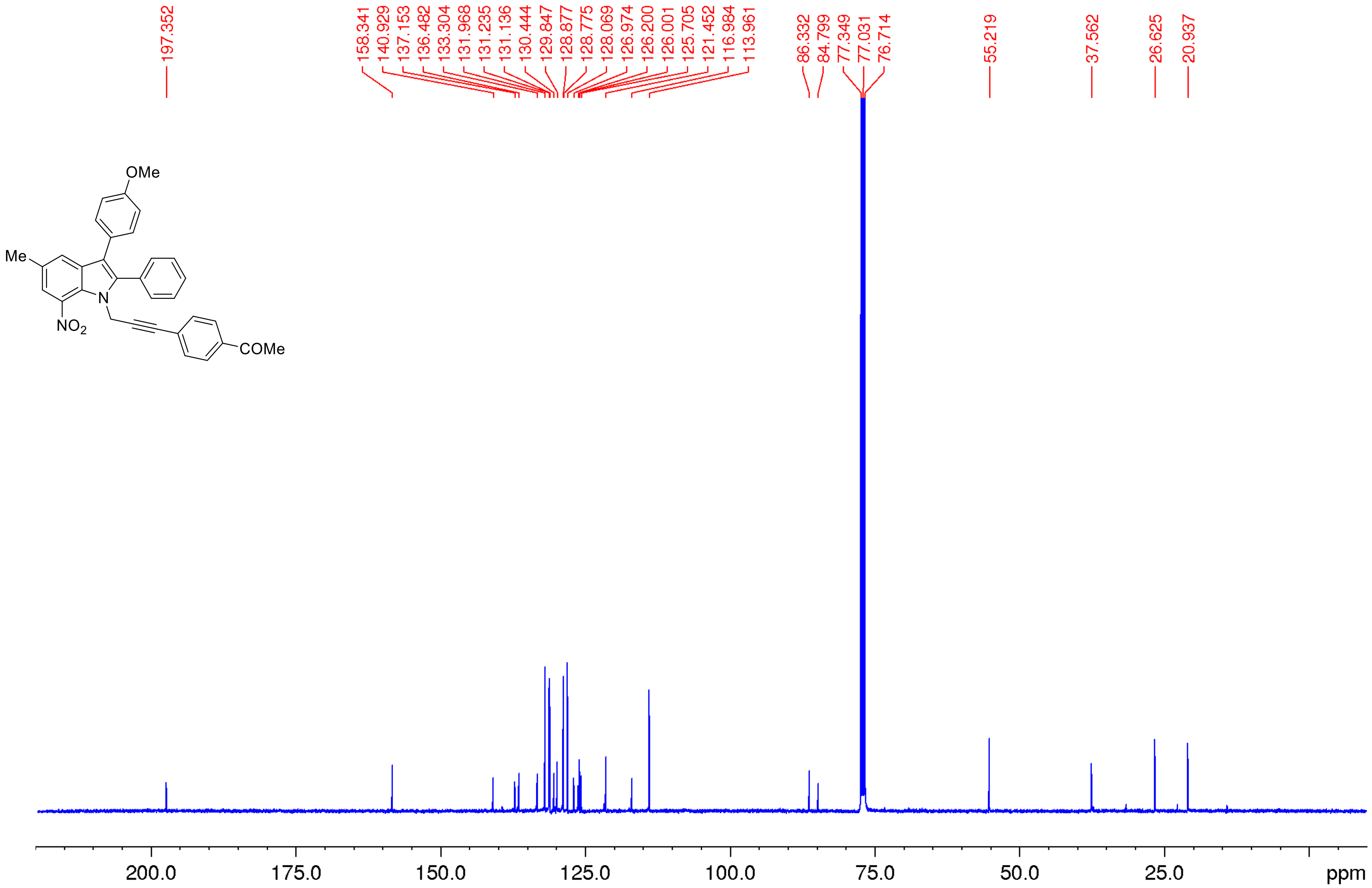

1-(4-(3-(3-(4-methoxyphenyl)-5-methyl-7-nitro-2-phenyl-1*H*-indol-1-yl)prop-1-yn-1-yl)phenyl)ethan-1-one 14

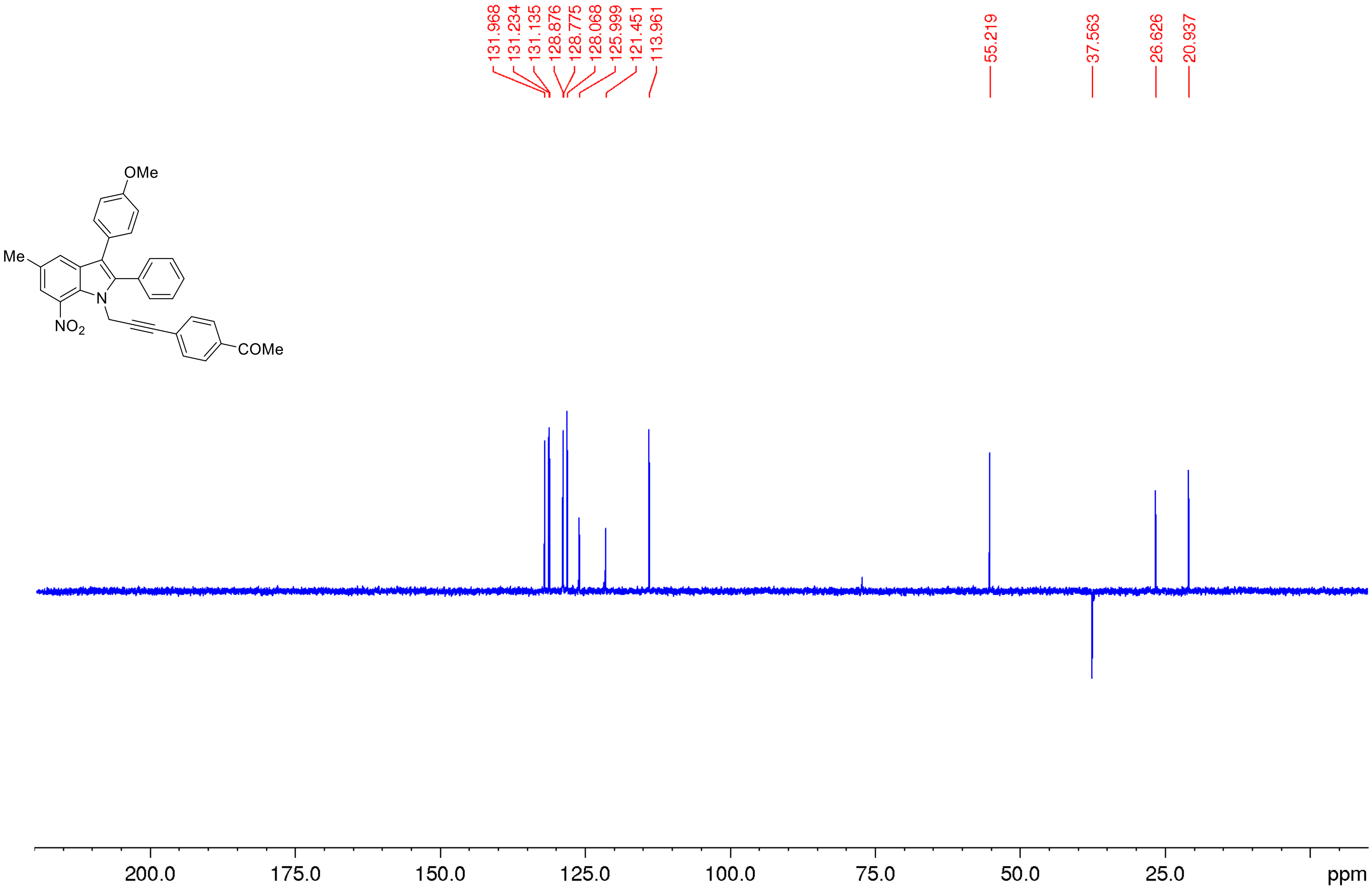

Supplement: Supplementary file 1 [file molecules-28-05831-s001.zip › molecules-2527500-supplementary.pdf]
